# *Tangerine*: A new family of *Starships* from lichen-forming fungi

**DOI:** 10.1073/pnas.2534402123

**Published:** 2026-08-11

**Authors:** Gulnara Tagirdzhanova, Noah E. Brown, Angus H. Bucknell, Ellen S. Cameron, Robert D. Finn, Mark Blaxter, Megan C. McDonald, Emile Gluck-Thaler, Nicholas J. Talbot

**Affiliations:** ^a^ The Sainsbury Laboratory, University of East Anglia, Norwich NR4 7UH, United Kingdom; ^b^ Department of Ecology, Environment and Plant Sciences, Science for Life Laboratory, Stockholm University, Stockholm 106 91, Sweden; ^c^ Department of Plant Pathology, University of Wisconsin-Madison, Madison, WI 53706; ^d^ European Molecular Biology Laboratory, European Bioinformatics Institute, Cambridge CB10 1SD, United Kingdom; ^e^ Tree of Life, Wellcome Sanger Institute, Wellcome Genome Campus, Cambridge CB10 1SA, United Kingdom; ^f^ Institute of Microbiology and Infection, School of Biosciences, University of Birmingham, Edgbaston B15 2TT, United Kingdom; ^g^ Plant Disease Dynamics, Institute of Integrative Biology, ETH Zürich, Zurich 8092, Switzerland

**Keywords:** lichen, symbiosis, genomics, Starship, transposon

## Abstract

Lichens are symbiotic associations between filamentous fungi and photosynthetic micro-organisms, such as green algae and/or cyanobacteria, that result in a single anatomically complex structure that can thrive in environments inhospitable to most organisms, including arctic tundra, high mountains, and deserts. Recent evidence suggests that lichens may be even more complex than previously appreciated, containing multiple microbial constituents, but how genomes of the principal fungal symbiont (which provides the majority of biomass in lichen tissue) have been shaped during evolution is largely unexplored. Recently, giant transposable elements called *Starships* have been found in many genomes of filamentous fungi, but to which extent they occur in lichen-forming fungi is not known. In this report, we describe a *Starship* element from the lichen fungus *Xanthoria parietina*. This element, named *Tangerine*, contains several genes that have signatures of horizontal gene transfer from nonlichen-forming fungi, most likely from black yeasts of the Chaetothyriales, that are often lichen-associated. Repetitive sequences carried by *Tangerine*, and found in other sites in *Xanthoria* genomes, are affected by repeat-induced point mutation, a mechanism of genome defense against transposable elements, consistent with fungal sexual reproduction which always precedes new lichen formation by *X. parietina*. *Tangerine*’s “captain” belongs to a newly defined family of tyrosine recombinases specific to lichen-forming Lecanoromycetes. Several other captain clades have signatures of horizontal gene transfer between distantly related lichen-forming fungi and nonmycobiont lichen-associated fungi. We speculate that *Starships* may play a significant, yet hitherto unrecognized role, in lichen genome evolution and provide a roadmap for further investigation.

## Significance

Lichens are ubiquitous in most terrestrial ecosystems and yet remain poorly understood. In lichens, fungi form a mutualistic symbiosis with photosynthetic micro-organisms that results in a macroscopic plant-like organism. The recent discovery of giant transposons named Starships has challenged our understanding of fungal evolution, but whether Starships have played a role in the evolution of lichen-forming fungi is unknown. Here, we report a new family of Starships exclusive to Lecanoromycetes, most of which are lichen-forming fungi, and several putative instances of Starship-mediated horizontal gene transfer among fungal symbionts present within lichens. Our study suggests that Starship activity may be a significant factor in lichen biology and adds to the body of evidence that Starships are shared among ecologically similar fungi.

The lichen symbiosis is one of the most common, yet poorly understood fungal lifestyles. Lichens are an association between a hyphal fungus (the mycobiont) and a photosynthetic microorganism (the photobiont), in which the majority of the biomass comes from the fungal symbiont (1) but the overall shape and organization of the lichen thallus is much more complex than either partner alone. Recent findings also suggest that lichen thalli can in addition contain many more taxa than the photobiont and the mycobiont, including additional fungi (sometimes referred to as endolichenic or lichenicolous fungi) and bacteria (2–4). Lichen-forming fungi account for approximately 17% of all described fungal species and 27% of ascomycetes (5). The majority of mycobionts are filamentous fungi belonging to the Pezizomycotina and chiefly to the class Lecanoromycetes, which consists of mostly lichen-forming fungi and is a sister clade to Eurotiomycetes (5, 6). Despite being ubiquitous in terrestrial ecosystems, lichens remain very poorly understood and much is unknown about the biology and evolution of these symbiotic associations. Lichen-forming fungi are challenging to isolate in axenic culture and notoriously slow-growing (7), which complicates lichen research—including the production of genomic data. The class Lecanoromycetes, for instance, has three times as many described species as the Eurotiomycetes, but the International Nucleotide Sequence Database Collaboration currently contains six times fewer Lecanoromycetes genomes, the vast majority of which are extracted from short-read metagenomic data and are therefore highly fragmented. Understanding genome evolution in lichen-forming fungi is therefore severely limited by available high quality genome data.

Transposable elements (TEs) are genetic units that may change their own position within their host’s genome (8). Found across all domains of life, TEs are thought to have a profound impact on their host’s biology by both mediating host evolution and inducing genomic instability (9–11). Recently, a novel group of TEs, known as the *Starships*, have been reported and implicated as vectors of fungal–fungal horizontal gene transfer (HGT) (12). Unlike other types of TEs, *Starships* are vast in size and carry variable sets of genes as cargo, many of which encode adaptive phenotypes (13, 14). Additionally, *Starships* carry a more conserved set of four “auxiliary” genes which are thought to be beneficial to the maintenance of the TE. The result of carrying both sets of genes is that *Starships* are significantly larger than other transposons, often surpassing 100 kb in size and containing between ten to several hundred genes (15). Very recent evidence has experimentally demonstrated *Starships’* transposition and their capacity to horizontally transfer between distantly related fungal species (16). While the mechanism of *Starship* transposition is unknown, it is driven by a tyrosine recombinase (tyrR) known as the “captain.” This tyrR is always located as the first gene at the 5′-end of the *Starship* element. *Starships* occur exclusively in Pezizomycotina fungi, the most species-rich group of fungi that includes over 90,000 species with ecologies ranging from saprotrophs to plant symbionts to animal and plant pathogens (17). *Starships* have been proposed to shape the evolution of Pezizomycotina fungi by constituting a large and mobile compartment of the genome and might in part be responsible for rapid and repeated evolution of ecological strategies observed in filamentous fungi (18).

Detection of *Starships* is challenging due to their large size and low copy number (15). Existing tools for *Starship* annotation, starfish (15), and stargraph (19), rely on aligning contiguous genome assemblies from the same species and identifying gaps in the alignment that contain tyrR-encoding genes. Because only a handful of high-quality genomes are available for lichen-forming fungi, few surveys of *Starships* in this group have been completed. Yet, given that Pezizomycotina contains several, independently evolved lichen lineages, it is reasonable to predict the presence of *Starships* in lichen-forming fungi. Indeed, a large-scale screen of *Starships* across ascomycete fungi yielded several predicted *Starships* in the genomes of Lecanoromycete fungi (si appendix, table S8 in ref. 15). Here, we analyze four genomes of lichen-forming fungi from the genus *Xanthoria* (Teloschistales, Lecanoromycetes) derived from long-read sequencing data, two of which are newly generated. We describe *Tangerine*, a putative *Starship* element present in *X. parietina*, and report signatures of its cargo horizontal gene transfer (HGT) with a lineage enriched in nonmycobiont fungi that are known to inhabit lichen thalli (Chaetothryiales, Eurotiomycetes). Following this, we report that the captain of *Tangerine* is nested within a new phylogenetic family [*sensu* Gluck-Thaler and Vogan (15)] of *Starship* captains that are endemic to Lecanoromycetes. Additionally, we present a wider phylogenetic analysis of captains within lichen-forming and nonmycobiont lichen-associated fungi and report signatures of putative horizontal *Starship* transfers based on incongruencies in captain phylogenies among distantly related lichen fungal symbionts.

## Results

### Comparative Analysis of Long-Read *Xanthoria* Genomes.

**Fig. 1.**
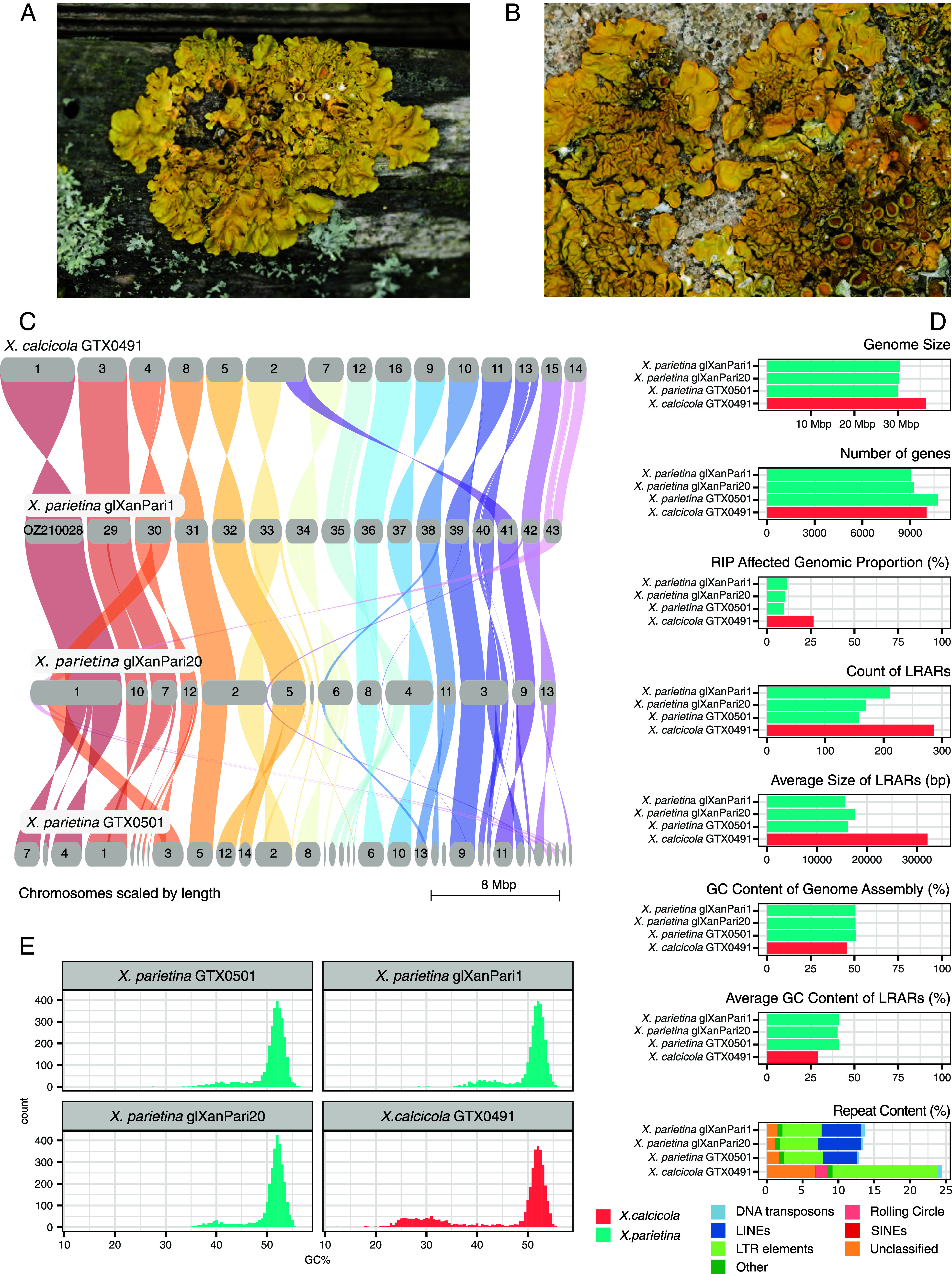
The long-read genomes of *Xanthoria parietina* and *X. calcicola*. (*A*) Lichen thallus of *X. parietina* on wood. (*B*) Thallus of *X. calcicola* on concrete. (*C*) Syntenic map of orthologous regions among three nuclear genomes of *X. parietina* and a genome of *X. calcicola*. Synteny was calculated using GENESPACE based on orthogroup analysis. (*D*) Genome statistics for the four *Xanthoria* genomes. Statistics for *X. parietina* genomes are shown as blue bars; for *X. calcicola* as red bars. RIP stands for repeat-induced point mutation; LRAR stands for large RIP-affected region; LINE stands for long interspersed nuclear element; LTR stands for long terminal repeat; SINE stands for short interspersed nuclear element. (*E*) Distribution of GC content values across the four *Xanthoria* genomes using nonoverlapping windows of 10,000 base pairs.

We compared long-read genomes of three isolates of *X. parietina* and its close relative, *X. calcicola* (Fig. 1 *A* and *B* and *SI Appendix*, Fig. S1). The four genomes showed a high level of synteny, with a similar size and number of predicted genes, but differed in repeat content and GC content (Fig. 1 *C*–*E* and *SI Appendix*, Table S1). For example, in *X. calcicola* repeats accounted for 23% of the nuclear genome, compared to 13% in *X. parietina*. In all examined genomes, long terminal repeat (LTRs) retrotransposons accounted for a large portion of repeat content (*SI Appendix*, Fig. S2 and Table S2). By contrast, long interspersed nuclear elements (LINEs) are absent in the *X. calcicola* genome, yet account for nearly a half of the repeat content in *X. parietina* (Fig. 1*D*). In all genomes, repeat elements were not randomly dispersed but instead organized into specific regions of the genome (*SI Appendix*, Figs. S3 and S4). In addition to high repeat density, these regions also had low GC content and showed signs of repeat-induced point mutation (RIP), and were therefore classified as large RIP-Affected Regions (LRARs, defined as genomic regions >4 kb affected by RIP) (20). RIP is a fungal-specific mutation-based mechanism of genome defense against TEs which acts during the sexual cycle (21). RIP appears to have had a more prominent effect on the frequently asexual *X. calcicola* compared to the obligately sexual *X. parietina* (Fig. 1*D*). The LRARs of the former were both larger and more numerous and had a lower GC content compared to the latter. LRARs appear responsible for the overall lower GC content of *X. calcicola* genome, as the non-LRAR portion of the genomes showed the same median GC content in all four *Xanthoria* isolates (Fig. 1*E*).

### *Tangerine*, A Putative *Starship* Element in *X. parietina*.

Using the Starfish pipeline (15), we screened the four *Xanthoria* genomes for the presence of putative *Starship* elements. We identified three elements, each of which contained a tyrR gene and could be aligned to a corresponding empty site in one of the other genomes (*SI Appendix*, Table S3). One of the identified elements was likely a false positive, because it lacked many key characteristics: It was only 17.9 kb long, mapped to an empty site of nearly 2 kb in length, and lacked any genes except for the tyrR. The two remaining putative *Starships*, which we termed *Tangerine,* were found in two *X. parietina* genomes (glXanPari1 and glXanPari20). They belong to the same navis (orthologous gene group of the tyrR captain; from latin for “ship,” plural “naves”) and haplotype (based on k-mer based similarity across the entire element sequence).

**Fig. 2.**
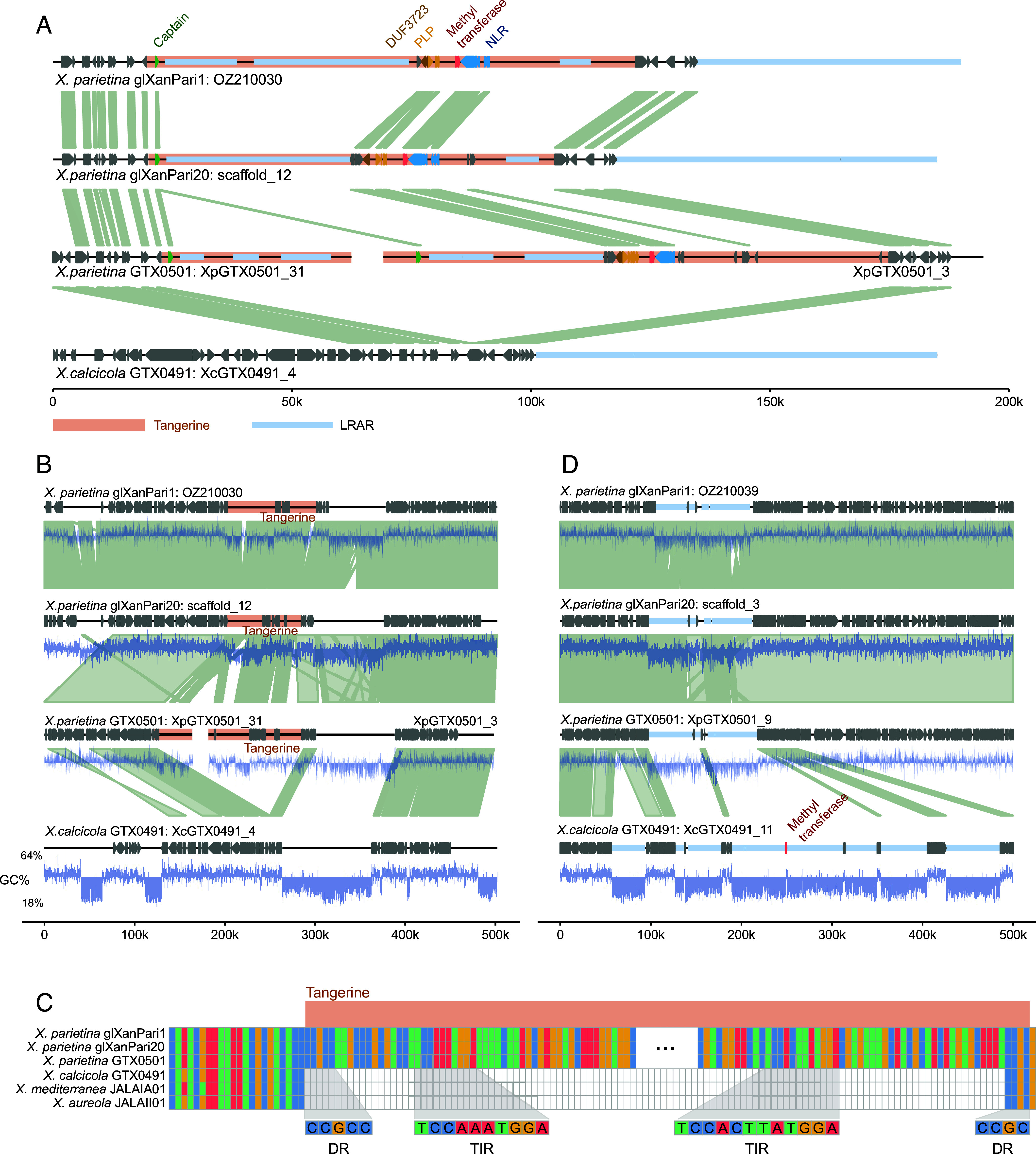
*Tangerine*, a *Starship* element in the genomes of *Xanthoria* fungi. (*A*) *Tangerine* (highlighted in orange) in three *X. parietina* genomes aligned to the empty site in *X. calcicola*. In the genome assembly of *X. parietina* GTX0501, *Tangerine* is split between two contigs. Genes are shown as arrows; the captain, accessory, and cargo genes are highlighted in different colors. The links between genomic fragments show genes belonging to the same orthogroup. Large Repeat induced point mutation Affected Regions (LRARs) are shown as grayblue bars. PLP stands for Patatin-like phospholipase, NLR stands for NOD-like receptor. (*B*) Alignment of *Tangerine* and 200 kb flanking regions. Links between chromosome fragments indicate nucmer nucleotide alignments with >90% sequence identity and >3,000 bp in length. The lower track shows the GC-content. (*C*) Alignment of *Tangerine* and its flanking regions to the empty site in *X. calcicola* and the short-read genomes of *X. aureola* and *X. mediterranea*. Putative direct repeats (DR) and tandem inverted repeats (TIR) are shown as predicted by the automated Starfish annotation. (*D*) Putative degraded copy of *Tangerine* in the genome of *X. calcicola* aligned to the corresponding regions of the three *X. parietina* genomes. Methyltransferase gene is highlighted in red. Blue highlights correspond to LRARs.

*Tangerine* architecture is consistent with that of a *Starship* transposon (Fig. 2). Its size (102 kb in *X. parietina* glXanPari1 and 85 kb in *X. parietina* glXanPari20) is close to the median *Starship* length (15). The captain is located 1,390 bp downstream of the 5′ end of the element; it is the first gene in the 5′ direction (Fig. 2*A*). In addition to the tyrR-encoding captain, *Tangerine* contains typical auxiliary genes encoding a protein with a DUF3723 domain, a patatin-like phosphatase (PLP), and a NOD-like receptor (NLR) (15). The boundaries of *Tangerine* were cleanly defined by alignment to the empty site in the genome of *X. calcicola* (Fig. 2 *A* and *B*). The ends of *Tangerine* contain both 4 to 5 bp direct repeats and 10 to 12 bp terminal inverted repeats (Fig. 2*C*), which notably were separated by 14 bp in the 5′ end of the element and 28 bp in the 3′ end of the element, more than in other reported *Starships* (13).

*Tangerine* was present in all examined *X. parietina* genomes at the same position. At first, *Tangerine* appeared to be missing in *X. parietina* GTX0501, however upon closer examination, we found the element present in the same region but split between two contigs (Fig. 2 *A* and *B*), consistent with this genome being more fragmented than others (Fig. 1*C*). The splitting resulted in two copies of the captain being identified in this assembly, both of them were assigned to the same orthologous group as the captains from *X. parietina* glXanPari1 and glXanPari20 (*SI Appendix*, Table S4). While short-read genomic assemblies are generally too fragmented to allow for *Starship* profiling (15), we confirmed that the 5′ end of *Tangerine* is located in the same region in all eight short-read genomes of *X. parietina* from our earlier study (2) (*SI Appendix*, Fig. S5). Since we only detected *Tangerine* at a single genomic locus, we do not have an indication of whether it is still transpositionally active, and hence, consider it a putative *Starship*.

*Tangerine* encodes additional cargo genes beyond the captain and auxiliary genes (*SI Appendix*, Table S5). These include seven predicted genes that lack functional annotation, a gene model encoding a protein with an Ankyrin domain, and, notably, a putative methyltransferase annotated as IPR013216 (Methyltransferase type 11) from the S-adenosyl-L-methionine-dependent methyltransferase superfamily. Across the three *Tangerine* copies in *X. parietina,* predicted proteins from each orthogroup were >95% identical. This was the case for both cargo and auxiliary genes (*SI Appendix*, Fig. S6). The captain sequences were identical in all three genomes.

We used previously produced transcriptomic data from the mycobiont of *X. parietina* and intact *X. parietina* thalli (2) to investigate whether *Tangerine*-associated genes are expressed. Most of the genes, including the captain, were expressed in at least some of the samples, with the NLR and methyltransferase showing highest levels of expression in both lichen thalli and the fungus cultured separately in the lab (*SI Appendix*, Figs. S7 and S8). Out of seven individual lichens from which metatranscriptomic data was produced, two had near zero-levels of expression for all *Tangerine*-associated genes, which might indicate that *Tangerine* has been lost in some *X. parietina* individuals.

More than a half of *Tangerine* (63% in *X. parietina* glXanPari1 and 54% in *X. parietina* glXanPari20) consists of smaller TEs, which is consistent with previous reports of TEs nesting within *Starships* (14, 22) *Tangerine*-associated TEs are affected by RIP and are mostly assigned to LTR/Ty3 and LINE/Tad1 from the same families that dominate repeat content in the rest of the genome (Fig. 2*A* and *SI Appendix*, Table S6). In addition, a 47 to 61 kb LRAR is located 13 kb downstream of the 3′ end of *Tangerine* in the *X. parietina* genomes and can be also detected in *X. calcicola*, where it is present 13 kb downstream of the empty site. This LRAR corresponds to the 100 kb largely unaligned region downstream of *Tangerine* (Fig. 2*B*), in which only small fragments, mostly corresponding to LTR/Ty3 TEs, could be aligned between *X. parietina* and *X. calcicola* (*SI Appendix*, Fig. S9). Compared to *X. parietina*, the LRAR in *X. calcicola* has a lower GC content and a larger portion of repeats that could not be assigned to known superfamilies (32% compared to 0.4% in *X. parietina*; Fig. 2*B* and *SI Appendix*, Fig. S9), perhaps owing to sequence modification caused by intensive RIP.

To test for the possibility of misassembly, we examined read alignments to the boundaries of *Tangerine* in *X. parietina* glXanPari1 and to the empty site in *X. calcicola* (*SI Appendix*, Fig. S10). No likely misassembly was detected. At the same time, we detected regions with an uneven depth of coverage within *Tangerine* (*SI Appendix*, Fig. S10). Using the read alignments, we annotated structural variants (SVs) within the element and in 200 kb flanking regions. This analysis yielded 16 SVs, of which 10 were present within *Tangerine* (*SI Appendix*, Table S7). The majority of the SV were indels present in GC-poor regions lacking genes, downstream of the captain or upstream of the 3′ end of *Tangerine*. Two notable exceptions are a break point 907 bp downstream of the captain and an insertion within the gene model XANPAOZ2100_002164-T1 (DUF3723), which appears to represent an intron. An identical intron is present in the corresponding gene in *X. parietina* glXanPari20 but is absent from *X. parietina* GTX0501.

### *Tangerine* May Be Present in Other *Xanthoria* species at Different Locations.

To test whether the *Tangerine* element can be located anywhere else in the genome of *X. calcicola,* we searched all *Tangerine*-associated genes in the predicted proteome, genome assembly, and raw reads generated from the *X. calcicola* GTX0491 sample from this study. This search yielded one hit: The amino acid sequence of predicted protein XANCAGTX0491_008091-T1 was 95% identical to the methyltransferase from *Tangerine* cargo (*SI Appendix*, Fig. S6). This similarity was in line with the similarity of most ortholog pairs in *X. calcicola* and *X. parietina* (*SI Appendix*, Fig. S11). The gene was located in a different contig compared to the empty site described above and was flanked by two LRARs, 61.5 and 45 kb in length (Fig. 2*D*). Compared with the corresponding region in the three *X. parietina* genomes, *X. calcicola* sequence is inflated by numerous repeat-rich LRARs, which resulted in patchy alignment between the genomes of the two species. It is possible that a copy of *Tangerine* was present in *X. calcicola* at this site and that it has been degraded by RIP [as has been shown for other *Starships* (13)] until the methyltransferase gene was left as the only remaining “fossil.” Alternatively, the methyltransferase may have been captured and transposed by a different mobile element.

In short-read genome assemblies of *X. mediterranea* and *X. aureola* (23), we found the same empty site as in *X. calcicola* (Fig. 2*C* and *SI Appendix*, Fig. S12*A*). Similarly to *X. calcicola*, in the assembly of *X. aureola* we identified only the *Tangerine*-associated methyltransferase, perhaps reflecting a similar lost element, or possibly the original fungal locus picked up as cargo in the *Tangerine*. In *X. mediterranea* we identified the captain, all the auxiliary genes, and the methyltransferase, but these were split between three contigs (*SI Appendix*, Fig. S12*B*), suggesting that a more intact copy of *Tangerine* may be present in this species at a different genomic locus than that of *X. parietina*.

### The *Tangerine* Captain Defines a Lecanoromycetes-Specific Family of *Starships*.

Initial HMM-based profiling by Starfish assigned the *Tangerine* captain to clade 1 of the *Starship* tyrR tree but did not confidently assign it to any of the previously described families. This contrasted with other putative tyrRs detected in *Xanthoria* genomes, many of which were assigned to families such as Phoenix and Galactica (*SI Appendix*, Table S4). To investigate the *Tangerine* captains further, we aligned their sequences to the sequences of 1,222 representative *Starship* tyrRs from Gluck-Thaler & Vogan (15), and reconstructed a phylogeny. In the resulting tree, the *Tangerine* captain was recovered together with three tyrRs from fungi in the same class Lecanoromycetes (*SI Appendix*, Fig. S13).

**Fig. 3.**
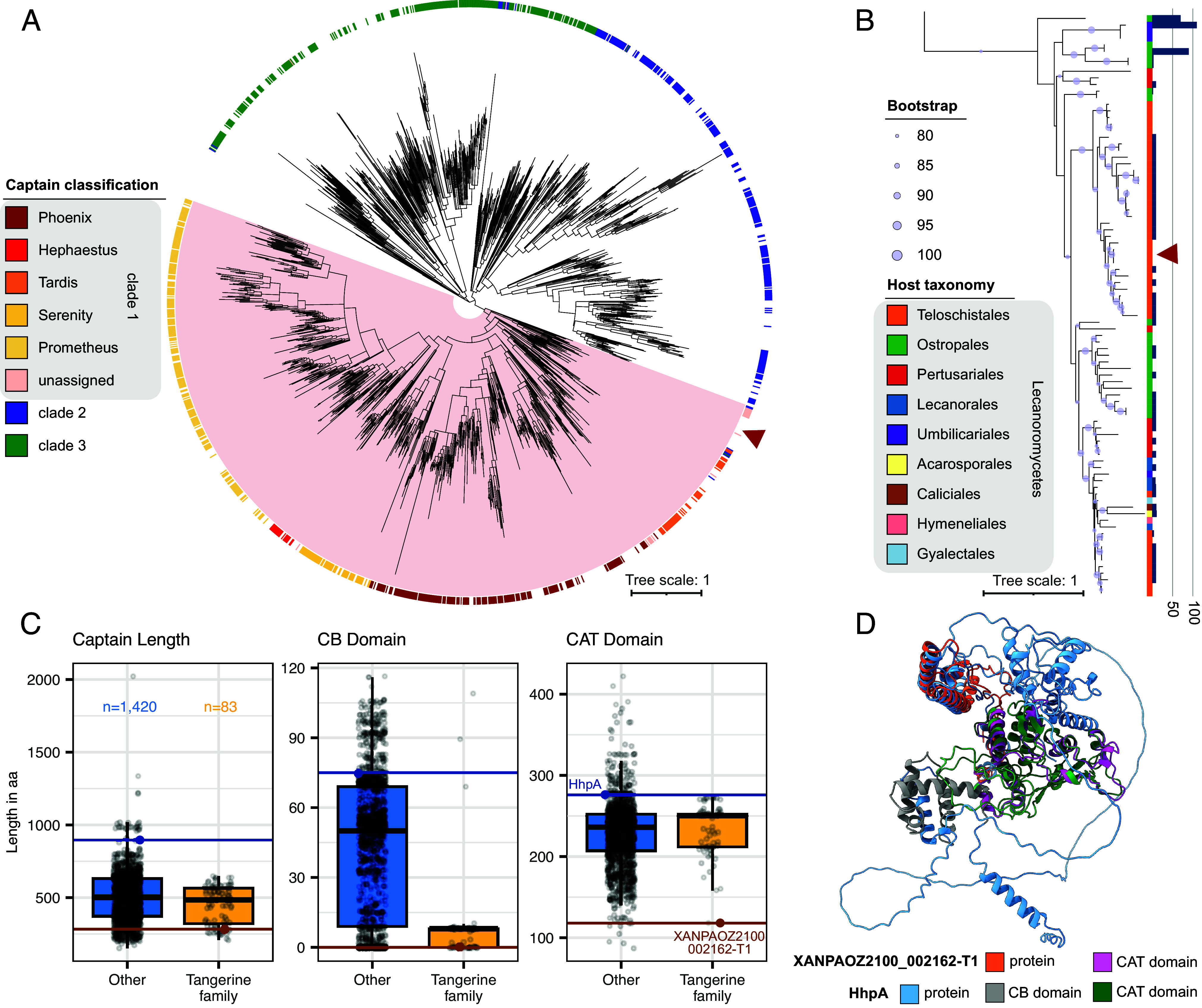
*Tangerine* in the context of the *Starship* captain phylogeny. (*A*) Sequence-based *Starship* captain tree with the Tangerine-family indicated by the red arrow. Predicted captains from lichen-associated fungi were clustered into representative sequences and aligned with 1,222 representative *Starship* captains; the alignment was used to generate a maximum-likelihood phylogeny with 100 bootstrap replicates. Clades with <70 bootstrap support are collapsed. The full tree in Newick format with bootstrap values, together with the sequence alignment can be found in the supplemental data files (FigShare). The outer color track shows captain family assignments; clade 1 is highlighted as a pink block. (*B*) Magnified clade of the Tangerine-family showing the full set of captains (both representative and not), annotated based on the taxonomy of their host. The *Tangerine* captain from *X. parietina* is indicated with the arrow. Bootstrap support shown next to the nodes. The bars to the *Right* of the tree show the length of the CB (core-binding) domains for each captain. The full tree with taxa labeled is in *SI Appendix*, Fig. S14. (*C*) Distribution of full captain, CB domain, and catalytic (CAT) domain lengths (aa) between the Tangerine-family (n = 83) and other *Starship* captains (n = 1,420). The Tangerine-family set includes the complete set of unclustered captains from the family. The other families are represented by captains annotated de novo in genomes of lichen-associated fungi and a subset of captain representatives from Gluck-Thaler and Vogan (15). The *Tangerine* captain from *X. parietina* (XANPAOZ2100_002162-T1) is highlighted in orange, and HhpA captain from *Hephaestus* in blue. (*D*) Structurally aligned, AlphaFold3-generated structural predictions of the *Tangerine* captain from *X. parietina* (XANPAOZ2100_002162-T1) and HhpA. Different colors indicate conserved domains: CB and CAT in HhpA and CAT in XANPAOZ2100_002162-T1.

To determine whether the *Tangerine* clade met the standards of being considered a new *Starship* family, we supplemented the dataset with 1,508 tyrRs (dereplicated into 494 representative sequences using amino acid based sequence clustering) that we identified in a larger set of 554 genomes from lichen-forming and lichen-associated fungi (*SI Appendix*, Table S8). In the reconstructed phylogeny, the *Tangerine* clade sat at the basal part of clade 1 and contained exclusively tyrRs from Lecanoromycetes, most of them lichen-forming (Fig. 3 *A* and *B* and *SI Appendix*, Fig. S14 and Table S9). The clade met the threshold to be recognized as a new *Starship* family (Tangerine-family), as described by Gluck-Thaler & Vogan (15) (>20 representative captains forming a monophyletic clade with >80% SH-ALRT and >95% ultrafast bootstrap support). We performed an additional search against an unfiltered set of captains from 1,649 diverse fungal genomes from Gluck-Thaler & Vogan (15) but failed to recover any Tangerine-family sequences from non-Lecanoromycete fungi.

While the median length of captains from the Tangerine-family did not differ from the other *Starship* families, they had a markedly shorter core-binding (CB) domain, one of the characteristic DNA binding domains of tyrosine recombinases (Fig. 3*C*). Over a third (34.9%, n = 29) lacked a CB domain entirely, and an additional 53% (n = 44) had a putative CB domain only eight amino acids long and with no similarity to the CB domain of the HhpA captain from the well-studied *Hephaestus Starship* (*SI Appendix*, Fig. S15), suggesting that the CB domain was lost in these captains as well. To confirm that the absence of a CB domain was not an artifact of annotation, we searched HhpA against the nucleotide sequence of the *Tangerine* element, which yielded no hit for the CB domain. In contrast, the larger Tangerine*-*family captains tended to retain the catalytic (CAT) domain (Fig. 3*C*), and all conserved active sites within it (*SI Appendix*, Table S10). Notably, however, the captain of the *Tangerine* element from *X. parietina* had a short sequence of 283 amino acids and lacked sites 1 and 2 out of the six conserved canonical active sites.

To validate the results of the sequence-based analyses, we predicted and analyzed the *Tangerine* captain structure. A predicted structure of the captain monomer was generated via AlphaFold 3 (*SI Appendix*, Fig. S16) and searched against the Protein Data Bank (PDB), which yielded no significant (*E*-value < 0.001) results (*SI Appendix*, Table S11). However, when this *Tangerine* captain was structurally aligned to and compared against the predicted structure of HhpA (Fig. 3*D* and *SI Appendix*, Fig. S17*A*), strong structural similarity was observed between the CAT domains with a rmsd value of 1.89 Å across the 134 atomic pairs. Three of the six characterized active sites within HhpA had corresponding residues within the *Tangerine* captain (*SI Appendix*, Fig. S17*B*). No region of the *Tangerine* captain could be identified to be corresponding to the CB domain found in HhpA (Fig. 3*D* and *SI Appendix*, Fig. S17*A*). When comparing HhpA to an additional Tangerine-family captain from *Caloplaca sp.* (*SI Appendix*, Fig. S17*C*) containing eight residues of the CB domain, no similarity was observed (*SI Appendix*, Fig. S17*D*), providing another line of evidence that CB domain may be lost in most of the Tangerine-family captains.

To investigate the structural relationships between the Tangerine-family and other *Starship* captains, we generated structural phylogenies using Foldtree based on various distance matrices derived from protein–protein alignments (24). The phylogeny based on the Foldtree metric, which combines both local structure and sequence information, reveals the Tangerine-family captains form a distinct clade (*SI Appendix*, Fig. S18). However, both structure-only phylogenies, based either on local distance difference tests (LDDT) or template modeling score (TM), had large inconsistencies with the sequence-based phylogeny. Only the LDDT-based phylogeny formed a Tangerine-family clade, which did not include some members of the Tangerine-family, including the captain from the *Tangerine* element in *X. parietina* (*SI Appendix*, Fig. S18).

### *Tangerine*-Associated Genes Show Signatures of Horizontal Gene Transfer.

**Fig. 4.**
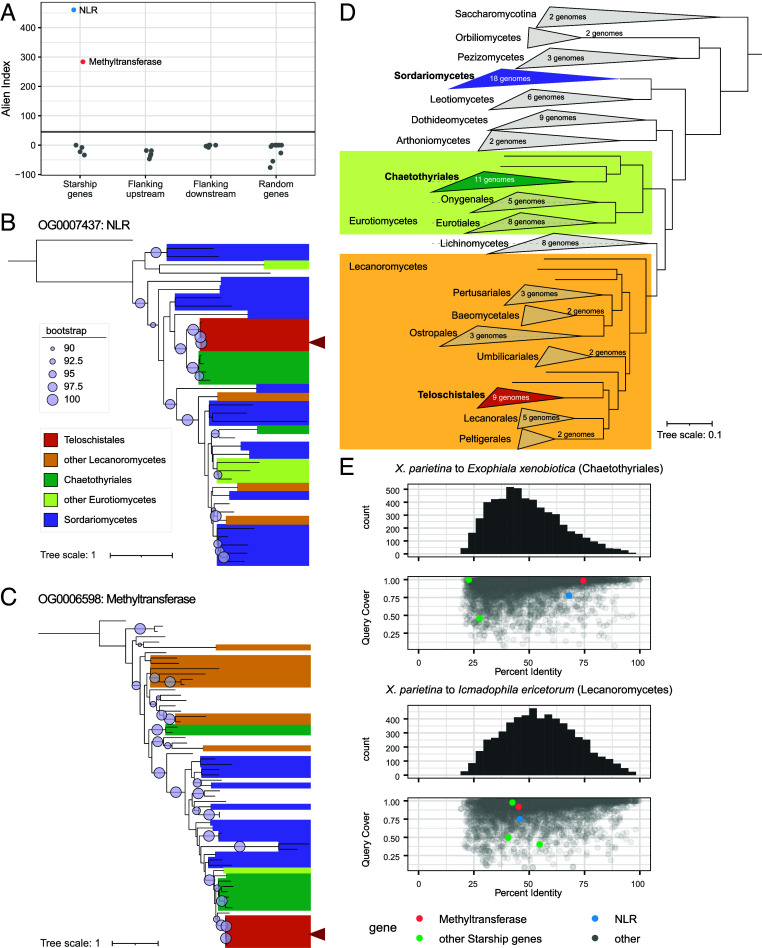
Putative horizontal gene transfer (HGT) of *Tangerine*-associated genes. (*A*) Alien Index (AI) of *Tangerine*-associated genes, gene models in the immediate vicinity of *Tangerine*, and 10 randomly selected gene models from *X. parietina* glXanPari1. AI is defined as ln(best E-value of nonlecanoromycete hit + 1e^−200^) − ln(best E-value of non-Teloschistales lecanoromycete hit + 1e^−200^). The horizontal line corresponds to the AI of 45, which is considered the threshold for likely HGT (28). (*B* and *C*) Gene tree for the orthogroup containing *Tangerine*-associated NOD-like receptor (NLR, *B*) and methyltransferase (*C*). To obtain the trees, we performed orthogroup analysis on 107 ascomycete genomes (*SI Appendix*, Table S14). For the selected orthogroups, we computed maximum-likelihood phylogeny. The tree is colored based on the taxonomic origin of sequences; bootstrap values are shown for the clades with >90 support. The sequences from *X. parietina* glXanPari1 are indicated with arrows. Full trees with taxa labeled are in *SI Appendix*, Figs. S25 and S26. (*D*) Species tree generated by Orthofinder for the 107 ascomycete genomes. The tree is rooted using two Saccharomycotina genomes. (*E*) Cross-mapping of the predicted proteome of *X. parietina* glXanPari1 to *Exophiala xenobiotica* and *Icmadophila ericetorum*. For each *X. parietina* glXanPari1 predicted protein, we identified the best match in the other predicted proteome. The *Top* panel shows the distribution of percentage identity values across all matches; the *Bottom* panel shows how the matches were positioned in relation to percentage identity and query cover. NLR, methyltransferase, and other *Tangerine*-associated genes are highlighted in color.

*Starships* have been implicated as vectors of HGT in fungi (16, 25–27). Within the *Tangerine* element in *X. parietina*, one auxiliary gene, the NLR, and one cargo gene, the methyltransferase, appear to have been horizontally transferred. The two genes neighbor each other and are separated by only 350 bp. When searched against the NCBI nr database, these genes returned numerous high-quality hits (>95% identity over >95% sequence) to immediate relatives of *Xanthoria* from the order Teloschistales and to distantly related Chaetothyriales fungi (Eurotiomycetes). Crucially, no hits to Lecanoromycete fungi other than from Teloschistales were obtained (*SI Appendix*, Fig. S19). To rule out the possibility that these genes were missing from the NCBI nr database due to variable genome annotation, we searched the NCBI nt database but failed to obtain a non-Teloschistales Lecanoromycete hit of a better quality than hits to Chaetothyriales (*SI Appendix*, Table S12). The Alien Index (AI) (28) for these two genes was 461 and 284 respectively, which is well above 45, the threshold for likely HGT (Fig. 4*A* and *SI Appendix*, Table S13). This pattern was unique to the NLR and methyltransferase and was not detected in other *Tangerine*-associated genes, flanking genes in the vicinity of *Tangerine*, or 10 randomly selected genes from the genomic background as controls (*SI Appendix*, Figs. S20 and S21).

To further examine the evolutionary histories of the putative HGT candidates, we ran an orthogroup analysis on 107 Ascomycete genomes (*SI Appendix*, Table S14). Gene trees for the NLR and methyltransferase-containing orthogroups were discordant compared to the species tree (Fig. 4 *B*–*D* and *SI Appendix*, Figs. S22 and S23). For both genes, a well-supported clade of genes from Teloschistales is closely related to Chaetothyriales genes. In the case of the NLR, the Teloschistales clade is sister to the Chaetothyriales clade. In the case of the methyltransferase, Teloschistales clade is nested within Chaetothyriales. Notably, the same methyltransferase clade contains genes from two additional distantly related lichen-forming fungi: *Chaenotheca gracillima* (Coniocybales, Lichinomycetes; protein similarity 76% over 99% coverage) and *Endocarpon pussilum* (Verrucariales, Eurotiomycetes; protein similarity 73% over 98% coverage). In both gene trees, the clade containing Teloschistales and Chaetothyriales genes is nested within a larger clade consisting primarily of genes from Sordariomycete fungi. Again, the discordance between gene trees and the species tree was observed only for the NLR and methyltransferase, and not for other *Tangerine-*associated genes or genes in its vicinity (*SI Appendix*, Figs. S24 and S25). The two orthogroups including the NLR and methyltransferase were present in 21 genomes included in the analysis. Excluding *Xanthoria*, in eight genomes the two genes are located in the vicinity of each other, and only in three genomes—two from Chaetothyriales and one from Sordariales—did the “tail-to-tail” gene configuration match that observed in *Tangerine* (*SI Appendix*, Fig. S26).

We cross-mapped the proteomes of *X. parietina* with *Icmadophila ericetorum*, a fungus from the same class of Lecanoromycetes, and *Exophiala xenobiotica*, a Chaetothyriales fungus whose genome encoded homologs of the key *Tangerine*-associated genes (Fig. 4*E*). Both the NLR and methyltransferase had higher percentage identity between *X. parietina* and *E. xenobiotica* compared to the more closely related pairing of *X. parietina* and *I. ericetorum*.

### Other *Starships* May Be Present in *Xanthoria* Genomes.

In addition to the captain of the *Tangerine* elements, the four *Xanthoria* genomes encoded 16 other putative tyrR genes which grouped into nine orthologous gene groups i.e., naves (*SI Appendix*, Table S4). While none of them was associated with a predicted element with clearly defined boundaries, several were positioned within 100 kb of genes encoding PLP or NLR proteins, or other *Starship* auxiliary genes. As more high quality *Xanthoria* become genomes available, we may be able to validate whether any of these non-*Tangerine* tyrRs are associated with bonafide *Starship* elements.

### Three Captain tyrR Clades Show Signatures of Horizontal *Starship* Transfer Among Distantly Related Lichen Fungal Symbionts.

**Fig. 5.**
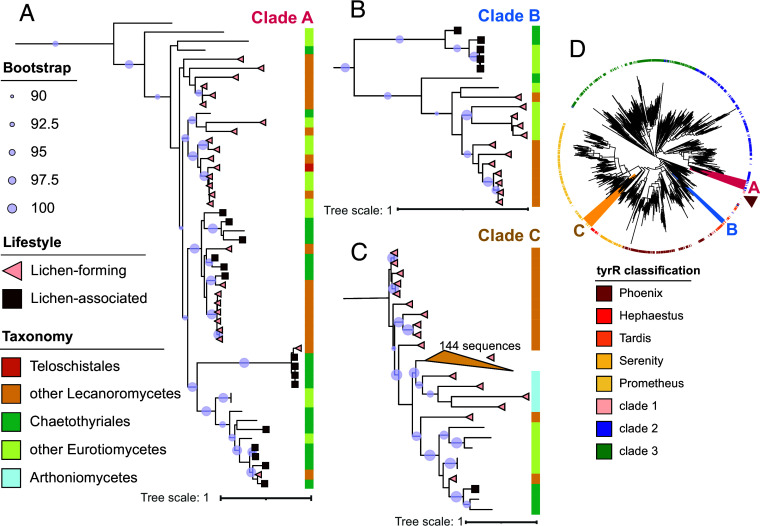
Clades of *Starship* tyrR captains exhibiting signatures of horizontal gene transfer (HGT) between distantly related lichen symbionts. (*A*–*C*) Phylogenetic clades of interest. Sequences from all tyrR captains (both representative and not) within clades from the larger captain phylogeny that showed signatures of HGT in lichen fungi were realigned, and the alignment was used to recreate more refined clade-specific maximum-likelihood phylogenies. Bootstrap values are shown for branches with >90 support. The symbols at the ends of branches indicate lifestyle: tyrRs from lichen-forming fungi (also known as mycobionts) are shown in pink triangles, tyrRs from nonmycobiont lichen-associated (also known as lichenicolous or endolichenic) fungi are shown as brown squares. Sequences from nonlichen fungi lack annotation. The color strip is colored according to the taxonomic group. Full annotated trees are shown in *SI Appendix*, Figs. S27–S29. Information on the sequences from Clades A–C is shown in *SI Appendix*, Table S15. (*D*) Clades A–C in the context of the larger tyrR tree. The color strip shows the family and clade assignments. The Tangerine-clade is indicated by the red arrow.

We used the newly constructed phylogenetic tree of tyrR captains to identify other instances of possible HGT between distantly related lichen-forming and nonmycobiont lichen-associated fungi, this time based on incongruencies between the captain tree and the species tree. In the captain tree, we located three clades of interest (Clades A, B, and C), in which captains from different classes of lichen-associated fungi clustered together in a way that is discordant with the species tree (Fig. 5 *A*–*C* and *SI Appendix*, Figs. S27–S29 and Table S15). Clade A is nested within the Galactica-family and consists primarily of captains from lichen-forming Lecanoromycetes and the Eurotiomycete lichen *Endocarpon pusillum*, as well as nonmycobiont lichen-associated Chaetothryiales (Fig. 5 *A* and *D*). This clade shows a high degree of discordance with the species tree suggesting multiple potential HGT events. Clade B is positioned within the Tardis-family and suggests HGT events from Eurotiomycetes to Lecanoromycetes lichen-forming fungi (Fig. 5 *B* and *D*). Since Eurotiomycetes and Lecanoromycetes classes are quite closely related (Fig. 4*D*), this result warrants more caution. Yet, since the clade overwhelmingly consists of sequences from lichen-associated fungi with only two sequences from nonlichen Eurotiomycetes present, HGT within the context of a lichen symbiosis remains a plausible explanation. Clade C was recovered within the Prometheus-family and presents evidence for possible HGT between distantly related lichen-forming fungi from the classes Lecanoromycetes and Arthoniomycetes (Fig. 5 *C* and *D*). Given that Arthoniomycetes is a sister clade to Dothideomycetes and is even more distantly related to Lecanoromycetes and Eurotiomycetes (Fig. 4*D*), HGT appears likely. While this evidence further indicates that *Starship*-mediated HGT may occur between fungi united by their lichen-associated lifestyles, further studies based on more contiguous genomes are necessary to validate the described instances of HGT.

## Discussion

Since their recent discovery, *Starships* have been implicated in the evolution of multiple features crucial to the lifestyle of their fungal hosts, including specialized metabolism, virulence, and abiotic stress relief (13, 26, 27, 29, 30). We therefore set out to investigate whether the lichen lifestyle may also be shaped by *Starship* activity. Here, we provide the first detailed report of a putative *Starship* element in the genome of a lichen-forming fungus. We named the element *Tangerine* in reference to the yellow-orange color of *Xanthoria* lichens and after a spacecraft briefly featured in one of Douglas Adams’s novels (31). *Tangerine* possesses all the hallmark characteristics of a *Starship*, including its large size, boundaries defined by direct repeats four base pairs long, and characteristic gene content, including a tyrosine recombinase captain gene, auxiliary genes, and cargo.

We found the *Tangerine* element in the genomes of *Xanthoria parietina*, commonly known as the sunburst lichen. Across the genus *Xanthoria*, we had access to several genomes of sufficient quality to allow *Starship* detection, which is rarely the case for lichen-forming fungi, enabling this analysis to be carried out. Whether the *Tangerine* element in *Xanthoria* is still mobile or whether it has lost the ability to transpose is not yet clear. In all of the *X. parietina* genomes analyzed, including short-read genomes used for additional screening, *Tangerine* was always found in the same genomic region, suggesting it no longer is mobile. In the other three surveyed *Xanthoria* species, however, this region contained an empty site. *Tangerine* may be present elsewhere in the genome of *X. mediterranea*, but its exact location cannot be determined due to a lack of long contiguous regions in the genome assembly. In the long-read genome of *X. calcicola*, only a potential degraded copy of *Tangerine* could be detected, again positioned at a distinct site in the genome. When considered together, this evidence suggests that if *Tangerine* is not currently active, it may have been transposing as recently as the time of *Xanthoria* diversification. Regardless of whether *Tangerine* is still mobile or not, the *Tangerine* captain, auxiliary genes, and cargo are expressed in lichen thalli, which may indicate their encoded products have continued to play some biological role in the fungus.

By annotating captains in a larger set of lichen-associated fungi, we revealed *Tangerine* to be the first representative of a new family of *Starships* endemic to the Lecanoromycetes. The CB domain is truncated in this family, which may indicate altered or loss of DNA binding activity. Yet, loss of the CB domain does not necessarily imply the loss of recombination activity, as several bacterial tyrosine recombinases with no CB domain can nonetheless recognize and bind DNA and carry out recombination (32). Notably, the *Tangerine* captain from *X. parietina—*the only member of the Tangerine-family whose exon boundaries could be validated using RNA data —has a truncated CAT domain in addition to the loss of CB domain, raising further questions about its functionality. However, the overall conservation of the *Tangerine* captain’s predicted structure with the *Hephaestus* HhpA captain, coupled with the fact that it has maintained its position at the 5′ terminus of a putative *Starship*, suggests it still plays some role in the biology of *Starship* transposons. Future research will clarify whether Tangerine-family captains are capable of transposition and/or some other function.

*Starship* cargo genes often perform functions beneficial to their hosts and directly involved in their lifestyle, such as effector genes in plant pathogenic fungi. Since little is known about the machinery of the lichen symbiosis, our ability to make such inferences regarding the cargo of the *Tangerine* element is limited. However, one gene, the methyltransferase (XANPAOZ2100_002166-T1 in *X. parietina* isolate glXanPari1) does stand out. This gene was the only *Tangerine-*associated gene also detected in the genomes of *X. calcicola* and *X. aureola*. In *X. calcicola*, it appears within a potential degraded copy of *Tangerine* as the only gene surrounded by large RIP-affected regions. Such conservation amid extensive RIP is unusual and might suggest that the gene is being maintained by selection because it may be beneficial to these lichen-forming fungal species. This might be analogous to the plant pathogen *Parastagonospora nodorum*, which carries an intact and active virulence factor *ToxA* in a degraded copy of the *Horizon Starship* (26). The gene was assigned to the S-adenosyl-L-methionine (SAM)-dependent methyltransferase superfamily. SAM-dependent methyltransferases are ubiquitous and act on a wide range of substrates (33). Among other functions, SAM-dependent methyltransferases are involved in secondary metabolism, including synthesis of polyketides, a class of metabolites prevalent in lichens (34, 35). It is plausible that this gene could be involved in the biosynthesis of natural products. The role of this gene and its potential *Starship*-mediated transfer therefore warrants further investigation.

Two *Tangerine-*associated genes, including the SAM-dependent methyltransferase, appear to have been horizontally transferred from other fungal taxa. The exact origin of the genes is difficult to pinpoint, but our phylogenetic analysis suggests origins either in the Chaetothyriales (Eurotiomycetes) or in the Sordariomycetes. Both of these groups harbor *Starships* (36), which suggests the possibility of *Starship*-mediated HGT; however, we cannot rule out that *Starships* acquired these genes after they had been horizontally transferred through some unknown mechanism. Chaetothyriales fungi consist primarily of black yeasts with diverse ecologies (37), including notably nonmycobiont lichen-associated fungi, which are present in lichen thalli in small quantities (38, 39). Indeed, one of the Chaetothyriales fungi harboring a highly similar methyltransferase gene, *Knufia peltigerae* (*SI Appendix*, Fig. S23), has been described from a lichen thallus (40). Moreover, in a recent analysis of *X. parietina* metagenomes, we reported several Chaetothyriales lineages present sporadically in *X. parietina* thalli (2). Since lichen mycobionts can coexist with Chaetothyriales in the context of the symbiotic lichen thallus, HGT between the two groups is plausible. Notably, one of the two genes, the methyltransferase, also appears to have been transferred not only to the order Teloschitales, including *Xanthoria* but also to the genomes of additional two lichen-forming fungi from different classes of Ascomycota, which might suggest a link to the lichen lifestyle. This transfer is not likely to be recent, as the methyltransferases from these fungi share only about 70% sequence similarity.

*Tangerine-*mediated HGT is not the only instance of possible HGT between distantly related lichen-associated fungi that we found. By examining the phylogeny of *Starship* captains, we identified three clades within the broader tyrR captain phylogeny with signatures of HGT among lichen-forming Lecanoromycetes, lichen-forming Eurotiomycetes, lichen-forming Arthoniomycetes, and lichen-associated Chaetothryiales (Eurotiomycetes). Host relatedness has been hypothesized to drive horizontal *Starship* transfer (36), but additional ecological factors might be at play. For example, these HGT events may have been driven by spatial proximity: either between two lichens living in the same microhabitat, or between a lichen-forming fungus and a lichen-associated (“endolichenic”) fungus within the same lichen thallus. Our ability to explore these possibilities was limited by the fact that the majority of the fungal genomes available for this analysis were assembled from short-read data—the assembly quality permitted annotation of captains but not the associated *Starship* elements. Future analyses using long-read genomes will stress-test the validity of the potential HGT events and allow us to explore potential links between host ecology and *Starships*. The presence or absence of ecologically relevant cargo genes within these *Starships* may shed light onto the drivers of lichen-mediated HGT.

Our dataset also allowed us to compare genomes of two closely related lichen-forming fungi that differ in the frequency of sexual reproduction. While largely similar, the genomes showed stark differences in repeat content, with the often-asexual *X. calcicola* having both higher repeat content and more pronounced RIP. This difference might indeed be linked to the difference in frequency of sexual cycle in *X. calcicola*. TEs might accumulate in the asexual propagation phase and then be efficiently RIPed during more rare crossing events, whereas frequent meiosis in *X. parietina* may prevent accumulation of repeats and therefore lead to fewer RIPed regions. In lichen-forming fungi, closely related species often differ in presence or absence of sexual reproduction (41). For these fungi, the evolutionary consequences of sex are larger than for nonsymbiotic fungi. In addition to effects on genome evolution and recombination, for lichen fungi the presence or absence of sex also affects their transmission strategy. Sexually reproducing species have to undergo “resynthesis” and reestablish the lichen symbiosis following ascospore germination, while asexual lichen-forming fungi typically cotransmit with their symbionts in larger vegetative propagules containing both fungus and alga/cyanobacterium (1). Future research will clarify the trade-offs associated with different lichen reproductive strategies, both in connection to genome evolution and the evolution of symbiosis.

Lichen biology remains largely unknown and mysterious, in large part due to the numerous methodological challenges lichens pose as study organisms. Our discovery of the *Tangerine* element in *Xanthoria*, the *Tangerine-*family of *Starships* in Lecanoromycetes, and signatures of *Starship* HGT among distantly related lichen symbions, however, raises the possibility that these unusual transposons may play a role in lichen biology. As long-read genome sequencing becomes more accessible, more high-quality genomes of lichen fungi will undoubtedly be generated from lichen metagenome studies allowing further evaluation of our hypothesis that *Starships* contribute to the evolution of the lichen lifestyle. The presence of *Tangerine* in *Xanthoria* genomes and signatures of *Starship*-mediated HGT among lichen symbionts provides the basis and rationale for such analyses.

## Methods

### Genome Assembly and Annotation.

To obtain the *Xanthoria calcicola* genome, we extracted DNA from a lichen sample using a NucleoBond High Molecular Weight DNA Kit and sequenced it with PromethION. We used Dorado v0.2 for basecalling and Flye v2.9-b1780 for assembly. For the *X. parietina* genome glXanPari20, DNA was extracted from a lichen sample following Park et al. (42) and submitted for PacBio HiFi Sequencing. Assembly was generated with metaMDBG v0.3 (43). For both, the metagenomic assembly was binned using MetaBAT (44) to generate the mycobiont genome. See *SI Appendix, Methods S1* for the details on DNA extraction, sequencing, and assembly.

Four genomes were analyzed: *X. parietina* isolate GTX0501 from Tagirdzhanova et al. (2) (PRJEB78723; genome accession GCA_964263705.1), *X. parietina* glXanPari1 from the isolate SAMEA111342678 (PRJEB83421; genome accession GCA_964656405.1), and the two genomes described above. The genomes were annotated using Funannotate v1.8.15 (45) (*SI Appendix, Methods S2*).

### *Starship* Detection.

To detect potential *Starships*, we used Starfish v1.0.0 (15). For the genes inside the *Tangerine* element, we confirmed the transcript annotation by cross-referencing the exon boundaries with mapping of the metatranscriptomic data from *X. parietina* (SRA: ERR4235132 and ERR4235133). This way, we corrected exon boundaries in the captain genes in *X. parietina* glXanPari1 and glXanPari20 (*SI Appendix*, Fig. S30). To test that the CB domain and a portion of CAT domain is genuinely truncated in the *Tangerine* captains, we attempted to find additional exons that would cover the “missing” portion of the *Tangerine* captain by searching HhpA against the nucleotide sequence of the *Tangerine* element, which yielded no additional hit. See *SI Appendix, Methods S3* for details on *Starship* annotation.

### Captain Characterization.

To place the *Tangerine* captain in the families of other *Starships*, we combined its sequence with the 1,222 selected representative sequences of tyrRs from Gluck-Thaler and Vogan (15) and Crypton sequences from fungi (AAO92638.2 and KAE8209120.1), aligned them with MAFFT v7.271 (46) using the E-INS-i method. We removed columns with >70% gaps using trimAl v1.2 (44) and, following Gluck-Thaler and Vogan (15), extracted 512 first positions from the trimmed alignment and reconstructed the phylogeny using IQ-TREE v2.2.2.2 (47) with automated model selection, 1,000 SH-ALRT, iterations, and 1,000 ultrafast rapid bootstraps.

Following the observation of the *Tangerine-*like captains forming a Lecanoromycetes-specific clade, we tested whether this clade forms a new lichen-associated *Starship* family. We annotated captains in publicly available genomes of diverse lichen-associated fungi (*SI Appendix*, Table S17) and clustered them to produce dereplicated representative sequences (*SI Appendix, Methods S4*). We aligned the 494 representative captains with the 1,222 tyrRs from Gluck-Thaler and Vogan (15) and Cryptons (48) as described above. Columns with ≥90% gaps were removed using ClipKIT v2.7.0 (49). We extracted the CB and CAT domains plus 50 aa flanking sites, annotated by cross-referencing the alignment with the trimmed alignment from Gluck-Thaler and Vogan (15). We constructed phylogeny with IQ-TREE as described above. We confirmed that the Tangerine-family is specific to Lecanoromycetes, by searching the unfiltered set of 10,771 captains from Gluck-Thaler and Vogan (15) and clustering these sequences with the Tangerine-family captains by 50% sequence identity and 80% overlap using MMseqs v2-13.0 (50).

The structural-based phylogenies were generated from 696 predicted models using Foldtree (24) (*SI Appendix*, Table S18), including *Starship* captains from clade 1 from Gluck-Thaler and Vogan (15), the *X. parietina Tangerine* captain, and de novo annotated captains from lichen-associated fungi. For the selected captains (*SI Appendix*, Fig. S31), we generated structural models via AlphaFold2 (42), which were then filtered by pLDDT >50. Models for pairwise superimposition were generated via AlphaFold3 (43). See details on structural predictions and selecting the proteins in *SI Appendix, Methods S5*.

### Structural Variant Annotation.

To call structural variants present in the *Tangerine* in the *X. parietina* glXanPari1 genome, we used Sniffles v2.5.2 (51). First, we mapped raw data (SRA: ERR13660094) on the genome assembly using Minimap2 v2.24-41122 (52). We sorted the read alignment with samtools v1.18 (53) and isolated the portion that corresponded to the putative *Starship* and a 200 kb flanking region on each side. Resulting alignment was analyzed with Sniffles2 using default settings.

### HGT Analysis for *Tangerine*-Associated Genes.

To test *Tangerine*-associated genes for HGT signatures, we first searched them against the NCBI nr database together with five flanking gene models on each side of the *Starship* element and 10 randomly selected gene models. Using the search results, we calculated the AI following Gladysev et al. (28), defined here as ln(best E-value of nonlecanoromycete hit + 1e^−200^) − ln(best E-value of non-Teloschistales lecanoromycete hit + 1e^−200^). To check whether the absence of certain gene models in the NCBI nr database is an artifact of genome annotation, we searched protein and transcript sequences of the likely HGT models against the NCBI core_nt and full nr/nt databases (*SI Appendix*, Table S12).

To further test HGT candidates, we used orthogroup analysis. We combined the four *Xanthoria* genomes with 104 previously published genomes (*SI Appendix*, Table S14) and analyzed them with OrthoFinder v2.5.4 (54). For the two orthogroups containing HGT candidates, we produced Maximum-Likelihood phylogenies, by realigning the sequences and reconstructing the phylogeny using IQ-TREE. See details on the HGT analysis in *SI Appendix, Methods S6*.

### HGT Analysis for *Starship* tyrRs.

To find captain clades with signatures of HGT between distantly related lichen symbionts, we first identified instances of lichen captains from different classes that clustered together during the step of clustering representative captains. Of the twelve identified instances, four were found in clades enriched in lichen-associated captains, defined as clades >70% of sequences from lichen-associated fungi. We reconstructed the phylogeny for each clade using the full unclustered set of captains. The trees were constructed using IQ-TREE as described above. Three clades showed signatures of HGT due to their discordance with the species tree. This phylogeny-based approach, rather than a sequence similarity approach like an alien index or blast-all analysis, was used to identify more ancient events of HGT with lower sequence similarity of the horizontally transferred genes.

## Supplementary Material

Appendix 01 (PDF)

## Data Availability

Genomic data including raw reads and genome assembly for *X. calcicola* are deposited in ENA (PRJEB96921; genome accession GCA_976602755.1) (55). Raw reads for *X. parietina* glXanPari20 are available at ENA as ERR14947898 (56). Starfish outputs, AlphaFold models, captain sequence alignments, sequence- and structure-based phylogenies, and genomic annotation data are available in FigShare (DOI: 10.6084/m9.figshare.30417202) (57). All scripts associated with the analysis are available at GitHub (https://github.com/metalichen/2025-Tangerine-Lichen-Starship) (58). Previously published genomic sequences used for this work and their ENA accessions are listed in *SI Appendix*, Tables S14 and S17. All other data are included in the article and/or *SI Appendix*.
