# Supplementary material for: Tangerine: A new family of Starships from lichen-forming fungi
Source: Proc Natl Acad Sci U S A. 2026 Aug 11;123(33):e2534402123. doi: 10.1073/pnas.2534402123 (PMC13486527; doi:10.1073/pnas.2534402123)
Supplement: Supplementary file 1 [file pnas.2534402123.sapp.pdf]

PNAS

Supporting Information for
Tangerine: a new family of Starships from lichen-forming fungi

Gulnara Tagirdzhanova, Noah E. Brown, Angus H. Bucknell, Ellen S. Cameron, Robert D. Finn, Mark Blaxter, Megan C. McDonald, Emile Gluck-Thaler, and Nicholas J. Talbot

Corresponding authors: Gulnara Tagirdzhanova, Emile Gluck-Thaler, Nicholas J. Talbot
Email: gulnara.tagirdzhanova@su.se, gluckthaler@wisc.edu, nick.talbot@tsl.ac.uk

This PDF file includes:

Supporting text
Figures S1 to S31
Tables S1 to S18
SI References

Supporting Information Text

Supplementary Methods

Methods S1. Genome sequencing and assembly.

To obtain a genome of *Xanthoria calcicola*, we collected a lichen sample from concrete at the Norwich Research Park (Norwich, UK; 52.623133°N, 1.221621°E). The voucher for this specimen is deposited in UC Berkeley University and Jepson Herbaria under ID UC2111092. We extracted metagenomic DNA using a NucleoBond High Molecular Weight DNA Kit. Short fragments were removed with a Circulomics Short Read Eliminator Kit with 25 kb cut-off. Remaining DNA was sequenced using PromethION Flow Cell FLO-PRO114M to 20.8 Gbp of raw data. We used Dorado v0.2 for basecalling and Flye v2.9-b1780 with flags "overlap 10K, error rate 0.005, no-alt-contigs'' for assembly. In addition, we produced short-read data from the same DNA extraction using Illumina NovaSeq 6000 platform to produce 2 Gbp of PE150 data. We used short-read data to polish the assembly with Pilon v1.23 (1). To produce the purified genomic assembly of the *X. calcicola* mycobiont, we binned the metagenomic assembly. For that, we first aligned the short reads onto the assembly with Bowtie v2.4.1 (2) and binned the assembly with MetaBAT v2.15 (3). To assess the quality of the resulting genome assembly, we used BUSCO v4.0.6 (4) with the ascomycota_odb10 database. To confirm the taxonomic identity of the fungus, we located the ITS sequence, combined it with published sequences of *Xanthoria* (Table S16), and aligned the set of sequences using MAFFT v7.271 (5). We trimmed the alignment to remove positions absent in over 90% of sequences using trimAl v1.2 (6) and reconstructed the phylogeny using IQ-TREE v2.2.2.2 (7) using 10,000 rapid bootstraps (Figure S1).

To obtain the *X. parietina* genome gIXanPari20 (isolate SAMEA115359166), we collected a lichen sample from a tree branch at the Wellcome Genome Campus (Hinxton UK; 52.07891°N, 0.184294°E). Samples were prepared for DNA extraction and Hi-C sequencing using a CryoPrep for flash frozen tissue pulverization. DNA extraction was performed using a potassium chloride extraction buffer with chloroform as highlighted in Park et al. (8). DNA extracts were sheared with a MegaRuptor at a speed of 130. Sheared DNA was SPRI cleaned up (0.88v/v concentration) with RNase to degrade any remaining RNA material. Final DNA extracts were submitted for PacBio HiFi Sequencing on the Revio platform through Scientific Operations at the Wellcome Sanger Institute. Pulverized lichen tissue was submitted for Hi-C sequencing using the Arima V2 kit on the Illumina NovaSeq X through Scientific Operations at the Wellcome Sanger Institute. PacBio HiFi reads were assembled with metaMDBG v0.3 (9). To determine assembly coverage, reads were mapped back to the assembly using minimap2 v2.23 (10). Contigs were binned with MetaBAT2 v2.12.1 (3) and bins were assessed for quality using EukCC v2.1.2 (11) and BUSCO v5.7.0 (4). Final scaffolds were generated using the Arima Hi-C mapping pipeline (https://github.com/ArimaGenomics/mapping_pipeline/blob/master/Arima_Mapping_UserGuide_A160156_v03.pdf) and yahs v1.2 (12).

Methods S2. Genome annotation.

Four genomes were analyzed: *X. parietina* isolate GTX0501 from Tagirdzhanova et al. (13) (PRJEB78723; genome accession GCA_964263705.1), *X. parietina* gIXanPari1 from the isolate SAMEA111342678 (PRJEB83421; genome accession GCA_964656405.1), and the two genomes described above. The genomes were annotated using Funannotate v1.8.15 (14). For *X. parietina* GTX0501 we re-used the annotations from Tagirdzhanova et al. (13) the remaining genomes were annotated in the same way as follows. The genomes were repeat-masked using stand-alone RepeatMasker v4.1.2 (https://www.repeatmasker.org/). For *X. calcicola* and *X. parietina* GTX0501 we used repeat libraries created with RepeatModeler v2.0.3 (15) with the -LTRStruct flag; for the other *X. parietina* isolates, we re-used the library produced for GTX0501. Next, we ran the 'funannotate predict' module, which used Genemark-ES v4.62 (16), Augustus v3.3.2 (17), CodingQuarry v2.0 (18), GlimmerHMM v3.0.4 (19) for *de novo* gene prediction, created consensus models with EVidence Modeler v1.1.1 (20), and annotated tRNA with tRNAscan-SE v2.0.9 (21). To produce functional annotations, we used the 'funannotate annotate'

module and following databases: PFAM v35.0 (22), UniProtDB v2023_01 (23), MEROPS v12.0 (24), dbCAN v11.0 (25), and BUSCO ascomycota_odb10 (4). Separately, we ran InterProScan v5.42-78.0(26) and the EMapper webserver (http://eggnog-mapper.embl.de/) (27). For family-level annotation of repeat elements, we used EarlGrey v5.1.1 (28) with the Dfam v3.8 (29). To annotate genome regions affected by repeat-induced point mutations we used RIPper (30). To visualize GC-content of the genomes, we used seqkit v0.12.0 (31). To visualize global synteny, we used GENESPACE v1.3.1 (32).

Methods S3. Annotation and characterization of *Starships*.

To detect potential *Starships*, we used Starfish v1.0.0 (33) following the tutorial (https://github.com/egluckthaler/starfish/wiki/Step-by-step-tutorial). Briefly, we ran 'starfish annotate' and 'starfish consolidate' modules to detect tyrosine recombinases. Next, we ran 'starfish insert' and 'starfish summarize' to identify insertion sites and element boundaries, and 'starfish dereplicate' to assign putative elements to genomic regions. To visualize the elements we used the 'starfish locus-viz' module and gggenomes v1.0.1 (34). The region alignments were produced with MUMmer v4.0.0 (35). To assess read coverage of the *Starship* element in *X. parietina* glXanPari1, we mapped raw data (SRA: ERR13660094) on the genome assembly using Minimap2 v2.24-41122 (10) and visualized the alignment with IGV (36). To align the element ends, we used MAFFT v7.271 (5). To locate *Tangerine* in *X. calcicola* and *X. parietina* GTX0501, we searched for sequences similar to *Tangerine*-associated genes in the genomic assemblies and raw metagenomic reads using blast+-2.9.0 (37). We also searched for homologues of *Tangerine*-associated genes in the short-read metagenome-assembled genomes of *X. parietina* and visualized the gene expression data from our earlier study (13). To locate *Tangerine* and the flanking genes in the short-read genomes of *Xanthoria* from Llewellyn et al. (38) (JALAII01 and JALAIA01), we used results of the orthogroup analysis (see description of the HGT analysis). To compare proteins encoded within *Tangerine* in different genomes, we used blast+-2.9.0 (37).

To confirm the transcript annotation for the *Tangerine*-associated genes, we aligned two metatranscriptomic libraries from *X. parietina* (SRA: ERR4235132 and ERR4235133) to each *X. parietina* genome using STAR v2.5.4b (39) and cross-referenced the exon boundaries with the RNA alignments. This way, we corrected exon boundaries in the captain genes in *X. parietina* glXanPari1 (XANPAOZ2100_002162-T1) and glXanPari20 (XANPARI20_008752-T1) (Fig. S30). Both the initial annotation and the RNA-corrected version differed from the annotation produced for this gene model by Starfish (33), which relied on MetaEuk (40) for de novo gene prediction. Starfish predicted an extra exon at the N-terminus of the protein, which however was not supported by the RNA data and not included in the final gene model (Fig. S30).

To test that the CB domain and a portion of CAT domain is genuinely truncated in the *Tangerine* captains, we attempted to find additional exons that would cover the 'missing' portion of the *Tangerine* captain. We used a tBLASTn search of the *Hephaestus* captain HhpA against the nucleotide sequence of the *Tangerine* element, which yielded no additional hit outside of the *Tangerine* captain gene model.

Methods S4. Producing a set of representative captains from the larger set of genomes from lichen-associated fungi.

We used 'starfish annotate' module from Starfish v1.1.0 (33) to annotate captains in genomes of lichen-associated fungi from Lecanoromycetes, Eurotiomycetes, Dothideomycetes, and Arthionomycetes from NCBI (Table S17). A total of 4,128 putative captains were identified. We added the captain sequences from *X. parietina* and *X.calcicola* to this dataset and clustered it to produce putative active representative captain sequences, following Gluck-Thaler and Vogan (33). First, captain sequences shorter than 203 amino acids (n=1,481) were removed. Next, we removed putative pseudogenes defined as sequences missing >1 of the six conserved active sites in tyrRs (n=1,139), excluding the *Tangerine* element captains from *X. parietina*. To annotate active sites, we aligned the lichen captains to representative sequences of tyrRs from Gluck-Thaler and Vogan (33) using MAFFT v7.271 (5) and cross-referenced the alignment with

annotated active sites. A large cluster of sequences, including the *Tangerine* captains, had a different column for active sites 3 and 4 (Table S10); filtering for this cluster was done separately. We clustered the remaining lichen captains (n=1,508) into representative sequences using MMseqs v2-13.0 (41). First, we used 'easy-clust' to cluster sequences by 90% sequence identity and 80% overlap with the longest seed sequence of the cluster. The resulting representative sequences were clustered again, by 50% sequence identity and 80% overlap.

Methods S5. Constructing phylogenies based on protein structural predictions. The structural-based phylogenies were generated from a set of 696 predicted models using Foldtree (42). Initially a set of 745 captains was chosen combining a manually selected representative group of *Starship* captains from clade 1 from Gluck-Thaler and Vogan (43), the *X. parietina Tangerine* captain XANPAGTX0501_001716-T1, and a set of *de-novo* annotated representative captains from lichen-associated fungi. To select the *de-novo* annotated representatives, the set of 2,684 captains from lichen-associated fungi was filtered to sequences 203–999 (inclusive) residues in length and then clustered by 30% similarity using MMseq2. This resulted in 453 representatives which were then combined into the final set to make 745 captains total (Fig. S31). Structural models of the final set of captains were generated via AlphaFold2 (44), with four failing predictions. The resulting models were then filtered by pLDDT > 50, leaving the 696 models used as input for Foldtree (Table S18). Models for pairwise superimposition were generated via AlphaFold3 (45). For each protein, five predictions were generated and the most confident model was chosen based on pTM and average pLDDT. Model superimposition and RMSD values were generated via the matchmaker function in ChimeraX (46) with the default settings. The PAE plots were visualised using PAE Viewer (47).

Methods S6. HGT analysis for Tangerine-associated genes. To test *Tangerine*-associated genes for HGT signatures, we first searched them against the NCBI nr database using BLASTp web interface (accessed 2025/03/21). As controls, we included five flanking gene models on each side of the *Starship* element and 10 randomly selected gene models. Using the search results for each gene, we calculated the AI following Gladysev et al. (48). We defined AI as ln(best E-value of non-lecanoromycete hit + $1e^{-200}$) - ln(best E-value of non-Teloschistales lecanoromycete hit + $1e^{-200}$). To check whether the absence of certain gene models in the NCBI nr database is an artifact of genome annotation, we searched likely-HGT models using tBLASTn web interface for the protein sequences (accessed 2025/05/24) and BLASTn for the transcript sequences (accessed 2025/10/10) against the NCBI core_nt (with no taxonomic restriction) and full nr/nt (restricted to Lecanoromycetes) databases (Table S12).

To further test HGT candidates, we used orthogroup analysis. We combined the four *Xanthoria* genomes with 104 previously published genomes (Table S14) and analyzed them with OrthoFinder v2.5.4(49). Species and gene trees were visualized using iTOL v7(50). We rooted the trees using *Saccharomyces cerevisiae* and *Lipomyces starkeyi*. Using MMseqs v2-13.0 (41), we crossed-mapped predicted proteome of *X. parietina* glXanPari1 and proteomes of two fungi: *Exophiala xenobiotica* (GCA_035930045) and *Icmadophila ericetorum* (GCA_022814295).

For the two orthogroups containing HGT candidates, we produced Maximum-Likelihood phylogenies. For each orthogroup, we aligned the sequences with MAFFT v7.271 (5), removed columns with >80% gaps using trimAl v1.2 (6), and reconstructed the phylogeny using IQ-TREE v2.2.2.2 (7) using 10,000 rapid bootstraps.

Figures

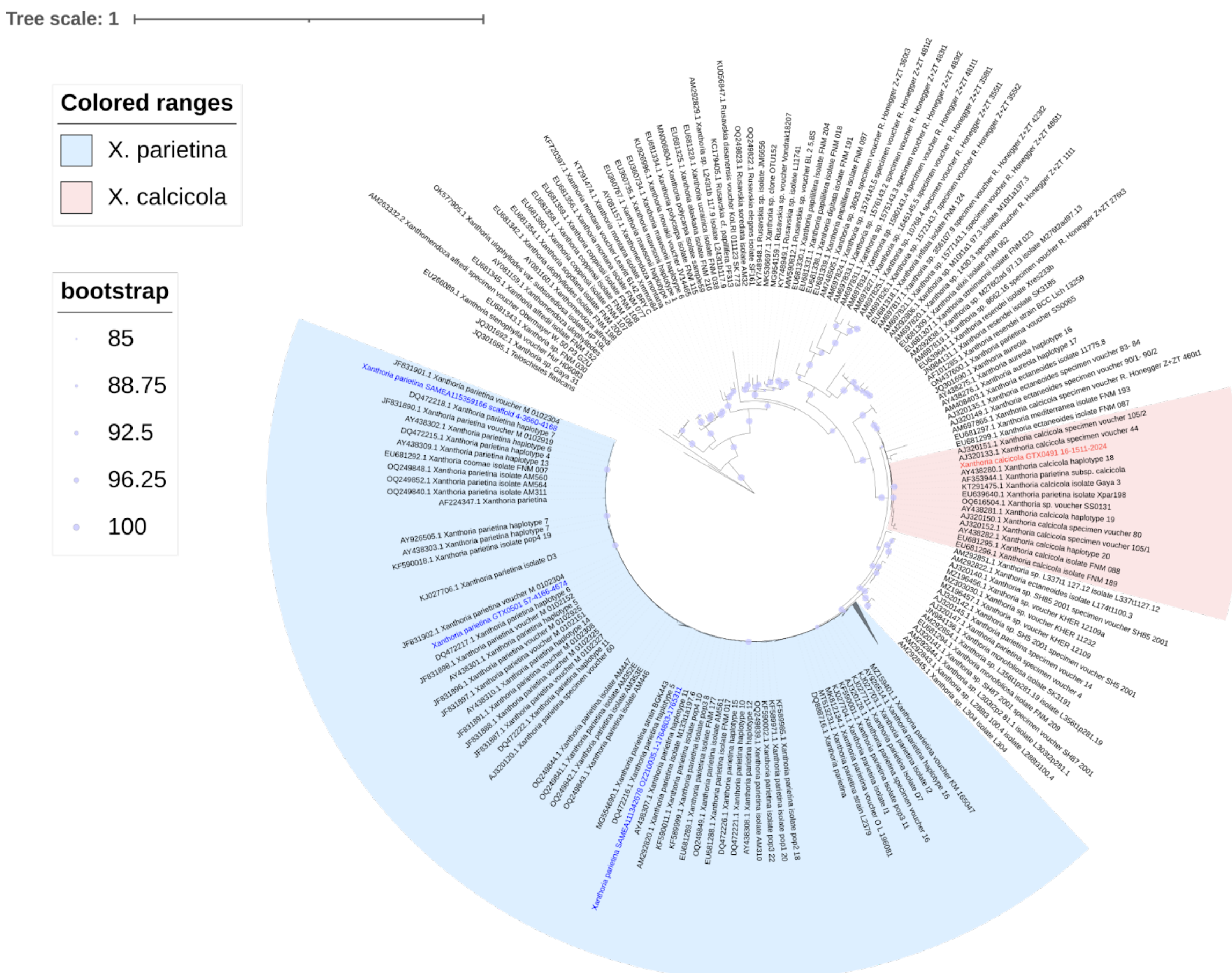

Fig. S1. Maximum-likelihood phylogeny based on internal transcribed spacer (ITS) sequences. We combined sequences from literature (Table S16) with ITS sequences extracted from the four *Xanthoria* genomes included in this analysis (highlighted in blue for *X. parietina* and in red for *X. calcicola*). Clades corresponding to *X. parietina* and *X. calcicola* are highlighted.

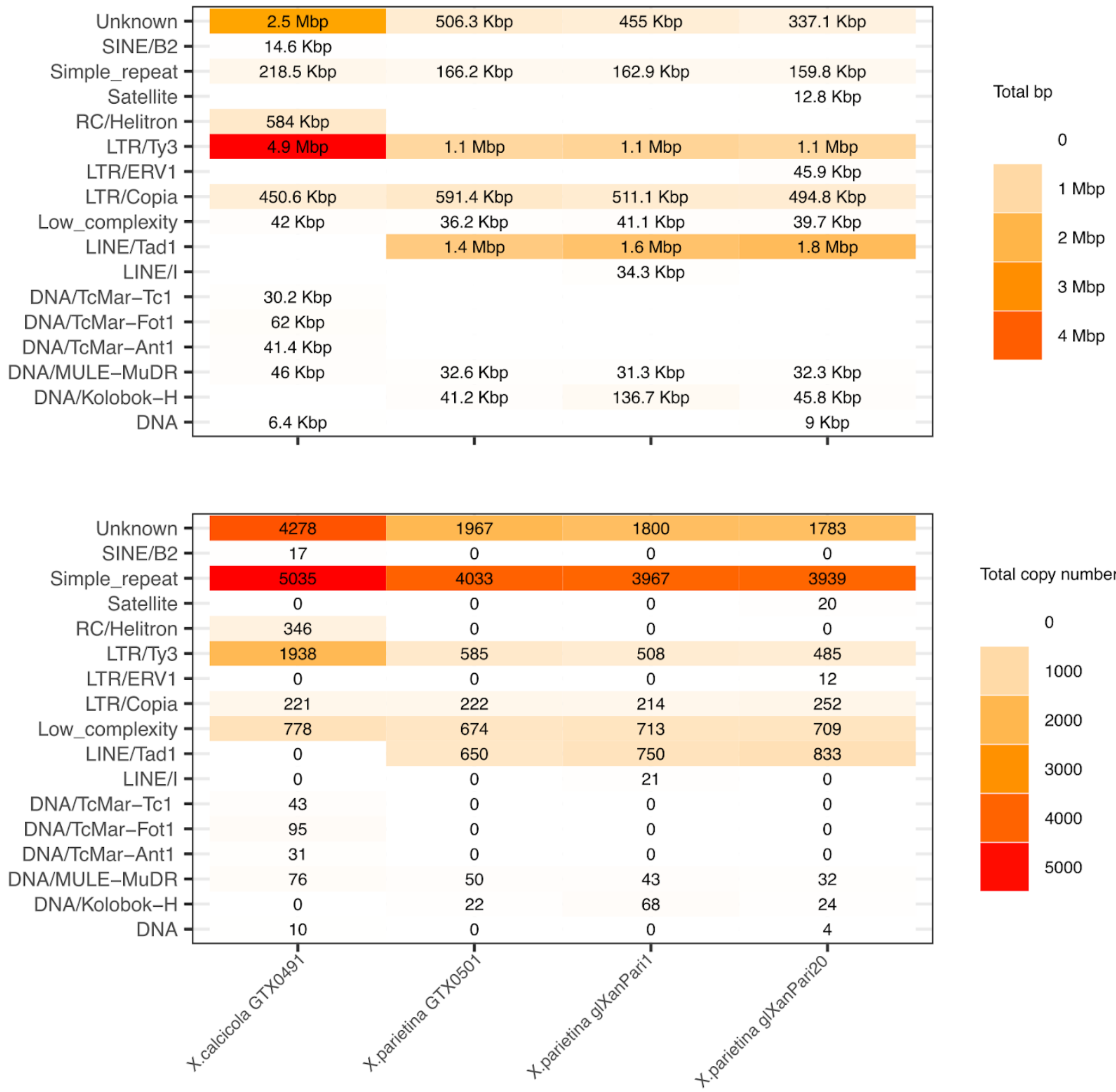

	X.calcicola GTX0491	X.parietina GTX0501	X.parietina glXanPari1	X.parietina glXanPari20
Unknown	4278	1967	1800	1783
SINE/B2	17	0	0	0
Simple_repeat	5035	4033	3967	3939
Satellite	0	0	0	20
RC/Helitron	346	0	0	0
LTR/Ty3	1938	585	508	485
LTR/ERV1	0	0	0	12
LTR/Copia	221	222	214	252
Low_complexity	778	674	713	709
LINE/Tad1	0	650	750	833
LINE/I	0	0	21	0
DNA/TcMar–Tc1	43	0	0	0
DNA/TcMar–Fot1	95	0	0	0
DNA/TcMar–Ant1	31	0	0	0
DNA/MULE–MuDR	76	50	43	32
DNA/Kolobok–H	0	22	68	24
DNA	10	0	0	4

Fig. S2. Repeat superfamilies present in the four *Xanthoria* genomes. Repeats were annotated and classified using Earl Grey. The top panel shows total genomic coverage (bp) for each superfamily, summed across all representatives. The bottom panel shows total copy number for each superfamily, summed across all representatives.

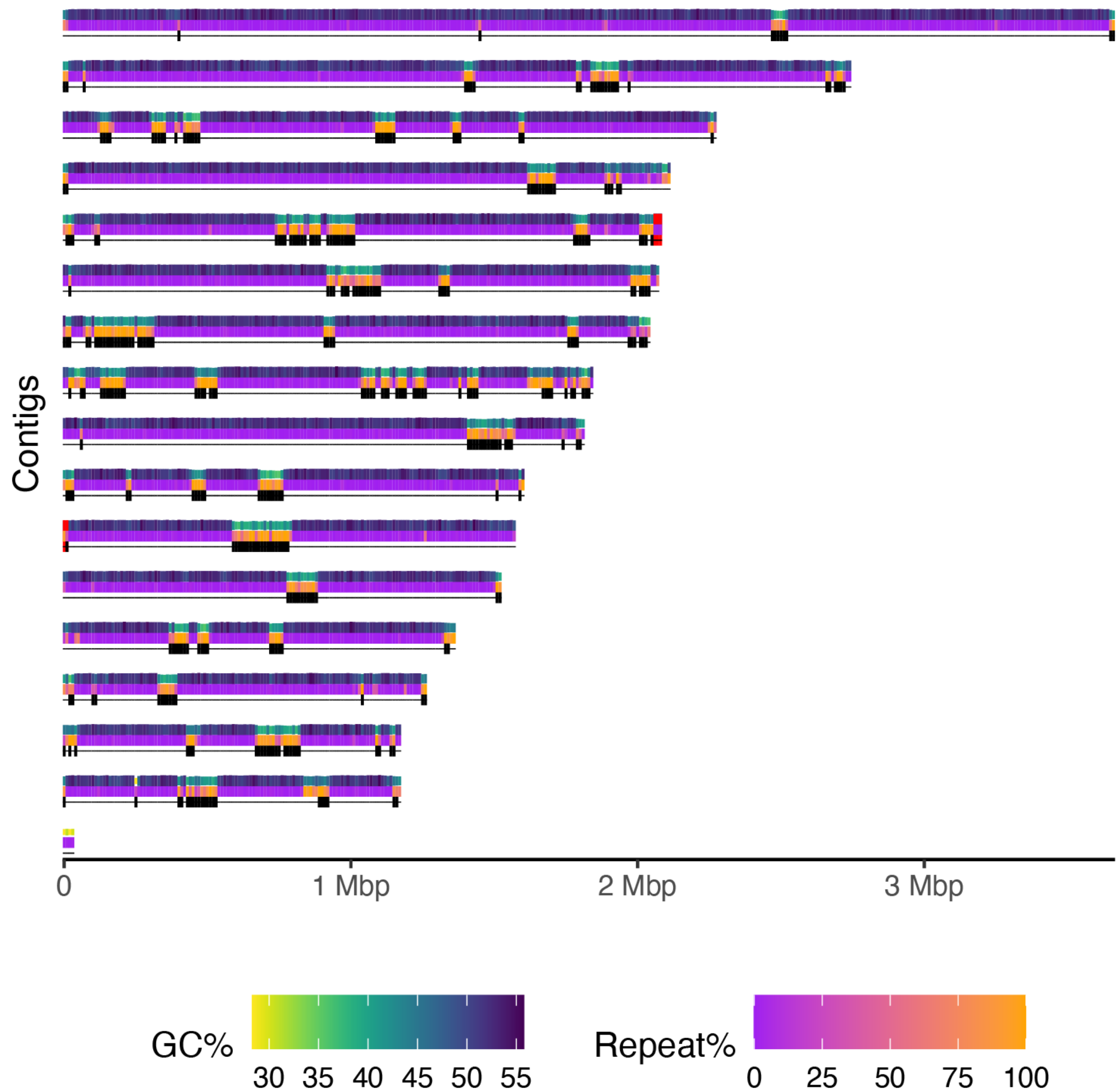

Fig. S3. The nuclear genome of *X. parietina* isolate gIXanPari1. Each bar represents a contig arranged by the decreasing length, divided into three annotation tracks: GC content, repeat content, and presence of large RIP-affected regions (LRARs). Red bars at the contig ends show telomeric repeats. The x axis shows contig length.

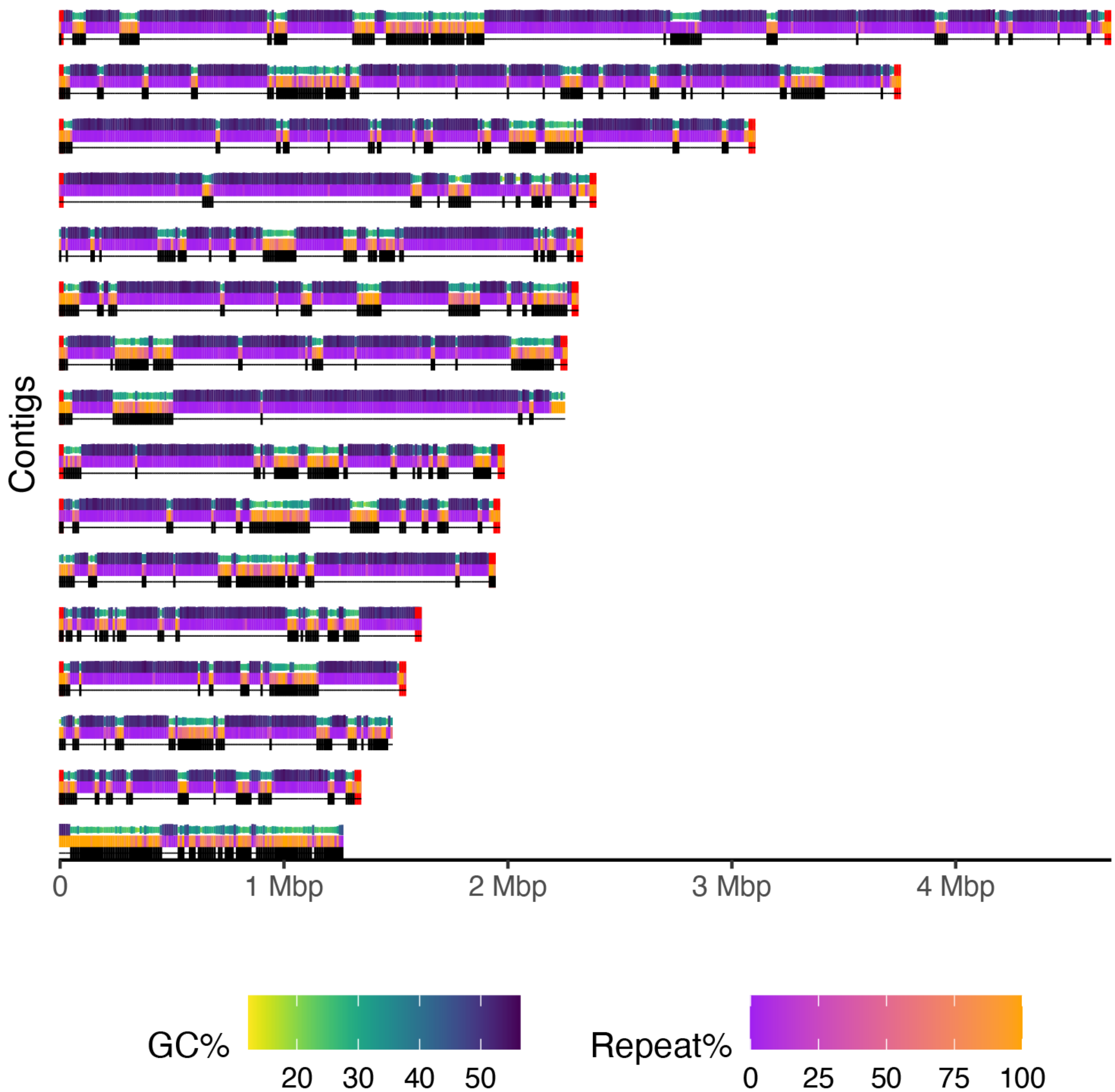

Fig. S4. The nuclear genome of *X. calcicola* isolate GTX0491. Each bar represents a contig arranged by the decreasing length, divided into three annotation tracks: GC content, repeat content, and presence of large RIP-affected regions (LRARs). Red bars at the contig ends show telomeric repeats. The x axis shows contig length.

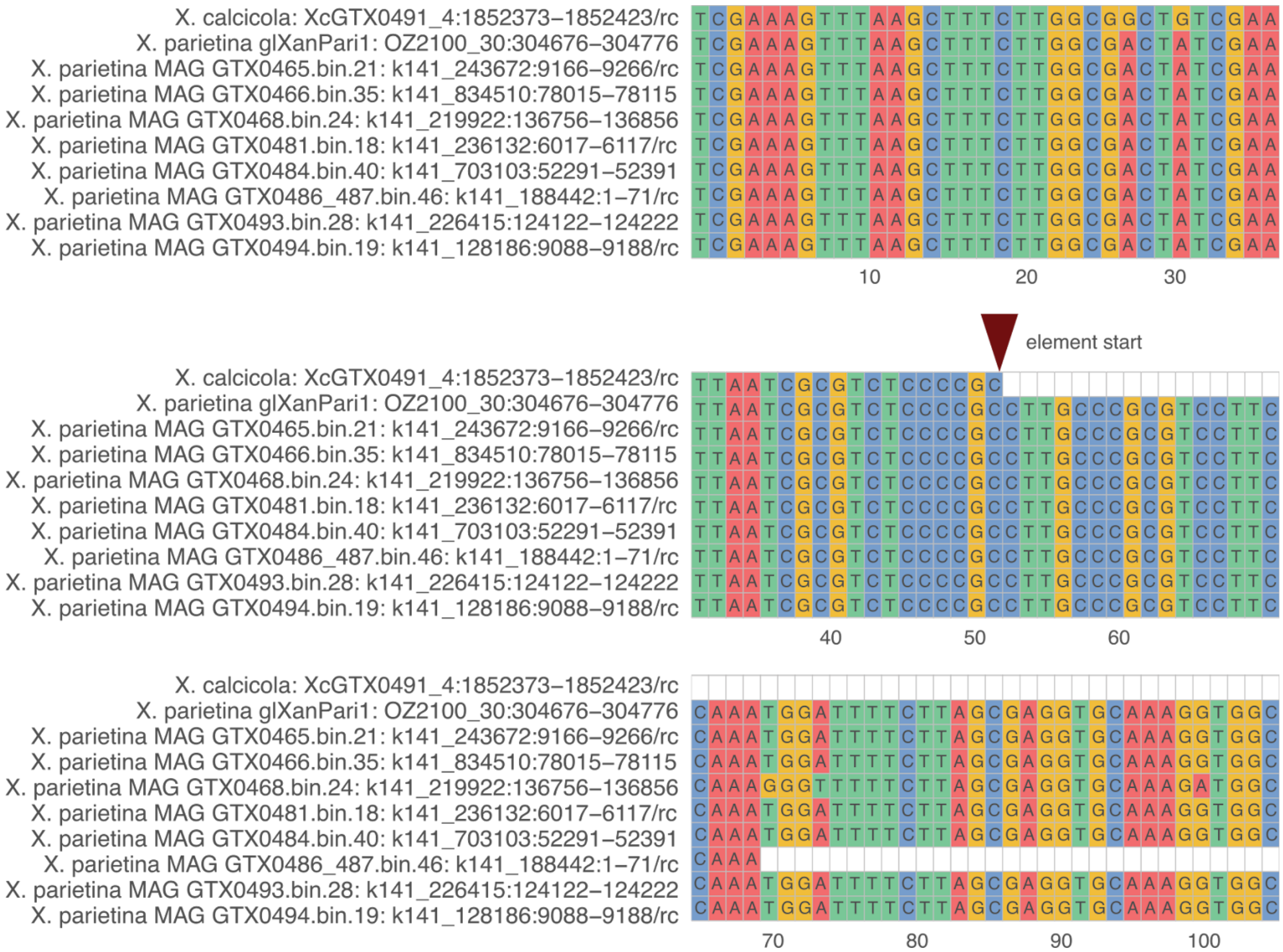

Fig. S5. Alignment of the first 50 bp of *Tangerine* and 50 bp on the 5' flank in the short-read metagenome-assembled genomes (MAGs) of *X. parietina* from Tagirdzhanova et al. (2025). The first two sequences represent the empty site in *X. calcicola* and the corresponding region in *X. parietina* glXanPari1 shown for context. The remaining eight sequences show the 5' end of *Tangerine* in the MAGs. In one of the MAGs (GTX0486_487.bin.46), the region of interest was located at the end of the contig, thus explaining the gap in the alignment. In all MAGs except for GTX0486_487.bin.46 and GTX0468.bin.24, the same contig that contained the 5' end of *Tangerine* also encoded a tyrosine recombinase with >99% similarity to the *Tangerine* captain from *X. parietina* glXanPari1 (XANPAOZ2100_002162-T1). In GTX0468.bin.24, the corresponding contig encoded a tyrosine recombinase with 68% similarity to XANPAOZ2100_002162-T1. In GTX0468.bin.24, we failed to get a match to XANPAOZ2100_002162-T1.

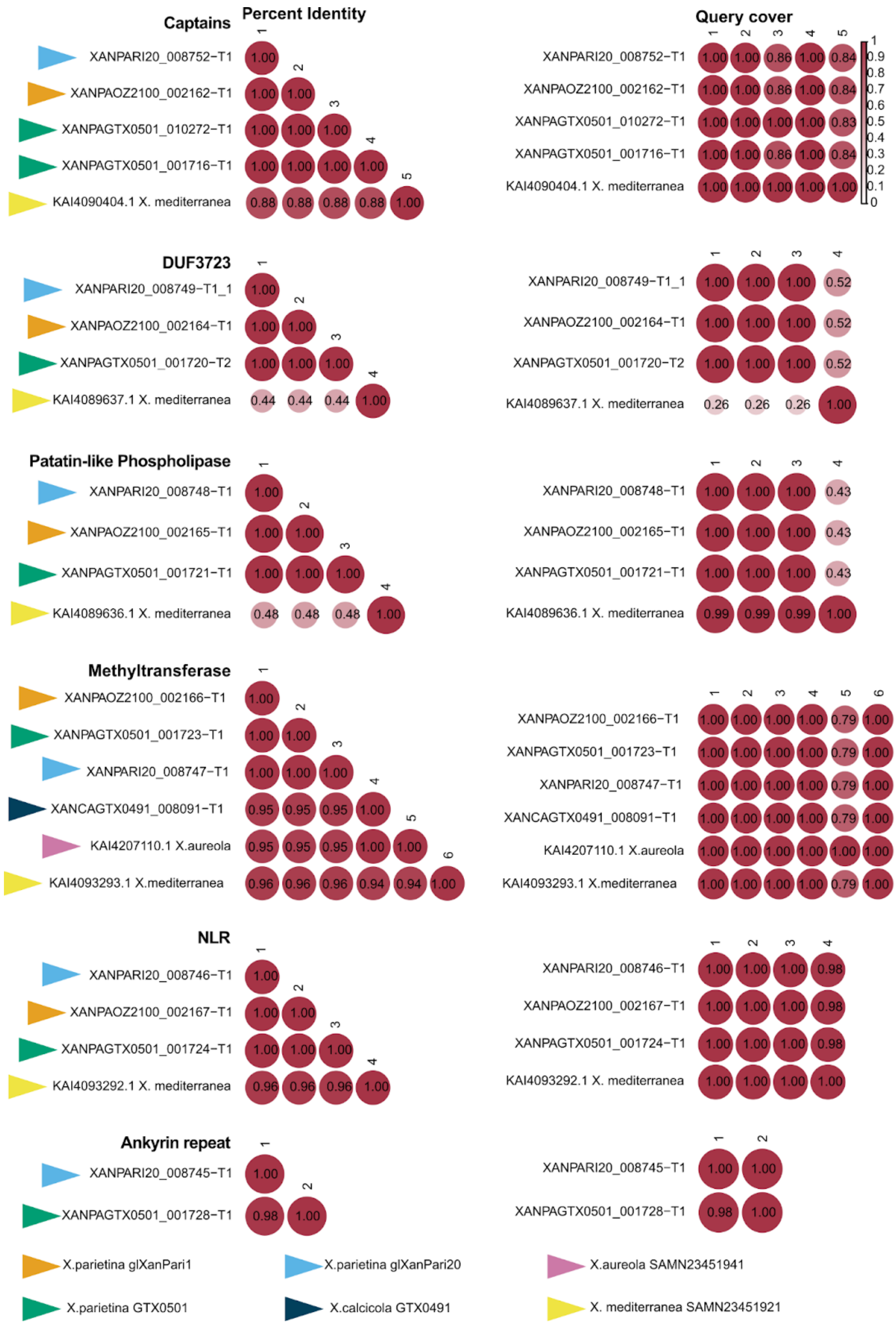

Percent Identity
Query cover
Captains
XANPARI20_008752−T1
XANPAOZ2100_002162−T1
XANPAGTX0501_010272−T1
XANPAGTX0501_001716−T1
KAI4090404.1 X. mediterranea
1.00
1.00 1.00
1.00 1.00 1.00
1.00 1.00 1.00 1.00
0.88 0.88 0.88 0.88 1.00
1.00 1.00 0.86 1.00 0.84
1.00 1.00 0.86 1.00 0.84
1.00 1.00 1.00 1.00 0.83
1.00 1.00 0.86 1.00 0.84
1.00 1.00 1.00 1.00 1.00
1 0.9 0.8 0.7 0.6 0.5 0.4 0.3 0.2 0.1 0
DUF3723
XANPARI20_008749−T1_1
XANPAOZ2100_002164−T1
XANPAGTX0501_001720−T2
KAI4089637.1 X. mediterranea
1.00
1.00 1.00
1.00 1.00 1.00
0.44 0.44 0.44 1.00
1.00 1.00 1.00 0.52
1.00 1.00 1.00 0.52
1.00 1.00 1.00 0.52
0.26 0.26 0.26 1.00
Patatin-like Phospholipase
XANPARI20_008748−T1
XANPAOZ2100_002165−T1
XANPAGTX0501_001721−T1
KAI4089636.1 X. mediterranea
1.00
1.00 1.00
1.00 1.00 1.00
0.48 0.48 0.48 1.00
1.00 1.00 1.00 0.43
1.00 1.00 1.00 0.43
1.00 1.00 1.00 0.43
0.99 0.99 0.99 1.00
Methyltransferase
XANPAOZ2100_002166−T1
XANPAGTX0501_001723−T1
XANPARI20_008747−T1
XANCAGTX0491_008091−T1
KAI4207110.1 X.aureola
KAI4093293.1 X.mediterranea
1.00
1.00 1.00
1.00 1.00 1.00
0.95 0.95 0.95 1.00
0.95 0.95 0.95 1.00 1.00
0.96 0.96 0.96 0.94 0.94 1.00
1.00 1.00 1.00 1.00 0.79 1.00
1.00 1.00 1.00 1.00 0.79 1.00
1.00 1.00 1.00 1.00 0.79 1.00
1.00 1.00 1.00 1.00 0.79 1.00
1.00 1.00 1.00 1.00 1.00 1.00
1.00 1.00 1.00 1.00 0.79 1.00
NLR
XANPARI20_008746−T1
XANPAOZ2100_002167−T1
XANPAGTX0501_001724−T1
KAI4093292.1 X. mediterranea
1.00
1.00 1.00
1.00 1.00 1.00
0.96 0.96 0.96 1.00
1.00 1.00 1.00 0.98
1.00 1.00 1.00 0.98
1.00 1.00 1.00 0.98
1.00 1.00 1.00 1.00
Ankyrin repeat
XANPARI20_008745−T1
XANPAGTX0501_001728−T1
1.00
0.98 1.00
1.00 1.00
0.98 1.00
X.parietina glXanPari1
X.parietina glXanPari20
X.aureola SAMN23451941
X.parietina GTX0501
X.calcicola GTX0491
X. mediterranea SAMN23451921

Fig. S6. Blast alignments between sequences of proteins encoded within *Tangerine*. For *X. parietina* glXanPari1 and glXanPari20, only sequences within the *Tangerine* are included; for the other genomes the proteins were selected based on their orthogroup assignments. We aligned sequences from each orthogroup in pair-wise combinations using blast, and visualized percent identity (left column) and query coverage (right column) for each pair. Arrows show which genome the protein originated from. This figure does not show gene models that lacked functional annotation. For the *Tangerine* captain in X. parietina GTX501, we show two gene models, which were present in the genome due to the fragmentation of *Tangerine* onto two contigs.

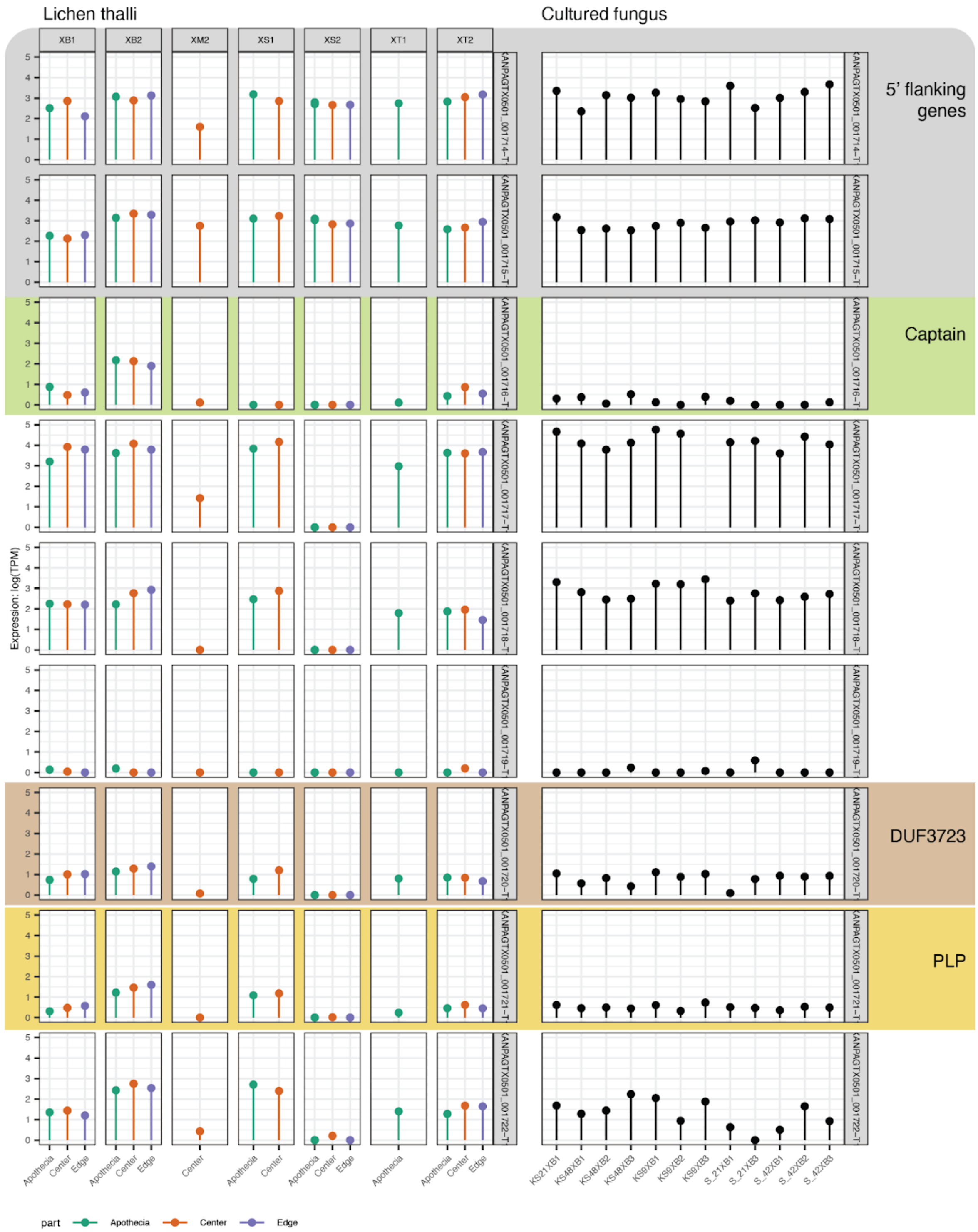

Fig. S7. Expression of *Tangerine*-associated genes and flanking genes in lichen thalli (left panel) and fungus culture (right panel), part 1. Lichen thalli samples are grouped based on the thallus and colored based on the developmental stage. Lichen thallus XS2 shows near-zero levels of genes expression for all *Tangerine*-associated genes. The data are taken from Tagirdzhanova et al. (2025; PRJEB38537). TPM stands for transcripts per million, PLP stands for Patatin-like phospholipase, NLR stands for NOD-like receptor.

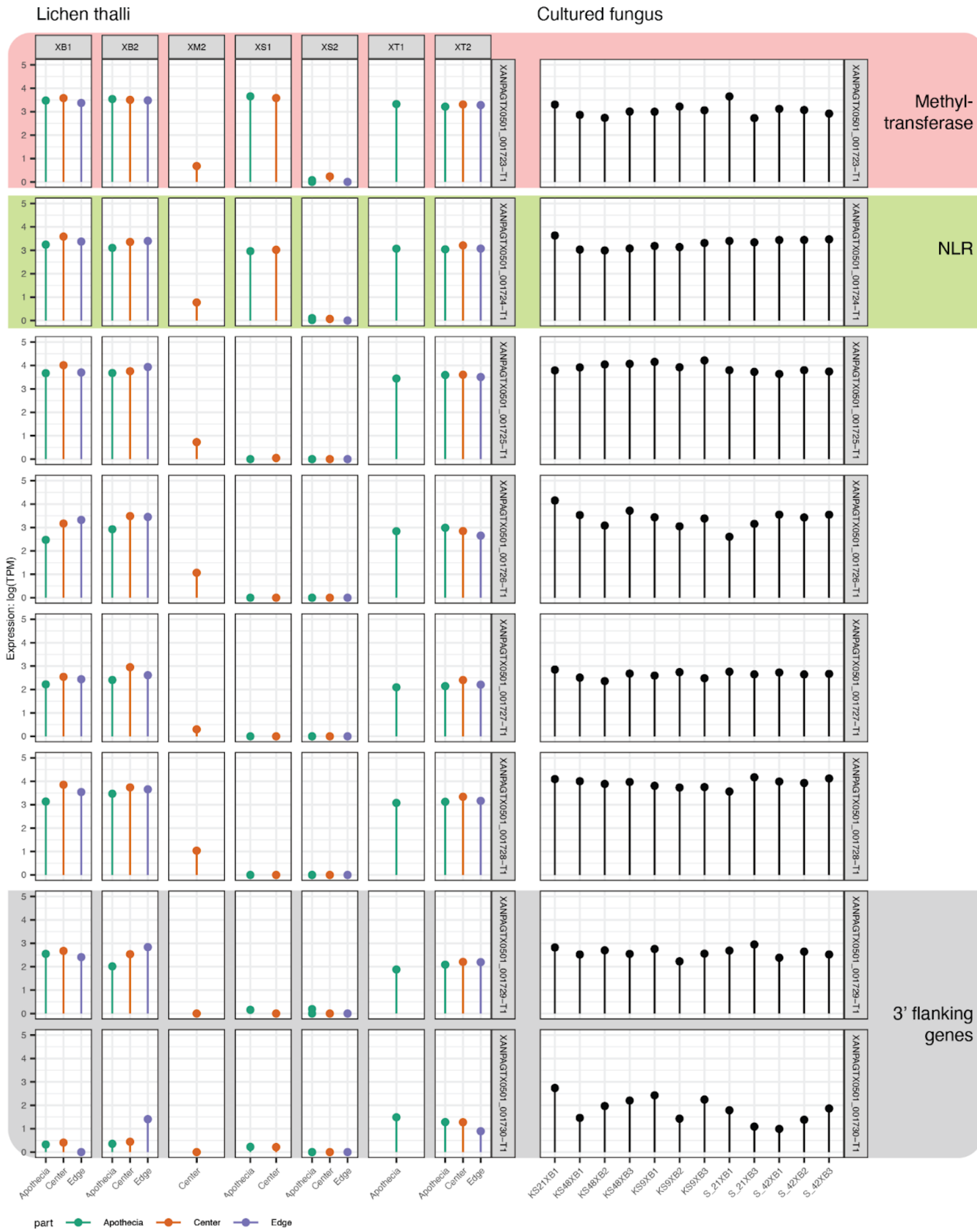

Fig. S8. Expression of *Tangerine*-associated genes and flanking genes in lichen thalli (left panel) and fungus culture (right panel), part 2. Lichen thalli samples are grouped based on the thallus and colored based on the developmental stage. Lichen thallus XS2 shows near-zero levels of genes expression for all *Tangerine*-associated genes. The data are taken from Tagirdzhanova et al. (2025; PRJEB38537). TPM stands for transcripts per million, PLP stands for Patatin-like phospholipase, NLR stands for NOD-like receptor.

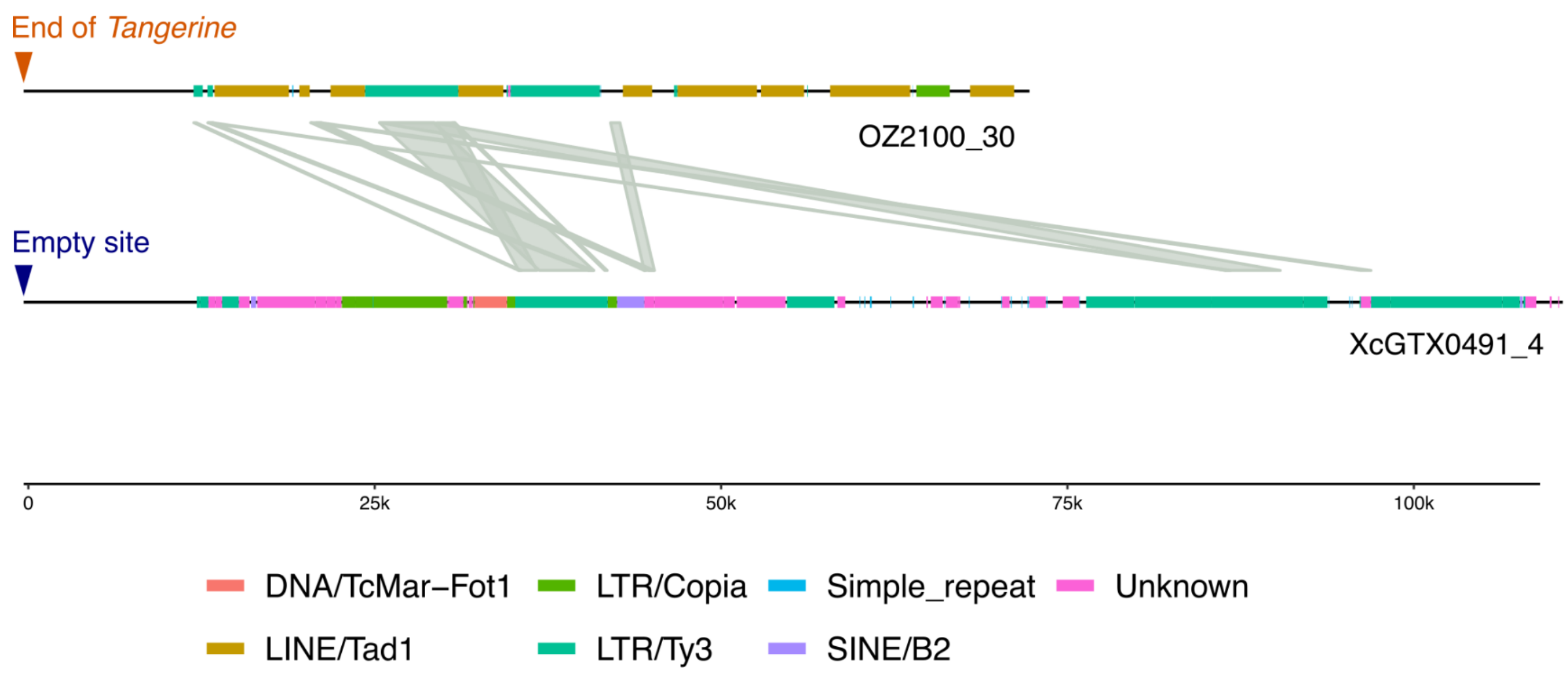

Fig. S9. Alignment of the LRARs (large RIP-affected regions) downstream of *Tangerine* in the genome of *X. parietina* glXanPari1 (upper track) and downstream of the corresponding empty site in the genome of *X. calcicola* GTX0491 (lower track). Links between the fragments indicate alignments generated with BLASTn. Repeat element types are indicated with different color tracks. Repeats were annotated using Earl Grey.

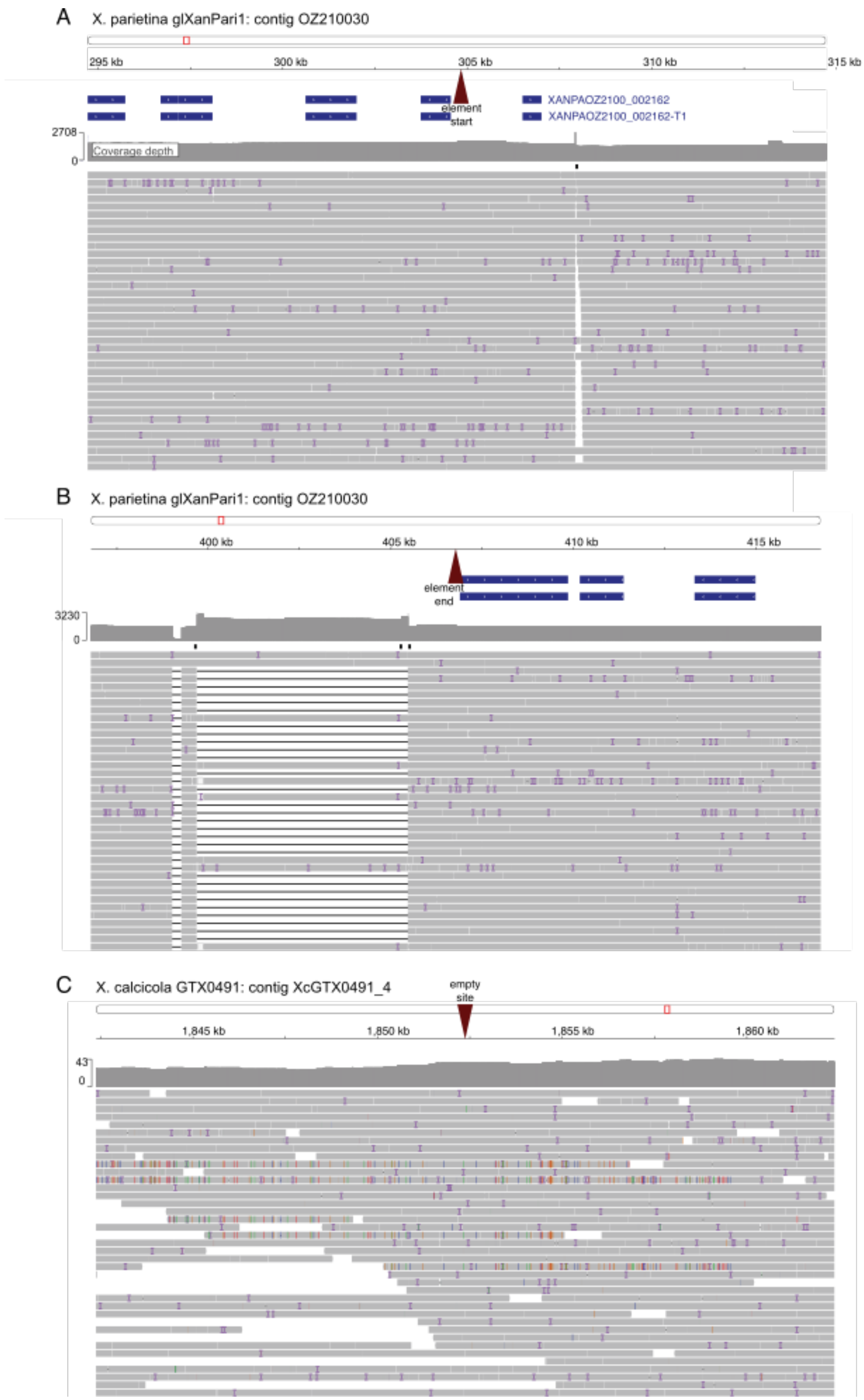

A
X. parietina glXanPari1: contig OZ210030
295 kb
300 kb
305 kb
310 kb
315 kb
element start
XANPAOZ2100_002162
XANPAOZ2100_002162-T1
2708
0
Coverage depth
B
X. parietina glXanPari1: contig OZ210030
400 kb
405 kb
410 kb
415 kb
element end
3230
0
C
X. calcicola GTX0491: contig XcGTX0491_4
empty site
1,845 kb
1,850 kb
1,855 kb
1,860 kb
43
0

Fig. S10. Long-read data mapped to the genomic assemblies. The upper track represents the entire contig with the red rectangle showing the position of the 20 Kbp area shown below. Gene annotations (upper track) and transcripts (lower track) are shown in blue. The grey track below shows the depth of coverage. Black rectangles under the coverage track indicate structure variants. Below, is shown a subset of reads mapped to the genome fragment. **A.** Start of *Tangerine* element on *X. parietina* glXanPari1 and 10 Kbp flanking regions. **B.** End of *Tangerine* element on *X. parietina* glXanPari1 contig and 10 Kbp flanking regions. **C.** Empty site in *X. calcicola* genome and 10 Kbp flanking regions.

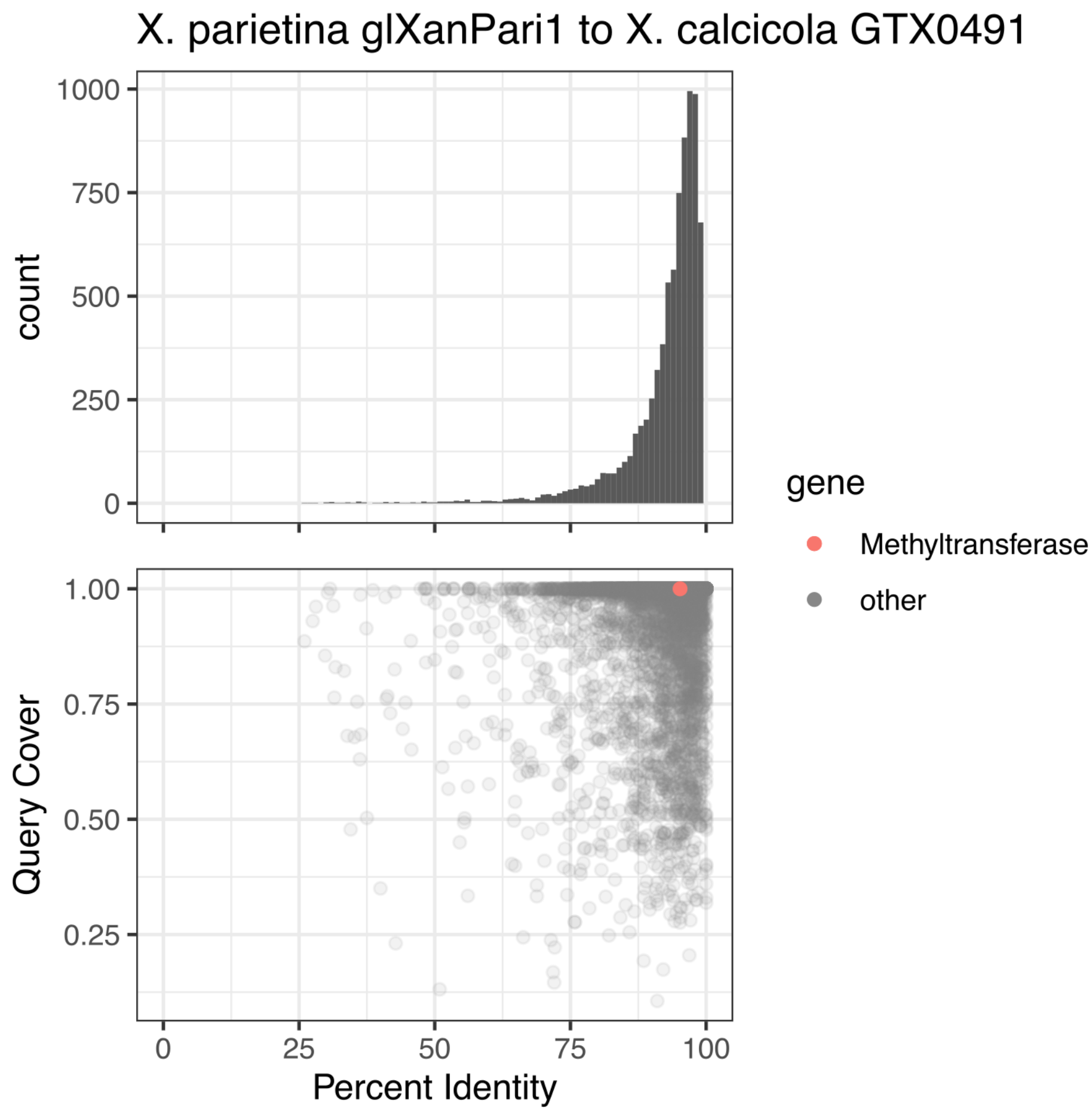

Fig. S11. Cross-mapping of the predicted proteome of *X. parietina* glXanPari1 to *X. calcicola* GTX0491. For each *X. parietina* glXanPari1 predicted protein, we identified the best match in the other predicted proteome. The top panel shows the distribution of percentage identity values across all matches; the bottom panel shows how the matches were positioned in relation to percentage identity and query cover. Methyltransferase is highlighted in color.

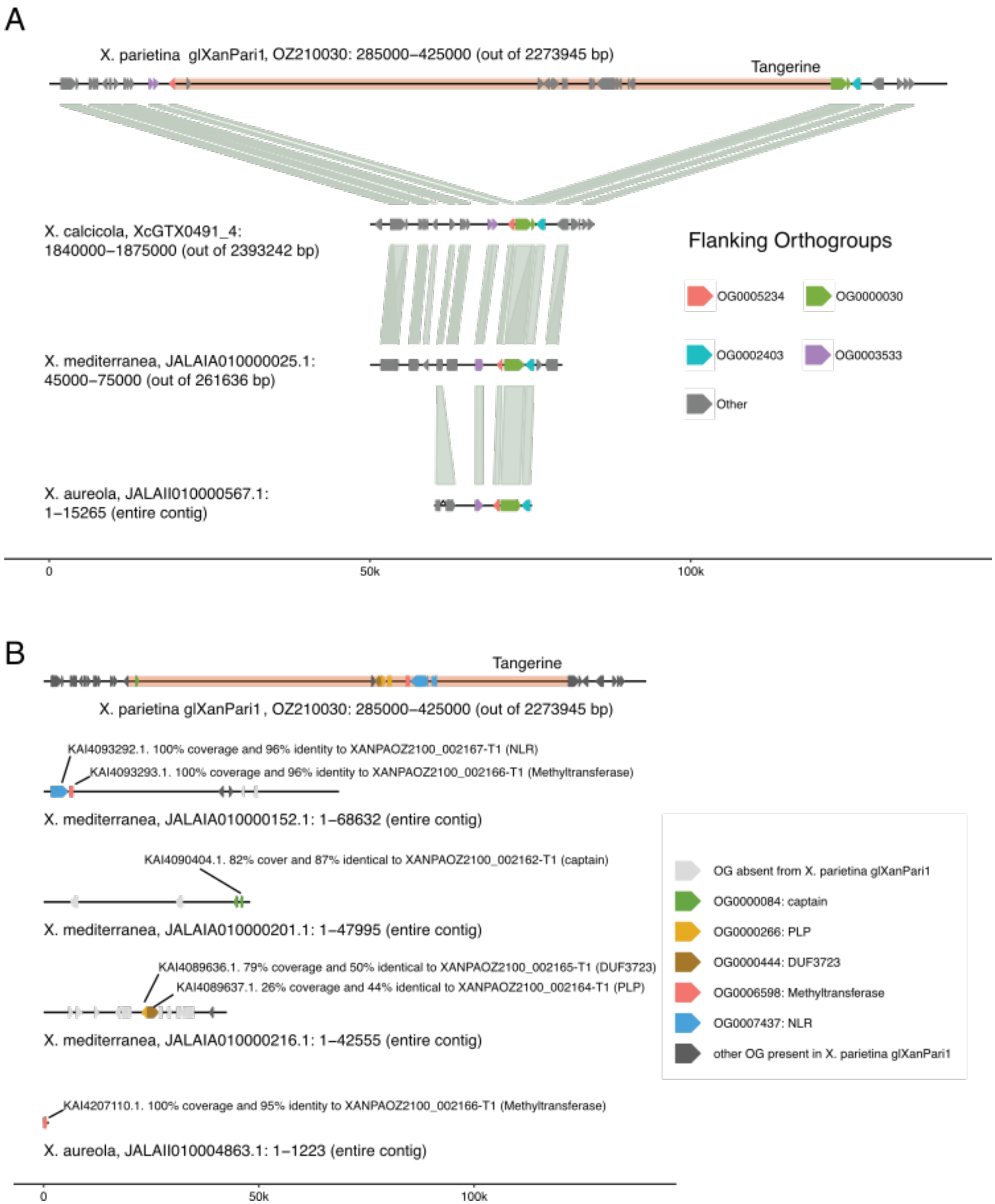

Fig. S12. *Tangerine* in the short-read genome assemblies of *X. mediterranea* and *X. aureola*. A. Alignment of the genomic region containing *Tangerine* in *X. parietina* glXanPari1 to the empty site in the genomes of *X. calcicola* GTX0491, *X. mediterranea*, and X. *aureola*. Genes are shown as arrows; the four flanking orthogroups are highlighted. The links connecting two genes indicate that these belong to the same orthogroup. B. *Tangerine*-associated genes in *X. parietina* glXanPari1 and in *X. mediterranea* and *X. aureola*. Orthogroups containing the captain,

accessory and cargo genes are highlighted in different colors. For each gene from *X. mediterranea* and *X. aureola* similarity scores are listed between the predicted protein and the corresponding predicted protein from *X. parietina* glXanPari1.

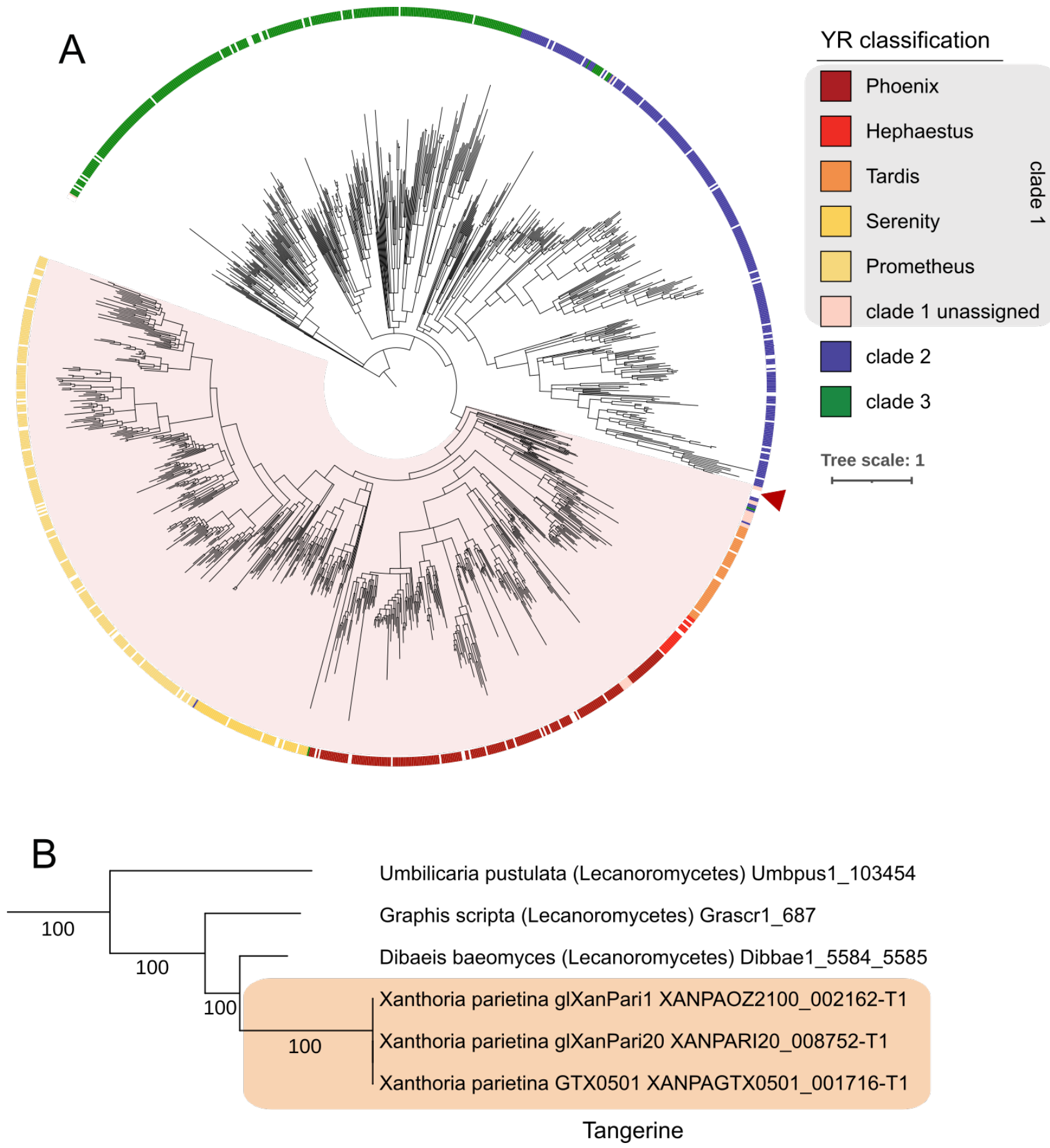

Fig. S13. Tangerine captain in the context of the tyrosine recombinase (tyrR) typology. A. Sequence-based captain (*Starship* tyrR) tree with the *Tangerine* captain indicated with the arrow. *Tangerine* captain sequences were aligned with 1222 predicted captains; the alignment was used to recreate maximum-likelihood phylogeny. Clades with <80 bootstrap support are collapsed. The outer color track shows captain family assignments; clade 1 is highlighted as a pink block. B. Magnified clade of Lecanoromycetes sequence including the *Tangerine* captain. Bootstrap support shown next to the nodes.

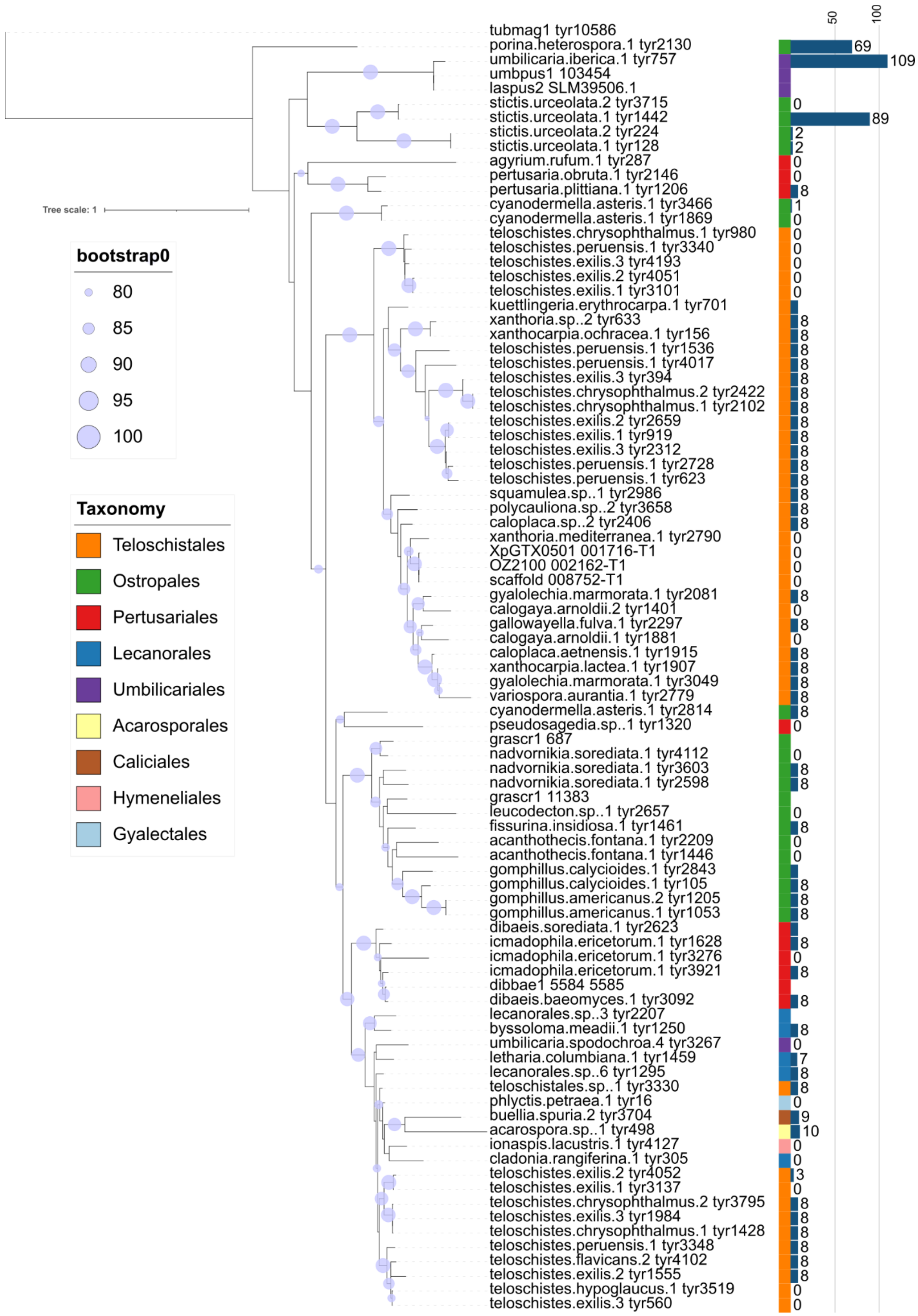

Fig. S14. Magnified clade of the Tangerine-family showing the full set of unclustered captains, annotated based on the taxonomy of their host. The *Tangerine* captain from *X. parietina* is indicated with the arrow. Bootstrap support shown next to the nodes. The bars to the right of the tree show the length of the CB (core-binding) domains for each captain.

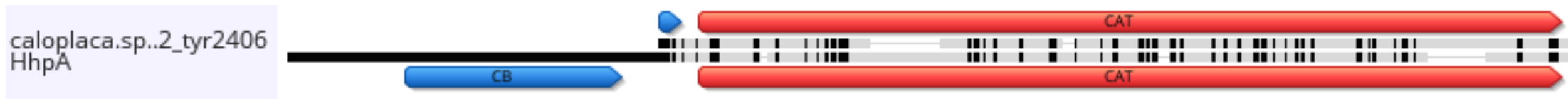

Fig. S15. Sequence alignment of a captain from the Tangerine clade (caloplaca.sp..2 tyr2406) and Hephaestus captain HhpA. The catalytic (CAT) domain is annotated in red. The core-binding (CB) domain of HhpA and the eight amino acid-long putative CB domain in caloplaca.sp..2 tyr2406 are annotated in blue. This putative CB domain does not align to the CB domain from HhpA. The alignment was generated using MUSCLE 5.1 and visualised in Geneious Prime 2025.2.2 (https://www.geneious.com).

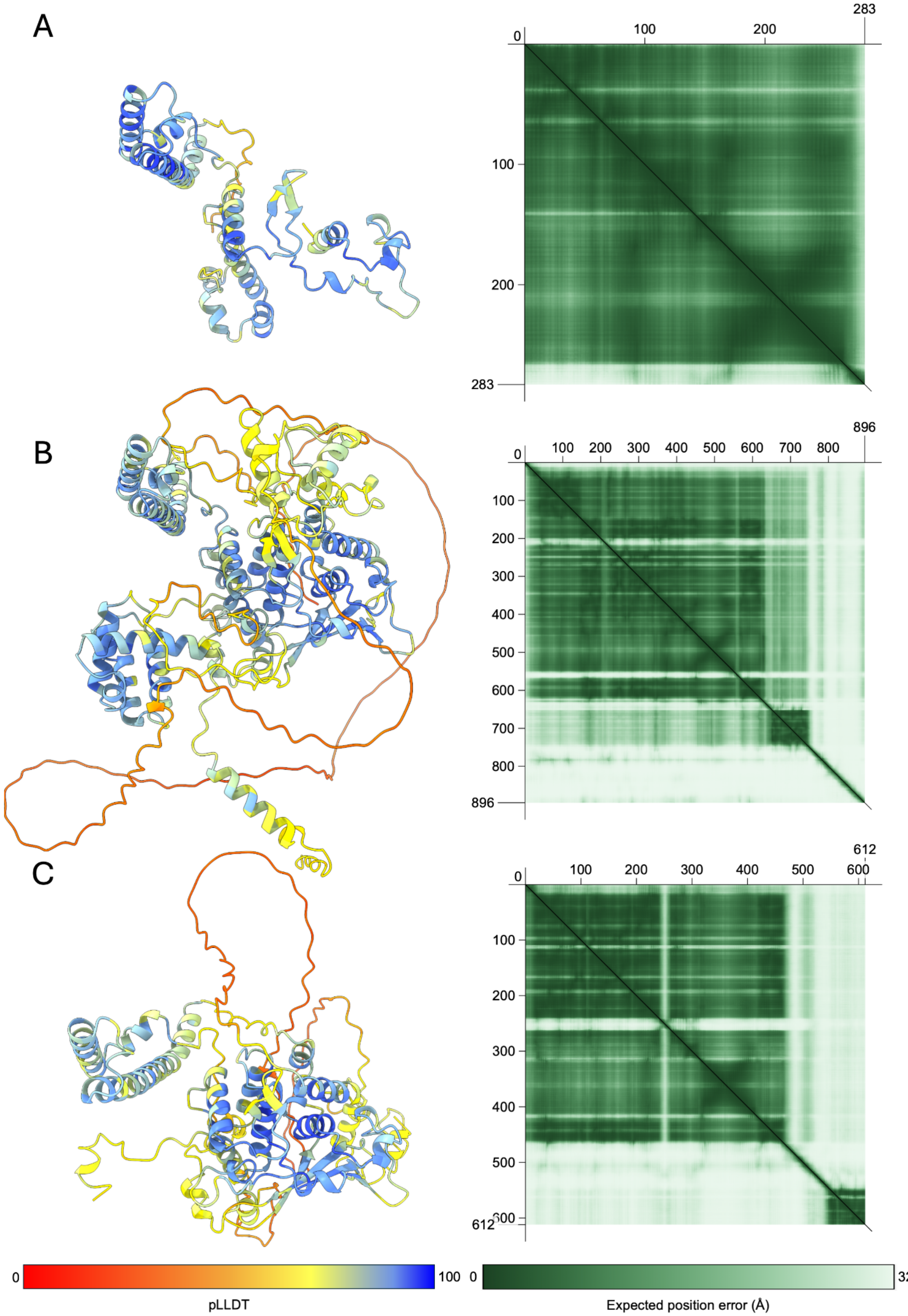

A
B
C
283
0
100
200
283
896
0
100
200
300
400
500
600
700
800
896
612
0
100
200
300
400
500
600
612
0
100
pLLDT
0
32
Expected position error (Å)

Fig. S16. Prediction quality of AlphaFold3-generated monomers. Models coloured by predicted Local Distance Difference Test (pLDDT). Associated predicted aligned error (PAE) plots, measured in Ångström (Å), included to the right of each model. A. *Tangerine* captain XANPAGTX0501_001716-T1 (pTM = 0.69). B. *Hephaestus* captain HhpA (pTM = 0.68). **C.** *Tangerine* captain caloplaca.sp..2_tyr2406 containing a section of the missing Core Binding domain (pTM = 0.68).

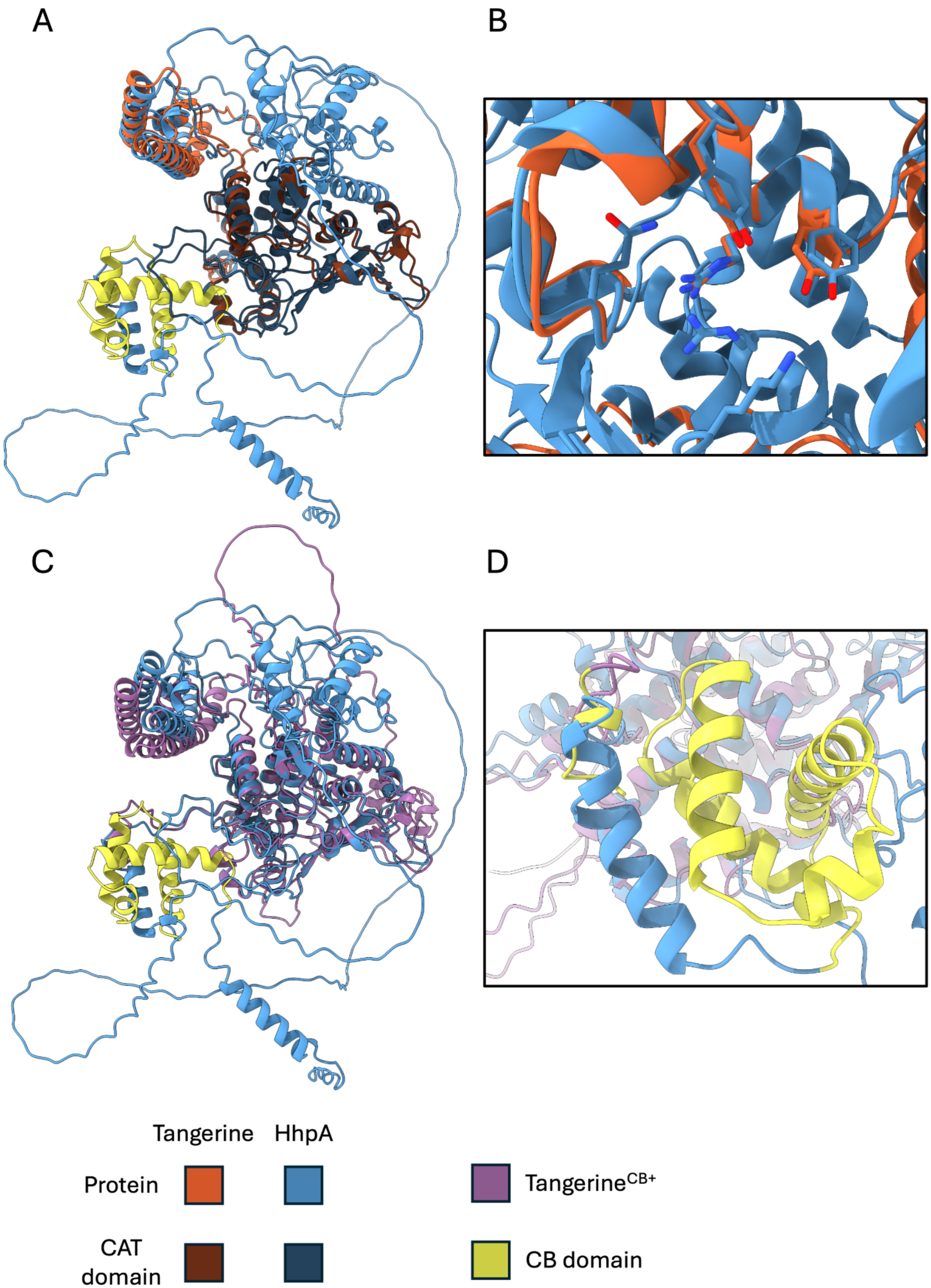
A
B
C
D
Tangerine
HhpA
Protein
CAT domain
Tangerine^CB+
CB domain

Fig. S17. Structural superimposition of AlphaFold3-generated captains. The catalytic (CAT) domains are differentially coloured by model, but where present the core binding (CB) domain coloured yellow. A. *Hephaestus* captain HhpA (blue) superimposed on top of the *Tangerine* captain XANPAGTX0501_001716-T1 (orange). Catalytic (CAT) domains highlighted in dark blue and dark orange respectively. B. Cut-out of the HhpA active site superimposed onto the *Tangerine* captain XANPAGTX0501_001716-T1. C. *Hephaestus* captain HhpA superimposed on top of the *Tangerine* captain caloplaca.sp..2_tyr2406 (purple). D. Cut-out of the HhpA core binding (CB) domain superimposed onto the *Tangerine* captain caloplaca.sp..2_tyr2406 with the 8 residue CB region highlighted.

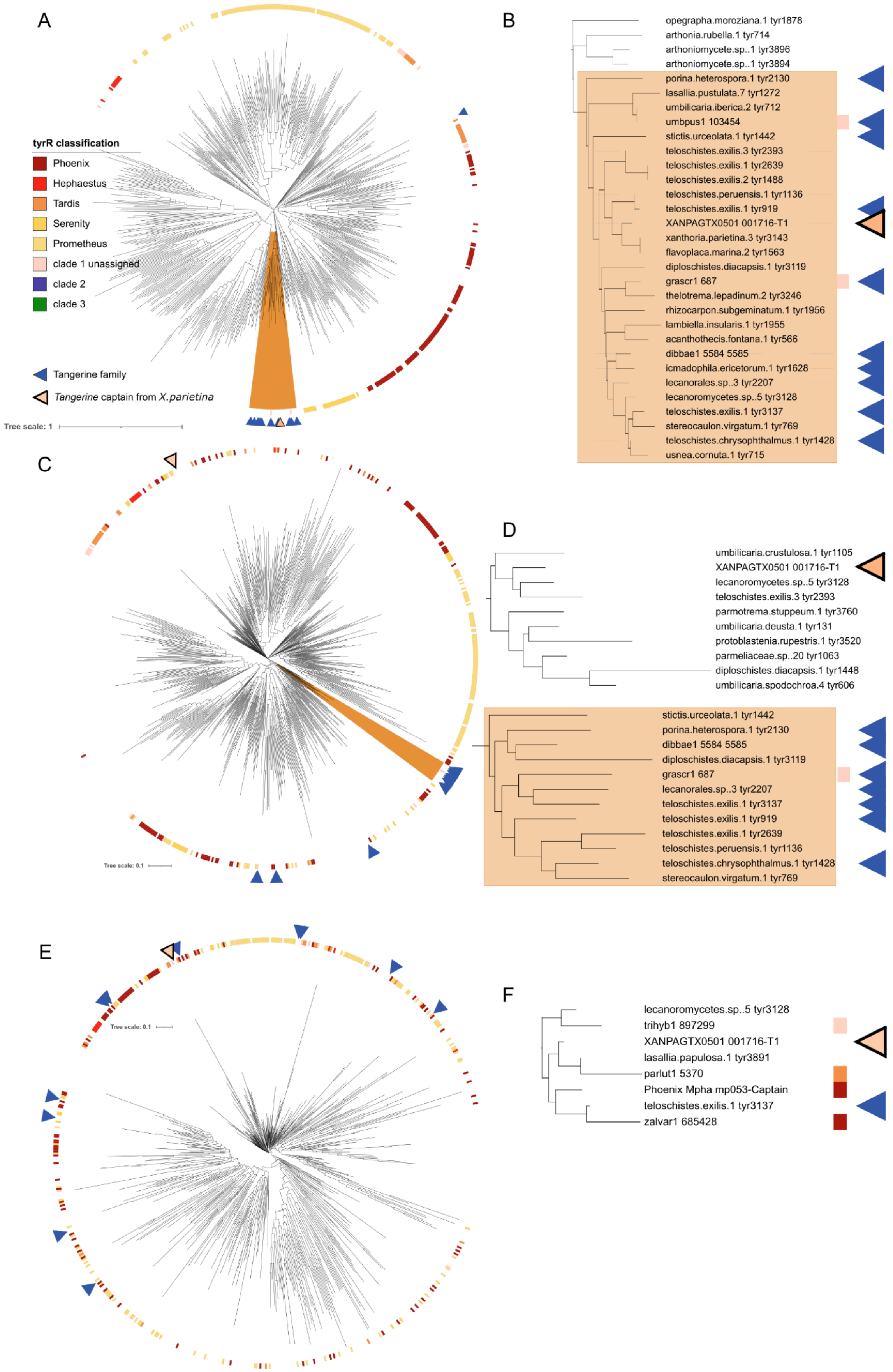
A
B
C
D
E
F
tyrR classification
Phoenix
Hephaestus
Tardis
Serenity
Prometheus
clade 1 unassigned
clade 2
clade 3
Tangerine family
Tangerine captain from X.parietina
Tree scale: 1
opegrapha.moroziana.1 tyr1878
arthonia.rubella.1 tyr714
arthoniomycete.sp..1 tyr3896
arthoniomycete.sp..1 tyr3894
porina.heterospora.1 tyr2130
lasallia.pustulata.7 tyr1272
umbilicaria.iberica.2 tyr712
umbpus1 103454
stictis.urceolata.1 tyr1442
teloschistes.exilis.3 tyr2393
teloschistes.exilis.1 tyr2639
teloschistes.exilis.2 tyr1488
teloschistes.peruensis.1 tyr1136
teloschistes.exilis.1 tyr919
XANPAGTX0501 001716-T1
xanthoria.parietina.3 tyr3143
flavoplaca.marina.2 tyr1563
diploschistes.diacapsis.1 tyr3119
grascr1 687
thelotrema.lepadinum.2 tyr3246
rhizocarpon.subgeminatum.1 tyr1956
lambiella.insularis.1 tyr1955
acanthothecis.fontana.1 tyr566
dibbae1 5584 5585
icmadophila.ericetorum.1 tyr1628
lecanorales.sp..3 tyr2207
lecanoromycetes.sp..5 tyr3128
teloschistes.exilis.1 tyr3137
stereocaulon.virgatum.1 tyr769
teloschistes.chrysophthalmus.1 tyr1428
usnea.cornuta.1 tyr715
Tree scale: 0.1
umbilicaria.crustulosa.1 tyr1105
XANPAGTX0501 001716-T1
lecanoromycetes.sp..5 tyr3128
teloschistes.exilis.3 tyr2393
parmotrema.stuppeum.1 tyr3760
umbilicaria.deusta.1 tyr131
protoblastenia.rupestris.1 tyr3520
parmeliaceae.sp..20 tyr1063
diploschistes.diacapsis.1 tyr1448
umbilicaria.spodochroa.4 tyr606
stictis.urceolata.1 tyr1442
porina.heterospora.1 tyr2130
dibbae1 5584 5585
diploschistes.diacapsis.1 tyr3119
grascr1 687
lecanorales.sp..3 tyr2207
teloschistes.exilis.1 tyr3137
teloschistes.exilis.1 tyr919
teloschistes.exilis.1 tyr2639
teloschistes.peruensis.1 tyr1136
teloschistes.chrysophthalmus.1 tyr1428
stereocaulon.virgatum.1 tyr769
Tree scale: 0.1
lecanoromycetes.sp..5 tyr3128
trihyb1 897299
XANPAGTX0501 001716-T1
lasallia.papulosa.1 tyr3891
parlut1 5370
Phoenix Mpha mp053-Captain
teloschistes.exilis.1 tyr3137
zalvar1 685428

Fig. S18. *Tangerine* captains in the content of the structural phylogeny of tyrosine recombinases (tyrR). We predicted a secondary structure for the *Tangerine* captain (XANPAGTX0501_001716-T1 from *X. parietina* GTX0501), manually selected representative group of *Starship* captains from clade 1 from Gluck-Thaler and Vogan (2024), and a set of *de-novo* annotated representative captains from lichen-associated fungi (Table S18) using AlphaFold, and used FoldTree to reconstruct structure-based phylogeny. The color strip represents the tyrR family according to the sequence-based classification from Vogan & Gluck-Thaler. Blue arrows show Tangerine-family captains, light-orange arrows show the *Tangerine* captain from *X. parietina*. The clades consisting primarily of the Tangerine-family captains are highlighted in orange. The tree files in the Newick format are available at the FigShare repo. A. Full tree based on the Fident similarity metric ('foldtree' phylogeny in the output of FoldTree). B. Tangerine-family clade of the tree based on the Fident similarity metric. C. Full tree based on the local structure alignment (LDDT). D. The clade containing the *Tangerine* captain from *X. parietina* (top) and the majority of the Tangerine-family clade (bottom) of the tree based on LDDT. E. Full tree based on the TM-scores. F. The clade containing the *Tangerine* captain from *X. parietina* of the tree based on the TM-scores.

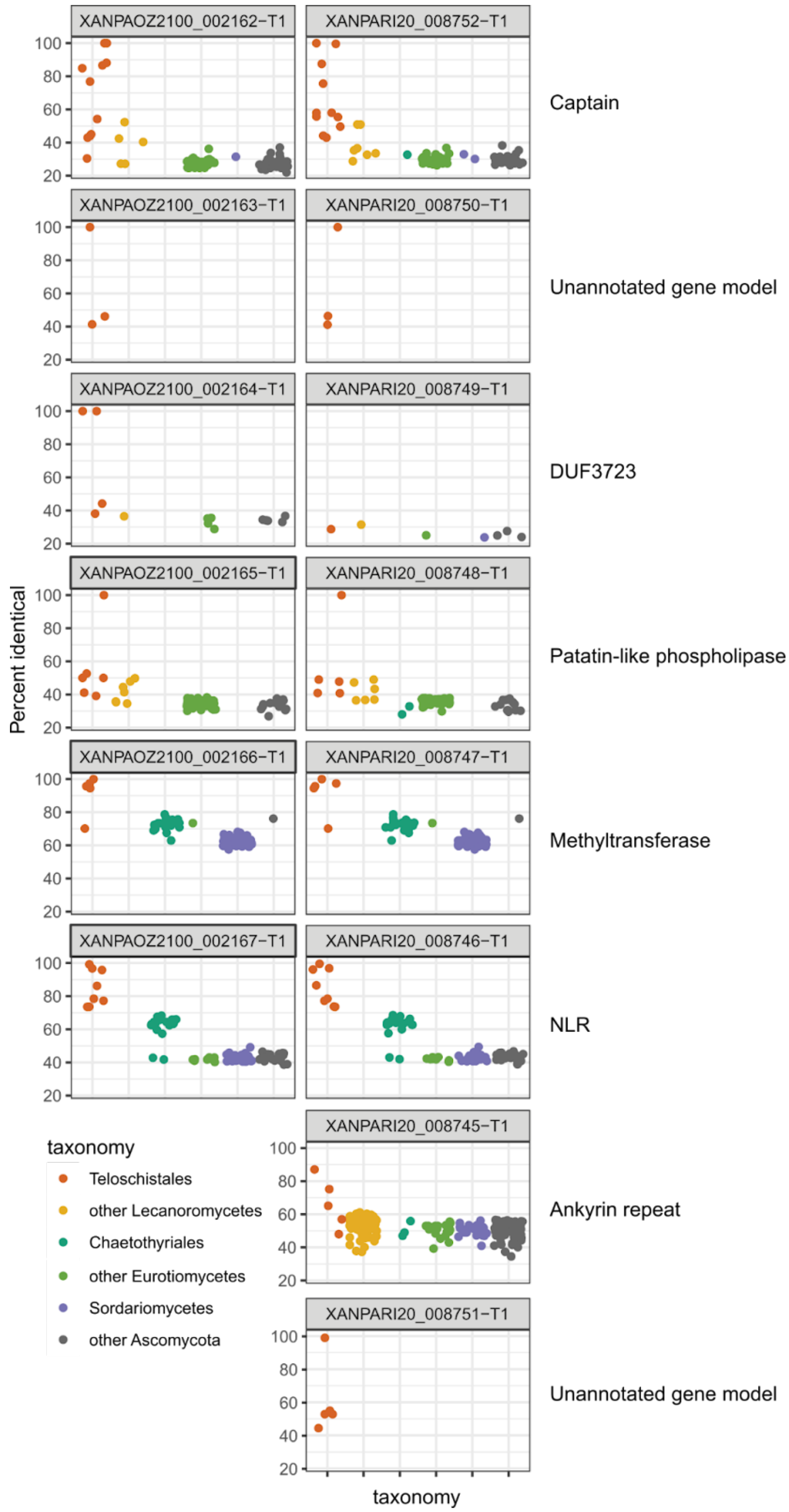

Fig. S19. Hits to the NCBI nr database for *Tangerine*-associate genes. We searched *Tangerine*-associate genes from *X. parietina* glXanPari1 (locus tag XANPAOZ2100) and glXanPari20 (locus tag XANPARI20) against NCBI nr, removed sequences with <50% query cover, and plotted the hits with the y-axis representing percent identity and the x-axis and color representing taxonomic origin of the hit.

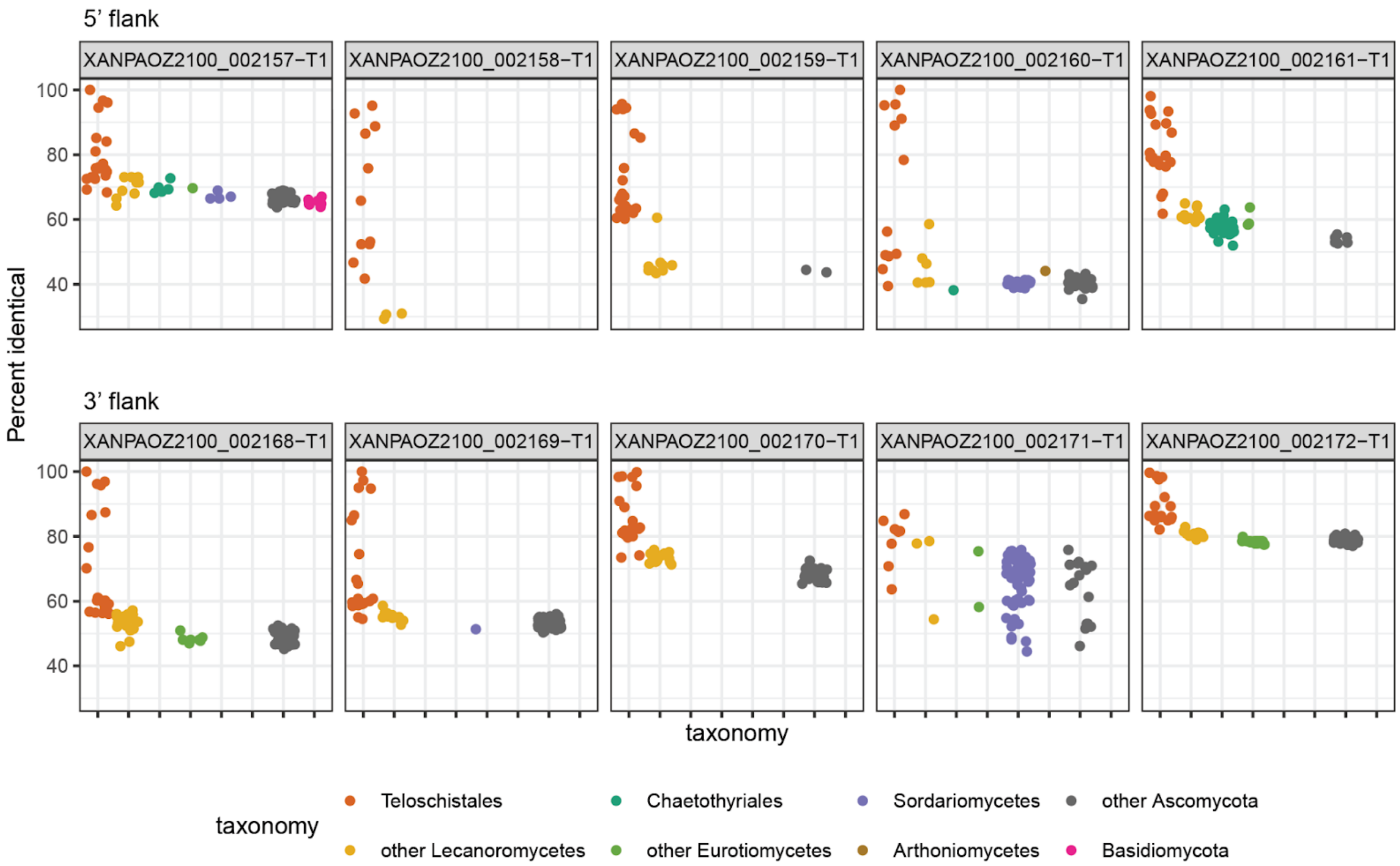

Fig. S20. Hits to the NCBI nr database for genes in the vicinity of *Tangerine*. We selected five gene models on the 5' flank and five gene models on the 3' flank of *Tangerine* in *X. parietina* glXanPari1. We searched the genes against NCBI nr, removed sequences with <50% query cover, and plotted the hits with the y-axis representing percent identity and the x-axis and color representing taxonomic origin of the hit.

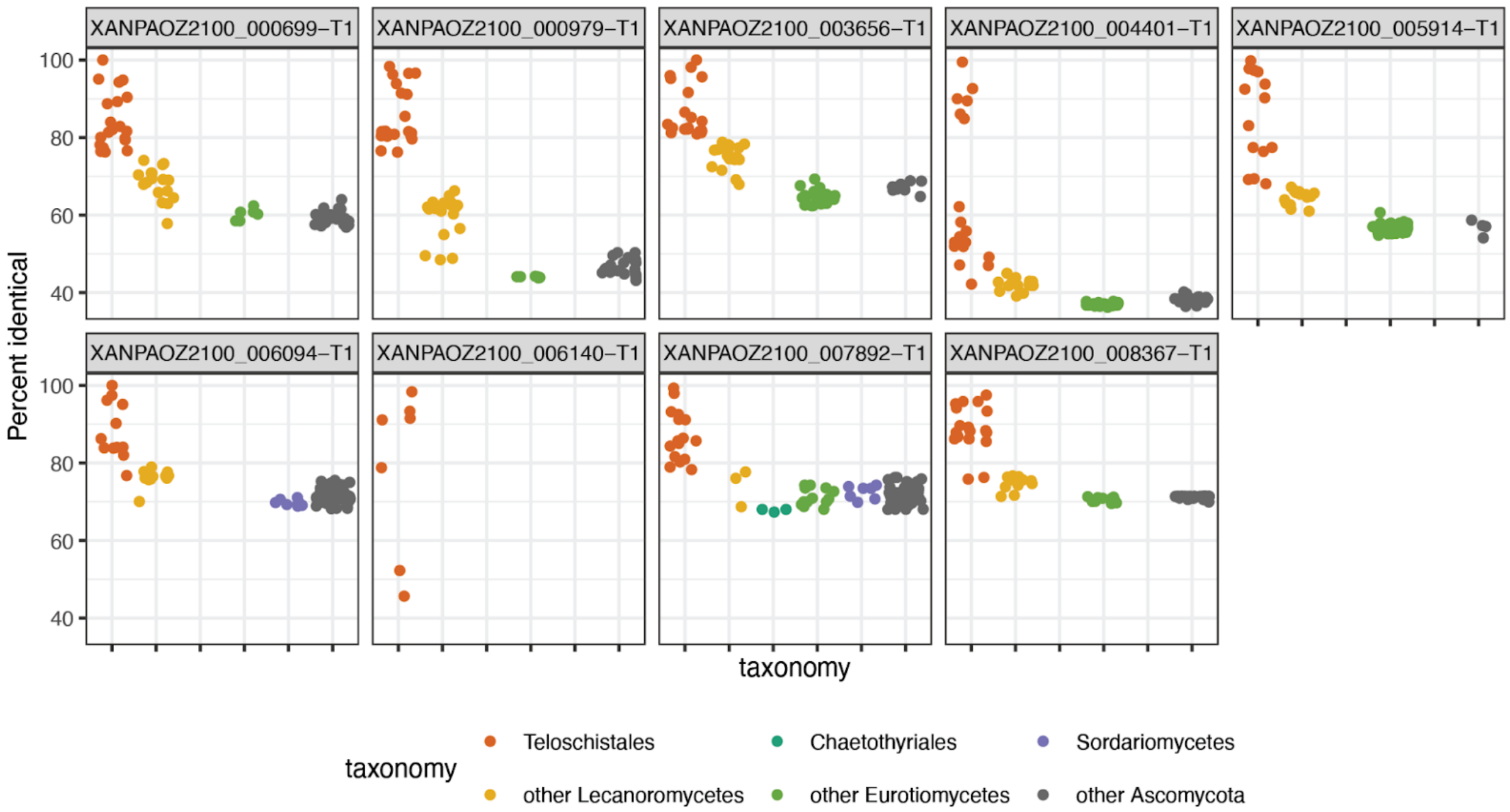

Fig. S21. Hits to the NCBI nr database for randomly selected genes in the *X. parietina* glXanPari1 genome. We randomly selected 10 gene models in *X. parietina* glXanPari1 and searched them against NCBI nr, removed sequences with <50% query cover, and plotted the hits with the y-axis representing percent identity and the x-axis and color representing taxonomic origin of the hit. One of the genes, XANPAOZ2100_002117-T1, did not produce any hits above the query cover threshold.

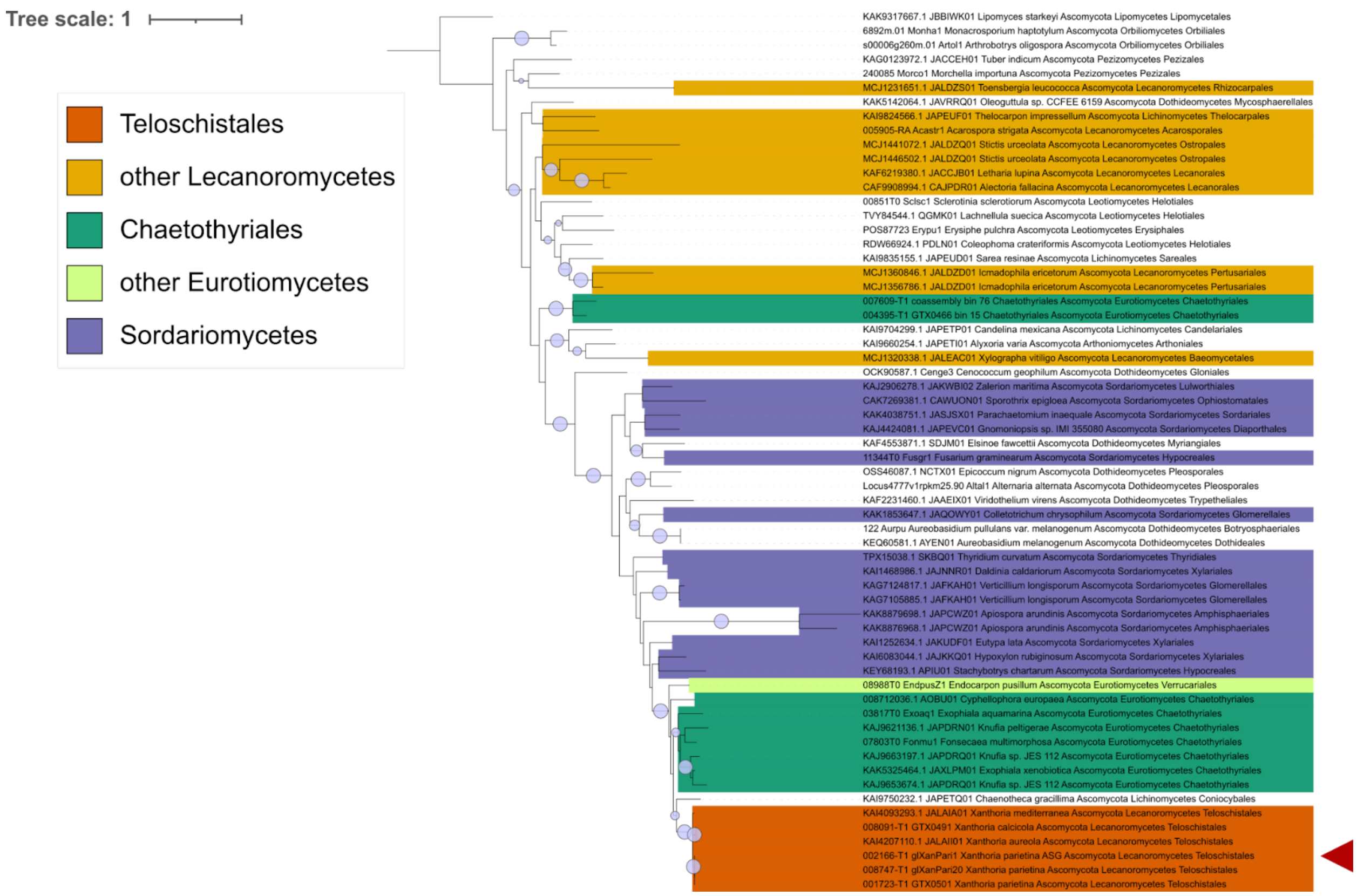

Fig. S22. Gene tree for the orthogroup OG0006598 containing the methyltransferase. To obtain the tree, we performed orthogroup analysis on 108 ascomycete genomes. Using the sequences for the identified orthogroup, we computed maximum-likelihood phylogeny. The tree is colored based on the taxonomic origin of sequences; bootstrap values are shown for the clades with >90 support. The sequences from *X. parietina* glXanPari1 are indicated with arrows.

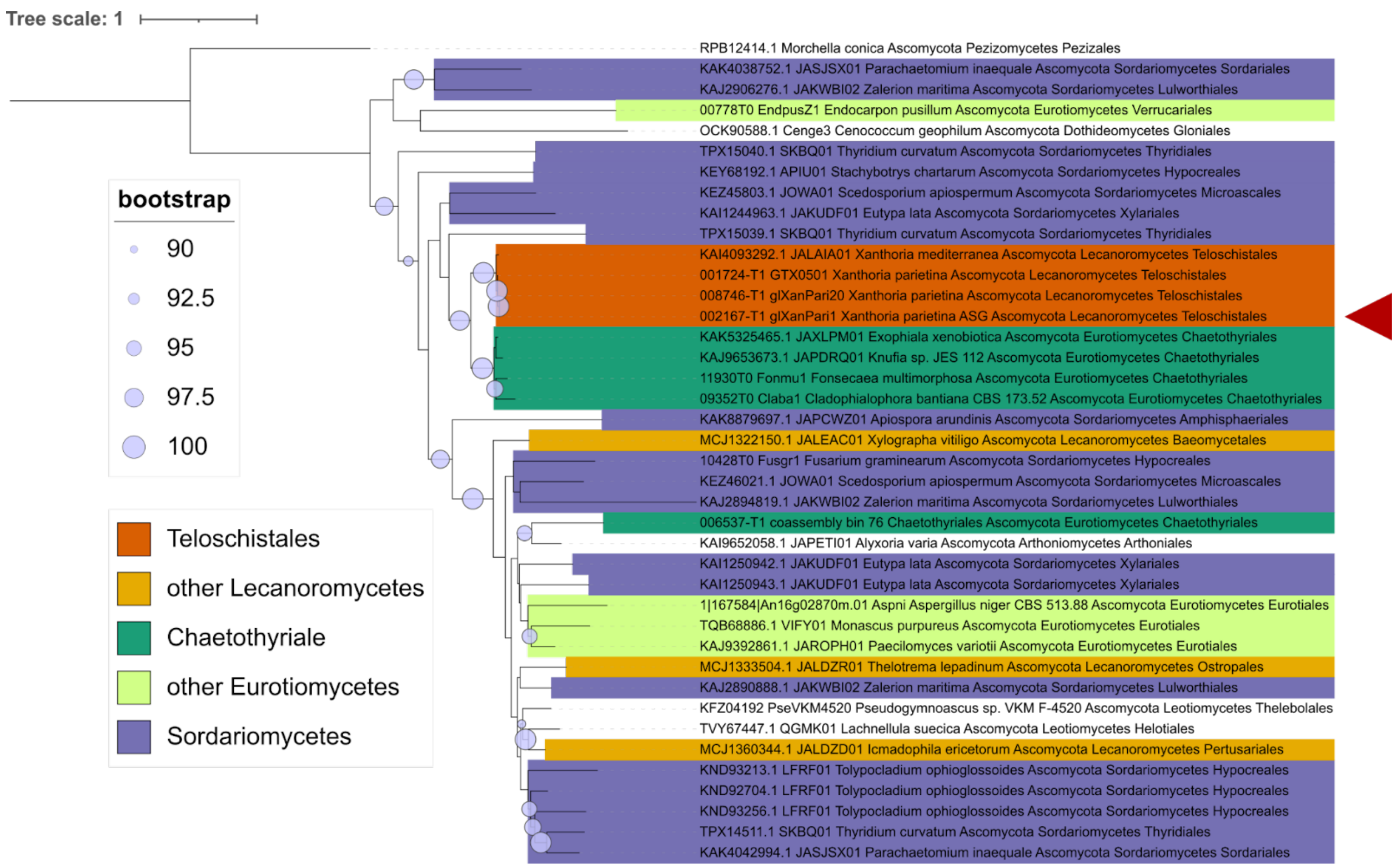

Fig. S23. Gene tree for the orthogroup OG0007437 containing the NLR gene. To obtain the tree, we performed orthogroup analysis on 108 ascomycete genomes. Using the sequences for the identified orthogroup, we computed maximum-likelihood phylogeny. The tree is colored based on the taxonomic origin of sequences; bootstrap values are shown for the clades with >90 support. The sequences from *X. parietina* glXanPari1 are indicated with arrows.

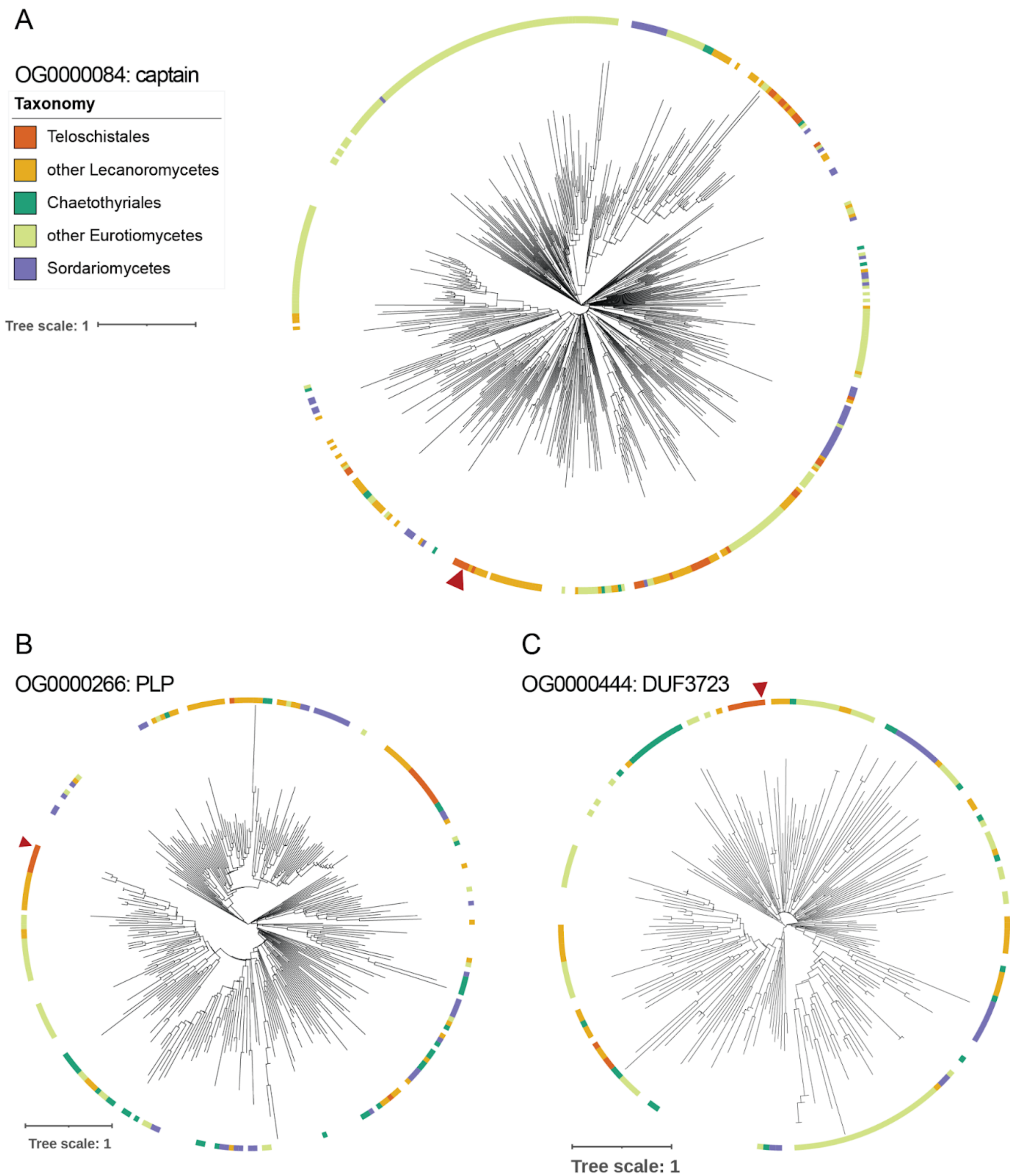

Fig. S24. Gene trees for orthogroups containing *Tangerine*-associated genes produced by OrthoFinder. A. Tree for OG0000084. The *Tangerine* captain (XANPAOZ2100_002162-T1) is indicated with the arrow. B. Tree for OG0000266. The *Tangerine* patatin-like phospholipase (PLP; XANPAOZ2100_002165-T1) is indicated with the arrow. C. Tree for OG0000444. The *Tangerine* DUF3723 (XANPAOZ2100_002164-T1) is indicated with the arrow. The outer ring indicates the taxonomic origin of the sequence. For DUF3723, the Teloschistales clade continuing the gene of interest is sister to a Lecanoromycete gene. For the remaining two genes, the Teloschistales clade continuing the gene of interest is nested within a larger Lecanoromycete clade.

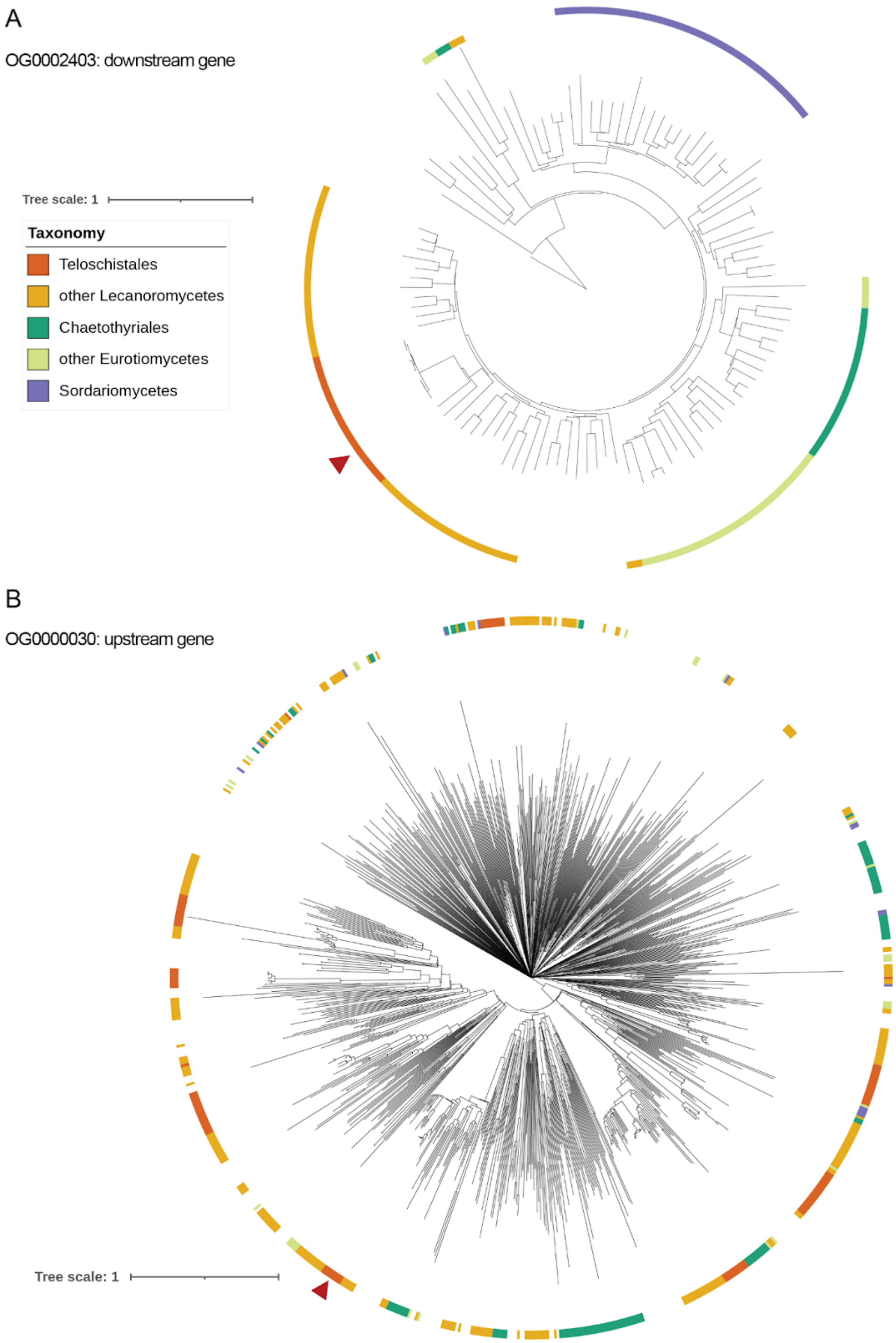

Fig. S25. Gene trees for orthogroups containing the genes flanking *Tangerine* in *Xanthoria parietina* isolate glXanPari1. A. Tree for OG0002403. The gene on the 3' flank of *Tangerine* (XANPAOZ2100_002168-T1) is indicated with the arrow. B. Tree for OG0000030. The gene on the 5' flank of *Tangerine* (XANPAOZ2100_002161-T1) is indicated with the arrow. The outer ring indicates the taxonomic origin of the sequence. For both genes, the Teloschistales clade continuing the gene of interest is nested within a larger Lecanoromycete clade.

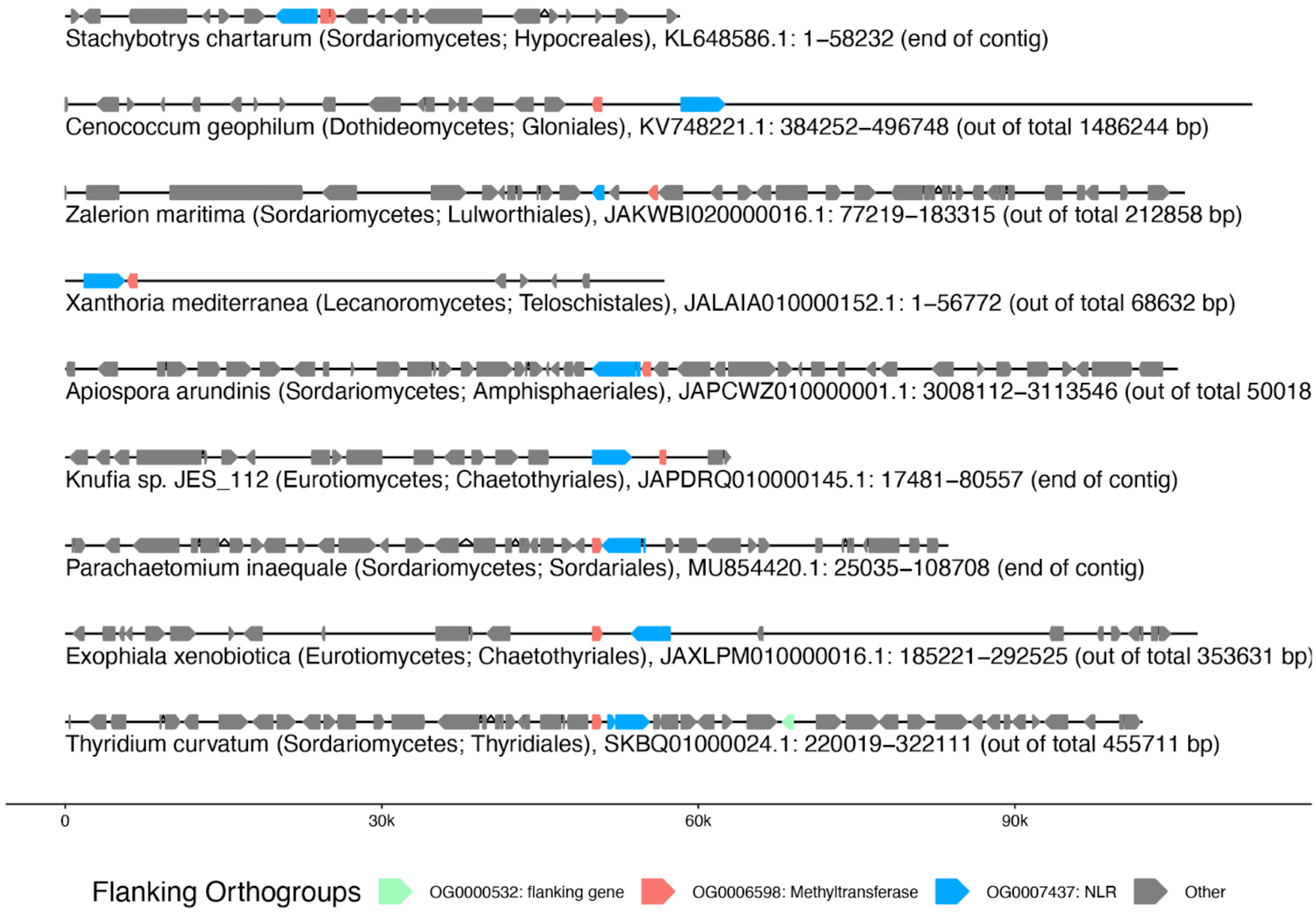

Fig. S26. Fragments of genomes containing genes assigned to the orthogroups OG0006598 (methyltransferase) and OG0007437 (NLR) and 50 Kbp flanks. Among the genomes included in the orthogroup analysis (Table S14), we selected genomes in which the genes assigned to the two orthogroups are placed in the same contig and separated by no more than one gene model. We plotted the contig fragments with all gene models shown as arrows. The genes assigned to orthogroups OG0006598 and OG0007437 are highlighted.

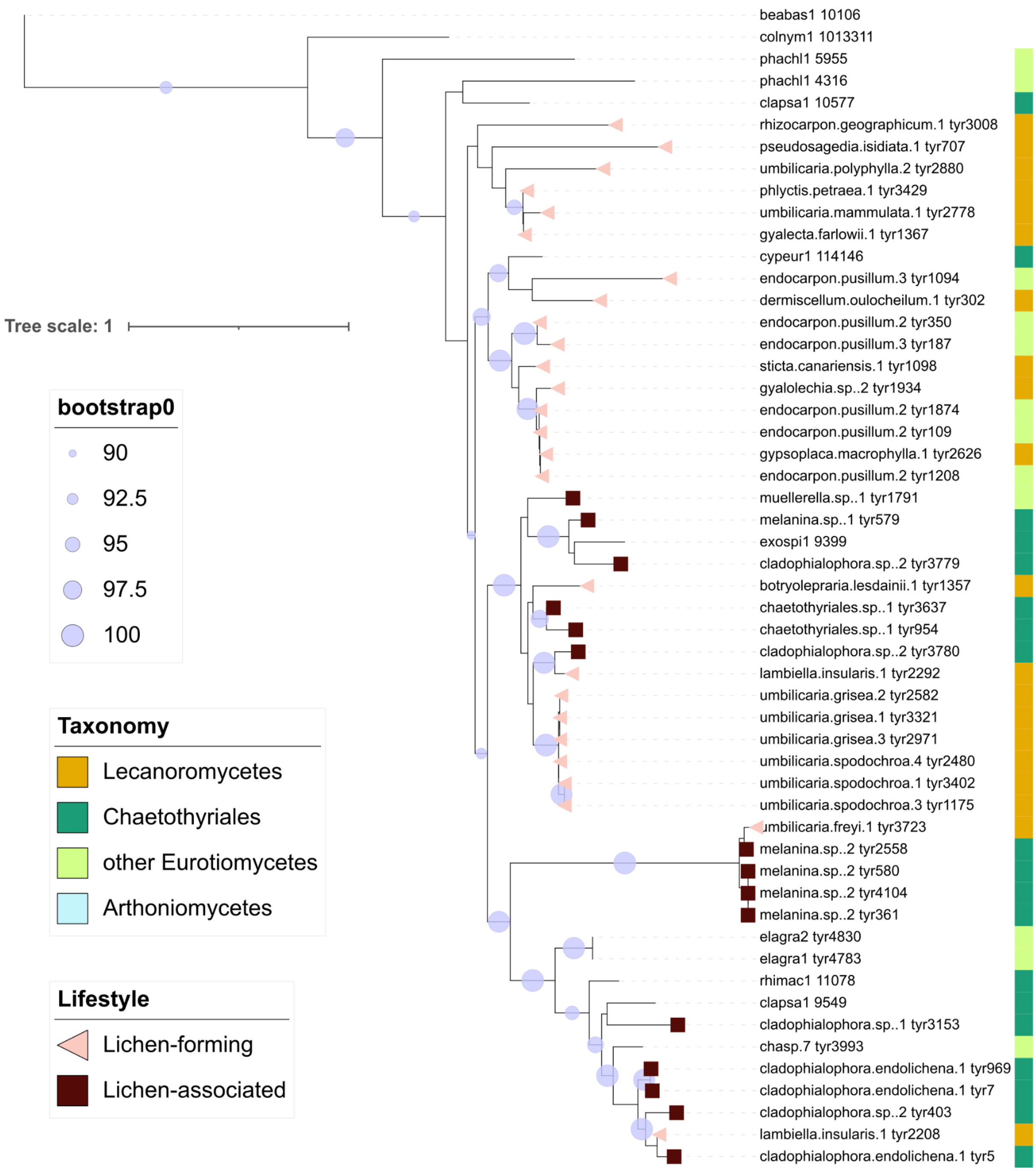

Fig. S27. Clade A in the *Starship* tyrR phylogeny, which shows signatures of horizontal gene transfer (HGT) between distantly related lichen symbionts. Captains within clades in the larger representative captain phylogenetic tree that showed signatures of HGT in lichen fungi were unclustered and then re-aligned; the alignment was used to recreate maximum-likelihood phylogeny. Bootstrap values are shown for the clades with >90 support. The symbols at the ends of branches indicate lifestyle: tyrRs from lichen-forming fungi (also known as mycobionts) are shown in pink triangles, tyrRs from non mycobiont lichen-associated (also known as lichenicolous or endolichenic) fungi are shown as brown squares. Sequences from non-lichen fungi lack annotation. The color strip shows the taxonomic group of the fungus. Information on the sequences is shown in Table S15.

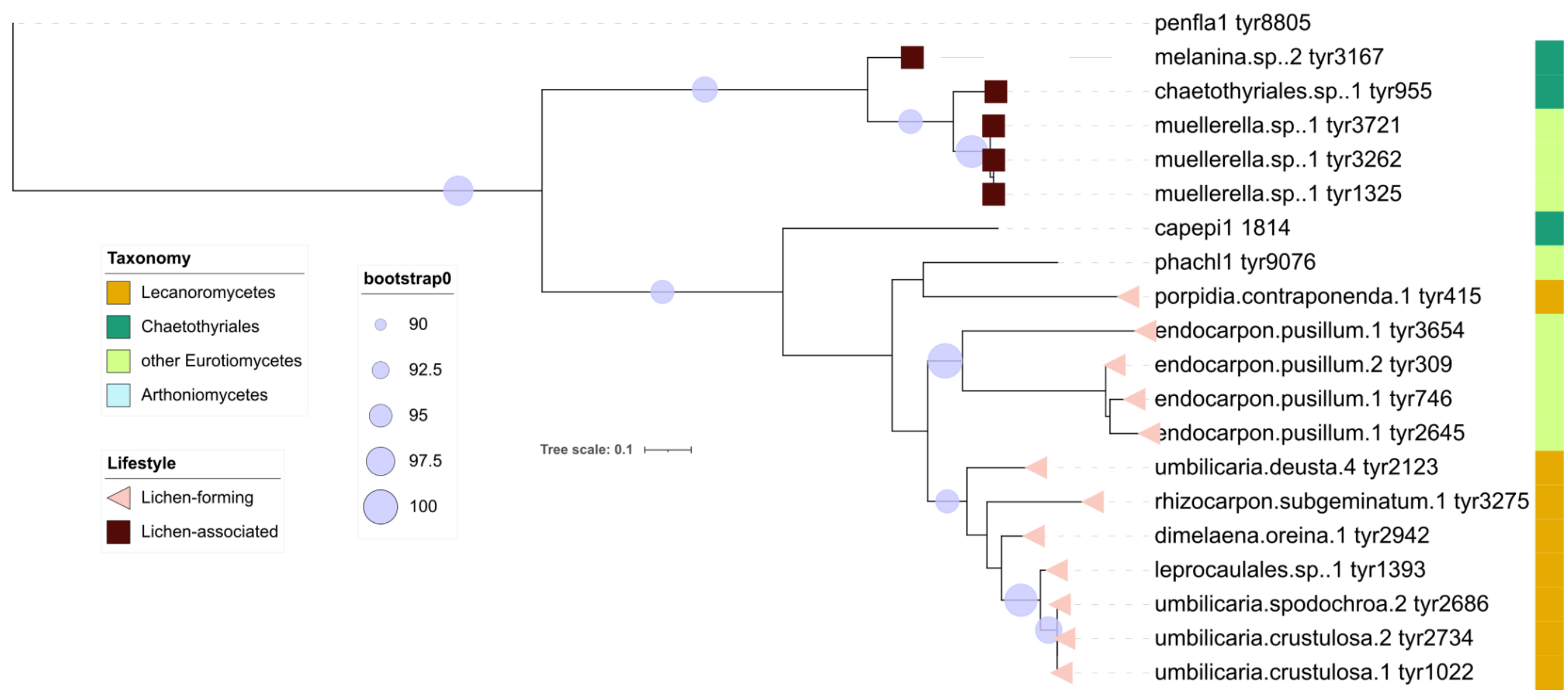

Fig. S28. Clade B in the *Starship* tyrR phylogeny, which shows signatures of horizontal gene transfer (HGT) between distantly related lichen symbionts. Captains within clades in the larger representative captain phylogenetic tree that showed signatures of HGT in lichen fungi were unclustered and then re-aligned; the alignment was used to recreate maximum-likelihood phylogeny. Bootstrap values are shown for the clades with >90 support. The symbols at the ends of branches indicate lifestyle: tyrRs from lichen-forming fungi (also known as mycobionts) are shown in pink triangles, tyrRs from non mycobiont lichen-associated (also known as lichenicolous or endolichenic) fungi are shown as brown squares. Sequences from non-lichen fungi lack annotation. The color strip shows the taxonomic group of the fungus. Information on the sequences is shown in Table S15.

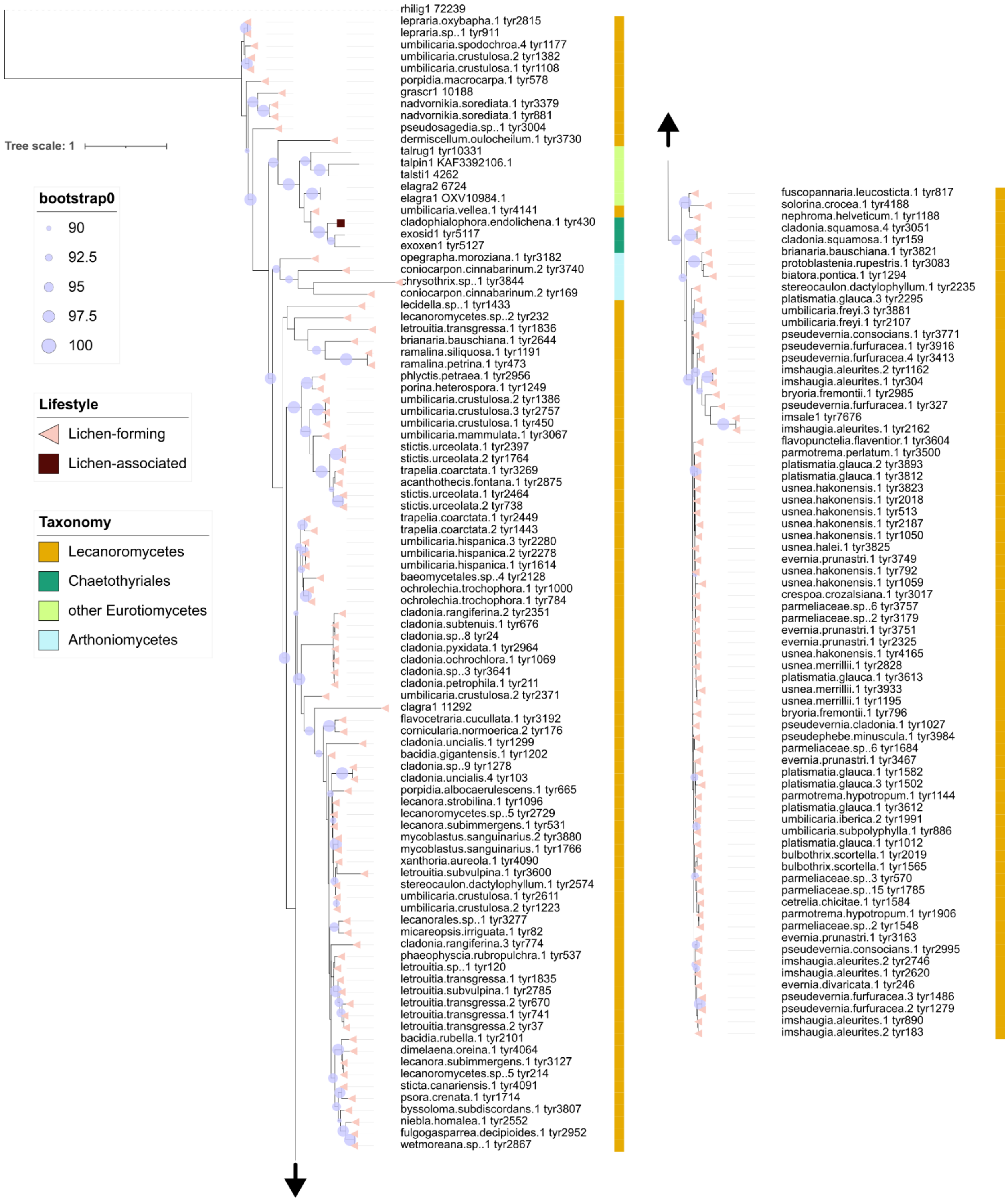

Fig. S29. Clade C in the *Starship* tyrR phylogeny, which shows signatures of horizontal gene transfer (HGT) between distantly related lichen symbionts. Captains within clades in the larger representative captain phylogenetic tree that showed signatures of HGT in lichen fungi were unclustered and then re-aligned; the alignment was used to recreate maximum-likelihood phylogeny. Bootstrap values are shown for the clades with >90 support. The symbols at the ends of branches indicate lifestyle: tyrRs from lichen-forming fungi (also known as mycobionts) are

shown in pink triangles, tyrRs from non mycobiont lichen-associated (also known as lichenicolous or endolichenic) fungi are shown as brown squares. Sequences from non-lichen fungi lack annotation. The color strip shows the taxonomic group of the fungus. Information on the sequences is shown in Table S15.

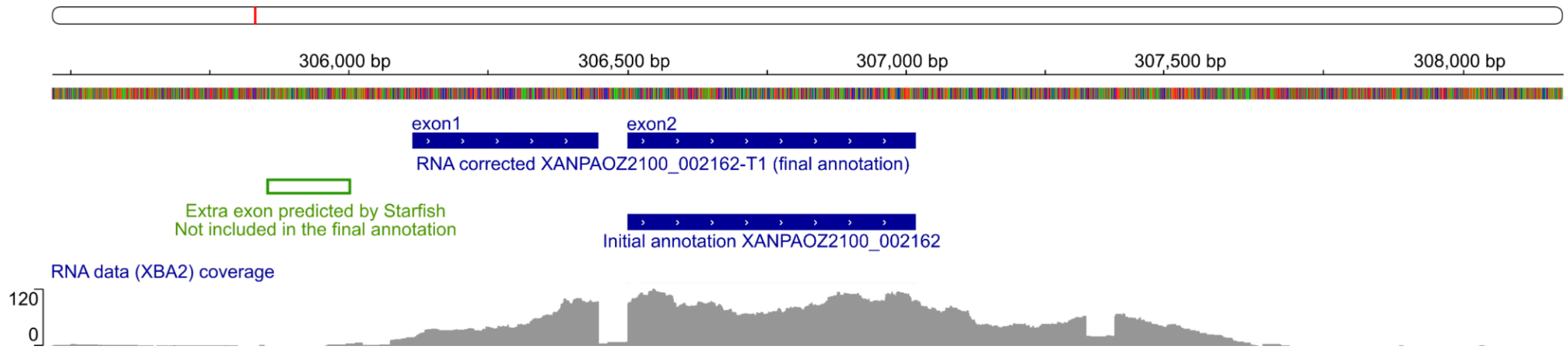

Fig. S30. Annotation of the *Tangerine* captain XANPAOZ2100_2162 from glXanPari1 genome juxtaposed with RNA data. Metatranscriptomic data from a *X. parietina* thallus (library XBA2) was mapped to the genome assembly using STAR. Initial annotation of the XANPAOZ2100_2162 gene model was produced by Funannotate and subsequently corrected based on the RNA mapping. When we ran the Starfish gene prediction module, a different model for this gene was produced. This model included an additional exon on the N-terminus, here shown in green. As the existence of this exon was not supported by the RNA data, it was included in the final gene model used in the downstream analyses. The data was visualized using IGV web interface.

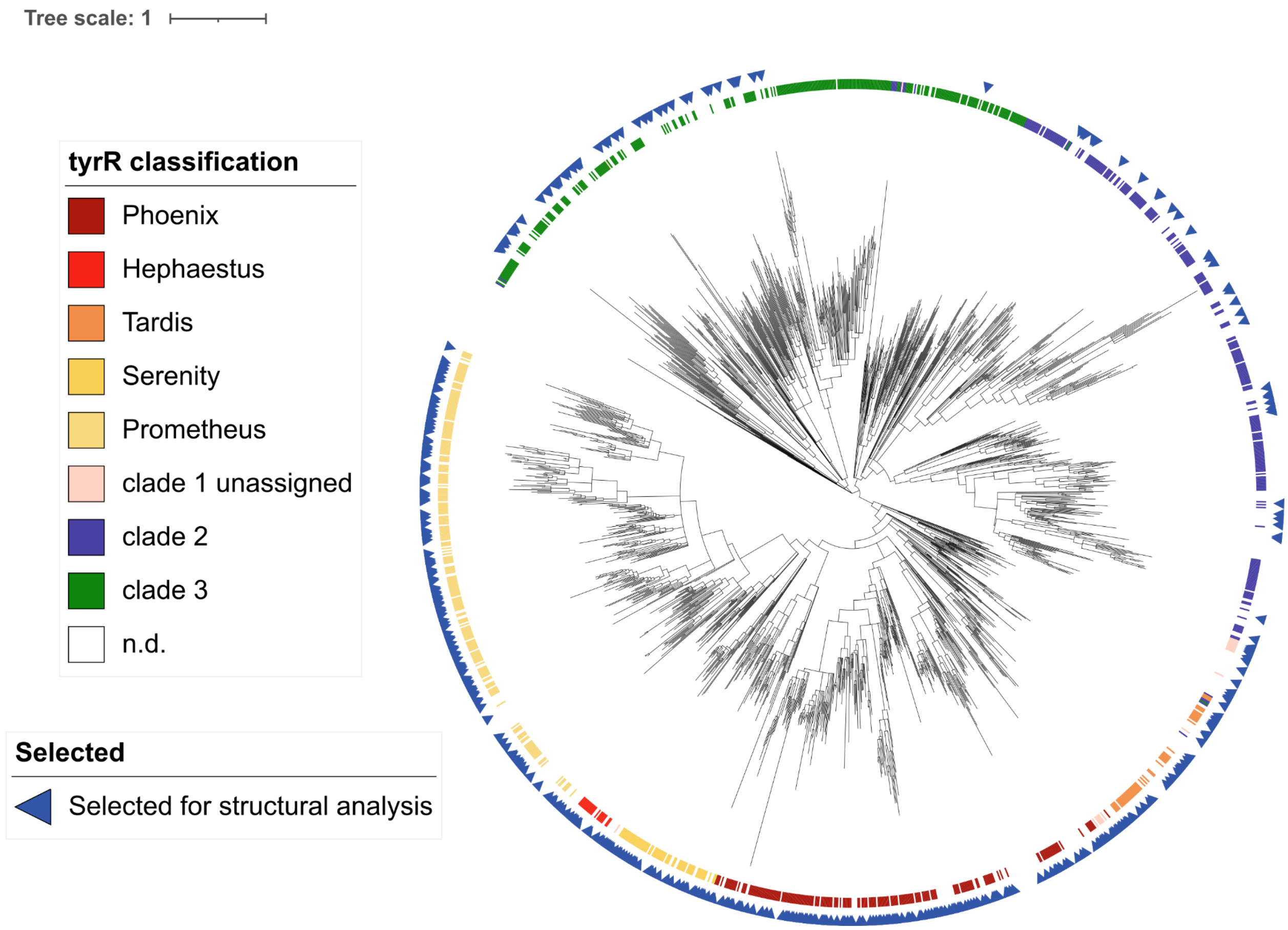

Fig. S31. Proteins selected for structural analysis. The sequence alignment-based tree (Fig. 3A) is shown with a color track showing family assignments for the reference tyrRs. Blue arrows show captains from lichen-associated fungi that were selected for AlphaFold structural modelling.

Tables

Table S1. Statistics for the four long-read genomes of Xanthoria included in this study.

Genome	Source	Data type	Genome size, bp	Number of gene models	Number of scaffolds	N50 of scaffolds, Mbp	Repeat content, %	GC, %
Xanthoria parietina isolate glXanPari1	PRJEB83421	PacBio + HiC	30389298	9049	17	2.048	13.72	50.55
Xanthoria parietina isolate glXanPari20	PRJEB63593	PacBio + HiC	30164939	9230	15	3.027	13.46	50.61
Xanthoria parietina isolate GTX0501	PRJEB78723	Nanopore	29958160	10743	58	1.589	12.87	50.81
Xanthoria calcicola isolate GTX0491	This paper	Nanopore	36282728	10027	16	2.319	24.45	45.59

Table S2. Repeat family-level summaries for the four Xanthoria genomes generated with Earl Grey.

TE Family	Coverage, bp.	Copy Number	Genome	type
RND-1_FAMILY-1#LINE/Tad1	1013664	378	X.parietina glXanPari20	LINE/Tad1
RND-1_FAMILY-43#LINE/Tad1	332630	142	X.parietina glXanPari20	LINE/Tad1
RND-1_FAMILY-75#LINE/Tad1	285193	190	X.parietina glXanPari20	LINE/Tad1
RND-2_FAMILY-14#LTR/Ty3	142192	62	X.parietina glXanPari20	LTR/Ty3
RND-1_FAMILY-2#LINE/Tad1	119116	87	X.parietina glXanPari20	LINE/Tad1
RND-2_FAMILY-12#LTR/Ty3	116965	46	X.parietina glXanPari20	LTR/Ty3
RND-3_FAMILY-123#LTR/Ty3	115143	63	X.parietina glXanPari20	LTR/Ty3
RND-1_FAMILY-53#LTR/Ty3	114988	56	X.parietina glXanPari20	LTR/Ty3
RND-1_FAMILY-20#LTR/Copia	92228	43	X.parietina glXanPari20	LTR/Copia
RND-1_FAMILY-47#LTR/Copia	86621	24	X.parietina glXanPari20	LTR/Copia
RND-1_FAMILY-60#LTR/Ty3	77883	21	X.parietina glXanPari20	LTR/Ty3
RND-1_FAMILY-57#LTR/Ty3	76499	22	X.parietina glXanPari20	LTR/Ty3
RND-1_FAMILY-52#LTR/Copia	73664	34	X.parietina glXanPari20	LTR/Copia
RND-3_FAMILY-100#LTR/Copia	67763	26	X.parietina glXanPari20	LTR/Copia
RND-1_FAMILY-27#LTR/Ty3	66495	29	X.parietina glXanPari20	LTR/Ty3
RND-1_FAMILY-33#LTR/Copia	64179	34	X.parietina glXanPari20	LTR/Copia
RND-1_FAMILY-51#LTR/Ty3	55368	20	X.parietina glXanPari20	LTR/Ty3
RND-1_FAMILY-24#LTR/Ty3	51480	18	X.parietina glXanPari20	LTR/Ty3
RND-1_FAMILY-31#LTR/Copia	49743	34	X.parietina glXanPari20	LTR/Copia
RND-1_FAMILY-54#LTR/ERV1	45903	12	X.parietina glXanPari20	LTR/ERV1
RND-1_FAMILY-71#DNA/Kolobok-H	45764	24	X.parietina glXanPari20	DNA/Kolobok-H
RND-1_FAMILY-50#LTR/Ty3	43889	14	X.parietina glXanPari20	LTR/Ty3
RND-2_FAMILY-51#Unknown	42734	81	X.parietina glXanPari20	Unknown

RND-1_FAMILY-49#LTR/Ty3	36777	11	X.parietina glXanPari20	LTR/Ty3
RND-1_FAMILY-7#LTR/Ty3	35391	17	X.parietina glXanPari20	LTR/Ty3
RND-1_FAMILY-37#LTR/Copia	32252	25	X.parietina glXanPari20	LTR/Copia
RND-1_FAMILY-59#LINE/Tad1	31992	13	X.parietina glXanPari20	LINE/Tad1
RND-1_FAMILY-102#LTR/Ty3	31719	13	X.parietina glXanPari20	LTR/Ty3
RND-1_FAMILY-98#LTR/Ty3	27977	25	X.parietina glXanPari20	LTR/Ty3
RND-1_FAMILY-32#Unknown	24836	153	X.parietina glXanPari20	Unknown
RND-1_FAMILY-46#DNA/MULE-MuDR	24799	19	X.parietina glXanPari20	DNA/MULE-MuDR
RND-1_FAMILY-70#LTR/Copia	21602	20	X.parietina glXanPari20	LTR/Copia
RND-1_FAMILY-34#LINE/Tad1	20013	8	X.parietina glXanPari20	LINE/Tad1
GA-RICH#Low_complexity	18253	330	X.parietina glXanPari20	Low_complexity
RND-1_FAMILY-18#Unknown	17796	38	X.parietina glXanPari20	Unknown
G-RICH#Low_complexity	16911	284	X.parietina glXanPari20	Low_complexity
RND-1_FAMILY-68#LTR/Ty3	14286	18	X.parietina glXanPari20	LTR/Ty3
RND-2_FAMILY-40#Unknown	13911	90	X.parietina glXanPari20	Unknown
RND-1_FAMILY-92#LTR/Ty3	13627	16	X.parietina glXanPari20	LTR/Ty3
RND-3_FAMILY-20#Unknown	13457	140	X.parietina glXanPari20	Unknown
RND-1_FAMILY-82#LINE/Tad1	13370	8	X.parietina glXanPari20	LINE/Tad1
RND-1_FAMILY-38#Unknown	13138	11	X.parietina glXanPari20	Unknown
RND-1_FAMILY-30#Satellite	12799	20	X.parietina glXanPari20	Satellite
RND-1_FAMILY-3#Unknown	12028	76	X.parietina glXanPari20	Unknown
RND-1_FAMILY-63#Unknown	10936	29	X.parietina glXanPari20	Unknown
RND-3_FAMILY-896#Unknown	9934	70	X.parietina glXanPari20	Unknown
RND-1_FAMILY-35#LTR/Ty3	9441	7	X.parietina glXanPari20	LTR/Ty3
RND-1_FAMILY-100#Unknown	9365	13	X.parietina glXanPari20	Unknown
RND-3_FAMILY-132#Unknown	8928	29	X.parietina glXanPari20	Unknown

RND-1_FAMILY-91#Unknown	7527	24	X.parietina glXanPari20	Unknown
RND-1_FAMILY-15#LINE/Tad1	7506	7	X.parietina glXanPari20	LINE/Tad1
RND-1_FAMILY-97#LTR/Ty3	7270	6	X.parietina glXanPari20	LTR/Ty3
RND-3_FAMILY-197#Unknown	6876	122	X.parietina glXanPari20	Unknown
RND-1_FAMILY-78#LTR/Copia	6754	12	X.parietina glXanPari20	LTR/Copia
RND-1_FAMILY-80#LTR/Ty3	6508	7	X.parietina glXanPari20	LTR/Ty3
RND-1_FAMILY-36#DNA	6216	3	X.parietina glXanPari20	DNA
RND-1_FAMILY-84#LTR/Ty3	5880	7	X.parietina glXanPari20	LTR/Ty3
RND-1_FAMILY-61#Unknown	5649	86	X.parietina glXanPari20	Unknown
RND-1_FAMILY-66#Unknown	5492	10	X.parietina glXanPari20	Unknown
RND-1_FAMILY-21#Unknown	5199	11	X.parietina glXanPari20	Unknown
RND-1_FAMILY-42#Unknown	5195	6	X.parietina glXanPari20	Unknown
RND-1_FAMILY-6#Unknown	5181	22	X.parietina glXanPari20	Unknown
RND-1_FAMILY-86#Unknown	5170	16	X.parietina glXanPari20	Unknown
RND-1_FAMILY-65#DNA/MULE-MuDR	4880	5	X.parietina glXanPari20	DNA/MULE-MuDR
RND-1_FAMILY-96#Unknown	4784	61	X.parietina glXanPari20	Unknown
RND-1_FAMILY-45#Unknown	4691	36	X.parietina glXanPari20	Unknown
A-RICH#Low_complexity	4555	95	X.parietina glXanPari20	Low_complexity
RND-1_FAMILY-69#Unknown	4523	7	X.parietina glXanPari20	Unknown
RND-1_FAMILY-67#Unknown	4136	17	X.parietina glXanPari20	Unknown
RND-1_FAMILY-89#Unknown	3980	8	X.parietina glXanPari20	Unknown
RND-1_FAMILY-19#Unknown	3755	8	X.parietina glXanPari20	Unknown
RND-1_FAMILY-41#Unknown	3703	23	X.parietina glXanPari20	Unknown
(CCT)N#Simple_repeat	3695	82	X.parietina glXanPari20	Simple_repeat
RND-1_FAMILY-81#Unknown	3658	10	X.parietina glXanPari20	Unknown
RND-1_FAMILY-8#Unknown	3630	14	X.parietina glXanPari20	Unknown

RND-1_FAMILY-28#Unknown	3617	9	X.parietina glXanPari20	Unknown
RND-3_FAMILY-228#Unknown	3552	44	X.parietina glXanPari20	Unknown
RND-1_FAMILY-94#Unknown	3409	14	X.parietina glXanPari20	Unknown
RND-1_FAMILY-9#Unknown	3248	9	X.parietina glXanPari20	Unknown
RND-1_FAMILY-11#Unknown	2904	13	X.parietina glXanPari20	Unknown
RND-1_FAMILY-10#Unknown	2875	36	X.parietina glXanPari20	Unknown
(C)N#Simple_repeat	2848	110	X.parietina glXanPari20	Simple_repeat
RND-1_FAMILY-29#Unknown	2843	36	X.parietina glXanPari20	Unknown
RND-1_FAMILY-101#DNA	2821	1	X.parietina glXanPari20	DNA
RND-1_FAMILY-58#Unknown	2751	11	X.parietina glXanPari20	Unknown
RND-1_FAMILY-76#Unknown	2747	36	X.parietina glXanPari20	Unknown
RND-1_FAMILY-40#Unknown	2705	17	X.parietina glXanPari20	Unknown
RND-1_FAMILY-44#DNA/MULE-MuDR	2631	8	X.parietina glXanPari20	DNA/MULE-MuDR
RND-1_FAMILY-74#Unknown	2592	32	X.parietina glXanPari20	Unknown
RND-1_FAMILY-26#Unknown	2575	10	X.parietina glXanPari20	Unknown
RND-1_FAMILY-72#Unknown	2546	13	X.parietina glXanPari20	Unknown
RND-1_FAMILY-12#Unknown	2531	15	X.parietina glXanPari20	Unknown
(TCC)N#Simple_repeat	2464	50	X.parietina glXanPari20	Simple_repeat
(G)N#Simple_repeat	2424	101	X.parietina glXanPari20	Simple_repeat
RND-1_FAMILY-48#Unknown	2315	24	X.parietina glXanPari20	Unknown
RND-1_FAMILY-17#Unknown	2118	6	X.parietina glXanPari20	Unknown
(GGT)N#Simple_repeat	2050	51	X.parietina glXanPari20	Simple_repeat
RND-1_FAMILY-88#Unknown	2003	16	X.parietina glXanPari20	Unknown
(CTT)N#Simple_repeat	2001	54	X.parietina glXanPari20	Simple_repeat
RND-1_FAMILY-104#Unknown	1967	5	X.parietina glXanPari20	Unknown
RND-1_FAMILY-95#Unknown	1923	17	X.parietina glXanPari20	Unknown

(CTC)N#Simple_repeat	1921	33	X.parietina glXanPari20	Simple_repeat
RND-1_FAMILY-25#Unknown	1889	11	X.parietina glXanPari20	Unknown
RND-1_FAMILY-90#Unknown	1859	15	X.parietina glXanPari20	Unknown
RND-1_FAMILY-99#Unknown	1842	8	X.parietina glXanPari20	Unknown
(CCA)N#Simple_repeat	1807	44	X.parietina glXanPari20	Simple_repeat
(TTC)N#Simple_repeat	1740	36	X.parietina glXanPari20	Simple_repeat
RND-3_FAMILY-369#Unknown	1719	27	X.parietina glXanPari20	Unknown
RND-1_FAMILY-22#Unknown	1649	11	X.parietina glXanPari20	Unknown
(GAT)N#Simple_repeat	1609	37	X.parietina glXanPari20	Simple_repeat
RND-1_FAMILY-77#Unknown	1594	10	X.parietina glXanPari20	Unknown
RND-1_FAMILY-103#Unknown	1558	6	X.parietina glXanPari20	Unknown
(T)N#Simple_repeat	1551	53	X.parietina glXanPari20	Simple_repeat
(TCT)N#Simple_repeat	1523	38	X.parietina glXanPari20	Simple_repeat
RND-1_FAMILY-73#Unknown	1500	21	X.parietina glXanPari20	Unknown
RND-1_FAMILY-56#Unknown	1481	1	X.parietina glXanPari20	Unknown
RND-1_FAMILY-83#Unknown	1425	11	X.parietina glXanPari20	Unknown
RND-1_FAMILY-14#Unknown	1379	22	X.parietina glXanPari20	Unknown
(CAA)N#Simple_repeat	1370	43	X.parietina glXanPari20	Simple_repeat
(ACC)N#Simple_repeat	1308	23	X.parietina glXanPari20	Simple_repeat
RND-1_FAMILY-85#Unknown	1308	7	X.parietina glXanPari20	Unknown
RND-1_FAMILY-55#Unknown	1264	5	X.parietina glXanPari20	Unknown
RND-1_FAMILY-23#LTR/Ty3	1144	7	X.parietina glXanPari20	LTR/Ty3
(TGG)N#Simple_repeat	1119	29	X.parietina glXanPari20	Simple_repeat
(GAA)N#Simple_repeat	1046	34	X.parietina glXanPari20	Simple_repeat
(CAC)N#Simple_repeat	1042	25	X.parietina glXanPari20	Simple_repeat
RND-1_FAMILY-87#Unknown	998	7	X.parietina glXanPari20	Unknown

(GTG)N#Simple_repeat	952	22	X.parietina glXanPari20	Simple_repeat
(CAG)N#Simple_repeat	930	29	X.parietina glXanPari20	Simple_repeat
(TC)N#Simple_repeat	923	30	X.parietina glXanPari20	Simple_repeat
(GAC)N#Simple_repeat	915	25	X.parietina glXanPari20	Simple_repeat
(GCT)N#Simple_repeat	909	26	X.parietina glXanPari20	Simple_repeat
(GGA)N#Simple_repeat	898	32	X.parietina glXanPari20	Simple_repeat
(ACA)N#Simple_repeat	831	28	X.parietina glXanPari20	Simple_repeat
(TCG)N#Simple_repeat	810	22	X.parietina glXanPari20	Simple_repeat
(CAT)N#Simple_repeat	809	27	X.parietina glXanPari20	Simple_repeat
(TGA)N#Simple_repeat	807	25	X.parietina glXanPari20	Simple_repeat
(GCA)N#Simple_repeat	801	21	X.parietina glXanPari20	Simple_repeat
RND-1_FAMILY-79#Unknown	791	5	X.parietina glXanPari20	Unknown
(TCA)N#Simple_repeat	779	20	X.parietina glXanPari20	Simple_repeat
(AGC)N#Simple_repeat	778	22	X.parietina glXanPari20	Simple_repeat
(CCCT)N#Simple_repeat	777	17	X.parietina glXanPari20	Simple_repeat
(AG)N#Simple_repeat	746	25	X.parietina glXanPari20	Simple_repeat
(CTG)N#Simple_repeat	744	16	X.parietina glXanPari20	Simple_repeat
(GTT)N#Simple_repeat	729	22	X.parietina glXanPari20	Simple_repeat
RND-3_FAMILY-538#Unknown	723	11	X.parietina glXanPari20	Unknown
(ATC)N#Simple_repeat	717	23	X.parietina glXanPari20	Simple_repeat
(GCC)N#Simple_repeat	716	20	X.parietina glXanPari20	Simple_repeat
(TGT)N#Simple_repeat	701	21	X.parietina glXanPari20	Simple_repeat
(CCG)N#Simple_repeat	700	22	X.parietina glXanPari20	Simple_repeat
(TGC)N#Simple_repeat	698	22	X.parietina glXanPari20	Simple_repeat
(CCCCTC)N#Simple_repeat	658	8	X.parietina glXanPari20	Simple_repeat
(CCCCCT)N#Simple_repeat	636	12	X.parietina glXanPari20	Simple_repeat

(GAG)N#Simple_repeat	622	20	X.parietina glXanPari20	Simple_repeat
RND-1_FAMILY-4#Unknown	602	5	X.parietina glXanPari20	Unknown
(TGGA)N#Simple_repeat	594	14	X.parietina glXanPari20	Simple_repeat
(AAG)N#Simple_repeat	590	21	X.parietina glXanPari20	Simple_repeat
RND-1_FAMILY-16#Unknown	572	4	X.parietina glXanPari20	Unknown
(CGC)N#Simple_repeat	563	14	X.parietina glXanPari20	Simple_repeat
RND-1_FAMILY-5#Unknown	562	8	X.parietina glXanPari20	Unknown
RND-1_FAMILY-93#Unknown	551	10	X.parietina glXanPari20	Unknown
(CGA)N#Simple_repeat	546	13	X.parietina glXanPari20	Simple_repeat
(A)N#Simple_repeat	534	24	X.parietina glXanPari20	Simple_repeat
(CT)N#Simple_repeat	527	20	X.parietina glXanPari20	Simple_repeat
(GGAT)N#Simple_repeat	514	11	X.parietina glXanPari20	Simple_repeat
(CCTCCA)N#Simple_repeat	499	8	X.parietina glXanPari20	Simple_repeat
(AC)N#Simple_repeat	493	16	X.parietina glXanPari20	Simple_repeat
(GGC)N#Simple_repeat	490	13	X.parietina glXanPari20	Simple_repeat
(ATG)N#Simple_repeat	481	11	X.parietina glXanPari20	Simple_repeat
(CTCCCC)N#Simple_repeat	473	5	X.parietina glXanPari20	Simple_repeat
(CCTC)N#Simple_repeat	468	12	X.parietina glXanPari20	Simple_repeat
(CTCC)N#Simple_repeat	465	10	X.parietina glXanPari20	Simple_repeat
(GGAGGT)N#Simple_repeat	465	8	X.parietina glXanPari20	Simple_repeat
(TCCTTC)N#Simple_repeat	455	5	X.parietina glXanPari20	Simple_repeat
(AAC)N#Simple_repeat	451	15	X.parietina glXanPari20	Simple_repeat
(CGG)N#Simple_repeat	448	16	X.parietina glXanPari20	Simple_repeat
(TG)N#Simple_repeat	426	14	X.parietina glXanPari20	Simple_repeat
(TACCT)N#Simple_repeat	425	11	X.parietina glXanPari20	Simple_repeat
(TCCC)N#Simple_repeat	425	10	X.parietina glXanPari20	Simple_repeat

(GGTGGA)N#Simple_repeat	421	5	X.parietina glXanPari20	Simple_repeat
(CCTCCC)N#Simple_repeat	416	6	X.parietina glXanPari20	Simple_repeat
(GTC)N#Simple_repeat	400	12	X.parietina glXanPari20	Simple_repeat
(CCAC)N#Simple_repeat	388	8	X.parietina glXanPari20	Simple_repeat
(TTTC)N#Simple_repeat	388	10	X.parietina glXanPari20	Simple_repeat
(AGA)N#Simple_repeat	383	13	X.parietina glXanPari20	Simple_repeat
(CGT)N#Simple_repeat	383	9	X.parietina glXanPari20	Simple_repeat
(CCCATC)N#Simple_repeat	382	4	X.parietina glXanPari20	Simple_repeat
(TA)N#Simple_repeat	374	13	X.parietina glXanPari20	Simple_repeat
(AT)N#Simple_repeat	354	10	X.parietina glXanPari20	Simple_repeat
(GT)N#Simple_repeat	353	13	X.parietina glXanPari20	Simple_repeat
(CCCACC)N#Simple_repeat	345	3	X.parietina glXanPari20	Simple_repeat
(TGGAGG)N#Simple_repeat	345	8	X.parietina glXanPari20	Simple_repeat
(TGCTGT)N#Simple_repeat	342	4	X.parietina glXanPari20	Simple_repeat
(GTGG)N#Simple_repeat	337	6	X.parietina glXanPari20	Simple_repeat
(CCCCAT)N#Simple_repeat	330	6	X.parietina glXanPari20	Simple_repeat
(CCACCT)N#Simple_repeat	328	5	X.parietina glXanPari20	Simple_repeat
(CATCC)N#Simple_repeat	322	8	X.parietina glXanPari20	Simple_repeat
(GAGGAC)N#Simple_repeat	322	4	X.parietina glXanPari20	Simple_repeat
(GCG)N#Simple_repeat	320	9	X.parietina glXanPari20	Simple_repeat
(TTG)N#Simple_repeat	319	10	X.parietina glXanPari20	Simple_repeat
(GGAGGC)N#Simple_repeat	317	4	X.parietina glXanPari20	Simple_repeat
(TCCA)N#Simple_repeat	313	8	X.parietina glXanPari20	Simple_repeat
(CA)N#Simple_repeat	312	11	X.parietina glXanPari20	Simple_repeat
(ACG)N#Simple_repeat	311	11	X.parietina glXanPari20	Simple_repeat
(GA)N#Simple_repeat	311	13	X.parietina glXanPari20	Simple_repeat

(TCGTCC)N#Simple_repeat	308	5	X.parietina glXanPari20	Simple_repeat
(CCC)N#Simple_repeat	307	4	X.parietina glXanPari20	Simple_repeat
(CCTTCC)N#Simple_repeat	305	5	X.parietina glXanPari20	Simple_repeat
(CATCCC)N#Simple_repeat	297	3	X.parietina glXanPari20	Simple_repeat
(TCCCCC)N#Simple_repeat	293	5	X.parietina glXanPari20	Simple_repeat
(ACCCCC)N#Simple_repeat	290	6	X.parietina glXanPari20	Simple_repeat
(CCTCGT)N#Simple_repeat	282	4	X.parietina glXanPari20	Simple_repeat
(CATCTC)N#Simple_repeat	272	6	X.parietina glXanPari20	Simple_repeat
(CCGCCT)N#Simple_repeat	272	5	X.parietina glXanPari20	Simple_repeat
(GATGCTGT)N#Simple_repeat	270	1	X.parietina glXanPari20	Simple_repeat
(CTCCTT)N#Simple_repeat	268	3	X.parietina glXanPari20	Simple_repeat
(TAT)N#Simple_repeat	262	5	X.parietina glXanPari20	Simple_repeat
(CCTCC)N#Simple_repeat	261	5	X.parietina glXanPari20	Simple_repeat
(CCAT)N#Simple_repeat	259	6	X.parietina glXanPari20	Simple_repeat
(AATC)N#Simple_repeat	257	6	X.parietina glXanPari20	Simple_repeat
(GAAGAC)N#Simple_repeat	255	4	X.parietina glXanPari20	Simple_repeat
(TCCTCT)N#Simple_repeat	251	3	X.parietina glXanPari20	Simple_repeat
(GAAGAG)N#Simple_repeat	248	2	X.parietina glXanPari20	Simple_repeat
(GAGGAGGAA)N#Simple_repeat	248	1	X.parietina glXanPari20	Simple_repeat
(GATG)N#Simple_repeat	246	7	X.parietina glXanPari20	Simple_repeat
(AGG)N#Simple_repeat	243	9	X.parietina glXanPari20	Simple_repeat
(CCCTCT)N#Simple_repeat	242	6	X.parietina glXanPari20	Simple_repeat
(TCCCC)N#Simple_repeat	242	4	X.parietina glXanPari20	Simple_repeat
(CCACCG)N#Simple_repeat	236	5	X.parietina glXanPari20	Simple_repeat
(CCCTCC)N#Simple_repeat	236	3	X.parietina glXanPari20	Simple_repeat
(GACGAA)N#Simple_repeat	233	5	X.parietina glXanPari20	Simple_repeat

(CCCA)N#Simple_repeat	229	4	X.parietina glXanPari20	Simple_repeat
(GGACGA)N#Simple_repeat	228	4	X.parietina glXanPari20	Simple_repeat
(ATT)N#Simple_repeat	225	6	X.parietina glXanPari20	Simple_repeat
(TCTTCG)N#Simple_repeat	220	4	X.parietina glXanPari20	Simple_repeat
(ACCTCC)N#Simple_repeat	216	4	X.parietina glXanPari20	Simple_repeat
(AGGTA)N#Simple_repeat	216	6	X.parietina glXanPari20	Simple_repeat
(TTA)N#Simple_repeat	215	8	X.parietina glXanPari20	Simple_repeat
(CATCCT)N#Simple_repeat	212	4	X.parietina glXanPari20	Simple_repeat
(TCCTCC)N#Simple_repeat	210	2	X.parietina glXanPari20	Simple_repeat
(AAT)N#Simple_repeat	207	6	X.parietina glXanPari20	Simple_repeat
(CCATC)N#Simple_repeat	207	6	X.parietina glXanPari20	Simple_repeat
(AGAGCC)N#Simple_repeat	204	5	X.parietina glXanPari20	Simple_repeat
(ATTG)N#Simple_repeat	203	5	X.parietina glXanPari20	Simple_repeat
(CCTCTC)N#Simple_repeat	203	3	X.parietina glXanPari20	Simple_repeat
(CCTCCT)N#Simple_repeat	201	2	X.parietina glXanPari20	Simple_repeat
(ATGG)N#Simple_repeat	197	3	X.parietina glXanPari20	Simple_repeat
(CCCCAA)N#Simple_repeat	196	4	X.parietina glXanPari20	Simple_repeat
(CTTTCTCCCC)N#Simple_repeat	193	1	X.parietina glXanPari20	Simple_repeat
(GATT)N#Simple_repeat	192	5	X.parietina glXanPari20	Simple_repeat
(CTTCCC)N#Simple_repeat	191	3	X.parietina glXanPari20	Simple_repeat
(GGGAA)N#Simple_repeat	191	3	X.parietina glXanPari20	Simple_repeat
(ATCCC)N#Simple_repeat	190	4	X.parietina glXanPari20	Simple_repeat
(GATGAC)N#Simple_repeat	190	5	X.parietina glXanPari20	Simple_repeat
(GGATGA)N#Simple_repeat	190	5	X.parietina glXanPari20	Simple_repeat
(CACCAC)N#Simple_repeat	189	3	X.parietina glXanPari20	Simple_repeat
(GAGAT)N#Simple_repeat	188	4	X.parietina glXanPari20	Simple_repeat

(CCTCTT)N#Simple_repeat	186	3	X.parietina glXanPari20	Simple_repeat
(GTAAG)N#Simple_repeat	186	6	X.parietina glXanPari20	Simple_repeat
(CCTCCCC)N#Simple_repeat	185	3	X.parietina glXanPari20	Simple_repeat
(CTCCCAC)N#Simple_repeat	184	2	X.parietina glXanPari20	Simple_repeat
(GGAA)N#Simple_repeat	184	7	X.parietina glXanPari20	Simple_repeat
(TGAGAG)N#Simple_repeat	184	2	X.parietina glXanPari20	Simple_repeat
(TGGTGA)N#Simple_repeat	183	3	X.parietina glXanPari20	Simple_repeat
(TTT)N#Simple_repeat	183	4	X.parietina glXanPari20	Simple_repeat
(AGATGA)N#Simple_repeat	182	4	X.parietina glXanPari20	Simple_repeat
(CCACCA)N#Simple_repeat	181	3	X.parietina glXanPari20	Simple_repeat
(CGCTCC)N#Simple_repeat	181	1	X.parietina glXanPari20	Simple_repeat
(CTTTT)N#Simple_repeat	181	5	X.parietina glXanPari20	Simple_repeat
(CCCTCG)N#Simple_repeat	180	3	X.parietina glXanPari20	Simple_repeat
(CCTCAT)N#Simple_repeat	180	3	X.parietina glXanPari20	Simple_repeat
(CCTCGACCT)N#Simple_repeat	180	4	X.parietina glXanPari20	Simple_repeat
(GAGGTCGAG)N#Simple_repeat	180	4	X.parietina glXanPari20	Simple_repeat
(GCCACC)N#Simple_repeat	180	2	X.parietina glXanPari20	Simple_repeat
(TACC)N#Simple_repeat	180	5	X.parietina glXanPari20	Simple_repeat
(AGGGAAA)N#Simple_repeat	176	1	X.parietina glXanPari20	Simple_repeat
(GACGAT)N#Simple_repeat	175	4	X.parietina glXanPari20	Simple_repeat
(TGGAGA)N#Simple_repeat	175	4	X.parietina glXanPari20	Simple_repeat
RND-1_FAMILY-13#Unknown	175	2	X.parietina glXanPari20	Unknown
(CCCTTC)N#Simple_repeat	174	2	X.parietina glXanPari20	Simple_repeat
(GCAGGA)N#Simple_repeat	173	3	X.parietina glXanPari20	Simple_repeat
(GGTAA)N#Simple_repeat	173	5	X.parietina glXanPari20	Simple_repeat
(TGTTGG)N#Simple_repeat	173	4	X.parietina glXanPari20	Simple_repeat

(TCTTCA)N#Simple_repeat	172	3	X.parietina glXanPari20	Simple_repeat
(CACCAT)N#Simple_repeat	171	4	X.parietina glXanPari20	Simple_repeat
(CTCGTC)N#Simple_repeat	171	4	X.parietina glXanPari20	Simple_repeat
(CTCGCC)N#Simple_repeat	170	3	X.parietina glXanPari20	Simple_repeat
(CCTTTC)N#Simple_repeat	168	3	X.parietina glXanPari20	Simple_repeat
(TCATCG)N#Simple_repeat	168	4	X.parietina glXanPari20	Simple_repeat
(TCCCCA)N#Simple_repeat	168	2	X.parietina glXanPari20	Simple_repeat
(CATC)N#Simple_repeat	166	5	X.parietina glXanPari20	Simple_repeat
(TGTCAT)N#Simple_repeat	165	2	X.parietina glXanPari20	Simple_repeat
(CCTCT)N#Simple_repeat	164	5	X.parietina glXanPari20	Simple_repeat
(TTCTCC)N#Simple_repeat	163	3	X.parietina glXanPari20	Simple_repeat
(GAGGCG)N#Simple_repeat	162	2	X.parietina glXanPari20	Simple_repeat
(GAAGCA)N#Simple_repeat	161	3	X.parietina glXanPari20	Simple_repeat
(ACCCTC)N#Simple_repeat	160	3	X.parietina glXanPari20	Simple_repeat
(TCTT)N#Simple_repeat	160	4	X.parietina glXanPari20	Simple_repeat
(TCTTCC)N#Simple_repeat	159	3	X.parietina glXanPari20	Simple_repeat
(AGTAGC)N#Simple_repeat	157	1	X.parietina glXanPari20	Simple_repeat
(TTTTC)N#Simple_repeat	156	4	X.parietina glXanPari20	Simple_repeat
(CATA)N#Simple_repeat	155	5	X.parietina glXanPari20	Simple_repeat
(TAAGG)N#Simple_repeat	155	4	X.parietina glXanPari20	Simple_repeat
(TGGC)N#Simple_repeat	155	5	X.parietina glXanPari20	Simple_repeat
(CCTTCT)N#Simple_repeat	154	2	X.parietina glXanPari20	Simple_repeat
(GTCATC)N#Simple_repeat	153	2	X.parietina glXanPari20	Simple_repeat
(TGATGG)N#Simple_repeat	149	3	X.parietina glXanPari20	Simple_repeat
(CGAGGAGGT)N#Simple_repeat	148	1	X.parietina glXanPari20	Simple_repeat
(CTTT)N#Simple_repeat	148	5	X.parietina glXanPari20	Simple_repeat

(GATGAG)N#Simple_repeat	148	3	X.parietina glXanPari20	Simple_repeat
(GAGGATG)N#Simple_repeat	147	3	X.parietina glXanPari20	Simple_repeat
(AGGT)N#Simple_repeat	146	4	X.parietina glXanPari20	Simple_repeat
(GAGGTG)N#Simple_repeat	146	3	X.parietina glXanPari20	Simple_repeat
(GATGAA)N#Simple_repeat	146	3	X.parietina glXanPari20	Simple_repeat
(TCAA)N#Simple_repeat	146	5	X.parietina glXanPari20	Simple_repeat
(AAGCCA)N#Simple_repeat	145	3	X.parietina glXanPari20	Simple_repeat
(CCCCT)N#Simple_repeat	145	3	X.parietina glXanPari20	Simple_repeat
(GCTTT)N#Simple_repeat	145	1	X.parietina glXanPari20	Simple_repeat
(AGGATG)N#Simple_repeat	144	3	X.parietina glXanPari20	Simple_repeat
(GCAA)N#Simple_repeat	142	4	X.parietina glXanPari20	Simple_repeat
(TAA)N#Simple_repeat	142	4	X.parietina glXanPari20	Simple_repeat
(TCAACC)N#Simple_repeat	142	3	X.parietina glXanPari20	Simple_repeat
(CCCAA)N#Simple_repeat	141	4	X.parietina glXanPari20	Simple_repeat
(CGGTG)N#Simple_repeat	141	2	X.parietina glXanPari20	Simple_repeat
(CTA)N#Simple_repeat	140	4	X.parietina glXanPari20	Simple_repeat
(CTCCAC)N#Simple_repeat	140	5	X.parietina glXanPari20	Simple_repeat
(CCTA)N#Simple_repeat	139	5	X.parietina glXanPari20	Simple_repeat
(TGTGCT)N#Simple_repeat	138	1	X.parietina glXanPari20	Simple_repeat
(TTCCT)N#Simple_repeat	138	3	X.parietina glXanPari20	Simple_repeat
(AAAGA)N#Simple_repeat	137	2	X.parietina glXanPari20	Simple_repeat
(CGAAGA)N#Simple_repeat	137	2	X.parietina glXanPari20	Simple_repeat
(TCATCT)N#Simple_repeat	137	2	X.parietina glXanPari20	Simple_repeat
(CCCATCT)N#Simple_repeat	136	3	X.parietina glXanPari20	Simple_repeat
(TCGTCT)N#Simple_repeat	136	2	X.parietina glXanPari20	Simple_repeat
RND-1_FAMILY-64#Unknown	136	1	X.parietina glXanPari20	Unknown

(GCCTCC)N#Simple_repeat	135	3	X.parietina glXanPari20	Simple_repeat
(GGTGGTTGT)N#Simple_repeat	135	1	X.parietina glXanPari20	Simple_repeat
(ACT)N#Simple_repeat	134	4	X.parietina glXanPari20	Simple_repeat
(GGTGGT)N#Simple_repeat	132	3	X.parietina glXanPari20	Simple_repeat
(CGCCAA)N#Simple_repeat	131	3	X.parietina glXanPari20	Simple_repeat
(CTTCC)N#Simple_repeat	131	2	X.parietina glXanPari20	Simple_repeat
(GGTGTG)N#Simple_repeat	131	2	X.parietina glXanPari20	Simple_repeat
(TTGCTG)N#Simple_repeat	131	3	X.parietina glXanPari20	Simple_repeat
(ATCCCC)N#Simple_repeat	130	1	X.parietina glXanPari20	Simple_repeat
(CTAC)N#Simple_repeat	130	4	X.parietina glXanPari20	Simple_repeat
(TCCTCA)N#Simple_repeat	130	3	X.parietina glXanPari20	Simple_repeat
(CCATCC)N#Simple_repeat	129	2	X.parietina glXanPari20	Simple_repeat
(GGAAGG)N#Simple_repeat	129	2	X.parietina glXanPari20	Simple_repeat
(CCCCTT)N#Simple_repeat	128	3	X.parietina glXanPari20	Simple_repeat
(GAGGAT)N#Simple_repeat	128	2	X.parietina glXanPari20	Simple_repeat
(CCCCCG)N#Simple_repeat	127	2	X.parietina glXanPari20	Simple_repeat
(CCTCGC)N#Simple_repeat	127	3	X.parietina glXanPari20	Simple_repeat
(TCTCCC)N#Simple_repeat	127	2	X.parietina glXanPari20	Simple_repeat
(TGGATG)N#Simple_repeat	127	1	X.parietina glXanPari20	Simple_repeat
(CCCAT)N#Simple_repeat	126	4	X.parietina glXanPari20	Simple_repeat
(GTGGA)N#Simple_repeat	126	1	X.parietina glXanPari20	Simple_repeat
(TTCCCC)N#Simple_repeat	126	2	X.parietina glXanPari20	Simple_repeat
(AGATGG)N#Simple_repeat	125	3	X.parietina glXanPari20	Simple_repeat
(ATGAA)N#Simple_repeat	125	3	X.parietina glXanPari20	Simple_repeat
(CTCCT)N#Simple_repeat	124	4	X.parietina glXanPari20	Simple_repeat
(TCCCAA)N#Simple_repeat	123	2	X.parietina glXanPari20	Simple_repeat

(CTCTTC)N#Simple_repeat	122	3	X.parietina glXanPari20	Simple_repeat
(GGAGCT)N#Simple_repeat	122	3	X.parietina glXanPari20	Simple_repeat
(CCCCGC)N#Simple_repeat	121	2	X.parietina glXanPari20	Simple_repeat
(CTCTCC)N#Simple_repeat	121	3	X.parietina glXanPari20	Simple_repeat
(GAGTGG)N#Simple_repeat	121	1	X.parietina glXanPari20	Simple_repeat
(TCTCC)N#Simple_repeat	120	3	X.parietina glXanPari20	Simple_repeat
(CTTCTC)N#Simple_repeat	119	2	X.parietina glXanPari20	Simple_repeat
(GGATA)N#Simple_repeat	119	2	X.parietina glXanPari20	Simple_repeat
(GGATGG)N#Simple_repeat	119	2	X.parietina glXanPari20	Simple_repeat
(GTGGAT)N#Simple_repeat	119	3	X.parietina glXanPari20	Simple_repeat
(CAGCAACAG)N#Simple_repeat	118	1	X.parietina glXanPari20	Simple_repeat
(GGTGAGA)N#Simple_repeat	118	2	X.parietina glXanPari20	Simple_repeat
(CACT)N#Simple_repeat	117	4	X.parietina glXanPari20	Simple_repeat
(CTTC)N#Simple_repeat	117	4	X.parietina glXanPari20	Simple_repeat
(TCCCT)N#Simple_repeat	117	3	X.parietina glXanPari20	Simple_repeat
(ACCCC)N#Simple_repeat	116	3	X.parietina glXanPari20	Simple_repeat
(GCTGAT)N#Simple_repeat	116	3	X.parietina glXanPari20	Simple_repeat
(TCATCC)N#Simple_repeat	116	3	X.parietina glXanPari20	Simple_repeat
(TCTTCT)N#Simple_repeat	116	2	X.parietina glXanPari20	Simple_repeat
(CAGAAC)N#Simple_repeat	115	1	X.parietina glXanPari20	Simple_repeat
(CAAGCC)N#Simple_repeat	114	2	X.parietina glXanPari20	Simple_repeat
(CCCCGA)N#Simple_repeat	113	2	X.parietina glXanPari20	Simple_repeat
(TCCAGA)N#Simple_repeat	113	1	X.parietina glXanPari20	Simple_repeat
(TCGTCA)N#Simple_repeat	113	2	X.parietina glXanPari20	Simple_repeat
(TTTATC)N#Simple_repeat	113	2	X.parietina glXanPari20	Simple_repeat
(CACCAAC)N#Simple_repeat	112	2	X.parietina glXanPari20	Simple_repeat

(TATTA)N#Simple_repeat	112	2	X.parietina glXanPari20	Simple_repeat
(CGAAGG)N#Simple_repeat	111	2	X.parietina glXanPari20	Simple_repeat
(CTCCCT)N#Simple_repeat	111	2	X.parietina glXanPari20	Simple_repeat
(GTGGTG)N#Simple_repeat	111	2	X.parietina glXanPari20	Simple_repeat
(TCCCCTC)N#Simple_repeat	111	3	X.parietina glXanPari20	Simple_repeat
(TTCTT)N#Simple_repeat	111	3	X.parietina glXanPari20	Simple_repeat
(ACACCA)N#Simple_repeat	110	3	X.parietina glXanPari20	Simple_repeat
(ATGGGTCA)N#Simple_repeat	110	1	X.parietina glXanPari20	Simple_repeat
(CAACAG)N#Simple_repeat	110	3	X.parietina glXanPari20	Simple_repeat
(TCTTCTT)N#Simple_repeat	109	3	X.parietina glXanPari20	Simple_repeat
(CCCTTTC)N#Simple_repeat	108	2	X.parietina glXanPari20	Simple_repeat
(CTCCTG)N#Simple_repeat	108	3	X.parietina glXanPari20	Simple_repeat
(ACCT)N#Simple_repeat	107	4	X.parietina glXanPari20	Simple_repeat
(TTTCTC)N#Simple_repeat	107	3	X.parietina glXanPari20	Simple_repeat
(CATCCCT)N#Simple_repeat	106	2	X.parietina glXanPari20	Simple_repeat
(CCCACCAC)N#Simple_repeat	106	1	X.parietina glXanPari20	Simple_repeat
(CTTCGC)N#Simple_repeat	106	1	X.parietina glXanPari20	Simple_repeat
(TGTG)N#Simple_repeat	106	1	X.parietina glXanPari20	Simple_repeat
(AGGA)N#Simple_repeat	105	3	X.parietina glXanPari20	Simple_repeat
(GATAT)N#Simple_repeat	105	2	X.parietina glXanPari20	Simple_repeat
(GGATG)N#Simple_repeat	105	2	X.parietina glXanPari20	Simple_repeat
(CCCAAT)N#Simple_repeat	104	2	X.parietina glXanPari20	Simple_repeat
(CCCCCCT)N#Simple_repeat	104	2	X.parietina glXanPari20	Simple_repeat
(CTATATA)N#Simple_repeat	104	2	X.parietina glXanPari20	Simple_repeat
(CTGAGG)N#Simple_repeat	104	1	X.parietina glXanPari20	Simple_repeat
(TAG)N#Simple_repeat	104	3	X.parietina glXanPari20	Simple_repeat

(TTTCT)N#Simple_repeat	104	2	X.parietina glXanPari20	Simple_repeat
(AAGACG)N#Simple_repeat	103	3	X.parietina glXanPari20	Simple_repeat
(ACCTCA)N#Simple_repeat	102	2	X.parietina glXanPari20	Simple_repeat
(ACGAAG)N#Simple_repeat	102	1	X.parietina glXanPari20	Simple_repeat
(GAGG)N#Simple_repeat	102	3	X.parietina glXanPari20	Simple_repeat
(GGCGGT)N#Simple_repeat	102	3	X.parietina glXanPari20	Simple_repeat
(GTAG)N#Simple_repeat	102	4	X.parietina glXanPari20	Simple_repeat
(CCCCC)N#Simple_repeat	101	2	X.parietina glXanPari20	Simple_repeat
(GACGAC)N#Simple_repeat	101	2	X.parietina glXanPari20	Simple_repeat
(TCATCA)N#Simple_repeat	101	2	X.parietina glXanPari20	Simple_repeat
(TCCCTT)N#Simple_repeat	101	2	X.parietina glXanPari20	Simple_repeat
(ACCA)N#Simple_repeat	100	2	X.parietina glXanPari20	Simple_repeat
(CCTCCAT)N#Simple_repeat	100	2	X.parietina glXanPari20	Simple_repeat
(CGGCGC)N#Simple_repeat	100	2	X.parietina glXanPari20	Simple_repeat
(ATCACC)N#Simple_repeat	99	1	X.parietina glXanPari20	Simple_repeat
(CCATCCT)N#Simple_repeat	99	1	X.parietina glXanPari20	Simple_repeat
(CTCACC)N#Simple_repeat	99	2	X.parietina glXanPari20	Simple_repeat
(TCCTCG)N#Simple_repeat	99	3	X.parietina glXanPari20	Simple_repeat
(TGGTGG)N#Simple_repeat	99	2	X.parietina glXanPari20	Simple_repeat
RND-1_FAMILY-39#Unknown	99	1	X.parietina glXanPari20	Unknown
(CATCAC)N#Simple_repeat	98	2	X.parietina glXanPari20	Simple_repeat
(GGTGGC)N#Simple_repeat	98	2	X.parietina glXanPari20	Simple_repeat
(GTCGGA)N#Simple_repeat	98	1	X.parietina glXanPari20	Simple_repeat
(CACCCT)N#Simple_repeat	97	2	X.parietina glXanPari20	Simple_repeat
(TCCCA)N#Simple_repeat	97	3	X.parietina glXanPari20	Simple_repeat
(CTCACCA)N#Simple_repeat	96	2	X.parietina glXanPari20	Simple_repeat

(TGGAGGA)N#Simple_repeat	96	2	X.parietina glXanPari20	Simple_repeat
(AGGAGAAGG)N#Simple_repeat	95	1	X.parietina glXanPari20	Simple_repeat
(CAACACC)N#Simple_repeat	95	2	X.parietina glXanPari20	Simple_repeat
(GCGTTG)N#Simple_repeat	95	1	X.parietina glXanPari20	Simple_repeat
(TTGA)N#Simple_repeat	95	3	X.parietina glXanPari20	Simple_repeat
(AGATG)N#Simple_repeat	94	3	X.parietina glXanPari20	Simple_repeat
(CTTAC)N#Simple_repeat	94	3	X.parietina glXanPari20	Simple_repeat
(GCGACA)N#Simple_repeat	94	1	X.parietina glXanPari20	Simple_repeat
(TATAT)N#Simple_repeat	94	1	X.parietina glXanPari20	Simple_repeat
(TTCT)N#Simple_repeat	94	3	X.parietina glXanPari20	Simple_repeat
(ACCC)N#Simple_repeat	93	3	X.parietina glXanPari20	Simple_repeat
(AGGAT)N#Simple_repeat	93	1	X.parietina glXanPari20	Simple_repeat
(CGCCCT)N#Simple_repeat	93	2	X.parietina glXanPari20	Simple_repeat
(GATGGT)N#Simple_repeat	93	2	X.parietina glXanPari20	Simple_repeat
(CCCTC)N#Simple_repeat	92	2	X.parietina glXanPari20	Simple_repeat
(GGAG)N#Simple_repeat	92	3	X.parietina glXanPari20	Simple_repeat
(GGTGAAG)N#Simple_repeat	92	2	X.parietina glXanPari20	Simple_repeat
(TCAT)N#Simple_repeat	92	3	X.parietina glXanPari20	Simple_repeat
(TGAT)N#Simple_repeat	92	3	X.parietina glXanPari20	Simple_repeat
(CCCCCA)N#Simple_repeat	91	3	X.parietina glXanPari20	Simple_repeat
(GGATTT)N#Simple_repeat	91	2	X.parietina glXanPari20	Simple_repeat
(TCCCTC)N#Simple_repeat	91	2	X.parietina glXanPari20	Simple_repeat
(TCTA)N#Simple_repeat	91	2	X.parietina glXanPari20	Simple_repeat
(TGTTGA)N#Simple_repeat	91	1	X.parietina glXanPari20	Simple_repeat
(CCACCAC)N#Simple_repeat	90	1	X.parietina glXanPari20	Simple_repeat
(GGTGCG)N#Simple_repeat	90	1	X.parietina glXanPari20	Simple_repeat

(CAACT)N#Simple_repeat	89	3	X.parietina glXanPari20	Simple_repeat
(CACCCCT)N#Simple_repeat	89	2	X.parietina glXanPari20	Simple_repeat
(CCATCA)N#Simple_repeat	89	2	X.parietina glXanPari20	Simple_repeat
(GAGAG)N#Simple_repeat	89	2	X.parietina glXanPari20	Simple_repeat
(TCCCTCA)N#Simple_repeat	89	2	X.parietina glXanPari20	Simple_repeat
(TTGTGA)N#Simple_repeat	89	2	X.parietina glXanPari20	Simple_repeat
(ATGACT)N#Simple_repeat	88	2	X.parietina glXanPari20	Simple_repeat
(CCTCCG)N#Simple_repeat	88	2	X.parietina glXanPari20	Simple_repeat
(CTCCAT)N#Simple_repeat	88	1	X.parietina glXanPari20	Simple_repeat
(GAAGAAGGG)N#Simple_repeat	88	1	X.parietina glXanPari20	Simple_repeat
(TCCAA)N#Simple_repeat	88	2	X.parietina glXanPari20	Simple_repeat
(AAGCCG)N#Simple_repeat	87	1	X.parietina glXanPari20	Simple_repeat
(CTCTAGTAC)N#Simple_repeat	87	1	X.parietina glXanPari20	Simple_repeat
(TTCCC)N#Simple_repeat	87	3	X.parietina glXanPari20	Simple_repeat
(TTGG)N#Simple_repeat	87	3	X.parietina glXanPari20	Simple_repeat
(ACAC)N#Simple_repeat	86	2	X.parietina glXanPari20	Simple_repeat
(ATAATAA)N#Simple_repeat	86	1	X.parietina glXanPari20	Simple_repeat
(CAACCCT)N#Simple_repeat	86	2	X.parietina glXanPari20	Simple_repeat
(GCAGAG)N#Simple_repeat	86	2	X.parietina glXanPari20	Simple_repeat
(GTTAGAA)N#Simple_repeat	86	1	X.parietina glXanPari20	Simple_repeat
(TCCTC)N#Simple_repeat	86	3	X.parietina glXanPari20	Simple_repeat
(TTCCCCT)N#Simple_repeat	86	2	X.parietina glXanPari20	Simple_repeat
(TTCTCTC)N#Simple_repeat	86	2	X.parietina glXanPari20	Simple_repeat
(ATCA)N#Simple_repeat	85	3	X.parietina glXanPari20	Simple_repeat
(CCAACA)N#Simple_repeat	85	2	X.parietina glXanPari20	Simple_repeat
(GCGATG)N#Simple_repeat	85	2	X.parietina glXanPari20	Simple_repeat

(GGGAT)N#Simple_repeat	85	3	X.parietina glXanPari20	Simple_repeat
(GTGA)N#Simple_repeat	85	4	X.parietina glXanPari20	Simple_repeat
(TGAAGA)N#Simple_repeat	85	2	X.parietina glXanPari20	Simple_repeat
(TGGTTGA)N#Simple_repeat	85	1	X.parietina glXanPari20	Simple_repeat
(TGTGAGA)N#Simple_repeat	85	1	X.parietina glXanPari20	Simple_repeat
(TCGTT)N#Simple_repeat	84	2	X.parietina glXanPari20	Simple_repeat
(TGGAC)N#Simple_repeat	84	2	X.parietina glXanPari20	Simple_repeat
(TGTA)N#Simple_repeat	84	3	X.parietina glXanPari20	Simple_repeat
(CCACAA)N#Simple_repeat	83	2	X.parietina glXanPari20	Simple_repeat
(CTCCTC)N#Simple_repeat	83	2	X.parietina glXanPari20	Simple_repeat
(TCACCA)N#Simple_repeat	83	1	X.parietina glXanPari20	Simple_repeat
(TCTCCA)N#Simple_repeat	83	1	X.parietina glXanPari20	Simple_repeat
(ACAA)N#Simple_repeat	82	2	X.parietina glXanPari20	Simple_repeat
(CCTTTG)N#Simple_repeat	82	1	X.parietina glXanPari20	Simple_repeat
(TCACT)N#Simple_repeat	82	2	X.parietina glXanPari20	Simple_repeat
(TCCACACC)N#Simple_repeat	82	1	X.parietina glXanPari20	Simple_repeat
(TCTTGCT)N#Simple_repeat	82	1	X.parietina glXanPari20	Simple_repeat
(AACATCGC)N#Simple_repeat	81	1	X.parietina glXanPari20	Simple_repeat
(AATTTTA)N#Simple_repeat	81	1	X.parietina glXanPari20	Simple_repeat
(ACAACA)N#Simple_repeat	81	2	X.parietina glXanPari20	Simple_repeat
(AGGGGA)N#Simple_repeat	81	2	X.parietina glXanPari20	Simple_repeat
(CCACTC)N#Simple_repeat	81	2	X.parietina glXanPari20	Simple_repeat
(CCATCT)N#Simple_repeat	81	2	X.parietina glXanPari20	Simple_repeat
(GATAATA)N#Simple_repeat	81	1	X.parietina glXanPari20	Simple_repeat
(TGGAT)N#Simple_repeat	81	2	X.parietina glXanPari20	Simple_repeat
(ACGAGC)N#Simple_repeat	80	2	X.parietina glXanPari20	Simple_repeat

(ATGGT)N#Simple_repeat	80	2	X.parietina glXanPari20	Simple_repeat
(CCAG)N#Simple_repeat	80	3	X.parietina glXanPari20	Simple_repeat
(CCTCATC)N#Simple_repeat	80	1	X.parietina glXanPari20	Simple_repeat
(GAGGCA)N#Simple_repeat	80	2	X.parietina glXanPari20	Simple_repeat
(GGCGCT)N#Simple_repeat	80	2	X.parietina glXanPari20	Simple_repeat
(GTGGTT)N#Simple_repeat	80	2	X.parietina glXanPari20	Simple_repeat
(TTAAGA)N#Simple_repeat	80	2	X.parietina glXanPari20	Simple_repeat
(ATCTCC)N#Simple_repeat	79	2	X.parietina glXanPari20	Simple_repeat
(CACAGA)N#Simple_repeat	79	1	X.parietina glXanPari20	Simple_repeat
(GGTGG)N#Simple_repeat	79	2	X.parietina glXanPari20	Simple_repeat
(AGAAA)N#Simple_repeat	78	2	X.parietina glXanPari20	Simple_repeat
(ATCC)N#Simple_repeat	78	2	X.parietina glXanPari20	Simple_repeat
(CACAAGAAG)N#Simple_repeat	78	1	X.parietina glXanPari20	Simple_repeat
(CACCCA)N#Simple_repeat	78	2	X.parietina glXanPari20	Simple_repeat
(CGATGA)N#Simple_repeat	78	2	X.parietina glXanPari20	Simple_repeat
(CTTCCT)N#Simple_repeat	78	2	X.parietina glXanPari20	Simple_repeat
(GAAGAT)N#Simple_repeat	78	2	X.parietina glXanPari20	Simple_repeat
(TAC)N#Simple_repeat	78	3	X.parietina glXanPari20	Simple_repeat
(AGAAGATT)N#Simple_repeat	77	1	X.parietina glXanPari20	Simple_repeat
(CGTCGA)N#Simple_repeat	77	1	X.parietina glXanPari20	Simple_repeat
(GAGAC)N#Simple_repeat	77	2	X.parietina glXanPari20	Simple_repeat
(GGAGAA)N#Simple_repeat	77	2	X.parietina glXanPari20	Simple_repeat
(GGCTTT)N#Simple_repeat	77	2	X.parietina glXanPari20	Simple_repeat
(GGTAT)N#Simple_repeat	77	2	X.parietina glXanPari20	Simple_repeat
(GTGATGG)N#Simple_repeat	77	1	X.parietina glXanPari20	Simple_repeat
(TTACC)N#Simple_repeat	77	2	X.parietina glXanPari20	Simple_repeat

(CCCAG)N#Simple_repeat	76	2	X.parietina glXanPari20	Simple_repeat
(CGCACCT)N#Simple_repeat	76	1	X.parietina glXanPari20	Simple_repeat
(CTCCC)N#Simple_repeat	76	3	X.parietina glXanPari20	Simple_repeat
(GATA)N#Simple_repeat	76	3	X.parietina glXanPari20	Simple_repeat
(TCTGGC)N#Simple_repeat	76	2	X.parietina glXanPari20	Simple_repeat
(TCTTC)N#Simple_repeat	76	2	X.parietina glXanPari20	Simple_repeat
(TGGAGC)N#Simple_repeat	76	2	X.parietina glXanPari20	Simple_repeat
(TTCGCC)N#Simple_repeat	76	2	X.parietina glXanPari20	Simple_repeat
(TTGAGA)N#Simple_repeat	76	1	X.parietina glXanPari20	Simple_repeat
(TTTCCC)N#Simple_repeat	76	2	X.parietina glXanPari20	Simple_repeat
(AACACC)N#Simple_repeat	75	1	X.parietina glXanPari20	Simple_repeat
(AGAA)N#Simple_repeat	75	3	X.parietina glXanPari20	Simple_repeat
(CACCT)N#Simple_repeat	75	2	X.parietina glXanPari20	Simple_repeat
(CTCCCA)N#Simple_repeat	75	1	X.parietina glXanPari20	Simple_repeat
(CTTCTTT)N#Simple_repeat	75	1	X.parietina glXanPari20	Simple_repeat
(GTCCTC)N#Simple_repeat	75	2	X.parietina glXanPari20	Simple_repeat
(TTATA)N#Simple_repeat	75	1	X.parietina glXanPari20	Simple_repeat
(AACC)N#Simple_repeat	74	3	X.parietina glXanPari20	Simple_repeat
(ATCCCA)N#Simple_repeat	74	1	X.parietina glXanPari20	Simple_repeat
(CGCTAC)N#Simple_repeat	74	1	X.parietina glXanPari20	Simple_repeat
(GAGA)N#Simple_repeat	74	2	X.parietina glXanPari20	Simple_repeat
(TCCACC)N#Simple_repeat	74	2	X.parietina glXanPari20	Simple_repeat
(ACCCCCT)N#Simple_repeat	73	1	X.parietina glXanPari20	Simple_repeat
(ATA)N#Simple_repeat	73	3	X.parietina glXanPari20	Simple_repeat
(CACTCTC)N#Simple_repeat	73	1	X.parietina glXanPari20	Simple_repeat
(GCCGAA)N#Simple_repeat	73	2	X.parietina glXanPari20	Simple_repeat

(GGCGTC)N#Simple_repeat	73	2	X.parietina glXanPari20	Simple_repeat
(GGTGAG)N#Simple_repeat	73	1	X.parietina glXanPari20	Simple_repeat
(TAGAGT)N#Simple_repeat	73	1	X.parietina glXanPari20	Simple_repeat
(TTTTCG)N#Simple_repeat	73	2	X.parietina glXanPari20	Simple_repeat
(AATTCA)N#Simple_repeat	72	1	X.parietina glXanPari20	Simple_repeat
(CCACAT)N#Simple_repeat	72	2	X.parietina glXanPari20	Simple_repeat
(CCCTT)N#Simple_repeat	72	2	X.parietina glXanPari20	Simple_repeat
(CGATGC)N#Simple_repeat	72	2	X.parietina glXanPari20	Simple_repeat
(GAAGATG)N#Simple_repeat	72	1	X.parietina glXanPari20	Simple_repeat
(AAGATC)N#Simple_repeat	71	1	X.parietina glXanPari20	Simple_repeat
(AATATA)N#Simple_repeat	71	1	X.parietina glXanPari20	Simple_repeat
(ACTGCCAAGG)N#Simple_repeat	71	1	X.parietina glXanPari20	Simple_repeat
(CCAACC)N#Simple_repeat	71	1	X.parietina glXanPari20	Simple_repeat
(CCCTCCT)N#Simple_repeat	71	2	X.parietina glXanPari20	Simple_repeat
(CGCCAC)N#Simple_repeat	71	1	X.parietina glXanPari20	Simple_repeat
(GAGGAA)N#Simple_repeat	71	1	X.parietina glXanPari20	Simple_repeat
(TCCAT)N#Simple_repeat	71	2	X.parietina glXanPari20	Simple_repeat
(TCCCCT)N#Simple_repeat	71	2	X.parietina glXanPari20	Simple_repeat
(TGCT)N#Simple_repeat	71	2	X.parietina glXanPari20	Simple_repeat
(ACAGAGATAC)N#Simple_repeat	70	1	X.parietina glXanPari20	Simple_repeat
(ATTTA)N#Simple_repeat	70	2	X.parietina glXanPari20	Simple_repeat
(CAGT)N#Simple_repeat	70	2	X.parietina glXanPari20	Simple_repeat
(GACA)N#Simple_repeat	70	1	X.parietina glXanPari20	Simple_repeat
(GGCGAG)N#Simple_repeat	70	1	X.parietina glXanPari20	Simple_repeat
(GGTGAT)N#Simple_repeat	70	2	X.parietina glXanPari20	Simple_repeat
(GTTGTC)N#Simple_repeat	70	1	X.parietina glXanPari20	Simple_repeat

(TATG)N#Simple_repeat	70	2	X.parietina glXanPari20	Simple_repeat
(TTCTTGC)N#Simple_repeat	70	1	X.parietina glXanPari20	Simple_repeat
(ACACCC)N#Simple_repeat	69	1	X.parietina glXanPari20	Simple_repeat
(AGGTGG)N#Simple_repeat	69	2	X.parietina glXanPari20	Simple_repeat
(ATCATC)N#Simple_repeat	69	1	X.parietina glXanPari20	Simple_repeat
(CGCGGT)N#Simple_repeat	69	1	X.parietina glXanPari20	Simple_repeat
(GAGTCGGA)N#Simple_repeat	69	1	X.parietina glXanPari20	Simple_repeat
(GGAAA)N#Simple_repeat	69	2	X.parietina glXanPari20	Simple_repeat
(GTGAGAGG)N#Simple_repeat	69	1	X.parietina glXanPari20	Simple_repeat
(TCACGC)N#Simple_repeat	69	1	X.parietina glXanPari20	Simple_repeat
(TCTCGC)N#Simple_repeat	69	1	X.parietina glXanPari20	Simple_repeat
(TCTCT)N#Simple_repeat	69	2	X.parietina glXanPari20	Simple_repeat
(ATCTCTTC)N#Simple_repeat	68	2	X.parietina glXanPari20	Simple_repeat
(CAACAT)N#Simple_repeat	68	2	X.parietina glXanPari20	Simple_repeat
(CAGCAAAC)N#Simple_repeat	68	1	X.parietina glXanPari20	Simple_repeat
(CCCTCTC)N#Simple_repeat	68	2	X.parietina glXanPari20	Simple_repeat
(CCGCTC)N#Simple_repeat	68	2	X.parietina glXanPari20	Simple_repeat
(CCGGCAT)N#Simple_repeat	68	1	X.parietina glXanPari20	Simple_repeat
(CGATCCT)N#Simple_repeat	68	1	X.parietina glXanPari20	Simple_repeat
(CGGAGA)N#Simple_repeat	68	1	X.parietina glXanPari20	Simple_repeat
(GAGGAG)N#Simple_repeat	68	1	X.parietina glXanPari20	Simple_repeat
(GAGT)N#Simple_repeat	68	3	X.parietina glXanPari20	Simple_repeat
(GCTGATGAT)N#Simple_repeat	68	1	X.parietina glXanPari20	Simple_repeat
(GGAGAT)N#Simple_repeat	68	1	X.parietina glXanPari20	Simple_repeat
(GGCAGT)N#Simple_repeat	68	1	X.parietina glXanPari20	Simple_repeat
(TCCGCG)N#Simple_repeat	68	2	X.parietina glXanPari20	Simple_repeat

(CGATGG)N#Simple_repeat	67	2	X.parietina glXanPari20	Simple_repeat
(TCTC)N#Simple_repeat	67	1	X.parietina glXanPari20	Simple_repeat
(AGCAAC)N#Simple_repeat	66	2	X.parietina glXanPari20	Simple_repeat
(ATGGGG)N#Simple_repeat	66	2	X.parietina glXanPari20	Simple_repeat
(CCAGAG)N#Simple_repeat	66	2	X.parietina glXanPari20	Simple_repeat
(TGGTG)N#Simple_repeat	66	1	X.parietina glXanPari20	Simple_repeat
(AGAAGT)N#Simple_repeat	65	1	X.parietina glXanPari20	Simple_repeat
(CGATCT)N#Simple_repeat	65	1	X.parietina glXanPari20	Simple_repeat
(GACC)N#Simple_repeat	65	2	X.parietina glXanPari20	Simple_repeat
(GATGGAA)N#Simple_repeat	65	1	X.parietina glXanPari20	Simple_repeat
(GGGATG)N#Simple_repeat	65	2	X.parietina glXanPari20	Simple_repeat
(TATTC)N#Simple_repeat	65	1	X.parietina glXanPari20	Simple_repeat
(TCCCTCC)N#Simple_repeat	65	1	X.parietina glXanPari20	Simple_repeat
(TTCTGC)N#Simple_repeat	65	2	X.parietina glXanPari20	Simple_repeat
(CACCTCC)N#Simple_repeat	64	1	X.parietina glXanPari20	Simple_repeat
(CACCTTC)N#Simple_repeat	64	1	X.parietina glXanPari20	Simple_repeat
(CGAAGC)N#Simple_repeat	64	2	X.parietina glXanPari20	Simple_repeat
(CTGA)N#Simple_repeat	64	1	X.parietina glXanPari20	Simple_repeat
(GGCGTTG)N#Simple_repeat	64	1	X.parietina glXanPari20	Simple_repeat
(TCTTTAC)N#Simple_repeat	64	1	X.parietina glXanPari20	Simple_repeat
(TTACATT)N#Simple_repeat	64	1	X.parietina glXanPari20	Simple_repeat
(TTACCCC)N#Simple_repeat	64	1	X.parietina glXanPari20	Simple_repeat
(AAGGGG)N#Simple_repeat	63	2	X.parietina glXanPari20	Simple_repeat
(AATACC)N#Simple_repeat	63	1	X.parietina glXanPari20	Simple_repeat
(ATAC)N#Simple_repeat	63	2	X.parietina glXanPari20	Simple_repeat
(GGGGGA)N#Simple_repeat	63	2	X.parietina glXanPari20	Simple_repeat

(ACACAA)N#Simple_repeat	62	2	X.parietina glXanPari20	Simple_repeat
(AGCCAG)N#Simple_repeat	62	1	X.parietina glXanPari20	Simple_repeat
(ATCTTT)N#Simple_repeat	62	1	X.parietina glXanPari20	Simple_repeat
(CAACCA)N#Simple_repeat	62	2	X.parietina glXanPari20	Simple_repeat
(CTCCCTC)N#Simple_repeat	62	1	X.parietina glXanPari20	Simple_repeat
(GCAT)N#Simple_repeat	62	2	X.parietina glXanPari20	Simple_repeat
(GCGTCA)N#Simple_repeat	62	2	X.parietina glXanPari20	Simple_repeat
(GGTTT)N#Simple_repeat	62	1	X.parietina glXanPari20	Simple_repeat
(GTACGGA)N#Simple_repeat	62	1	X.parietina glXanPari20	Simple_repeat
(TGGCGTTC)N#Simple_repeat	62	1	X.parietina glXanPari20	Simple_repeat
(TTATTAT)N#Simple_repeat	62	1	X.parietina glXanPari20	Simple_repeat
(TTCGTC)N#Simple_repeat	62	1	X.parietina glXanPari20	Simple_repeat
(ACCAAC)N#Simple_repeat	61	1	X.parietina glXanPari20	Simple_repeat
(AGATGT)N#Simple_repeat	61	2	X.parietina glXanPari20	Simple_repeat
(CAACCTC)N#Simple_repeat	61	1	X.parietina glXanPari20	Simple_repeat
(CCACT)N#Simple_repeat	61	2	X.parietina glXanPari20	Simple_repeat
(CCCAGCA)N#Simple_repeat	61	1	X.parietina glXanPari20	Simple_repeat
(CTCCCCA)N#Simple_repeat	61	1	X.parietina glXanPari20	Simple_repeat
(GCTGGT)N#Simple_repeat	61	1	X.parietina glXanPari20	Simple_repeat
(TCACCATC)N#Simple_repeat	61	1	X.parietina glXanPari20	Simple_repeat
(TGAGG)N#Simple_repeat	61	2	X.parietina glXanPari20	Simple_repeat
(AGAGC)N#Simple_repeat	60	2	X.parietina glXanPari20	Simple_repeat
(CAAC)N#Simple_repeat	60	2	X.parietina glXanPari20	Simple_repeat
(CC)N#Simple_repeat	60	1	X.parietina glXanPari20	Simple_repeat
(CCCCCTC)N#Simple_repeat	60	1	X.parietina glXanPari20	Simple_repeat
(CCTTC)N#Simple_repeat	60	2	X.parietina glXanPari20	Simple_repeat

(CGCCCC)N#Simple_repeat	60	1	X.parietina glXanPari20	Simple_repeat
(CTATT)N#Simple_repeat	60	1	X.parietina glXanPari20	Simple_repeat
(CTCAACC)N#Simple_repeat	60	1	X.parietina glXanPari20	Simple_repeat
(GAATTGT)N#Simple_repeat	60	1	X.parietina glXanPari20	Simple_repeat
(TCAAT)N#Simple_repeat	60	2	X.parietina glXanPari20	Simple_repeat
(TCCACA)N#Simple_repeat	60	1	X.parietina glXanPari20	Simple_repeat
(ATATT)N#Simple_repeat	59	1	X.parietina glXanPari20	Simple_repeat
(ATGAGTG)N#Simple_repeat	59	1	X.parietina glXanPari20	Simple_repeat
(CAAGC)N#Simple_repeat	59	2	X.parietina glXanPari20	Simple_repeat
(CCCATCC)N#Simple_repeat	59	1	X.parietina glXanPari20	Simple_repeat
(CCGCCA)N#Simple_repeat	59	1	X.parietina glXanPari20	Simple_repeat
(CGGCGG)N#Simple_repeat	59	1	X.parietina glXanPari20	Simple_repeat
(GAATG)N#Simple_repeat	59	1	X.parietina glXanPari20	Simple_repeat
(GAGAAGT)N#Simple_repeat	59	1	X.parietina glXanPari20	Simple_repeat
(GAGCAG)N#Simple_repeat	59	1	X.parietina glXanPari20	Simple_repeat
(GCCAAGC)N#Simple_repeat	59	1	X.parietina glXanPari20	Simple_repeat
(TATA)N#Simple_repeat	59	1	X.parietina glXanPari20	Simple_repeat
(TCTTCCTCT)N#Simple_repeat	59	1	X.parietina glXanPari20	Simple_repeat
(TGGT)N#Simple_repeat	59	2	X.parietina glXanPari20	Simple_repeat
(CCACACCA)N#Simple_repeat	58	1	X.parietina glXanPari20	Simple_repeat
(CCCCG)N#Simple_repeat	58	2	X.parietina glXanPari20	Simple_repeat
(CGGAGC)N#Simple_repeat	58	1	X.parietina glXanPari20	Simple_repeat
(CTTTC)N#Simple_repeat	58	2	X.parietina glXanPari20	Simple_repeat
(CTTTCCC)N#Simple_repeat	58	1	X.parietina glXanPari20	Simple_repeat
(GCTCTCT)N#Simple_repeat	58	1	X.parietina glXanPari20	Simple_repeat
(GGAGGTC)N#Simple_repeat	58	1	X.parietina glXanPari20	Simple_repeat

(TATC)N#Simple_repeat	58	1	X.parietina glXanPari20	Simple_repeat
(TCAC)N#Simple_repeat	58	2	X.parietina glXanPari20	Simple_repeat
(TCACTC)N#Simple_repeat	58	1	X.parietina glXanPari20	Simple_repeat
(TGATG)N#Simple_repeat	58	1	X.parietina glXanPari20	Simple_repeat
(AACAACC)N#Simple_repeat	57	1	X.parietina glXanPari20	Simple_repeat
(ATACTAA)N#Simple_repeat	57	1	X.parietina glXanPari20	Simple_repeat
(ATAGA)N#Simple_repeat	57	1	X.parietina glXanPari20	Simple_repeat
(ATTATG)N#Simple_repeat	57	1	X.parietina glXanPari20	Simple_repeat
(CATCCA)N#Simple_repeat	57	1	X.parietina glXanPari20	Simple_repeat
(CCACCC)N#Simple_repeat	57	1	X.parietina glXanPari20	Simple_repeat
(CCAGCA)N#Simple_repeat	57	1	X.parietina glXanPari20	Simple_repeat
(CCTCCAC)N#Simple_repeat	57	1	X.parietina glXanPari20	Simple_repeat
(CTCATC)N#Simple_repeat	57	2	X.parietina glXanPari20	Simple_repeat
(CTCGGC)N#Simple_repeat	57	1	X.parietina glXanPari20	Simple_repeat
(GCACTC)N#Simple_repeat	57	1	X.parietina glXanPari20	Simple_repeat
(TCTCCCT)N#Simple_repeat	57	1	X.parietina glXanPari20	Simple_repeat
(AACGA)N#Simple_repeat	56	2	X.parietina glXanPari20	Simple_repeat
(AAGA)N#Simple_repeat	56	2	X.parietina glXanPari20	Simple_repeat
(AAGCA)N#Simple_repeat	56	1	X.parietina glXanPari20	Simple_repeat
(ACCTC)N#Simple_repeat	56	2	X.parietina glXanPari20	Simple_repeat
(AGCATC)N#Simple_repeat	56	1	X.parietina glXanPari20	Simple_repeat
(CAATC)N#Simple_repeat	56	1	X.parietina glXanPari20	Simple_repeat
(CCCTCAC)N#Simple_repeat	56	1	X.parietina glXanPari20	Simple_repeat
(CGGAGG)N#Simple_repeat	56	1	X.parietina glXanPari20	Simple_repeat
(CGGCTT)N#Simple_repeat	56	2	X.parietina glXanPari20	Simple_repeat
(GAGCA)N#Simple_repeat	56	2	X.parietina glXanPari20	Simple_repeat

(GTGT)N#Simple_repeat	56	2	X.parietina glXanPari20	Simple_repeat
(TTTCCT)N#Simple_repeat	56	1	X.parietina glXanPari20	Simple_repeat
(AAAG)N#Simple_repeat	55	2	X.parietina glXanPari20	Simple_repeat
(ATCAA)N#Simple_repeat	55	2	X.parietina glXanPari20	Simple_repeat
(ATCCCCAGC)N#Simple_repeat	55	1	X.parietina glXanPari20	Simple_repeat
(CCAGCG)N#Simple_repeat	55	1	X.parietina glXanPari20	Simple_repeat
(CTACACT)N#Simple_repeat	55	1	X.parietina glXanPari20	Simple_repeat
(CTCTTTG)N#Simple_repeat	55	1	X.parietina glXanPari20	Simple_repeat
(GACGGAA)N#Simple_repeat	55	1	X.parietina glXanPari20	Simple_repeat
(GGAATG)N#Simple_repeat	55	1	X.parietina glXanPari20	Simple_repeat
(GGAGATG)N#Simple_repeat	55	1	X.parietina glXanPari20	Simple_repeat
(TATGATG)N#Simple_repeat	55	1	X.parietina glXanPari20	Simple_repeat
(TCCTTCC)N#Simple_repeat	55	1	X.parietina glXanPari20	Simple_repeat
(TGGGGA)N#Simple_repeat	55	1	X.parietina glXanPari20	Simple_repeat
(TTAATA)N#Simple_repeat	55	1	X.parietina glXanPari20	Simple_repeat
(ATGATTG)N#Simple_repeat	54	1	X.parietina glXanPari20	Simple_repeat
(CAGCTA)N#Simple_repeat	54	1	X.parietina glXanPari20	Simple_repeat
(CCAACT)N#Simple_repeat	54	1	X.parietina glXanPari20	Simple_repeat
(CTCCTAC)N#Simple_repeat	54	1	X.parietina glXanPari20	Simple_repeat
(CTGCTC)N#Simple_repeat	54	1	X.parietina glXanPari20	Simple_repeat
(CTTTTA)N#Simple_repeat	54	1	X.parietina glXanPari20	Simple_repeat
(GGAAGT)N#Simple_repeat	54	1	X.parietina glXanPari20	Simple_repeat
(GGCAATGA)N#Simple_repeat	54	1	X.parietina glXanPari20	Simple_repeat
(GGCGAC)N#Simple_repeat	54	1	X.parietina glXanPari20	Simple_repeat
(GTGCG)N#Simple_repeat	54	1	X.parietina glXanPari20	Simple_repeat
(TAAAGA)N#Simple_repeat	54	1	X.parietina glXanPari20	Simple_repeat

(TCACAG)N#Simple_repeat	54	1	X.parietina glXanPari20	Simple_repeat
(TCTGT)N#Simple_repeat	54	1	X.parietina glXanPari20	Simple_repeat
(TGAATGA)N#Simple_repeat	54	1	X.parietina glXanPari20	Simple_repeat
(TGACTG)N#Simple_repeat	54	1	X.parietina glXanPari20	Simple_repeat
(CACCTC)N#Simple_repeat	53	1	X.parietina glXanPari20	Simple_repeat
(CCAAG)N#Simple_repeat	53	1	X.parietina glXanPari20	Simple_repeat
(CCACAAG)N#Simple_repeat	53	1	X.parietina glXanPari20	Simple_repeat
(CCTCTCC)N#Simple_repeat	53	1	X.parietina glXanPari20	Simple_repeat
(CGATCG)N#Simple_repeat	53	1	X.parietina glXanPari20	Simple_repeat
(CGCCCCC)N#Simple_repeat	53	1	X.parietina glXanPari20	Simple_repeat
(GAGGTA)N#Simple_repeat	53	1	X.parietina glXanPari20	Simple_repeat
(GATGCAGCA)N#Simple_repeat	53	1	X.parietina glXanPari20	Simple_repeat
(GCGGTG)N#Simple_repeat	53	1	X.parietina glXanPari20	Simple_repeat
(GGCT)N#Simple_repeat	53	2	X.parietina glXanPari20	Simple_repeat
(GGTCCT)N#Simple_repeat	53	1	X.parietina glXanPari20	Simple_repeat
(GGTGCT)N#Simple_repeat	53	1	X.parietina glXanPari20	Simple_repeat
(GTACA)N#Simple_repeat	53	2	X.parietina glXanPari20	Simple_repeat
(TCAGGGAT)N#Simple_repeat	53	1	X.parietina glXanPari20	Simple_repeat
(AACTTCA)N#Simple_repeat	52	1	X.parietina glXanPari20	Simple_repeat
(AAGG)N#Simple_repeat	52	2	X.parietina glXanPari20	Simple_repeat
(AATGCA)N#Simple_repeat	52	1	X.parietina glXanPari20	Simple_repeat
(AGCTCTCC)N#Simple_repeat	52	1	X.parietina glXanPari20	Simple_repeat
(CAACAC)N#Simple_repeat	52	1	X.parietina glXanPari20	Simple_repeat
(CAGATGGGG)N#Simple_repeat	52	1	X.parietina glXanPari20	Simple_repeat
(CCCATA)N#Simple_repeat	52	1	X.parietina glXanPari20	Simple_repeat
(CCTCTTC)N#Simple_repeat	52	1	X.parietina glXanPari20	Simple_repeat

(GATCCA)N#Simple_repeat	52	1	X.parietina glXanPari20	Simple_repeat
(GCACCG)N#Simple_repeat	52	1	X.parietina glXanPari20	Simple_repeat
(GGATGGAAGA)N#Simple_repeat	52	1	X.parietina glXanPari20	Simple_repeat
(GGCAGAA)N#Simple_repeat	52	1	X.parietina glXanPari20	Simple_repeat
(GGTTGG)N#Simple_repeat	52	1	X.parietina glXanPari20	Simple_repeat
(TAGAC)N#Simple_repeat	52	1	X.parietina glXanPari20	Simple_repeat
(TATACTA)N#Simple_repeat	52	1	X.parietina glXanPari20	Simple_repeat
(TCGACCTCC)N#Simple_repeat	52	1	X.parietina glXanPari20	Simple_repeat
(TGCTTGG)N#Simple_repeat	52	1	X.parietina glXanPari20	Simple_repeat
(TGGTT)N#Simple_repeat	52	1	X.parietina glXanPari20	Simple_repeat
(AAGATGA)N#Simple_repeat	51	1	X.parietina glXanPari20	Simple_repeat
(ACCGCC)N#Simple_repeat	51	1	X.parietina glXanPari20	Simple_repeat
(AGTTGATG)N#Simple_repeat	51	1	X.parietina glXanPari20	Simple_repeat
(ATTTTA)N#Simple_repeat	51	1	X.parietina glXanPari20	Simple_repeat
(CCAA)N#Simple_repeat	51	2	X.parietina glXanPari20	Simple_repeat
(CCTCTCT)N#Simple_repeat	51	1	X.parietina glXanPari20	Simple_repeat
(CGACCAAG)N#Simple_repeat	51	1	X.parietina glXanPari20	Simple_repeat
(CGCAGC)N#Simple_repeat	51	1	X.parietina glXanPari20	Simple_repeat
(GAAA)N#Simple_repeat	51	2	X.parietina glXanPari20	Simple_repeat
(GAAT)N#Simple_repeat	51	2	X.parietina glXanPari20	Simple_repeat
(GAGAAGCTAT)N#Simple_repeat	51	1	X.parietina glXanPari20	Simple_repeat
(GAGGCC)N#Simple_repeat	51	1	X.parietina glXanPari20	Simple_repeat
(GGTGGTT)N#Simple_repeat	51	1	X.parietina glXanPari20	Simple_repeat
(GTTGTG)N#Simple_repeat	51	1	X.parietina glXanPari20	Simple_repeat
(TCCCTTCC)N#Simple_repeat	51	1	X.parietina glXanPari20	Simple_repeat
(TGTTGC)N#Simple_repeat	51	1	X.parietina glXanPari20	Simple_repeat

(TTAA)N#Simple_repeat	51	1	X.parietina glXanPari20	Simple_repeat
(TTATAA)N#Simple_repeat	51	1	X.parietina glXanPari20	Simple_repeat
(AACCAT)N#Simple_repeat	50	1	X.parietina glXanPari20	Simple_repeat
(AAGTG)N#Simple_repeat	50	1	X.parietina glXanPari20	Simple_repeat
(ACACTCA)N#Simple_repeat	50	1	X.parietina glXanPari20	Simple_repeat
(ACAT)N#Simple_repeat	50	1	X.parietina glXanPari20	Simple_repeat
(AGGGAAG)N#Simple_repeat	50	1	X.parietina glXanPari20	Simple_repeat
(CCTTT)N#Simple_repeat	50	1	X.parietina glXanPari20	Simple_repeat
(CTCCACC)N#Simple_repeat	50	1	X.parietina glXanPari20	Simple_repeat
(CTTTCCT)N#Simple_repeat	50	1	X.parietina glXanPari20	Simple_repeat
(GCGGT)N#Simple_repeat	50	1	X.parietina glXanPari20	Simple_repeat
(GGAGA)N#Simple_repeat	50	2	X.parietina glXanPari20	Simple_repeat
(GGCGGTG)N#Simple_repeat	50	1	X.parietina glXanPari20	Simple_repeat
(GTGGATCGT)N#Simple_repeat	50	1	X.parietina glXanPari20	Simple_repeat
(TGCA)N#Simple_repeat	50	2	X.parietina glXanPari20	Simple_repeat
(TGTT)N#Simple_repeat	50	2	X.parietina glXanPari20	Simple_repeat
(TTTTATA)N#Simple_repeat	50	1	X.parietina glXanPari20	Simple_repeat
(ATGT)N#Simple_repeat	49	2	X.parietina glXanPari20	Simple_repeat
(ATTTAT)N#Simple_repeat	49	1	X.parietina glXanPari20	Simple_repeat
(CCCAACA)N#Simple_repeat	49	1	X.parietina glXanPari20	Simple_repeat
(CCCCTCC)N#Simple_repeat	49	1	X.parietina glXanPari20	Simple_repeat
(CGGCGA)N#Simple_repeat	49	1	X.parietina glXanPari20	Simple_repeat
(CGTCCTTTGG)N#Simple_repeat	49	1	X.parietina glXanPari20	Simple_repeat
(CTGCTGG)N#Simple_repeat	49	1	X.parietina glXanPari20	Simple_repeat
(GGTGTGG)N#Simple_repeat	49	1	X.parietina glXanPari20	Simple_repeat
(GGTTG)N#Simple_repeat	49	1	X.parietina glXanPari20	Simple_repeat

(TCATCCC)N#Simple_repeat	49	1	X.parietina glXanPari20	Simple_repeat
(TCTCCG)N#Simple_repeat	49	1	X.parietina glXanPari20	Simple_repeat
(TGTCTC)N#Simple_repeat	49	1	X.parietina glXanPari20	Simple_repeat
(AAGGGAA)N#Simple_repeat	48	1	X.parietina glXanPari20	Simple_repeat
(AAGTGG)N#Simple_repeat	48	1	X.parietina glXanPari20	Simple_repeat
(ACCTCTC)N#Simple_repeat	48	1	X.parietina glXanPari20	Simple_repeat
(AGAG)N#Simple_repeat	48	1	X.parietina glXanPari20	Simple_repeat
(ATGATGT)N#Simple_repeat	48	1	X.parietina glXanPari20	Simple_repeat
(CCCTCCC)N#Simple_repeat	48	1	X.parietina glXanPari20	Simple_repeat
(CCTCCTA)N#Simple_repeat	48	1	X.parietina glXanPari20	Simple_repeat
(CGCTCGT)N#Simple_repeat	48	1	X.parietina glXanPari20	Simple_repeat
(CTCGTCT)N#Simple_repeat	48	1	X.parietina glXanPari20	Simple_repeat
(CTGCTT)N#Simple_repeat	48	1	X.parietina glXanPari20	Simple_repeat
(CTTCTT)N#Simple_repeat	48	1	X.parietina glXanPari20	Simple_repeat
(GAGAGCA)N#Simple_repeat	48	1	X.parietina glXanPari20	Simple_repeat
(GCTGAG)N#Simple_repeat	48	1	X.parietina glXanPari20	Simple_repeat
(GTAGAT)N#Simple_repeat	48	1	X.parietina glXanPari20	Simple_repeat
(GTTTTT)N#Simple_repeat	48	1	X.parietina glXanPari20	Simple_repeat
(TCGCA)N#Simple_repeat	48	1	X.parietina glXanPari20	Simple_repeat
(TCTCCT)N#Simple_repeat	48	1	X.parietina glXanPari20	Simple_repeat
(TGAGGC)N#Simple_repeat	48	1	X.parietina glXanPari20	Simple_repeat
(TTATCT)N#Simple_repeat	48	1	X.parietina glXanPari20	Simple_repeat
(ACGAA)N#Simple_repeat	47	1	X.parietina glXanPari20	Simple_repeat
(ACGCCA)N#Simple_repeat	47	1	X.parietina glXanPari20	Simple_repeat
(AGTACTC)N#Simple_repeat	47	1	X.parietina glXanPari20	Simple_repeat
(ATACAT)N#Simple_repeat	47	1	X.parietina glXanPari20	Simple_repeat

(ATGATGC)N#Simple_repeat	47	1	X.parietina glXanPari20	Simple_repeat
(ATTCTC)N#Simple_repeat	47	1	X.parietina glXanPari20	Simple_repeat
(CAACCTGC)N#Simple_repeat	47	1	X.parietina glXanPari20	Simple_repeat
(CAAG)N#Simple_repeat	47	1	X.parietina glXanPari20	Simple_repeat
(CATCTG)N#Simple_repeat	47	1	X.parietina glXanPari20	Simple_repeat
(CCCATT)N#Simple_repeat	47	1	X.parietina glXanPari20	Simple_repeat
(CCTCCTC)N#Simple_repeat	47	1	X.parietina glXanPari20	Simple_repeat
(CCTT)N#Simple_repeat	47	1	X.parietina glXanPari20	Simple_repeat
(GCGAGAG)N#Simple_repeat	47	1	X.parietina glXanPari20	Simple_repeat
(GGCATGGGA)N#Simple_repeat	47	1	X.parietina glXanPari20	Simple_repeat
(TCTCTT)N#Simple_repeat	47	1	X.parietina glXanPari20	Simple_repeat
(TGCTCC)N#Simple_repeat	47	1	X.parietina glXanPari20	Simple_repeat
(TGGCGG)N#Simple_repeat	47	1	X.parietina glXanPari20	Simple_repeat
(CACGA)N#Simple_repeat	46	1	X.parietina glXanPari20	Simple_repeat
(CCCC)N#Simple_repeat	46	1	X.parietina glXanPari20	Simple_repeat
(CCGCAG)N#Simple_repeat	46	1	X.parietina glXanPari20	Simple_repeat
(CGGAAGG)N#Simple_repeat	46	1	X.parietina glXanPari20	Simple_repeat
(CTGCAGA)N#Simple_repeat	46	1	X.parietina glXanPari20	Simple_repeat
(CTGG)N#Simple_repeat	46	2	X.parietina glXanPari20	Simple_repeat
(GCCTCTG)N#Simple_repeat	46	1	X.parietina glXanPari20	Simple_repeat
(GCGGCT)N#Simple_repeat	46	1	X.parietina glXanPari20	Simple_repeat
(GGACGT)N#Simple_repeat	46	1	X.parietina glXanPari20	Simple_repeat
(GGCAAC)N#Simple_repeat	46	1	X.parietina glXanPari20	Simple_repeat
(GTGTGG)N#Simple_repeat	46	1	X.parietina glXanPari20	Simple_repeat
(TAGTA)N#Simple_repeat	46	1	X.parietina glXanPari20	Simple_repeat
(TCCAGCC)N#Simple_repeat	46	1	X.parietina glXanPari20	Simple_repeat

(TCCCAT)N#Simple_repeat	46	1	X.parietina glXanPari20	Simple_repeat
(TGGTAGTGC)N#Simple_repeat	46	1	X.parietina glXanPari20	Simple_repeat
(TTCTTC)N#Simple_repeat	46	1	X.parietina glXanPari20	Simple_repeat
(AAGATAC)N#Simple_repeat	45	1	X.parietina glXanPari20	Simple_repeat
(AAGGTAG)N#Simple_repeat	45	1	X.parietina glXanPari20	Simple_repeat
(ACCACAA)N#Simple_repeat	45	1	X.parietina glXanPari20	Simple_repeat
(ACGGTC)N#Simple_repeat	45	1	X.parietina glXanPari20	Simple_repeat
(ACGGTG)N#Simple_repeat	45	1	X.parietina glXanPari20	Simple_repeat
(AGAGCA)N#Simple_repeat	45	1	X.parietina glXanPari20	Simple_repeat
(AGGACG)N#Simple_repeat	45	1	X.parietina glXanPari20	Simple_repeat
(ATCCA)N#Simple_repeat	45	1	X.parietina glXanPari20	Simple_repeat
(ATCCTCG)N#Simple_repeat	45	1	X.parietina glXanPari20	Simple_repeat
(CATCGAG)N#Simple_repeat	45	1	X.parietina glXanPari20	Simple_repeat
(CATTTC)N#Simple_repeat	45	1	X.parietina glXanPari20	Simple_repeat
(CCGCCGC)N#Simple_repeat	45	1	X.parietina glXanPari20	Simple_repeat
(CCTGAAC)N#Simple_repeat	45	1	X.parietina glXanPari20	Simple_repeat
(CCTTCAT)N#Simple_repeat	45	1	X.parietina glXanPari20	Simple_repeat
(CTCGAGG)N#Simple_repeat	45	1	X.parietina glXanPari20	Simple_repeat
(GAGGGGGAG)N#Simple_repeat	45	1	X.parietina glXanPari20	Simple_repeat
(GATGAT)N#Simple_repeat	45	1	X.parietina glXanPari20	Simple_repeat
(GATGG)N#Simple_repeat	45	1	X.parietina glXanPari20	Simple_repeat
(GCTGTG)N#Simple_repeat	45	1	X.parietina glXanPari20	Simple_repeat
(GGTGTA)N#Simple_repeat	45	1	X.parietina glXanPari20	Simple_repeat
(TCTGGTG)N#Simple_repeat	45	1	X.parietina glXanPari20	Simple_repeat
(TGGTGC)N#Simple_repeat	45	1	X.parietina glXanPari20	Simple_repeat
(ACCATCATT)N#Simple_repeat	44	1	X.parietina glXanPari20	Simple_repeat

(AGAT)N#Simple_repeat	44	1	X.parietina glXanPari20	Simple_repeat
(AGCTGA)N#Simple_repeat	44	1	X.parietina glXanPari20	Simple_repeat
(AGTAGTC)N#Simple_repeat	44	1	X.parietina glXanPari20	Simple_repeat
(ATAA)N#Simple_repeat	44	1	X.parietina glXanPari20	Simple_repeat
(ATCTC)N#Simple_repeat	44	1	X.parietina glXanPari20	Simple_repeat
(ATCTGGC)N#Simple_repeat	44	1	X.parietina glXanPari20	Simple_repeat
(ATTTAAG)N#Simple_repeat	44	1	X.parietina glXanPari20	Simple_repeat
(CAGCCAG)N#Simple_repeat	44	1	X.parietina glXanPari20	Simple_repeat
(CATCCCA)N#Simple_repeat	44	1	X.parietina glXanPari20	Simple_repeat
(CATCCTC)N#Simple_repeat	44	1	X.parietina glXanPari20	Simple_repeat
(CCCTTT)N#Simple_repeat	44	1	X.parietina glXanPari20	Simple_repeat
(CCGCACC)N#Simple_repeat	44	1	X.parietina glXanPari20	Simple_repeat
(CGCCGA)N#Simple_repeat	44	1	X.parietina glXanPari20	Simple_repeat
(CTCCCAT)N#Simple_repeat	44	1	X.parietina glXanPari20	Simple_repeat
(CTCGGT)N#Simple_repeat	44	1	X.parietina glXanPari20	Simple_repeat
(CTTCCTT)N#Simple_repeat	44	1	X.parietina glXanPari20	Simple_repeat
(GATTG)N#Simple_repeat	44	1	X.parietina glXanPari20	Simple_repeat
(GATTGCA)N#Simple_repeat	44	1	X.parietina glXanPari20	Simple_repeat
(GGCTGTAG)N#Simple_repeat	44	1	X.parietina glXanPari20	Simple_repeat
(GTGGGT)N#Simple_repeat	44	1	X.parietina glXanPari20	Simple_repeat
(TGCTGTC)N#Simple_repeat	44	1	X.parietina glXanPari20	Simple_repeat
(TGGTGT)N#Simple_repeat	44	1	X.parietina glXanPari20	Simple_repeat
(TTGCCG)N#Simple_repeat	44	1	X.parietina glXanPari20	Simple_repeat
(TTTGTG)N#Simple_repeat	44	1	X.parietina glXanPari20	Simple_repeat
(TTTTCT)N#Simple_repeat	44	1	X.parietina glXanPari20	Simple_repeat
(AAGTGAG)N#Simple_repeat	43	1	X.parietina glXanPari20	Simple_repeat

(ACCCCG)N#Simple_repeat	43	1	X.parietina glXanPari20	Simple_repeat
(AGACGG)N#Simple_repeat	43	1	X.parietina glXanPari20	Simple_repeat
(AGGCGC)N#Simple_repeat	43	1	X.parietina glXanPari20	Simple_repeat
(AGGTATATA)N#Simple_repeat	43	1	X.parietina glXanPari20	Simple_repeat
(AGTGTAG)N#Simple_repeat	43	1	X.parietina glXanPari20	Simple_repeat
(CACCAG)N#Simple_repeat	43	1	X.parietina glXanPari20	Simple_repeat
(CACCCTCAA)N#Simple_repeat	43	1	X.parietina glXanPari20	Simple_repeat
(CAGC)N#Simple_repeat	43	2	X.parietina glXanPari20	Simple_repeat
(CCATCCC)N#Simple_repeat	43	1	X.parietina glXanPari20	Simple_repeat
(CCTCGA)N#Simple_repeat	43	1	X.parietina glXanPari20	Simple_repeat
(CTGGTG)N#Simple_repeat	43	1	X.parietina glXanPari20	Simple_repeat
(CTTCTCTT)N#Simple_repeat	43	1	X.parietina glXanPari20	Simple_repeat
(CTTGAG)N#Simple_repeat	43	1	X.parietina glXanPari20	Simple_repeat
(CTTTGCG)N#Simple_repeat	43	1	X.parietina glXanPari20	Simple_repeat
(GAACA)N#Simple_repeat	43	1	X.parietina glXanPari20	Simple_repeat
(GAATCC)N#Simple_repeat	43	1	X.parietina glXanPari20	Simple_repeat
(GATGATGAC)N#Simple_repeat	43	1	X.parietina glXanPari20	Simple_repeat
(GATGCC)N#Simple_repeat	43	1	X.parietina glXanPari20	Simple_repeat
(GATGCT)N#Simple_repeat	43	1	X.parietina glXanPari20	Simple_repeat
(GCTCCCAGCA)N#Simple_repeat	43	1	X.parietina glXanPari20	Simple_repeat
(GGCTTGT)N#Simple_repeat	43	1	X.parietina glXanPari20	Simple_repeat
(GGTTCG)N#Simple_repeat	43	1	X.parietina glXanPari20	Simple_repeat
(GGTTGTT)N#Simple_repeat	43	1	X.parietina glXanPari20	Simple_repeat
(GTAGCG)N#Simple_repeat	43	1	X.parietina glXanPari20	Simple_repeat
(GTGGAG)N#Simple_repeat	43	1	X.parietina glXanPari20	Simple_repeat
(TATCTAC)N#Simple_repeat	43	1	X.parietina glXanPari20	Simple_repeat

(TCCCAAG)N#Simple_repeat	43	1	X.parietina glXanPari20	Simple_repeat
(TCGCTC)N#Simple_repeat	43	1	X.parietina glXanPari20	Simple_repeat
(TGACCT)N#Simple_repeat	43	1	X.parietina glXanPari20	Simple_repeat
(TGATTT)N#Simple_repeat	43	1	X.parietina glXanPari20	Simple_repeat
(TGGCGT)N#Simple_repeat	43	1	X.parietina glXanPari20	Simple_repeat
(AACCCCTG)N#Simple_repeat	42	1	X.parietina glXanPari20	Simple_repeat
(AATGG)N#Simple_repeat	42	1	X.parietina glXanPari20	Simple_repeat
(ACTATAT)N#Simple_repeat	42	1	X.parietina glXanPari20	Simple_repeat
(ACTCGG)N#Simple_repeat	42	1	X.parietina glXanPari20	Simple_repeat
(AGAGGT)N#Simple_repeat	42	1	X.parietina glXanPari20	Simple_repeat
(ATAATT)N#Simple_repeat	42	1	X.parietina glXanPari20	Simple_repeat
(ATCCGA)N#Simple_repeat	42	1	X.parietina glXanPari20	Simple_repeat
(ATCCTCA)N#Simple_repeat	42	1	X.parietina glXanPari20	Simple_repeat
(CAACCG)N#Simple_repeat	42	1	X.parietina glXanPari20	Simple_repeat
(CAGACAG)N#Simple_repeat	42	1	X.parietina glXanPari20	Simple_repeat
(CCAATC)N#Simple_repeat	42	1	X.parietina glXanPari20	Simple_repeat
(CCCGTC)N#Simple_repeat	42	1	X.parietina glXanPari20	Simple_repeat
(CCGACT)N#Simple_repeat	42	1	X.parietina glXanPari20	Simple_repeat
(CGGAGGT)N#Simple_repeat	42	1	X.parietina glXanPari20	Simple_repeat
(CTTGCA)N#Simple_repeat	42	1	X.parietina glXanPari20	Simple_repeat
(CTTTCC)N#Simple_repeat	42	1	X.parietina glXanPari20	Simple_repeat
(GAGC)N#Simple_repeat	42	1	X.parietina glXanPari20	Simple_repeat
(GAGCCA)N#Simple_repeat	42	1	X.parietina glXanPari20	Simple_repeat
(GATCC)N#Simple_repeat	42	1	X.parietina glXanPari20	Simple_repeat
(GCATCA)N#Simple_repeat	42	1	X.parietina glXanPari20	Simple_repeat
(GCATGG)N#Simple_repeat	42	1	X.parietina glXanPari20	Simple_repeat

(GCCAAC)N#Simple_repeat	42	1	X.parietina glXanPari20	Simple_repeat
(GGAAGAT)N#Simple_repeat	42	1	X.parietina glXanPari20	Simple_repeat
(TATCCCA)N#Simple_repeat	42	1	X.parietina glXanPari20	Simple_repeat
(TCCCACC)N#Simple_repeat	42	1	X.parietina glXanPari20	Simple_repeat
(TCGTCAGAT)N#Simple_repeat	42	1	X.parietina glXanPari20	Simple_repeat
(TCTACCC)N#Simple_repeat	42	1	X.parietina glXanPari20	Simple_repeat
(TCTCTTT)N#Simple_repeat	42	1	X.parietina glXanPari20	Simple_repeat
(TGGTTACGG)N#Simple_repeat	42	1	X.parietina glXanPari20	Simple_repeat
(TTCACT)N#Simple_repeat	42	1	X.parietina glXanPari20	Simple_repeat
(TTCGTGG)N#Simple_repeat	42	1	X.parietina glXanPari20	Simple_repeat
(TTTCGACT)N#Simple_repeat	42	1	X.parietina glXanPari20	Simple_repeat
(TTTTTCC)N#Simple_repeat	42	1	X.parietina glXanPari20	Simple_repeat
(AATAGG)N#Simple_repeat	41	1	X.parietina glXanPari20	Simple_repeat
(AGATAGT)N#Simple_repeat	41	1	X.parietina glXanPari20	Simple_repeat
(AGGGACAGGG)N#Simple_repeat	41	1	X.parietina glXanPari20	Simple_repeat
(ATCTGCA)N#Simple_repeat	41	1	X.parietina glXanPari20	Simple_repeat
(ATGGA)N#Simple_repeat	41	1	X.parietina glXanPari20	Simple_repeat
(CACCGA)N#Simple_repeat	41	1	X.parietina glXanPari20	Simple_repeat
(CATTGA)N#Simple_repeat	41	1	X.parietina glXanPari20	Simple_repeat
(CCAACAC)N#Simple_repeat	41	1	X.parietina glXanPari20	Simple_repeat
(CCCCCCA)N#Simple_repeat	41	1	X.parietina glXanPari20	Simple_repeat
(CCCCTTT)N#Simple_repeat	41	1	X.parietina glXanPari20	Simple_repeat
(CCCGGC)N#Simple_repeat	41	1	X.parietina glXanPari20	Simple_repeat
(CGAGGA)N#Simple_repeat	41	1	X.parietina glXanPari20	Simple_repeat
(CGTCTT)N#Simple_repeat	41	1	X.parietina glXanPari20	Simple_repeat
(CTATACTAG)N#Simple_repeat	41	1	X.parietina glXanPari20	Simple_repeat

(CTTCT)N#Simple_repeat	41	1	X.parietina glXanPari20	Simple_repeat
(GAAGGC)N#Simple_repeat	41	1	X.parietina glXanPari20	Simple_repeat
(GACGAG)N#Simple_repeat	41	1	X.parietina glXanPari20	Simple_repeat
(GACGGC)N#Simple_repeat	41	1	X.parietina glXanPari20	Simple_repeat
(GAGAACT)N#Simple_repeat	41	1	X.parietina glXanPari20	Simple_repeat
(GAGCGGA)N#Simple_repeat	41	1	X.parietina glXanPari20	Simple_repeat
(GCCCCTGA)N#Simple_repeat	41	1	X.parietina glXanPari20	Simple_repeat
(GGACCA)N#Simple_repeat	41	1	X.parietina glXanPari20	Simple_repeat
(GGATTC)N#Simple_repeat	41	1	X.parietina glXanPari20	Simple_repeat
(GGCTTC)N#Simple_repeat	41	1	X.parietina glXanPari20	Simple_repeat
(GGGAAGA)N#Simple_repeat	41	1	X.parietina glXanPari20	Simple_repeat
(GTCCAGT)N#Simple_repeat	41	1	X.parietina glXanPari20	Simple_repeat
(GTTGGAGA)N#Simple_repeat	41	1	X.parietina glXanPari20	Simple_repeat
(GTTGGTCTG)N#Simple_repeat	41	1	X.parietina glXanPari20	Simple_repeat
(TATATAC)N#Simple_repeat	41	1	X.parietina glXanPari20	Simple_repeat
(TATCAA)N#Simple_repeat	41	1	X.parietina glXanPari20	Simple_repeat
(TCCACG)N#Simple_repeat	41	1	X.parietina glXanPari20	Simple_repeat
(TCCAGAC)N#Simple_repeat	41	1	X.parietina glXanPari20	Simple_repeat
(TCTTTC)N#Simple_repeat	41	1	X.parietina glXanPari20	Simple_repeat
(TGAGA)N#Simple_repeat	41	1	X.parietina glXanPari20	Simple_repeat
(TTATATT)N#Simple_repeat	41	1	X.parietina glXanPari20	Simple_repeat
(TTTGGCT)N#Simple_repeat	41	1	X.parietina glXanPari20	Simple_repeat
(ACAATG)N#Simple_repeat	40	1	X.parietina glXanPari20	Simple_repeat
(ACACAC)N#Simple_repeat	40	1	X.parietina glXanPari20	Simple_repeat
(ACACC)N#Simple_repeat	40	1	X.parietina glXanPari20	Simple_repeat
(AGAATGA)N#Simple_repeat	40	1	X.parietina glXanPari20	Simple_repeat

(ATACGGG)N#Simple_repeat	40	1	X.parietina glXanPari20	Simple_repeat
(ATCCAC)N#Simple_repeat	40	1	X.parietina glXanPari20	Simple_repeat
(ATCCTGC)N#Simple_repeat	40	1	X.parietina glXanPari20	Simple_repeat
(CACC)N#Simple_repeat	40	1	X.parietina glXanPari20	Simple_repeat
(CACCAA)N#Simple_repeat	40	1	X.parietina glXanPari20	Simple_repeat
(CACCACACAC)N#Simple_repeat	40	1	X.parietina glXanPari20	Simple_repeat
(CACTCAT)N#Simple_repeat	40	1	X.parietina glXanPari20	Simple_repeat
(CAGCACT)N#Simple_repeat	40	1	X.parietina glXanPari20	Simple_repeat
(CCAACCC)N#Simple_repeat	40	1	X.parietina glXanPari20	Simple_repeat
(CCACCTCCG)N#Simple_repeat	40	1	X.parietina glXanPari20	Simple_repeat
(CCAGAC)N#Simple_repeat	40	1	X.parietina glXanPari20	Simple_repeat
(CCTCAA)N#Simple_repeat	40	1	X.parietina glXanPari20	Simple_repeat
(CCTTCGC)N#Simple_repeat	40	1	X.parietina glXanPari20	Simple_repeat
(CGAGAT)N#Simple_repeat	40	1	X.parietina glXanPari20	Simple_repeat
(CGATCTGCTT)N#Simple_repeat	40	1	X.parietina glXanPari20	Simple_repeat
(CTCCTTC)N#Simple_repeat	40	1	X.parietina glXanPari20	Simple_repeat
(GATCTC)N#Simple_repeat	40	1	X.parietina glXanPari20	Simple_repeat
(GCCTGGAT)N#Simple_repeat	40	1	X.parietina glXanPari20	Simple_repeat
(GCCTTC)N#Simple_repeat	40	1	X.parietina glXanPari20	Simple_repeat
(GCTTCT)N#Simple_repeat	40	1	X.parietina glXanPari20	Simple_repeat
(GCTTTT)N#Simple_repeat	40	1	X.parietina glXanPari20	Simple_repeat
(GGTGTTG)N#Simple_repeat	40	1	X.parietina glXanPari20	Simple_repeat
(GTTGGCG)N#Simple_repeat	40	1	X.parietina glXanPari20	Simple_repeat
(GTTGGTGGTG)N#Simple_repeat	40	1	X.parietina glXanPari20	Simple_repeat
(TACTG)N#Simple_repeat	40	1	X.parietina glXanPari20	Simple_repeat
(TCATCATCG)N#Simple_repeat	40	1	X.parietina glXanPari20	Simple_repeat

(TCCGCC)N#Simple_repeat	40	1	X.parietina glXanPari20	Simple_repeat
(TCCTTCT)N#Simple_repeat	40	1	X.parietina glXanPari20	Simple_repeat
(TCGCCT)N#Simple_repeat	40	1	X.parietina glXanPari20	Simple_repeat
(TCGTCTATC)N#Simple_repeat	40	1	X.parietina glXanPari20	Simple_repeat
(TGCACC)N#Simple_repeat	40	1	X.parietina glXanPari20	Simple_repeat
(TTGGT)N#Simple_repeat	40	1	X.parietina glXanPari20	Simple_repeat
(TTTGCTT)N#Simple_repeat	40	1	X.parietina glXanPari20	Simple_repeat
(TTTTA)N#Simple_repeat	40	1	X.parietina glXanPari20	Simple_repeat
(AACAGC)N#Simple_repeat	39	1	X.parietina glXanPari20	Simple_repeat
(AACCTC)N#Simple_repeat	39	1	X.parietina glXanPari20	Simple_repeat
(AATCAACG)N#Simple_repeat	39	1	X.parietina glXanPari20	Simple_repeat
(AATTTA)N#Simple_repeat	39	1	X.parietina glXanPari20	Simple_repeat
(ACATCC)N#Simple_repeat	39	1	X.parietina glXanPari20	Simple_repeat
(ACATCTG)N#Simple_repeat	39	1	X.parietina glXanPari20	Simple_repeat
(AGGAGA)N#Simple_repeat	39	1	X.parietina glXanPari20	Simple_repeat
(ATCTCAA)N#Simple_repeat	39	1	X.parietina glXanPari20	Simple_repeat
(CAACC)N#Simple_repeat	39	1	X.parietina glXanPari20	Simple_repeat
(CACAAC)N#Simple_repeat	39	1	X.parietina glXanPari20	Simple_repeat
(CAGAAG)N#Simple_repeat	39	1	X.parietina glXanPari20	Simple_repeat
(CAGCGG)N#Simple_repeat	39	1	X.parietina glXanPari20	Simple_repeat
(CCACTCCT)N#Simple_repeat	39	1	X.parietina glXanPari20	Simple_repeat
(CCAGCTC)N#Simple_repeat	39	1	X.parietina glXanPari20	Simple_repeat
(CCATTC)N#Simple_repeat	39	1	X.parietina glXanPari20	Simple_repeat
(CTCCCCT)N#Simple_repeat	39	1	X.parietina glXanPari20	Simple_repeat
(CTCGTTGCT)N#Simple_repeat	39	1	X.parietina glXanPari20	Simple_repeat
(CTGTGT)N#Simple_repeat	39	1	X.parietina glXanPari20	Simple_repeat

(CTTCGCT)N#Simple_repeat	39	1	X.parietina glXanPari20	Simple_repeat
(GAGCATTA)N#Simple_repeat	39	1	X.parietina glXanPari20	Simple_repeat
(GATAGT)N#Simple_repeat	39	1	X.parietina glXanPari20	Simple_repeat
(GCTGT)N#Simple_repeat	39	1	X.parietina glXanPari20	Simple_repeat
(GGAGTGG)N#Simple_repeat	39	1	X.parietina glXanPari20	Simple_repeat
(GGCAG)N#Simple_repeat	39	1	X.parietina glXanPari20	Simple_repeat
(GGGAGAGA)N#Simple_repeat	39	1	X.parietina glXanPari20	Simple_repeat
(GGTTGAG)N#Simple_repeat	39	1	X.parietina glXanPari20	Simple_repeat
(GTCTTCC)N#Simple_repeat	39	1	X.parietina glXanPari20	Simple_repeat
(TATT)N#Simple_repeat	39	1	X.parietina glXanPari20	Simple_repeat
(TCACTCA)N#Simple_repeat	39	1	X.parietina glXanPari20	Simple_repeat
(TCAGCAG)N#Simple_repeat	39	1	X.parietina glXanPari20	Simple_repeat
(TCTAA)N#Simple_repeat	39	1	X.parietina glXanPari20	Simple_repeat
(TCTG)N#Simple_repeat	39	1	X.parietina glXanPari20	Simple_repeat
(TCTGCTC)N#Simple_repeat	39	1	X.parietina glXanPari20	Simple_repeat
(TGCGATC)N#Simple_repeat	39	1	X.parietina glXanPari20	Simple_repeat
(TGCTGAC)N#Simple_repeat	39	1	X.parietina glXanPari20	Simple_repeat
(TTCATTC)N#Simple_repeat	39	1	X.parietina glXanPari20	Simple_repeat
(TTGCTC)N#Simple_repeat	39	1	X.parietina glXanPari20	Simple_repeat
(TTGTCGT)N#Simple_repeat	39	1	X.parietina glXanPari20	Simple_repeat
(TTTGAGA)N#Simple_repeat	39	1	X.parietina glXanPari20	Simple_repeat
(AATTCAC)N#Simple_repeat	38	1	X.parietina glXanPari20	Simple_repeat
(ACAGCA)N#Simple_repeat	38	1	X.parietina glXanPari20	Simple_repeat
(ACTC)N#Simple_repeat	38	1	X.parietina glXanPari20	Simple_repeat
(AGATGTT)N#Simple_repeat	38	1	X.parietina glXanPari20	Simple_repeat
(AGGGTGG)N#Simple_repeat	38	1	X.parietina glXanPari20	Simple_repeat

(ATAGCG)N#Simple_repeat	38	1	X.parietina glXanPari20	Simple_repeat
(ATGGC)N#Simple_repeat	38	1	X.parietina glXanPari20	Simple_repeat
(ATGTCC)N#Simple_repeat	38	1	X.parietina glXanPari20	Simple_repeat
(ATTGGC)N#Simple_repeat	38	1	X.parietina glXanPari20	Simple_repeat
(CACCCCC)N#Simple_repeat	38	1	X.parietina glXanPari20	Simple_repeat
(CATCCAC)N#Simple_repeat	38	1	X.parietina glXanPari20	Simple_repeat
(CCCCACA)N#Simple_repeat	38	1	X.parietina glXanPari20	Simple_repeat
(CCGGCG)N#Simple_repeat	38	1	X.parietina glXanPari20	Simple_repeat
(CCGTATA)N#Simple_repeat	38	1	X.parietina glXanPari20	Simple_repeat
(CGGAAG)N#Simple_repeat	38	1	X.parietina glXanPari20	Simple_repeat
(CGTCGC)N#Simple_repeat	38	1	X.parietina glXanPari20	Simple_repeat
(GAAGCG)N#Simple_repeat	38	1	X.parietina glXanPari20	Simple_repeat
(GAATCA)N#Simple_repeat	38	1	X.parietina glXanPari20	Simple_repeat
(GATGTG)N#Simple_repeat	38	1	X.parietina glXanPari20	Simple_repeat
(GCCAAGAT)N#Simple_repeat	38	1	X.parietina glXanPari20	Simple_repeat
(GCGGCG)N#Simple_repeat	38	1	X.parietina glXanPari20	Simple_repeat
(GGAGG)N#Simple_repeat	38	1	X.parietina glXanPari20	Simple_repeat
(GGGAAAGGA)N#Simple_repeat	38	1	X.parietina glXanPari20	Simple_repeat
(GGTT)N#Simple_repeat	38	1	X.parietina glXanPari20	Simple_repeat
(GTCCA)N#Simple_repeat	38	1	X.parietina glXanPari20	Simple_repeat
(GTCTATT)N#Simple_repeat	38	1	X.parietina glXanPari20	Simple_repeat
(GTGTTG)N#Simple_repeat	38	1	X.parietina glXanPari20	Simple_repeat
(TAACTTA)N#Simple_repeat	38	1	X.parietina glXanPari20	Simple_repeat
(TACTTAC)N#Simple_repeat	38	1	X.parietina glXanPari20	Simple_repeat
(TCCATC)N#Simple_repeat	38	1	X.parietina glXanPari20	Simple_repeat
(TCGTGG)N#Simple_repeat	38	1	X.parietina glXanPari20	Simple_repeat

(TCTCA)N#Simple_repeat	38	1	X.parietina glXanPari20	Simple_repeat
(TCTGCCA)N#Simple_repeat	38	1	X.parietina glXanPari20	Simple_repeat
(TGGGTGGA)N#Simple_repeat	38	1	X.parietina glXanPari20	Simple_repeat
(TTGGTCA)N#Simple_repeat	38	1	X.parietina glXanPari20	Simple_repeat
(TTTTCTG)N#Simple_repeat	38	1	X.parietina glXanPari20	Simple_repeat
(AAAAGAA)N#Simple_repeat	37	1	X.parietina glXanPari20	Simple_repeat
(AAGCT)N#Simple_repeat	37	1	X.parietina glXanPari20	Simple_repeat
(AATTATC)N#Simple_repeat	37	1	X.parietina glXanPari20	Simple_repeat
(AGGAAGC)N#Simple_repeat	37	1	X.parietina glXanPari20	Simple_repeat
(AGGG)N#Simple_repeat	37	1	X.parietina glXanPari20	Simple_repeat
(AGTCACA)N#Simple_repeat	37	1	X.parietina glXanPari20	Simple_repeat
(ATAACGA)N#Simple_repeat	37	1	X.parietina glXanPari20	Simple_repeat
(CAAGATCC)N#Simple_repeat	37	1	X.parietina glXanPari20	Simple_repeat
(CACCATC)N#Simple_repeat	37	1	X.parietina glXanPari20	Simple_repeat
(CCAACG)N#Simple_repeat	37	1	X.parietina glXanPari20	Simple_repeat
(CCAAT)N#Simple_repeat	37	1	X.parietina glXanPari20	Simple_repeat
(CCATCTC)N#Simple_repeat	37	1	X.parietina glXanPari20	Simple_repeat
(CCCACCC)N#Simple_repeat	37	1	X.parietina glXanPari20	Simple_repeat
(CGTAGAG)N#Simple_repeat	37	1	X.parietina glXanPari20	Simple_repeat
(GCCGGAGCA)N#Simple_repeat	37	1	X.parietina glXanPari20	Simple_repeat
(GCGCCA)N#Simple_repeat	37	1	X.parietina glXanPari20	Simple_repeat
(GCTCAT)N#Simple_repeat	37	1	X.parietina glXanPari20	Simple_repeat
(GGTAAG)N#Simple_repeat	37	1	X.parietina glXanPari20	Simple_repeat
(GTAGAA)N#Simple_repeat	37	1	X.parietina glXanPari20	Simple_repeat
(GTAGTAG)N#Simple_repeat	37	1	X.parietina glXanPari20	Simple_repeat
(GTCACA)N#Simple_repeat	37	1	X.parietina glXanPari20	Simple_repeat

(TATCC)N#Simple_repeat	37	1	X.parietina glXanPari20	Simple_repeat
(TATCGA)N#Simple_repeat	37	1	X.parietina glXanPari20	Simple_repeat
(TCCCTAT)N#Simple_repeat	37	1	X.parietina glXanPari20	Simple_repeat
(TCCTTGCCC)N#Simple_repeat	37	1	X.parietina glXanPari20	Simple_repeat
(TGCCTT)N#Simple_repeat	37	1	X.parietina glXanPari20	Simple_repeat
(TGGAGCG)N#Simple_repeat	37	1	X.parietina glXanPari20	Simple_repeat
(TGGATGA)N#Simple_repeat	37	1	X.parietina glXanPari20	Simple_repeat
(TGGCCG)N#Simple_repeat	37	1	X.parietina glXanPari20	Simple_repeat
(TGTTGCTGC)N#Simple_repeat	37	1	X.parietina glXanPari20	Simple_repeat
(TTTTTCT)N#Simple_repeat	37	1	X.parietina glXanPari20	Simple_repeat
(TTTTTTG)N#Simple_repeat	37	1	X.parietina glXanPari20	Simple_repeat
(AACCCC)N#Simple_repeat	36	1	X.parietina glXanPari20	Simple_repeat
(AATCTA)N#Simple_repeat	36	1	X.parietina glXanPari20	Simple_repeat
(ACCCATCC)N#Simple_repeat	36	1	X.parietina glXanPari20	Simple_repeat
(AGAGT)N#Simple_repeat	36	1	X.parietina glXanPari20	Simple_repeat
(AGTCGGG)N#Simple_repeat	36	1	X.parietina glXanPari20	Simple_repeat
(ATTAAC)N#Simple_repeat	36	1	X.parietina glXanPari20	Simple_repeat
(CAAGGC)N#Simple_repeat	36	1	X.parietina glXanPari20	Simple_repeat
(CACCGC)N#Simple_repeat	36	1	X.parietina glXanPari20	Simple_repeat
(CAGAATC)N#Simple_repeat	36	1	X.parietina glXanPari20	Simple_repeat
(CAGGA)N#Simple_repeat	36	1	X.parietina glXanPari20	Simple_repeat
(CCCTCTT)N#Simple_repeat	36	1	X.parietina glXanPari20	Simple_repeat
(CCTATC)N#Simple_repeat	36	1	X.parietina glXanPari20	Simple_repeat
(CGAGTT)N#Simple_repeat	36	1	X.parietina glXanPari20	Simple_repeat
(CGTTTTT)N#Simple_repeat	36	1	X.parietina glXanPari20	Simple_repeat
(CTACGA)N#Simple_repeat	36	1	X.parietina glXanPari20	Simple_repeat

(CTCACT)N#Simple_repeat	36	1	X.parietina glXanPari20	Simple_repeat
(CTGCCC)N#Simple_repeat	36	1	X.parietina glXanPari20	Simple_repeat
(CTTGACAAT)N#Simple_repeat	36	1	X.parietina glXanPari20	Simple_repeat
(GAACCA)N#Simple_repeat	36	1	X.parietina glXanPari20	Simple_repeat
(GAGAGC)N#Simple_repeat	36	1	X.parietina glXanPari20	Simple_repeat
(GAGGCTG)N#Simple_repeat	36	1	X.parietina glXanPari20	Simple_repeat
(GCTAT)N#Simple_repeat	36	1	X.parietina glXanPari20	Simple_repeat
(GCTTCGA)N#Simple_repeat	36	1	X.parietina glXanPari20	Simple_repeat
(GGAGGGGTG)N#Simple_repeat	36	1	X.parietina glXanPari20	Simple_repeat
(GGAGTG)N#Simple_repeat	36	1	X.parietina glXanPari20	Simple_repeat
(GGTCCA)N#Simple_repeat	36	1	X.parietina glXanPari20	Simple_repeat
(GGTGA)N#Simple_repeat	36	1	X.parietina glXanPari20	Simple_repeat
(GTGATT)N#Simple_repeat	36	1	X.parietina glXanPari20	Simple_repeat
(GTTATT)N#Simple_repeat	36	1	X.parietina glXanPari20	Simple_repeat
(GTTGGT)N#Simple_repeat	36	1	X.parietina glXanPari20	Simple_repeat
(TCCTGG)N#Simple_repeat	36	1	X.parietina glXanPari20	Simple_repeat
(TCTAC)N#Simple_repeat	36	1	X.parietina glXanPari20	Simple_repeat
(TCTTCCT)N#Simple_repeat	36	1	X.parietina glXanPari20	Simple_repeat
(TGCTCA)N#Simple_repeat	36	1	X.parietina glXanPari20	Simple_repeat
(TGTCGCT)N#Simple_repeat	36	1	X.parietina glXanPari20	Simple_repeat
(TGTTGTTGG)N#Simple_repeat	36	1	X.parietina glXanPari20	Simple_repeat
(TTCTGTTA)N#Simple_repeat	36	1	X.parietina glXanPari20	Simple_repeat
(TTGAGG)N#Simple_repeat	36	1	X.parietina glXanPari20	Simple_repeat
(TTTGATT)N#Simple_repeat	36	1	X.parietina glXanPari20	Simple_repeat
(AACAAC)N#Simple_repeat	35	1	X.parietina glXanPari20	Simple_repeat
(AACAT)N#Simple_repeat	35	1	X.parietina glXanPari20	Simple_repeat

(AACGAC)N#Simple_repeat	35	1	X.parietina glXanPari20	Simple_repeat
(AATCGG)N#Simple_repeat	35	1	X.parietina glXanPari20	Simple_repeat
(AATTC)N#Simple_repeat	35	1	X.parietina glXanPari20	Simple_repeat
(ACGATG)N#Simple_repeat	35	1	X.parietina glXanPari20	Simple_repeat
(AGACG)N#Simple_repeat	35	1	X.parietina glXanPari20	Simple_repeat
(AGCT)N#Simple_repeat	35	1	X.parietina glXanPari20	Simple_repeat
(ATATCC)N#Simple_repeat	35	1	X.parietina glXanPari20	Simple_repeat
(ATGCTC)N#Simple_repeat	35	1	X.parietina glXanPari20	Simple_repeat
(CAACCGC)N#Simple_repeat	35	1	X.parietina glXanPari20	Simple_repeat
(CACGAG)N#Simple_repeat	35	1	X.parietina glXanPari20	Simple_repeat
(CCCAATC)N#Simple_repeat	35	1	X.parietina glXanPari20	Simple_repeat
(CCCCATT)N#Simple_repeat	35	1	X.parietina glXanPari20	Simple_repeat
(CCCTTG)N#Simple_repeat	35	1	X.parietina glXanPari20	Simple_repeat
(CCTATT)N#Simple_repeat	35	1	X.parietina glXanPari20	Simple_repeat
(CCTCAG)N#Simple_repeat	35	1	X.parietina glXanPari20	Simple_repeat
(CCTCGTT)N#Simple_repeat	35	1	X.parietina glXanPari20	Simple_repeat
(CGAATA)N#Simple_repeat	35	1	X.parietina glXanPari20	Simple_repeat
(CGCTGT)N#Simple_repeat	35	1	X.parietina glXanPari20	Simple_repeat
(CTTTCG)N#Simple_repeat	35	1	X.parietina glXanPari20	Simple_repeat
(GAATGG)N#Simple_repeat	35	1	X.parietina glXanPari20	Simple_repeat
(GAGGTTG)N#Simple_repeat	35	1	X.parietina glXanPari20	Simple_repeat
(GATTGG)N#Simple_repeat	35	1	X.parietina glXanPari20	Simple_repeat
(GGCGGTT)N#Simple_repeat	35	1	X.parietina glXanPari20	Simple_repeat
(GGTAGA)N#Simple_repeat	35	1	X.parietina glXanPari20	Simple_repeat
(GTACCA)N#Simple_repeat	35	1	X.parietina glXanPari20	Simple_repeat
(GTCCT)N#Simple_repeat	35	1	X.parietina glXanPari20	Simple_repeat

(GTCTTC)N#Simple_repeat	35	1	X.parietina glXanPari20	Simple_repeat
(TATAG)N#Simple_repeat	35	1	X.parietina glXanPari20	Simple_repeat
(TCACACT)N#Simple_repeat	35	1	X.parietina glXanPari20	Simple_repeat
(TCCGTC)N#Simple_repeat	35	1	X.parietina glXanPari20	Simple_repeat
(TCTTT)N#Simple_repeat	35	1	X.parietina glXanPari20	Simple_repeat
(TGGTGGA)N#Simple_repeat	35	1	X.parietina glXanPari20	Simple_repeat
(TGTCCA)N#Simple_repeat	35	1	X.parietina glXanPari20	Simple_repeat
(TTCCCCC)N#Simple_repeat	35	1	X.parietina glXanPari20	Simple_repeat
(TTCCTCC)N#Simple_repeat	35	1	X.parietina glXanPari20	Simple_repeat
(AAAGGGG)N#Simple_repeat	34	1	X.parietina glXanPari20	Simple_repeat
(ACATC)N#Simple_repeat	34	1	X.parietina glXanPari20	Simple_repeat
(ACGGG)N#Simple_repeat	34	1	X.parietina glXanPari20	Simple_repeat
(AGAACAC)N#Simple_repeat	34	1	X.parietina glXanPari20	Simple_repeat
(AGAGAGTG)N#Simple_repeat	34	1	X.parietina glXanPari20	Simple_repeat
(ATAATC)N#Simple_repeat	34	1	X.parietina glXanPari20	Simple_repeat
(ATCCCG)N#Simple_repeat	34	1	X.parietina glXanPari20	Simple_repeat
(ATGTG)N#Simple_repeat	34	1	X.parietina glXanPari20	Simple_repeat
(ATTAGG)N#Simple_repeat	34	1	X.parietina glXanPari20	Simple_repeat
(CACAT)N#Simple_repeat	34	1	X.parietina glXanPari20	Simple_repeat
(CATCAA)N#Simple_repeat	34	1	X.parietina glXanPari20	Simple_repeat
(CATCGG)N#Simple_repeat	34	1	X.parietina glXanPari20	Simple_repeat
(CCGACA)N#Simple_repeat	34	1	X.parietina glXanPari20	Simple_repeat
(CGACGA)N#Simple_repeat	34	1	X.parietina glXanPari20	Simple_repeat
(GAAGGA)N#Simple_repeat	34	1	X.parietina glXanPari20	Simple_repeat
(GAATGA)N#Simple_repeat	34	1	X.parietina glXanPari20	Simple_repeat
(GACGTA)N#Simple_repeat	34	1	X.parietina glXanPari20	Simple_repeat

(GACTTC)N#Simple_repeat	34	1	X.parietina glXanPari20	Simple_repeat
(GAGAAG)N#Simple_repeat	34	1	X.parietina glXanPari20	Simple_repeat
(GATTTG)N#Simple_repeat	34	1	X.parietina glXanPari20	Simple_repeat
(GCAGCG)N#Simple_repeat	34	1	X.parietina glXanPari20	Simple_repeat
(GCCAT)N#Simple_repeat	34	1	X.parietina glXanPari20	Simple_repeat
(GCGGA)N#Simple_repeat	34	1	X.parietina glXanPari20	Simple_repeat
(GCTTCGT)N#Simple_repeat	34	1	X.parietina glXanPari20	Simple_repeat
(GGCGAT)N#Simple_repeat	34	1	X.parietina glXanPari20	Simple_repeat
(GGTTGT)N#Simple_repeat	34	1	X.parietina glXanPari20	Simple_repeat
(GTTTGGA)N#Simple_repeat	34	1	X.parietina glXanPari20	Simple_repeat
(TAAATC)N#Simple_repeat	34	1	X.parietina glXanPari20	Simple_repeat
(TACA)N#Simple_repeat	34	1	X.parietina glXanPari20	Simple_repeat
(TGACGG)N#Simple_repeat	34	1	X.parietina glXanPari20	Simple_repeat
(TGAG)N#Simple_repeat	34	1	X.parietina glXanPari20	Simple_repeat
(TGCTGAT)N#Simple_repeat	34	1	X.parietina glXanPari20	Simple_repeat
(TGTTTT)N#Simple_repeat	34	1	X.parietina glXanPari20	Simple_repeat
(TTAGA)N#Simple_repeat	34	1	X.parietina glXanPari20	Simple_repeat
(TTATAG)N#Simple_repeat	34	1	X.parietina glXanPari20	Simple_repeat
(TTGATC)N#Simple_repeat	34	1	X.parietina glXanPari20	Simple_repeat
(TTTCGG)N#Simple_repeat	34	1	X.parietina glXanPari20	Simple_repeat
(TTTGCT)N#Simple_repeat	34	1	X.parietina glXanPari20	Simple_repeat
(AACAGAT)N#Simple_repeat	33	1	X.parietina glXanPari20	Simple_repeat
(AACTGG)N#Simple_repeat	33	1	X.parietina glXanPari20	Simple_repeat
(AAGGTG)N#Simple_repeat	33	1	X.parietina glXanPari20	Simple_repeat
(AATAGA)N#Simple_repeat	33	1	X.parietina glXanPari20	Simple_repeat
(AATATT)N#Simple_repeat	33	1	X.parietina glXanPari20	Simple_repeat

(AATGCGG)N#Simple_repeat	33	1	X.parietina glXanPari20	Simple_repeat
(AGAGGC)N#Simple_repeat	33	1	X.parietina glXanPari20	Simple_repeat
(AGGGG)N#Simple_repeat	33	1	X.parietina glXanPari20	Simple_repeat
(AGGTGA)N#Simple_repeat	33	1	X.parietina glXanPari20	Simple_repeat
(ATAGCAA)N#Simple_repeat	33	1	X.parietina glXanPari20	Simple_repeat
(ATCAACC)N#Simple_repeat	33	1	X.parietina glXanPari20	Simple_repeat
(CACTCCG)N#Simple_repeat	33	1	X.parietina glXanPari20	Simple_repeat
(CATGCC)N#Simple_repeat	33	1	X.parietina glXanPari20	Simple_repeat
(CCAAA)N#Simple_repeat	33	1	X.parietina glXanPari20	Simple_repeat
(CCGAAG)N#Simple_repeat	33	1	X.parietina glXanPari20	Simple_repeat
(CCGAGT)N#Simple_repeat	33	1	X.parietina glXanPari20	Simple_repeat
(CCTCGACC)N#Simple_repeat	33	1	X.parietina glXanPari20	Simple_repeat
(CTCCAGC)N#Simple_repeat	33	1	X.parietina glXanPari20	Simple_repeat
(CTCGAAG)N#Simple_repeat	33	1	X.parietina glXanPari20	Simple_repeat
(CTTCTG)N#Simple_repeat	33	1	X.parietina glXanPari20	Simple_repeat
(CTTGCT)N#Simple_repeat	33	1	X.parietina glXanPari20	Simple_repeat
(CTTTGC)N#Simple_repeat	33	1	X.parietina glXanPari20	Simple_repeat
(GACACC)N#Simple_repeat	33	1	X.parietina glXanPari20	Simple_repeat
(GACCCTC)N#Simple_repeat	33	1	X.parietina glXanPari20	Simple_repeat
(GAGACT)N#Simple_repeat	33	1	X.parietina glXanPari20	Simple_repeat
(GAGCAC)N#Simple_repeat	33	1	X.parietina glXanPari20	Simple_repeat
(GATCGA)N#Simple_repeat	33	1	X.parietina glXanPari20	Simple_repeat
(GCTGGA)N#Simple_repeat	33	1	X.parietina glXanPari20	Simple_repeat
(GGTTCT)N#Simple_repeat	33	1	X.parietina glXanPari20	Simple_repeat
(GTACT)N#Simple_repeat	33	1	X.parietina glXanPari20	Simple_repeat
(GTGGCA)N#Simple_repeat	33	1	X.parietina glXanPari20	Simple_repeat

(GTTTTG)N#Simple_repeat	33	1	X.parietina glXanPari20	Simple_repeat
(TCCACCT)N#Simple_repeat	33	1	X.parietina glXanPari20	Simple_repeat
(TCTTTCGC)N#Simple_repeat	33	1	X.parietina glXanPari20	Simple_repeat
(TGCTTG)N#Simple_repeat	33	1	X.parietina glXanPari20	Simple_repeat
(TGGG)N#Simple_repeat	33	1	X.parietina glXanPari20	Simple_repeat
(TGTCGT)N#Simple_repeat	33	1	X.parietina glXanPari20	Simple_repeat
(TTCCCT)N#Simple_repeat	33	1	X.parietina glXanPari20	Simple_repeat
(TTCTTT)N#Simple_repeat	33	1	X.parietina glXanPari20	Simple_repeat
(TTGGTG)N#Simple_repeat	33	1	X.parietina glXanPari20	Simple_repeat
(TTGTCA)N#Simple_repeat	33	1	X.parietina glXanPari20	Simple_repeat
(AACATC)N#Simple_repeat	32	1	X.parietina glXanPari20	Simple_repeat
(AAGGAA)N#Simple_repeat	32	1	X.parietina glXanPari20	Simple_repeat
(AAGGT)N#Simple_repeat	32	1	X.parietina glXanPari20	Simple_repeat
(AGAAG)N#Simple_repeat	32	1	X.parietina glXanPari20	Simple_repeat
(AGATT)N#Simple_repeat	32	1	X.parietina glXanPari20	Simple_repeat
(AGTGGC)N#Simple_repeat	32	1	X.parietina glXanPari20	Simple_repeat
(ATAGATC)N#Simple_repeat	32	1	X.parietina glXanPari20	Simple_repeat
(CAAACT)N#Simple_repeat	32	1	X.parietina glXanPari20	Simple_repeat
(CAATGG)N#Simple_repeat	32	1	X.parietina glXanPari20	Simple_repeat
(CAGTGG)N#Simple_repeat	32	1	X.parietina glXanPari20	Simple_repeat
(CCACAG)N#Simple_repeat	32	1	X.parietina glXanPari20	Simple_repeat
(CCCCTGA)N#Simple_repeat	32	1	X.parietina glXanPari20	Simple_repeat
(CCTTCG)N#Simple_repeat	32	1	X.parietina glXanPari20	Simple_repeat
(CGATGGT)N#Simple_repeat	32	1	X.parietina glXanPari20	Simple_repeat
(CGCCTT)N#Simple_repeat	32	1	X.parietina glXanPari20	Simple_repeat
(CGGCAT)N#Simple_repeat	32	1	X.parietina glXanPari20	Simple_repeat

(CTCGCGT)N#Simple_repeat	32	1	X.parietina glXanPari20	Simple_repeat
(GAGGTC)N#Simple_repeat	32	1	X.parietina glXanPari20	Simple_repeat
(GATC)N#Simple_repeat	32	1	X.parietina glXanPari20	Simple_repeat
(GCGGAA)N#Simple_repeat	32	1	X.parietina glXanPari20	Simple_repeat
(GGCGGC)N#Simple_repeat	32	1	X.parietina glXanPari20	Simple_repeat
(GGGTTG)N#Simple_repeat	32	1	X.parietina glXanPari20	Simple_repeat
(GTAT)N#Simple_repeat	32	1	X.parietina glXanPari20	Simple_repeat
(GTGAGA)N#Simple_repeat	32	1	X.parietina glXanPari20	Simple_repeat
(TCCTTG)N#Simple_repeat	32	1	X.parietina glXanPari20	Simple_repeat
(TGGCCAT)N#Simple_repeat	32	1	X.parietina glXanPari20	Simple_repeat
(TGGGT)N#Simple_repeat	32	1	X.parietina glXanPari20	Simple_repeat
(TGTTGTGC)N#Simple_repeat	32	1	X.parietina glXanPari20	Simple_repeat
(TTCAC)N#Simple_repeat	32	1	X.parietina glXanPari20	Simple_repeat
(TTCATC)N#Simple_repeat	32	1	X.parietina glXanPari20	Simple_repeat
(TTTGAT)N#Simple_repeat	32	1	X.parietina glXanPari20	Simple_repeat
(AAGAGA)N#Simple_repeat	31	1	X.parietina glXanPari20	Simple_repeat
(ACGACT)N#Simple_repeat	31	1	X.parietina glXanPari20	Simple_repeat
(AGAAGG)N#Simple_repeat	31	1	X.parietina glXanPari20	Simple_repeat
(ATAG)N#Simple_repeat	31	1	X.parietina glXanPari20	Simple_repeat
(ATCCAA)N#Simple_repeat	31	1	X.parietina glXanPari20	Simple_repeat
(ATGA)N#Simple_repeat	31	1	X.parietina glXanPari20	Simple_repeat
(ATTAAT)N#Simple_repeat	31	1	X.parietina glXanPari20	Simple_repeat
(CACCTT)N#Simple_repeat	31	1	X.parietina glXanPari20	Simple_repeat
(CCCTA)N#Simple_repeat	31	1	X.parietina glXanPari20	Simple_repeat
(CCGAGA)N#Simple_repeat	31	1	X.parietina glXanPari20	Simple_repeat
(CCGCTT)N#Simple_repeat	31	1	X.parietina glXanPari20	Simple_repeat

(CCTGT)N#Simple_repeat	31	1	X.parietina glXanPari20	Simple_repeat
(CGAATC)N#Simple_repeat	31	1	X.parietina glXanPari20	Simple_repeat
(CGATCC)N#Simple_repeat	31	1	X.parietina glXanPari20	Simple_repeat
(CGATTT)N#Simple_repeat	31	1	X.parietina glXanPari20	Simple_repeat
(CTGCAA)N#Simple_repeat	31	1	X.parietina glXanPari20	Simple_repeat
(GAAGTG)N#Simple_repeat	31	1	X.parietina glXanPari20	Simple_repeat
(GAATT)N#Simple_repeat	31	1	X.parietina glXanPari20	Simple_repeat
(GAGGGA)N#Simple_repeat	31	1	X.parietina glXanPari20	Simple_repeat
(GCAAGT)N#Simple_repeat	31	1	X.parietina glXanPari20	Simple_repeat
(GCCATC)N#Simple_repeat	31	1	X.parietina glXanPari20	Simple_repeat
(GCTGTT)N#Simple_repeat	31	1	X.parietina glXanPari20	Simple_repeat
(GGGAAA)N#Simple_repeat	31	1	X.parietina glXanPari20	Simple_repeat
(GGGGAG)N#Simple_repeat	31	1	X.parietina glXanPari20	Simple_repeat
(GGTCAA)N#Simple_repeat	31	1	X.parietina glXanPari20	Simple_repeat
(GGTGAC)N#Simple_repeat	31	1	X.parietina glXanPari20	Simple_repeat
(GGTGTC)N#Simple_repeat	31	1	X.parietina glXanPari20	Simple_repeat
(TCCCG)N#Simple_repeat	31	1	X.parietina glXanPari20	Simple_repeat
(TCCGCT)N#Simple_repeat	31	1	X.parietina glXanPari20	Simple_repeat
(TCGGAA)N#Simple_repeat	31	1	X.parietina glXanPari20	Simple_repeat
(TGCTAT)N#Simple_repeat	31	1	X.parietina glXanPari20	Simple_repeat
(TGCTGG)N#Simple_repeat	31	1	X.parietina glXanPari20	Simple_repeat
(TGGAA)N#Simple_repeat	31	1	X.parietina glXanPari20	Simple_repeat
(TGTTAG)N#Simple_repeat	31	1	X.parietina glXanPari20	Simple_repeat
(TTACA)N#Simple_repeat	31	1	X.parietina glXanPari20	Simple_repeat
(TTGTT)N#Simple_repeat	31	1	X.parietina glXanPari20	Simple_repeat
(AAAGAA)N#Simple_repeat	30	1	X.parietina glXanPari20	Simple_repeat

(AATCCC)N#Simple_repeat	30	1	X.parietina glXanPari20	Simple_repeat
(AGAACG)N#Simple_repeat	30	1	X.parietina glXanPari20	Simple_repeat
(AGGTGC)N#Simple_repeat	30	1	X.parietina glXanPari20	Simple_repeat
(ATCCTT)N#Simple_repeat	30	1	X.parietina glXanPari20	Simple_repeat
(ATCTT)N#Simple_repeat	30	1	X.parietina glXanPari20	Simple_repeat
(CAATT)N#Simple_repeat	30	1	X.parietina glXanPari20	Simple_repeat
(CATCA)N#Simple_repeat	30	1	X.parietina glXanPari20	Simple_repeat
(CATCTA)N#Simple_repeat	30	1	X.parietina glXanPari20	Simple_repeat
(CCATAC)N#Simple_repeat	30	1	X.parietina glXanPari20	Simple_repeat
(CCGCCG)N#Simple_repeat	30	1	X.parietina glXanPari20	Simple_repeat
(CGTAA)N#Simple_repeat	30	1	X.parietina glXanPari20	Simple_repeat
(CTCAG)N#Simple_repeat	30	1	X.parietina glXanPari20	Simple_repeat
(CTCCGG)N#Simple_repeat	30	1	X.parietina glXanPari20	Simple_repeat
(CTTCG)N#Simple_repeat	30	1	X.parietina glXanPari20	Simple_repeat
(GACGTG)N#Simple_repeat	30	1	X.parietina glXanPari20	Simple_repeat
(GGAGAG)N#Simple_repeat	30	1	X.parietina glXanPari20	Simple_repeat
(GGCGA)N#Simple_repeat	30	1	X.parietina glXanPari20	Simple_repeat
(GTTCCA)N#Simple_repeat	30	1	X.parietina glXanPari20	Simple_repeat
(GTTTAC)N#Simple_repeat	30	1	X.parietina glXanPari20	Simple_repeat
(GTTTG)N#Simple_repeat	30	1	X.parietina glXanPari20	Simple_repeat
(TATGTT)N#Simple_repeat	30	1	X.parietina glXanPari20	Simple_repeat
(TCAGCT)N#Simple_repeat	30	1	X.parietina glXanPari20	Simple_repeat
(TCCACCC)N#Simple_repeat	30	1	X.parietina glXanPari20	Simple_repeat
(ACCCTA)N#Simple_repeat	29	1	X.parietina glXanPari20	Simple_repeat
(AGCAA)N#Simple_repeat	29	1	X.parietina glXanPari20	Simple_repeat
(ATGTAC)N#Simple_repeat	29	1	X.parietina glXanPari20	Simple_repeat

(CACAC)N#Simple_repeat	29	1	X.parietina glXanPari20	Simple_repeat
(CACGAC)N#Simple_repeat	29	1	X.parietina glXanPari20	Simple_repeat
(CATTCC)N#Simple_repeat	29	1	X.parietina glXanPari20	Simple_repeat
(CCCACG)N#Simple_repeat	29	1	X.parietina glXanPari20	Simple_repeat
(CTCCG)N#Simple_repeat	29	1	X.parietina glXanPari20	Simple_repeat
(CTCGGA)N#Simple_repeat	29	1	X.parietina glXanPari20	Simple_repeat
(CTGCT)N#Simple_repeat	29	1	X.parietina glXanPari20	Simple_repeat
(GAAGC)N#Simple_repeat	29	1	X.parietina glXanPari20	Simple_repeat
(GAAGGAA)N#Simple_repeat	29	1	X.parietina glXanPari20	Simple_repeat
(GAATA)N#Simple_repeat	29	1	X.parietina glXanPari20	Simple_repeat
(GCGACG)N#Simple_repeat	29	1	X.parietina glXanPari20	Simple_repeat
(GCGTGA)N#Simple_repeat	29	1	X.parietina glXanPari20	Simple_repeat
(GCTGCA)N#Simple_repeat	29	1	X.parietina glXanPari20	Simple_repeat
(GGGGA)N#Simple_repeat	29	1	X.parietina glXanPari20	Simple_repeat
(GGGGT)N#Simple_repeat	29	1	X.parietina glXanPari20	Simple_repeat
(TAAT)N#Simple_repeat	29	1	X.parietina glXanPari20	Simple_repeat
(TGACA)N#Simple_repeat	29	1	X.parietina glXanPari20	Simple_repeat
(TGCTG)N#Simple_repeat	29	1	X.parietina glXanPari20	Simple_repeat
(TGTC)N#Simple_repeat	29	1	X.parietina glXanPari20	Simple_repeat
(TTTACC)N#Simple_repeat	29	1	X.parietina glXanPari20	Simple_repeat
(AAAAGG)N#Simple_repeat	28	1	X.parietina glXanPari20	Simple_repeat
(AAGCTG)N#Simple_repeat	28	1	X.parietina glXanPari20	Simple_repeat
(ACCGT)N#Simple_repeat	28	1	X.parietina glXanPari20	Simple_repeat
(ACCTT)N#Simple_repeat	28	1	X.parietina glXanPari20	Simple_repeat
(AGACA)N#Simple_repeat	28	1	X.parietina glXanPari20	Simple_repeat
(AGGCG)N#Simple_repeat	28	1	X.parietina glXanPari20	Simple_repeat

(AGGTCA)N#Simple_repeat	28	1	X.parietina glXanPari20	Simple_repeat
(CACCA)N#Simple_repeat	28	1	X.parietina glXanPari20	Simple_repeat
(CAGA)N#Simple_repeat	28	1	X.parietina glXanPari20	Simple_repeat
(CCTGTG)N#Simple_repeat	28	1	X.parietina glXanPari20	Simple_repeat
(CTCTC)N#Simple_repeat	28	1	X.parietina glXanPari20	Simple_repeat
(CTGAT)N#Simple_repeat	28	1	X.parietina glXanPari20	Simple_repeat
(CTGCTA)N#Simple_repeat	28	1	X.parietina glXanPari20	Simple_repeat
(CTTGAC)N#Simple_repeat	28	1	X.parietina glXanPari20	Simple_repeat
(GCGG)N#Simple_repeat	28	1	X.parietina glXanPari20	Simple_repeat
(GCTG)N#Simple_repeat	28	1	X.parietina glXanPari20	Simple_repeat
(GGACG)N#Simple_repeat	28	1	X.parietina glXanPari20	Simple_repeat
(GGAGCTT)N#Simple_repeat	28	1	X.parietina glXanPari20	Simple_repeat
(GGGAG)N#Simple_repeat	28	1	X.parietina glXanPari20	Simple_repeat
(GGTA)N#Simple_repeat	28	1	X.parietina glXanPari20	Simple_repeat
(GGTTA)N#Simple_repeat	28	1	X.parietina glXanPari20	Simple_repeat
(GTTGCT)N#Simple_repeat	28	1	X.parietina glXanPari20	Simple_repeat
(TATCA)N#Simple_repeat	28	1	X.parietina glXanPari20	Simple_repeat
(TGTCT)N#Simple_repeat	28	1	X.parietina glXanPari20	Simple_repeat
(TTGT)N#Simple_repeat	28	1	X.parietina glXanPari20	Simple_repeat
(AAACC)N#Simple_repeat	27	1	X.parietina glXanPari20	Simple_repeat
(AACAC)N#Simple_repeat	27	1	X.parietina glXanPari20	Simple_repeat
(AAGGAG)N#Simple_repeat	27	1	X.parietina glXanPari20	Simple_repeat
(AGAC)N#Simple_repeat	27	1	X.parietina glXanPari20	Simple_repeat
(AGCGG)N#Simple_repeat	27	1	X.parietina glXanPari20	Simple_repeat
(ATAGC)N#Simple_repeat	27	1	X.parietina glXanPari20	Simple_repeat
(CAATA)N#Simple_repeat	27	1	X.parietina glXanPari20	Simple_repeat

(CAGCA)N#Simple_repeat	27	1	X.parietina glXanPari20	Simple_repeat
(CCACC)N#Simple_repeat	27	1	X.parietina glXanPari20	Simple_repeat
(CCAGT)N#Simple_repeat	27	1	X.parietina glXanPari20	Simple_repeat
(CGGT)N#Simple_repeat	27	1	X.parietina glXanPari20	Simple_repeat
(CTACT)N#Simple_repeat	27	1	X.parietina glXanPari20	Simple_repeat
(GATCGG)N#Simple_repeat	27	1	X.parietina glXanPari20	Simple_repeat
(GCCA)N#Simple_repeat	27	1	X.parietina glXanPari20	Simple_repeat
(GCGA)N#Simple_repeat	27	1	X.parietina glXanPari20	Simple_repeat
(GCTGGG)N#Simple_repeat	27	1	X.parietina glXanPari20	Simple_repeat
(GGGGAT)N#Simple_repeat	27	1	X.parietina glXanPari20	Simple_repeat
(GTGTCG)N#Simple_repeat	27	1	X.parietina glXanPari20	Simple_repeat
(TCATA)N#Simple_repeat	27	1	X.parietina glXanPari20	Simple_repeat
(TGCCT)N#Simple_repeat	27	1	X.parietina glXanPari20	Simple_repeat
(TGGGA)N#Simple_repeat	27	1	X.parietina glXanPari20	Simple_repeat
(TTGAT)N#Simple_repeat	27	1	X.parietina glXanPari20	Simple_repeat
(TTGC)N#Simple_repeat	27	1	X.parietina glXanPari20	Simple_repeat
(TTTCC)N#Simple_repeat	27	1	X.parietina glXanPari20	Simple_repeat
(AACG)N#Simple_repeat	26	1	X.parietina glXanPari20	Simple_repeat
(AAGAAC)N#Simple_repeat	26	1	X.parietina glXanPari20	Simple_repeat
(ACCAA)N#Simple_repeat	26	1	X.parietina glXanPari20	Simple_repeat
(ACGACC)N#Simple_repeat	26	1	X.parietina glXanPari20	Simple_repeat
(AGGAG)N#Simple_repeat	26	1	X.parietina glXanPari20	Simple_repeat
(AGGCA)N#Simple_repeat	26	1	X.parietina glXanPari20	Simple_repeat
(CAAA)N#Simple_repeat	26	1	X.parietina glXanPari20	Simple_repeat
(CAACCC)N#Simple_repeat	26	1	X.parietina glXanPari20	Simple_repeat
(CAGCAA)N#Simple_repeat	26	1	X.parietina glXanPari20	Simple_repeat

(CCATG)N#Simple_repeat	26	1	X.parietina glXanPari20	Simple_repeat
(CTCGCT)N#Simple_repeat	26	1	X.parietina glXanPari20	Simple_repeat
(CTTG)N#Simple_repeat	26	1	X.parietina glXanPari20	Simple_repeat
(GACTG)N#Simple_repeat	26	1	X.parietina glXanPari20	Simple_repeat
(GAGAA)N#Simple_repeat	26	1	X.parietina glXanPari20	Simple_repeat
(GCGCT)N#Simple_repeat	26	1	X.parietina glXanPari20	Simple_repeat
(GGAAT)N#Simple_repeat	26	1	X.parietina glXanPari20	Simple_repeat
(GGATGTT)N#Simple_repeat	26	1	X.parietina glXanPari20	Simple_repeat
(TCCTT)N#Simple_repeat	26	1	X.parietina glXanPari20	Simple_repeat
(TGAAA)N#Simple_repeat	26	1	X.parietina glXanPari20	Simple_repeat
(TGTAT)N#Simple_repeat	26	1	X.parietina glXanPari20	Simple_repeat
(TGTCGG)N#Simple_repeat	26	1	X.parietina glXanPari20	Simple_repeat
(TGTGA)N#Simple_repeat	26	1	X.parietina glXanPari20	Simple_repeat
(TGTGT)N#Simple_repeat	26	1	X.parietina glXanPari20	Simple_repeat
(TTCGCA)N#Simple_repeat	26	1	X.parietina glXanPari20	Simple_repeat
(AAAGC)N#Simple_repeat	25	1	X.parietina glXanPari20	Simple_repeat
(ACGAT)N#Simple_repeat	25	1	X.parietina glXanPari20	Simple_repeat
(AGCG)N#Simple_repeat	25	1	X.parietina glXanPari20	Simple_repeat
(AGCGA)N#Simple_repeat	25	1	X.parietina glXanPari20	Simple_repeat
(AGTA)N#Simple_repeat	25	1	X.parietina glXanPari20	Simple_repeat
(CACG)N#Simple_repeat	25	1	X.parietina glXanPari20	Simple_repeat
(CCTG)N#Simple_repeat	25	1	X.parietina glXanPari20	Simple_repeat
(CGACTG)N#Simple_repeat	25	1	X.parietina glXanPari20	Simple_repeat
(CGTCT)N#Simple_repeat	25	1	X.parietina glXanPari20	Simple_repeat
(CGTGAG)N#Simple_repeat	25	1	X.parietina glXanPari20	Simple_repeat
(GAAATG)N#Simple_repeat	25	1	X.parietina glXanPari20	Simple_repeat

(GACAGC)N#Simple_repeat	25	1	X.parietina glXanPari20	Simple_repeat
(GAGTT)N#Simple_repeat	25	1	X.parietina glXanPari20	Simple_repeat
(GCCT)N#Simple_repeat	25	1	X.parietina glXanPari20	Simple_repeat
(GGCA)N#Simple_repeat	25	1	X.parietina glXanPari20	Simple_repeat
(GGGGC)N#Simple_repeat	25	1	X.parietina glXanPari20	Simple_repeat
(GTGCTT)N#Simple_repeat	25	1	X.parietina glXanPari20	Simple_repeat
(GTTTTC)N#Simple_repeat	25	1	X.parietina glXanPari20	Simple_repeat
(TCCT)N#Simple_repeat	25	1	X.parietina glXanPari20	Simple_repeat
(TGATCG)N#Simple_repeat	25	1	X.parietina glXanPari20	Simple_repeat
(TGGGGC)N#Simple_repeat	25	1	X.parietina glXanPari20	Simple_repeat
(TTCGT)N#Simple_repeat	25	1	X.parietina glXanPari20	Simple_repeat
(AGCA)N#Simple_repeat	24	1	X.parietina glXanPari20	Simple_repeat
(AGGC)N#Simple_repeat	24	1	X.parietina glXanPari20	Simple_repeat
(AGGGAG)N#Simple_repeat	24	1	X.parietina glXanPari20	Simple_repeat
(ATAACA)N#Simple_repeat	24	1	X.parietina glXanPari20	Simple_repeat
(ATTT)N#Simple_repeat	24	1	X.parietina glXanPari20	Simple_repeat
(CCACA)N#Simple_repeat	24	1	X.parietina glXanPari20	Simple_repeat
(CCCTTCC)N#Simple_repeat	24	1	X.parietina glXanPari20	Simple_repeat
(CGTCCT)N#Simple_repeat	24	1	X.parietina glXanPari20	Simple_repeat
(CTCA)N#Simple_repeat	24	1	X.parietina glXanPari20	Simple_repeat
(CTCG)N#Simple_repeat	24	1	X.parietina glXanPari20	Simple_repeat
(CTTCGT)N#Simple_repeat	24	1	X.parietina glXanPari20	Simple_repeat
(GACT)N#Simple_repeat	24	1	X.parietina glXanPari20	Simple_repeat
(GAGCCT)N#Simple_repeat	24	1	X.parietina glXanPari20	Simple_repeat
(GGCTT)N#Simple_repeat	24	1	X.parietina glXanPari20	Simple_repeat
(GGGA)N#Simple_repeat	24	1	X.parietina glXanPari20	Simple_repeat

(TTCA)N#Simple_repeat	24	1	X.parietina glXanPari20	Simple_repeat
(AAGC)N#Simple_repeat	23	1	X.parietina glXanPari20	Simple_repeat
(AGT)N#Simple_repeat	23	1	X.parietina glXanPari20	Simple_repeat
(ATGCT)N#Simple_repeat	23	1	X.parietina glXanPari20	Simple_repeat
(ATTA)N#Simple_repeat	23	1	X.parietina glXanPari20	Simple_repeat
(CCGC)N#Simple_repeat	23	1	X.parietina glXanPari20	Simple_repeat
(CGAT)N#Simple_repeat	23	1	X.parietina glXanPari20	Simple_repeat
(CTCAA)N#Simple_repeat	23	1	X.parietina glXanPari20	Simple_repeat
(CTCT)N#Simple_repeat	23	1	X.parietina glXanPari20	Simple_repeat
(GAAG)N#Simple_repeat	23	1	X.parietina glXanPari20	Simple_repeat
(GTATA)N#Simple_repeat	23	1	X.parietina glXanPari20	Simple_repeat
(GTGGTGG)N#Simple_repeat	23	1	X.parietina glXanPari20	Simple_repeat
(TACG)N#Simple_repeat	23	1	X.parietina glXanPari20	Simple_repeat
(TAGCA)N#Simple_repeat	23	1	X.parietina glXanPari20	Simple_repeat
(TGAA)N#Simple_repeat	23	1	X.parietina glXanPari20	Simple_repeat
(TGCG)N#Simple_repeat	23	1	X.parietina glXanPari20	Simple_repeat
(AATG)N#Simple_repeat	22	1	X.parietina glXanPari20	Simple_repeat
(ATGC)N#Simple_repeat	22	1	X.parietina glXanPari20	Simple_repeat
(GTCA)N#Simple_repeat	22	1	X.parietina glXanPari20	Simple_repeat
(CATG)N#Simple_repeat	21	1	X.parietina glXanPari20	Simple_repeat
(CCGG)N#Simple_repeat	21	1	X.parietina glXanPari20	Simple_repeat
(TAGC)N#Simple_repeat	21	1	X.parietina glXanPari20	Simple_repeat
(TGAGGTTG)N#Simple_repeat	21	1	X.parietina glXanPari20	Simple_repeat
(CGTG)N#Simple_repeat	20	1	X.parietina glXanPari20	Simple_repeat
(ATATA)N#Simple_repeat	19	1	X.parietina glXanPari20	Simple_repeat
(GCAACA)N#Simple_repeat	18	1	X.parietina glXanPari20	Simple_repeat

(GTA)N#Simple_repeat	16	1	X.parietina glXanPari20	Simple_repeat
(TCCCCCC)N#Simple_repeat	16	1	X.parietina glXanPari20	Simple_repeat
(ATGAAG)N#Simple_repeat	12	1	X.parietina glXanPari20	Simple_repeat
(CCACCATCA)N#Simple_repeat	10	1	X.parietina glXanPari20	Simple_repeat
(TGATGA)N#Simple_repeat	9	1	X.parietina glXanPari20	Simple_repeat
(AACCAC)N#Simple_repeat	7	1	X.parietina glXanPari20	Simple_repeat
(CGCGGC)N#Simple_repeat	7	1	X.parietina glXanPari20	Simple_repeat
(GCCGCC)N#Simple_repeat	7	1	X.parietina glXanPari20	Simple_repeat
(CAGCAC)N#Simple_repeat	6	1	X.parietina glXanPari20	Simple_repeat
(TTCTC)N#Simple_repeat	6	1	X.parietina glXanPari20	Simple_repeat
(TTTA)N#Simple_repeat	6	1	X.parietina glXanPari20	Simple_repeat
(GTGGTC)N#Simple_repeat	5	1	X.parietina glXanPari20	Simple_repeat
RND-1_FAMILY-1#LINE/Tad1	1071525	428	X.parietina glXanPari1	LINE/Tad1
RND-1_FAMILY-48#LINE/Tad1	301490	137	X.parietina glXanPari1	LINE/Tad1
RND-1_FAMILY-57#LINE/Tad1	263165	175	X.parietina glXanPari1	LINE/Tad1
RND-1_FAMILY-29#LTR/Ty3	255614	128	X.parietina glXanPari1	LTR/Ty3
RND-1_FAMILY-26#LTR/Copia	243700	107	X.parietina glXanPari1	LTR/Copia
RND-1_FAMILY-36#LTR/Copia	215045	61	X.parietina glXanPari1	LTR/Copia
RND-2_FAMILY-86#LTR/Ty3	156333	58	X.parietina glXanPari1	LTR/Ty3
RND-1_FAMILY-13#DNA/Kolobok-H	136664	68	X.parietina glXanPari1	DNA/Kolobok-H
RND-1_FAMILY-67#LTR/Ty3	132964	54	X.parietina glXanPari1	LTR/Ty3
RND-1_FAMILY-20#Unknown	114462	82	X.parietina glXanPari1	Unknown
RND-1_FAMILY-22#LTR/Ty3	66082	26	X.parietina glXanPari1	LTR/Ty3
RND-1_FAMILY-66#LTR/Ty3	65044	19	X.parietina glXanPari1	LTR/Ty3
RND-1_FAMILY-15#LTR/Ty3	62925	20	X.parietina glXanPari1	LTR/Ty3
RND-3_FAMILY-139#LTR/Ty3	61092	26	X.parietina glXanPari1	LTR/Ty3

RND-1_FAMILY-28#LTR/Ty3	55945	15	X.parietina glXanPari1	LTR/Ty3
RND-1_FAMILY-31#LTR/Ty3	51048	16	X.parietina glXanPari1	LTR/Ty3
RND-3_FAMILY-136#LTR/Ty3	50502	18	X.parietina glXanPari1	LTR/Ty3
RND-1_FAMILY-27#LTR/Ty3	48731	34	X.parietina glXanPari1	LTR/Ty3
RND-2_FAMILY-9#Unknown	34742	64	X.parietina glXanPari1	Unknown
RND-1_FAMILY-32#LINE/I	34291	21	X.parietina glXanPari1	LINE/I
RND-1_FAMILY-62#Unknown	25223	8	X.parietina glXanPari1	Unknown
RND-1_FAMILY-39#LTR/Ty3	25162	19	X.parietina glXanPari1	LTR/Ty3
RND-1_FAMILY-7#LTR/Ty3	24678	6	X.parietina glXanPari1	LTR/Ty3
RND-1_FAMILY-51#LTR/Copia	23024	10	X.parietina glXanPari1	LTR/Copia
RND-1_FAMILY-19#Unknown	22280	129	X.parietina glXanPari1	Unknown
RND-1_FAMILY-37#LTR/Copia	21511	26	X.parietina glXanPari1	LTR/Copia
RND-3_FAMILY-138#LTR/Ty3	19105	8	X.parietina glXanPari1	LTR/Ty3
RND-1_FAMILY-14#DNA/MULE-MuDR	18862	27	X.parietina glXanPari1	DNA/MULE-MuDR
G-RICH#Low_complexity	18366	294	X.parietina glXanPari1	Low_complexity
GA-RICH#Low_complexity	18348	323	X.parietina glXanPari1	Low_complexity
RND-3_FAMILY-117#Unknown	17616	162	X.parietina glXanPari1	Unknown
RND-1_FAMILY-69#LTR/Ty3	17491	14	X.parietina glXanPari1	LTR/Ty3
RND-2_FAMILY-34#LTR/Ty3	17487	16	X.parietina glXanPari1	LTR/Ty3
RND-2_FAMILY-15#Unknown	15143	97	X.parietina glXanPari1	Unknown
RND-1_FAMILY-38#LTR/Ty3	15021	4	X.parietina glXanPari1	LTR/Ty3
RND-1_FAMILY-68#LTR/Ty3	12808	10	X.parietina glXanPari1	LTR/Ty3
RND-1_FAMILY-25#Unknown	12074	16	X.parietina glXanPari1	Unknown
RND-1_FAMILY-56#DNA/MULE-MuDR	11179	9	X.parietina glXanPari1	DNA/MULE-MuDR
RND-1_FAMILY-12#Unknown	9621	49	X.parietina glXanPari1	Unknown

RND-1_FAMILY-2#Unknown	9418	25	X.parietina glXanPari1	Unknown
RND-1_FAMILY-11#Unknown	9187	15	X.parietina glXanPari1	Unknown
RND-3_FAMILY-145#Unknown	9169	70	X.parietina glXanPari1	Unknown
RND-1_FAMILY-70#Unknown	8064	26	X.parietina glXanPari1	Unknown
RND-1_FAMILY-8#Unknown	8019	46	X.parietina glXanPari1	Unknown
RND-1_FAMILY-80#Unknown	7997	10	X.parietina glXanPari1	Unknown
RND-1_FAMILY-44#LTR/Copia	7842	10	X.parietina glXanPari1	LTR/Copia
RND-3_FAMILY-75#Unknown	7456	19	X.parietina glXanPari1	Unknown
RND-1_FAMILY-64#Unknown	7111	19	X.parietina glXanPari1	Unknown
RND-1_FAMILY-50#Unknown	7053	8	X.parietina glXanPari1	Unknown
RND-1_FAMILY-46#Unknown	6945	17	X.parietina glXanPari1	Unknown
RND-1_FAMILY-21#Unknown	6248	20	X.parietina glXanPari1	Unknown
RND-1_FAMILY-30#Unknown	6144	6	X.parietina glXanPari1	Unknown
RND-3_FAMILY-255#Unknown	5979	93	X.parietina glXanPari1	Unknown
RND-1_FAMILY-23#Unknown	5766	68	X.parietina glXanPari1	Unknown
RND-1_FAMILY-60#Unknown	5753	11	X.parietina glXanPari1	Unknown
RND-1_FAMILY-10#Unknown	5473	11	X.parietina glXanPari1	Unknown
RND-1_FAMILY-41#Unknown	5026	74	X.parietina glXanPari1	Unknown
RND-1_FAMILY-40#Unknown	4890	37	X.parietina glXanPari1	Unknown
RND-1_FAMILY-72#Unknown	4785	22	X.parietina glXanPari1	Unknown
RND-1_FAMILY-47#LINE/Tad1	4569	10	X.parietina glXanPari1	LINE/Tad1
RND-1_FAMILY-81#Unknown	4505	10	X.parietina glXanPari1	Unknown
(CCT)N#Simple_repeat	4482	90	X.parietina glXanPari1	Simple_repeat
RND-1_FAMILY-6#Unknown	4411	18	X.parietina glXanPari1	Unknown
A-RICH#Low_complexity	4357	95	X.parietina glXanPari1	Low_complexity
RND-1_FAMILY-17#Unknown	4227	24	X.parietina glXanPari1	Unknown

RND-1_FAMILY-58#LTR/Ty3	4218	11	X.parietina glXanPari1	LTR/Ty3
RND-1_FAMILY-34#Unknown	4120	25	X.parietina glXanPari1	Unknown
RND-1_FAMILY-3#LTR/Ty3	3626	3	X.parietina glXanPari1	LTR/Ty3
RND-1_FAMILY-79#LTR/Ty3	3473	3	X.parietina glXanPari1	LTR/Ty3
RND-1_FAMILY-43#Unknown	3295	24	X.parietina glXanPari1	Unknown
RND-1_FAMILY-24#Unknown	3225	13	X.parietina glXanPari1	Unknown
RND-1_FAMILY-52#Unknown	3134	23	X.parietina glXanPari1	Unknown
RND-1_FAMILY-9#Unknown	3112	12	X.parietina glXanPari1	Unknown
RND-1_FAMILY-76#Unknown	2878	20	X.parietina glXanPari1	Unknown
RND-1_FAMILY-5#Unknown	2876	36	X.parietina glXanPari1	Unknown
RND-1_FAMILY-16#Unknown	2843	35	X.parietina glXanPari1	Unknown
(C)N#Simple_repeat	2706	105	X.parietina glXanPari1	Simple_repeat
RND-1_FAMILY-54#Unknown	2650	35	X.parietina glXanPari1	Unknown
(CTC)N#Simple_repeat	2449	45	X.parietina glXanPari1	Simple_repeat
(TCC)N#Simple_repeat	2438	55	X.parietina glXanPari1	Simple_repeat
(G)N#Simple_repeat	2378	100	X.parietina glXanPari1	Simple_repeat
RND-1_FAMILY-4#Unknown	2371	9	X.parietina glXanPari1	Unknown
(GGT)N#Simple_repeat	2280	56	X.parietina glXanPari1	Simple_repeat
RND-1_FAMILY-75#Unknown	2271	16	X.parietina glXanPari1	Unknown
RND-1_FAMILY-61#Unknown	2270	10	X.parietina glXanPari1	Unknown
RND-1_FAMILY-77#Unknown	2268	17	X.parietina glXanPari1	Unknown
RND-1_FAMILY-49#Unknown	2175	23	X.parietina glXanPari1	Unknown
RND-1_FAMILY-87#Unknown	2048	28	X.parietina glXanPari1	Unknown
RND-1_FAMILY-18#Unknown	2008	37	X.parietina glXanPari1	Unknown
RND-1_FAMILY-59#Unknown	1949	26	X.parietina glXanPari1	Unknown
(CCA)N#Simple_repeat	1880	45	X.parietina glXanPari1	Simple_repeat

RND-1_FAMILY-65#Unknown	1833	26	X.parietina glXanPari1	Unknown
(CTT)N#Simple_repeat	1830	51	X.parietina glXanPari1	Simple_repeat
RND-1_FAMILY-83#Unknown	1826	10	X.parietina glXanPari1	Unknown
RND-3_FAMILY-840#Unknown	1630	29	X.parietina glXanPari1	Unknown
RND-1_FAMILY-82#Unknown	1600	10	X.parietina glXanPari1	Unknown
RND-1_FAMILY-53#Unknown	1561	20	X.parietina glXanPari1	Unknown
(GAT)N#Simple_repeat	1469	40	X.parietina glXanPari1	Simple_repeat
(CAC)N#Simple_repeat	1395	30	X.parietina glXanPari1	Simple_repeat
(TTC)N#Simple_repeat	1317	35	X.parietina glXanPari1	Simple_repeat
(CAA)N#Simple_repeat	1308	38	X.parietina glXanPari1	Simple_repeat
RND-1_FAMILY-35#Unknown	1301	6	X.parietina glXanPari1	Unknown
(TGG)N#Simple_repeat	1264	33	X.parietina glXanPari1	Simple_repeat
(T)N#Simple_repeat	1257	49	X.parietina glXanPari1	Simple_repeat
(ACC)N#Simple_repeat	1225	22	X.parietina glXanPari1	Simple_repeat
(TGC)N#Simple_repeat	1192	27	X.parietina glXanPari1	Simple_repeat
(GTT)N#Simple_repeat	1183	35	X.parietina glXanPari1	Simple_repeat
RND-1_FAMILY-84#Unknown	1177	6	X.parietina glXanPari1	Unknown
(TCT)N#Simple_repeat	1147	34	X.parietina glXanPari1	Simple_repeat
(CAT)N#Simple_repeat	1012	28	X.parietina glXanPari1	Simple_repeat
(GTG)N#Simple_repeat	991	24	X.parietina glXanPari1	Simple_repeat
(GAA)N#Simple_repeat	971	34	X.parietina glXanPari1	Simple_repeat
(CCG)N#Simple_repeat	959	27	X.parietina glXanPari1	Simple_repeat
(CAG)N#Simple_repeat	942	29	X.parietina glXanPari1	Simple_repeat
(GCA)N#Simple_repeat	917	26	X.parietina glXanPari1	Simple_repeat
(TGA)N#Simple_repeat	889	25	X.parietina glXanPari1	Simple_repeat
(TTTTAT)N#Simple_repeat	846	1	X.parietina glXanPari1	Simple_repeat

RND-1_FAMILY-63#Unknown	844	7	X.parietina glXanPari1	Unknown
(TCG)N#Simple_repeat	819	21	X.parietina glXanPari1	Simple_repeat
RND-1_FAMILY-74#Unknown	806	11	X.parietina glXanPari1	Unknown
(GAC)N#Simple_repeat	790	21	X.parietina glXanPari1	Simple_repeat
(TGT)N#Simple_repeat	774	21	X.parietina glXanPari1	Simple_repeat
(GGA)N#Simple_repeat	773	29	X.parietina glXanPari1	Simple_repeat
RND-1_FAMILY-42#DNA/MULE-MuDR	768	4	X.parietina glXanPari1	DNA/MULE-MuDR
(ACA)N#Simple_repeat	765	20	X.parietina glXanPari1	Simple_repeat
(TC)N#Simple_repeat	753	27	X.parietina glXanPari1	Simple_repeat
(ATC)N#Simple_repeat	749	22	X.parietina glXanPari1	Simple_repeat
(TCA)N#Simple_repeat	722	19	X.parietina glXanPari1	Simple_repeat
(GAG)N#Simple_repeat	719	19	X.parietina glXanPari1	Simple_repeat
(CGA)N#Simple_repeat	708	17	X.parietina glXanPari1	Simple_repeat
(AAG)N#Simple_repeat	691	24	X.parietina glXanPari1	Simple_repeat
(GCC)N#Simple_repeat	671	20	X.parietina glXanPari1	Simple_repeat
(A)N#Simple_repeat	648	28	X.parietina glXanPari1	Simple_repeat
(GCT)N#Simple_repeat	639	21	X.parietina glXanPari1	Simple_repeat
(AGA)N#Simple_repeat	627	21	X.parietina glXanPari1	Simple_repeat
RND-1_FAMILY-85#Unknown	602	5	X.parietina glXanPari1	Unknown
(GA)N#Simple_repeat	589	20	X.parietina glXanPari1	Simple_repeat
(CCTCCC)N#Simple_repeat	583	7	X.parietina glXanPari1	Simple_repeat
(GGC)N#Simple_repeat	576	14	X.parietina glXanPari1	Simple_repeat
(CT)N#Simple_repeat	575	18	X.parietina glXanPari1	Simple_repeat
RND-1_FAMILY-78#Unknown	551	8	X.parietina glXanPari1	Unknown
RND-1_FAMILY-73#Unknown	548	11	X.parietina glXanPari1	Unknown
(CCTC)N#Simple_repeat	547	14	X.parietina glXanPari1	Simple_repeat

(GTC)N#Simple_repeat	544	15	X.parietina glXanPari1	Simple_repeat
(TTG)N#Simple_repeat	535	16	X.parietina glXanPari1	Simple_repeat
(AG)N#Simple_repeat	528	19	X.parietina glXanPari1	Simple_repeat
RND-1_FAMILY-45#DNA/MULE-MuDR	520	3	X.parietina glXanPari1	DNA/MULE-MuDR
(TG)N#Simple_repeat	502	16	X.parietina glXanPari1	Simple_repeat
(ATG)N#Simple_repeat	465	13	X.parietina glXanPari1	Simple_repeat
(CCTCC)N#Simple_repeat	460	10	X.parietina glXanPari1	Simple_repeat
(CTCCTT)N#Simple_repeat	448	6	X.parietina glXanPari1	Simple_repeat
(AGC)N#Simple_repeat	446	12	X.parietina glXanPari1	Simple_repeat
(CTG)N#Simple_repeat	428	13	X.parietina glXanPari1	Simple_repeat
(TCCTTC)N#Simple_repeat	421	6	X.parietina glXanPari1	Simple_repeat
(GCG)N#Simple_repeat	416	12	X.parietina glXanPari1	Simple_repeat
(TCCCCT)N#Simple_repeat	416	6	X.parietina glXanPari1	Simple_repeat
(CGC)N#Simple_repeat	400	13	X.parietina glXanPari1	Simple_repeat
(CA)N#Simple_repeat	399	14	X.parietina glXanPari1	Simple_repeat
(CCCTAA)N#Simple_repeat	396	3	X.parietina glXanPari1	Simple_repeat
(GT)N#Simple_repeat	391	13	X.parietina glXanPari1	Simple_repeat
(CCCCTT)N#Simple_repeat	389	7	X.parietina glXanPari1	Simple_repeat
(CCTCCA)N#Simple_repeat	385	5	X.parietina glXanPari1	Simple_repeat
(ACG)N#Simple_repeat	381	11	X.parietina glXanPari1	Simple_repeat
(CGG)N#Simple_repeat	380	12	X.parietina glXanPari1	Simple_repeat
(TCCC)N#Simple_repeat	380	8	X.parietina glXanPari1	Simple_repeat
(CCCCCT)N#Simple_repeat	371	8	X.parietina glXanPari1	Simple_repeat
(CCACCT)N#Simple_repeat	365	8	X.parietina glXanPari1	Simple_repeat
(CCCCT)N#Simple_repeat	360	4	X.parietina glXanPari1	Simple_repeat
(TTTC)N#Simple_repeat	359	9	X.parietina glXanPari1	Simple_repeat

(GGAT)N#Simple_repeat	352	7	X.parietina glXanPari1	Simple_repeat
RND-1_FAMILY-86#Unknown	352	4	X.parietina glXanPari1	Unknown
(TGGA)N#Simple_repeat	348	9	X.parietina glXanPari1	Simple_repeat
(TGCTGT)N#Simple_repeat	344	3	X.parietina glXanPari1	Simple_repeat
(TA)N#Simple_repeat	328	11	X.parietina glXanPari1	Simple_repeat
(CGT)N#Simple_repeat	317	9	X.parietina glXanPari1	Simple_repeat
(GTTAGG)N#Simple_repeat	317	2	X.parietina glXanPari1	Simple_repeat
(CTCCTC)N#Simple_repeat	314	4	X.parietina glXanPari1	Simple_repeat
(CCAC)N#Simple_repeat	307	8	X.parietina glXanPari1	Simple_repeat
(CCCCCA)N#Simple_repeat	307	5	X.parietina glXanPari1	Simple_repeat
(GGAGGC)N#Simple_repeat	306	4	X.parietina glXanPari1	Simple_repeat
(CACCCT)N#Simple_repeat	305	4	X.parietina glXanPari1	Simple_repeat
(GAGGTG)N#Simple_repeat	303	6	X.parietina glXanPari1	Simple_repeat
(GGAGGT)N#Simple_repeat	298	6	X.parietina glXanPari1	Simple_repeat
(GAGGCG)N#Simple_repeat	297	5	X.parietina glXanPari1	Simple_repeat
(CTCCT)N#Simple_repeat	294	7	X.parietina glXanPari1	Simple_repeat
(AAC)N#Simple_repeat	291	9	X.parietina glXanPari1	Simple_repeat
(TAAGG)N#Simple_repeat	290	7	X.parietina glXanPari1	Simple_repeat
(CCAG)N#Simple_repeat	287	4	X.parietina glXanPari1	Simple_repeat
(TCCA)N#Simple_repeat	285	7	X.parietina glXanPari1	Simple_repeat
(GAAGAC)N#Simple_repeat	283	5	X.parietina glXanPari1	Simple_repeat
(CCCCAT)N#Simple_repeat	281	4	X.parietina glXanPari1	Simple_repeat
(ATTG)N#Simple_repeat	277	7	X.parietina glXanPari1	Simple_repeat
(TCGTCC)N#Simple_repeat	274	4	X.parietina glXanPari1	Simple_repeat
(TGGAGG)N#Simple_repeat	274	6	X.parietina glXanPari1	Simple_repeat
(GACGAT)N#Simple_repeat	271	5	X.parietina glXanPari1	Simple_repeat

(AAT)N#Simple_repeat	270	10	X.parietina glXanPari1	Simple_repeat
(CCC)N#Simple_repeat	269	5	X.parietina glXanPari1	Simple_repeat
(GAGGTCGAG)N#Simple_repeat	268	5	X.parietina glXanPari1	Simple_repeat
(CCCTC)N#Simple_repeat	267	6	X.parietina glXanPari1	Simple_repeat
(CTTCCC)N#Simple_repeat	265	4	X.parietina glXanPari1	Simple_repeat
(GTAAG)N#Simple_repeat	265	8	X.parietina glXanPari1	Simple_repeat
(CCGCCT)N#Simple_repeat	264	4	X.parietina glXanPari1	Simple_repeat
(TCACTC)N#Simple_repeat	264	3	X.parietina glXanPari1	Simple_repeat
(CACCAT)N#Simple_repeat	262	6	X.parietina glXanPari1	Simple_repeat
(CCCA)N#Simple_repeat	258	5	X.parietina glXanPari1	Simple_repeat
(GAGGAGGAA)N#Simple_repeat	257	1	X.parietina glXanPari1	Simple_repeat
(GGTGGA)N#Simple_repeat	256	5	X.parietina glXanPari1	Simple_repeat
(CTCCCC)N#Simple_repeat	251	4	X.parietina glXanPari1	Simple_repeat
(ATGG)N#Simple_repeat	249	6	X.parietina glXanPari1	Simple_repeat
(CCCT)N#Simple_repeat	247	6	X.parietina glXanPari1	Simple_repeat
(TTCC)N#Simple_repeat	245	7	X.parietina glXanPari1	Simple_repeat
(GGTGGC)N#Simple_repeat	241	5	X.parietina glXanPari1	Simple_repeat
(AGAGCC)N#Simple_repeat	240	6	X.parietina glXanPari1	Simple_repeat
(TACCT)N#Simple_repeat	237	7	X.parietina glXanPari1	Simple_repeat
(CTCAGGGG)N#Simple_repeat	231	2	X.parietina glXanPari1	Simple_repeat
(TCCACC)N#Simple_repeat	224	5	X.parietina glXanPari1	Simple_repeat
(TGAGAG)N#Simple_repeat	224	3	X.parietina glXanPari1	Simple_repeat
(TTTTC)N#Simple_repeat	224	5	X.parietina glXanPari1	Simple_repeat
(TCCCCCT)N#Simple_repeat	223	2	X.parietina glXanPari1	Simple_repeat
(CCTCAT)N#Simple_repeat	222	6	X.parietina glXanPari1	Simple_repeat
(CCCTT)N#Simple_repeat	221	5	X.parietina glXanPari1	Simple_repeat

(GGTAA)N#Simple_repeat	221	6	X.parietina glXanPari1	Simple_repeat
(AAAATGTA)N#Simple_repeat	219	1	X.parietina glXanPari1	Simple_repeat
(TCTTCC)N#Simple_repeat	217	6	X.parietina glXanPari1	Simple_repeat
(AGG)N#Simple_repeat	215	7	X.parietina glXanPari1	Simple_repeat
(CCTCCCC)N#Simple_repeat	214	2	X.parietina glXanPari1	Simple_repeat
(CCTCGT)N#Simple_repeat	214	3	X.parietina glXanPari1	Simple_repeat
(CCTTCC)N#Simple_repeat	213	3	X.parietina glXanPari1	Simple_repeat
(GGCGGT)N#Simple_repeat	213	4	X.parietina glXanPari1	Simple_repeat
(AT)N#Simple_repeat	212	10	X.parietina glXanPari1	Simple_repeat
(CTCCC)N#Simple_repeat	212	6	X.parietina glXanPari1	Simple_repeat
(GATG)N#Simple_repeat	210	6	X.parietina glXanPari1	Simple_repeat
(ATCC)N#Simple_repeat	209	6	X.parietina glXanPari1	Simple_repeat
(CCTCCT)N#Simple_repeat	209	3	X.parietina glXanPari1	Simple_repeat
(CTTC)N#Simple_repeat	207	4	X.parietina glXanPari1	Simple_repeat
(TTA)N#Simple_repeat	207	6	X.parietina glXanPari1	Simple_repeat
(TCCCA)N#Simple_repeat	205	5	X.parietina glXanPari1	Simple_repeat
(CATCC)N#Simple_repeat	204	6	X.parietina glXanPari1	Simple_repeat
(TCTTAT)N#Simple_repeat	204	1	X.parietina glXanPari1	Simple_repeat
(TTGAGG)N#Simple_repeat	202	3	X.parietina glXanPari1	Simple_repeat
(TTT)N#Simple_repeat	202	3	X.parietina glXanPari1	Simple_repeat
(CCACCG)N#Simple_repeat	201	4	X.parietina glXanPari1	Simple_repeat
(GGAGCA)N#Simple_repeat	200	2	X.parietina glXanPari1	Simple_repeat
(CCAT)N#Simple_repeat	198	7	X.parietina glXanPari1	Simple_repeat
(TCATCG)N#Simple_repeat	197	4	X.parietina glXanPari1	Simple_repeat
(GATGGT)N#Simple_repeat	195	3	X.parietina glXanPari1	Simple_repeat
(TTTTCCCC)N#Simple_repeat	194	1	X.parietina glXanPari1	Simple_repeat

(CCAGCA)N#Simple_repeat	193	4	X.parietina glXanPari1	Simple_repeat
(AC)N#Simple_repeat	191	9	X.parietina glXanPari1	Simple_repeat
(CCCCTC)N#Simple_repeat	191	4	X.parietina glXanPari1	Simple_repeat
(CCTTC)N#Simple_repeat	191	3	X.parietina glXanPari1	Simple_repeat
(CTA)N#Simple_repeat	191	5	X.parietina glXanPari1	Simple_repeat
(TAA)N#Simple_repeat	191	5	X.parietina glXanPari1	Simple_repeat
(CATCAT)N#Simple_repeat	188	3	X.parietina glXanPari1	Simple_repeat
(ACCCTA)N#Simple_repeat	187	1	X.parietina glXanPari1	Simple_repeat
(CCTCTC)N#Simple_repeat	187	3	X.parietina glXanPari1	Simple_repeat
(GGGAA)N#Simple_repeat	187	2	X.parietina glXanPari1	Simple_repeat
(TAC)N#Simple_repeat	187	6	X.parietina glXanPari1	Simple_repeat
(CCCATC)N#Simple_repeat	186	1	X.parietina glXanPari1	Simple_repeat
(CGAGGAAA)N#Simple_repeat	186	1	X.parietina glXanPari1	Simple_repeat
(TTAGGG)N#Simple_repeat	185	1	X.parietina glXanPari1	Simple_repeat
(CTCCCAC)N#Simple_repeat	184	2	X.parietina glXanPari1	Simple_repeat
(CAACCG)N#Simple_repeat	182	2	X.parietina glXanPari1	Simple_repeat
(GAGGAC)N#Simple_repeat	182	4	X.parietina glXanPari1	Simple_repeat
(GGAGTG)N#Simple_repeat	182	4	X.parietina glXanPari1	Simple_repeat
(CCTAAC)N#Simple_repeat	181	1	X.parietina glXanPari1	Simple_repeat
(CTCC)N#Simple_repeat	181	3	X.parietina glXanPari1	Simple_repeat
(TTGA)N#Simple_repeat	181	4	X.parietina glXanPari1	Simple_repeat
(CCCAT)N#Simple_repeat	180	5	X.parietina glXanPari1	Simple_repeat
(CATC)N#Simple_repeat	179	5	X.parietina glXanPari1	Simple_repeat
(CCCTCG)N#Simple_repeat	179	3	X.parietina glXanPari1	Simple_repeat
(CCTCAC)N#Simple_repeat	178	2	X.parietina glXanPari1	Simple_repeat
(GAGGAT)N#Simple_repeat	178	2	X.parietina glXanPari1	Simple_repeat

(AATC)N#Simple_repeat	177	6	X.parietina glXanPari1	Simple_repeat
(GAGAT)N#Simple_repeat	177	4	X.parietina glXanPari1	Simple_repeat
(TCCCT)N#Simple_repeat	176	3	X.parietina glXanPari1	Simple_repeat
(GATGAA)N#Simple_repeat	175	3	X.parietina glXanPari1	Simple_repeat
(GATGCTGT)N#Simple_repeat	174	1	X.parietina glXanPari1	Simple_repeat
(GGATGA)N#Simple_repeat	171	5	X.parietina glXanPari1	Simple_repeat
(CCCAC)N#Simple_repeat	170	3	X.parietina glXanPari1	Simple_repeat
(TCTTCG)N#Simple_repeat	170	4	X.parietina glXanPari1	Simple_repeat
(TAT)N#Simple_repeat	169	5	X.parietina glXanPari1	Simple_repeat
(CTTT)N#Simple_repeat	166	6	X.parietina glXanPari1	Simple_repeat
(ACCA)N#Simple_repeat	165	4	X.parietina glXanPari1	Simple_repeat
(TACC)N#Simple_repeat	165	5	X.parietina glXanPari1	Simple_repeat
(GTGG)N#Simple_repeat	163	2	X.parietina glXanPari1	Simple_repeat
(GGATGT)N#Simple_repeat	162	4	X.parietina glXanPari1	Simple_repeat
(CAACC)N#Simple_repeat	161	4	X.parietina glXanPari1	Simple_repeat
(CCATCCC)N#Simple_repeat	159	3	X.parietina glXanPari1	Simple_repeat
(TGAT)N#Simple_repeat	159	5	X.parietina glXanPari1	Simple_repeat
(CAGT)N#Simple_repeat	158	4	X.parietina glXanPari1	Simple_repeat
(CATCCT)N#Simple_repeat	158	3	X.parietina glXanPari1	Simple_repeat
(CTTTAT)N#Simple_repeat	158	2	X.parietina glXanPari1	Simple_repeat
(GGAAGG)N#Simple_repeat	158	2	X.parietina glXanPari1	Simple_repeat
(CCCTCCC)N#Simple_repeat	154	3	X.parietina glXanPari1	Simple_repeat
(CTTCCT)N#Simple_repeat	153	2	X.parietina glXanPari1	Simple_repeat
(CTCCCAT)N#Simple_repeat	150	2	X.parietina glXanPari1	Simple_repeat
(GCTTT)N#Simple_repeat	150	1	X.parietina glXanPari1	Simple_repeat
(GATGAG)N#Simple_repeat	148	3	X.parietina glXanPari1	Simple_repeat

(GGACGA)N#Simple_repeat	148	3	X.parietina glXanPari1	Simple_repeat
(CCCTCT)N#Simple_repeat	147	4	X.parietina glXanPari1	Simple_repeat
(CCCTCTCT)N#Simple_repeat	147	1	X.parietina glXanPari1	Simple_repeat
(CGAAGA)N#Simple_repeat	147	2	X.parietina glXanPari1	Simple_repeat
(GGATG)N#Simple_repeat	147	4	X.parietina glXanPari1	Simple_repeat
(ACCC)N#Simple_repeat	145	3	X.parietina glXanPari1	Simple_repeat
(CCTTTC)N#Simple_repeat	145	3	X.parietina glXanPari1	Simple_repeat
(AGGGTT)N#Simple_repeat	144	1	X.parietina glXanPari1	Simple_repeat
(GGGAT)N#Simple_repeat	144	4	X.parietina glXanPari1	Simple_repeat
(TGGAT)N#Simple_repeat	144	3	X.parietina glXanPari1	Simple_repeat
(CCCTCC)N#Simple_repeat	143	2	X.parietina glXanPari1	Simple_repeat
(TCATCC)N#Simple_repeat	143	3	X.parietina glXanPari1	Simple_repeat
(AATA)N#Simple_repeat	142	3	X.parietina glXanPari1	Simple_repeat
(GTAT)N#Simple_repeat	142	4	X.parietina glXanPari1	Simple_repeat
(TCAACC)N#Simple_repeat	142	3	X.parietina glXanPari1	Simple_repeat
(AAAGA)N#Simple_repeat	141	2	X.parietina glXanPari1	Simple_repeat
(TCGTCA)N#Simple_repeat	141	3	X.parietina glXanPari1	Simple_repeat
(ACCCTC)N#Simple_repeat	140	3	X.parietina glXanPari1	Simple_repeat
(ATGAA)N#Simple_repeat	140	2	X.parietina glXanPari1	Simple_repeat
(GATGG)N#Simple_repeat	139	3	X.parietina glXanPari1	Simple_repeat
(CATA)N#Simple_repeat	138	3	X.parietina glXanPari1	Simple_repeat
(GTGGTG)N#Simple_repeat	138	2	X.parietina glXanPari1	Simple_repeat
(CTTTCTCCCC)N#Simple_repeat	137	1	X.parietina glXanPari1	Simple_repeat
(GGTGAG)N#Simple_repeat	137	2	X.parietina glXanPari1	Simple_repeat
(CTGCTC)N#Simple_repeat	136	3	X.parietina glXanPari1	Simple_repeat
(AGGAAGG)N#Simple_repeat	135	1	X.parietina glXanPari1	Simple_repeat

(CCTCGACCT)N#Simple_repeat	135	3	X.parietina glXanPari1	Simple_repeat
(CTTCGC)N#Simple_repeat	135	2	X.parietina glXanPari1	Simple_repeat
(TCCTCT)N#Simple_repeat	135	3	X.parietina glXanPari1	Simple_repeat
(TCTCCC)N#Simple_repeat	135	4	X.parietina glXanPari1	Simple_repeat
(CCAACA)N#Simple_repeat	132	3	X.parietina glXanPari1	Simple_repeat
(TGGAGA)N#Simple_repeat	132	3	X.parietina glXanPari1	Simple_repeat
(GCAGGA)N#Simple_repeat	131	2	X.parietina glXanPari1	Simple_repeat
(TATAT)N#Simple_repeat	131	2	X.parietina glXanPari1	Simple_repeat
(TCTTCA)N#Simple_repeat	131	2	X.parietina glXanPari1	Simple_repeat
(ACACC)N#Simple_repeat	130	3	X.parietina glXanPari1	Simple_repeat
(AGATGA)N#Simple_repeat	130	3	X.parietina glXanPari1	Simple_repeat
(CCACAA)N#Simple_repeat	130	3	X.parietina glXanPari1	Simple_repeat
(CTAC)N#Simple_repeat	130	4	X.parietina glXanPari1	Simple_repeat
(TCTCCA)N#Simple_repeat	130	2	X.parietina glXanPari1	Simple_repeat
(ACCAAC)N#Simple_repeat	129	2	X.parietina glXanPari1	Simple_repeat
(CCAACC)N#Simple_repeat	129	2	X.parietina glXanPari1	Simple_repeat
(ACGATG)N#Simple_repeat	128	2	X.parietina glXanPari1	Simple_repeat
(CACCT)N#Simple_repeat	128	3	X.parietina glXanPari1	Simple_repeat
(TCCCC)N#Simple_repeat	127	2	X.parietina glXanPari1	Simple_repeat
(TCGTCT)N#Simple_repeat	127	2	X.parietina glXanPari1	Simple_repeat
(TGGCGG)N#Simple_repeat	127	3	X.parietina glXanPari1	Simple_repeat
(CACCAC)N#Simple_repeat	126	3	X.parietina glXanPari1	Simple_repeat
(CTCCAC)N#Simple_repeat	126	5	X.parietina glXanPari1	Simple_repeat
(GTTG)N#Simple_repeat	126	3	X.parietina glXanPari1	Simple_repeat
(AAGG)N#Simple_repeat	125	5	X.parietina glXanPari1	Simple_repeat
(CCATCC)N#Simple_repeat	125	3	X.parietina glXanPari1	Simple_repeat

(GGTGA)N#Simple_repeat	125	3	X.parietina glXanPari1	Simple_repeat
(TTGG)N#Simple_repeat	125	5	X.parietina glXanPari1	Simple_repeat
(CAGCAAAC)N#Simple_repeat	124	1	X.parietina glXanPari1	Simple_repeat
(CTAACC)N#Simple_repeat	124	1	X.parietina glXanPari1	Simple_repeat
(GACGAA)N#Simple_repeat	124	2	X.parietina glXanPari1	Simple_repeat
(TGGC)N#Simple_repeat	123	5	X.parietina glXanPari1	Simple_repeat
(ACCCCC)N#Simple_repeat	122	2	X.parietina glXanPari1	Simple_repeat
(GGAGCT)N#Simple_repeat	122	3	X.parietina glXanPari1	Simple_repeat
(TGATG)N#Simple_repeat	122	3	X.parietina glXanPari1	Simple_repeat
(TTCTTG)N#Simple_repeat	122	1	X.parietina glXanPari1	Simple_repeat
(CCATCTC)N#Simple_repeat	121	2	X.parietina glXanPari1	Simple_repeat
(TTCCCC)N#Simple_repeat	120	2	X.parietina glXanPari1	Simple_repeat
(TTTATC)N#Simple_repeat	120	3	X.parietina glXanPari1	Simple_repeat
(CCTT)N#Simple_repeat	119	4	X.parietina glXanPari1	Simple_repeat
(TGTTGC)N#Simple_repeat	119	3	X.parietina glXanPari1	Simple_repeat
(TGTTGG)N#Simple_repeat	119	3	X.parietina glXanPari1	Simple_repeat
(ACACCC)N#Simple_repeat	118	2	X.parietina glXanPari1	Simple_repeat
(ATGCTGGGG)N#Simple_repeat	118	1	X.parietina glXanPari1	Simple_repeat
(CCCCA)N#Simple_repeat	118	3	X.parietina glXanPari1	Simple_repeat
(TCCTCG)N#Simple_repeat	118	3	X.parietina glXanPari1	Simple_repeat
(TCTCC)N#Simple_repeat	118	3	X.parietina glXanPari1	Simple_repeat
(TGGTTGA)N#Simple_repeat	118	2	X.parietina glXanPari1	Simple_repeat
(ACAACA)N#Simple_repeat	117	3	X.parietina glXanPari1	Simple_repeat
(ATAA)N#Simple_repeat	117	4	X.parietina glXanPari1	Simple_repeat
(GTCATC)N#Simple_repeat	117	2	X.parietina glXanPari1	Simple_repeat
(GAGGATT)N#Simple_repeat	116	1	X.parietina glXanPari1	Simple_repeat

(TGGAGGA)N#Simple_repeat	116	2	X.parietina glXanPari1	Simple_repeat
(TTCCCA)N#Simple_repeat	116	2	X.parietina glXanPari1	Simple_repeat
(CAGAAC)N#Simple_repeat	115	1	X.parietina glXanPari1	Simple_repeat
(GAAGAAGGG)N#Simple_repeat	115	1	X.parietina glXanPari1	Simple_repeat
(TTCGTC)N#Simple_repeat	115	2	X.parietina glXanPari1	Simple_repeat
(CCCGCC)N#Simple_repeat	114	2	X.parietina glXanPari1	Simple_repeat
(CTCACT)N#Simple_repeat	114	2	X.parietina glXanPari1	Simple_repeat
(GACTGG)N#Simple_repeat	114	2	X.parietina glXanPari1	Simple_repeat
(GATGAC)N#Simple_repeat	114	3	X.parietina glXanPari1	Simple_repeat
(GGTA)N#Simple_repeat	114	3	X.parietina glXanPari1	Simple_repeat
(TGTGCT)N#Simple_repeat	114	1	X.parietina glXanPari1	Simple_repeat
(CTTCCTT)N#Simple_repeat	113	2	X.parietina glXanPari1	Simple_repeat
(CACCAAC)N#Simple_repeat	112	2	X.parietina glXanPari1	Simple_repeat
(CTCACAT)N#Simple_repeat	112	1	X.parietina glXanPari1	Simple_repeat
(GATT)N#Simple_repeat	111	2	X.parietina glXanPari1	Simple_repeat
(TCCCTCC)N#Simple_repeat	111	2	X.parietina glXanPari1	Simple_repeat
(TGATGG)N#Simple_repeat	111	2	X.parietina glXanPari1	Simple_repeat
(TGTCAT)N#Simple_repeat	111	2	X.parietina glXanPari1	Simple_repeat
(AACACC)N#Simple_repeat	110	1	X.parietina glXanPari1	Simple_repeat
(ACATAT)N#Simple_repeat	110	1	X.parietina glXanPari1	Simple_repeat
(AGGAT)N#Simple_repeat	110	2	X.parietina glXanPari1	Simple_repeat
(ATGGGTCA)N#Simple_repeat	110	1	X.parietina glXanPari1	Simple_repeat
(CCCCCCA)N#Simple_repeat	110	2	X.parietina glXanPari1	Simple_repeat
(TAG)N#Simple_repeat	110	4	X.parietina glXanPari1	Simple_repeat
(TTTCCC)N#Simple_repeat	110	3	X.parietina glXanPari1	Simple_repeat
(CAACAG)N#Simple_repeat	109	3	X.parietina glXanPari1	Simple_repeat

(GAGGATG)N#Simple_repeat	109	2	X.parietina glXanPari1	Simple_repeat
(GATGTG)N#Simple_repeat	109	3	X.parietina glXanPari1	Simple_repeat
(TCACGC)N#Simple_repeat	109	2	X.parietina glXanPari1	Simple_repeat
(AAGGT)N#Simple_repeat	108	2	X.parietina glXanPari1	Simple_repeat
(CCACC)N#Simple_repeat	108	3	X.parietina glXanPari1	Simple_repeat
(CTCCCTC)N#Simple_repeat	108	2	X.parietina glXanPari1	Simple_repeat
(GCAACC)N#Simple_repeat	108	1	X.parietina glXanPari1	Simple_repeat
(TGAA)N#Simple_repeat	108	3	X.parietina glXanPari1	Simple_repeat
(AACCTCC)N#Simple_repeat	107	1	X.parietina glXanPari1	Simple_repeat
(CACCTTC)N#Simple_repeat	107	2	X.parietina glXanPari1	Simple_repeat
(TATCC)N#Simple_repeat	107	2	X.parietina glXanPari1	Simple_repeat
(GCAGCA)N#Simple_repeat	106	1	X.parietina glXanPari1	Simple_repeat
(GGAG)N#Simple_repeat	106	4	X.parietina glXanPari1	Simple_repeat
(GAGTGG)N#Simple_repeat	105	1	X.parietina glXanPari1	Simple_repeat
(GATAT)N#Simple_repeat	105	2	X.parietina glXanPari1	Simple_repeat
(GGATGG)N#Simple_repeat	105	3	X.parietina glXanPari1	Simple_repeat
(TTCCTC)N#Simple_repeat	105	2	X.parietina glXanPari1	Simple_repeat
(CTGAGG)N#Simple_repeat	104	1	X.parietina glXanPari1	Simple_repeat
(CTTTCC)N#Simple_repeat	104	2	X.parietina glXanPari1	Simple_repeat
(AGAAA)N#Simple_repeat	103	2	X.parietina glXanPari1	Simple_repeat
(AGGA)N#Simple_repeat	103	3	X.parietina glXanPari1	Simple_repeat
(GACGAG)N#Simple_repeat	103	2	X.parietina glXanPari1	Simple_repeat
(TCCCCC)N#Simple_repeat	103	2	X.parietina glXanPari1	Simple_repeat
(ACGAAG)N#Simple_repeat	102	1	X.parietina glXanPari1	Simple_repeat
(CATCAC)N#Simple_repeat	102	2	X.parietina glXanPari1	Simple_repeat
(CC)N#Simple_repeat	101	1	X.parietina glXanPari1	Simple_repeat

(CCCAACC)N#Simple_repeat	101	1	X.parietina glXanPari1	Simple_repeat
(CGAAGG)N#Simple_repeat	101	1	X.parietina glXanPari1	Simple_repeat
(GACGAC)N#Simple_repeat	101	2	X.parietina glXanPari1	Simple_repeat
(GACGGC)N#Simple_repeat	101	2	X.parietina glXanPari1	Simple_repeat
(GCAACA)N#Simple_repeat	101	3	X.parietina glXanPari1	Simple_repeat
(TCATCA)N#Simple_repeat	101	2	X.parietina glXanPari1	Simple_repeat
(TTCATC)N#Simple_repeat	101	3	X.parietina glXanPari1	Simple_repeat
(CTCCTCG)N#Simple_repeat	100	2	X.parietina glXanPari1	Simple_repeat
(GCCTCC)N#Simple_repeat	100	1	X.parietina glXanPari1	Simple_repeat
(TCTT)N#Simple_repeat	100	4	X.parietina glXanPari1	Simple_repeat
(CTGG)N#Simple_repeat	99	4	X.parietina glXanPari1	Simple_repeat
(TGGTCG)N#Simple_repeat	99	3	X.parietina glXanPari1	Simple_repeat
(GGAGT)N#Simple_repeat	98	2	X.parietina glXanPari1	Simple_repeat
(TCCGAC)N#Simple_repeat	98	1	X.parietina glXanPari1	Simple_repeat
(GAGT)N#Simple_repeat	97	4	X.parietina glXanPari1	Simple_repeat
(ACCCC)N#Simple_repeat	96	3	X.parietina glXanPari1	Simple_repeat
(CAGC)N#Simple_repeat	96	3	X.parietina glXanPari1	Simple_repeat
(CTTCCTC)N#Simple_repeat	96	2	X.parietina glXanPari1	Simple_repeat
(CTTTT)N#Simple_repeat	96	2	X.parietina glXanPari1	Simple_repeat
(GGAGAT)N#Simple_repeat	96	2	X.parietina glXanPari1	Simple_repeat
(GATTGG)N#Simple_repeat	95	1	X.parietina glXanPari1	Simple_repeat
(TAGAGT)N#Simple_repeat	95	1	X.parietina glXanPari1	Simple_repeat
(AACCTC)N#Simple_repeat	94	2	X.parietina glXanPari1	Simple_repeat
(ACCACAC)N#Simple_repeat	94	1	X.parietina glXanPari1	Simple_repeat
(ACCT)N#Simple_repeat	94	3	X.parietina glXanPari1	Simple_repeat
(AGGT)N#Simple_repeat	94	2	X.parietina glXanPari1	Simple_repeat

(AGGTA)N#Simple_repeat	94	3	X.parietina glXanPari1	Simple_repeat
(GCCAAGAT)N#Simple_repeat	94	1	X.parietina glXanPari1	Simple_repeat
(GCGACA)N#Simple_repeat	94	1	X.parietina glXanPari1	Simple_repeat
(ACCAT)N#Simple_repeat	93	2	X.parietina glXanPari1	Simple_repeat
(ACT)N#Simple_repeat	93	3	X.parietina glXanPari1	Simple_repeat
(AGT)N#Simple_repeat	93	3	X.parietina glXanPari1	Simple_repeat
(CGCCCC)N#Simple_repeat	93	2	X.parietina glXanPari1	Simple_repeat
(AAGA)N#Simple_repeat	92	3	X.parietina glXanPari1	Simple_repeat
(ACCTCC)N#Simple_repeat	92	2	X.parietina glXanPari1	Simple_repeat
(AGATATTC)N#Simple_repeat	92	1	X.parietina glXanPari1	Simple_repeat
(CTGA)N#Simple_repeat	92	2	X.parietina glXanPari1	Simple_repeat
(GACA)N#Simple_repeat	92	2	X.parietina glXanPari1	Simple_repeat
(GTGGCG)N#Simple_repeat	92	2	X.parietina glXanPari1	Simple_repeat
(ATT)N#Simple_repeat	91	4	X.parietina glXanPari1	Simple_repeat
(GCTGAT)N#Simple_repeat	91	2	X.parietina glXanPari1	Simple_repeat
(TGTTGA)N#Simple_repeat	91	1	X.parietina glXanPari1	Simple_repeat
(CCATCA)N#Simple_repeat	90	2	X.parietina glXanPari1	Simple_repeat
(CTTGCA)N#Simple_repeat	90	1	X.parietina glXanPari1	Simple_repeat
(GAAGGAGAA)N#Simple_repeat	90	1	X.parietina glXanPari1	Simple_repeat
(GATCTT)N#Simple_repeat	90	1	X.parietina glXanPari1	Simple_repeat
(GGTGCG)N#Simple_repeat	90	1	X.parietina glXanPari1	Simple_repeat
(GGTGGT)N#Simple_repeat	90	2	X.parietina glXanPari1	Simple_repeat
(TCCATC)N#Simple_repeat	90	2	X.parietina glXanPari1	Simple_repeat
(TCCT)N#Simple_repeat	90	4	X.parietina glXanPari1	Simple_repeat
(TCGATC)N#Simple_repeat	90	2	X.parietina glXanPari1	Simple_repeat
(TGGCGT)N#Simple_repeat	90	2	X.parietina glXanPari1	Simple_repeat

(TGGTG)N#Simple_repeat	90	2	X.parietina glXanPari1	Simple_repeat
(CAAGCAGGG)N#Simple_repeat	89	1	X.parietina glXanPari1	Simple_repeat
(GATGTTGC)N#Simple_repeat	89	1	X.parietina glXanPari1	Simple_repeat
(GTGGAG)N#Simple_repeat	89	2	X.parietina glXanPari1	Simple_repeat
(GTGGAT)N#Simple_repeat	89	2	X.parietina glXanPari1	Simple_repeat
(TCCCACC)N#Simple_repeat	89	2	X.parietina glXanPari1	Simple_repeat
(TCCCTCA)N#Simple_repeat	89	2	X.parietina glXanPari1	Simple_repeat
(TGAGGA)N#Simple_repeat	89	1	X.parietina glXanPari1	Simple_repeat
(AGGACG)N#Simple_repeat	88	2	X.parietina glXanPari1	Simple_repeat
(CAACCA)N#Simple_repeat	88	3	X.parietina glXanPari1	Simple_repeat
(CCCATCT)N#Simple_repeat	88	2	X.parietina glXanPari1	Simple_repeat
(CCCCC)N#Simple_repeat	88	2	X.parietina glXanPari1	Simple_repeat
(CCGACT)N#Simple_repeat	88	2	X.parietina glXanPari1	Simple_repeat
(CTCCAT)N#Simple_repeat	88	1	X.parietina glXanPari1	Simple_repeat
(GAGATG)N#Simple_repeat	88	2	X.parietina glXanPari1	Simple_repeat
(AAGCCG)N#Simple_repeat	87	1	X.parietina glXanPari1	Simple_repeat
(CAAC)N#Simple_repeat	87	2	X.parietina glXanPari1	Simple_repeat
(CTCTCCA)N#Simple_repeat	87	2	X.parietina glXanPari1	Simple_repeat
(TCATAGCTGC)N#Simple_repeat	87	1	X.parietina glXanPari1	Simple_repeat
(TGGGA)N#Simple_repeat	87	3	X.parietina glXanPari1	Simple_repeat
(TTTCCT)N#Simple_repeat	87	2	X.parietina glXanPari1	Simple_repeat
(CACAAC)N#Simple_repeat	86	2	X.parietina glXanPari1	Simple_repeat
(CTCTCC)N#Simple_repeat	86	1	X.parietina glXanPari1	Simple_repeat
(GACC)N#Simple_repeat	86	3	X.parietina glXanPari1	Simple_repeat
(TCCCCA)N#Simple_repeat	86	2	X.parietina glXanPari1	Simple_repeat
(AAGACG)N#Simple_repeat	85	2	X.parietina glXanPari1	Simple_repeat

(ATCTGCA)N#Simple_repeat	85	2	X.parietina glXanPari1	Simple_repeat
(CTTAC)N#Simple_repeat	85	3	X.parietina glXanPari1	Simple_repeat
(GGTGG)N#Simple_repeat	85	1	X.parietina glXanPari1	Simple_repeat
(GTCGGTGAGG)N#Simple_repeat	85	1	X.parietina glXanPari1	Simple_repeat
(TCTCACA)N#Simple_repeat	85	1	X.parietina glXanPari1	Simple_repeat
(ATATTA)N#Simple_repeat	84	1	X.parietina glXanPari1	Simple_repeat
(CTTCTC)N#Simple_repeat	84	2	X.parietina glXanPari1	Simple_repeat
(TCCCTC)N#Simple_repeat	84	2	X.parietina glXanPari1	Simple_repeat
(TGAG)N#Simple_repeat	84	3	X.parietina glXanPari1	Simple_repeat
(TGTA)N#Simple_repeat	84	3	X.parietina glXanPari1	Simple_repeat
(TGTGTA)N#Simple_repeat	84	2	X.parietina glXanPari1	Simple_repeat
(CCTCAG)N#Simple_repeat	83	2	X.parietina glXanPari1	Simple_repeat
(CTGAGCCC)N#Simple_repeat	83	1	X.parietina glXanPari1	Simple_repeat
(GAGCGA)N#Simple_repeat	83	1	X.parietina glXanPari1	Simple_repeat
(TAATATTA)N#Simple_repeat	83	1	X.parietina glXanPari1	Simple_repeat
(TCATCATCG)N#Simple_repeat	83	2	X.parietina glXanPari1	Simple_repeat
(ATCCC)N#Simple_repeat	82	2	X.parietina glXanPari1	Simple_repeat
(GCAAGAA)N#Simple_repeat	82	1	X.parietina glXanPari1	Simple_repeat
(AAAG)N#Simple_repeat	81	3	X.parietina glXanPari1	Simple_repeat
(CAACCT)N#Simple_repeat	81	1	X.parietina glXanPari1	Simple_repeat
(CAGGA)N#Simple_repeat	80	2	X.parietina glXanPari1	Simple_repeat
(CCGAGA)N#Simple_repeat	80	2	X.parietina glXanPari1	Simple_repeat
(CCTCATC)N#Simple_repeat	80	1	X.parietina glXanPari1	Simple_repeat
(GAGGCA)N#Simple_repeat	80	2	X.parietina glXanPari1	Simple_repeat
(GTGGTT)N#Simple_repeat	80	2	X.parietina glXanPari1	Simple_repeat
(TCGCCT)N#Simple_repeat	80	2	X.parietina glXanPari1	Simple_repeat

(TTTGTTT)N#Simple_repeat	80	1	X.parietina glXanPari1	Simple_repeat
(CACAGA)N#Simple_repeat	79	1	X.parietina glXanPari1	Simple_repeat
(CCCCAA)N#Simple_repeat	79	2	X.parietina glXanPari1	Simple_repeat
(CTTCT)N#Simple_repeat	79	2	X.parietina glXanPari1	Simple_repeat
(GTGA)N#Simple_repeat	79	3	X.parietina glXanPari1	Simple_repeat
(GTGAGAG)N#Simple_repeat	79	1	X.parietina glXanPari1	Simple_repeat
(TCTTCCT)N#Simple_repeat	79	2	X.parietina glXanPari1	Simple_repeat
(AGCACC)N#Simple_repeat	78	2	X.parietina glXanPari1	Simple_repeat
(CACCAA)N#Simple_repeat	78	2	X.parietina glXanPari1	Simple_repeat
(CACCCA)N#Simple_repeat	78	2	X.parietina glXanPari1	Simple_repeat
(CATCAGCAG)N#Simple_repeat	78	1	X.parietina glXanPari1	Simple_repeat
(GAAGAT)N#Simple_repeat	78	2	X.parietina glXanPari1	Simple_repeat
(GGGA)N#Simple_repeat	78	3	X.parietina glXanPari1	Simple_repeat
(TCAC)N#Simple_repeat	78	3	X.parietina glXanPari1	Simple_repeat
(ATATA)N#Simple_repeat	77	2	X.parietina glXanPari1	Simple_repeat
(CCATCT)N#Simple_repeat	77	2	X.parietina glXanPari1	Simple_repeat
(CCCTTC)N#Simple_repeat	77	1	X.parietina glXanPari1	Simple_repeat
(GGAA)N#Simple_repeat	77	3	X.parietina glXanPari1	Simple_repeat
(GTGGAGG)N#Simple_repeat	77	1	X.parietina glXanPari1	Simple_repeat
(TTTATA)N#Simple_repeat	77	1	X.parietina glXanPari1	Simple_repeat
(CTCCCT)N#Simple_repeat	76	1	X.parietina glXanPari1	Simple_repeat
(CTCTC)N#Simple_repeat	76	2	X.parietina glXanPari1	Simple_repeat
(GCCGAA)N#Simple_repeat	76	2	X.parietina glXanPari1	Simple_repeat
(TAATAA)N#Simple_repeat	76	1	X.parietina glXanPari1	Simple_repeat
(TCCTTTC)N#Simple_repeat	76	1	X.parietina glXanPari1	Simple_repeat
(TCTCCG)N#Simple_repeat	76	1	X.parietina glXanPari1	Simple_repeat

(TCTGGC)N#Simple_repeat	76	2	X.parietina glXanPari1	Simple_repeat
(TCTTC)N#Simple_repeat	76	2	X.parietina glXanPari1	Simple_repeat
(TGCATAA)N#Simple_repeat	76	1	X.parietina glXanPari1	Simple_repeat
(TGGAGC)N#Simple_repeat	76	2	X.parietina glXanPari1	Simple_repeat
(TGTGTGTA)N#Simple_repeat	76	1	X.parietina glXanPari1	Simple_repeat
(TTCGCC)N#Simple_repeat	76	2	X.parietina glXanPari1	Simple_repeat
(TTCTA)N#Simple_repeat	76	1	X.parietina glXanPari1	Simple_repeat
(CATCCC)N#Simple_repeat	75	2	X.parietina glXanPari1	Simple_repeat
(GTCCTC)N#Simple_repeat	75	2	X.parietina glXanPari1	Simple_repeat
(TACATCT)N#Simple_repeat	75	1	X.parietina glXanPari1	Simple_repeat
(TTTGAT)N#Simple_repeat	75	2	X.parietina glXanPari1	Simple_repeat
(ACAT)N#Simple_repeat	74	2	X.parietina glXanPari1	Simple_repeat
(ACCCCGC)N#Simple_repeat	74	1	X.parietina glXanPari1	Simple_repeat
(CGCTAC)N#Simple_repeat	74	1	X.parietina glXanPari1	Simple_repeat
(GGATTT)N#Simple_repeat	74	1	X.parietina glXanPari1	Simple_repeat
(TGGGTGGA)N#Simple_repeat	74	2	X.parietina glXanPari1	Simple_repeat
(TGTGGT)N#Simple_repeat	74	1	X.parietina glXanPari1	Simple_repeat
(ACCCCCT)N#Simple_repeat	73	1	X.parietina glXanPari1	Simple_repeat
(ATCTTCC)N#Simple_repeat	73	1	X.parietina glXanPari1	Simple_repeat
(CCACTT)N#Simple_repeat	73	2	X.parietina glXanPari1	Simple_repeat
(TTTTCG)N#Simple_repeat	73	2	X.parietina glXanPari1	Simple_repeat
(ACCCCCG)N#Simple_repeat	72	1	X.parietina glXanPari1	Simple_repeat
(CCACCA)N#Simple_repeat	72	1	X.parietina glXanPari1	Simple_repeat
(CCATCAA)N#Simple_repeat	72	1	X.parietina glXanPari1	Simple_repeat
(CCCTTTC)N#Simple_repeat	72	1	X.parietina glXanPari1	Simple_repeat
(CGAATC)N#Simple_repeat	72	2	X.parietina glXanPari1	Simple_repeat

(CGAGGA)N#Simple_repeat	72	2	X.parietina glXanPari1	Simple_repeat
(CTCGTC)N#Simple_repeat	72	2	X.parietina glXanPari1	Simple_repeat
(GAACCA)N#Simple_repeat	72	2	X.parietina glXanPari1	Simple_repeat
(GAATCA)N#Simple_repeat	72	2	X.parietina glXanPari1	Simple_repeat
(GCCATC)N#Simple_repeat	72	2	X.parietina glXanPari1	Simple_repeat
(TGTT)N#Simple_repeat	72	3	X.parietina glXanPari1	Simple_repeat
(TTATAA)N#Simple_repeat	72	1	X.parietina glXanPari1	Simple_repeat
(AAGATC)N#Simple_repeat	71	1	X.parietina glXanPari1	Simple_repeat
(ATCCCCC)N#Simple_repeat	71	1	X.parietina glXanPari1	Simple_repeat
(CATCCCT)N#Simple_repeat	71	1	X.parietina glXanPari1	Simple_repeat
(TGCA)N#Simple_repeat	71	3	X.parietina glXanPari1	Simple_repeat
(TTCCC)N#Simple_repeat	71	2	X.parietina glXanPari1	Simple_repeat
(TTGCGT)N#Simple_repeat	71	1	X.parietina glXanPari1	Simple_repeat
(ACACCA)N#Simple_repeat	70	2	X.parietina glXanPari1	Simple_repeat
(CCAACAT)N#Simple_repeat	70	1	X.parietina glXanPari1	Simple_repeat
(CGCCAA)N#Simple_repeat	70	2	X.parietina glXanPari1	Simple_repeat
(CTCCTG)N#Simple_repeat	70	2	X.parietina glXanPari1	Simple_repeat
(GAAGGAGGA)N#Simple_repeat	70	1	X.parietina glXanPari1	Simple_repeat
(GGCAATGA)N#Simple_repeat	70	1	X.parietina glXanPari1	Simple_repeat
(TTGCTG)N#Simple_repeat	70	2	X.parietina glXanPari1	Simple_repeat
(AAAGGGG)N#Simple_repeat	69	1	X.parietina glXanPari1	Simple_repeat
(CAGCCT)N#Simple_repeat	69	1	X.parietina glXanPari1	Simple_repeat
(GAGAG)N#Simple_repeat	69	1	X.parietina glXanPari1	Simple_repeat
(GAGTCGGA)N#Simple_repeat	69	1	X.parietina glXanPari1	Simple_repeat
(GGAAA)N#Simple_repeat	69	2	X.parietina glXanPari1	Simple_repeat
(TCCCTT)N#Simple_repeat	69	1	X.parietina glXanPari1	Simple_repeat

(TCTCGC)N#Simple_repeat	69	1	X.parietina glXanPari1	Simple_repeat
(TTCT)N#Simple_repeat	69	3	X.parietina glXanPari1	Simple_repeat
(AGATG)N#Simple_repeat	68	2	X.parietina glXanPari1	Simple_repeat
(ATCCTC)N#Simple_repeat	68	1	X.parietina glXanPari1	Simple_repeat
(CACCA)N#Simple_repeat	68	2	X.parietina glXanPari1	Simple_repeat
(CGATCCT)N#Simple_repeat	68	1	X.parietina glXanPari1	Simple_repeat
(CGGAGA)N#Simple_repeat	68	1	X.parietina glXanPari1	Simple_repeat
(GAGGAG)N#Simple_repeat	68	1	X.parietina glXanPari1	Simple_repeat
(GCATCA)N#Simple_repeat	68	2	X.parietina glXanPari1	Simple_repeat
(GGCAGT)N#Simple_repeat	68	1	X.parietina glXanPari1	Simple_repeat
(GGTGAC)N#Simple_repeat	68	1	X.parietina glXanPari1	Simple_repeat
(TCCGCG)N#Simple_repeat	68	2	X.parietina glXanPari1	Simple_repeat
(TCTAGC)N#Simple_repeat	68	1	X.parietina glXanPari1	Simple_repeat
(TTCTGTTA)N#Simple_repeat	68	1	X.parietina glXanPari1	Simple_repeat
(AACC)N#Simple_repeat	67	2	X.parietina glXanPari1	Simple_repeat
(AAGAAC)N#Simple_repeat	67	2	X.parietina glXanPari1	Simple_repeat
(ATTTATT)N#Simple_repeat	67	1	X.parietina glXanPari1	Simple_repeat
(CCCCCG)N#Simple_repeat	67	2	X.parietina glXanPari1	Simple_repeat
(CTTCC)N#Simple_repeat	67	1	X.parietina glXanPari1	Simple_repeat
(TCTC)N#Simple_repeat	67	1	X.parietina glXanPari1	Simple_repeat
(AGCAAC)N#Simple_repeat	66	2	X.parietina glXanPari1	Simple_repeat
(ATGATG)N#Simple_repeat	66	1	X.parietina glXanPari1	Simple_repeat
(CGGCTT)N#Simple_repeat	66	2	X.parietina glXanPari1	Simple_repeat
(GGAAGT)N#Simple_repeat	66	1	X.parietina glXanPari1	Simple_repeat
(GTTGTT)N#Simple_repeat	66	1	X.parietina glXanPari1	Simple_repeat
(TGCCCTCG)N#Simple_repeat	66	1	X.parietina glXanPari1	Simple_repeat

(GGGATG)N#Simple_repeat	65	2	X.parietina glXanPari1	Simple_repeat
(TCTTCCA)N#Simple_repeat	65	1	X.parietina glXanPari1	Simple_repeat
(TTCTGC)N#Simple_repeat	65	2	X.parietina glXanPari1	Simple_repeat
(TTCTT)N#Simple_repeat	65	2	X.parietina glXanPari1	Simple_repeat
(AGCAGA)N#Simple_repeat	64	2	X.parietina glXanPari1	Simple_repeat
(AGCATC)N#Simple_repeat	64	1	X.parietina glXanPari1	Simple_repeat
(ATTA)N#Simple_repeat	64	2	X.parietina glXanPari1	Simple_repeat
(CCAACGC)N#Simple_repeat	64	1	X.parietina glXanPari1	Simple_repeat
(CCATC)N#Simple_repeat	64	2	X.parietina glXanPari1	Simple_repeat
(GCGA)N#Simple_repeat	64	2	X.parietina glXanPari1	Simple_repeat
(GGAGG)N#Simple_repeat	64	2	X.parietina glXanPari1	Simple_repeat
(GGGAG)N#Simple_repeat	64	2	X.parietina glXanPari1	Simple_repeat
(TTCCT)N#Simple_repeat	64	1	X.parietina glXanPari1	Simple_repeat
(AATACC)N#Simple_repeat	63	1	X.parietina glXanPari1	Simple_repeat
(ACGGTG)N#Simple_repeat	63	1	X.parietina glXanPari1	Simple_repeat
(CCAA)N#Simple_repeat	63	2	X.parietina glXanPari1	Simple_repeat
(GGCGGA)N#Simple_repeat	63	2	X.parietina glXanPari1	Simple_repeat
(TCCTAA)N#Simple_repeat	63	1	X.parietina glXanPari1	Simple_repeat
(TCTCT)N#Simple_repeat	63	2	X.parietina glXanPari1	Simple_repeat
(TTTCG)N#Simple_repeat	63	1	X.parietina glXanPari1	Simple_repeat
(AACGCCAG)N#Simple_repeat	62	1	X.parietina glXanPari1	Simple_repeat
(ACCTC)N#Simple_repeat	62	1	X.parietina glXanPari1	Simple_repeat
(CTGCC)N#Simple_repeat	62	1	X.parietina glXanPari1	Simple_repeat
(GACTCC)N#Simple_repeat	62	1	X.parietina glXanPari1	Simple_repeat
(GAGAGC)N#Simple_repeat	62	2	X.parietina glXanPari1	Simple_repeat
(GCGAGG)N#Simple_repeat	62	1	X.parietina glXanPari1	Simple_repeat

(GTACGGA)N#Simple_repeat	62	1	X.parietina glXanPari1	Simple_repeat
(GTGGAA)N#Simple_repeat	62	2	X.parietina glXanPari1	Simple_repeat
(TCCAA)N#Simple_repeat	62	1	X.parietina glXanPari1	Simple_repeat
(TTATTAT)N#Simple_repeat	62	1	X.parietina glXanPari1	Simple_repeat
(AGATA)N#Simple_repeat	61	2	X.parietina glXanPari1	Simple_repeat
(CAGCGA)N#Simple_repeat	61	1	X.parietina glXanPari1	Simple_repeat
(CTACCTA)N#Simple_repeat	61	1	X.parietina glXanPari1	Simple_repeat
(CTCCCCA)N#Simple_repeat	61	1	X.parietina glXanPari1	Simple_repeat
(CTGAAT)N#Simple_repeat	61	1	X.parietina glXanPari1	Simple_repeat
(GCCGGA)N#Simple_repeat	61	1	X.parietina glXanPari1	Simple_repeat
(GCGTCA)N#Simple_repeat	61	2	X.parietina glXanPari1	Simple_repeat
(GGGAAG)N#Simple_repeat	61	1	X.parietina glXanPari1	Simple_repeat
(TCCCAA)N#Simple_repeat	61	1	X.parietina glXanPari1	Simple_repeat
(TGTGT)N#Simple_repeat	61	2	X.parietina glXanPari1	Simple_repeat
(ACAA)N#Simple_repeat	60	1	X.parietina glXanPari1	Simple_repeat
(CCACCC)N#Simple_repeat	60	1	X.parietina glXanPari1	Simple_repeat
(CCTCT)N#Simple_repeat	60	2	X.parietina glXanPari1	Simple_repeat
(CTATT)N#Simple_repeat	60	1	X.parietina glXanPari1	Simple_repeat
(CTCAACC)N#Simple_repeat	60	1	X.parietina glXanPari1	Simple_repeat
(TCAAT)N#Simple_repeat	60	2	X.parietina glXanPari1	Simple_repeat
(TCCCCTCCA)N#Simple_repeat	60	1	X.parietina glXanPari1	Simple_repeat
(TTCCCCT)N#Simple_repeat	60	1	X.parietina glXanPari1	Simple_repeat
(TTGGCG)N#Simple_repeat	60	1	X.parietina glXanPari1	Simple_repeat
(TTTGG)N#Simple_repeat	60	2	X.parietina glXanPari1	Simple_repeat
(ACCCCCA)N#Simple_repeat	59	1	X.parietina glXanPari1	Simple_repeat
(ACCCCCC)N#Simple_repeat	59	1	X.parietina glXanPari1	Simple_repeat

(CGCCTCCGT)N#Simple_repeat	59	1	X.parietina glXanPari1	Simple_repeat
(CTCTT)N#Simple_repeat	59	1	X.parietina glXanPari1	Simple_repeat
(GAGCAG)N#Simple_repeat	59	1	X.parietina glXanPari1	Simple_repeat
(GCGGTG)N#Simple_repeat	59	1	X.parietina glXanPari1	Simple_repeat
(GGTT)N#Simple_repeat	59	2	X.parietina glXanPari1	Simple_repeat
(TATAGA)N#Simple_repeat	59	2	X.parietina glXanPari1	Simple_repeat
(TCACTCA)N#Simple_repeat	59	1	X.parietina glXanPari1	Simple_repeat
(TTTAA)N#Simple_repeat	59	1	X.parietina glXanPari1	Simple_repeat
(TTTTT)N#Simple_repeat	59	1	X.parietina glXanPari1	Simple_repeat
(ACGGTGCGGA)N#Simple_repeat	58	1	X.parietina glXanPari1	Simple_repeat
(AGGAAAG)N#Simple_repeat	58	1	X.parietina glXanPari1	Simple_repeat
(CAGTACGAG)N#Simple_repeat	58	1	X.parietina glXanPari1	Simple_repeat
(CCACACCA)N#Simple_repeat	58	1	X.parietina glXanPari1	Simple_repeat
(CGATCT)N#Simple_repeat	58	1	X.parietina glXanPari1	Simple_repeat
(CGGAGC)N#Simple_repeat	58	1	X.parietina glXanPari1	Simple_repeat
(GCTCTCT)N#Simple_repeat	58	1	X.parietina glXanPari1	Simple_repeat
(GGAGGTC)N#Simple_repeat	58	1	X.parietina glXanPari1	Simple_repeat
(GTTGTC)N#Simple_repeat	58	1	X.parietina glXanPari1	Simple_repeat
(TATAAT)N#Simple_repeat	58	1	X.parietina glXanPari1	Simple_repeat
(TATC)N#Simple_repeat	58	1	X.parietina glXanPari1	Simple_repeat
(TGGAGCG)N#Simple_repeat	58	1	X.parietina glXanPari1	Simple_repeat
(ATAGA)N#Simple_repeat	57	1	X.parietina glXanPari1	Simple_repeat
(ATATTTA)N#Simple_repeat	57	1	X.parietina glXanPari1	Simple_repeat
(ATGGT)N#Simple_repeat	57	2	X.parietina glXanPari1	Simple_repeat
(ATTATG)N#Simple_repeat	57	1	X.parietina glXanPari1	Simple_repeat
(CCTCCAC)N#Simple_repeat	57	1	X.parietina glXanPari1	Simple_repeat

(CCTCCG)N#Simple_repeat	57	1	X.parietina glXanPari1	Simple_repeat
(CGGCCT)N#Simple_repeat	57	1	X.parietina glXanPari1	Simple_repeat
(GAAGCG)N#Simple_repeat	57	1	X.parietina glXanPari1	Simple_repeat
(GCACTC)N#Simple_repeat	57	1	X.parietina glXanPari1	Simple_repeat
(GCAGA)N#Simple_repeat	57	2	X.parietina glXanPari1	Simple_repeat
(GGGGGA)N#Simple_repeat	57	2	X.parietina glXanPari1	Simple_repeat
(TAGCCC)N#Simple_repeat	57	1	X.parietina glXanPari1	Simple_repeat
(TCTCCCT)N#Simple_repeat	57	1	X.parietina glXanPari1	Simple_repeat
(TTTCT)N#Simple_repeat	57	2	X.parietina glXanPari1	Simple_repeat
(AAGGG)N#Simple_repeat	56	2	X.parietina glXanPari1	Simple_repeat
(ACAC)N#Simple_repeat	56	2	X.parietina glXanPari1	Simple_repeat
(AGCAGAG)N#Simple_repeat	56	1	X.parietina glXanPari1	Simple_repeat
(ATATAAT)N#Simple_repeat	56	1	X.parietina glXanPari1	Simple_repeat
(CGGAGG)N#Simple_repeat	56	1	X.parietina glXanPari1	Simple_repeat
(GAAA)N#Simple_repeat	56	2	X.parietina glXanPari1	Simple_repeat
(GAGCA)N#Simple_repeat	56	2	X.parietina glXanPari1	Simple_repeat
(TGAAG)N#Simple_repeat	56	1	X.parietina glXanPari1	Simple_repeat
(TTATTTA)N#Simple_repeat	56	1	X.parietina glXanPari1	Simple_repeat
(AGTGGTG)N#Simple_repeat	55	1	X.parietina glXanPari1	Simple_repeat
(CAGCAGCAA)N#Simple_repeat	55	1	X.parietina glXanPari1	Simple_repeat
(CTCTTTG)N#Simple_repeat	55	1	X.parietina glXanPari1	Simple_repeat
(CTTCCGT)N#Simple_repeat	55	1	X.parietina glXanPari1	Simple_repeat
(GGTGTG)N#Simple_repeat	55	1	X.parietina glXanPari1	Simple_repeat
(TATGATG)N#Simple_repeat	55	1	X.parietina glXanPari1	Simple_repeat
(TCTA)N#Simple_repeat	55	2	X.parietina glXanPari1	Simple_repeat
(ATGATTG)N#Simple_repeat	54	1	X.parietina glXanPari1	Simple_repeat

(CAGCTA)N#Simple_repeat	54	1	X.parietina glXanPari1	Simple_repeat
(CATTCAT)N#Simple_repeat	54	1	X.parietina glXanPari1	Simple_repeat
(CCAACT)N#Simple_repeat	54	1	X.parietina glXanPari1	Simple_repeat
(GGACA)N#Simple_repeat	54	1	X.parietina glXanPari1	Simple_repeat
(GGCGAC)N#Simple_repeat	54	1	X.parietina glXanPari1	Simple_repeat
(GGTAGCT)N#Simple_repeat	54	1	X.parietina glXanPari1	Simple_repeat
(TCCTTT)N#Simple_repeat	54	1	X.parietina glXanPari1	Simple_repeat
(TCTTTTCT)N#Simple_repeat	54	1	X.parietina glXanPari1	Simple_repeat
(TGAC)N#Simple_repeat	54	1	X.parietina glXanPari1	Simple_repeat
(TGACTG)N#Simple_repeat	54	1	X.parietina glXanPari1	Simple_repeat
(TTGAT)N#Simple_repeat	54	2	X.parietina glXanPari1	Simple_repeat
(TTGT)N#Simple_repeat	54	2	X.parietina glXanPari1	Simple_repeat
(TTTCTC)N#Simple_repeat	54	2	X.parietina glXanPari1	Simple_repeat
(AGGACC)N#Simple_repeat	53	1	X.parietina glXanPari1	Simple_repeat
(ATTT)N#Simple_repeat	53	2	X.parietina glXanPari1	Simple_repeat
(CAACCCCG)N#Simple_repeat	53	1	X.parietina glXanPari1	Simple_repeat
(CACC)N#Simple_repeat	53	2	X.parietina glXanPari1	Simple_repeat
(CCACAAG)N#Simple_repeat	53	1	X.parietina glXanPari1	Simple_repeat
(CCCCTTC)N#Simple_repeat	53	1	X.parietina glXanPari1	Simple_repeat
(CCTCTCC)N#Simple_repeat	53	1	X.parietina glXanPari1	Simple_repeat
(CGATCG)N#Simple_repeat	53	1	X.parietina glXanPari1	Simple_repeat
(GAGGTA)N#Simple_repeat	53	1	X.parietina glXanPari1	Simple_repeat
(GATGCAGCA)N#Simple_repeat	53	1	X.parietina glXanPari1	Simple_repeat
(GGCT)N#Simple_repeat	53	2	X.parietina glXanPari1	Simple_repeat
(GGTTTGG)N#Simple_repeat	53	1	X.parietina glXanPari1	Simple_repeat
(GTACA)N#Simple_repeat	53	2	X.parietina glXanPari1	Simple_repeat

(GTGGA)N#Simple_repeat	53	1	X.parietina glXanPari1	Simple_repeat
(TATAACT)N#Simple_repeat	53	1	X.parietina glXanPari1	Simple_repeat
(TTTTCTC)N#Simple_repeat	53	1	X.parietina glXanPari1	Simple_repeat
(ACCAA)N#Simple_repeat	52	1	X.parietina glXanPari1	Simple_repeat
(AGCTCTCC)N#Simple_repeat	52	1	X.parietina glXanPari1	Simple_repeat
(CATCACC)N#Simple_repeat	52	1	X.parietina glXanPari1	Simple_repeat
(CCAAA)N#Simple_repeat	52	1	X.parietina glXanPari1	Simple_repeat
(CCCTCCT)N#Simple_repeat	52	1	X.parietina glXanPari1	Simple_repeat
(CCTCCAT)N#Simple_repeat	52	1	X.parietina glXanPari1	Simple_repeat
(CTCTCCC)N#Simple_repeat	52	1	X.parietina glXanPari1	Simple_repeat
(GATCCA)N#Simple_repeat	52	1	X.parietina glXanPari1	Simple_repeat
(GCTTTG)N#Simple_repeat	52	1	X.parietina glXanPari1	Simple_repeat
(GGCAGAA)N#Simple_repeat	52	1	X.parietina glXanPari1	Simple_repeat
(GGTGAT)N#Simple_repeat	52	1	X.parietina glXanPari1	Simple_repeat
(TAGAC)N#Simple_repeat	52	1	X.parietina glXanPari1	Simple_repeat
(TGCTTGG)N#Simple_repeat	52	1	X.parietina glXanPari1	Simple_repeat
(TTCCTTT)N#Simple_repeat	52	1	X.parietina glXanPari1	Simple_repeat
RND-3_FAMILY-140#Unknown	52	2	X.parietina glXanPari1	Unknown
(AACCACC)N#Simple_repeat	51	1	X.parietina glXanPari1	Simple_repeat
(CCACA)N#Simple_repeat	51	2	X.parietina glXanPari1	Simple_repeat
(CGCAGC)N#Simple_repeat	51	1	X.parietina glXanPari1	Simple_repeat
(GAAT)N#Simple_repeat	51	2	X.parietina glXanPari1	Simple_repeat
(GAGAGG)N#Simple_repeat	51	1	X.parietina glXanPari1	Simple_repeat
(GCAGGAG)N#Simple_repeat	51	1	X.parietina glXanPari1	Simple_repeat
(GGCGCC)N#Simple_repeat	51	1	X.parietina glXanPari1	Simple_repeat
(GGTG)N#Simple_repeat	51	1	X.parietina glXanPari1	Simple_repeat

(TCCCTTCC)N#Simple_repeat	51	1	X.parietina glXanPari1	Simple_repeat
(TCTCCCA)N#Simple_repeat	51	1	X.parietina glXanPari1	Simple_repeat
(TGGTCGCT)N#Simple_repeat	51	1	X.parietina glXanPari1	Simple_repeat
(AACCAT)N#Simple_repeat	50	1	X.parietina glXanPari1	Simple_repeat
(ACACTCA)N#Simple_repeat	50	1	X.parietina glXanPari1	Simple_repeat
(AGTGTAG)N#Simple_repeat	50	1	X.parietina glXanPari1	Simple_repeat
(CAGCAG)N#Simple_repeat	50	1	X.parietina glXanPari1	Simple_repeat
(CCACTC)N#Simple_repeat	50	1	X.parietina glXanPari1	Simple_repeat
(CCAGCGG)N#Simple_repeat	50	1	X.parietina glXanPari1	Simple_repeat
(CCTCAA)N#Simple_repeat	50	1	X.parietina glXanPari1	Simple_repeat
(CCTCCCA)N#Simple_repeat	50	1	X.parietina glXanPari1	Simple_repeat
(CTCT)N#Simple_repeat	50	2	X.parietina glXanPari1	Simple_repeat
(GAGTCC)N#Simple_repeat	50	1	X.parietina glXanPari1	Simple_repeat
(GAGTT)N#Simple_repeat	50	2	X.parietina glXanPari1	Simple_repeat
(GCGGT)N#Simple_repeat	50	1	X.parietina glXanPari1	Simple_repeat
(GCTGATGAT)N#Simple_repeat	50	1	X.parietina glXanPari1	Simple_repeat
(GCTTGT)N#Simple_repeat	50	1	X.parietina glXanPari1	Simple_repeat
(GGCA)N#Simple_repeat	50	2	X.parietina glXanPari1	Simple_repeat
(GGCAAC)N#Simple_repeat	50	1	X.parietina glXanPari1	Simple_repeat
(TGCT)N#Simple_repeat	50	1	X.parietina glXanPari1	Simple_repeat
(TGGAGGC)N#Simple_repeat	50	1	X.parietina glXanPari1	Simple_repeat
(TTCAGGGG)N#Simple_repeat	50	1	X.parietina glXanPari1	Simple_repeat
(AGAATC)N#Simple_repeat	49	1	X.parietina glXanPari1	Simple_repeat
(CACCGTC)N#Simple_repeat	49	1	X.parietina glXanPari1	Simple_repeat
(CAGAGA)N#Simple_repeat	49	1	X.parietina glXanPari1	Simple_repeat
(CCACTAA)N#Simple_repeat	49	1	X.parietina glXanPari1	Simple_repeat

(CCAGCAG)N#Simple_repeat	49	1	X.parietina glXanPari1	Simple_repeat
(CCCACC)N#Simple_repeat	49	1	X.parietina glXanPari1	Simple_repeat
(CGGCGC)N#Simple_repeat	49	1	X.parietina glXanPari1	Simple_repeat
(CGTCCTTTGG)N#Simple_repeat	49	1	X.parietina glXanPari1	Simple_repeat
(CGTTGT)N#Simple_repeat	49	1	X.parietina glXanPari1	Simple_repeat
(CTTCCCT)N#Simple_repeat	49	1	X.parietina glXanPari1	Simple_repeat
(GCTT)N#Simple_repeat	49	2	X.parietina glXanPari1	Simple_repeat
(GGTTG)N#Simple_repeat	49	1	X.parietina glXanPari1	Simple_repeat
(TCCTCCT)N#Simple_repeat	49	1	X.parietina glXanPari1	Simple_repeat
(TGCCGC)N#Simple_repeat	49	1	X.parietina glXanPari1	Simple_repeat
(TGGGTGTG)N#Simple_repeat	49	1	X.parietina glXanPari1	Simple_repeat
(ACCTCTC)N#Simple_repeat	48	1	X.parietina glXanPari1	Simple_repeat
(AGAATGG)N#Simple_repeat	48	1	X.parietina glXanPari1	Simple_repeat
(AGACGAG)N#Simple_repeat	48	1	X.parietina glXanPari1	Simple_repeat
(ATGATGT)N#Simple_repeat	48	1	X.parietina glXanPari1	Simple_repeat
(CCTCCTA)N#Simple_repeat	48	1	X.parietina glXanPari1	Simple_repeat
(CGCTCGT)N#Simple_repeat	48	1	X.parietina glXanPari1	Simple_repeat
(CTTG)N#Simple_repeat	48	1	X.parietina glXanPari1	Simple_repeat
(GAGAGCA)N#Simple_repeat	48	1	X.parietina glXanPari1	Simple_repeat
(GATTG)N#Simple_repeat	48	1	X.parietina glXanPari1	Simple_repeat
(GGCTGA)N#Simple_repeat	48	1	X.parietina glXanPari1	Simple_repeat
(TCCTTCC)N#Simple_repeat	48	1	X.parietina glXanPari1	Simple_repeat
(TCGCA)N#Simple_repeat	48	1	X.parietina glXanPari1	Simple_repeat
(TGAGGC)N#Simple_repeat	48	1	X.parietina glXanPari1	Simple_repeat
(TGGCT)N#Simple_repeat	48	1	X.parietina glXanPari1	Simple_repeat
(TGGTATTGT)N#Simple_repeat	48	1	X.parietina glXanPari1	Simple_repeat

(TGGTCC)N#Simple_repeat	48	1	X.parietina glXanPari1	Simple_repeat
(AAGC)N#Simple_repeat	47	2	X.parietina glXanPari1	Simple_repeat
(ACTGTC)N#Simple_repeat	47	1	X.parietina glXanPari1	Simple_repeat
(AGTACTC)N#Simple_repeat	47	1	X.parietina glXanPari1	Simple_repeat
(AGTATCG)N#Simple_repeat	47	1	X.parietina glXanPari1	Simple_repeat
(ATACAT)N#Simple_repeat	47	1	X.parietina glXanPari1	Simple_repeat
(ATGAGA)N#Simple_repeat	47	1	X.parietina glXanPari1	Simple_repeat
(ATTCTC)N#Simple_repeat	47	1	X.parietina glXanPari1	Simple_repeat
(CAACCCT)N#Simple_repeat	47	1	X.parietina glXanPari1	Simple_repeat
(CAAG)N#Simple_repeat	47	1	X.parietina glXanPari1	Simple_repeat
(CCTCCTC)N#Simple_repeat	47	1	X.parietina glXanPari1	Simple_repeat
(CTCAAAT)N#Simple_repeat	47	1	X.parietina glXanPari1	Simple_repeat
(CTCTTC)N#Simple_repeat	47	1	X.parietina glXanPari1	Simple_repeat
(GAAG)N#Simple_repeat	47	2	X.parietina glXanPari1	Simple_repeat
(GGCATGGGA)N#Simple_repeat	47	1	X.parietina glXanPari1	Simple_repeat
(GGTTGGT)N#Simple_repeat	47	1	X.parietina glXanPari1	Simple_repeat
(TAGCAG)N#Simple_repeat	47	1	X.parietina glXanPari1	Simple_repeat
(TCCTC)N#Simple_repeat	47	1	X.parietina glXanPari1	Simple_repeat
(TCTCTT)N#Simple_repeat	47	1	X.parietina glXanPari1	Simple_repeat
(TGAACA)N#Simple_repeat	47	1	X.parietina glXanPari1	Simple_repeat
(TGCC)N#Simple_repeat	47	2	X.parietina glXanPari1	Simple_repeat
(TGTCTGA)N#Simple_repeat	47	1	X.parietina glXanPari1	Simple_repeat
POLYPURINE#Low_complexity	47	1	X.parietina glXanPari1	Low_complexity
(AAGTTGG)N#Simple_repeat	46	1	X.parietina glXanPari1	Simple_repeat
(ACCCCTA)N#Simple_repeat	46	1	X.parietina glXanPari1	Simple_repeat
(CACGA)N#Simple_repeat	46	1	X.parietina glXanPari1	Simple_repeat

(CAGAC)N#Simple_repeat	46	1	X.parietina glXanPari1	Simple_repeat
(CAGCACTAC)N#Simple_repeat	46	1	X.parietina glXanPari1	Simple_repeat
(CATCA)N#Simple_repeat	46	1	X.parietina glXanPari1	Simple_repeat
(CCCC)N#Simple_repeat	46	1	X.parietina glXanPari1	Simple_repeat
(CCGCAG)N#Simple_repeat	46	1	X.parietina glXanPari1	Simple_repeat
(CGAATTTT)N#Simple_repeat	46	1	X.parietina glXanPari1	Simple_repeat
(CGACTTCT)N#Simple_repeat	46	1	X.parietina glXanPari1	Simple_repeat
(CTCCA)N#Simple_repeat	46	1	X.parietina glXanPari1	Simple_repeat
(GCCTCTG)N#Simple_repeat	46	1	X.parietina glXanPari1	Simple_repeat
(GGACGT)N#Simple_repeat	46	1	X.parietina glXanPari1	Simple_repeat
(GTGTGG)N#Simple_repeat	46	1	X.parietina glXanPari1	Simple_repeat
(TAGTA)N#Simple_repeat	46	1	X.parietina glXanPari1	Simple_repeat
(TCCCAT)N#Simple_repeat	46	1	X.parietina glXanPari1	Simple_repeat
(TCCCCAC)N#Simple_repeat	46	1	X.parietina glXanPari1	Simple_repeat
(TGGTGG)N#Simple_repeat	46	1	X.parietina glXanPari1	Simple_repeat
(TTCCGCC)N#Simple_repeat	46	1	X.parietina glXanPari1	Simple_repeat
(TTGTGA)N#Simple_repeat	46	1	X.parietina glXanPari1	Simple_repeat
(AAGGTAG)N#Simple_repeat	45	1	X.parietina glXanPari1	Simple_repeat
(AATAAGA)N#Simple_repeat	45	1	X.parietina glXanPari1	Simple_repeat
(ACCACAA)N#Simple_repeat	45	1	X.parietina glXanPari1	Simple_repeat
(ACCATCG)N#Simple_repeat	45	1	X.parietina glXanPari1	Simple_repeat
(ACGGTC)N#Simple_repeat	45	1	X.parietina glXanPari1	Simple_repeat
(AGGGGA)N#Simple_repeat	45	1	X.parietina glXanPari1	Simple_repeat
(ATACT)N#Simple_repeat	45	1	X.parietina glXanPari1	Simple_repeat
(ATAGTAT)N#Simple_repeat	45	1	X.parietina glXanPari1	Simple_repeat
(CACACCC)N#Simple_repeat	45	1	X.parietina glXanPari1	Simple_repeat

(CACCCGCT)N#Simple_repeat	45	1	X.parietina glXanPari1	Simple_repeat
(CAGCCA)N#Simple_repeat	45	1	X.parietina glXanPari1	Simple_repeat
(CCTCCCCCT)N#Simple_repeat	45	1	X.parietina glXanPari1	Simple_repeat
(CCTGAAC)N#Simple_repeat	45	1	X.parietina glXanPari1	Simple_repeat
(GACCCA)N#Simple_repeat	45	1	X.parietina glXanPari1	Simple_repeat
(GGTGTA)N#Simple_repeat	45	1	X.parietina glXanPari1	Simple_repeat
(TAGG)N#Simple_repeat	45	2	X.parietina glXanPari1	Simple_repeat
(TATATAA)N#Simple_repeat	45	1	X.parietina glXanPari1	Simple_repeat
(TCTTGTA)N#Simple_repeat	45	1	X.parietina glXanPari1	Simple_repeat
(TGAGATGG)N#Simple_repeat	45	1	X.parietina glXanPari1	Simple_repeat
(TTCTGTG)N#Simple_repeat	45	1	X.parietina glXanPari1	Simple_repeat
(TTGAGAT)N#Simple_repeat	45	1	X.parietina glXanPari1	Simple_repeat
(TTTCATC)N#Simple_repeat	45	1	X.parietina glXanPari1	Simple_repeat
(TTTTAAT)N#Simple_repeat	45	1	X.parietina glXanPari1	Simple_repeat
(AATG)N#Simple_repeat	44	2	X.parietina glXanPari1	Simple_repeat
(ACCATCATT)N#Simple_repeat	44	1	X.parietina glXanPari1	Simple_repeat
(AGGAGC)N#Simple_repeat	44	1	X.parietina glXanPari1	Simple_repeat
(ATCTGGC)N#Simple_repeat	44	1	X.parietina glXanPari1	Simple_repeat
(ATGCTAA)N#Simple_repeat	44	1	X.parietina glXanPari1	Simple_repeat
(ATTCCC)N#Simple_repeat	44	1	X.parietina glXanPari1	Simple_repeat
(CAATC)N#Simple_repeat	44	1	X.parietina glXanPari1	Simple_repeat
(CACACC)N#Simple_repeat	44	1	X.parietina glXanPari1	Simple_repeat
(CAGCCAG)N#Simple_repeat	44	1	X.parietina glXanPari1	Simple_repeat
(CCACAGCC)N#Simple_repeat	44	1	X.parietina glXanPari1	Simple_repeat
(CCCCCAT)N#Simple_repeat	44	1	X.parietina glXanPari1	Simple_repeat
(CCCCCCT)N#Simple_repeat	44	1	X.parietina glXanPari1	Simple_repeat

(CCCCGA)N#Simple_repeat	44	1	X.parietina glXanPari1	Simple_repeat
(CCCTTT)N#Simple_repeat	44	1	X.parietina glXanPari1	Simple_repeat
(CCGCACC)N#Simple_repeat	44	1	X.parietina glXanPari1	Simple_repeat
(CTCGCC)N#Simple_repeat	44	1	X.parietina glXanPari1	Simple_repeat
(CTCGGT)N#Simple_repeat	44	1	X.parietina glXanPari1	Simple_repeat
(CTCTTCT)N#Simple_repeat	44	1	X.parietina glXanPari1	Simple_repeat
(CTTCAG)N#Simple_repeat	44	1	X.parietina glXanPari1	Simple_repeat
(CTTCTCA)N#Simple_repeat	44	1	X.parietina glXanPari1	Simple_repeat
(GAAGGGA)N#Simple_repeat	44	1	X.parietina glXanPari1	Simple_repeat
(GCTTC)N#Simple_repeat	44	1	X.parietina glXanPari1	Simple_repeat
(GGAATGA)N#Simple_repeat	44	1	X.parietina glXanPari1	Simple_repeat
(GTGACG)N#Simple_repeat	44	1	X.parietina glXanPari1	Simple_repeat
(GTTTTT)N#Simple_repeat	44	1	X.parietina glXanPari1	Simple_repeat
(TCAT)N#Simple_repeat	44	1	X.parietina glXanPari1	Simple_repeat
(TTTGGTG)N#Simple_repeat	44	1	X.parietina glXanPari1	Simple_repeat
(AAGTG)N#Simple_repeat	43	1	X.parietina glXanPari1	Simple_repeat
(ACCCCG)N#Simple_repeat	43	1	X.parietina glXanPari1	Simple_repeat
(AGACGG)N#Simple_repeat	43	1	X.parietina glXanPari1	Simple_repeat
(AGATGG)N#Simple_repeat	43	1	X.parietina glXanPari1	Simple_repeat
(ATCTCC)N#Simple_repeat	43	1	X.parietina glXanPari1	Simple_repeat
(ATTTCG)N#Simple_repeat	43	1	X.parietina glXanPari1	Simple_repeat
(CAACGC)N#Simple_repeat	43	1	X.parietina glXanPari1	Simple_repeat
(CAGAATC)N#Simple_repeat	43	1	X.parietina glXanPari1	Simple_repeat
(CCCATCC)N#Simple_repeat	43	1	X.parietina glXanPari1	Simple_repeat
(CCTCGA)N#Simple_repeat	43	1	X.parietina glXanPari1	Simple_repeat
(CGCCTG)N#Simple_repeat	43	1	X.parietina glXanPari1	Simple_repeat

(CTCATC)N#Simple_repeat	43	1	X.parietina glXanPari1	Simple_repeat
(CTCCCGA)N#Simple_repeat	43	1	X.parietina glXanPari1	Simple_repeat
(CTCCTTT)N#Simple_repeat	43	1	X.parietina glXanPari1	Simple_repeat
(CTTCTCTT)N#Simple_repeat	43	1	X.parietina glXanPari1	Simple_repeat
(GAACA)N#Simple_repeat	43	1	X.parietina glXanPari1	Simple_repeat
(GACTTGG)N#Simple_repeat	43	1	X.parietina glXanPari1	Simple_repeat
(GCTCAA)N#Simple_repeat	43	1	X.parietina glXanPari1	Simple_repeat
(GGAGCGA)N#Simple_repeat	43	1	X.parietina glXanPari1	Simple_repeat
(GGTTGTT)N#Simple_repeat	43	1	X.parietina glXanPari1	Simple_repeat
(GTAGCG)N#Simple_repeat	43	1	X.parietina glXanPari1	Simple_repeat
(GTCGCTG)N#Simple_repeat	43	1	X.parietina glXanPari1	Simple_repeat
(TATTA)N#Simple_repeat	43	1	X.parietina glXanPari1	Simple_repeat
(TCCACT)N#Simple_repeat	43	1	X.parietina glXanPari1	Simple_repeat
(TCGGCA)N#Simple_repeat	43	1	X.parietina glXanPari1	Simple_repeat
(TTTAATA)N#Simple_repeat	43	1	X.parietina glXanPari1	Simple_repeat
(TTTACT)N#Simple_repeat	43	1	X.parietina glXanPari1	Simple_repeat
(ATCCTCA)N#Simple_repeat	42	1	X.parietina glXanPari1	Simple_repeat
(ATGACT)N#Simple_repeat	42	1	X.parietina glXanPari1	Simple_repeat
(CAGAA)N#Simple_repeat	42	1	X.parietina glXanPari1	Simple_repeat
(CCTGC)N#Simple_repeat	42	1	X.parietina glXanPari1	Simple_repeat
(CGACCTCCT)N#Simple_repeat	42	1	X.parietina glXanPari1	Simple_repeat
(CTCCAAC)N#Simple_repeat	42	1	X.parietina glXanPari1	Simple_repeat
(CTGACGAAT)N#Simple_repeat	42	1	X.parietina glXanPari1	Simple_repeat
(GAGCCA)N#Simple_repeat	42	1	X.parietina glXanPari1	Simple_repeat
(GATCC)N#Simple_repeat	42	1	X.parietina glXanPari1	Simple_repeat
(GATGAT)N#Simple_repeat	42	1	X.parietina glXanPari1	Simple_repeat

(GCATGG)N#Simple_repeat	42	1	X.parietina glXanPari1	Simple_repeat
(GCCAAC)N#Simple_repeat	42	1	X.parietina glXanPari1	Simple_repeat
(GGAGGG)N#Simple_repeat	42	1	X.parietina glXanPari1	Simple_repeat
(GGGAGGAA)N#Simple_repeat	42	1	X.parietina glXanPari1	Simple_repeat
(GTAACCACC)N#Simple_repeat	42	1	X.parietina glXanPari1	Simple_repeat
(TCCGAG)N#Simple_repeat	42	1	X.parietina glXanPari1	Simple_repeat
(TCTACCC)N#Simple_repeat	42	1	X.parietina glXanPari1	Simple_repeat
(TCTTGTGCT)N#Simple_repeat	42	1	X.parietina glXanPari1	Simple_repeat
(TGGGCTCGG)N#Simple_repeat	42	1	X.parietina glXanPari1	Simple_repeat
(TGGTGA)N#Simple_repeat	42	1	X.parietina glXanPari1	Simple_repeat
(TTTCGACT)N#Simple_repeat	42	1	X.parietina glXanPari1	Simple_repeat
(TTTGCAC)N#Simple_repeat	42	1	X.parietina glXanPari1	Simple_repeat
(AGAGCGAA)N#Simple_repeat	41	1	X.parietina glXanPari1	Simple_repeat
(AGAGTAG)N#Simple_repeat	41	1	X.parietina glXanPari1	Simple_repeat
(AGATAGT)N#Simple_repeat	41	1	X.parietina glXanPari1	Simple_repeat
(AGGGACAGGG)N#Simple_repeat	41	1	X.parietina glXanPari1	Simple_repeat
(AGTACGG)N#Simple_repeat	41	1	X.parietina glXanPari1	Simple_repeat
(CAACAGCAA)N#Simple_repeat	41	1	X.parietina glXanPari1	Simple_repeat
(CACCGA)N#Simple_repeat	41	1	X.parietina glXanPari1	Simple_repeat
(CATCACGC)N#Simple_repeat	41	1	X.parietina glXanPari1	Simple_repeat
(CATCGC)N#Simple_repeat	41	1	X.parietina glXanPari1	Simple_repeat
(CATTGA)N#Simple_repeat	41	1	X.parietina glXanPari1	Simple_repeat
(CCAACAC)N#Simple_repeat	41	1	X.parietina glXanPari1	Simple_repeat
(CCCACCA)N#Simple_repeat	41	1	X.parietina glXanPari1	Simple_repeat
(CCCCTTT)N#Simple_repeat	41	1	X.parietina glXanPari1	Simple_repeat
(CCTA)N#Simple_repeat	41	2	X.parietina glXanPari1	Simple_repeat

(CCTTCT)N#Simple_repeat	41	1	X.parietina glXanPari1	Simple_repeat
(CCTTCTCC)N#Simple_repeat	41	1	X.parietina glXanPari1	Simple_repeat
(CTCACCA)N#Simple_repeat	41	1	X.parietina glXanPari1	Simple_repeat
(CTCTTCC)N#Simple_repeat	41	1	X.parietina glXanPari1	Simple_repeat
(GAGCGGA)N#Simple_repeat	41	1	X.parietina glXanPari1	Simple_repeat
(GATGA)N#Simple_repeat	41	1	X.parietina glXanPari1	Simple_repeat
(GCCCCTGA)N#Simple_repeat	41	1	X.parietina glXanPari1	Simple_repeat
(GGAGAA)N#Simple_repeat	41	2	X.parietina glXanPari1	Simple_repeat
(GGATTC)N#Simple_repeat	41	1	X.parietina glXanPari1	Simple_repeat
(GGGAGA)N#Simple_repeat	41	1	X.parietina glXanPari1	Simple_repeat
(GGGTCGAG)N#Simple_repeat	41	1	X.parietina glXanPari1	Simple_repeat
(GTCCAGT)N#Simple_repeat	41	1	X.parietina glXanPari1	Simple_repeat
(GTTCTCA)N#Simple_repeat	41	1	X.parietina glXanPari1	Simple_repeat
(TCACCG)N#Simple_repeat	41	1	X.parietina glXanPari1	Simple_repeat
(TCCACG)N#Simple_repeat	41	1	X.parietina glXanPari1	Simple_repeat
(TCCTGG)N#Simple_repeat	41	1	X.parietina glXanPari1	Simple_repeat
(TCGGTTT)N#Simple_repeat	41	1	X.parietina glXanPari1	Simple_repeat
(TCTCCCC)N#Simple_repeat	41	1	X.parietina glXanPari1	Simple_repeat
(TCTTCCTCT)N#Simple_repeat	41	1	X.parietina glXanPari1	Simple_repeat
(TGACGCT)N#Simple_repeat	41	1	X.parietina glXanPari1	Simple_repeat
(TGAGA)N#Simple_repeat	41	1	X.parietina glXanPari1	Simple_repeat
(TT)N#Simple_repeat	41	1	X.parietina glXanPari1	Simple_repeat
(AATGCA)N#Simple_repeat	40	1	X.parietina glXanPari1	Simple_repeat
(ACAATG)N#Simple_repeat	40	1	X.parietina glXanPari1	Simple_repeat
(ACCACCACCA)N#Simple_repeat	40	1	X.parietina glXanPari1	Simple_repeat
(ACGAGC)N#Simple_repeat	40	1	X.parietina glXanPari1	Simple_repeat

(AGAATA)N#Simple_repeat	40	1	X.parietina glXanPari1	Simple_repeat
(AGGTGA)N#Simple_repeat	40	1	X.parietina glXanPari1	Simple_repeat
(AGTAC)N#Simple_repeat	40	1	X.parietina glXanPari1	Simple_repeat
(ATCCATC)N#Simple_repeat	40	1	X.parietina glXanPari1	Simple_repeat
(ATTTGG)N#Simple_repeat	40	1	X.parietina glXanPari1	Simple_repeat
(CAACACC)N#Simple_repeat	40	1	X.parietina glXanPari1	Simple_repeat
(CACACAC)N#Simple_repeat	40	1	X.parietina glXanPari1	Simple_repeat
(CAGCACT)N#Simple_repeat	40	1	X.parietina glXanPari1	Simple_repeat
(CCACCTCCG)N#Simple_repeat	40	1	X.parietina glXanPari1	Simple_repeat
(CCCAAC)N#Simple_repeat	40	1	X.parietina glXanPari1	Simple_repeat
(CCGCGC)N#Simple_repeat	40	1	X.parietina glXanPari1	Simple_repeat
(CCTCAATCAA)N#Simple_repeat	40	1	X.parietina glXanPari1	Simple_repeat
(CCTCGC)N#Simple_repeat	40	1	X.parietina glXanPari1	Simple_repeat
(CCTTCGC)N#Simple_repeat	40	1	X.parietina glXanPari1	Simple_repeat
(CGAGAT)N#Simple_repeat	40	1	X.parietina glXanPari1	Simple_repeat
(CTCAT)N#Simple_repeat	40	1	X.parietina glXanPari1	Simple_repeat
(CTCCCCC)N#Simple_repeat	40	1	X.parietina glXanPari1	Simple_repeat
(CTCCTTC)N#Simple_repeat	40	1	X.parietina glXanPari1	Simple_repeat
(GAAGCAGATC)N#Simple_repeat	40	1	X.parietina glXanPari1	Simple_repeat
(GAGAC)N#Simple_repeat	40	1	X.parietina glXanPari1	Simple_repeat
(GATCTC)N#Simple_repeat	40	1	X.parietina glXanPari1	Simple_repeat
(GATGAAT)N#Simple_repeat	40	1	X.parietina glXanPari1	Simple_repeat
(GCACCA)N#Simple_repeat	40	1	X.parietina glXanPari1	Simple_repeat
(GCTTTT)N#Simple_repeat	40	1	X.parietina glXanPari1	Simple_repeat
(GGATTGAACG)N#Simple_repeat	40	1	X.parietina glXanPari1	Simple_repeat
(GGCTTG)N#Simple_repeat	40	1	X.parietina glXanPari1	Simple_repeat

(GGTTT)N#Simple_repeat	40	1	X.parietina glXanPari1	Simple_repeat
(GGTTTT)N#Simple_repeat	40	1	X.parietina glXanPari1	Simple_repeat
(GTGGCA)N#Simple_repeat	40	1	X.parietina glXanPari1	Simple_repeat
(TCTTGC)N#Simple_repeat	40	1	X.parietina glXanPari1	Simple_repeat
(TGCACC)N#Simple_repeat	40	1	X.parietina glXanPari1	Simple_repeat
(TGGATG)N#Simple_repeat	40	1	X.parietina glXanPari1	Simple_repeat
(TGTCGA)N#Simple_repeat	40	1	X.parietina glXanPari1	Simple_repeat
(TTGGTT)N#Simple_repeat	40	1	X.parietina glXanPari1	Simple_repeat
(AACAGC)N#Simple_repeat	39	1	X.parietina glXanPari1	Simple_repeat
(AACCAA)N#Simple_repeat	39	1	X.parietina glXanPari1	Simple_repeat
(AATCAACG)N#Simple_repeat	39	1	X.parietina glXanPari1	Simple_repeat
(ACATCC)N#Simple_repeat	39	1	X.parietina glXanPari1	Simple_repeat
(ACATCTG)N#Simple_repeat	39	1	X.parietina glXanPari1	Simple_repeat
(ACCACA)N#Simple_repeat	39	1	X.parietina glXanPari1	Simple_repeat
(ACCCA)N#Simple_repeat	39	1	X.parietina glXanPari1	Simple_repeat
(AGGAGT)N#Simple_repeat	39	1	X.parietina glXanPari1	Simple_repeat
(ATCTCAA)N#Simple_repeat	39	1	X.parietina glXanPari1	Simple_repeat
(ATCTTA)N#Simple_repeat	39	1	X.parietina glXanPari1	Simple_repeat
(ATTAG)N#Simple_repeat	39	1	X.parietina glXanPari1	Simple_repeat
(CAACGA)N#Simple_repeat	39	1	X.parietina glXanPari1	Simple_repeat
(CAGAAG)N#Simple_repeat	39	1	X.parietina glXanPari1	Simple_repeat
(CATCGT)N#Simple_repeat	39	1	X.parietina glXanPari1	Simple_repeat
(CCAGCT)N#Simple_repeat	39	1	X.parietina glXanPari1	Simple_repeat
(CCAGCTC)N#Simple_repeat	39	1	X.parietina glXanPari1	Simple_repeat
(CGCGG)N#Simple_repeat	39	1	X.parietina glXanPari1	Simple_repeat
(CTCGCGT)N#Simple_repeat	39	1	X.parietina glXanPari1	Simple_repeat

(CTCGTTGCT)N#Simple_repeat	39	1	X.parietina glXanPari1	Simple_repeat
(CTCTGCT)N#Simple_repeat	39	1	X.parietina glXanPari1	Simple_repeat
(CTGTGT)N#Simple_repeat	39	1	X.parietina glXanPari1	Simple_repeat
(GAATGAA)N#Simple_repeat	39	1	X.parietina glXanPari1	Simple_repeat
(GAGCATTA)N#Simple_repeat	39	1	X.parietina glXanPari1	Simple_repeat
(GCCACT)N#Simple_repeat	39	1	X.parietina glXanPari1	Simple_repeat
(GCTGGTTG)N#Simple_repeat	39	1	X.parietina glXanPari1	Simple_repeat
(GCTGT)N#Simple_repeat	39	1	X.parietina glXanPari1	Simple_repeat
(GGAGTGG)N#Simple_repeat	39	1	X.parietina glXanPari1	Simple_repeat
(GGCAG)N#Simple_repeat	39	1	X.parietina glXanPari1	Simple_repeat
(GGTTGAG)N#Simple_repeat	39	1	X.parietina glXanPari1	Simple_repeat
(GTA)N#Simple_repeat	39	2	X.parietina glXanPari1	Simple_repeat
(TATCAC)N#Simple_repeat	39	1	X.parietina glXanPari1	Simple_repeat
(TCAGCAG)N#Simple_repeat	39	1	X.parietina glXanPari1	Simple_repeat
(TCCCCTC)N#Simple_repeat	39	1	X.parietina glXanPari1	Simple_repeat
(TCTCTCCC)N#Simple_repeat	39	1	X.parietina glXanPari1	Simple_repeat
(TCTG)N#Simple_repeat	39	1	X.parietina glXanPari1	Simple_repeat
(TCTGCTC)N#Simple_repeat	39	1	X.parietina glXanPari1	Simple_repeat
(TCTTCT)N#Simple_repeat	39	1	X.parietina glXanPari1	Simple_repeat
(TGTGGA)N#Simple_repeat	39	1	X.parietina glXanPari1	Simple_repeat
(TTCTTGA)N#Simple_repeat	39	1	X.parietina glXanPari1	Simple_repeat
(TTGCTC)N#Simple_repeat	39	1	X.parietina glXanPari1	Simple_repeat
(TTGTCGT)N#Simple_repeat	39	1	X.parietina glXanPari1	Simple_repeat
(ACGATC)N#Simple_repeat	38	1	X.parietina glXanPari1	Simple_repeat
(ACGGCG)N#Simple_repeat	38	1	X.parietina glXanPari1	Simple_repeat
(AGGGTGG)N#Simple_repeat	38	1	X.parietina glXanPari1	Simple_repeat

(ATAG)N#Simple_repeat	38	1	X.parietina glXanPari1	Simple_repeat
(ATGTCC)N#Simple_repeat	38	1	X.parietina glXanPari1	Simple_repeat
(ATTGGC)N#Simple_repeat	38	1	X.parietina glXanPari1	Simple_repeat
(CAAGAA)N#Simple_repeat	38	1	X.parietina glXanPari1	Simple_repeat
(CACCCTC)N#Simple_repeat	38	1	X.parietina glXanPari1	Simple_repeat
(CCAGCG)N#Simple_repeat	38	1	X.parietina glXanPari1	Simple_repeat
(CCATG)N#Simple_repeat	38	1	X.parietina glXanPari1	Simple_repeat
(CCCCACA)N#Simple_repeat	38	1	X.parietina glXanPari1	Simple_repeat
(CCGCCA)N#Simple_repeat	38	1	X.parietina glXanPari1	Simple_repeat
(CGCC)N#Simple_repeat	38	1	X.parietina glXanPari1	Simple_repeat
(CGGCA)N#Simple_repeat	38	1	X.parietina glXanPari1	Simple_repeat
(CGTTTG)N#Simple_repeat	38	1	X.parietina glXanPari1	Simple_repeat
(CTTCGT)N#Simple_repeat	38	1	X.parietina glXanPari1	Simple_repeat
(CTTTCTC)N#Simple_repeat	38	1	X.parietina glXanPari1	Simple_repeat
(GCGATG)N#Simple_repeat	38	1	X.parietina glXanPari1	Simple_repeat
(GCGGCG)N#Simple_repeat	38	1	X.parietina glXanPari1	Simple_repeat
(GGACT)N#Simple_repeat	38	1	X.parietina glXanPari1	Simple_repeat
(GTCTATT)N#Simple_repeat	38	1	X.parietina glXanPari1	Simple_repeat
(TCAGCCC)N#Simple_repeat	38	1	X.parietina glXanPari1	Simple_repeat
(TCATCCA)N#Simple_repeat	38	1	X.parietina glXanPari1	Simple_repeat
(TCGTGG)N#Simple_repeat	38	1	X.parietina glXanPari1	Simple_repeat
(TCTCA)N#Simple_repeat	38	1	X.parietina glXanPari1	Simple_repeat
(TGAGAT)N#Simple_repeat	38	1	X.parietina glXanPari1	Simple_repeat
(TGAGGT)N#Simple_repeat	38	1	X.parietina glXanPari1	Simple_repeat
(TGGCAGA)N#Simple_repeat	38	1	X.parietina glXanPari1	Simple_repeat
(TTCCGC)N#Simple_repeat	38	1	X.parietina glXanPari1	Simple_repeat

(TTGATG)N#Simple_repeat	38	1	X.parietina glXanPari1	Simple_repeat
(TTTTCTG)N#Simple_repeat	38	1	X.parietina glXanPari1	Simple_repeat
(AAAAGAA)N#Simple_repeat	37	1	X.parietina glXanPari1	Simple_repeat
(ACTGACT)N#Simple_repeat	37	1	X.parietina glXanPari1	Simple_repeat
(ACTTCT)N#Simple_repeat	37	1	X.parietina glXanPari1	Simple_repeat
(AGGG)N#Simple_repeat	37	1	X.parietina glXanPari1	Simple_repeat
(AGTCACA)N#Simple_repeat	37	1	X.parietina glXanPari1	Simple_repeat
(ATAACGA)N#Simple_repeat	37	1	X.parietina glXanPari1	Simple_repeat
(ATTGATA)N#Simple_repeat	37	1	X.parietina glXanPari1	Simple_repeat
(ATTTT)N#Simple_repeat	37	1	X.parietina glXanPari1	Simple_repeat
(CAAGATCC)N#Simple_repeat	37	1	X.parietina glXanPari1	Simple_repeat
(CATGACT)N#Simple_repeat	37	1	X.parietina glXanPari1	Simple_repeat
(CCAACG)N#Simple_repeat	37	1	X.parietina glXanPari1	Simple_repeat
(CCAAT)N#Simple_repeat	37	1	X.parietina glXanPari1	Simple_repeat
(CCACGG)N#Simple_repeat	37	1	X.parietina glXanPari1	Simple_repeat
(CCACT)N#Simple_repeat	37	1	X.parietina glXanPari1	Simple_repeat
(CCAGAG)N#Simple_repeat	37	1	X.parietina glXanPari1	Simple_repeat
(CCCACCC)N#Simple_repeat	37	1	X.parietina glXanPari1	Simple_repeat
(CCCATT)N#Simple_repeat	37	1	X.parietina glXanPari1	Simple_repeat
(CCCTCA)N#Simple_repeat	37	1	X.parietina glXanPari1	Simple_repeat
(CGTAGAG)N#Simple_repeat	37	1	X.parietina glXanPari1	Simple_repeat
(CTACCAA)N#Simple_repeat	37	1	X.parietina glXanPari1	Simple_repeat
(CTTAG)N#Simple_repeat	37	1	X.parietina glXanPari1	Simple_repeat
(GAGGGCAAG)N#Simple_repeat	37	1	X.parietina glXanPari1	Simple_repeat
(GATATG)N#Simple_repeat	37	1	X.parietina glXanPari1	Simple_repeat
(GCTCAT)N#Simple_repeat	37	1	X.parietina glXanPari1	Simple_repeat

(GGACTT)N#Simple_repeat	37	1	X.parietina glXanPari1	Simple_repeat
(GGAGTGGT)N#Simple_repeat	37	1	X.parietina glXanPari1	Simple_repeat
(GGTAAG)N#Simple_repeat	37	1	X.parietina glXanPari1	Simple_repeat
(GTCGTA)N#Simple_repeat	37	1	X.parietina glXanPari1	Simple_repeat
(GTCTTTC)N#Simple_repeat	37	1	X.parietina glXanPari1	Simple_repeat
(TATTT)N#Simple_repeat	37	1	X.parietina glXanPari1	Simple_repeat
(TCACCA)N#Simple_repeat	37	1	X.parietina glXanPari1	Simple_repeat
(TCATA)N#Simple_repeat	37	1	X.parietina glXanPari1	Simple_repeat
(TCCTCCA)N#Simple_repeat	37	1	X.parietina glXanPari1	Simple_repeat
(TGAATAG)N#Simple_repeat	37	1	X.parietina glXanPari1	Simple_repeat
(TGATGA)N#Simple_repeat	37	1	X.parietina glXanPari1	Simple_repeat
(TGCAGTG)N#Simple_repeat	37	1	X.parietina glXanPari1	Simple_repeat
(TGCCTT)N#Simple_repeat	37	1	X.parietina glXanPari1	Simple_repeat
(TGGATGA)N#Simple_repeat	37	1	X.parietina glXanPari1	Simple_repeat
(TGGG)N#Simple_repeat	37	1	X.parietina glXanPari1	Simple_repeat
(TGGGTG)N#Simple_repeat	37	1	X.parietina glXanPari1	Simple_repeat
(TGTTT)N#Simple_repeat	37	1	X.parietina glXanPari1	Simple_repeat
(TTTTTCT)N#Simple_repeat	37	1	X.parietina glXanPari1	Simple_repeat
(TTTTTTG)N#Simple_repeat	37	1	X.parietina glXanPari1	Simple_repeat
(AAGATG)N#Simple_repeat	36	1	X.parietina glXanPari1	Simple_repeat
(ACCCTCC)N#Simple_repeat	36	1	X.parietina glXanPari1	Simple_repeat
(AGAGA)N#Simple_repeat	36	1	X.parietina glXanPari1	Simple_repeat
(AGAGGAGTA)N#Simple_repeat	36	1	X.parietina glXanPari1	Simple_repeat
(AGAGT)N#Simple_repeat	36	1	X.parietina glXanPari1	Simple_repeat
(AGCTCT)N#Simple_repeat	36	1	X.parietina glXanPari1	Simple_repeat
(AGGAA)N#Simple_repeat	36	1	X.parietina glXanPari1	Simple_repeat

(ATCAGC)N#Simple_repeat	36	1	X.parietina glXanPari1	Simple_repeat
(ATGATTC)N#Simple_repeat	36	1	X.parietina glXanPari1	Simple_repeat
(CAAGGC)N#Simple_repeat	36	1	X.parietina glXanPari1	Simple_repeat
(CACCGC)N#Simple_repeat	36	1	X.parietina glXanPari1	Simple_repeat
(CCCTTCA)N#Simple_repeat	36	1	X.parietina glXanPari1	Simple_repeat
(CGAGTT)N#Simple_repeat	36	1	X.parietina glXanPari1	Simple_repeat
(CGTGAG)N#Simple_repeat	36	1	X.parietina glXanPari1	Simple_repeat
(CGTTTTT)N#Simple_repeat	36	1	X.parietina glXanPari1	Simple_repeat
(CTCTCG)N#Simple_repeat	36	1	X.parietina glXanPari1	Simple_repeat
(CTGCCC)N#Simple_repeat	36	1	X.parietina glXanPari1	Simple_repeat
(CTTGACAAT)N#Simple_repeat	36	1	X.parietina glXanPari1	Simple_repeat
(CTTTTA)N#Simple_repeat	36	1	X.parietina glXanPari1	Simple_repeat
(GACCAG)N#Simple_repeat	36	1	X.parietina glXanPari1	Simple_repeat
(GCAT)N#Simple_repeat	36	1	X.parietina glXanPari1	Simple_repeat
(GCTAT)N#Simple_repeat	36	1	X.parietina glXanPari1	Simple_repeat
(GCTCGAA)N#Simple_repeat	36	1	X.parietina glXanPari1	Simple_repeat
(GGCCACT)N#Simple_repeat	36	1	X.parietina glXanPari1	Simple_repeat
(GGTCCA)N#Simple_repeat	36	1	X.parietina glXanPari1	Simple_repeat
(GTGATT)N#Simple_repeat	36	1	X.parietina glXanPari1	Simple_repeat
(GTTGA)N#Simple_repeat	36	1	X.parietina glXanPari1	Simple_repeat
(TCTAC)N#Simple_repeat	36	1	X.parietina glXanPari1	Simple_repeat
(TCTTGG)N#Simple_repeat	36	1	X.parietina glXanPari1	Simple_repeat
(TGAGG)N#Simple_repeat	36	1	X.parietina glXanPari1	Simple_repeat
(TGCAGC)N#Simple_repeat	36	1	X.parietina glXanPari1	Simple_repeat
(TGCTCA)N#Simple_repeat	36	1	X.parietina glXanPari1	Simple_repeat
(TGTTGTTGG)N#Simple_repeat	36	1	X.parietina glXanPari1	Simple_repeat

(AACAAC)N#Simple_repeat	35	1	X.parietina glXanPari1	Simple_repeat
(AACGAC)N#Simple_repeat	35	1	X.parietina glXanPari1	Simple_repeat
(ATAC)N#Simple_repeat	35	1	X.parietina glXanPari1	Simple_repeat
(ATCGGA)N#Simple_repeat	35	1	X.parietina glXanPari1	Simple_repeat
(CACGAG)N#Simple_repeat	35	1	X.parietina glXanPari1	Simple_repeat
(CATCCAC)N#Simple_repeat	35	1	X.parietina glXanPari1	Simple_repeat
(CCCCATT)N#Simple_repeat	35	1	X.parietina glXanPari1	Simple_repeat
(CCCCTCT)N#Simple_repeat	35	1	X.parietina glXanPari1	Simple_repeat
(CCGCTC)N#Simple_repeat	35	1	X.parietina glXanPari1	Simple_repeat
(CCTAGC)N#Simple_repeat	35	1	X.parietina glXanPari1	Simple_repeat
(CCTCGTT)N#Simple_repeat	35	1	X.parietina glXanPari1	Simple_repeat
(CCTTA)N#Simple_repeat	35	1	X.parietina glXanPari1	Simple_repeat
(CTCA)N#Simple_repeat	35	1	X.parietina glXanPari1	Simple_repeat
(CTTTCG)N#Simple_repeat	35	1	X.parietina glXanPari1	Simple_repeat
(GAAGTGT)N#Simple_repeat	35	1	X.parietina glXanPari1	Simple_repeat
(GAATGG)N#Simple_repeat	35	1	X.parietina glXanPari1	Simple_repeat
(GAATT)N#Simple_repeat	35	1	X.parietina glXanPari1	Simple_repeat
(GAGGACG)N#Simple_repeat	35	1	X.parietina glXanPari1	Simple_repeat
(GGCAA)N#Simple_repeat	35	1	X.parietina glXanPari1	Simple_repeat
(GGTAGA)N#Simple_repeat	35	1	X.parietina glXanPari1	Simple_repeat
(GTCTCA)N#Simple_repeat	35	1	X.parietina glXanPari1	Simple_repeat
(GTCTTC)N#Simple_repeat	35	1	X.parietina glXanPari1	Simple_repeat
(TATAG)N#Simple_repeat	35	1	X.parietina glXanPari1	Simple_repeat
(TATGT)N#Simple_repeat	35	1	X.parietina glXanPari1	Simple_repeat
(TCCGTC)N#Simple_repeat	35	1	X.parietina glXanPari1	Simple_repeat
(TCGTC)N#Simple_repeat	35	1	X.parietina glXanPari1	Simple_repeat

(TCTTCTT)N#Simple_repeat	35	1	X.parietina glXanPari1	Simple_repeat
(TCTTT)N#Simple_repeat	35	1	X.parietina glXanPari1	Simple_repeat
(TGAGCA)N#Simple_repeat	35	1	X.parietina glXanPari1	Simple_repeat
(TGGT)N#Simple_repeat	35	1	X.parietina glXanPari1	Simple_repeat
(TTCCCCC)N#Simple_repeat	35	1	X.parietina glXanPari1	Simple_repeat
(AACCCC)N#Simple_repeat	34	1	X.parietina glXanPari1	Simple_repeat
(AAGGGAA)N#Simple_repeat	34	1	X.parietina glXanPari1	Simple_repeat
(ACAAAA)N#Simple_repeat	34	1	X.parietina glXanPari1	Simple_repeat
(ACAGA)N#Simple_repeat	34	1	X.parietina glXanPari1	Simple_repeat
(ACCCAG)N#Simple_repeat	34	1	X.parietina glXanPari1	Simple_repeat
(ACGGG)N#Simple_repeat	34	1	X.parietina glXanPari1	Simple_repeat
(ACGTCT)N#Simple_repeat	34	1	X.parietina glXanPari1	Simple_repeat
(AGAACAC)N#Simple_repeat	34	1	X.parietina glXanPari1	Simple_repeat
(AGAGAGTG)N#Simple_repeat	34	1	X.parietina glXanPari1	Simple_repeat
(AGAGC)N#Simple_repeat	34	1	X.parietina glXanPari1	Simple_repeat
(AGATAT)N#Simple_repeat	34	1	X.parietina glXanPari1	Simple_repeat
(AGGGGG)N#Simple_repeat	34	1	X.parietina glXanPari1	Simple_repeat
(AGGTGG)N#Simple_repeat	34	1	X.parietina glXanPari1	Simple_repeat
(ATTAAG)N#Simple_repeat	34	1	X.parietina glXanPari1	Simple_repeat
(ATTAGG)N#Simple_repeat	34	1	X.parietina glXanPari1	Simple_repeat
(ATTATA)N#Simple_repeat	34	1	X.parietina glXanPari1	Simple_repeat
(CACA)N#Simple_repeat	34	1	X.parietina glXanPari1	Simple_repeat
(CACAT)N#Simple_repeat	34	1	X.parietina glXanPari1	Simple_repeat
(CACCCC)N#Simple_repeat	34	1	X.parietina glXanPari1	Simple_repeat
(CATCGG)N#Simple_repeat	34	1	X.parietina glXanPari1	Simple_repeat
(CCCTTTT)N#Simple_repeat	34	1	X.parietina glXanPari1	Simple_repeat

(CCTCTT)N#Simple_repeat	34	1	X.parietina glXanPari1	Simple_repeat
(CGAAAC)N#Simple_repeat	34	1	X.parietina glXanPari1	Simple_repeat
(CGTACTC)N#Simple_repeat	34	1	X.parietina glXanPari1	Simple_repeat
(GAAGGA)N#Simple_repeat	34	1	X.parietina glXanPari1	Simple_repeat
(GAATGA)N#Simple_repeat	34	1	X.parietina glXanPari1	Simple_repeat
(GACTGAT)N#Simple_repeat	34	1	X.parietina glXanPari1	Simple_repeat
(GACTTC)N#Simple_repeat	34	1	X.parietina glXanPari1	Simple_repeat
(GAGTA)N#Simple_repeat	34	1	X.parietina glXanPari1	Simple_repeat
(GATTTG)N#Simple_repeat	34	1	X.parietina glXanPari1	Simple_repeat
(GCACCG)N#Simple_repeat	34	1	X.parietina glXanPari1	Simple_repeat
(GCACCT)N#Simple_repeat	34	1	X.parietina glXanPari1	Simple_repeat
(GCTGCC)N#Simple_repeat	34	1	X.parietina glXanPari1	Simple_repeat
(GGACAG)N#Simple_repeat	34	1	X.parietina glXanPari1	Simple_repeat
(GGACTG)N#Simple_repeat	34	1	X.parietina glXanPari1	Simple_repeat
(GGCGGAG)N#Simple_repeat	34	1	X.parietina glXanPari1	Simple_repeat
(GTCGTC)N#Simple_repeat	34	1	X.parietina glXanPari1	Simple_repeat
(GTTTGGA)N#Simple_repeat	34	1	X.parietina glXanPari1	Simple_repeat
(TACTAT)N#Simple_repeat	34	1	X.parietina glXanPari1	Simple_repeat
(TAGA)N#Simple_repeat	34	1	X.parietina glXanPari1	Simple_repeat
(TCTGA)N#Simple_repeat	34	1	X.parietina glXanPari1	Simple_repeat
(TGCAT)N#Simple_repeat	34	1	X.parietina glXanPari1	Simple_repeat
(TTCTCC)N#Simple_repeat	34	1	X.parietina glXanPari1	Simple_repeat
(TTGATC)N#Simple_repeat	34	1	X.parietina glXanPari1	Simple_repeat
(TTTGCT)N#Simple_repeat	34	1	X.parietina glXanPari1	Simple_repeat
(AACTGG)N#Simple_repeat	33	1	X.parietina glXanPari1	Simple_repeat
(AAGGTG)N#Simple_repeat	33	1	X.parietina glXanPari1	Simple_repeat

(ACAGAA)N#Simple_repeat	33	1	X.parietina glXanPari1	Simple_repeat
(AGAGGC)N#Simple_repeat	33	1	X.parietina glXanPari1	Simple_repeat
(ATATCAA)N#Simple_repeat	33	1	X.parietina glXanPari1	Simple_repeat
(CAAGCGG)N#Simple_repeat	33	1	X.parietina glXanPari1	Simple_repeat
(CACGTTG)N#Simple_repeat	33	1	X.parietina glXanPari1	Simple_repeat
(CACT)N#Simple_repeat	33	1	X.parietina glXanPari1	Simple_repeat
(CCGAAG)N#Simple_repeat	33	1	X.parietina glXanPari1	Simple_repeat
(CGCATTC)N#Simple_repeat	33	1	X.parietina glXanPari1	Simple_repeat
(CGGTTA)N#Simple_repeat	33	1	X.parietina glXanPari1	Simple_repeat
(CGTCTT)N#Simple_repeat	33	1	X.parietina glXanPari1	Simple_repeat
(CTCCAGC)N#Simple_repeat	33	1	X.parietina glXanPari1	Simple_repeat
(GAAGGT)N#Simple_repeat	33	1	X.parietina glXanPari1	Simple_repeat
(GACAAC)N#Simple_repeat	33	1	X.parietina glXanPari1	Simple_repeat
(GACACC)N#Simple_repeat	33	1	X.parietina glXanPari1	Simple_repeat
(GATCGA)N#Simple_repeat	33	1	X.parietina glXanPari1	Simple_repeat
(GCATTT)N#Simple_repeat	33	1	X.parietina glXanPari1	Simple_repeat
(GCTGGA)N#Simple_repeat	33	1	X.parietina glXanPari1	Simple_repeat
(GGCGTC)N#Simple_repeat	33	1	X.parietina glXanPari1	Simple_repeat
(GGTGTT)N#Simple_repeat	33	1	X.parietina glXanPari1	Simple_repeat
(GGTTCT)N#Simple_repeat	33	1	X.parietina glXanPari1	Simple_repeat
(GTACT)N#Simple_repeat	33	1	X.parietina glXanPari1	Simple_repeat
(GTTGCT)N#Simple_repeat	33	1	X.parietina glXanPari1	Simple_repeat
(GTTGG)N#Simple_repeat	33	1	X.parietina glXanPari1	Simple_repeat
(TCCACCT)N#Simple_repeat	33	1	X.parietina glXanPari1	Simple_repeat
(TCTTCTCT)N#Simple_repeat	33	1	X.parietina glXanPari1	Simple_repeat
(TGAGAC)N#Simple_repeat	33	1	X.parietina glXanPari1	Simple_repeat

(TTACC)N#Simple_repeat	33	1	X.parietina glXanPari1	Simple_repeat
(TTATAAC)N#Simple_repeat	33	1	X.parietina glXanPari1	Simple_repeat
(TTCTTT)N#Simple_repeat	33	1	X.parietina glXanPari1	Simple_repeat
(TTGGTG)N#Simple_repeat	33	1	X.parietina glXanPari1	Simple_repeat
(TTGTCA)N#Simple_repeat	33	1	X.parietina glXanPari1	Simple_repeat
(TTGTTG)N#Simple_repeat	33	1	X.parietina glXanPari1	Simple_repeat
(ATCTATG)N#Simple_repeat	32	1	X.parietina glXanPari1	Simple_repeat
(CAATGG)N#Simple_repeat	32	1	X.parietina glXanPari1	Simple_repeat
(CAGTGG)N#Simple_repeat	32	1	X.parietina glXanPari1	Simple_repeat
(CATAAG)N#Simple_repeat	32	1	X.parietina glXanPari1	Simple_repeat
(CCTTCG)N#Simple_repeat	32	1	X.parietina glXanPari1	Simple_repeat
(CGATGGT)N#Simple_repeat	32	1	X.parietina glXanPari1	Simple_repeat
(CGGCAT)N#Simple_repeat	32	1	X.parietina glXanPari1	Simple_repeat
(CTGCCA)N#Simple_repeat	32	1	X.parietina glXanPari1	Simple_repeat
(GAGGTC)N#Simple_repeat	32	1	X.parietina glXanPari1	Simple_repeat
(GCAG)N#Simple_repeat	32	1	X.parietina glXanPari1	Simple_repeat
(GCGAAG)N#Simple_repeat	32	1	X.parietina glXanPari1	Simple_repeat
(GCGGAA)N#Simple_repeat	32	1	X.parietina glXanPari1	Simple_repeat
(GCTGTT)N#Simple_repeat	32	1	X.parietina glXanPari1	Simple_repeat
(GGATA)N#Simple_repeat	32	1	X.parietina glXanPari1	Simple_repeat
(GGGTCT)N#Simple_repeat	32	1	X.parietina glXanPari1	Simple_repeat
(GTGAGA)N#Simple_repeat	32	1	X.parietina glXanPari1	Simple_repeat
(GTGCG)N#Simple_repeat	32	1	X.parietina glXanPari1	Simple_repeat
(TCAACT)N#Simple_repeat	32	1	X.parietina glXanPari1	Simple_repeat
(TCGCCC)N#Simple_repeat	32	1	X.parietina glXanPari1	Simple_repeat
(TGAGAA)N#Simple_repeat	32	1	X.parietina glXanPari1	Simple_repeat

(TGAGTT)N#Simple_repeat	32	1	X.parietina glXanPari1	Simple_repeat
(TGGCCAT)N#Simple_repeat	32	1	X.parietina glXanPari1	Simple_repeat
(TGGCTG)N#Simple_repeat	32	1	X.parietina glXanPari1	Simple_repeat
(TGGGT)N#Simple_repeat	32	1	X.parietina glXanPari1	Simple_repeat
(TGTTGTGC)N#Simple_repeat	32	1	X.parietina glXanPari1	Simple_repeat
(TTCAC)N#Simple_repeat	32	1	X.parietina glXanPari1	Simple_repeat
(TTGTGG)N#Simple_repeat	32	1	X.parietina glXanPari1	Simple_repeat
(AGAAGG)N#Simple_repeat	31	1	X.parietina glXanPari1	Simple_repeat
(AGAGCG)N#Simple_repeat	31	1	X.parietina glXanPari1	Simple_repeat
(AGGTAG)N#Simple_repeat	31	1	X.parietina glXanPari1	Simple_repeat
(ATAAT)N#Simple_repeat	31	1	X.parietina glXanPari1	Simple_repeat
(ATCCCG)N#Simple_repeat	31	1	X.parietina glXanPari1	Simple_repeat
(ATGA)N#Simple_repeat	31	1	X.parietina glXanPari1	Simple_repeat
(ATTAAT)N#Simple_repeat	31	1	X.parietina glXanPari1	Simple_repeat
(CACCTT)N#Simple_repeat	31	1	X.parietina glXanPari1	Simple_repeat
(CATCT)N#Simple_repeat	31	1	X.parietina glXanPari1	Simple_repeat
(CCAATGA)N#Simple_repeat	31	1	X.parietina glXanPari1	Simple_repeat
(CCGCTT)N#Simple_repeat	31	1	X.parietina glXanPari1	Simple_repeat
(CCTCGG)N#Simple_repeat	31	1	X.parietina glXanPari1	Simple_repeat
(CCTGT)N#Simple_repeat	31	1	X.parietina glXanPari1	Simple_repeat
(CTCCAA)N#Simple_repeat	31	1	X.parietina glXanPari1	Simple_repeat
(CTCTTT)N#Simple_repeat	31	1	X.parietina glXanPari1	Simple_repeat
(CTGCT)N#Simple_repeat	31	1	X.parietina glXanPari1	Simple_repeat
(GACGG)N#Simple_repeat	31	1	X.parietina glXanPari1	Simple_repeat
(GAGGACT)N#Simple_repeat	31	1	X.parietina glXanPari1	Simple_repeat
(GATGCA)N#Simple_repeat	31	1	X.parietina glXanPari1	Simple_repeat

(GCAAGT)N#Simple_repeat	31	1	X.parietina glXanPari1	Simple_repeat
(GCGGCA)N#Simple_repeat	31	1	X.parietina glXanPari1	Simple_repeat
(GGGAAA)N#Simple_repeat	31	1	X.parietina glXanPari1	Simple_repeat
(GGTAT)N#Simple_repeat	31	1	X.parietina glXanPari1	Simple_repeat
(GGTCAA)N#Simple_repeat	31	1	X.parietina glXanPari1	Simple_repeat
(GGTGTC)N#Simple_repeat	31	1	X.parietina glXanPari1	Simple_repeat
(GTAGTC)N#Simple_repeat	31	1	X.parietina glXanPari1	Simple_repeat
(GTCGAT)N#Simple_repeat	31	1	X.parietina glXanPari1	Simple_repeat
(GTGTG)N#Simple_repeat	31	1	X.parietina glXanPari1	Simple_repeat
(TCATTT)N#Simple_repeat	31	1	X.parietina glXanPari1	Simple_repeat
(TCCGCC)N#Simple_repeat	31	1	X.parietina glXanPari1	Simple_repeat
(TCCGCT)N#Simple_repeat	31	1	X.parietina glXanPari1	Simple_repeat
(TCGGAA)N#Simple_repeat	31	1	X.parietina glXanPari1	Simple_repeat
(TGCTAT)N#Simple_repeat	31	1	X.parietina glXanPari1	Simple_repeat
(TGCTGG)N#Simple_repeat	31	1	X.parietina glXanPari1	Simple_repeat
(TGGAA)N#Simple_repeat	31	1	X.parietina glXanPari1	Simple_repeat
(TTACA)N#Simple_repeat	31	1	X.parietina glXanPari1	Simple_repeat
(AAAATAA)N#Simple_repeat	30	1	X.parietina glXanPari1	Simple_repeat
(AACCA)N#Simple_repeat	30	1	X.parietina glXanPari1	Simple_repeat
(AATCCC)N#Simple_repeat	30	1	X.parietina glXanPari1	Simple_repeat
(AATTG)N#Simple_repeat	30	1	X.parietina glXanPari1	Simple_repeat
(ACACAA)N#Simple_repeat	30	1	X.parietina glXanPari1	Simple_repeat
(AGAACG)N#Simple_repeat	30	1	X.parietina glXanPari1	Simple_repeat
(AGGTGC)N#Simple_repeat	30	1	X.parietina glXanPari1	Simple_repeat
(ATGGGG)N#Simple_repeat	30	1	X.parietina glXanPari1	Simple_repeat
(CAACAT)N#Simple_repeat	30	1	X.parietina glXanPari1	Simple_repeat

(CAGG)N#Simple_repeat	30	1	X.parietina glXanPari1	Simple_repeat
(CCATAC)N#Simple_repeat	30	1	X.parietina glXanPari1	Simple_repeat
(CCCCG)N#Simple_repeat	30	1	X.parietina glXanPari1	Simple_repeat
(CGAAG)N#Simple_repeat	30	1	X.parietina glXanPari1	Simple_repeat
(CGATGG)N#Simple_repeat	30	1	X.parietina glXanPari1	Simple_repeat
(CGTAA)N#Simple_repeat	30	1	X.parietina glXanPari1	Simple_repeat
(CTTTTTT)N#Simple_repeat	30	1	X.parietina glXanPari1	Simple_repeat
(GACGTG)N#Simple_repeat	30	1	X.parietina glXanPari1	Simple_repeat
(GCACGA)N#Simple_repeat	30	1	X.parietina glXanPari1	Simple_repeat
(GCTGA)N#Simple_repeat	30	1	X.parietina glXanPari1	Simple_repeat
(GGCCAA)N#Simple_repeat	30	1	X.parietina glXanPari1	Simple_repeat
(GGCGA)N#Simple_repeat	30	1	X.parietina glXanPari1	Simple_repeat
(GGCTTC)N#Simple_repeat	30	1	X.parietina glXanPari1	Simple_repeat
(GGCTTT)N#Simple_repeat	30	1	X.parietina glXanPari1	Simple_repeat
(TATAA)N#Simple_repeat	30	1	X.parietina glXanPari1	Simple_repeat
(TCTGAG)N#Simple_repeat	30	1	X.parietina glXanPari1	Simple_repeat
(TTTTTC)N#Simple_repeat	30	1	X.parietina glXanPari1	Simple_repeat
(AATCC)N#Simple_repeat	29	1	X.parietina glXanPari1	Simple_repeat
(ACCTT)N#Simple_repeat	29	1	X.parietina glXanPari1	Simple_repeat
(AGAA)N#Simple_repeat	29	1	X.parietina glXanPari1	Simple_repeat
(AGGGAAG)N#Simple_repeat	29	1	X.parietina glXanPari1	Simple_repeat
(ATATT)N#Simple_repeat	29	1	X.parietina glXanPari1	Simple_repeat
(ATCCCC)N#Simple_repeat	29	1	X.parietina glXanPari1	Simple_repeat
(ATCTC)N#Simple_repeat	29	1	X.parietina glXanPari1	Simple_repeat
(CACCCCT)N#Simple_repeat	29	1	X.parietina glXanPari1	Simple_repeat
(CCCAA)N#Simple_repeat	29	1	X.parietina glXanPari1	Simple_repeat

(CCCTA)N#Simple_repeat	29	1	X.parietina glXanPari1	Simple_repeat
(CGCCGT)N#Simple_repeat	29	1	X.parietina glXanPari1	Simple_repeat
(CTCCG)N#Simple_repeat	29	1	X.parietina glXanPari1	Simple_repeat
(CTCGAC)N#Simple_repeat	29	1	X.parietina glXanPari1	Simple_repeat
(CTCGGA)N#Simple_repeat	29	1	X.parietina glXanPari1	Simple_repeat
(CTCTGG)N#Simple_repeat	29	1	X.parietina glXanPari1	Simple_repeat
(CTGAACC)N#Simple_repeat	29	1	X.parietina glXanPari1	Simple_repeat
(GAATA)N#Simple_repeat	29	1	X.parietina glXanPari1	Simple_repeat
(GAATG)N#Simple_repeat	29	1	X.parietina glXanPari1	Simple_repeat
(GCAA)N#Simple_repeat	29	1	X.parietina glXanPari1	Simple_repeat
(GCGTGA)N#Simple_repeat	29	1	X.parietina glXanPari1	Simple_repeat
(GCTGCA)N#Simple_repeat	29	1	X.parietina glXanPari1	Simple_repeat
(GGATTG)N#Simple_repeat	29	1	X.parietina glXanPari1	Simple_repeat
(GGGAAGG)N#Simple_repeat	29	1	X.parietina glXanPari1	Simple_repeat
(GGGGA)N#Simple_repeat	29	1	X.parietina glXanPari1	Simple_repeat
(GGGGT)N#Simple_repeat	29	1	X.parietina glXanPari1	Simple_repeat
(GTCCAC)N#Simple_repeat	29	1	X.parietina glXanPari1	Simple_repeat
(GTGGGTG)N#Simple_repeat	29	1	X.parietina glXanPari1	Simple_repeat
(TAATT)N#Simple_repeat	29	1	X.parietina glXanPari1	Simple_repeat
(TAGAA)N#Simple_repeat	29	1	X.parietina glXanPari1	Simple_repeat
(TCCAGCC)N#Simple_repeat	29	1	X.parietina glXanPari1	Simple_repeat
(TCCTCA)N#Simple_repeat	29	1	X.parietina glXanPari1	Simple_repeat
(TGACA)N#Simple_repeat	29	1	X.parietina glXanPari1	Simple_repeat
(TGCTG)N#Simple_repeat	29	1	X.parietina glXanPari1	Simple_repeat
(TGTACA)N#Simple_repeat	29	1	X.parietina glXanPari1	Simple_repeat
(TGTC)N#Simple_repeat	29	1	X.parietina glXanPari1	Simple_repeat

(TGTTTT)N#Simple_repeat	29	1	X.parietina glXanPari1	Simple_repeat
(TTTACC)N#Simple_repeat	29	1	X.parietina glXanPari1	Simple_repeat
(TTTGC)N#Simple_repeat	29	1	X.parietina glXanPari1	Simple_repeat
(ACCGT)N#Simple_repeat	28	1	X.parietina glXanPari1	Simple_repeat
(ACTTGAG)N#Simple_repeat	28	1	X.parietina glXanPari1	Simple_repeat
(AGCAA)N#Simple_repeat	28	1	X.parietina glXanPari1	Simple_repeat
(CAACT)N#Simple_repeat	28	1	X.parietina glXanPari1	Simple_repeat
(CAGA)N#Simple_repeat	28	1	X.parietina glXanPari1	Simple_repeat
(CCTCG)N#Simple_repeat	28	1	X.parietina glXanPari1	Simple_repeat
(CTAA)N#Simple_repeat	28	1	X.parietina glXanPari1	Simple_repeat
(CTTGAC)N#Simple_repeat	28	1	X.parietina glXanPari1	Simple_repeat
(GAGCAA)N#Simple_repeat	28	1	X.parietina glXanPari1	Simple_repeat
(GCTG)N#Simple_repeat	28	1	X.parietina glXanPari1	Simple_repeat
(GGACG)N#Simple_repeat	28	1	X.parietina glXanPari1	Simple_repeat
(GGAGCTT)N#Simple_repeat	28	1	X.parietina glXanPari1	Simple_repeat
(GGTTA)N#Simple_repeat	28	1	X.parietina glXanPari1	Simple_repeat
(TAGC)N#Simple_repeat	28	1	X.parietina glXanPari1	Simple_repeat
(TGATA)N#Simple_repeat	28	1	X.parietina glXanPari1	Simple_repeat
(TGTCT)N#Simple_repeat	28	1	X.parietina glXanPari1	Simple_repeat
(TTTA)N#Simple_repeat	28	1	X.parietina glXanPari1	Simple_repeat
(TTTCC)N#Simple_repeat	28	1	X.parietina glXanPari1	Simple_repeat
(ACCG)N#Simple_repeat	27	1	X.parietina glXanPari1	Simple_repeat
(AGAC)N#Simple_repeat	27	1	X.parietina glXanPari1	Simple_repeat
(AGCGG)N#Simple_repeat	27	1	X.parietina glXanPari1	Simple_repeat
(CAAGCC)N#Simple_repeat	27	1	X.parietina glXanPari1	Simple_repeat
(CAGCA)N#Simple_repeat	27	1	X.parietina glXanPari1	Simple_repeat

(CTACT)N#Simple_repeat	27	1	X.parietina glXanPari1	Simple_repeat
(CTTGG)N#Simple_repeat	27	1	X.parietina glXanPari1	Simple_repeat
(GACG)N#Simple_repeat	27	1	X.parietina glXanPari1	Simple_repeat
(GAGA)N#Simple_repeat	27	1	X.parietina glXanPari1	Simple_repeat
(GATCCC)N#Simple_repeat	27	1	X.parietina glXanPari1	Simple_repeat
(GATCGG)N#Simple_repeat	27	1	X.parietina glXanPari1	Simple_repeat
(GCTGGG)N#Simple_repeat	27	1	X.parietina glXanPari1	Simple_repeat
(GGTGCT)N#Simple_repeat	27	1	X.parietina glXanPari1	Simple_repeat
(TATGC)N#Simple_repeat	27	1	X.parietina glXanPari1	Simple_repeat
(TGAAT)N#Simple_repeat	27	1	X.parietina glXanPari1	Simple_repeat
(TGCCT)N#Simple_repeat	27	1	X.parietina glXanPari1	Simple_repeat
(TTGC)N#Simple_repeat	27	1	X.parietina glXanPari1	Simple_repeat
(TTGTA)N#Simple_repeat	27	1	X.parietina glXanPari1	Simple_repeat
(AGAGG)N#Simple_repeat	26	1	X.parietina glXanPari1	Simple_repeat
(AGGAG)N#Simple_repeat	26	1	X.parietina glXanPari1	Simple_repeat
(ATGTGG)N#Simple_repeat	26	1	X.parietina glXanPari1	Simple_repeat
(ATTCA)N#Simple_repeat	26	1	X.parietina glXanPari1	Simple_repeat
(CAAT)N#Simple_repeat	26	1	X.parietina glXanPari1	Simple_repeat
(CAGCAA)N#Simple_repeat	26	1	X.parietina glXanPari1	Simple_repeat
(CATG)N#Simple_repeat	26	1	X.parietina glXanPari1	Simple_repeat
(CCAAC)N#Simple_repeat	26	1	X.parietina glXanPari1	Simple_repeat
(GAATGC)N#Simple_repeat	26	1	X.parietina glXanPari1	Simple_repeat
(GGAAG)N#Simple_repeat	26	1	X.parietina glXanPari1	Simple_repeat
(GGAAT)N#Simple_repeat	26	1	X.parietina glXanPari1	Simple_repeat
(GGATGTT)N#Simple_repeat	26	1	X.parietina glXanPari1	Simple_repeat
(GGATT)N#Simple_repeat	26	1	X.parietina glXanPari1	Simple_repeat

(GGGTTG)N#Simple_repeat	26	1	X.parietina glXanPari1	Simple_repeat
(GGTGT)N#Simple_repeat	26	1	X.parietina glXanPari1	Simple_repeat
(TAAA)N#Simple_repeat	26	1	X.parietina glXanPari1	Simple_repeat
(TATCGC)N#Simple_repeat	26	1	X.parietina glXanPari1	Simple_repeat
(TCACT)N#Simple_repeat	26	1	X.parietina glXanPari1	Simple_repeat
(TGGCA)N#Simple_repeat	26	1	X.parietina glXanPari1	Simple_repeat
(TGTGA)N#Simple_repeat	26	1	X.parietina glXanPari1	Simple_repeat
(TTCG)N#Simple_repeat	26	1	X.parietina glXanPari1	Simple_repeat
(AAACCC)N#Simple_repeat	25	1	X.parietina glXanPari1	Simple_repeat
(AAAGC)N#Simple_repeat	25	1	X.parietina glXanPari1	Simple_repeat
(ACGAT)N#Simple_repeat	25	1	X.parietina glXanPari1	Simple_repeat
(AGTAG)N#Simple_repeat	25	1	X.parietina glXanPari1	Simple_repeat
(ATTAT)N#Simple_repeat	25	1	X.parietina glXanPari1	Simple_repeat
(CCACAC)N#Simple_repeat	25	1	X.parietina glXanPari1	Simple_repeat
(CGCAGT)N#Simple_repeat	25	1	X.parietina glXanPari1	Simple_repeat
(CGTCT)N#Simple_repeat	25	1	X.parietina glXanPari1	Simple_repeat
(CTCGA)N#Simple_repeat	25	1	X.parietina glXanPari1	Simple_repeat
(CTTA)N#Simple_repeat	25	1	X.parietina glXanPari1	Simple_repeat
(GAAATG)N#Simple_repeat	25	1	X.parietina glXanPari1	Simple_repeat
(GACAGC)N#Simple_repeat	25	1	X.parietina glXanPari1	Simple_repeat
(GCCAA)N#Simple_repeat	25	1	X.parietina glXanPari1	Simple_repeat
(GCCT)N#Simple_repeat	25	1	X.parietina glXanPari1	Simple_repeat
(GTTTTC)N#Simple_repeat	25	1	X.parietina glXanPari1	Simple_repeat
(TGGGGC)N#Simple_repeat	25	1	X.parietina glXanPari1	Simple_repeat
(TTCGC)N#Simple_repeat	25	1	X.parietina glXanPari1	Simple_repeat
(TTCGT)N#Simple_repeat	25	1	X.parietina glXanPari1	Simple_repeat

(ACACCG)N#Simple_repeat	24	1	X.parietina glXanPari1	Simple_repeat
(ACTCCC)N#Simple_repeat	24	1	X.parietina glXanPari1	Simple_repeat
(AGCA)N#Simple_repeat	24	1	X.parietina glXanPari1	Simple_repeat
(ATAACA)N#Simple_repeat	24	1	X.parietina glXanPari1	Simple_repeat
(ATGGG)N#Simple_repeat	24	1	X.parietina glXanPari1	Simple_repeat
(CACTCCT)N#Simple_repeat	24	1	X.parietina glXanPari1	Simple_repeat
(CCTG)N#Simple_repeat	24	1	X.parietina glXanPari1	Simple_repeat
(CGTCCT)N#Simple_repeat	24	1	X.parietina glXanPari1	Simple_repeat
(CTCG)N#Simple_repeat	24	1	X.parietina glXanPari1	Simple_repeat
(GAGCCT)N#Simple_repeat	24	1	X.parietina glXanPari1	Simple_repeat
(GGCTT)N#Simple_repeat	24	1	X.parietina glXanPari1	Simple_repeat
(GTTCCA)N#Simple_repeat	24	1	X.parietina glXanPari1	Simple_repeat
(TTCA)N#Simple_repeat	24	1	X.parietina glXanPari1	Simple_repeat
(AATGG)N#Simple_repeat	23	1	X.parietina glXanPari1	Simple_repeat
(AGAT)N#Simple_repeat	23	1	X.parietina glXanPari1	Simple_repeat
(AGTG)N#Simple_repeat	23	1	X.parietina glXanPari1	Simple_repeat
(ATGCT)N#Simple_repeat	23	1	X.parietina glXanPari1	Simple_repeat
(CGAT)N#Simple_repeat	23	1	X.parietina glXanPari1	Simple_repeat
(CTCAA)N#Simple_repeat	23	1	X.parietina glXanPari1	Simple_repeat
(GATC)N#Simple_repeat	23	1	X.parietina glXanPari1	Simple_repeat
(GTAG)N#Simple_repeat	23	1	X.parietina glXanPari1	Simple_repeat
(GTATA)N#Simple_repeat	23	1	X.parietina glXanPari1	Simple_repeat
(GTGGTGG)N#Simple_repeat	23	1	X.parietina glXanPari1	Simple_repeat
(TACG)N#Simple_repeat	23	1	X.parietina glXanPari1	Simple_repeat
(TGAAGA)N#Simple_repeat	23	1	X.parietina glXanPari1	Simple_repeat
(TGCG)N#Simple_repeat	23	1	X.parietina glXanPari1	Simple_repeat

(ATCA)N#Simple_repeat	22	1	X.parietina glXanPari1	Simple_repeat
(CAAA)N#Simple_repeat	22	1	X.parietina glXanPari1	Simple_repeat
(GTCA)N#Simple_repeat	22	1	X.parietina glXanPari1	Simple_repeat
(AGGC)N#Simple_repeat	21	1	X.parietina glXanPari1	Simple_repeat
(ATGT)N#Simple_repeat	21	1	X.parietina glXanPari1	Simple_repeat
(CCGG)N#Simple_repeat	21	1	X.parietina glXanPari1	Simple_repeat
(GGGT)N#Simple_repeat	21	1	X.parietina glXanPari1	Simple_repeat
(CTTCTT)N#Simple_repeat	20	1	X.parietina glXanPari1	Simple_repeat
(GCAC)N#Simple_repeat	20	1	X.parietina glXanPari1	Simple_repeat
(GTTT)N#Simple_repeat	15	1	X.parietina glXanPari1	Simple_repeat
(ATGAAG)N#Simple_repeat	12	1	X.parietina glXanPari1	Simple_repeat
(CCACCATCA)N#Simple_repeat	10	1	X.parietina glXanPari1	Simple_repeat
(CGCGGC)N#Simple_repeat	7	1	X.parietina glXanPari1	Simple_repeat
(AACCAC)N#Simple_repeat	6	1	X.parietina glXanPari1	Simple_repeat
(CAGCAC)N#Simple_repeat	6	1	X.parietina glXanPari1	Simple_repeat
(TGGATTG)N#Simple_repeat	6	1	X.parietina glXanPari1	Simple_repeat
(TTTTCCT)N#Simple_repeat	6	1	X.parietina glXanPari1	Simple_repeat
(CTTCTTT)N#Simple_repeat	5	1	X.parietina glXanPari1	Simple_repeat
(GGATTTC)N#Simple_repeat	5	1	X.parietina glXanPari1	Simple_repeat
(TCCTA)N#Simple_repeat	5	1	X.parietina glXanPari1	Simple_repeat
RND-1_FAMILY-1#LINE/Tad1	954637	370	X.parietina GTX0501	LINE/Tad1
RND-1_FAMILY-64#LINE/Tad1	258601	114	X.parietina GTX0501	LINE/Tad1
RND-1_FAMILY-28#LTR/Ty3	246963	171	X.parietina GTX0501	LTR/Ty3
RND-1_FAMILY-84#LINE/Tad1	192813	146	X.parietina GTX0501	LINE/Tad1
RND-1_FAMILY-81#LTR/Copia	184447	74	X.parietina GTX0501	LTR/Copia
RND-1_FAMILY-11#LTR/Copia	132351	38	X.parietina GTX0501	LTR/Copia

RND-3_FAMILY-34#LTR/Copia	131947	48	X.parietina GTX0501	LTR/Copia
RND-3_FAMILY-346#LTR/Ty3	125808	58	X.parietina GTX0501	LTR/Ty3
RND-1_FAMILY-47#LTR/Ty3	125070	65	X.parietina GTX0501	LTR/Ty3
RND-3_FAMILY-92#LTR/Ty3	99653	52	X.parietina GTX0501	LTR/Ty3
RND-1_FAMILY-36#Unknown	95919	67	X.parietina GTX0501	Unknown
RND-1_FAMILY-24#LTR/Ty3	89662	42	X.parietina GTX0501	LTR/Ty3
RND-3_FAMILY-343#LTR/Copia	85554	39	X.parietina GTX0501	LTR/Copia
RND-2_FAMILY-39#LTR/Ty3	83650	35	X.parietina GTX0501	LTR/Ty3
RND-2_FAMILY-315#LTR/Ty3	72408	28	X.parietina GTX0501	LTR/Ty3
RND-1_FAMILY-17#LTR/Ty3	68310	34	X.parietina GTX0501	LTR/Ty3
RND-1_FAMILY-51#LTR/Ty3	41976	12	X.parietina GTX0501	LTR/Ty3
RND-1_FAMILY-45#LTR/Copia	33679	13	X.parietina GTX0501	LTR/Copia
RND-1_FAMILY-54#LTR/Ty3	32700	24	X.parietina GTX0501	LTR/Ty3
RND-2_FAMILY-92#Unknown	31508	60	X.parietina GTX0501	Unknown
RND-1_FAMILY-75#DNA/Kolobok-H	31061	14	X.parietina GTX0501	DNA/Kolobok-H
RND-1_FAMILY-92#LTR/Ty3	29816	19	X.parietina GTX0501	LTR/Ty3
RND-1_FAMILY-79#Unknown	29718	20	X.parietina GTX0501	Unknown
RND-1_FAMILY-57#LTR/Copia	23372	10	X.parietina GTX0501	LTR/Copia
RND-1_FAMILY-27#Unknown	21704	117	X.parietina GTX0501	Unknown
RND-3_FAMILY-390#Unknown	17560	164	X.parietina GTX0501	Unknown
GA-RICH#Low_complexity	17428	320	X.parietina GTX0501	Low_complexity
RND-1_FAMILY-33#Unknown	17040	95	X.parietina GTX0501	Unknown
RND-1_FAMILY-86#LTR/Ty3	16932	17	X.parietina GTX0501	LTR/Ty3
RND-1_FAMILY-69#Unknown	15871	6	X.parietina GTX0501	Unknown
RND-1_FAMILY-34#Unknown	15863	20	X.parietina GTX0501	Unknown
RND-1_FAMILY-29#Unknown	14870	26	X.parietina GTX0501	Unknown

G-RICH#Low_complexity	14476	260	X.parietina GTX0501	Low_complexity
RND-1_FAMILY-49#DNA/MULE-MuDR	14014	12	X.parietina GTX0501	DNA/MULE-MuDR
RND-1_FAMILY-71#Unknown	13878	10	X.parietina GTX0501	Unknown
RND-1_FAMILY-39#DNA/MULE-MuDR	13499	10	X.parietina GTX0501	DNA/MULE-MuDR
RND-1_FAMILY-6#Unknown	11641	26	X.parietina GTX0501	Unknown
RND-1_FAMILY-70#LTR/Ty3	11163	11	X.parietina GTX0501	LTR/Ty3
RND-1_FAMILY-89#Unknown	10454	19	X.parietina GTX0501	Unknown
RND-2_FAMILY-65#Unknown	10375	74	X.parietina GTX0501	Unknown
RND-1_FAMILY-48#DNA/Kolobok-H	10151	8	X.parietina GTX0501	DNA/Kolobok-H
RND-1_FAMILY-40#Unknown	9634	23	X.parietina GTX0501	Unknown
RND-3_FAMILY-62#Unknown	9554	29	X.parietina GTX0501	Unknown
RND-1_FAMILY-38#LTR/Ty3	9306	7	X.parietina GTX0501	LTR/Ty3
RND-3_FAMILY-448#Unknown	9195	94	X.parietina GTX0501	Unknown
RND-3_FAMILY-559#Unknown	7755	58	X.parietina GTX0501	Unknown
RND-1_FAMILY-44#Unknown	7744	17	X.parietina GTX0501	Unknown
RND-1_FAMILY-25#Unknown	7667	17	X.parietina GTX0501	Unknown
RND-1_FAMILY-20#Unknown	7196	20	X.parietina GTX0501	Unknown
RND-1_FAMILY-50#Unknown	6889	30	X.parietina GTX0501	Unknown
RND-1_FAMILY-3#Unknown	6235	2	X.parietina GTX0501	Unknown
RND-1_FAMILY-52#Unknown	6121	20	X.parietina GTX0501	Unknown
RND-1_FAMILY-76#LTR/Ty3	5903	9	X.parietina GTX0501	LTR/Ty3
RND-1_FAMILY-87#Unknown	5772	19	X.parietina GTX0501	Unknown
RND-1_FAMILY-97#Unknown	5627	18	X.parietina GTX0501	Unknown
RND-1_FAMILY-41#Unknown	5362	42	X.parietina GTX0501	Unknown
RND-1_FAMILY-56#DNA/MULE-MuDR	5134	28	X.parietina GTX0501	DNA/MULE-MuDR

RND-1_FAMILY-46#Unknown	4862	69	X.parietina GTX0501	Unknown
(CCT)N#Simple_repeat	4816	108	X.parietina GTX0501	Simple_repeat
RND-3_FAMILY-258#Unknown	4344	64	X.parietina GTX0501	Unknown
A-RICH#Low_complexity	4316	94	X.parietina GTX0501	Low_complexity
RND-1_FAMILY-16#Unknown	4042	12	X.parietina GTX0501	Unknown
RND-1_FAMILY-42#Unknown	3991	23	X.parietina GTX0501	Unknown
RND-1_FAMILY-90#Unknown	3985	49	X.parietina GTX0501	Unknown
RND-1_FAMILY-73#LINE/Tad1	3950	9	X.parietina GTX0501	LINE/Tad1
RND-1_FAMILY-19#Unknown	3753	25	X.parietina GTX0501	Unknown
RND-1_FAMILY-23#Unknown	3644	25	X.parietina GTX0501	Unknown
RND-1_FAMILY-93#Unknown	3587	19	X.parietina GTX0501	Unknown
RND-1_FAMILY-43#Unknown	3511	6	X.parietina GTX0501	Unknown
RND-1_FAMILY-62#Unknown	3420	17	X.parietina GTX0501	Unknown
RND-1_FAMILY-80#Unknown	3383	15	X.parietina GTX0501	Unknown
RND-1_FAMILY-99#Unknown	3354	41	X.parietina GTX0501	Unknown
RND-1_FAMILY-88#Unknown	3303	17	X.parietina GTX0501	Unknown
RND-1_FAMILY-4#Unknown	3298	7	X.parietina GTX0501	Unknown
(CTC)N#Simple_repeat	3203	52	X.parietina GTX0501	Simple_repeat
RND-1_FAMILY-83#Unknown	3053	38	X.parietina GTX0501	Unknown
RND-1_FAMILY-82#Unknown	2990	11	X.parietina GTX0501	Unknown
RND-1_FAMILY-66#LINE/Tad1	2936	8	X.parietina GTX0501	LINE/Tad1
RND-3_FAMILY-135#Unknown	2900	21	X.parietina GTX0501	Unknown
RND-1_FAMILY-85#Unknown	2840	7	X.parietina GTX0501	Unknown
(C)N#Simple_repeat	2785	110	X.parietina GTX0501	Simple_repeat
RND-1_FAMILY-22#Unknown	2669	8	X.parietina GTX0501	Unknown
RND-1_FAMILY-58#Unknown	2658	19	X.parietina GTX0501	Unknown

RND-1_FAMILY-65#Unknown	2573	34	X.parietina GTX0501	Unknown
RND-1_FAMILY-60#Unknown	2456	30	X.parietina GTX0501	Unknown
(TCC)N#Simple_repeat	2450	48	X.parietina GTX0501	Simple_repeat
RND-1_FAMILY-37#Unknown	2444	10	X.parietina GTX0501	Unknown
(G)N#Simple_repeat	2162	96	X.parietina GTX0501	Simple_repeat
RND-1_FAMILY-74#Unknown	2136	32	X.parietina GTX0501	Unknown
RND-1_FAMILY-61#Unknown	2127	27	X.parietina GTX0501	Unknown
(CCA)N#Simple_repeat	2084	52	X.parietina GTX0501	Simple_repeat
RND-1_FAMILY-96#Unknown	2058	28	X.parietina GTX0501	Unknown
(TTC)N#Simple_repeat	1997	44	X.parietina GTX0501	Simple_repeat
RND-1_FAMILY-7#Unknown	1989	15	X.parietina GTX0501	Unknown
RND-1_FAMILY-63#Unknown	1802	17	X.parietina GTX0501	Unknown
RND-1_FAMILY-53#Unknown	1653	11	X.parietina GTX0501	Unknown
RND-1_FAMILY-5#LINE/Tad1	1578	3	X.parietina GTX0501	LINE/Tad1
(GGT)N#Simple_repeat	1574	39	X.parietina GTX0501	Simple_repeat
RND-1_FAMILY-91#Unknown	1508	20	X.parietina GTX0501	Unknown
RND-1_FAMILY-72#Unknown	1464	6	X.parietina GTX0501	Unknown
RND-1_FAMILY-8#Unknown	1427	20	X.parietina GTX0501	Unknown
(CAA)N#Simple_repeat	1402	43	X.parietina GTX0501	Simple_repeat
RND-1_FAMILY-67#Unknown	1395	16	X.parietina GTX0501	Unknown
(CAC)N#Simple_repeat	1373	33	X.parietina GTX0501	Simple_repeat
(TGA)N#Simple_repeat	1365	38	X.parietina GTX0501	Simple_repeat
(CTT)N#Simple_repeat	1358	39	X.parietina GTX0501	Simple_repeat
(ACC)N#Simple_repeat	1292	23	X.parietina GTX0501	Simple_repeat
(GAT)N#Simple_repeat	1259	33	X.parietina GTX0501	Simple_repeat
(CAT)N#Simple_repeat	1243	36	X.parietina GTX0501	Simple_repeat

(TGG)N#Simple_repeat	1197	27	X.parietina GTX0501	Simple_repeat
RND-1_FAMILY-32#Unknown	1197	22	X.parietina GTX0501	Unknown
(GTT)N#Simple_repeat	1142	39	X.parietina GTX0501	Simple_repeat
(TGC)N#Simple_repeat	1117	30	X.parietina GTX0501	Simple_repeat
(TCT)N#Simple_repeat	1097	34	X.parietina GTX0501	Simple_repeat
(GCA)N#Simple_repeat	1079	30	X.parietina GTX0501	Simple_repeat
RND-1_FAMILY-59#Unknown	1063	14	X.parietina GTX0501	Unknown
(TGT)N#Simple_repeat	1053	31	X.parietina GTX0501	Simple_repeat
(CAG)N#Simple_repeat	1031	29	X.parietina GTX0501	Simple_repeat
RND-1_FAMILY-9#Unknown	967	5	X.parietina GTX0501	Unknown
(TCA)N#Simple_repeat	939	25	X.parietina GTX0501	Simple_repeat
(TC)N#Simple_repeat	920	29	X.parietina GTX0501	Simple_repeat
RND-1_FAMILY-31#Unknown	903	15	X.parietina GTX0501	Unknown
RND-1_FAMILY-77#Unknown	862	7	X.parietina GTX0501	Unknown
(T)N#Simple_repeat	821	32	X.parietina GTX0501	Simple_repeat
(A)N#Simple_repeat	801	28	X.parietina GTX0501	Simple_repeat
(ACA)N#Simple_repeat	795	21	X.parietina GTX0501	Simple_repeat
(GAA)N#Simple_repeat	788	29	X.parietina GTX0501	Simple_repeat
RND-1_FAMILY-55#Unknown	779	7	X.parietina GTX0501	Unknown
(CGG)N#Simple_repeat	733	22	X.parietina GTX0501	Simple_repeat
(CCCT)N#Simple_repeat	730	15	X.parietina GTX0501	Simple_repeat
(GTG)N#Simple_repeat	729	17	X.parietina GTX0501	Simple_repeat
(AAG)N#Simple_repeat	719	25	X.parietina GTX0501	Simple_repeat
RND-1_FAMILY-14#Unknown	716	5	X.parietina GTX0501	Unknown
(ACAGAG)N#Simple_repeat	703	3	X.parietina GTX0501	Simple_repeat
RND-1_FAMILY-12#Unknown	701	5	X.parietina GTX0501	Unknown

(CT)N#Simple_repeat	694	21	X.parietina GTX0501	Simple_repeat
(GGC)N#Simple_repeat	692	20	X.parietina GTX0501	Simple_repeat
(TCG)N#Simple_repeat	676	19	X.parietina GTX0501	Simple_repeat
(TCCCT)N#Simple_repeat	664	8	X.parietina GTX0501	Simple_repeat
(CGA)N#Simple_repeat	647	17	X.parietina GTX0501	Simple_repeat
(GA)N#Simple_repeat	642	24	X.parietina GTX0501	Simple_repeat
RND-1_FAMILY-10#Unknown	608	5	X.parietina GTX0501	Unknown
RND-1_FAMILY-21#Simple_repeat	607	5	X.parietina GTX0501	Simple_repeat
(GGA)N#Simple_repeat	604	23	X.parietina GTX0501	Simple_repeat
(TCCC)N#Simple_repeat	600	12	X.parietina GTX0501	Simple_repeat
(TTTC)N#Simple_repeat	598	14	X.parietina GTX0501	Simple_repeat
(CA)N#Simple_repeat	597	17	X.parietina GTX0501	Simple_repeat
(CCTCCT)N#Simple_repeat	594	6	X.parietina GTX0501	Simple_repeat
RND-1_FAMILY-15#Unknown	584	5	X.parietina GTX0501	Unknown
(AGA)N#Simple_repeat	574	21	X.parietina GTX0501	Simple_repeat
(CCTC)N#Simple_repeat	567	14	X.parietina GTX0501	Simple_repeat
(CTG)N#Simple_repeat	551	17	X.parietina GTX0501	Simple_repeat
RND-1_FAMILY-13#Unknown	547	4	X.parietina GTX0501	Unknown
(TCCCCT)N#Simple_repeat	544	6	X.parietina GTX0501	Simple_repeat
(TCCCA)N#Simple_repeat	541	10	X.parietina GTX0501	Simple_repeat
RND-1_FAMILY-35#Unknown	532	6	X.parietina GTX0501	Unknown
(GAC)N#Simple_repeat	519	15	X.parietina GTX0501	Simple_repeat
(GCT)N#Simple_repeat	515	18	X.parietina GTX0501	Simple_repeat
(TA)N#Simple_repeat	508	16	X.parietina GTX0501	Simple_repeat
(ATC)N#Simple_repeat	507	14	X.parietina GTX0501	Simple_repeat
(GTC)N#Simple_repeat	505	13	X.parietina GTX0501	Simple_repeat

(GGTGGA)N#Simple_repeat	499	8	X.parietina GTX0501	Simple_repeat
(TCCCCA)N#Simple_repeat	467	5	X.parietina GTX0501	Simple_repeat
(AAGATA)N#Simple_repeat	463	1	X.parietina GTX0501	Simple_repeat
(CCACCT)N#Simple_repeat	455	9	X.parietina GTX0501	Simple_repeat
(GCC)N#Simple_repeat	451	14	X.parietina GTX0501	Simple_repeat
(CATC)N#Simple_repeat	449	10	X.parietina GTX0501	Simple_repeat
(TG)N#Simple_repeat	448	17	X.parietina GTX0501	Simple_repeat
(CCAT)N#Simple_repeat	443	9	X.parietina GTX0501	Simple_repeat
(AGC)N#Simple_repeat	442	13	X.parietina GTX0501	Simple_repeat
(TCCTCT)N#Simple_repeat	439	8	X.parietina GTX0501	Simple_repeat
(TCTTTA)N#Simple_repeat	434	1	X.parietina GTX0501	Simple_repeat
(AC)N#Simple_repeat	430	13	X.parietina GTX0501	Simple_repeat
(AAT)N#Simple_repeat	408	14	X.parietina GTX0501	Simple_repeat
RND-1_FAMILY-94#Unknown	404	6	X.parietina GTX0501	Unknown
(GAG)N#Simple_repeat	399	14	X.parietina GTX0501	Simple_repeat
(CCTCCC)N#Simple_repeat	394	6	X.parietina GTX0501	Simple_repeat
(TCCTTC)N#Simple_repeat	389	5	X.parietina GTX0501	Simple_repeat
(GAGGTG)N#Simple_repeat	377	6	X.parietina GTX0501	Simple_repeat
(CCTCAT)N#Simple_repeat	372	8	X.parietina GTX0501	Simple_repeat
(CCG)N#Simple_repeat	371	11	X.parietina GTX0501	Simple_repeat
(GCG)N#Simple_repeat	371	11	X.parietina GTX0501	Simple_repeat
(GACGAT)N#Simple_repeat	368	7	X.parietina GTX0501	Simple_repeat
(AAC)N#Simple_repeat	366	12	X.parietina GTX0501	Simple_repeat
(ATG)N#Simple_repeat	364	10	X.parietina GTX0501	Simple_repeat
(CCTCC)N#Simple_repeat	363	7	X.parietina GTX0501	Simple_repeat
(CTCCTT)N#Simple_repeat	359	5	X.parietina GTX0501	Simple_repeat

(AG)N#Simple_repeat	355	14	X.parietina GTX0501	Simple_repeat
(ACG)N#Simple_repeat	353	10	X.parietina GTX0501	Simple_repeat
(CCCTTC)N#Simple_repeat	351	2	X.parietina GTX0501	Simple_repeat
(CTTC)N#Simple_repeat	347	10	X.parietina GTX0501	Simple_repeat
(CCCCT)N#Simple_repeat	345	5	X.parietina GTX0501	Simple_repeat
(CGC)N#Simple_repeat	340	8	X.parietina GTX0501	Simple_repeat
RND-1_FAMILY-18#Unknown	339	6	X.parietina GTX0501	Unknown
RND-1_FAMILY-98#Unknown	335	3	X.parietina GTX0501	Unknown
(AT)N#Simple_repeat	333	10	X.parietina GTX0501	Simple_repeat
(CATCCT)N#Simple_repeat	332	5	X.parietina GTX0501	Simple_repeat
(CGT)N#Simple_repeat	329	10	X.parietina GTX0501	Simple_repeat
(TTAGGG)N#Simple_repeat	326	3	X.parietina GTX0501	Simple_repeat
(GACGAG)N#Simple_repeat	320	3	X.parietina GTX0501	Simple_repeat
(TCTTCC)N#Simple_repeat	314	8	X.parietina GTX0501	Simple_repeat
(TCCA)N#Simple_repeat	310	7	X.parietina GTX0501	Simple_repeat
(TTG)N#Simple_repeat	306	11	X.parietina GTX0501	Simple_repeat
(CAGCAA)N#Simple_repeat	304	5	X.parietina GTX0501	Simple_repeat
(CCCCCT)N#Simple_repeat	303	5	X.parietina GTX0501	Simple_repeat
(CCTCCA)N#Simple_repeat	303	5	X.parietina GTX0501	Simple_repeat
(TTCTCC)N#Simple_repeat	301	6	X.parietina GTX0501	Simple_repeat
(CACC)N#Simple_repeat	299	4	X.parietina GTX0501	Simple_repeat
(CCCCCA)N#Simple_repeat	297	5	X.parietina GTX0501	Simple_repeat
(CAGCATCA)N#Simple_repeat	294	1	X.parietina GTX0501	Simple_repeat
(GT)N#Simple_repeat	291	12	X.parietina GTX0501	Simple_repeat
(TGGA)N#Simple_repeat	288	8	X.parietina GTX0501	Simple_repeat
(CTCCTC)N#Simple_repeat	284	4	X.parietina GTX0501	Simple_repeat

(CCATCCC)N#Simple_repeat	281	3	X.parietina GTX0501	Simple_repeat
(GGAGGT)N#Simple_repeat	280	4	X.parietina GTX0501	Simple_repeat
(CCCTT)N#Simple_repeat	277	5	X.parietina GTX0501	Simple_repeat
(CCCTCC)N#Simple_repeat	276	5	X.parietina GTX0501	Simple_repeat
(CCTCG)N#Simple_repeat	273	8	X.parietina GTX0501	Simple_repeat
(CTCCC)N#Simple_repeat	273	8	X.parietina GTX0501	Simple_repeat
(CTCCTCTTC)N#Simple_repeat	266	1	X.parietina GTX0501	Simple_repeat
(TCTCC)N#Simple_repeat	265	6	X.parietina GTX0501	Simple_repeat
(GTAAG)N#Simple_repeat	264	8	X.parietina GTX0501	Simple_repeat
(CATCCC)N#Simple_repeat	260	2	X.parietina GTX0501	Simple_repeat
(CGAGAG)N#Simple_repeat	255	1	X.parietina GTX0501	Simple_repeat
(TGTTGC)N#Simple_repeat	254	3	X.parietina GTX0501	Simple_repeat
(TTTTC)N#Simple_repeat	242	4	X.parietina GTX0501	Simple_repeat
(GAGGTCGAG)N#Simple_repeat	241	5	X.parietina GTX0501	Simple_repeat
(TCACTC)N#Simple_repeat	241	3	X.parietina GTX0501	Simple_repeat
(CACCAT)N#Simple_repeat	239	5	X.parietina GTX0501	Simple_repeat
(CTTCCT)N#Simple_repeat	238	4	X.parietina GTX0501	Simple_repeat
(CCTCCCC)N#Simple_repeat	237	2	X.parietina GTX0501	Simple_repeat
(TCCCCC)N#Simple_repeat	235	5	X.parietina GTX0501	Simple_repeat
(AGGT)N#Simple_repeat	233	6	X.parietina GTX0501	Simple_repeat
(GGTGGT)N#Simple_repeat	232	5	X.parietina GTX0501	Simple_repeat
(TCCCC)N#Simple_repeat	232	5	X.parietina GTX0501	Simple_repeat
(CTTTT)N#Simple_repeat	230	6	X.parietina GTX0501	Simple_repeat
(CCCCTT)N#Simple_repeat	229	5	X.parietina GTX0501	Simple_repeat
(CATCTC)N#Simple_repeat	227	3	X.parietina GTX0501	Simple_repeat
(CCTT)N#Simple_repeat	225	6	X.parietina GTX0501	Simple_repeat

(ACCC)N#Simple_repeat	224	4	X.parietina GTX0501	Simple_repeat
(CCCC)N#Simple_repeat	223	4	X.parietina GTX0501	Simple_repeat
(CTCCCC)N#Simple_repeat	223	2	X.parietina GTX0501	Simple_repeat
(GGTAA)N#Simple_repeat	222	6	X.parietina GTX0501	Simple_repeat
(CCACCG)N#Simple_repeat	219	4	X.parietina GTX0501	Simple_repeat
(CCCTCT)N#Simple_repeat	219	5	X.parietina GTX0501	Simple_repeat
(AATC)N#Simple_repeat	218	7	X.parietina GTX0501	Simple_repeat
(GGAT)N#Simple_repeat	218	6	X.parietina GTX0501	Simple_repeat
(GTGGAG)N#Simple_repeat	216	4	X.parietina GTX0501	Simple_repeat
(TGTTGG)N#Simple_repeat	215	4	X.parietina GTX0501	Simple_repeat
(ACAC)N#Simple_repeat	214	5	X.parietina GTX0501	Simple_repeat
(CCAC)N#Simple_repeat	213	4	X.parietina GTX0501	Simple_repeat
(GCCTCC)N#Simple_repeat	213	3	X.parietina GTX0501	Simple_repeat
(CCCCAA)N#Simple_repeat	208	4	X.parietina GTX0501	Simple_repeat
(CTCCCT)N#Simple_repeat	208	4	X.parietina GTX0501	Simple_repeat
(CCCCCAA)N#Simple_repeat	203	4	X.parietina GTX0501	Simple_repeat
(CTCCGC)N#Simple_repeat	202	2	X.parietina GTX0501	Simple_repeat
(TGGCGG)N#Simple_repeat	201	5	X.parietina GTX0501	Simple_repeat
(ATGG)N#Simple_repeat	198	5	X.parietina GTX0501	Simple_repeat
(AAGGT)N#Simple_repeat	197	5	X.parietina GTX0501	Simple_repeat
(CTCC)N#Simple_repeat	196	6	X.parietina GTX0501	Simple_repeat
(GAAGAC)N#Simple_repeat	196	3	X.parietina GTX0501	Simple_repeat
(CCACCC)N#Simple_repeat	192	2	X.parietina GTX0501	Simple_repeat
(GGTA)N#Simple_repeat	191	5	X.parietina GTX0501	Simple_repeat
(CCAACC)N#Simple_repeat	190	3	X.parietina GTX0501	Simple_repeat
(GGGAAAAG)N#Simple_repeat	188	1	X.parietina GTX0501	Simple_repeat

(TCCACCA)N#Simple_repeat	188	4	X.parietina GTX0501	Simple_repeat
(CCCCTC)N#Simple_repeat	187	4	X.parietina GTX0501	Simple_repeat
(CCTCAG)N#Simple_repeat	187	3	X.parietina GTX0501	Simple_repeat
(TGGAT)N#Simple_repeat	187	2	X.parietina GTX0501	Simple_repeat
(CCAACA)N#Simple_repeat	186	5	X.parietina GTX0501	Simple_repeat
(CCCCA)N#Simple_repeat	186	4	X.parietina GTX0501	Simple_repeat
(CGCCT)N#Simple_repeat	186	6	X.parietina GTX0501	Simple_repeat
(ATCC)N#Simple_repeat	185	6	X.parietina GTX0501	Simple_repeat
(CTCTC)N#Simple_repeat	185	4	X.parietina GTX0501	Simple_repeat
(CTTT)N#Simple_repeat	184	6	X.parietina GTX0501	Simple_repeat
(CACCAA)N#Simple_repeat	183	4	X.parietina GTX0501	Simple_repeat
(CCTTTC)N#Simple_repeat	181	4	X.parietina GTX0501	Simple_repeat
(CCATCC)N#Simple_repeat	177	4	X.parietina GTX0501	Simple_repeat
(ACCCTCC)N#Simple_repeat	175	3	X.parietina GTX0501	Simple_repeat
(CTCGTC)N#Simple_repeat	175	4	X.parietina GTX0501	Simple_repeat
(GAGATG)N#Simple_repeat	174	4	X.parietina GTX0501	Simple_repeat
(GGAGGC)N#Simple_repeat	174	3	X.parietina GTX0501	Simple_repeat
(CCTCGACCT)N#Simple_repeat	172	4	X.parietina GTX0501	Simple_repeat
(GGTGGC)N#Simple_repeat	172	4	X.parietina GTX0501	Simple_repeat
(TCCTCC)N#Simple_repeat	172	3	X.parietina GTX0501	Simple_repeat
(TCTGGC)N#Simple_repeat	172	5	X.parietina GTX0501	Simple_repeat
(TGGTGT)N#Simple_repeat	172	3	X.parietina GTX0501	Simple_repeat
(TCTTCA)N#Simple_repeat	171	3	X.parietina GTX0501	Simple_repeat
(TGGTG)N#Simple_repeat	171	4	X.parietina GTX0501	Simple_repeat
(TACCT)N#Simple_repeat	170	5	X.parietina GTX0501	Simple_repeat
(TCCTCG)N#Simple_repeat	169	4	X.parietina GTX0501	Simple_repeat

(AACCCC)N#Simple_repeat	168	3	X.parietina GTX0501	Simple_repeat
(CCCTCCTT)N#Simple_repeat	167	1	X.parietina GTX0501	Simple_repeat
(ACCAAC)N#Simple_repeat	166	3	X.parietina GTX0501	Simple_repeat
(AGG)N#Simple_repeat	165	6	X.parietina GTX0501	Simple_repeat
(CCCAC)N#Simple_repeat	165	3	X.parietina GTX0501	Simple_repeat
(TGAT)N#Simple_repeat	165	5	X.parietina GTX0501	Simple_repeat
(CCCCAT)N#Simple_repeat	163	4	X.parietina GTX0501	Simple_repeat
(GGATGA)N#Simple_repeat	163	3	X.parietina GTX0501	Simple_repeat
(CCCTCG)N#Simple_repeat	162	2	X.parietina GTX0501	Simple_repeat
(CCTCAC)N#Simple_repeat	162	3	X.parietina GTX0501	Simple_repeat
(CTGCTC)N#Simple_repeat	161	3	X.parietina GTX0501	Simple_repeat
(AGT)N#Simple_repeat	160	5	X.parietina GTX0501	Simple_repeat
(TCTCCC)N#Simple_repeat	159	5	X.parietina GTX0501	Simple_repeat
(ATCTC)N#Simple_repeat	158	4	X.parietina GTX0501	Simple_repeat
(GATGAC)N#Simple_repeat	158	3	X.parietina GTX0501	Simple_repeat
(GTCATC)N#Simple_repeat	158	3	X.parietina GTX0501	Simple_repeat
(TCTATTATC)N#Simple_repeat	157	1	X.parietina GTX0501	Simple_repeat
(TCTTCG)N#Simple_repeat	157	3	X.parietina GTX0501	Simple_repeat
(GACGAA)N#Simple_repeat	155	2	X.parietina GTX0501	Simple_repeat
(TCTCCA)N#Simple_repeat	155	2	X.parietina GTX0501	Simple_repeat
(AGACCG)N#Simple_repeat	154	1	X.parietina GTX0501	Simple_repeat
(GTAT)N#Simple_repeat	153	5	X.parietina GTX0501	Simple_repeat
(AGGGGAA)N#Simple_repeat	150	1	X.parietina GTX0501	Simple_repeat
(TACC)N#Simple_repeat	150	5	X.parietina GTX0501	Simple_repeat
(GATGAG)N#Simple_repeat	149	3	X.parietina GTX0501	Simple_repeat
(TCATCG)N#Simple_repeat	148	3	X.parietina GTX0501	Simple_repeat

(CCTTCC)N#Simple_repeat	147	2	X.parietina GTX0501	Simple_repeat
(TCCACC)N#Simple_repeat	147	3	X.parietina GTX0501	Simple_repeat
(ATCCC)N#Simple_repeat	146	3	X.parietina GTX0501	Simple_repeat
(TTCGTC)N#Simple_repeat	146	3	X.parietina GTX0501	Simple_repeat
(ACAGCA)N#Simple_repeat	145	2	X.parietina GTX0501	Simple_repeat
(GCAA)N#Simple_repeat	143	3	X.parietina GTX0501	Simple_repeat
(TGGTGA)N#Simple_repeat	143	2	X.parietina GTX0501	Simple_repeat
(CC)N#Simple_repeat	142	3	X.parietina GTX0501	Simple_repeat
(GAAA)N#Simple_repeat	142	5	X.parietina GTX0501	Simple_repeat
(GATG)N#Simple_repeat	142	4	X.parietina GTX0501	Simple_repeat
(CTTCGT)N#Simple_repeat	141	2	X.parietina GTX0501	Simple_repeat
(GTA)N#Simple_repeat	141	5	X.parietina GTX0501	Simple_repeat
(TGTCAT)N#Simple_repeat	141	1	X.parietina GTX0501	Simple_repeat
(CCTTCT)N#Simple_repeat	140	2	X.parietina GTX0501	Simple_repeat
(ACGAAG)N#Simple_repeat	138	2	X.parietina GTX0501	Simple_repeat
(CCCACCC)N#Simple_repeat	138	2	X.parietina GTX0501	Simple_repeat
(CTCTCC)N#Simple_repeat	138	2	X.parietina GTX0501	Simple_repeat
(ACTCC)N#Simple_repeat	137	1	X.parietina GTX0501	Simple_repeat
(TCCACA)N#Simple_repeat	137	3	X.parietina GTX0501	Simple_repeat
(AAGG)N#Simple_repeat	136	2	X.parietina GTX0501	Simple_repeat
(ACCTCA)N#Simple_repeat	136	1	X.parietina GTX0501	Simple_repeat
(GATGGC)N#Simple_repeat	136	3	X.parietina GTX0501	Simple_repeat
(GCGGTG)N#Simple_repeat	136	3	X.parietina GTX0501	Simple_repeat
(CAAAG)N#Simple_repeat	135	1	X.parietina GTX0501	Simple_repeat
(GTCGTC)N#Simple_repeat	135	3	X.parietina GTX0501	Simple_repeat
(CTCTCCA)N#Simple_repeat	134	2	X.parietina GTX0501	Simple_repeat

(CCTTCG)N#Simple_repeat	133	2	X.parietina GTX0501	Simple_repeat
(TTGA)N#Simple_repeat	133	2	X.parietina GTX0501	Simple_repeat
(AAGCAC)N#Simple_repeat	132	1	X.parietina GTX0501	Simple_repeat
(TAC)N#Simple_repeat	132	3	X.parietina GTX0501	Simple_repeat
(AGGTA)N#Simple_repeat	131	4	X.parietina GTX0501	Simple_repeat
(GAGGCG)N#Simple_repeat	131	3	X.parietina GTX0501	Simple_repeat
(GCAGGA)N#Simple_repeat	131	2	X.parietina GTX0501	Simple_repeat
(ACACC)N#Simple_repeat	130	3	X.parietina GTX0501	Simple_repeat
(CCACAA)N#Simple_repeat	130	2	X.parietina GTX0501	Simple_repeat
(ACGATG)N#Simple_repeat	128	2	X.parietina GTX0501	Simple_repeat
(AGGAAGG)N#Simple_repeat	128	1	X.parietina GTX0501	Simple_repeat
(GGGGGAA)N#Simple_repeat	128	1	X.parietina GTX0501	Simple_repeat
(AACCCT)N#Simple_repeat	127	1	X.parietina GTX0501	Simple_repeat
(AAGA)N#Simple_repeat	127	4	X.parietina GTX0501	Simple_repeat
(GGAG)N#Simple_repeat	127	5	X.parietina GTX0501	Simple_repeat
(TCGTCT)N#Simple_repeat	127	2	X.parietina GTX0501	Simple_repeat
(TCTT)N#Simple_repeat	127	4	X.parietina GTX0501	Simple_repeat
(TCCGCC)N#Simple_repeat	126	1	X.parietina GTX0501	Simple_repeat
(ACCA)N#Simple_repeat	125	3	X.parietina GTX0501	Simple_repeat
(TGGTGG)N#Simple_repeat	125	3	X.parietina GTX0501	Simple_repeat
(TTGAGG)N#Simple_repeat	125	2	X.parietina GTX0501	Simple_repeat
(ATCTCC)N#Simple_repeat	123	3	X.parietina GTX0501	Simple_repeat
(CCAA)N#Simple_repeat	123	4	X.parietina GTX0501	Simple_repeat
(GGAGTG)N#Simple_repeat	123	3	X.parietina GTX0501	Simple_repeat
(TAAGG)N#Simple_repeat	123	4	X.parietina GTX0501	Simple_repeat
(TTCCCA)N#Simple_repeat	123	2	X.parietina GTX0501	Simple_repeat

(GGCGGT)N#Simple_repeat	122	2	X.parietina GTX0501	Simple_repeat
(TGGAGC)N#Simple_repeat	121	3	X.parietina GTX0501	Simple_repeat
(CCCCCAT)N#Simple_repeat	118	2	X.parietina GTX0501	Simple_repeat
(GAGAGT)N#Simple_repeat	118	2	X.parietina GTX0501	Simple_repeat
(GTTGTC)N#Simple_repeat	118	2	X.parietina GTX0501	Simple_repeat
(TGGGA)N#Simple_repeat	118	4	X.parietina GTX0501	Simple_repeat
(CCCAACC)N#Simple_repeat	117	1	X.parietina GTX0501	Simple_repeat
(CCTTC)N#Simple_repeat	117	3	X.parietina GTX0501	Simple_repeat
(CGAGGA)N#Simple_repeat	117	3	X.parietina GTX0501	Simple_repeat
(CAGC)N#Simple_repeat	114	4	X.parietina GTX0501	Simple_repeat
(GGCGAG)N#Simple_repeat	114	2	X.parietina GTX0501	Simple_repeat
(ATGT)N#Simple_repeat	113	2	X.parietina GTX0501	Simple_repeat
(CAGCAGCAA)N#Simple_repeat	113	2	X.parietina GTX0501	Simple_repeat
(GGGTTA)N#Simple_repeat	113	1	X.parietina GTX0501	Simple_repeat
(TCCCCCT)N#Simple_repeat	113	2	X.parietina GTX0501	Simple_repeat
(TGAGGA)N#Simple_repeat	113	2	X.parietina GTX0501	Simple_repeat
(CCCAT)N#Simple_repeat	112	4	X.parietina GTX0501	Simple_repeat
(CTCACAT)N#Simple_repeat	112	1	X.parietina GTX0501	Simple_repeat
(GAGGAT)N#Simple_repeat	112	2	X.parietina GTX0501	Simple_repeat
(CCCCTTT)N#Simple_repeat	110	2	X.parietina GTX0501	Simple_repeat
(CTTCCC)N#Simple_repeat	110	3	X.parietina GTX0501	Simple_repeat
(GACCCATT)N#Simple_repeat	110	1	X.parietina GTX0501	Simple_repeat
(TCTTC)N#Simple_repeat	110	2	X.parietina GTX0501	Simple_repeat
(TGGTCGTTCG)N#Simple_repeat	110	1	X.parietina GTX0501	Simple_repeat
(AGATGA)N#Simple_repeat	109	3	X.parietina GTX0501	Simple_repeat
(CATCA)N#Simple_repeat	108	2	X.parietina GTX0501	Simple_repeat

(GAAGAGC)N#Simple_repeat	108	2	X.parietina GTX0501	Simple_repeat
(GCAACC)N#Simple_repeat	108	1	X.parietina GTX0501	Simple_repeat
(TTCC)N#Simple_repeat	108	4	X.parietina GTX0501	Simple_repeat
(TACTA)N#Simple_repeat	107	2	X.parietina GTX0501	Simple_repeat
(CTTTCC)N#Simple_repeat	104	2	X.parietina GTX0501	Simple_repeat
(CCTTTTC)N#Simple_repeat	103	1	X.parietina GTX0501	Simple_repeat
(CTCCCAC)N#Simple_repeat	103	1	X.parietina GTX0501	Simple_repeat
(GGTGAG)N#Simple_repeat	103	2	X.parietina GTX0501	Simple_repeat
(TGGAGA)N#Simple_repeat	103	2	X.parietina GTX0501	Simple_repeat
(ACCCCC)N#Simple_repeat	102	3	X.parietina GTX0501	Simple_repeat
(GACTGG)N#Simple_repeat	102	2	X.parietina GTX0501	Simple_repeat
(GGAGAAGGC)N#Simple_repeat	102	1	X.parietina GTX0501	Simple_repeat
(ATTG)N#Simple_repeat	101	3	X.parietina GTX0501	Simple_repeat
(CAACCA)N#Simple_repeat	101	3	X.parietina GTX0501	Simple_repeat
(CTTCTC)N#Simple_repeat	101	2	X.parietina GTX0501	Simple_repeat
(TCCTC)N#Simple_repeat	101	2	X.parietina GTX0501	Simple_repeat
(CCCGCC)N#Simple_repeat	100	2	X.parietina GTX0501	Simple_repeat
(CCTCCTC)N#Simple_repeat	100	2	X.parietina GTX0501	Simple_repeat
(GGTT)N#Simple_repeat	100	3	X.parietina GTX0501	Simple_repeat
(GGTTGT)N#Simple_repeat	99	2	X.parietina GTX0501	Simple_repeat
(ACAT)N#Simple_repeat	98	2	X.parietina GTX0501	Simple_repeat
(CATACC)N#Simple_repeat	98	2	X.parietina GTX0501	Simple_repeat
(CCCTTT)N#Simple_repeat	98	2	X.parietina GTX0501	Simple_repeat
(TCCAGAG)N#Simple_repeat	98	1	X.parietina GTX0501	Simple_repeat
(TGAGAG)N#Simple_repeat	97	1	X.parietina GTX0501	Simple_repeat
(TTCCCCC)N#Simple_repeat	97	1	X.parietina GTX0501	Simple_repeat

(TTTCTC)N#Simple_repeat	97	3	X.parietina GTX0501	Simple_repeat
(AATG)N#Simple_repeat	96	3	X.parietina GTX0501	Simple_repeat
(AGAGCC)N#Simple_repeat	96	3	X.parietina GTX0501	Simple_repeat
(GAGGAC)N#Simple_repeat	96	3	X.parietina GTX0501	Simple_repeat
(TAT)N#Simple_repeat	96	3	X.parietina GTX0501	Simple_repeat
(CCCTCAA)N#Simple_repeat	95	1	X.parietina GTX0501	Simple_repeat
(CCTCGC)N#Simple_repeat	95	2	X.parietina GTX0501	Simple_repeat
(GGGAAG)N#Simple_repeat	95	2	X.parietina GTX0501	Simple_repeat
(TAA)N#Simple_repeat	95	3	X.parietina GTX0501	Simple_repeat
(CCGTCA)N#Simple_repeat	94	2	X.parietina GTX0501	Simple_repeat
(CTCCAC)N#Simple_repeat	94	2	X.parietina GTX0501	Simple_repeat
(CTTCC)N#Simple_repeat	94	2	X.parietina GTX0501	Simple_repeat
(GAGAT)N#Simple_repeat	94	3	X.parietina GTX0501	Simple_repeat
(GTCGCT)N#Simple_repeat	94	1	X.parietina GTX0501	Simple_repeat
(TAGAGGCAC)N#Simple_repeat	94	1	X.parietina GTX0501	Simple_repeat
(TCGTTATC)N#Simple_repeat	94	2	X.parietina GTX0501	Simple_repeat
(CCAGCA)N#Simple_repeat	93	2	X.parietina GTX0501	Simple_repeat
(CGCCCC)N#Simple_repeat	93	2	X.parietina GTX0501	Simple_repeat
(ACCCTA)N#Simple_repeat	92	1	X.parietina GTX0501	Simple_repeat
(AGATGG)N#Simple_repeat	92	2	X.parietina GTX0501	Simple_repeat
(CCCTCCC)N#Simple_repeat	92	2	X.parietina GTX0501	Simple_repeat
(CCTCTCC)N#Simple_repeat	92	1	X.parietina GTX0501	Simple_repeat
(GACA)N#Simple_repeat	92	2	X.parietina GTX0501	Simple_repeat
(TCTCCCA)N#Simple_repeat	92	2	X.parietina GTX0501	Simple_repeat
(ATCAC)N#Simple_repeat	91	1	X.parietina GTX0501	Simple_repeat
(CTTCTT)N#Simple_repeat	91	1	X.parietina GTX0501	Simple_repeat

(GAGGT)N#Simple_repeat	91	2	X.parietina GTX0501	Simple_repeat
(GATGG)N#Simple_repeat	91	2	X.parietina GTX0501	Simple_repeat
(GCTGAT)N#Simple_repeat	91	2	X.parietina GTX0501	Simple_repeat
(TCAC)N#Simple_repeat	91	3	X.parietina GTX0501	Simple_repeat
(CCATCTC)N#Simple_repeat	90	2	X.parietina GTX0501	Simple_repeat
(CCC)N#Simple_repeat	90	2	X.parietina GTX0501	Simple_repeat
(CCGCGCC)N#Simple_repeat	90	1	X.parietina GTX0501	Simple_repeat
(CGAGGAAA)N#Simple_repeat	90	1	X.parietina GTX0501	Simple_repeat
(CTCAC)N#Simple_repeat	90	2	X.parietina GTX0501	Simple_repeat
(GAGG)N#Simple_repeat	90	3	X.parietina GTX0501	Simple_repeat
(GATGAA)N#Simple_repeat	90	2	X.parietina GTX0501	Simple_repeat
(GGATG)N#Simple_repeat	90	3	X.parietina GTX0501	Simple_repeat
(GGTGCG)N#Simple_repeat	90	1	X.parietina GTX0501	Simple_repeat
(TATTAA)N#Simple_repeat	90	1	X.parietina GTX0501	Simple_repeat
(TCCATC)N#Simple_repeat	90	2	X.parietina GTX0501	Simple_repeat
(TGGCGT)N#Simple_repeat	90	2	X.parietina GTX0501	Simple_repeat
(TGGCT)N#Simple_repeat	90	2	X.parietina GTX0501	Simple_repeat
(ATTATATA)N#Simple_repeat	89	1	X.parietina GTX0501	Simple_repeat
(CCATCA)N#Simple_repeat	89	2	X.parietina GTX0501	Simple_repeat
(GAAGT)N#Simple_repeat	89	3	X.parietina GTX0501	Simple_repeat
(GGATA)N#Simple_repeat	89	2	X.parietina GTX0501	Simple_repeat
(GTGGAT)N#Simple_repeat	89	2	X.parietina GTX0501	Simple_repeat
(CTCCAT)N#Simple_repeat	88	1	X.parietina GTX0501	Simple_repeat
(GGTAT)N#Simple_repeat	88	2	X.parietina GTX0501	Simple_repeat
(ACCGCC)N#Simple_repeat	87	1	X.parietina GTX0501	Simple_repeat
(CACATC)N#Simple_repeat	87	2	X.parietina GTX0501	Simple_repeat

(TCCCTC)N#Simple_repeat	87	3	X.parietina GTX0501	Simple_repeat
(CACCCTT)N#Simple_repeat	86	2	X.parietina GTX0501	Simple_repeat
(CGACAG)N#Simple_repeat	86	1	X.parietina GTX0501	Simple_repeat
(TCGTCC)N#Simple_repeat	86	2	X.parietina GTX0501	Simple_repeat
(TGGTC)N#Simple_repeat	86	1	X.parietina GTX0501	Simple_repeat
(ACCTTT)N#Simple_repeat	85	1	X.parietina GTX0501	Simple_repeat
(ATCTGCA)N#Simple_repeat	85	2	X.parietina GTX0501	Simple_repeat
(TCCTCCA)N#Simple_repeat	85	2	X.parietina GTX0501	Simple_repeat
(TCTTT)N#Simple_repeat	85	2	X.parietina GTX0501	Simple_repeat
(TGAA)N#Simple_repeat	85	2	X.parietina GTX0501	Simple_repeat
(AATCTTT)N#Simple_repeat	84	1	X.parietina GTX0501	Simple_repeat
(CAACACC)N#Simple_repeat	84	2	X.parietina GTX0501	Simple_repeat
(TCTA)N#Simple_repeat	84	3	X.parietina GTX0501	Simple_repeat
(TGTGTA)N#Simple_repeat	84	2	X.parietina GTX0501	Simple_repeat
(CGCGAT)N#Simple_repeat	83	1	X.parietina GTX0501	Simple_repeat
(GCAAGAA)N#Simple_repeat	83	1	X.parietina GTX0501	Simple_repeat
(GGAGCT)N#Simple_repeat	83	2	X.parietina GTX0501	Simple_repeat
(GGCGAA)N#Simple_repeat	83	2	X.parietina GTX0501	Simple_repeat
(ACCTT)N#Simple_repeat	82	2	X.parietina GTX0501	Simple_repeat
(AGCAC)N#Simple_repeat	82	2	X.parietina GTX0501	Simple_repeat
(CATCAT)N#Simple_repeat	82	2	X.parietina GTX0501	Simple_repeat
(CCCCACT)N#Simple_repeat	82	1	X.parietina GTX0501	Simple_repeat
(CCGGCAT)N#Simple_repeat	82	1	X.parietina GTX0501	Simple_repeat
(CTCTTC)N#Simple_repeat	82	2	X.parietina GTX0501	Simple_repeat
(GCACCA)N#Simple_repeat	82	2	X.parietina GTX0501	Simple_repeat
(GGATTC)N#Simple_repeat	82	2	X.parietina GTX0501	Simple_repeat

(GTTGGT)N#Simple_repeat	82	2	X.parietina GTX0501	Simple_repeat
(AAGAAC)N#Simple_repeat	81	1	X.parietina GTX0501	Simple_repeat
(CTTCCCC)N#Simple_repeat	81	1	X.parietina GTX0501	Simple_repeat
(TCGACG)N#Simple_repeat	81	2	X.parietina GTX0501	Simple_repeat
(TGTA)N#Simple_repeat	81	3	X.parietina GTX0501	Simple_repeat
(CCCTTCCT)N#Simple_repeat	80	1	X.parietina GTX0501	Simple_repeat
(CCTCT)N#Simple_repeat	80	3	X.parietina GTX0501	Simple_repeat
(GTCAGCTTA)N#Simple_repeat	80	1	X.parietina GTX0501	Simple_repeat
(AGATA)N#Simple_repeat	79	2	X.parietina GTX0501	Simple_repeat
(CCTTCTTCC)N#Simple_repeat	79	1	X.parietina GTX0501	Simple_repeat
(GGATTG)N#Simple_repeat	79	2	X.parietina GTX0501	Simple_repeat
(TAGG)N#Simple_repeat	79	3	X.parietina GTX0501	Simple_repeat
(AGAA)N#Simple_repeat	78	3	X.parietina GTX0501	Simple_repeat
(AGGAT)N#Simple_repeat	78	1	X.parietina GTX0501	Simple_repeat
(ATACT)N#Simple_repeat	78	2	X.parietina GTX0501	Simple_repeat
(ATGAAG)N#Simple_repeat	78	2	X.parietina GTX0501	Simple_repeat
(ATTTCG)N#Simple_repeat	78	2	X.parietina GTX0501	Simple_repeat
(CAAT)N#Simple_repeat	78	3	X.parietina GTX0501	Simple_repeat
(CACAAGAAG)N#Simple_repeat	78	1	X.parietina GTX0501	Simple_repeat
(CACCCA)N#Simple_repeat	78	2	X.parietina GTX0501	Simple_repeat
(GCCATC)N#Simple_repeat	78	2	X.parietina GTX0501	Simple_repeat
(GTCTTC)N#Simple_repeat	78	2	X.parietina GTX0501	Simple_repeat
(AAGC)N#Simple_repeat	77	3	X.parietina GTX0501	Simple_repeat
(CAACAG)N#Simple_repeat	77	2	X.parietina GTX0501	Simple_repeat
(CCTCTC)N#Simple_repeat	77	2	X.parietina GTX0501	Simple_repeat
(TAATAA)N#Simple_repeat	77	1	X.parietina GTX0501	Simple_repeat

(TCGTC)N#Simple_repeat	77	2	X.parietina GTX0501	Simple_repeat
(TCTTCCTCT)N#Simple_repeat	77	1	X.parietina GTX0501	Simple_repeat
(CCAGCT)N#Simple_repeat	76	2	X.parietina GTX0501	Simple_repeat
(CTCCT)N#Simple_repeat	76	3	X.parietina GTX0501	Simple_repeat
(TCCCACC)N#Simple_repeat	76	2	X.parietina GTX0501	Simple_repeat
(TCCTTTC)N#Simple_repeat	76	1	X.parietina GTX0501	Simple_repeat
(TGCATAA)N#Simple_repeat	76	1	X.parietina GTX0501	Simple_repeat
(CAATC)N#Simple_repeat	75	2	X.parietina GTX0501	Simple_repeat
(CACAAC)N#Simple_repeat	75	2	X.parietina GTX0501	Simple_repeat
(CATCGC)N#Simple_repeat	75	2	X.parietina GTX0501	Simple_repeat
(CTTCT)N#Simple_repeat	75	2	X.parietina GTX0501	Simple_repeat
(GATGTAA)N#Simple_repeat	75	1	X.parietina GTX0501	Simple_repeat
(GCTCGT)N#Simple_repeat	75	2	X.parietina GTX0501	Simple_repeat
(GGAGTGG)N#Simple_repeat	75	2	X.parietina GTX0501	Simple_repeat
(GTAG)N#Simple_repeat	75	3	X.parietina GTX0501	Simple_repeat
(TTCCCC)N#Simple_repeat	75	2	X.parietina GTX0501	Simple_repeat
(TTTGAT)N#Simple_repeat	75	2	X.parietina GTX0501	Simple_repeat
(AACC)N#Simple_repeat	74	2	X.parietina GTX0501	Simple_repeat
(ACCCCGC)N#Simple_repeat	74	1	X.parietina GTX0501	Simple_repeat
(AGCGGT)N#Simple_repeat	74	1	X.parietina GTX0501	Simple_repeat
(ATTAT)N#Simple_repeat	74	2	X.parietina GTX0501	Simple_repeat
(CAGAAG)N#Simple_repeat	74	2	X.parietina GTX0501	Simple_repeat
(CATCCCC)N#Simple_repeat	74	1	X.parietina GTX0501	Simple_repeat
(TGAC)N#Simple_repeat	74	3	X.parietina GTX0501	Simple_repeat
(TTA)N#Simple_repeat	74	3	X.parietina GTX0501	Simple_repeat
(ACGCCG)N#Simple_repeat	73	1	X.parietina GTX0501	Simple_repeat

(AGACGA)N#Simple_repeat	73	2	X.parietina GTX0501	Simple_repeat
(ATCTTCC)N#Simple_repeat	73	1	X.parietina GTX0501	Simple_repeat
(GGTGA)N#Simple_repeat	73	2	X.parietina GTX0501	Simple_repeat
(TCACCA)N#Simple_repeat	73	1	X.parietina GTX0501	Simple_repeat
(TGCTGT)N#Simple_repeat	73	2	X.parietina GTX0501	Simple_repeat
(TTGTCA)N#Simple_repeat	73	2	X.parietina GTX0501	Simple_repeat
(AACCAA)N#Simple_repeat	72	2	X.parietina GTX0501	Simple_repeat
(ACGGAC)N#Simple_repeat	72	1	X.parietina GTX0501	Simple_repeat
(AGAGT)N#Simple_repeat	72	2	X.parietina GTX0501	Simple_repeat
(ATTTC)N#Simple_repeat	72	1	X.parietina GTX0501	Simple_repeat
(TCCT)N#Simple_repeat	72	3	X.parietina GTX0501	Simple_repeat
(AAGATC)N#Simple_repeat	71	1	X.parietina GTX0501	Simple_repeat
(CATCC)N#Simple_repeat	71	2	X.parietina GTX0501	Simple_repeat
(CATTTC)N#Simple_repeat	71	2	X.parietina GTX0501	Simple_repeat
(CCCACC)N#Simple_repeat	71	2	X.parietina GTX0501	Simple_repeat
(TATATAA)N#Simple_repeat	71	1	X.parietina GTX0501	Simple_repeat
(TTCTTT)N#Simple_repeat	71	2	X.parietina GTX0501	Simple_repeat
(CTA)N#Simple_repeat	70	1	X.parietina GTX0501	Simple_repeat
(GAAGCAGATC)N#Simple_repeat	70	1	X.parietina GTX0501	Simple_repeat
(GGCAATGA)N#Simple_repeat	70	1	X.parietina GTX0501	Simple_repeat
(TCTTCT)N#Simple_repeat	70	2	X.parietina GTX0501	Simple_repeat
(TGGC)N#Simple_repeat	70	3	X.parietina GTX0501	Simple_repeat
(ACCT)N#Simple_repeat	69	2	X.parietina GTX0501	Simple_repeat
(AGGAGC)N#Simple_repeat	69	1	X.parietina GTX0501	Simple_repeat
(AGGTGG)N#Simple_repeat	69	2	X.parietina GTX0501	Simple_repeat
(ATAT)N#Simple_repeat	69	2	X.parietina GTX0501	Simple_repeat

(CAACC)N#Simple_repeat	69	2	X.parietina GTX0501	Simple_repeat
(CCGCA)N#Simple_repeat	69	1	X.parietina GTX0501	Simple_repeat
(CGCGGT)N#Simple_repeat	69	1	X.parietina GTX0501	Simple_repeat
(GACCACC)N#Simple_repeat	69	1	X.parietina GTX0501	Simple_repeat
(GCGAGA)N#Simple_repeat	69	1	X.parietina GTX0501	Simple_repeat
(GGTTT)N#Simple_repeat	69	2	X.parietina GTX0501	Simple_repeat
(GTATTG)N#Simple_repeat	69	1	X.parietina GTX0501	Simple_repeat
(GTGATC)N#Simple_repeat	69	1	X.parietina GTX0501	Simple_repeat
(TATTATC)N#Simple_repeat	69	1	X.parietina GTX0501	Simple_repeat
(TCACGC)N#Simple_repeat	69	2	X.parietina GTX0501	Simple_repeat
(TCCCTT)N#Simple_repeat	69	1	X.parietina GTX0501	Simple_repeat
(AACTAT)N#Simple_repeat	68	1	X.parietina GTX0501	Simple_repeat
(ATCAGCATC)N#Simple_repeat	68	1	X.parietina GTX0501	Simple_repeat
(ATCCTC)N#Simple_repeat	68	1	X.parietina GTX0501	Simple_repeat
(CACCTCC)N#Simple_repeat	68	1	X.parietina GTX0501	Simple_repeat
(CAGA)N#Simple_repeat	68	2	X.parietina GTX0501	Simple_repeat
(CCACTG)N#Simple_repeat	68	1	X.parietina GTX0501	Simple_repeat
(CGGAGA)N#Simple_repeat	68	1	X.parietina GTX0501	Simple_repeat
(GAGTGGT)N#Simple_repeat	68	1	X.parietina GTX0501	Simple_repeat
(GCATCA)N#Simple_repeat	68	2	X.parietina GTX0501	Simple_repeat
(GGCGA)N#Simple_repeat	68	2	X.parietina GTX0501	Simple_repeat
(TCCTCA)N#Simple_repeat	68	2	X.parietina GTX0501	Simple_repeat
(TTTCT)N#Simple_repeat	68	2	X.parietina GTX0501	Simple_repeat
(ACCACG)N#Simple_repeat	67	2	X.parietina GTX0501	Simple_repeat
(AGAGC)N#Simple_repeat	67	2	X.parietina GTX0501	Simple_repeat
(CTCTCG)N#Simple_repeat	67	2	X.parietina GTX0501	Simple_repeat

(GAAGTCG)N#Simple_repeat	67	1	X.parietina GTX0501	Simple_repeat
(GGTGTG)N#Simple_repeat	67	1	X.parietina GTX0501	Simple_repeat
RND-1_FAMILY-95#LTR/Ty3	67	1	X.parietina GTX0501	LTR/Ty3
(ACCCTCT)N#Simple_repeat	66	1	X.parietina GTX0501	Simple_repeat
(CATCTG)N#Simple_repeat	66	1	X.parietina GTX0501	Simple_repeat
(CCAGAG)N#Simple_repeat	66	2	X.parietina GTX0501	Simple_repeat
(CTGG)N#Simple_repeat	66	3	X.parietina GTX0501	Simple_repeat
(GAAGGGA)N#Simple_repeat	66	1	X.parietina GTX0501	Simple_repeat
(GGCGGA)N#Simple_repeat	66	2	X.parietina GTX0501	Simple_repeat
(GTTGTT)N#Simple_repeat	66	1	X.parietina GTX0501	Simple_repeat
(TCGCAGGA)N#Simple_repeat	66	1	X.parietina GTX0501	Simple_repeat
(TGCA)N#Simple_repeat	66	3	X.parietina GTX0501	Simple_repeat
(TTTCC)N#Simple_repeat	66	2	X.parietina GTX0501	Simple_repeat
(ACCGACCCTC)N#Simple_repeat	65	1	X.parietina GTX0501	Simple_repeat
(AGGG)N#Simple_repeat	65	2	X.parietina GTX0501	Simple_repeat
(ATAGA)N#Simple_repeat	65	1	X.parietina GTX0501	Simple_repeat
(CACT)N#Simple_repeat	65	2	X.parietina GTX0501	Simple_repeat
(CAGGATG)N#Simple_repeat	65	1	X.parietina GTX0501	Simple_repeat
(CCCATTC)N#Simple_repeat	65	1	X.parietina GTX0501	Simple_repeat
(CCCCAC)N#Simple_repeat	65	1	X.parietina GTX0501	Simple_repeat
(CTGCCT)N#Simple_repeat	65	1	X.parietina GTX0501	Simple_repeat
(CTTCTCC)N#Simple_repeat	65	1	X.parietina GTX0501	Simple_repeat
(GGTC)N#Simple_repeat	65	2	X.parietina GTX0501	Simple_repeat
(GGTCGT)N#Simple_repeat	65	1	X.parietina GTX0501	Simple_repeat
(TCCCTCC)N#Simple_repeat	65	1	X.parietina GTX0501	Simple_repeat
(TCTTCCA)N#Simple_repeat	65	1	X.parietina GTX0501	Simple_repeat

(TTCCT)N#Simple_repeat	65	2	X.parietina GTX0501	Simple_repeat
(AATATA)N#Simple_repeat	64	1	X.parietina GTX0501	Simple_repeat
(AGCAGA)N#Simple_repeat	64	2	X.parietina GTX0501	Simple_repeat
(ATAATTT)N#Simple_repeat	64	1	X.parietina GTX0501	Simple_repeat
(CACCTTC)N#Simple_repeat	64	1	X.parietina GTX0501	Simple_repeat
(CACTCCC)N#Simple_repeat	64	1	X.parietina GTX0501	Simple_repeat
(CCAACGC)N#Simple_repeat	64	1	X.parietina GTX0501	Simple_repeat
(CTCCGT)N#Simple_repeat	64	1	X.parietina GTX0501	Simple_repeat
(CTGA)N#Simple_repeat	64	1	X.parietina GTX0501	Simple_repeat
(GGATTGT)N#Simple_repeat	64	1	X.parietina GTX0501	Simple_repeat
(GTTGA)N#Simple_repeat	64	2	X.parietina GTX0501	Simple_repeat
(TCTTTAC)N#Simple_repeat	64	1	X.parietina GTX0501	Simple_repeat
(TGACAA)N#Simple_repeat	64	1	X.parietina GTX0501	Simple_repeat
(AAAGA)N#Simple_repeat	63	2	X.parietina GTX0501	Simple_repeat
(ACTC)N#Simple_repeat	63	2	X.parietina GTX0501	Simple_repeat
(CACCCC)N#Simple_repeat	63	1	X.parietina GTX0501	Simple_repeat
(CATCTT)N#Simple_repeat	63	2	X.parietina GTX0501	Simple_repeat
(GGGAA)N#Simple_repeat	63	2	X.parietina GTX0501	Simple_repeat
(GTTGT)N#Simple_repeat	63	2	X.parietina GTX0501	Simple_repeat
(TACTG)N#Simple_repeat	63	2	X.parietina GTX0501	Simple_repeat
(TATCC)N#Simple_repeat	63	1	X.parietina GTX0501	Simple_repeat
(TGCCAC)N#Simple_repeat	63	2	X.parietina GTX0501	Simple_repeat
(AACGCCAG)N#Simple_repeat	62	1	X.parietina GTX0501	Simple_repeat
(CCCCCAG)N#Simple_repeat	62	1	X.parietina GTX0501	Simple_repeat
(CCTGTC)N#Simple_repeat	62	2	X.parietina GTX0501	Simple_repeat
(CGGAGT)N#Simple_repeat	62	1	X.parietina GTX0501	Simple_repeat

(CTGCC)N#Simple_repeat	62	1	X.parietina GTX0501	Simple_repeat
(GTACTCC)N#Simple_repeat	62	1	X.parietina GTX0501	Simple_repeat
(GTGA)N#Simple_repeat	62	2	X.parietina GTX0501	Simple_repeat
(TAGGATA)N#Simple_repeat	62	1	X.parietina GTX0501	Simple_repeat
(TCAAT)N#Simple_repeat	62	2	X.parietina GTX0501	Simple_repeat
(ACGGTGG)N#Simple_repeat	61	1	X.parietina GTX0501	Simple_repeat
(ATATT)N#Simple_repeat	61	1	X.parietina GTX0501	Simple_repeat
(ATT)N#Simple_repeat	61	2	X.parietina GTX0501	Simple_repeat
(CCATC)N#Simple_repeat	61	2	X.parietina GTX0501	Simple_repeat
(CGACATG)N#Simple_repeat	61	1	X.parietina GTX0501	Simple_repeat
(CTAC)N#Simple_repeat	61	2	X.parietina GTX0501	Simple_repeat
(CTATATA)N#Simple_repeat	61	1	X.parietina GTX0501	Simple_repeat
(CTCCCCA)N#Simple_repeat	61	1	X.parietina GTX0501	Simple_repeat
(CTCT)N#Simple_repeat	61	2	X.parietina GTX0501	Simple_repeat
(TATCTATTC)N#Simple_repeat	61	1	X.parietina GTX0501	Simple_repeat
(TCATAGCTTC)N#Simple_repeat	61	1	X.parietina GTX0501	Simple_repeat
(TGAGCT)N#Simple_repeat	61	1	X.parietina GTX0501	Simple_repeat
(TGCTGGG)N#Simple_repeat	61	1	X.parietina GTX0501	Simple_repeat
(CCCATC)N#Simple_repeat	60	1	X.parietina GTX0501	Simple_repeat
(CTATT)N#Simple_repeat	60	1	X.parietina GTX0501	Simple_repeat
(CTCGCC)N#Simple_repeat	60	1	X.parietina GTX0501	Simple_repeat
(GAATTGT)N#Simple_repeat	60	1	X.parietina GTX0501	Simple_repeat
(GACGGC)N#Simple_repeat	60	1	X.parietina GTX0501	Simple_repeat
(GATT)N#Simple_repeat	60	2	X.parietina GTX0501	Simple_repeat
(GCAGAAGA)N#Simple_repeat	60	1	X.parietina GTX0501	Simple_repeat
(GCTG)N#Simple_repeat	60	2	X.parietina GTX0501	Simple_repeat

(TACTT)N#Simple_repeat	60	1	X.parietina GTX0501	Simple_repeat
(TCCCCTCCA)N#Simple_repeat	60	1	X.parietina GTX0501	Simple_repeat
(ACCCCCC)N#Simple_repeat	59	1	X.parietina GTX0501	Simple_repeat
(ACCTACT)N#Simple_repeat	59	1	X.parietina GTX0501	Simple_repeat
(ACCTCC)N#Simple_repeat	59	1	X.parietina GTX0501	Simple_repeat
(CACCACC)N#Simple_repeat	59	1	X.parietina GTX0501	Simple_repeat
(CGGCGG)N#Simple_repeat	59	1	X.parietina GTX0501	Simple_repeat
(GAATCCC)N#Simple_repeat	59	1	X.parietina GTX0501	Simple_repeat
(GAGAAGT)N#Simple_repeat	59	1	X.parietina GTX0501	Simple_repeat
(GGTTTT)N#Simple_repeat	59	1	X.parietina GTX0501	Simple_repeat
(TCCAT)N#Simple_repeat	59	1	X.parietina GTX0501	Simple_repeat
(AACGA)N#Simple_repeat	58	2	X.parietina GTX0501	Simple_repeat
(AGTAG)N#Simple_repeat	58	2	X.parietina GTX0501	Simple_repeat
(CCACCAC)N#Simple_repeat	58	1	X.parietina GTX0501	Simple_repeat
(CCCTC)N#Simple_repeat	58	2	X.parietina GTX0501	Simple_repeat
(CTCTCTT)N#Simple_repeat	58	1	X.parietina GTX0501	Simple_repeat
(GACCGA)N#Simple_repeat	58	1	X.parietina GTX0501	Simple_repeat
(GGAGGTC)N#Simple_repeat	58	1	X.parietina GTX0501	Simple_repeat
(GTTG)N#Simple_repeat	58	2	X.parietina GTX0501	Simple_repeat
(TAGA)N#Simple_repeat	58	1	X.parietina GTX0501	Simple_repeat
(TTTTGTT)N#Simple_repeat	58	1	X.parietina GTX0501	Simple_repeat
(ATCCCT)N#Simple_repeat	57	1	X.parietina GTX0501	Simple_repeat
(CATTT)N#Simple_repeat	57	1	X.parietina GTX0501	Simple_repeat
(CCAAA)N#Simple_repeat	57	1	X.parietina GTX0501	Simple_repeat
(CCTCCAC)N#Simple_repeat	57	1	X.parietina GTX0501	Simple_repeat
(CCTCCG)N#Simple_repeat	57	1	X.parietina GTX0501	Simple_repeat

(CCTCGT)N#Simple_repeat	57	1	X.parietina GTX0501	Simple_repeat
(GAAGCG)N#Simple_repeat	57	1	X.parietina GTX0501	Simple_repeat
(GCACTC)N#Simple_repeat	57	1	X.parietina GTX0501	Simple_repeat
(GCAG)N#Simple_repeat	57	2	X.parietina GTX0501	Simple_repeat
(GCATGG)N#Simple_repeat	57	1	X.parietina GTX0501	Simple_repeat
(TCATCA)N#Simple_repeat	57	1	X.parietina GTX0501	Simple_repeat
(TCTCCCT)N#Simple_repeat	57	1	X.parietina GTX0501	Simple_repeat
(AATCTC)N#Simple_repeat	56	1	X.parietina GTX0501	Simple_repeat
(ACAACCCC)N#Simple_repeat	56	1	X.parietina GTX0501	Simple_repeat
(AGATGGA)N#Simple_repeat	56	1	X.parietina GTX0501	Simple_repeat
(AGCCT)N#Simple_repeat	56	1	X.parietina GTX0501	Simple_repeat
(ATAGATG)N#Simple_repeat	56	1	X.parietina GTX0501	Simple_repeat
(CAGCA)N#Simple_repeat	56	2	X.parietina GTX0501	Simple_repeat
(CCATT)N#Simple_repeat	56	2	X.parietina GTX0501	Simple_repeat
(CGGAGG)N#Simple_repeat	56	1	X.parietina GTX0501	Simple_repeat
(CTCCTTCTC)N#Simple_repeat	56	1	X.parietina GTX0501	Simple_repeat
(GAATTG)N#Simple_repeat	56	1	X.parietina GTX0501	Simple_repeat
(GAGAC)N#Simple_repeat	56	1	X.parietina GTX0501	Simple_repeat
(GAGGATG)N#Simple_repeat	56	1	X.parietina GTX0501	Simple_repeat
(TAAT)N#Simple_repeat	56	1	X.parietina GTX0501	Simple_repeat
(TCTCAAG)N#Simple_repeat	56	2	X.parietina GTX0501	Simple_repeat
(TGAAG)N#Simple_repeat	56	1	X.parietina GTX0501	Simple_repeat
(TTCT)N#Simple_repeat	56	2	X.parietina GTX0501	Simple_repeat
(AAGCCA)N#Simple_repeat	55	1	X.parietina GTX0501	Simple_repeat
(AGTGGTG)N#Simple_repeat	55	1	X.parietina GTX0501	Simple_repeat
(CATT)N#Simple_repeat	55	2	X.parietina GTX0501	Simple_repeat

(CCGCCT)N#Simple_repeat	55	1	X.parietina GTX0501	Simple_repeat
(CTTCCGT)N#Simple_repeat	55	1	X.parietina GTX0501	Simple_repeat
(GAGGTT)N#Simple_repeat	55	1	X.parietina GTX0501	Simple_repeat
(TATGATG)N#Simple_repeat	55	1	X.parietina GTX0501	Simple_repeat
(TCCCTCA)N#Simple_repeat	55	1	X.parietina GTX0501	Simple_repeat
(TGTC)N#Simple_repeat	55	2	X.parietina GTX0501	Simple_repeat
(AAGACG)N#Simple_repeat	54	1	X.parietina GTX0501	Simple_repeat
(ACTTCC)N#Simple_repeat	54	1	X.parietina GTX0501	Simple_repeat
(AGATTAT)N#Simple_repeat	54	1	X.parietina GTX0501	Simple_repeat
(ATGATTG)N#Simple_repeat	54	1	X.parietina GTX0501	Simple_repeat
(CACAACCAC)N#Simple_repeat	54	1	X.parietina GTX0501	Simple_repeat
(CACCAAC)N#Simple_repeat	54	1	X.parietina GTX0501	Simple_repeat
(CAGCTA)N#Simple_repeat	54	1	X.parietina GTX0501	Simple_repeat
(CATCAC)N#Simple_repeat	54	1	X.parietina GTX0501	Simple_repeat
(CATTCAT)N#Simple_repeat	54	1	X.parietina GTX0501	Simple_repeat
(CTCCTAC)N#Simple_repeat	54	1	X.parietina GTX0501	Simple_repeat
(GAGGCA)N#Simple_repeat	54	1	X.parietina GTX0501	Simple_repeat
(GATAT)N#Simple_repeat	54	1	X.parietina GTX0501	Simple_repeat
(GCCAAGAT)N#Simple_repeat	54	1	X.parietina GTX0501	Simple_repeat
(GCGGAG)N#Simple_repeat	54	1	X.parietina GTX0501	Simple_repeat
(GGCGAC)N#Simple_repeat	54	1	X.parietina GTX0501	Simple_repeat
(GGTG)N#Simple_repeat	54	1	X.parietina GTX0501	Simple_repeat
(GTGCG)N#Simple_repeat	54	1	X.parietina GTX0501	Simple_repeat
(TCACCC)N#Simple_repeat	54	1	X.parietina GTX0501	Simple_repeat
(TCCGAC)N#Simple_repeat	54	1	X.parietina GTX0501	Simple_repeat
(TCTTTTCT)N#Simple_repeat	54	1	X.parietina GTX0501	Simple_repeat

(TGAAGA)N#Simple_repeat	54	2	X.parietina GTX0501	Simple_repeat
(TGACTG)N#Simple_repeat	54	1	X.parietina GTX0501	Simple_repeat
(TGCTTT)N#Simple_repeat	54	1	X.parietina GTX0501	Simple_repeat
(CAACCCCG)N#Simple_repeat	53	1	X.parietina GTX0501	Simple_repeat
(CCAACTC)N#Simple_repeat	53	1	X.parietina GTX0501	Simple_repeat
(CGATCG)N#Simple_repeat	53	1	X.parietina GTX0501	Simple_repeat
(GAAT)N#Simple_repeat	53	2	X.parietina GTX0501	Simple_repeat
(GAATA)N#Simple_repeat	53	2	X.parietina GTX0501	Simple_repeat
(GAGGTA)N#Simple_repeat	53	1	X.parietina GTX0501	Simple_repeat
(GCCTCG)N#Simple_repeat	53	1	X.parietina GTX0501	Simple_repeat
(GGAGGTG)N#Simple_repeat	53	1	X.parietina GTX0501	Simple_repeat
(GTGGA)N#Simple_repeat	53	1	X.parietina GTX0501	Simple_repeat
(TCATCT)N#Simple_repeat	53	1	X.parietina GTX0501	Simple_repeat
(TGAAA)N#Simple_repeat	53	2	X.parietina GTX0501	Simple_repeat
(TGCTGCATC)N#Simple_repeat	53	1	X.parietina GTX0501	Simple_repeat
(TTGTGA)N#Simple_repeat	53	1	X.parietina GTX0501	Simple_repeat
(ATCTAAC)N#Simple_repeat	52	1	X.parietina GTX0501	Simple_repeat
(CAGATGGGG)N#Simple_repeat	52	1	X.parietina GTX0501	Simple_repeat
(CATCACC)N#Simple_repeat	52	1	X.parietina GTX0501	Simple_repeat
(CCCCAAC)N#Simple_repeat	52	1	X.parietina GTX0501	Simple_repeat
(CCCTCCT)N#Simple_repeat	52	1	X.parietina GTX0501	Simple_repeat
(CCTCCAT)N#Simple_repeat	52	1	X.parietina GTX0501	Simple_repeat
(CGTCAC)N#Simple_repeat	52	1	X.parietina GTX0501	Simple_repeat
(CTCTCCC)N#Simple_repeat	52	1	X.parietina GTX0501	Simple_repeat
(CTCTTCCATC)N#Simple_repeat	52	1	X.parietina GTX0501	Simple_repeat
(GCGACC)N#Simple_repeat	52	1	X.parietina GTX0501	Simple_repeat

(GCGGGA)N#Simple_repeat	52	1	X.parietina GTX0501	Simple_repeat
(GCTTTG)N#Simple_repeat	52	1	X.parietina GTX0501	Simple_repeat
(GTGTTGAT)N#Simple_repeat	52	1	X.parietina GTX0501	Simple_repeat
(TGCTTGG)N#Simple_repeat	52	1	X.parietina GTX0501	Simple_repeat
(TTTAATT)N#Simple_repeat	52	1	X.parietina GTX0501	Simple_repeat
(AACCACC)N#Simple_repeat	51	1	X.parietina GTX0501	Simple_repeat
(AATAAC)N#Simple_repeat	51	1	X.parietina GTX0501	Simple_repeat
(AGCC)N#Simple_repeat	51	2	X.parietina GTX0501	Simple_repeat
(AGTTGATG)N#Simple_repeat	51	1	X.parietina GTX0501	Simple_repeat
(ATCAT)N#Simple_repeat	51	1	X.parietina GTX0501	Simple_repeat
(ATCTTC)N#Simple_repeat	51	1	X.parietina GTX0501	Simple_repeat
(ATGTGT)N#Simple_repeat	51	1	X.parietina GTX0501	Simple_repeat
(CAACAA)N#Simple_repeat	51	1	X.parietina GTX0501	Simple_repeat
(CCCACTC)N#Simple_repeat	51	1	X.parietina GTX0501	Simple_repeat
(CCTA)N#Simple_repeat	51	2	X.parietina GTX0501	Simple_repeat
(CGCAGC)N#Simple_repeat	51	1	X.parietina GTX0501	Simple_repeat
(CGCCTCC)N#Simple_repeat	51	1	X.parietina GTX0501	Simple_repeat
(CTCATA)N#Simple_repeat	51	1	X.parietina GTX0501	Simple_repeat
(CTCCCTGTCC)N#Simple_repeat	51	1	X.parietina GTX0501	Simple_repeat
(GAATC)N#Simple_repeat	51	1	X.parietina GTX0501	Simple_repeat
(GAGGCC)N#Simple_repeat	51	1	X.parietina GTX0501	Simple_repeat
(GATA)N#Simple_repeat	51	2	X.parietina GTX0501	Simple_repeat
(GCAGGAG)N#Simple_repeat	51	1	X.parietina GTX0501	Simple_repeat
(GGCGCC)N#Simple_repeat	51	1	X.parietina GTX0501	Simple_repeat
(TGGTCGCT)N#Simple_repeat	51	1	X.parietina GTX0501	Simple_repeat
(ACCCACC)N#Simple_repeat	50	1	X.parietina GTX0501	Simple_repeat

(CACGATCCA)N#Simple_repeat	50	1	X.parietina GTX0501	Simple_repeat
(CATACTC)N#Simple_repeat	50	1	X.parietina GTX0501	Simple_repeat
(CGCGGCT)N#Simple_repeat	50	1	X.parietina GTX0501	Simple_repeat
(CGTCAT)N#Simple_repeat	50	2	X.parietina GTX0501	Simple_repeat
(CTCTACA)N#Simple_repeat	50	1	X.parietina GTX0501	Simple_repeat
(GGAGATA)N#Simple_repeat	50	1	X.parietina GTX0501	Simple_repeat
(GGAGT)N#Simple_repeat	50	1	X.parietina GTX0501	Simple_repeat
(TATA)N#Simple_repeat	50	1	X.parietina GTX0501	Simple_repeat
(TGGAGG)N#Simple_repeat	50	2	X.parietina GTX0501	Simple_repeat
(TGGAGGC)N#Simple_repeat	50	1	X.parietina GTX0501	Simple_repeat
(ACACCC)N#Simple_repeat	49	1	X.parietina GTX0501	Simple_repeat
(ACAGA)N#Simple_repeat	49	1	X.parietina GTX0501	Simple_repeat
(AGCG)N#Simple_repeat	49	2	X.parietina GTX0501	Simple_repeat
(AGTGCC)N#Simple_repeat	49	1	X.parietina GTX0501	Simple_repeat
(ATGCAGA)N#Simple_repeat	49	1	X.parietina GTX0501	Simple_repeat
(CAGAGA)N#Simple_repeat	49	1	X.parietina GTX0501	Simple_repeat
(CCAAAGGACG)N#Simple_repeat	49	1	X.parietina GTX0501	Simple_repeat
(CCCA)N#Simple_repeat	49	1	X.parietina GTX0501	Simple_repeat
(CCCGATC)N#Simple_repeat	49	1	X.parietina GTX0501	Simple_repeat
(CCGAGA)N#Simple_repeat	49	1	X.parietina GTX0501	Simple_repeat
(CGGCGC)N#Simple_repeat	49	1	X.parietina GTX0501	Simple_repeat
(CGTCGC)N#Simple_repeat	49	1	X.parietina GTX0501	Simple_repeat
(CTTCCCT)N#Simple_repeat	49	1	X.parietina GTX0501	Simple_repeat
(CTTTTTT)N#Simple_repeat	49	1	X.parietina GTX0501	Simple_repeat
(GCAACA)N#Simple_repeat	49	2	X.parietina GTX0501	Simple_repeat
(GCTT)N#Simple_repeat	49	2	X.parietina GTX0501	Simple_repeat

(GGAGCA)N#Simple_repeat	49	1	X.parietina GTX0501	Simple_repeat
(GGATGG)N#Simple_repeat	49	1	X.parietina GTX0501	Simple_repeat
(GGCA)N#Simple_repeat	49	2	X.parietina GTX0501	Simple_repeat
(GGTTG)N#Simple_repeat	49	1	X.parietina GTX0501	Simple_repeat
(TACTAA)N#Simple_repeat	49	1	X.parietina GTX0501	Simple_repeat
(TCTCCG)N#Simple_repeat	49	1	X.parietina GTX0501	Simple_repeat
(TGAGTGG)N#Simple_repeat	49	1	X.parietina GTX0501	Simple_repeat
(TTGG)N#Simple_repeat	49	2	X.parietina GTX0501	Simple_repeat
(TTTCCCT)N#Simple_repeat	49	1	X.parietina GTX0501	Simple_repeat
(AACACC)N#Simple_repeat	48	1	X.parietina GTX0501	Simple_repeat
(AAGAGG)N#Simple_repeat	48	1	X.parietina GTX0501	Simple_repeat
(ACCAACAAT)N#Simple_repeat	48	1	X.parietina GTX0501	Simple_repeat
(AGAATGG)N#Simple_repeat	48	1	X.parietina GTX0501	Simple_repeat
(CATCTA)N#Simple_repeat	48	1	X.parietina GTX0501	Simple_repeat
(CCTCCTA)N#Simple_repeat	48	1	X.parietina GTX0501	Simple_repeat
(CTCCTCC)N#Simple_repeat	48	1	X.parietina GTX0501	Simple_repeat
(GAATCC)N#Simple_repeat	48	1	X.parietina GTX0501	Simple_repeat
(GAGAGCA)N#Simple_repeat	48	1	X.parietina GTX0501	Simple_repeat
(GCCTCA)N#Simple_repeat	48	1	X.parietina GTX0501	Simple_repeat
(GCGAT)N#Simple_repeat	48	1	X.parietina GTX0501	Simple_repeat
(GCGG)N#Simple_repeat	48	1	X.parietina GTX0501	Simple_repeat
(GGGA)N#Simple_repeat	48	2	X.parietina GTX0501	Simple_repeat
(GTTGAG)N#Simple_repeat	48	1	X.parietina GTX0501	Simple_repeat
(TCCCTCCTT)N#Simple_repeat	48	1	X.parietina GTX0501	Simple_repeat
(TGGAGGA)N#Simple_repeat	48	1	X.parietina GTX0501	Simple_repeat
(AGGGG)N#Simple_repeat	47	2	X.parietina GTX0501	Simple_repeat

(AGGGGA)N#Simple_repeat	47	1	X.parietina GTX0501	Simple_repeat
(AGTACTC)N#Simple_repeat	47	1	X.parietina GTX0501	Simple_repeat
(ATGCT)N#Simple_repeat	47	1	X.parietina GTX0501	Simple_repeat
(ATTCTC)N#Simple_repeat	47	1	X.parietina GTX0501	Simple_repeat
(CATAC)N#Simple_repeat	47	1	X.parietina GTX0501	Simple_repeat
(CCCCC)N#Simple_repeat	47	1	X.parietina GTX0501	Simple_repeat
(CTCCCAT)N#Simple_repeat	47	1	X.parietina GTX0501	Simple_repeat
(CTTG)N#Simple_repeat	47	1	X.parietina GTX0501	Simple_repeat
(CTTTACC)N#Simple_repeat	47	1	X.parietina GTX0501	Simple_repeat
(GCCTCCCAT)N#Simple_repeat	47	1	X.parietina GTX0501	Simple_repeat
(TATAGA)N#Simple_repeat	47	1	X.parietina GTX0501	Simple_repeat
(TCATTC)N#Simple_repeat	47	1	X.parietina GTX0501	Simple_repeat
(TCGTAT)N#Simple_repeat	47	1	X.parietina GTX0501	Simple_repeat
(TTGC)N#Simple_repeat	47	2	X.parietina GTX0501	Simple_repeat
(TTTCG)N#Simple_repeat	47	1	X.parietina GTX0501	Simple_repeat
(AAGTTGG)N#Simple_repeat	46	1	X.parietina GTX0501	Simple_repeat
(ACGTCC)N#Simple_repeat	46	1	X.parietina GTX0501	Simple_repeat
(ACT)N#Simple_repeat	46	2	X.parietina GTX0501	Simple_repeat
(AGAAG)N#Simple_repeat	46	1	X.parietina GTX0501	Simple_repeat
(ATGCTGGGG)N#Simple_repeat	46	1	X.parietina GTX0501	Simple_repeat
(CACCTC)N#Simple_repeat	46	1	X.parietina GTX0501	Simple_repeat
(CACGA)N#Simple_repeat	46	1	X.parietina GTX0501	Simple_repeat
(CAGCACTAC)N#Simple_repeat	46	1	X.parietina GTX0501	Simple_repeat
(CCTCTA)N#Simple_repeat	46	1	X.parietina GTX0501	Simple_repeat
(CGACAC)N#Simple_repeat	46	1	X.parietina GTX0501	Simple_repeat
(CTCAAAT)N#Simple_repeat	46	1	X.parietina GTX0501	Simple_repeat

(CTCCA)N#Simple_repeat	46	1	X.parietina GTX0501	Simple_repeat
(CTCTTCC)N#Simple_repeat	46	1	X.parietina GTX0501	Simple_repeat
(GCCTCTG)N#Simple_repeat	46	1	X.parietina GTX0501	Simple_repeat
(GCGGCT)N#Simple_repeat	46	1	X.parietina GTX0501	Simple_repeat
(GGCAAC)N#Simple_repeat	46	1	X.parietina GTX0501	Simple_repeat
(GTGTGG)N#Simple_repeat	46	1	X.parietina GTX0501	Simple_repeat
(TAGATTA)N#Simple_repeat	46	1	X.parietina GTX0501	Simple_repeat
(TCCCGA)N#Simple_repeat	46	1	X.parietina GTX0501	Simple_repeat
(TCGACGATC)N#Simple_repeat	46	1	X.parietina GTX0501	Simple_repeat
(TTAGTTA)N#Simple_repeat	46	1	X.parietina GTX0501	Simple_repeat
(TTCCC)N#Simple_repeat	46	1	X.parietina GTX0501	Simple_repeat
(AATAG)N#Simple_repeat	45	1	X.parietina GTX0501	Simple_repeat
(AATCGG)N#Simple_repeat	45	1	X.parietina GTX0501	Simple_repeat
(ACCATCG)N#Simple_repeat	45	1	X.parietina GTX0501	Simple_repeat
(ACTCCTC)N#Simple_repeat	45	1	X.parietina GTX0501	Simple_repeat
(AGAGGTG)N#Simple_repeat	45	1	X.parietina GTX0501	Simple_repeat
(CACCCCTGAA)N#Simple_repeat	45	1	X.parietina GTX0501	Simple_repeat
(CACCCGCT)N#Simple_repeat	45	1	X.parietina GTX0501	Simple_repeat
(CAGCCA)N#Simple_repeat	45	1	X.parietina GTX0501	Simple_repeat
(CCATAC)N#Simple_repeat	45	1	X.parietina GTX0501	Simple_repeat
(CCGCCGC)N#Simple_repeat	45	1	X.parietina GTX0501	Simple_repeat
(CCTACA)N#Simple_repeat	45	1	X.parietina GTX0501	Simple_repeat
(CCTCCCCCT)N#Simple_repeat	45	1	X.parietina GTX0501	Simple_repeat
(CCTTCAT)N#Simple_repeat	45	1	X.parietina GTX0501	Simple_repeat
(CGAACC)N#Simple_repeat	45	1	X.parietina GTX0501	Simple_repeat
(CTTAC)N#Simple_repeat	45	1	X.parietina GTX0501	Simple_repeat

(CTTCTAC)N#Simple_repeat	45	1	X.parietina GTX0501	Simple_repeat
(GAAGATC)N#Simple_repeat	45	1	X.parietina GTX0501	Simple_repeat
(GCTGCTG)N#Simple_repeat	45	1	X.parietina GTX0501	Simple_repeat
(GGTCGC)N#Simple_repeat	45	1	X.parietina GTX0501	Simple_repeat
(GGTGGATGG)N#Simple_repeat	45	1	X.parietina GTX0501	Simple_repeat
(GGTGTT)N#Simple_repeat	45	2	X.parietina GTX0501	Simple_repeat
(TGCCACCCCC)N#Simple_repeat	45	1	X.parietina GTX0501	Simple_repeat
(TTCTGTG)N#Simple_repeat	45	1	X.parietina GTX0501	Simple_repeat
(ACCATCATT)N#Simple_repeat	44	1	X.parietina GTX0501	Simple_repeat
(ACTGACT)N#Simple_repeat	44	1	X.parietina GTX0501	Simple_repeat
(AGCTGA)N#Simple_repeat	44	1	X.parietina GTX0501	Simple_repeat
(AGCTTC)N#Simple_repeat	44	1	X.parietina GTX0501	Simple_repeat
(AGGGACAGGG)N#Simple_repeat	44	1	X.parietina GTX0501	Simple_repeat
(AGGTCG)N#Simple_repeat	44	1	X.parietina GTX0501	Simple_repeat
(ATA)N#Simple_repeat	44	2	X.parietina GTX0501	Simple_repeat
(CACACC)N#Simple_repeat	44	1	X.parietina GTX0501	Simple_repeat
(CACCAC)N#Simple_repeat	44	1	X.parietina GTX0501	Simple_repeat
(CAGGA)N#Simple_repeat	44	1	X.parietina GTX0501	Simple_repeat
(CATCCTC)N#Simple_repeat	44	1	X.parietina GTX0501	Simple_repeat
(CCCCGA)N#Simple_repeat	44	1	X.parietina GTX0501	Simple_repeat
(CCGTCG)N#Simple_repeat	44	1	X.parietina GTX0501	Simple_repeat
(CCTGAT)N#Simple_repeat	44	1	X.parietina GTX0501	Simple_repeat
(CGAAGA)N#Simple_repeat	44	1	X.parietina GTX0501	Simple_repeat
(CTCGGT)N#Simple_repeat	44	1	X.parietina GTX0501	Simple_repeat
(CTCTTCT)N#Simple_repeat	44	1	X.parietina GTX0501	Simple_repeat
(CTTCCTT)N#Simple_repeat	44	1	X.parietina GTX0501	Simple_repeat

(CTTCTCA)N#Simple_repeat	44	1	X.parietina GTX0501	Simple_repeat
(GAGA)N#Simple_repeat	44	1	X.parietina GTX0501	Simple_repeat
(GAGT)N#Simple_repeat	44	2	X.parietina GTX0501	Simple_repeat
(GCAACG)N#Simple_repeat	44	1	X.parietina GTX0501	Simple_repeat
(GCCAAC)N#Simple_repeat	44	1	X.parietina GTX0501	Simple_repeat
(GCTTC)N#Simple_repeat	44	1	X.parietina GTX0501	Simple_repeat
(GCTTCTA)N#Simple_repeat	44	1	X.parietina GTX0501	Simple_repeat
(GTGGTT)N#Simple_repeat	44	1	X.parietina GTX0501	Simple_repeat
(TATCTGAA)N#Simple_repeat	44	1	X.parietina GTX0501	Simple_repeat
(TCCTCCC)N#Simple_repeat	44	1	X.parietina GTX0501	Simple_repeat
(TCTTTTG)N#Simple_repeat	44	1	X.parietina GTX0501	Simple_repeat
(TGGG)N#Simple_repeat	44	2	X.parietina GTX0501	Simple_repeat
(TTT)N#Simple_repeat	44	1	X.parietina GTX0501	Simple_repeat
(AGTCT)N#Simple_repeat	43	1	X.parietina GTX0501	Simple_repeat
(CAACAAC)N#Simple_repeat	43	1	X.parietina GTX0501	Simple_repeat
(CCCGAA)N#Simple_repeat	43	1	X.parietina GTX0501	Simple_repeat
(CCTCGA)N#Simple_repeat	43	1	X.parietina GTX0501	Simple_repeat
(CGCCTG)N#Simple_repeat	43	1	X.parietina GTX0501	Simple_repeat
(CTGGTG)N#Simple_repeat	43	1	X.parietina GTX0501	Simple_repeat
(CTTCTCTT)N#Simple_repeat	43	1	X.parietina GTX0501	Simple_repeat
(CTTTGC)N#Simple_repeat	43	1	X.parietina GTX0501	Simple_repeat
(CTTTGCG)N#Simple_repeat	43	1	X.parietina GTX0501	Simple_repeat
(GAACA)N#Simple_repeat	43	1	X.parietina GTX0501	Simple_repeat
(GACTTGG)N#Simple_repeat	43	1	X.parietina GTX0501	Simple_repeat
(GCCC)N#Simple_repeat	43	1	X.parietina GTX0501	Simple_repeat
(GCTCAA)N#Simple_repeat	43	1	X.parietina GTX0501	Simple_repeat

(GGTTTTG)N#Simple_repeat	43	1	X.parietina GTX0501	Simple_repeat
(GTAGCG)N#Simple_repeat	43	1	X.parietina GTX0501	Simple_repeat
(TCATCATCG)N#Simple_repeat	43	1	X.parietina GTX0501	Simple_repeat
(TCGGCA)N#Simple_repeat	43	1	X.parietina GTX0501	Simple_repeat
(TGATTT)N#Simple_repeat	43	1	X.parietina GTX0501	Simple_repeat
(TGCTGG)N#Simple_repeat	43	1	X.parietina GTX0501	Simple_repeat
(TGTGGAG)N#Simple_repeat	43	1	X.parietina GTX0501	Simple_repeat
(TTTACT)N#Simple_repeat	43	1	X.parietina GTX0501	Simple_repeat
(TTTATTA)N#Simple_repeat	43	1	X.parietina GTX0501	Simple_repeat
(ACCGCCCAAG)N#Simple_repeat	42	1	X.parietina GTX0501	Simple_repeat
(AGCCGG)N#Simple_repeat	42	1	X.parietina GTX0501	Simple_repeat
(AGCTTTT)N#Simple_repeat	42	1	X.parietina GTX0501	Simple_repeat
(ATAATT)N#Simple_repeat	42	1	X.parietina GTX0501	Simple_repeat
(ATCAGGT)N#Simple_repeat	42	1	X.parietina GTX0501	Simple_repeat
(ATCCTCA)N#Simple_repeat	42	1	X.parietina GTX0501	Simple_repeat
(CAACCC)N#Simple_repeat	42	1	X.parietina GTX0501	Simple_repeat
(CAACGGTAA)N#Simple_repeat	42	1	X.parietina GTX0501	Simple_repeat
(CAGAA)N#Simple_repeat	42	1	X.parietina GTX0501	Simple_repeat
(CCGACT)N#Simple_repeat	42	1	X.parietina GTX0501	Simple_repeat
(CCTGC)N#Simple_repeat	42	1	X.parietina GTX0501	Simple_repeat
(CGAAT)N#Simple_repeat	42	1	X.parietina GTX0501	Simple_repeat
(GAAAGG)N#Simple_repeat	42	1	X.parietina GTX0501	Simple_repeat
(GAGCGG)N#Simple_repeat	42	1	X.parietina GTX0501	Simple_repeat
(GATCC)N#Simple_repeat	42	1	X.parietina GTX0501	Simple_repeat
(GCGGTT)N#Simple_repeat	42	1	X.parietina GTX0501	Simple_repeat
(GTAACCACC)N#Simple_repeat	42	1	X.parietina GTX0501	Simple_repeat

(TAGTCA)N#Simple_repeat	42	1	X.parietina GTX0501	Simple_repeat
(TATG)N#Simple_repeat	42	1	X.parietina GTX0501	Simple_repeat
(TCCCATTA)N#Simple_repeat	42	1	X.parietina GTX0501	Simple_repeat
(TCGC)N#Simple_repeat	42	1	X.parietina GTX0501	Simple_repeat
(TGAGAGG)N#Simple_repeat	42	1	X.parietina GTX0501	Simple_repeat
(TTGTTG)N#Simple_repeat	42	1	X.parietina GTX0501	Simple_repeat
(TTTCGACT)N#Simple_repeat	42	1	X.parietina GTX0501	Simple_repeat
(AACCCCC)N#Simple_repeat	41	1	X.parietina GTX0501	Simple_repeat
(AACT)N#Simple_repeat	41	1	X.parietina GTX0501	Simple_repeat
(ACACCCA)N#Simple_repeat	41	1	X.parietina GTX0501	Simple_repeat
(ATCCCC)N#Simple_repeat	41	1	X.parietina GTX0501	Simple_repeat
(CACCGA)N#Simple_repeat	41	1	X.parietina GTX0501	Simple_repeat
(CAGT)N#Simple_repeat	41	1	X.parietina GTX0501	Simple_repeat
(CATCACGC)N#Simple_repeat	41	1	X.parietina GTX0501	Simple_repeat
(CATCAG)N#Simple_repeat	41	1	X.parietina GTX0501	Simple_repeat
(CATTGA)N#Simple_repeat	41	1	X.parietina GTX0501	Simple_repeat
(CCAACAC)N#Simple_repeat	41	1	X.parietina GTX0501	Simple_repeat
(CCTTCCC)N#Simple_repeat	41	1	X.parietina GTX0501	Simple_repeat
(CGCGAGA)N#Simple_repeat	41	1	X.parietina GTX0501	Simple_repeat
(CTCGGA)N#Simple_repeat	41	1	X.parietina GTX0501	Simple_repeat
(GACCTT)N#Simple_repeat	41	1	X.parietina GTX0501	Simple_repeat
(GCCCCTGA)N#Simple_repeat	41	1	X.parietina GTX0501	Simple_repeat
(GCCGAA)N#Simple_repeat	41	1	X.parietina GTX0501	Simple_repeat
(GCCGTC)N#Simple_repeat	41	1	X.parietina GTX0501	Simple_repeat
(GCTGTTGTT)N#Simple_repeat	41	1	X.parietina GTX0501	Simple_repeat
(GGACCA)N#Simple_repeat	41	1	X.parietina GTX0501	Simple_repeat

(GGACGT)N#Simple_repeat	41	1	X.parietina GTX0501	Simple_repeat
(GGAGAGA)N#Simple_repeat	41	1	X.parietina GTX0501	Simple_repeat
(GGAGTAAG)N#Simple_repeat	41	1	X.parietina GTX0501	Simple_repeat
(TACTCTC)N#Simple_repeat	41	1	X.parietina GTX0501	Simple_repeat
(TCACCG)N#Simple_repeat	41	1	X.parietina GTX0501	Simple_repeat
(TCCAGAC)N#Simple_repeat	41	1	X.parietina GTX0501	Simple_repeat
(TGAGA)N#Simple_repeat	41	1	X.parietina GTX0501	Simple_repeat
(TGATGGT)N#Simple_repeat	41	1	X.parietina GTX0501	Simple_repeat
(TT)N#Simple_repeat	41	1	X.parietina GTX0501	Simple_repeat
(AACACCC)N#Simple_repeat	40	1	X.parietina GTX0501	Simple_repeat
(AATAATA)N#Simple_repeat	40	1	X.parietina GTX0501	Simple_repeat
(ACACCA)N#Simple_repeat	40	1	X.parietina GTX0501	Simple_repeat
(AGCTAA)N#Simple_repeat	40	1	X.parietina GTX0501	Simple_repeat
(AGTAC)N#Simple_repeat	40	1	X.parietina GTX0501	Simple_repeat
(AGTGCTG)N#Simple_repeat	40	1	X.parietina GTX0501	Simple_repeat
(ATTAGT)N#Simple_repeat	40	1	X.parietina GTX0501	Simple_repeat
(CAACAC)N#Simple_repeat	40	1	X.parietina GTX0501	Simple_repeat
(CACAGA)N#Simple_repeat	40	1	X.parietina GTX0501	Simple_repeat
(CACCATT)N#Simple_repeat	40	1	X.parietina GTX0501	Simple_repeat
(CATCCAGG)N#Simple_repeat	40	1	X.parietina GTX0501	Simple_repeat
(CATGGTGGG)N#Simple_repeat	40	1	X.parietina GTX0501	Simple_repeat
(CCAACCC)N#Simple_repeat	40	1	X.parietina GTX0501	Simple_repeat
(CCCATCT)N#Simple_repeat	40	1	X.parietina GTX0501	Simple_repeat
(CCGCGC)N#Simple_repeat	40	1	X.parietina GTX0501	Simple_repeat
(CTCCCCT)N#Simple_repeat	40	1	X.parietina GTX0501	Simple_repeat
(CTTCGG)N#Simple_repeat	40	1	X.parietina GTX0501	Simple_repeat

(GAAGCA)N#Simple_repeat	40	1	X.parietina GTX0501	Simple_repeat
(GATGACGAT)N#Simple_repeat	40	1	X.parietina GTX0501	Simple_repeat
(GGATAGAGT)N#Simple_repeat	40	1	X.parietina GTX0501	Simple_repeat
(GGTGCC)N#Simple_repeat	40	1	X.parietina GTX0501	Simple_repeat
(GGTGGCGGA)N#Simple_repeat	40	1	X.parietina GTX0501	Simple_repeat
(GTGAGGAT)N#Simple_repeat	40	1	X.parietina GTX0501	Simple_repeat
(TCCTTCT)N#Simple_repeat	40	1	X.parietina GTX0501	Simple_repeat
(TCGCCT)N#Simple_repeat	40	1	X.parietina GTX0501	Simple_repeat
(TCGGCT)N#Simple_repeat	40	1	X.parietina GTX0501	Simple_repeat
(TCTCA)N#Simple_repeat	40	1	X.parietina GTX0501	Simple_repeat
(TCTTGC)N#Simple_repeat	40	1	X.parietina GTX0501	Simple_repeat
(TGGATG)N#Simple_repeat	40	1	X.parietina GTX0501	Simple_repeat
(TTCTTCA)N#Simple_repeat	40	1	X.parietina GTX0501	Simple_repeat
(TTGGTT)N#Simple_repeat	40	1	X.parietina GTX0501	Simple_repeat
(TTTCCCCT)N#Simple_repeat	40	1	X.parietina GTX0501	Simple_repeat
(TTTTCT)N#Simple_repeat	40	1	X.parietina GTX0501	Simple_repeat
(AAGCTG)N#Simple_repeat	39	1	X.parietina GTX0501	Simple_repeat
(AATCAACG)N#Simple_repeat	39	1	X.parietina GTX0501	Simple_repeat
(AATTCG)N#Simple_repeat	39	1	X.parietina GTX0501	Simple_repeat
(AATTTA)N#Simple_repeat	39	1	X.parietina GTX0501	Simple_repeat
(ACACACC)N#Simple_repeat	39	1	X.parietina GTX0501	Simple_repeat
(ACATCC)N#Simple_repeat	39	1	X.parietina GTX0501	Simple_repeat
(ACATCTG)N#Simple_repeat	39	1	X.parietina GTX0501	Simple_repeat
(ACCAGC)N#Simple_repeat	39	1	X.parietina GTX0501	Simple_repeat
(ACCCTCA)N#Simple_repeat	39	1	X.parietina GTX0501	Simple_repeat
(AGGAGA)N#Simple_repeat	39	1	X.parietina GTX0501	Simple_repeat

(ATCTCAA)N#Simple_repeat	39	1	X.parietina GTX0501	Simple_repeat
(ATTAG)N#Simple_repeat	39	1	X.parietina GTX0501	Simple_repeat
(CATCGT)N#Simple_repeat	39	1	X.parietina GTX0501	Simple_repeat
(CCCAAT)N#Simple_repeat	39	1	X.parietina GTX0501	Simple_repeat
(CCCTTTC)N#Simple_repeat	39	1	X.parietina GTX0501	Simple_repeat
(CCTCAA)N#Simple_repeat	39	1	X.parietina GTX0501	Simple_repeat
(CGTTAT)N#Simple_repeat	39	1	X.parietina GTX0501	Simple_repeat
(CTCCCA)N#Simple_repeat	39	1	X.parietina GTX0501	Simple_repeat
(CTCCGA)N#Simple_repeat	39	1	X.parietina GTX0501	Simple_repeat
(GAAGAAT)N#Simple_repeat	39	1	X.parietina GTX0501	Simple_repeat
(GAATGAA)N#Simple_repeat	39	1	X.parietina GTX0501	Simple_repeat
(GATGGTT)N#Simple_repeat	39	1	X.parietina GTX0501	Simple_repeat
(GCCACT)N#Simple_repeat	39	1	X.parietina GTX0501	Simple_repeat
(GCTGGTTG)N#Simple_repeat	39	1	X.parietina GTX0501	Simple_repeat
(GGCGGCGGT)N#Simple_repeat	39	1	X.parietina GTX0501	Simple_repeat
(GTCTTCC)N#Simple_repeat	39	1	X.parietina GTX0501	Simple_repeat
(GTTGAAG)N#Simple_repeat	39	1	X.parietina GTX0501	Simple_repeat
(GTTGGC)N#Simple_repeat	39	1	X.parietina GTX0501	Simple_repeat
(TATCAC)N#Simple_repeat	39	1	X.parietina GTX0501	Simple_repeat
(TCAACC)N#Simple_repeat	39	1	X.parietina GTX0501	Simple_repeat
(TCTAA)N#Simple_repeat	39	1	X.parietina GTX0501	Simple_repeat
(TCTCTCCC)N#Simple_repeat	39	1	X.parietina GTX0501	Simple_repeat
(TCTCTGT)N#Simple_repeat	39	1	X.parietina GTX0501	Simple_repeat
(TGCCC)N#Simple_repeat	39	1	X.parietina GTX0501	Simple_repeat
(TGTCC)N#Simple_repeat	39	1	X.parietina GTX0501	Simple_repeat
(TGTTGT)N#Simple_repeat	39	1	X.parietina GTX0501	Simple_repeat

(TTATAA)N#Simple_repeat	39	1	X.parietina GTX0501	Simple_repeat
(TTGCTC)N#Simple_repeat	39	1	X.parietina GTX0501	Simple_repeat
(TTGTCGT)N#Simple_repeat	39	1	X.parietina GTX0501	Simple_repeat
(AAGGCAG)N#Simple_repeat	38	1	X.parietina GTX0501	Simple_repeat
(ACCCATCC)N#Simple_repeat	38	1	X.parietina GTX0501	Simple_repeat
(ACGCAA)N#Simple_repeat	38	1	X.parietina GTX0501	Simple_repeat
(ATGCCA)N#Simple_repeat	38	1	X.parietina GTX0501	Simple_repeat
(ATGTCC)N#Simple_repeat	38	1	X.parietina GTX0501	Simple_repeat
(CAAGAA)N#Simple_repeat	38	1	X.parietina GTX0501	Simple_repeat
(CAGGAG)N#Simple_repeat	38	1	X.parietina GTX0501	Simple_repeat
(CCACAT)N#Simple_repeat	38	1	X.parietina GTX0501	Simple_repeat
(CCACCTT)N#Simple_repeat	38	1	X.parietina GTX0501	Simple_repeat
(CCACGA)N#Simple_repeat	38	1	X.parietina GTX0501	Simple_repeat
(CCAGCG)N#Simple_repeat	38	1	X.parietina GTX0501	Simple_repeat
(CCATG)N#Simple_repeat	38	1	X.parietina GTX0501	Simple_repeat
(CCGGCG)N#Simple_repeat	38	1	X.parietina GTX0501	Simple_repeat
(CGCC)N#Simple_repeat	38	1	X.parietina GTX0501	Simple_repeat
(CGGCTT)N#Simple_repeat	38	1	X.parietina GTX0501	Simple_repeat
(CTCCATC)N#Simple_repeat	38	1	X.parietina GTX0501	Simple_repeat
(CTTCCGC)N#Simple_repeat	38	1	X.parietina GTX0501	Simple_repeat
(GAAGGC)N#Simple_repeat	38	1	X.parietina GTX0501	Simple_repeat
(GATGTG)N#Simple_repeat	38	1	X.parietina GTX0501	Simple_repeat
(GCGGCG)N#Simple_repeat	38	1	X.parietina GTX0501	Simple_repeat
(GCGTCAA)N#Simple_repeat	38	1	X.parietina GTX0501	Simple_repeat
(GCTGGT)N#Simple_repeat	38	1	X.parietina GTX0501	Simple_repeat
(GGAGG)N#Simple_repeat	38	1	X.parietina GTX0501	Simple_repeat

(GGATGAT)N#Simple_repeat	38	1	X.parietina GTX0501	Simple_repeat
(GGCCTGT)N#Simple_repeat	38	1	X.parietina GTX0501	Simple_repeat
(GGGAGG)N#Simple_repeat	38	1	X.parietina GTX0501	Simple_repeat
(GTCCA)N#Simple_repeat	38	1	X.parietina GTX0501	Simple_repeat
(GTCTATT)N#Simple_repeat	38	1	X.parietina GTX0501	Simple_repeat
(GTGATG)N#Simple_repeat	38	1	X.parietina GTX0501	Simple_repeat
(GTGTGGG)N#Simple_repeat	38	1	X.parietina GTX0501	Simple_repeat
(TACTATA)N#Simple_repeat	38	1	X.parietina GTX0501	Simple_repeat
(TCAGCCC)N#Simple_repeat	38	1	X.parietina GTX0501	Simple_repeat
(TCCAGC)N#Simple_repeat	38	1	X.parietina GTX0501	Simple_repeat
(TCGGCG)N#Simple_repeat	38	1	X.parietina GTX0501	Simple_repeat
(TGAAGG)N#Simple_repeat	38	1	X.parietina GTX0501	Simple_repeat
(TGAGAT)N#Simple_repeat	38	1	X.parietina GTX0501	Simple_repeat
(TGATGG)N#Simple_repeat	38	1	X.parietina GTX0501	Simple_repeat
(TGGCAGA)N#Simple_repeat	38	1	X.parietina GTX0501	Simple_repeat
(TTCCGC)N#Simple_repeat	38	1	X.parietina GTX0501	Simple_repeat
(TTCGCC)N#Simple_repeat	38	1	X.parietina GTX0501	Simple_repeat
(TTCTTCT)N#Simple_repeat	38	1	X.parietina GTX0501	Simple_repeat
(TTGATG)N#Simple_repeat	38	1	X.parietina GTX0501	Simple_repeat
(AAGGCA)N#Simple_repeat	37	1	X.parietina GTX0501	Simple_repeat
(ACCCC)N#Simple_repeat	37	1	X.parietina GTX0501	Simple_repeat
(ACTTCT)N#Simple_repeat	37	1	X.parietina GTX0501	Simple_repeat
(AGAGCA)N#Simple_repeat	37	1	X.parietina GTX0501	Simple_repeat
(AGCAAC)N#Simple_repeat	37	1	X.parietina GTX0501	Simple_repeat
(AGGGGGGA)N#Simple_repeat	37	1	X.parietina GTX0501	Simple_repeat
(ATCTGGC)N#Simple_repeat	37	1	X.parietina GTX0501	Simple_repeat

(ATTGATA)N#Simple_repeat	37	1	X.parietina GTX0501	Simple_repeat
(CACCCG)N#Simple_repeat	37	1	X.parietina GTX0501	Simple_repeat
(CATCATC)N#Simple_repeat	37	1	X.parietina GTX0501	Simple_repeat
(CATCCCG)N#Simple_repeat	37	1	X.parietina GTX0501	Simple_repeat
(CCAAT)N#Simple_repeat	37	1	X.parietina GTX0501	Simple_repeat
(CCACGG)N#Simple_repeat	37	1	X.parietina GTX0501	Simple_repeat
(CCACGGT)N#Simple_repeat	37	1	X.parietina GTX0501	Simple_repeat
(CCACT)N#Simple_repeat	37	1	X.parietina GTX0501	Simple_repeat
(CCATCAT)N#Simple_repeat	37	1	X.parietina GTX0501	Simple_repeat
(CGCCGC)N#Simple_repeat	37	1	X.parietina GTX0501	Simple_repeat
(CGTAGAG)N#Simple_repeat	37	1	X.parietina GTX0501	Simple_repeat
(CGTCGT)N#Simple_repeat	37	1	X.parietina GTX0501	Simple_repeat
(CTACCAA)N#Simple_repeat	37	1	X.parietina GTX0501	Simple_repeat
(CTCTTGT)N#Simple_repeat	37	1	X.parietina GTX0501	Simple_repeat
(CTGATT)N#Simple_repeat	37	1	X.parietina GTX0501	Simple_repeat
(CTTAG)N#Simple_repeat	37	1	X.parietina GTX0501	Simple_repeat
(GACACA)N#Simple_repeat	37	1	X.parietina GTX0501	Simple_repeat
(GAGGGCAAG)N#Simple_repeat	37	1	X.parietina GTX0501	Simple_repeat
(GATGAGC)N#Simple_repeat	37	1	X.parietina GTX0501	Simple_repeat
(GCTCAT)N#Simple_repeat	37	1	X.parietina GTX0501	Simple_repeat
(GCTGCTCCG)N#Simple_repeat	37	1	X.parietina GTX0501	Simple_repeat
(GGACTT)N#Simple_repeat	37	1	X.parietina GTX0501	Simple_repeat
(GGTAAG)N#Simple_repeat	37	1	X.parietina GTX0501	Simple_repeat
(GTCACA)N#Simple_repeat	37	1	X.parietina GTX0501	Simple_repeat
(GTCGTA)N#Simple_repeat	37	1	X.parietina GTX0501	Simple_repeat
(GTGGAGC)N#Simple_repeat	37	1	X.parietina GTX0501	Simple_repeat

(TATCGA)N#Simple_repeat	37	1	X.parietina GTX0501	Simple_repeat
(TCCAACC)N#Simple_repeat	37	1	X.parietina GTX0501	Simple_repeat
(TCCGCT)N#Simple_repeat	37	1	X.parietina GTX0501	Simple_repeat
(TCGTCA)N#Simple_repeat	37	1	X.parietina GTX0501	Simple_repeat
(TGAATAG)N#Simple_repeat	37	1	X.parietina GTX0501	Simple_repeat
(TGCAGTG)N#Simple_repeat	37	1	X.parietina GTX0501	Simple_repeat
(TTCCTTT)N#Simple_repeat	37	1	X.parietina GTX0501	Simple_repeat
(TTTAGT)N#Simple_repeat	37	1	X.parietina GTX0501	Simple_repeat
(AAAGAGA)N#Simple_repeat	36	1	X.parietina GTX0501	Simple_repeat
(AGAAA)N#Simple_repeat	36	1	X.parietina GTX0501	Simple_repeat
(AGAGGAGTA)N#Simple_repeat	36	1	X.parietina GTX0501	Simple_repeat
(AGCA)N#Simple_repeat	36	1	X.parietina GTX0501	Simple_repeat
(AGCATG)N#Simple_repeat	36	1	X.parietina GTX0501	Simple_repeat
(ATAGTA)N#Simple_repeat	36	1	X.parietina GTX0501	Simple_repeat
(CAACCAC)N#Simple_repeat	36	1	X.parietina GTX0501	Simple_repeat
(CACCGC)N#Simple_repeat	36	1	X.parietina GTX0501	Simple_repeat
(CCACTCA)N#Simple_repeat	36	1	X.parietina GTX0501	Simple_repeat
(CGCTCA)N#Simple_repeat	36	1	X.parietina GTX0501	Simple_repeat
(CGCTGC)N#Simple_repeat	36	1	X.parietina GTX0501	Simple_repeat
(CGGTGC)N#Simple_repeat	36	1	X.parietina GTX0501	Simple_repeat
(CTACGA)N#Simple_repeat	36	1	X.parietina GTX0501	Simple_repeat
(CTCCCGA)N#Simple_repeat	36	1	X.parietina GTX0501	Simple_repeat
(CTGCCAG)N#Simple_repeat	36	1	X.parietina GTX0501	Simple_repeat
(CTGGTT)N#Simple_repeat	36	1	X.parietina GTX0501	Simple_repeat
(CTTCCTC)N#Simple_repeat	36	1	X.parietina GTX0501	Simple_repeat
(CTTGTCT)N#Simple_repeat	36	1	X.parietina GTX0501	Simple_repeat

(GAACTC)N#Simple_repeat	36	1	X.parietina GTX0501	Simple_repeat
(GACAAG)N#Simple_repeat	36	1	X.parietina GTX0501	Simple_repeat
(GACCAG)N#Simple_repeat	36	1	X.parietina GTX0501	Simple_repeat
(GAGGCTG)N#Simple_repeat	36	1	X.parietina GTX0501	Simple_repeat
(GAGTGA)N#Simple_repeat	36	1	X.parietina GTX0501	Simple_repeat
(GAGTGAA)N#Simple_repeat	36	1	X.parietina GTX0501	Simple_repeat
(GAGTGC)N#Simple_repeat	36	1	X.parietina GTX0501	Simple_repeat
(GATTGTCAA)N#Simple_repeat	36	1	X.parietina GTX0501	Simple_repeat
(GCAT)N#Simple_repeat	36	1	X.parietina GTX0501	Simple_repeat
(GCCAGTG)N#Simple_repeat	36	1	X.parietina GTX0501	Simple_repeat
(GCTCGAA)N#Simple_repeat	36	1	X.parietina GTX0501	Simple_repeat
(GGCCTT)N#Simple_repeat	36	1	X.parietina GTX0501	Simple_repeat
(GGGGAA)N#Simple_repeat	36	1	X.parietina GTX0501	Simple_repeat
(GTCCT)N#Simple_repeat	36	1	X.parietina GTX0501	Simple_repeat
(GTGATT)N#Simple_repeat	36	1	X.parietina GTX0501	Simple_repeat
(GTGGCG)N#Simple_repeat	36	1	X.parietina GTX0501	Simple_repeat
(TCATC)N#Simple_repeat	36	1	X.parietina GTX0501	Simple_repeat
(TCTGGAT)N#Simple_repeat	36	1	X.parietina GTX0501	Simple_repeat
(TCTTGG)N#Simple_repeat	36	1	X.parietina GTX0501	Simple_repeat
(TGATA)N#Simple_repeat	36	1	X.parietina GTX0501	Simple_repeat
(TGCAGC)N#Simple_repeat	36	1	X.parietina GTX0501	Simple_repeat
(TGGAAGG)N#Simple_repeat	36	1	X.parietina GTX0501	Simple_repeat
(TGGGTGGA)N#Simple_repeat	36	1	X.parietina GTX0501	Simple_repeat
(TGTTGTTGG)N#Simple_repeat	36	1	X.parietina GTX0501	Simple_repeat
(TTGAGC)N#Simple_repeat	36	1	X.parietina GTX0501	Simple_repeat
(AAAAGAA)N#Simple_repeat	35	1	X.parietina GTX0501	Simple_repeat

(AACAAC)N#Simple_repeat	35	1	X.parietina GTX0501	Simple_repeat
(AAGTG)N#Simple_repeat	35	1	X.parietina GTX0501	Simple_repeat
(ACAGCG)N#Simple_repeat	35	1	X.parietina GTX0501	Simple_repeat
(ACTAT)N#Simple_repeat	35	1	X.parietina GTX0501	Simple_repeat
(AGACG)N#Simple_repeat	35	1	X.parietina GTX0501	Simple_repeat
(AGCT)N#Simple_repeat	35	1	X.parietina GTX0501	Simple_repeat
(AGGGGGA)N#Simple_repeat	35	1	X.parietina GTX0501	Simple_repeat
(ATAGGA)N#Simple_repeat	35	1	X.parietina GTX0501	Simple_repeat
(ATCGGA)N#Simple_repeat	35	1	X.parietina GTX0501	Simple_repeat
(ATGAGG)N#Simple_repeat	35	1	X.parietina GTX0501	Simple_repeat
(ATTTA)N#Simple_repeat	35	1	X.parietina GTX0501	Simple_repeat
(CCAATC)N#Simple_repeat	35	1	X.parietina GTX0501	Simple_repeat
(CCATAT)N#Simple_repeat	35	1	X.parietina GTX0501	Simple_repeat
(CCCAATC)N#Simple_repeat	35	1	X.parietina GTX0501	Simple_repeat
(CCGATT)N#Simple_repeat	35	1	X.parietina GTX0501	Simple_repeat
(CCTAGC)N#Simple_repeat	35	1	X.parietina GTX0501	Simple_repeat
(CGTTGG)N#Simple_repeat	35	1	X.parietina GTX0501	Simple_repeat
(CTCA)N#Simple_repeat	35	1	X.parietina GTX0501	Simple_repeat
(CTGCCCG)N#Simple_repeat	35	1	X.parietina GTX0501	Simple_repeat
(CTTGC)N#Simple_repeat	35	1	X.parietina GTX0501	Simple_repeat
(GAAGTGT)N#Simple_repeat	35	1	X.parietina GTX0501	Simple_repeat
(GAATT)N#Simple_repeat	35	1	X.parietina GTX0501	Simple_repeat
(GAGGACG)N#Simple_repeat	35	1	X.parietina GTX0501	Simple_repeat
(GATGGA)N#Simple_repeat	35	1	X.parietina GTX0501	Simple_repeat
(GGAACGA)N#Simple_repeat	35	1	X.parietina GTX0501	Simple_repeat
(GGCGGTT)N#Simple_repeat	35	1	X.parietina GTX0501	Simple_repeat

(GGGATG)N#Simple_repeat	35	1	X.parietina GTX0501	Simple_repeat
(GTTATG)N#Simple_repeat	35	1	X.parietina GTX0501	Simple_repeat
(TATAGTA)N#Simple_repeat	35	1	X.parietina GTX0501	Simple_repeat
(TATGT)N#Simple_repeat	35	1	X.parietina GTX0501	Simple_repeat
(TCACAT)N#Simple_repeat	35	1	X.parietina GTX0501	Simple_repeat
(TCCGCG)N#Simple_repeat	35	1	X.parietina GTX0501	Simple_repeat
(TCCGTC)N#Simple_repeat	35	1	X.parietina GTX0501	Simple_repeat
(TGGTGGA)N#Simple_repeat	35	1	X.parietina GTX0501	Simple_repeat
(TGTCCA)N#Simple_repeat	35	1	X.parietina GTX0501	Simple_repeat
(TGTGT)N#Simple_repeat	35	1	X.parietina GTX0501	Simple_repeat
(TTCATC)N#Simple_repeat	35	1	X.parietina GTX0501	Simple_repeat
(TTGCTG)N#Simple_repeat	35	1	X.parietina GTX0501	Simple_repeat
(TTGGAT)N#Simple_repeat	35	1	X.parietina GTX0501	Simple_repeat
(AACGAT)N#Simple_repeat	34	1	X.parietina GTX0501	Simple_repeat
(ACACAC)N#Simple_repeat	34	1	X.parietina GTX0501	Simple_repeat
(ACGTCT)N#Simple_repeat	34	1	X.parietina GTX0501	Simple_repeat
(AGATAT)N#Simple_repeat	34	1	X.parietina GTX0501	Simple_repeat
(ATCGAGG)N#Simple_repeat	34	1	X.parietina GTX0501	Simple_repeat
(ATGA)N#Simple_repeat	34	1	X.parietina GTX0501	Simple_repeat
(ATGAG)N#Simple_repeat	34	1	X.parietina GTX0501	Simple_repeat
(ATGGC)N#Simple_repeat	34	1	X.parietina GTX0501	Simple_repeat
(ATGTG)N#Simple_repeat	34	1	X.parietina GTX0501	Simple_repeat
(ATTAAG)N#Simple_repeat	34	1	X.parietina GTX0501	Simple_repeat
(ATTAGG)N#Simple_repeat	34	1	X.parietina GTX0501	Simple_repeat
(CCCTTTT)N#Simple_repeat	34	1	X.parietina GTX0501	Simple_repeat
(CCTCTT)N#Simple_repeat	34	1	X.parietina GTX0501	Simple_repeat

(CGAAGG)N#Simple_repeat	34	1	X.parietina GTX0501	Simple_repeat
(CGACAT)N#Simple_repeat	34	1	X.parietina GTX0501	Simple_repeat
(CGGTGG)N#Simple_repeat	34	1	X.parietina GTX0501	Simple_repeat
(CTACA)N#Simple_repeat	34	1	X.parietina GTX0501	Simple_repeat
(CTGTGTT)N#Simple_repeat	34	1	X.parietina GTX0501	Simple_repeat
(GAATCA)N#Simple_repeat	34	1	X.parietina GTX0501	Simple_repeat
(GACTGAT)N#Simple_repeat	34	1	X.parietina GTX0501	Simple_repeat
(GAGCGGA)N#Simple_repeat	34	1	X.parietina GTX0501	Simple_repeat
(GAGGCAG)N#Simple_repeat	34	1	X.parietina GTX0501	Simple_repeat
(GATCA)N#Simple_repeat	34	1	X.parietina GTX0501	Simple_repeat
(GATCAG)N#Simple_repeat	34	1	X.parietina GTX0501	Simple_repeat
(GATGGAG)N#Simple_repeat	34	1	X.parietina GTX0501	Simple_repeat
(GCTGCC)N#Simple_repeat	34	1	X.parietina GTX0501	Simple_repeat
(GCTTTC)N#Simple_repeat	34	1	X.parietina GTX0501	Simple_repeat
(GGAATGG)N#Simple_repeat	34	1	X.parietina GTX0501	Simple_repeat
(GGAGCAG)N#Simple_repeat	34	1	X.parietina GTX0501	Simple_repeat
(GGCGGAG)N#Simple_repeat	34	1	X.parietina GTX0501	Simple_repeat
(GGCGGC)N#Simple_repeat	34	1	X.parietina GTX0501	Simple_repeat
(GTTTGGA)N#Simple_repeat	34	1	X.parietina GTX0501	Simple_repeat
(TCCCG)N#Simple_repeat	34	1	X.parietina GTX0501	Simple_repeat
(TCCTA)N#Simple_repeat	34	1	X.parietina GTX0501	Simple_repeat
(TCGCAC)N#Simple_repeat	34	1	X.parietina GTX0501	Simple_repeat
(TCGCCA)N#Simple_repeat	34	1	X.parietina GTX0501	Simple_repeat
(TCTCACTC)N#Simple_repeat	34	1	X.parietina GTX0501	Simple_repeat
(TGAGAA)N#Simple_repeat	34	1	X.parietina GTX0501	Simple_repeat
(TGATG)N#Simple_repeat	34	1	X.parietina GTX0501	Simple_repeat

(TGGT)N#Simple_repeat	34	1	X.parietina GTX0501	Simple_repeat
(TTAGA)N#Simple_repeat	34	1	X.parietina GTX0501	Simple_repeat
(TTCCTC)N#Simple_repeat	34	1	X.parietina GTX0501	Simple_repeat
(TTGGCG)N#Simple_repeat	34	1	X.parietina GTX0501	Simple_repeat
(TTGGCT)N#Simple_repeat	34	1	X.parietina GTX0501	Simple_repeat
(TTTCCC)N#Simple_repeat	34	1	X.parietina GTX0501	Simple_repeat
(TTTGCCT)N#Simple_repeat	34	1	X.parietina GTX0501	Simple_repeat
(AAAACC)N#Simple_repeat	33	1	X.parietina GTX0501	Simple_repeat
(AATAGA)N#Simple_repeat	33	1	X.parietina GTX0501	Simple_repeat
(AATTGGG)N#Simple_repeat	33	1	X.parietina GTX0501	Simple_repeat
(ACCGTA)N#Simple_repeat	33	1	X.parietina GTX0501	Simple_repeat
(AGCCCCTG)N#Simple_repeat	33	1	X.parietina GTX0501	Simple_repeat
(CACCCT)N#Simple_repeat	33	1	X.parietina GTX0501	Simple_repeat
(CACGTTG)N#Simple_repeat	33	1	X.parietina GTX0501	Simple_repeat
(CACTCCG)N#Simple_repeat	33	1	X.parietina GTX0501	Simple_repeat
(CAGTA)N#Simple_repeat	33	1	X.parietina GTX0501	Simple_repeat
(CAGTTC)N#Simple_repeat	33	1	X.parietina GTX0501	Simple_repeat
(CATGCC)N#Simple_repeat	33	1	X.parietina GTX0501	Simple_repeat
(CATTCC)N#Simple_repeat	33	1	X.parietina GTX0501	Simple_repeat
(CCAGC)N#Simple_repeat	33	1	X.parietina GTX0501	Simple_repeat
(CCCCCG)N#Simple_repeat	33	1	X.parietina GTX0501	Simple_repeat
(CCCCGC)N#Simple_repeat	33	1	X.parietina GTX0501	Simple_repeat
(CCGAAG)N#Simple_repeat	33	1	X.parietina GTX0501	Simple_repeat
(CCGAGT)N#Simple_repeat	33	1	X.parietina GTX0501	Simple_repeat
(CCTCGACC)N#Simple_repeat	33	1	X.parietina GTX0501	Simple_repeat
(CGCATTC)N#Simple_repeat	33	1	X.parietina GTX0501	Simple_repeat

(CGCCAA)N#Simple_repeat	33	1	X.parietina GTX0501	Simple_repeat
(CTCAGT)N#Simple_repeat	33	1	X.parietina GTX0501	Simple_repeat
(GAAGGT)N#Simple_repeat	33	1	X.parietina GTX0501	Simple_repeat
(GACAAC)N#Simple_repeat	33	1	X.parietina GTX0501	Simple_repeat
(GACGCT)N#Simple_repeat	33	1	X.parietina GTX0501	Simple_repeat
(GAGCAC)N#Simple_repeat	33	1	X.parietina GTX0501	Simple_repeat
(GCATTT)N#Simple_repeat	33	1	X.parietina GTX0501	Simple_repeat
(GGAATAT)N#Simple_repeat	33	1	X.parietina GTX0501	Simple_repeat
(GGGAAT)N#Simple_repeat	33	1	X.parietina GTX0501	Simple_repeat
(GGTTCT)N#Simple_repeat	33	1	X.parietina GTX0501	Simple_repeat
(GTCCTTT)N#Simple_repeat	33	1	X.parietina GTX0501	Simple_repeat
(GTGGAA)N#Simple_repeat	33	1	X.parietina GTX0501	Simple_repeat
(GTTGCT)N#Simple_repeat	33	1	X.parietina GTX0501	Simple_repeat
(GTTGG)N#Simple_repeat	33	1	X.parietina GTX0501	Simple_repeat
(TCCACCT)N#Simple_repeat	33	1	X.parietina GTX0501	Simple_repeat
(TCTCCT)N#Simple_repeat	33	1	X.parietina GTX0501	Simple_repeat
(TCTGCT)N#Simple_repeat	33	1	X.parietina GTX0501	Simple_repeat
(TGAG)N#Simple_repeat	33	1	X.parietina GTX0501	Simple_repeat
(TGGTTGA)N#Simple_repeat	33	1	X.parietina GTX0501	Simple_repeat
(TGTCGG)N#Simple_repeat	33	1	X.parietina GTX0501	Simple_repeat
(TTTCAT)N#Simple_repeat	33	1	X.parietina GTX0501	Simple_repeat
(TTTCCTT)N#Simple_repeat	33	1	X.parietina GTX0501	Simple_repeat
(TTTGG)N#Simple_repeat	33	1	X.parietina GTX0501	Simple_repeat
(AAGATG)N#Simple_repeat	32	1	X.parietina GTX0501	Simple_repeat
(AGGGGTC)N#Simple_repeat	32	1	X.parietina GTX0501	Simple_repeat
(AGTGGC)N#Simple_repeat	32	1	X.parietina GTX0501	Simple_repeat

(ATCTATG)N#Simple_repeat	32	1	X.parietina GTX0501	Simple_repeat
(ATTCG)N#Simple_repeat	32	1	X.parietina GTX0501	Simple_repeat
(CAGTGG)N#Simple_repeat	32	1	X.parietina GTX0501	Simple_repeat
(CATCGAC)N#Simple_repeat	32	1	X.parietina GTX0501	Simple_repeat
(CCCATA)N#Simple_repeat	32	1	X.parietina GTX0501	Simple_repeat
(CCGATC)N#Simple_repeat	32	1	X.parietina GTX0501	Simple_repeat
(CGGCAT)N#Simple_repeat	32	1	X.parietina GTX0501	Simple_repeat
(CTATCT)N#Simple_repeat	32	1	X.parietina GTX0501	Simple_repeat
(CTGTG)N#Simple_repeat	32	1	X.parietina GTX0501	Simple_repeat
(GATCCC)N#Simple_repeat	32	1	X.parietina GTX0501	Simple_repeat
(GATGC)N#Simple_repeat	32	1	X.parietina GTX0501	Simple_repeat
(GCGAAG)N#Simple_repeat	32	1	X.parietina GTX0501	Simple_repeat
(GCGGAA)N#Simple_repeat	32	1	X.parietina GTX0501	Simple_repeat
(GCTGTT)N#Simple_repeat	32	1	X.parietina GTX0501	Simple_repeat
(GTATG)N#Simple_repeat	32	1	X.parietina GTX0501	Simple_repeat
(GTGAGA)N#Simple_repeat	32	1	X.parietina GTX0501	Simple_repeat
(GTGT)N#Simple_repeat	32	1	X.parietina GTX0501	Simple_repeat
(TCCGGC)N#Simple_repeat	32	1	X.parietina GTX0501	Simple_repeat
(TCGA)N#Simple_repeat	32	1	X.parietina GTX0501	Simple_repeat
(TGAAGT)N#Simple_repeat	32	1	X.parietina GTX0501	Simple_repeat
(TGAGTT)N#Simple_repeat	32	1	X.parietina GTX0501	Simple_repeat
(TGGCTG)N#Simple_repeat	32	1	X.parietina GTX0501	Simple_repeat
(TTCTGC)N#Simple_repeat	32	1	X.parietina GTX0501	Simple_repeat
(TTTCCT)N#Simple_repeat	32	1	X.parietina GTX0501	Simple_repeat
(TTTCTT)N#Simple_repeat	32	1	X.parietina GTX0501	Simple_repeat
(TTTTTC)N#Simple_repeat	32	1	X.parietina GTX0501	Simple_repeat

(ACAATC)N#Simple_repeat	31	1	X.parietina GTX0501	Simple_repeat
(AGGAA)N#Simple_repeat	31	1	X.parietina GTX0501	Simple_repeat
(AGGATG)N#Simple_repeat	31	1	X.parietina GTX0501	Simple_repeat
(AGGTAG)N#Simple_repeat	31	1	X.parietina GTX0501	Simple_repeat
(ATCTAC)N#Simple_repeat	31	1	X.parietina GTX0501	Simple_repeat
(ATCTT)N#Simple_repeat	31	1	X.parietina GTX0501	Simple_repeat
(CACCTT)N#Simple_repeat	31	1	X.parietina GTX0501	Simple_repeat
(CCATA)N#Simple_repeat	31	1	X.parietina GTX0501	Simple_repeat
(CCCGT)N#Simple_repeat	31	1	X.parietina GTX0501	Simple_repeat
(CCTCGG)N#Simple_repeat	31	1	X.parietina GTX0501	Simple_repeat
(CCTGT)N#Simple_repeat	31	1	X.parietina GTX0501	Simple_repeat
(CCTTCA)N#Simple_repeat	31	1	X.parietina GTX0501	Simple_repeat
(CGAGAT)N#Simple_repeat	31	1	X.parietina GTX0501	Simple_repeat
(CGCCGA)N#Simple_repeat	31	1	X.parietina GTX0501	Simple_repeat
(CGGATT)N#Simple_repeat	31	1	X.parietina GTX0501	Simple_repeat
(CTCCAA)N#Simple_repeat	31	1	X.parietina GTX0501	Simple_repeat
(CTCCTG)N#Simple_repeat	31	1	X.parietina GTX0501	Simple_repeat
(CTCGCT)N#Simple_repeat	31	1	X.parietina GTX0501	Simple_repeat
(GAAGTG)N#Simple_repeat	31	1	X.parietina GTX0501	Simple_repeat
(GACGG)N#Simple_repeat	31	1	X.parietina GTX0501	Simple_repeat
(GAGATT)N#Simple_repeat	31	1	X.parietina GTX0501	Simple_repeat
(GATGCA)N#Simple_repeat	31	1	X.parietina GTX0501	Simple_repeat
(GCACTT)N#Simple_repeat	31	1	X.parietina GTX0501	Simple_repeat
(GGAAGT)N#Simple_repeat	31	1	X.parietina GTX0501	Simple_repeat
(GGGGGT)N#Simple_repeat	31	1	X.parietina GTX0501	Simple_repeat
(GGTAC)N#Simple_repeat	31	1	X.parietina GTX0501	Simple_repeat

(GGTCTC)N#Simple_repeat	31	1	X.parietina GTX0501	Simple_repeat
(GTGTG)N#Simple_repeat	31	1	X.parietina GTX0501	Simple_repeat
(TAGATA)N#Simple_repeat	31	1	X.parietina GTX0501	Simple_repeat
(TCGGAA)N#Simple_repeat	31	1	X.parietina GTX0501	Simple_repeat
(TCTGGTG)N#Simple_repeat	31	1	X.parietina GTX0501	Simple_repeat
(TGACCT)N#Simple_repeat	31	1	X.parietina GTX0501	Simple_repeat
(TGGGTG)N#Simple_repeat	31	1	X.parietina GTX0501	Simple_repeat
(TTCAA)N#Simple_repeat	31	1	X.parietina GTX0501	Simple_repeat
(TTGGTCA)N#Simple_repeat	31	1	X.parietina GTX0501	Simple_repeat
(TTTTTTC)N#Simple_repeat	31	1	X.parietina GTX0501	Simple_repeat
(AACCA)N#Simple_repeat	30	1	X.parietina GTX0501	Simple_repeat
(AATCCC)N#Simple_repeat	30	1	X.parietina GTX0501	Simple_repeat
(AATTG)N#Simple_repeat	30	1	X.parietina GTX0501	Simple_repeat
(ACACAA)N#Simple_repeat	30	1	X.parietina GTX0501	Simple_repeat
(ACCAT)N#Simple_repeat	30	1	X.parietina GTX0501	Simple_repeat
(ACGACG)N#Simple_repeat	30	1	X.parietina GTX0501	Simple_repeat
(ATCCTT)N#Simple_repeat	30	1	X.parietina GTX0501	Simple_repeat
(ATGGGG)N#Simple_repeat	30	1	X.parietina GTX0501	Simple_repeat
(ATTGA)N#Simple_repeat	30	1	X.parietina GTX0501	Simple_repeat
(CATATT)N#Simple_repeat	30	1	X.parietina GTX0501	Simple_repeat
(CCCCG)N#Simple_repeat	30	1	X.parietina GTX0501	Simple_repeat
(CCCTAA)N#Simple_repeat	30	1	X.parietina GTX0501	Simple_repeat
(CCGCAG)N#Simple_repeat	30	1	X.parietina GTX0501	Simple_repeat
(CGAAG)N#Simple_repeat	30	1	X.parietina GTX0501	Simple_repeat
(CGAGG)N#Simple_repeat	30	1	X.parietina GTX0501	Simple_repeat
(CGATGG)N#Simple_repeat	30	1	X.parietina GTX0501	Simple_repeat

(CGTAA)N#Simple_repeat	30	1	X.parietina GTX0501	Simple_repeat
(CTCGC)N#Simple_repeat	30	1	X.parietina GTX0501	Simple_repeat
(CTCGTT)N#Simple_repeat	30	1	X.parietina GTX0501	Simple_repeat
(CTTGCA)N#Simple_repeat	30	1	X.parietina GTX0501	Simple_repeat
(GAAGA)N#Simple_repeat	30	1	X.parietina GTX0501	Simple_repeat
(GAGAGAC)N#Simple_repeat	30	1	X.parietina GTX0501	Simple_repeat
(GATGTT)N#Simple_repeat	30	1	X.parietina GTX0501	Simple_repeat
(GCTGA)N#Simple_repeat	30	1	X.parietina GTX0501	Simple_repeat
(GGAGAG)N#Simple_repeat	30	1	X.parietina GTX0501	Simple_repeat
(GGAGGGT)N#Simple_repeat	30	1	X.parietina GTX0501	Simple_repeat
(GGCTTC)N#Simple_repeat	30	1	X.parietina GTX0501	Simple_repeat
(GGCTTT)N#Simple_repeat	30	1	X.parietina GTX0501	Simple_repeat
(GGGTAT)N#Simple_repeat	30	1	X.parietina GTX0501	Simple_repeat
(GTCCTC)N#Simple_repeat	30	1	X.parietina GTX0501	Simple_repeat
(TATAA)N#Simple_repeat	30	1	X.parietina GTX0501	Simple_repeat
(TCCACG)N#Simple_repeat	30	1	X.parietina GTX0501	Simple_repeat
(TGAAGGT)N#Simple_repeat	30	1	X.parietina GTX0501	Simple_repeat
(TTTGTG)N#Simple_repeat	30	1	X.parietina GTX0501	Simple_repeat
(AAGGTA)N#Simple_repeat	29	1	X.parietina GTX0501	Simple_repeat
(AATCC)N#Simple_repeat	29	1	X.parietina GTX0501	Simple_repeat
(ACCCCA)N#Simple_repeat	29	1	X.parietina GTX0501	Simple_repeat
(AGAGGC)N#Simple_repeat	29	1	X.parietina GTX0501	Simple_repeat
(AGCAA)N#Simple_repeat	29	1	X.parietina GTX0501	Simple_repeat
(AGGGAAG)N#Simple_repeat	29	1	X.parietina GTX0501	Simple_repeat
(ATATA)N#Simple_repeat	29	1	X.parietina GTX0501	Simple_repeat
(ATGTAC)N#Simple_repeat	29	1	X.parietina GTX0501	Simple_repeat

(ATTA)N#Simple_repeat	29	1	X.parietina GTX0501	Simple_repeat
(CACA)N#Simple_repeat	29	1	X.parietina GTX0501	Simple_repeat
(CCCTA)N#Simple_repeat	29	1	X.parietina GTX0501	Simple_repeat
(CGCTGA)N#Simple_repeat	29	1	X.parietina GTX0501	Simple_repeat
(CTCGAC)N#Simple_repeat	29	1	X.parietina GTX0501	Simple_repeat
(CTGAACC)N#Simple_repeat	29	1	X.parietina GTX0501	Simple_repeat
(GAGCA)N#Simple_repeat	29	1	X.parietina GTX0501	Simple_repeat
(GAGGA)N#Simple_repeat	29	1	X.parietina GTX0501	Simple_repeat
(GATAAA)N#Simple_repeat	29	1	X.parietina GTX0501	Simple_repeat
(GCAGA)N#Simple_repeat	29	1	X.parietina GTX0501	Simple_repeat
(GCGACG)N#Simple_repeat	29	1	X.parietina GTX0501	Simple_repeat
(GCGGA)N#Simple_repeat	29	1	X.parietina GTX0501	Simple_repeat
(GCTGCA)N#Simple_repeat	29	1	X.parietina GTX0501	Simple_repeat
(GGACGA)N#Simple_repeat	29	1	X.parietina GTX0501	Simple_repeat
(GGCGTG)N#Simple_repeat	29	1	X.parietina GTX0501	Simple_repeat
(GGCTT)N#Simple_repeat	29	1	X.parietina GTX0501	Simple_repeat
(GGGGT)N#Simple_repeat	29	1	X.parietina GTX0501	Simple_repeat
(GTGGGTG)N#Simple_repeat	29	1	X.parietina GTX0501	Simple_repeat
(TAATT)N#Simple_repeat	29	1	X.parietina GTX0501	Simple_repeat
(TGAGGGG)N#Simple_repeat	29	1	X.parietina GTX0501	Simple_repeat
(TGGTCG)N#Simple_repeat	29	1	X.parietina GTX0501	Simple_repeat
(TGTCA)N#Simple_repeat	29	1	X.parietina GTX0501	Simple_repeat
(TGTTTT)N#Simple_repeat	29	1	X.parietina GTX0501	Simple_repeat
(TTTATC)N#Simple_repeat	29	1	X.parietina GTX0501	Simple_repeat
(TTTGT)N#Simple_repeat	29	1	X.parietina GTX0501	Simple_repeat
(AAAAG)N#Simple_repeat	28	1	X.parietina GTX0501	Simple_repeat

(AGGTCA)N#Simple_repeat	28	1	X.parietina GTX0501	Simple_repeat
(AGTATA)N#Simple_repeat	28	1	X.parietina GTX0501	Simple_repeat
(CAAGCTC)N#Simple_repeat	28	1	X.parietina GTX0501	Simple_repeat
(CCCG)N#Simple_repeat	28	1	X.parietina GTX0501	Simple_repeat
(CTGAT)N#Simple_repeat	28	1	X.parietina GTX0501	Simple_repeat
(GAGCAA)N#Simple_repeat	28	1	X.parietina GTX0501	Simple_repeat
(GCCCA)N#Simple_repeat	28	1	X.parietina GTX0501	Simple_repeat
(GGACG)N#Simple_repeat	28	1	X.parietina GTX0501	Simple_repeat
(GGAGAT)N#Simple_repeat	28	1	X.parietina GTX0501	Simple_repeat
(GGGAG)N#Simple_repeat	28	1	X.parietina GTX0501	Simple_repeat
(GGTTA)N#Simple_repeat	28	1	X.parietina GTX0501	Simple_repeat
(GTGTGC)N#Simple_repeat	28	1	X.parietina GTX0501	Simple_repeat
(GTTA)N#Simple_repeat	28	1	X.parietina GTX0501	Simple_repeat
(TATCA)N#Simple_repeat	28	1	X.parietina GTX0501	Simple_repeat
(TCATT)N#Simple_repeat	28	1	X.parietina GTX0501	Simple_repeat
(TGTCT)N#Simple_repeat	28	1	X.parietina GTX0501	Simple_repeat
(TTATA)N#Simple_repeat	28	1	X.parietina GTX0501	Simple_repeat
(TTGGA)N#Simple_repeat	28	1	X.parietina GTX0501	Simple_repeat
(TTGT)N#Simple_repeat	28	1	X.parietina GTX0501	Simple_repeat
(TTTTG)N#Simple_repeat	28	1	X.parietina GTX0501	Simple_repeat
(AAACC)N#Simple_repeat	27	1	X.parietina GTX0501	Simple_repeat
(ACCCA)N#Simple_repeat	27	1	X.parietina GTX0501	Simple_repeat
(ACCG)N#Simple_repeat	27	1	X.parietina GTX0501	Simple_repeat
(AGGCA)N#Simple_repeat	27	1	X.parietina GTX0501	Simple_repeat
(AGTG)N#Simple_repeat	27	1	X.parietina GTX0501	Simple_repeat
(ATGGT)N#Simple_repeat	27	1	X.parietina GTX0501	Simple_repeat

(CAAG)N#Simple_repeat	27	1	X.parietina GTX0501	Simple_repeat
(CCACC)N#Simple_repeat	27	1	X.parietina GTX0501	Simple_repeat
(CCACCCC)N#Simple_repeat	27	1	X.parietina GTX0501	Simple_repeat
(CTACT)N#Simple_repeat	27	1	X.parietina GTX0501	Simple_repeat
(CTCCG)N#Simple_repeat	27	1	X.parietina GTX0501	Simple_repeat
(CTCTG)N#Simple_repeat	27	1	X.parietina GTX0501	Simple_repeat
(CTTGG)N#Simple_repeat	27	1	X.parietina GTX0501	Simple_repeat
(GCTGGG)N#Simple_repeat	27	1	X.parietina GTX0501	Simple_repeat
(TCCTT)N#Simple_repeat	27	1	X.parietina GTX0501	Simple_repeat
(TGAAT)N#Simple_repeat	27	1	X.parietina GTX0501	Simple_repeat
(TGCCG)N#Simple_repeat	27	1	X.parietina GTX0501	Simple_repeat
(TGGGT)N#Simple_repeat	27	1	X.parietina GTX0501	Simple_repeat
(TTGAT)N#Simple_repeat	27	1	X.parietina GTX0501	Simple_repeat
(TTGCT)N#Simple_repeat	27	1	X.parietina GTX0501	Simple_repeat
(TTGTA)N#Simple_repeat	27	1	X.parietina GTX0501	Simple_repeat
(AACG)N#Simple_repeat	26	1	X.parietina GTX0501	Simple_repeat
(AAGAA)N#Simple_repeat	26	1	X.parietina GTX0501	Simple_repeat
(ACTGT)N#Simple_repeat	26	1	X.parietina GTX0501	Simple_repeat
(AGAGG)N#Simple_repeat	26	1	X.parietina GTX0501	Simple_repeat
(AGGA)N#Simple_repeat	26	1	X.parietina GTX0501	Simple_repeat
(CAAA)N#Simple_repeat	26	1	X.parietina GTX0501	Simple_repeat
(CAACA)N#Simple_repeat	26	1	X.parietina GTX0501	Simple_repeat
(CGATGA)N#Simple_repeat	26	1	X.parietina GTX0501	Simple_repeat
(CTGCT)N#Simple_repeat	26	1	X.parietina GTX0501	Simple_repeat
(CTTGTT)N#Simple_repeat	26	1	X.parietina GTX0501	Simple_repeat
(GAATGC)N#Simple_repeat	26	1	X.parietina GTX0501	Simple_repeat

(GACTG)N#Simple_repeat	26	1	X.parietina GTX0501	Simple_repeat
(GAGAA)N#Simple_repeat	26	1	X.parietina GTX0501	Simple_repeat
(GAGAGC)N#Simple_repeat	26	1	X.parietina GTX0501	Simple_repeat
(GCTAT)N#Simple_repeat	26	1	X.parietina GTX0501	Simple_repeat
(GGAAG)N#Simple_repeat	26	1	X.parietina GTX0501	Simple_repeat
(GGATT)N#Simple_repeat	26	1	X.parietina GTX0501	Simple_repeat
(GGGC)N#Simple_repeat	26	1	X.parietina GTX0501	Simple_repeat
(GTTTG)N#Simple_repeat	26	1	X.parietina GTX0501	Simple_repeat
(TGAGG)N#Simple_repeat	26	1	X.parietina GTX0501	Simple_repeat
(TGGCA)N#Simple_repeat	26	1	X.parietina GTX0501	Simple_repeat
(TTCAT)N#Simple_repeat	26	1	X.parietina GTX0501	Simple_repeat
(ACGAAA)N#Simple_repeat	25	1	X.parietina GTX0501	Simple_repeat
(CCTG)N#Simple_repeat	25	1	X.parietina GTX0501	Simple_repeat
(CGCAGT)N#Simple_repeat	25	1	X.parietina GTX0501	Simple_repeat
(CGTAT)N#Simple_repeat	25	1	X.parietina GTX0501	Simple_repeat
(CGTCT)N#Simple_repeat	25	1	X.parietina GTX0501	Simple_repeat
(CTTTC)N#Simple_repeat	25	1	X.parietina GTX0501	Simple_repeat
(GAAATG)N#Simple_repeat	25	1	X.parietina GTX0501	Simple_repeat
(GAAGG)N#Simple_repeat	25	1	X.parietina GTX0501	Simple_repeat
(GACAGC)N#Simple_repeat	25	1	X.parietina GTX0501	Simple_repeat
(GAGTT)N#Simple_repeat	25	1	X.parietina GTX0501	Simple_repeat
(GCCAA)N#Simple_repeat	25	1	X.parietina GTX0501	Simple_repeat
(GGCT)N#Simple_repeat	25	1	X.parietina GTX0501	Simple_repeat
(GTGCTT)N#Simple_repeat	25	1	X.parietina GTX0501	Simple_repeat
(TCCAA)N#Simple_repeat	25	1	X.parietina GTX0501	Simple_repeat
(TGCG)N#Simple_repeat	25	1	X.parietina GTX0501	Simple_repeat

(TGCTT)N#Simple_repeat	25	1	X.parietina GTX0501	Simple_repeat
(TGGAC)N#Simple_repeat	25	1	X.parietina GTX0501	Simple_repeat
(TTATT)N#Simple_repeat	25	1	X.parietina GTX0501	Simple_repeat
(TTCGC)N#Simple_repeat	25	1	X.parietina GTX0501	Simple_repeat
(TTTG)N#Simple_repeat	25	1	X.parietina GTX0501	Simple_repeat
(AGGC)N#Simple_repeat	24	1	X.parietina GTX0501	Simple_repeat
(AGTGGT)N#Simple_repeat	24	1	X.parietina GTX0501	Simple_repeat
(CCACA)N#Simple_repeat	24	1	X.parietina GTX0501	Simple_repeat
(CCCATT)N#Simple_repeat	24	1	X.parietina GTX0501	Simple_repeat
(CTGGAA)N#Simple_repeat	24	1	X.parietina GTX0501	Simple_repeat
(GACT)N#Simple_repeat	24	1	X.parietina GTX0501	Simple_repeat
(GAGAGG)N#Simple_repeat	24	1	X.parietina GTX0501	Simple_repeat
(GAGCCT)N#Simple_repeat	24	1	X.parietina GTX0501	Simple_repeat
(GAGTG)N#Simple_repeat	24	1	X.parietina GTX0501	Simple_repeat
(GCAAGA)N#Simple_repeat	24	1	X.parietina GTX0501	Simple_repeat
(GGGAT)N#Simple_repeat	24	1	X.parietina GTX0501	Simple_repeat
(TGGGAG)N#Simple_repeat	24	1	X.parietina GTX0501	Simple_repeat
(TTCA)N#Simple_repeat	24	1	X.parietina GTX0501	Simple_repeat
(TTGTTA)N#Simple_repeat	24	1	X.parietina GTX0501	Simple_repeat
(AAGGG)N#Simple_repeat	23	1	X.parietina GTX0501	Simple_repeat
(CGCA)N#Simple_repeat	23	1	X.parietina GTX0501	Simple_repeat
(CGTA)N#Simple_repeat	23	1	X.parietina GTX0501	Simple_repeat
(CTATA)N#Simple_repeat	23	1	X.parietina GTX0501	Simple_repeat
(GATC)N#Simple_repeat	23	1	X.parietina GTX0501	Simple_repeat
(TCAA)N#Simple_repeat	23	1	X.parietina GTX0501	Simple_repeat
(TCAT)N#Simple_repeat	23	1	X.parietina GTX0501	Simple_repeat

(ACAA)N#Simple_repeat	22	1	X.parietina GTX0501	Simple_repeat
(ATAC)N#Simple_repeat	22	1	X.parietina GTX0501	Simple_repeat
(TAG)N#Simple_repeat	22	1	X.parietina GTX0501	Simple_repeat
(AACA)N#Simple_repeat	21	1	X.parietina GTX0501	Simple_repeat
(CATA)N#Simple_repeat	21	1	X.parietina GTX0501	Simple_repeat
(CCGG)N#Simple_repeat	21	1	X.parietina GTX0501	Simple_repeat
(GCCT)N#Simple_repeat	21	1	X.parietina GTX0501	Simple_repeat
(GGGT)N#Simple_repeat	21	1	X.parietina GTX0501	Simple_repeat
(TAGC)N#Simple_repeat	21	1	X.parietina GTX0501	Simple_repeat
(CCCTCTC)N#Simple_repeat	20	1	X.parietina GTX0501	Simple_repeat
(CGTG)N#Simple_repeat	20	1	X.parietina GTX0501	Simple_repeat
(GCTC)N#Simple_repeat	20	1	X.parietina GTX0501	Simple_repeat
(TGTT)N#Simple_repeat	20	1	X.parietina GTX0501	Simple_repeat
(TTCTCG)N#Simple_repeat	20	1	X.parietina GTX0501	Simple_repeat
(CGCTCT)N#Simple_repeat	17	1	X.parietina GTX0501	Simple_repeat
(GTATGT)N#Simple_repeat	16	1	X.parietina GTX0501	Simple_repeat
(GTTTTT)N#Simple_repeat	13	1	X.parietina GTX0501	Simple_repeat
(CCCCACC)N#Simple_repeat	11	1	X.parietina GTX0501	Simple_repeat
(TATT)N#Simple_repeat	11	1	X.parietina GTX0501	Simple_repeat
(TTTGCG)N#Simple_repeat	11	1	X.parietina GTX0501	Simple_repeat
(GAGTGG)N#Simple_repeat	10	1	X.parietina GTX0501	Simple_repeat
(ACCACCC)N#Simple_repeat	8	1	X.parietina GTX0501	Simple_repeat
(CAAGGA)N#Simple_repeat	8	1	X.parietina GTX0501	Simple_repeat
(GCCGCC)N#Simple_repeat	7	1	X.parietina GTX0501	Simple_repeat
(TCATCC)N#Simple_repeat	7	1	X.parietina GTX0501	Simple_repeat
(CAGCAC)N#Simple_repeat	6	1	X.parietina GTX0501	Simple_repeat

(TGGATTG)N#Simple_repeat	6	1	X.parietina GTX0501	Simple_repeat
(GCCACC)N#Simple_repeat	5	1	X.parietina GTX0501	Simple_repeat
(GGATTTC)N#Simple_repeat	5	1	X.parietina GTX0501	Simple_repeat
(GTGGTC)N#Simple_repeat	5	1	X.parietina GTX0501	Simple_repeat
RND-1_FAMILY-92#LTR/Ty3	414488	139	X.calcicola GTX0491	LTR/Ty3
RND-1_FAMILY-123#LTR/Ty3	396033	89	X.calcicola GTX0491	LTR/Ty3
RND-1_FAMILY-155#LTR/Ty3	376560	111	X.calcicola GTX0491	LTR/Ty3
RND-1_FAMILY-147#LTR/Ty3	375653	142	X.calcicola GTX0491	LTR/Ty3
RND-2_FAMILY-17#LTR/Ty3	341820	114	X.calcicola GTX0491	LTR/Ty3
RND-1_FAMILY-75#RC/Helitron	323113	172	X.calcicola GTX0491	RC/Helitron
RND-2_FAMILY-199#LTR/Ty3	305624	92	X.calcicola GTX0491	LTR/Ty3
RND-1_FAMILY-84#Unknown	288238	51	X.calcicola GTX0491	Unknown
RND-1_FAMILY-131#LTR/Ty3	254228	107	X.calcicola GTX0491	LTR/Ty3
RND-1_FAMILY-104#LTR/Ty3	238945	55	X.calcicola GTX0491	LTR/Ty3
RND-1_FAMILY-54#LTR/Ty3	230769	82	X.calcicola GTX0491	LTR/Ty3
RND-1_FAMILY-87#LTR/Ty3	210597	107	X.calcicola GTX0491	LTR/Ty3
RND-1_FAMILY-22#Unknown	194506	48	X.calcicola GTX0491	Unknown
RND-2_FAMILY-156#LTR/Ty3	190281	46	X.calcicola GTX0491	LTR/Ty3
RND-1_FAMILY-203#LTR/Ty3	174775	86	X.calcicola GTX0491	LTR/Ty3
RND-1_FAMILY-103#LTR/Ty3	169497	112	X.calcicola GTX0491	LTR/Ty3
RND-1_FAMILY-109#LTR/Copia	162363	51	X.calcicola GTX0491	LTR/Copia
RND-1_FAMILY-164#LTR/Ty3	138714	26	X.calcicola GTX0491	LTR/Ty3
RND-1_FAMILY-163#Unknown	130376	46	X.calcicola GTX0491	Unknown
RND-1_FAMILY-154#Unknown	118145	162	X.calcicola GTX0491	Unknown
RND-2_FAMILY-15#LTR/Copia	104305	48	X.calcicola GTX0491	LTR/Copia
RND-1_FAMILY-198#LTR/Ty3	95608	65	X.calcicola GTX0491	LTR/Ty3

RND-3_FAMILY-138#LTR/Copia	91614	38	X.calcicola GTX0491	LTR/Copia
RND-1_FAMILY-76#LTR/Ty3	91152	39	X.calcicola GTX0491	LTR/Ty3
RND-1_FAMILY-124#Unknown	90675	96	X.calcicola GTX0491	Unknown
RND-1_FAMILY-1#LTR/Ty3	89377	56	X.calcicola GTX0491	LTR/Ty3
RND-3_FAMILY-883#Unknown	85202	42	X.calcicola GTX0491	Unknown
RND-1_FAMILY-200#LTR/Ty3	81021	31	X.calcicola GTX0491	LTR/Ty3
RND-1_FAMILY-145#RC/Helitron	80282	43	X.calcicola GTX0491	RC/Helitron
RND-1_FAMILY-176#Unknown	80010	37	X.calcicola GTX0491	Unknown
RND-1_FAMILY-79#Unknown	76183	38	X.calcicola GTX0491	Unknown
RND-1_FAMILY-153#RC/Helitron	68951	36	X.calcicola GTX0491	RC/Helitron
RND-1_FAMILY-95#Unknown	64237	58	X.calcicola GTX0491	Unknown
RND-1_FAMILY-184#Unknown	58371	120	X.calcicola GTX0491	Unknown
RND-1_FAMILY-48#LTR/Ty3	58163	17	X.calcicola GTX0491	LTR/Ty3
RND-1_FAMILY-144#Unknown	57681	70	X.calcicola GTX0491	Unknown
RND-1_FAMILY-168#LTR/Ty3	57600	22	X.calcicola GTX0491	LTR/Ty3
RND-1_FAMILY-39#LTR/Ty3	56409	21	X.calcicola GTX0491	LTR/Ty3
RND-1_FAMILY-174#Unknown	54176	133	X.calcicola GTX0491	Unknown
RND-1_FAMILY-5#LTR/Ty3	54065	58	X.calcicola GTX0491	LTR/Ty3
RND-1_FAMILY-150#Unknown	53670	88	X.calcicola GTX0491	Unknown
RND-1_FAMILY-16#LTR/Ty3	49129	19	X.calcicola GTX0491	LTR/Ty3
RND-1_FAMILY-166#LTR/Copia	48893	49	X.calcicola GTX0491	LTR/Copia
RND-1_FAMILY-135#LTR/Ty3	47131	27	X.calcicola GTX0491	LTR/Ty3
RND-1_FAMILY-188#Unknown	45620	18	X.calcicola GTX0491	Unknown
RND-1_FAMILY-186#RC/Helitron	45146	27	X.calcicola GTX0491	RC/Helitron
RND-1_FAMILY-119#Unknown	43409	35	X.calcicola GTX0491	Unknown
RND-1_FAMILY-170#Unknown	41978	84	X.calcicola GTX0491	Unknown

RND-1_FAMILY-29#Unknown	41744	79	X.calcicola GTX0491	Unknown
RND-1_FAMILY-59#RC/Helitron	41535	46	X.calcicola GTX0491	RC/Helitron
RND-1_FAMILY-190#LTR/Ty3	40398	22	X.calcicola GTX0491	LTR/Ty3
RND-1_FAMILY-11#Unknown	39756	74	X.calcicola GTX0491	Unknown
RND-1_FAMILY-126#Unknown	37825	30	X.calcicola GTX0491	Unknown
RND-3_FAMILY-73#Unknown	36024	13	X.calcicola GTX0491	Unknown
RND-1_FAMILY-32#LTR/Ty3	35223	18	X.calcicola GTX0491	LTR/Ty3
RND-1_FAMILY-94#Unknown	32665	14	X.calcicola GTX0491	Unknown
RND-1_FAMILY-49#DNA/TcMar-Ant1	31168	23	X.calcicola GTX0491	DNA/TcMar-Ant1
RND-1_FAMILY-10#LTR/Ty3	29752	14	X.calcicola GTX0491	LTR/Ty3
RND-1_FAMILY-93#DNA/MULE-MuDR	28917	37	X.calcicola GTX0491	DNA/MULE-MuDR
RND-1_FAMILY-55#Unknown	28637	18	X.calcicola GTX0491	Unknown
RND-1_FAMILY-20#LTR/Ty3	26902	10	X.calcicola GTX0491	LTR/Ty3
RND-1_FAMILY-112#LTR/Ty3	26073	14	X.calcicola GTX0491	LTR/Ty3
RND-1_FAMILY-4#LTR/Ty3	25865	15	X.calcicola GTX0491	LTR/Ty3
RND-1_FAMILY-3#Unknown	25741	58	X.calcicola GTX0491	Unknown
RND-3_FAMILY-99#Unknown	24373	42	X.calcicola GTX0491	Unknown
RND-3_FAMILY-424#Unknown	23884	35	X.calcicola GTX0491	Unknown
RND-1_FAMILY-106#DNA/TcMar-Fot1	22710	25	X.calcicola GTX0491	DNA/TcMar-Fot1
RND-1_FAMILY-115#Unknown	22622	21	X.calcicola GTX0491	Unknown
RND-1_FAMILY-34#LTR/Copia	22516	12	X.calcicola GTX0491	LTR/Copia
RND-1_FAMILY-46#LTR/Ty3	22308	17	X.calcicola GTX0491	LTR/Ty3
RND-1_FAMILY-195#Unknown	21665	68	X.calcicola GTX0491	Unknown
RND-1_FAMILY-160#LTR/Ty3	21446	24	X.calcicola GTX0491	LTR/Ty3
RND-1_FAMILY-132#LTR/Ty3	21362	24	X.calcicola GTX0491	LTR/Ty3

RND-1_FAMILY-185#LTR/Ty3	21169	13	X.calcicola GTX0491	LTR/Ty3
RND-1_FAMILY-146#Unknown	20213	13	X.calcicola GTX0491	Unknown
RND-1_FAMILY-172#LTR/Ty3	19891	12	X.calcicola GTX0491	LTR/Ty3
RND-1_FAMILY-105#DNA/TcMar-Tc1	19625	36	X.calcicola GTX0491	DNA/TcMar-Tc1
RND-1_FAMILY-53#DNA/TcMar-Fot1	19439	23	X.calcicola GTX0491	DNA/TcMar-Fot1
RND-1_FAMILY-100#LTR/Ty3	17903	16	X.calcicola GTX0491	LTR/Ty3
RND-1_FAMILY-165#Unknown	16585	28	X.calcicola GTX0491	Unknown
RND-1_FAMILY-83#LTR/Ty3	16258	9	X.calcicola GTX0491	LTR/Ty3
G-RICH#Low_complexity	15998	279	X.calcicola GTX0491	Low_complexity
RND-1_FAMILY-60#LTR/Ty3	15660	7	X.calcicola GTX0491	LTR/Ty3
RND-1_FAMILY-141#Unknown	15614	34	X.calcicola GTX0491	Unknown
RND-1_FAMILY-161#LTR/Ty3	15523	12	X.calcicola GTX0491	LTR/Ty3
GA-RICH#Low_complexity	15222	282	X.calcicola GTX0491	Low_complexity
RND-1_FAMILY-118#Unknown	14961	15	X.calcicola GTX0491	Unknown
RND-1_FAMILY-138#LTR/Ty3	14760	9	X.calcicola GTX0491	LTR/Ty3
RND-3_FAMILY-208#Unknown	14661	99	X.calcicola GTX0491	Unknown
RND-1_FAMILY-97#Unknown	14623	25	X.calcicola GTX0491	Unknown
RND-1_FAMILY-121#SINE/B2	14564	17	X.calcicola GTX0491	SINE/B2
RND-1_FAMILY-65#Unknown	14301	36	X.calcicola GTX0491	Unknown
RND-1_FAMILY-201#LTR/Copia	13990	16	X.calcicola GTX0491	LTR/Copia
RND-1_FAMILY-158#Unknown	13824	27	X.calcicola GTX0491	Unknown
RND-1_FAMILY-61#Unknown	13340	10	X.calcicola GTX0491	Unknown
RND-1_FAMILY-33#Unknown	13284	24	X.calcicola GTX0491	Unknown
RND-1_FAMILY-196#Unknown	12986	7	X.calcicola GTX0491	Unknown
RND-1_FAMILY-63#LTR/Ty3	12802	10	X.calcicola GTX0491	LTR/Ty3
RND-1_FAMILY-142#Unknown	12766	85	X.calcicola GTX0491	Unknown

RND-3_FAMILY-80#Unknown	12720	95	X.calcicola GTX0491	Unknown
RND-1_FAMILY-197#Unknown	12355	12	X.calcicola GTX0491	Unknown
RND-1_FAMILY-67#Unknown	12339	22	X.calcicola GTX0491	Unknown
RND-3_FAMILY-646#RC/Helitron	12028	18	X.calcicola GTX0491	RC/Helitron
RND-1_FAMILY-13#Unknown	11818	8	X.calcicola GTX0491	Unknown
RND-1_FAMILY-143#DNA/TcMar-Fot1	11598	9	X.calcicola GTX0491	DNA/TcMar-Fot1
RND-1_FAMILY-151#Unknown	11129	74	X.calcicola GTX0491	Unknown
RND-1_FAMILY-128#Unknown	10909	16	X.calcicola GTX0491	Unknown
RND-1_FAMILY-2#Unknown	10825	59	X.calcicola GTX0491	Unknown
A-RICH#Low_complexity	10798	217	X.calcicola GTX0491	Low_complexity
RND-1_FAMILY-24#DNA/TcMar-Tc1	10597	7	X.calcicola GTX0491	DNA/TcMar-Tc1
RND-1_FAMILY-167#Unknown	10427	15	X.calcicola GTX0491	Unknown
RND-1_FAMILY-50#Unknown	10427	43	X.calcicola GTX0491	Unknown
RND-1_FAMILY-38#DNA/TcMar-Ant1	10234	8	X.calcicola GTX0491	DNA/TcMar-Ant1
RND-1_FAMILY-149#DNA/MULE-MuDR	9719	20	X.calcicola GTX0491	DNA/MULE-MuDR
RND-1_FAMILY-139#Unknown	9639	63	X.calcicola GTX0491	Unknown
RND-1_FAMILY-8#Unknown	9486	16	X.calcicola GTX0491	Unknown
RND-1_FAMILY-90#Unknown	9304	19	X.calcicola GTX0491	Unknown
RND-1_FAMILY-12#Unknown	9184	25	X.calcicola GTX0491	Unknown
RND-1_FAMILY-159#Unknown	9073	30	X.calcicola GTX0491	Unknown
RND-3_FAMILY-79#Unknown	8849	86	X.calcicola GTX0491	Unknown
RND-1_FAMILY-66#Unknown	8799	18	X.calcicola GTX0491	Unknown
RND-1_FAMILY-205#Unknown	8721	16	X.calcicola GTX0491	Unknown
RND-1_FAMILY-86#Unknown	8643	50	X.calcicola GTX0491	Unknown
RND-1_FAMILY-91#Unknown	8449	22	X.calcicola GTX0491	Unknown

RND-1_FAMILY-156#Unknown	8180	20	X.calcicola GTX0491	Unknown
RND-1_FAMILY-31#Unknown	7962	39	X.calcicola GTX0491	Unknown
RND-1_FAMILY-74#RC/Helitron	7913	1	X.calcicola GTX0491	RC/Helitron
RND-1_FAMILY-194#Unknown	7683	16	X.calcicola GTX0491	Unknown
RND-1_FAMILY-17#Unknown	7677	13	X.calcicola GTX0491	Unknown
RND-1_FAMILY-71#Unknown	7358	7	X.calcicola GTX0491	Unknown
RND-1_FAMILY-19#Unknown	7314	14	X.calcicola GTX0491	Unknown
RND-1_FAMILY-98#DNA/MULE-MuDR	7314	19	X.calcicola GTX0491	DNA/MULE-MuDR
RND-1_FAMILY-15#LTR/Ty3	7131	5	X.calcicola GTX0491	LTR/Ty3
RND-3_FAMILY-77#Unknown	7073	50	X.calcicola GTX0491	Unknown
RND-1_FAMILY-175#LTR/Ty3	6974	17	X.calcicola GTX0491	LTR/Ty3
RND-1_FAMILY-56#LTR/Copia	6892	7	X.calcicola GTX0491	LTR/Copia
RND-1_FAMILY-169#Unknown	6649	56	X.calcicola GTX0491	Unknown
RND-1_FAMILY-111#Unknown	6633	32	X.calcicola GTX0491	Unknown
RND-1_FAMILY-183#Unknown	6595	9	X.calcicola GTX0491	Unknown
RND-1_FAMILY-193#DNA	6380	10	X.calcicola GTX0491	DNA
RND-1_FAMILY-157#Unknown	6319	82	X.calcicola GTX0491	Unknown
RND-1_FAMILY-134#Unknown	6304	18	X.calcicola GTX0491	Unknown
RND-3_FAMILY-112#Unknown	6137	107	X.calcicola GTX0491	Unknown
RND-1_FAMILY-70#Unknown	5958	10	X.calcicola GTX0491	Unknown
RND-1_FAMILY-204#Unknown	5877	13	X.calcicola GTX0491	Unknown
RND-1_FAMILY-35#Unknown	5673	33	X.calcicola GTX0491	Unknown
RND-1_FAMILY-58#Unknown	5636	78	X.calcicola GTX0491	Unknown
RND-1_FAMILY-178#Unknown	5350	20	X.calcicola GTX0491	Unknown
RND-1_FAMILY-72#DNA/TcMar-Fot1	5290	30	X.calcicola GTX0491	DNA/TcMar-Fot1
RND-1_FAMILY-82#Unknown	5283	61	X.calcicola GTX0491	Unknown

RND-1_FAMILY-96#Unknown	4736	19	X.calcicola GTX0491	Unknown
RND-1_FAMILY-99#Unknown	4638	9	X.calcicola GTX0491	Unknown
RND-1_FAMILY-113#Unknown	4429	14	X.calcicola GTX0491	Unknown
RND-1_FAMILY-7#Unknown	4365	23	X.calcicola GTX0491	Unknown
RND-1_FAMILY-69#Unknown	4309	24	X.calcicola GTX0491	Unknown
RND-1_FAMILY-179#Unknown	4298	56	X.calcicola GTX0491	Unknown
RND-1_FAMILY-122#Unknown	4162	14	X.calcicola GTX0491	Unknown
RND-1_FAMILY-137#Unknown	3991	30	X.calcicola GTX0491	Unknown
RND-1_FAMILY-110#Unknown	3823	13	X.calcicola GTX0491	Unknown
RND-1_FAMILY-181#Unknown	3755	10	X.calcicola GTX0491	Unknown
(CCT)N#Simple_repeat	3707	78	X.calcicola GTX0491	Simple_repeat
RND-1_FAMILY-21#Unknown	3687	47	X.calcicola GTX0491	Unknown
RND-1_FAMILY-189#Unknown	3669	19	X.calcicola GTX0491	Unknown
RND-1_FAMILY-177#Unknown	3643	15	X.calcicola GTX0491	Unknown
RND-1_FAMILY-202#Unknown	3639	10	X.calcicola GTX0491	Unknown
RND-1_FAMILY-116#LTR/Ty3	3564	5	X.calcicola GTX0491	LTR/Ty3
RND-1_FAMILY-85#RC/Helitron	3414	2	X.calcicola GTX0491	RC/Helitron
RND-1_FAMILY-125#Unknown	3384	25	X.calcicola GTX0491	Unknown
RND-1_FAMILY-52#Unknown	3288	39	X.calcicola GTX0491	Unknown
RND-1_FAMILY-180#Unknown	3169	8	X.calcicola GTX0491	Unknown
RND-1_FAMILY-130#Unknown	3130	23	X.calcicola GTX0491	Unknown
(T)N#Simple_repeat	3121	107	X.calcicola GTX0491	Simple_repeat
RND-1_FAMILY-62#Unknown	3078	23	X.calcicola GTX0491	Unknown
RND-1_FAMILY-36#Unknown	3075	15	X.calcicola GTX0491	Unknown
RND-1_FAMILY-187#Unknown	3007	14	X.calcicola GTX0491	Unknown
RND-1_FAMILY-18#DNA/TcMar-Fot1	2993	8	X.calcicola GTX0491	DNA/TcMar-Fot1

(TCC)N#Simple_repeat	2843	55	X.calcicola GTX0491	Simple_repeat
RND-1_FAMILY-77#Unknown	2820	19	X.calcicola GTX0491	Unknown
RND-3_FAMILY-47#Unknown	2777	17	X.calcicola GTX0491	Unknown
(CTC)N#Simple_repeat	2772	56	X.calcicola GTX0491	Simple_repeat
RND-3_FAMILY-307#Unknown	2772	6	X.calcicola GTX0491	Unknown
RND-1_FAMILY-148#Unknown	2683	34	X.calcicola GTX0491	Unknown
RND-1_FAMILY-182#Unknown	2657	15	X.calcicola GTX0491	Unknown
(TA)N#Simple_repeat	2648	66	X.calcicola GTX0491	Simple_repeat
(C)N#Simple_repeat	2557	100	X.calcicola GTX0491	Simple_repeat
RND-1_FAMILY-107#Unknown	2543	12	X.calcicola GTX0491	Unknown
RND-1_FAMILY-152#Unknown	2519	8	X.calcicola GTX0491	Unknown
RND-1_FAMILY-40#Unknown	2402	16	X.calcicola GTX0491	Unknown
(G)N#Simple_repeat	2282	96	X.calcicola GTX0491	Simple_repeat
RND-1_FAMILY-45#LTR/Ty3	2273	2	X.calcicola GTX0491	LTR/Ty3
RND-1_FAMILY-162#Unknown	2187	17	X.calcicola GTX0491	Unknown
(CTT)N#Simple_repeat	2179	54	X.calcicola GTX0491	Simple_repeat
RND-1_FAMILY-117#Unknown	2170	9	X.calcicola GTX0491	Unknown
(GGT)N#Simple_repeat	2168	56	X.calcicola GTX0491	Simple_repeat
RND-1_FAMILY-28#Unknown	2109	12	X.calcicola GTX0491	Unknown
RND-1_FAMILY-192#Unknown	2094	3	X.calcicola GTX0491	Unknown
RND-1_FAMILY-47#Unknown	2087	3	X.calcicola GTX0491	Unknown
RND-1_FAMILY-25#Unknown	2083	10	X.calcicola GTX0491	Unknown
RND-1_FAMILY-133#Unknown	2074	32	X.calcicola GTX0491	Unknown
(CCA)N#Simple_repeat	2015	47	X.calcicola GTX0491	Simple_repeat
RND-1_FAMILY-78#Unknown	2007	8	X.calcicola GTX0491	Unknown
(TC)N#Simple_repeat	1997	37	X.calcicola GTX0491	Simple_repeat

RND-1_FAMILY-51#Unknown	1984	5	X.calcicola GTX0491	Unknown
(CAC)N#Simple_repeat	1974	41	X.calcicola GTX0491	Simple_repeat
RND-1_FAMILY-101#Unknown	1910	5	X.calcicola GTX0491	Unknown
RND-3_FAMILY-1291#Unknown	1902	14	X.calcicola GTX0491	Unknown
RND-1_FAMILY-140#Unknown	1878	18	X.calcicola GTX0491	Unknown
RND-1_FAMILY-120#Unknown	1765	13	X.calcicola GTX0491	Unknown
RND-1_FAMILY-129#Unknown	1720	4	X.calcicola GTX0491	Unknown
(AT)N#Simple_repeat	1685	49	X.calcicola GTX0491	Simple_repeat
RND-1_FAMILY-89#RC/Helitron	1649	1	X.calcicola GTX0491	RC/Helitron
RND-1_FAMILY-88#Unknown	1648	17	X.calcicola GTX0491	Unknown
RND-1_FAMILY-81#Unknown	1610	4	X.calcicola GTX0491	Unknown
RND-1_FAMILY-102#Unknown	1559	20	X.calcicola GTX0491	Unknown
(A)N#Simple_repeat	1551	61	X.calcicola GTX0491	Simple_repeat
RND-1_FAMILY-14#Unknown	1551	7	X.calcicola GTX0491	Unknown
RND-1_FAMILY-171#Unknown	1546	31	X.calcicola GTX0491	Unknown
(GTT)N#Simple_repeat	1545	36	X.calcicola GTX0491	Simple_repeat
(TAT)N#Simple_repeat	1504	34	X.calcicola GTX0491	Simple_repeat
(TAA)N#Simple_repeat	1497	34	X.calcicola GTX0491	Simple_repeat
RND-1_FAMILY-173#Unknown	1486	15	X.calcicola GTX0491	Unknown
(TGG)N#Simple_repeat	1458	38	X.calcicola GTX0491	Simple_repeat
(TCT)N#Simple_repeat	1428	35	X.calcicola GTX0491	Simple_repeat
(TGC)N#Simple_repeat	1330	34	X.calcicola GTX0491	Simple_repeat
(TTC)N#Simple_repeat	1328	33	X.calcicola GTX0491	Simple_repeat
(ATA)N#Simple_repeat	1320	28	X.calcicola GTX0491	Simple_repeat
(CAA)N#Simple_repeat	1295	36	X.calcicola GTX0491	Simple_repeat
RND-1_FAMILY-114#Unknown	1273	11	X.calcicola GTX0491	Unknown

(AGGGTT)N#Simple_repeat	1261	9	X.calcicola GTX0491	Simple_repeat
RND-1_FAMILY-108#Unknown	1226	13	X.calcicola GTX0491	Unknown
RND-1_FAMILY-41#Unknown	1206	8	X.calcicola GTX0491	Unknown
(GTG)N#Simple_repeat	1195	25	X.calcicola GTX0491	Simple_repeat
(TCA)N#Simple_repeat	1146	30	X.calcicola GTX0491	Simple_repeat
RND-1_FAMILY-37#Unknown	1128	6	X.calcicola GTX0491	Unknown
(TTA)N#Simple_repeat	1090	23	X.calcicola GTX0491	Simple_repeat
(CAT)N#Simple_repeat	1063	33	X.calcicola GTX0491	Simple_repeat
RND-1_FAMILY-23#Unknown	1029	12	X.calcicola GTX0491	Unknown
(GAT)N#Simple_repeat	988	29	X.calcicola GTX0491	Simple_repeat
(TGT)N#Simple_repeat	942	28	X.calcicola GTX0491	Simple_repeat
RND-1_FAMILY-73#Unknown	935	7	X.calcicola GTX0491	Unknown
(ATT)N#Simple_repeat	907	21	X.calcicola GTX0491	Simple_repeat
(GGC)N#Simple_repeat	907	22	X.calcicola GTX0491	Simple_repeat
(GAA)N#Simple_repeat	903	30	X.calcicola GTX0491	Simple_repeat
RND-1_FAMILY-57#Unknown	882	7	X.calcicola GTX0491	Unknown
(TTTA)N#Simple_repeat	853	17	X.calcicola GTX0491	Simple_repeat
(ACA)N#Simple_repeat	838	28	X.calcicola GTX0491	Simple_repeat
RND-1_FAMILY-27#Unknown	838	7	X.calcicola GTX0491	Unknown
(CAG)N#Simple_repeat	821	27	X.calcicola GTX0491	Simple_repeat
RND-1_FAMILY-68#Unknown	789	11	X.calcicola GTX0491	Unknown
(CT)N#Simple_repeat	788	24	X.calcicola GTX0491	Simple_repeat
(CGA)N#Simple_repeat	786	21	X.calcicola GTX0491	Simple_repeat
(GA)N#Simple_repeat	777	30	X.calcicola GTX0491	Simple_repeat
(AC)N#Simple_repeat	773	19	X.calcicola GTX0491	Simple_repeat
(TGGA)N#Simple_repeat	758	15	X.calcicola GTX0491	Simple_repeat

(ATC)N#Simple_repeat	747	20	X.calcicola GTX0491	Simple_repeat
(CTG)N#Simple_repeat	730	22	X.calcicola GTX0491	Simple_repeat
(TCG)N#Simple_repeat	715	20	X.calcicola GTX0491	Simple_repeat
(CGG)N#Simple_repeat	713	15	X.calcicola GTX0491	Simple_repeat
(TAACCC)N#Simple_repeat	684	4	X.calcicola GTX0491	Simple_repeat
(TGA)N#Simple_repeat	676	20	X.calcicola GTX0491	Simple_repeat
(GAC)N#Simple_repeat	668	18	X.calcicola GTX0491	Simple_repeat
(ACC)N#Simple_repeat	667	15	X.calcicola GTX0491	Simple_repeat
(GCT)N#Simple_repeat	665	18	X.calcicola GTX0491	Simple_repeat
(GCA)N#Simple_repeat	652	23	X.calcicola GTX0491	Simple_repeat
(AAT)N#Simple_repeat	649	11	X.calcicola GTX0491	Simple_repeat
(ATAA)N#Simple_repeat	630	12	X.calcicola GTX0491	Simple_repeat
(TATTA)N#Simple_repeat	630	11	X.calcicola GTX0491	Simple_repeat
(TATT)N#Simple_repeat	629	8	X.calcicola GTX0491	Simple_repeat
(TAAT)N#Simple_repeat	624	10	X.calcicola GTX0491	Simple_repeat
(AGA)N#Simple_repeat	620	24	X.calcicola GTX0491	Simple_repeat
(CA)N#Simple_repeat	579	20	X.calcicola GTX0491	Simple_repeat
(GAG)N#Simple_repeat	579	21	X.calcicola GTX0491	Simple_repeat
(TCCCCC)N#Simple_repeat	570	6	X.calcicola GTX0491	Simple_repeat
(CCG)N#Simple_repeat	567	16	X.calcicola GTX0491	Simple_repeat
(AG)N#Simple_repeat	557	20	X.calcicola GTX0491	Simple_repeat
(TCCC)N#Simple_repeat	554	12	X.calcicola GTX0491	Simple_repeat
(GGA)N#Simple_repeat	548	21	X.calcicola GTX0491	Simple_repeat
(TATAA)N#Simple_repeat	542	12	X.calcicola GTX0491	Simple_repeat
(TCTTCC)N#Simple_repeat	540	10	X.calcicola GTX0491	Simple_repeat
(TG)N#Simple_repeat	536	21	X.calcicola GTX0491	Simple_repeat

(CCTCCC)N#Simple_repeat	529	10	X.calcicola GTX0491	Simple_repeat
RND-1_FAMILY-191#Unknown	528	10	X.calcicola GTX0491	Unknown
(AATA)N#Simple_repeat	523	8	X.calcicola GTX0491	Simple_repeat
(CCTCCA)N#Simple_repeat	520	9	X.calcicola GTX0491	Simple_repeat
RND-1_FAMILY-9#Unknown	518	10	X.calcicola GTX0491	Unknown
(GT)N#Simple_repeat	515	15	X.calcicola GTX0491	Simple_repeat
(AGC)N#Simple_repeat	509	17	X.calcicola GTX0491	Simple_repeat
(ATTT)N#Simple_repeat	498	7	X.calcicola GTX0491	Simple_repeat
(TTCT)N#Simple_repeat	492	12	X.calcicola GTX0491	Simple_repeat
RND-1_FAMILY-43#Unknown	492	4	X.calcicola GTX0491	Unknown
(CCTTCC)N#Simple_repeat	488	8	X.calcicola GTX0491	Simple_repeat
(CTCC)N#Simple_repeat	465	11	X.calcicola GTX0491	Simple_repeat
(TAATA)N#Simple_repeat	460	10	X.calcicola GTX0491	Simple_repeat
(GTC)N#Simple_repeat	458	15	X.calcicola GTX0491	Simple_repeat
(TAGGGT)N#Simple_repeat	455	3	X.calcicola GTX0491	Simple_repeat
(CTCCCC)N#Simple_repeat	445	5	X.calcicola GTX0491	Simple_repeat
(CCTAAC)N#Simple_repeat	440	3	X.calcicola GTX0491	Simple_repeat
(GCG)N#Simple_repeat	440	14	X.calcicola GTX0491	Simple_repeat
(TCTCCT)N#Simple_repeat	437	5	X.calcicola GTX0491	Simple_repeat
(TTAGGG)N#Simple_repeat	437	3	X.calcicola GTX0491	Simple_repeat
(CCTCT)N#Simple_repeat	432	6	X.calcicola GTX0491	Simple_repeat
(CCCTCC)N#Simple_repeat	430	5	X.calcicola GTX0491	Simple_repeat
(TTG)N#Simple_repeat	426	16	X.calcicola GTX0491	Simple_repeat
(CCTC)N#Simple_repeat	423	8	X.calcicola GTX0491	Simple_repeat
(ATTAGAA)N#Simple_repeat	418	1	X.calcicola GTX0491	Simple_repeat
(CCCTCT)N#Simple_repeat	416	7	X.calcicola GTX0491	Simple_repeat

(TTAATA)N#Simple_repeat	415	6	X.calcicola GTX0491	Simple_repeat
(GGAT)N#Simple_repeat	413	11	X.calcicola GTX0491	Simple_repeat
(ATG)N#Simple_repeat	411	9	X.calcicola GTX0491	Simple_repeat
(CGC)N#Simple_repeat	404	13	X.calcicola GTX0491	Simple_repeat
(TATTTATTC)N#Simple_repeat	404	1	X.calcicola GTX0491	Simple_repeat
(CCAT)N#Simple_repeat	396	10	X.calcicola GTX0491	Simple_repeat
(AAG)N#Simple_repeat	378	12	X.calcicola GTX0491	Simple_repeat
(TCTTCA)N#Simple_repeat	375	8	X.calcicola GTX0491	Simple_repeat
(CGT)N#Simple_repeat	370	8	X.calcicola GTX0491	Simple_repeat
(GAGGAT)N#Simple_repeat	368	7	X.calcicola GTX0491	Simple_repeat
(AACCCT)N#Simple_repeat	367	2	X.calcicola GTX0491	Simple_repeat
(TAG)N#Simple_repeat	362	10	X.calcicola GTX0491	Simple_repeat
(CCCA)N#Simple_repeat	361	8	X.calcicola GTX0491	Simple_repeat
(CATCTC)N#Simple_repeat	358	8	X.calcicola GTX0491	Simple_repeat
(CCCCT)N#Simple_repeat	358	6	X.calcicola GTX0491	Simple_repeat
(CCTCTC)N#Simple_repeat	353	6	X.calcicola GTX0491	Simple_repeat
(TGAGGA)N#Simple_repeat	353	6	X.calcicola GTX0491	Simple_repeat
(TTTC)N#Simple_repeat	352	10	X.calcicola GTX0491	Simple_repeat
(TCCT)N#Simple_repeat	350	5	X.calcicola GTX0491	Simple_repeat
(ACCGCC)N#Simple_repeat	347	5	X.calcicola GTX0491	Simple_repeat
(TATAT)N#Simple_repeat	343	5	X.calcicola GTX0491	Simple_repeat
(CGTCAT)N#Simple_repeat	341	4	X.calcicola GTX0491	Simple_repeat
(CCCCAT)N#Simple_repeat	332	4	X.calcicola GTX0491	Simple_repeat
(GATG)N#Simple_repeat	331	10	X.calcicola GTX0491	Simple_repeat
(TCTCC)N#Simple_repeat	330	8	X.calcicola GTX0491	Simple_repeat
(ACG)N#Simple_repeat	329	12	X.calcicola GTX0491	Simple_repeat

(TCCCC)N#Simple_repeat	328	3	X.calcicola GTX0491	Simple_repeat
(CCTCTT)N#Simple_repeat	327	6	X.calcicola GTX0491	Simple_repeat
(TTTTTTA)N#Simple_repeat	325	2	X.calcicola GTX0491	Simple_repeat
(ACCCTA)N#Simple_repeat	315	3	X.calcicola GTX0491	Simple_repeat
(AAC)N#Simple_repeat	314	12	X.calcicola GTX0491	Simple_repeat
(CCGCCT)N#Simple_repeat	314	5	X.calcicola GTX0491	Simple_repeat
(CTCCTT)N#Simple_repeat	314	5	X.calcicola GTX0491	Simple_repeat
(TTATTTCAT)N#Simple_repeat	314	1	X.calcicola GTX0491	Simple_repeat
(CATCCT)N#Simple_repeat	310	4	X.calcicola GTX0491	Simple_repeat
(CCCCCA)N#Simple_repeat	310	4	X.calcicola GTX0491	Simple_repeat
(TCCTCT)N#Simple_repeat	309	4	X.calcicola GTX0491	Simple_repeat
(CAGCAA)N#Simple_repeat	308	4	X.calcicola GTX0491	Simple_repeat
(CC)N#Simple_repeat	305	6	X.calcicola GTX0491	Simple_repeat
(CCCT)N#Simple_repeat	305	9	X.calcicola GTX0491	Simple_repeat
(CATC)N#Simple_repeat	302	9	X.calcicola GTX0491	Simple_repeat
(GCC)N#Simple_repeat	299	10	X.calcicola GTX0491	Simple_repeat
(CCCATC)N#Simple_repeat	298	5	X.calcicola GTX0491	Simple_repeat
(CAAGAG)N#Simple_repeat	296	3	X.calcicola GTX0491	Simple_repeat
(CTA)N#Simple_repeat	291	8	X.calcicola GTX0491	Simple_repeat
(CCACCT)N#Simple_repeat	288	4	X.calcicola GTX0491	Simple_repeat
(TTAT)N#Simple_repeat	287	5	X.calcicola GTX0491	Simple_repeat
(CCTTC)N#Simple_repeat	284	7	X.calcicola GTX0491	Simple_repeat
(GGTGGA)N#Simple_repeat	284	6	X.calcicola GTX0491	Simple_repeat
(CCATCC)N#Simple_repeat	283	4	X.calcicola GTX0491	Simple_repeat
(AGG)N#Simple_repeat	282	10	X.calcicola GTX0491	Simple_repeat
(GTA)N#Simple_repeat	281	8	X.calcicola GTX0491	Simple_repeat

(CTTCTC)N#Simple_repeat	279	6	X.calcicola GTX0491	Simple_repeat
(TATC)N#Simple_repeat	277	5	X.calcicola GTX0491	Simple_repeat
(CCCACC)N#Simple_repeat	276	3	X.calcicola GTX0491	Simple_repeat
(TTTCT)N#Simple_repeat	273	6	X.calcicola GTX0491	Simple_repeat
(CCTTCT)N#Simple_repeat	270	4	X.calcicola GTX0491	Simple_repeat
(TCTT)N#Simple_repeat	269	7	X.calcicola GTX0491	Simple_repeat
(AATAT)N#Simple_repeat	268	3	X.calcicola GTX0491	Simple_repeat
(CCCCTC)N#Simple_repeat	267	6	X.calcicola GTX0491	Simple_repeat
(CTCTCC)N#Simple_repeat	264	4	X.calcicola GTX0491	Simple_repeat
(CTTCCC)N#Simple_repeat	263	5	X.calcicola GTX0491	Simple_repeat
(TTCTCC)N#Simple_repeat	263	4	X.calcicola GTX0491	Simple_repeat
(TTATAA)N#Simple_repeat	260	3	X.calcicola GTX0491	Simple_repeat
(GTTGGT)N#Simple_repeat	258	3	X.calcicola GTX0491	Simple_repeat
(ATATAAT)N#Simple_repeat	253	4	X.calcicola GTX0491	Simple_repeat
(CACC)N#Simple_repeat	250	5	X.calcicola GTX0491	Simple_repeat
(ATTA)N#Simple_repeat	249	6	X.calcicola GTX0491	Simple_repeat
(CCCCCAA)N#Simple_repeat	243	5	X.calcicola GTX0491	Simple_repeat
(CTTC)N#Simple_repeat	243	7	X.calcicola GTX0491	Simple_repeat
(GGAGGT)N#Simple_repeat	240	4	X.calcicola GTX0491	Simple_repeat
(GGATGA)N#Simple_repeat	240	5	X.calcicola GTX0491	Simple_repeat
(CTCCCT)N#Simple_repeat	237	4	X.calcicola GTX0491	Simple_repeat
(CCAC)N#Simple_repeat	234	4	X.calcicola GTX0491	Simple_repeat
(ATAAT)N#Simple_repeat	232	5	X.calcicola GTX0491	Simple_repeat
(GTGGAG)N#Simple_repeat	232	3	X.calcicola GTX0491	Simple_repeat
(CACCAC)N#Simple_repeat	231	3	X.calcicola GTX0491	Simple_repeat
(TCCA)N#Simple_repeat	230	6	X.calcicola GTX0491	Simple_repeat

(CTCCCA)N#Simple_repeat	229	4	X.calcicola GTX0491	Simple_repeat
(ATATT)N#Simple_repeat	226	4	X.calcicola GTX0491	Simple_repeat
(CCTA)N#Simple_repeat	226	5	X.calcicola GTX0491	Simple_repeat
(CTCCTC)N#Simple_repeat	226	4	X.calcicola GTX0491	Simple_repeat
(TATTAT)N#Simple_repeat	226	2	X.calcicola GTX0491	Simple_repeat
(TTTCTC)N#Simple_repeat	226	2	X.calcicola GTX0491	Simple_repeat
(CATCCC)N#Simple_repeat	225	5	X.calcicola GTX0491	Simple_repeat
(TCGTCT)N#Simple_repeat	222	5	X.calcicola GTX0491	Simple_repeat
(TATTATC)N#Simple_repeat	221	3	X.calcicola GTX0491	Simple_repeat
(GAGGTG)N#Simple_repeat	220	2	X.calcicola GTX0491	Simple_repeat
(CCTT)N#Simple_repeat	219	7	X.calcicola GTX0491	Simple_repeat
(ATATAA)N#Simple_repeat	218	5	X.calcicola GTX0491	Simple_repeat
(GATGAC)N#Simple_repeat	217	4	X.calcicola GTX0491	Simple_repeat
(ATTATA)N#Simple_repeat	216	3	X.calcicola GTX0491	Simple_repeat
(ATTTT)N#Simple_repeat	216	5	X.calcicola GTX0491	Simple_repeat
(ATGCTC)N#Simple_repeat	215	1	X.calcicola GTX0491	Simple_repeat
(CTCTCTCC)N#Simple_repeat	214	1	X.calcicola GTX0491	Simple_repeat
(TACC)N#Simple_repeat	214	5	X.calcicola GTX0491	Simple_repeat
(CCACAT)N#Simple_repeat	212	4	X.calcicola GTX0491	Simple_repeat
(TCTA)N#Simple_repeat	212	5	X.calcicola GTX0491	Simple_repeat
(CCATCA)N#Simple_repeat	211	4	X.calcicola GTX0491	Simple_repeat
(TATTT)N#Simple_repeat	211	5	X.calcicola GTX0491	Simple_repeat
(TCCCT)N#Simple_repeat	211	5	X.calcicola GTX0491	Simple_repeat
(CCAACA)N#Simple_repeat	210	5	X.calcicola GTX0491	Simple_repeat
(TACTATA)N#Simple_repeat	210	4	X.calcicola GTX0491	Simple_repeat
(CAACAA)N#Simple_repeat	209	2	X.calcicola GTX0491	Simple_repeat

(CCTCC)N#Simple_repeat	209	5	X.calcicola GTX0491	Simple_repeat
(GCAA)N#Simple_repeat	209	2	X.calcicola GTX0491	Simple_repeat
(ATCCC)N#Simple_repeat	208	4	X.calcicola GTX0491	Simple_repeat
(ATTAT)N#Simple_repeat	208	5	X.calcicola GTX0491	Simple_repeat
(TTTTA)N#Simple_repeat	208	6	X.calcicola GTX0491	Simple_repeat
(GGGAAA)N#Simple_repeat	207	1	X.calcicola GTX0491	Simple_repeat
(ATTTAT)N#Simple_repeat	204	4	X.calcicola GTX0491	Simple_repeat
(TTTTCT)N#Simple_repeat	204	3	X.calcicola GTX0491	Simple_repeat
(ATCTCTCC)N#Simple_repeat	201	1	X.calcicola GTX0491	Simple_repeat
(CAACAG)N#Simple_repeat	200	5	X.calcicola GTX0491	Simple_repeat
(GAGGTC)N#Simple_repeat	200	4	X.calcicola GTX0491	Simple_repeat
(AGT)N#Simple_repeat	198	6	X.calcicola GTX0491	Simple_repeat
(CCCCA)N#Simple_repeat	198	4	X.calcicola GTX0491	Simple_repeat
(CCC)N#Simple_repeat	196	2	X.calcicola GTX0491	Simple_repeat
(GGTGAG)N#Simple_repeat	196	3	X.calcicola GTX0491	Simple_repeat
(TTATA)N#Simple_repeat	195	4	X.calcicola GTX0491	Simple_repeat
(CTTCGT)N#Simple_repeat	194	1	X.calcicola GTX0491	Simple_repeat
(TCTATA)N#Simple_repeat	194	3	X.calcicola GTX0491	Simple_repeat
(TCCTTC)N#Simple_repeat	193	3	X.calcicola GTX0491	Simple_repeat
(CACCTC)N#Simple_repeat	192	4	X.calcicola GTX0491	Simple_repeat
(CACTCC)N#Simple_repeat	192	3	X.calcicola GTX0491	Simple_repeat
(CTGCGG)N#Simple_repeat	191	2	X.calcicola GTX0491	Simple_repeat
(TGGTGG)N#Simple_repeat	191	3	X.calcicola GTX0491	Simple_repeat
(CCTTCCTC)N#Simple_repeat	190	1	X.calcicola GTX0491	Simple_repeat
(GATTG)N#Simple_repeat	190	1	X.calcicola GTX0491	Simple_repeat
(CCCCCCT)N#Simple_repeat	189	2	X.calcicola GTX0491	Simple_repeat

(GGATG)N#Simple_repeat	189	5	X.calcicola GTX0491	Simple_repeat
(AATATT)N#Simple_repeat	187	4	X.calcicola GTX0491	Simple_repeat
(CCCCAA)N#Simple_repeat	187	5	X.calcicola GTX0491	Simple_repeat
(ATTAGAAA)N#Simple_repeat	185	1	X.calcicola GTX0491	Simple_repeat
(CCCTCG)N#Simple_repeat	184	3	X.calcicola GTX0491	Simple_repeat
(CCGCCA)N#Simple_repeat	184	4	X.calcicola GTX0491	Simple_repeat
(GGTGTT)N#Simple_repeat	184	3	X.calcicola GTX0491	Simple_repeat
(TGGTGT)N#Simple_repeat	184	4	X.calcicola GTX0491	Simple_repeat
(AAGGT)N#Simple_repeat	183	6	X.calcicola GTX0491	Simple_repeat
(CTCTATCC)N#Simple_repeat	183	1	X.calcicola GTX0491	Simple_repeat
(GACGAT)N#Simple_repeat	182	4	X.calcicola GTX0491	Simple_repeat
(TAC)N#Simple_repeat	182	5	X.calcicola GTX0491	Simple_repeat
(TCCCTC)N#Simple_repeat	182	5	X.calcicola GTX0491	Simple_repeat
(CTTTT)N#Simple_repeat	181	4	X.calcicola GTX0491	Simple_repeat
(CACACA)N#Simple_repeat	178	1	X.calcicola GTX0491	Simple_repeat
(CATCTT)N#Simple_repeat	178	4	X.calcicola GTX0491	Simple_repeat
(ATAG)N#Simple_repeat	175	5	X.calcicola GTX0491	Simple_repeat
(CCCAT)N#Simple_repeat	175	6	X.calcicola GTX0491	Simple_repeat
(GATGG)N#Simple_repeat	174	5	X.calcicola GTX0491	Simple_repeat
(AATTTA)N#Simple_repeat	173	2	X.calcicola GTX0491	Simple_repeat
(CCTCCG)N#Simple_repeat	173	3	X.calcicola GTX0491	Simple_repeat
(CTGCCG)N#Simple_repeat	173	1	X.calcicola GTX0491	Simple_repeat
(TAAGG)N#Simple_repeat	173	5	X.calcicola GTX0491	Simple_repeat
(GGATGT)N#Simple_repeat	172	2	X.calcicola GTX0491	Simple_repeat
(GCGGTT)N#Simple_repeat	170	3	X.calcicola GTX0491	Simple_repeat
(CATT)N#Simple_repeat	169	5	X.calcicola GTX0491	Simple_repeat

(GTGTCT)N#Simple_repeat	169	1	X.calcicola GTX0491	Simple_repeat
(CATCC)N#Simple_repeat	168	3	X.calcicola GTX0491	Simple_repeat
(AATAA)N#Simple_repeat	166	3	X.calcicola GTX0491	Simple_repeat
(CCTCCT)N#Simple_repeat	166	3	X.calcicola GTX0491	Simple_repeat
(CTTTCC)N#Simple_repeat	166	3	X.calcicola GTX0491	Simple_repeat
(AATACT)N#Simple_repeat	165	3	X.calcicola GTX0491	Simple_repeat
(CCCCCT)N#Simple_repeat	165	3	X.calcicola GTX0491	Simple_repeat
(GCAACA)N#Simple_repeat	165	2	X.calcicola GTX0491	Simple_repeat
(GGCGGA)N#Simple_repeat	165	4	X.calcicola GTX0491	Simple_repeat
(CCCTC)N#Simple_repeat	164	5	X.calcicola GTX0491	Simple_repeat
(CTTT)N#Simple_repeat	164	4	X.calcicola GTX0491	Simple_repeat
(TGGT)N#Simple_repeat	164	5	X.calcicola GTX0491	Simple_repeat
(ATCCTC)N#Simple_repeat	161	4	X.calcicola GTX0491	Simple_repeat
(CCCTT)N#Simple_repeat	160	3	X.calcicola GTX0491	Simple_repeat
(GTAGTA)N#Simple_repeat	160	2	X.calcicola GTX0491	Simple_repeat
(GATGAA)N#Simple_repeat	159	3	X.calcicola GTX0491	Simple_repeat
(TAATTA)N#Simple_repeat	159	3	X.calcicola GTX0491	Simple_repeat
(TCGTCA)N#Simple_repeat	159	3	X.calcicola GTX0491	Simple_repeat
(AGATGG)N#Simple_repeat	158	3	X.calcicola GTX0491	Simple_repeat
(GAAGAC)N#Simple_repeat	158	3	X.calcicola GTX0491	Simple_repeat
(AATAGA)N#Simple_repeat	157	3	X.calcicola GTX0491	Simple_repeat
(ATCTCC)N#Simple_repeat	157	4	X.calcicola GTX0491	Simple_repeat
(GGCGGT)N#Simple_repeat	155	2	X.calcicola GTX0491	Simple_repeat
(ATAT)N#Simple_repeat	154	4	X.calcicola GTX0491	Simple_repeat
(CCTCTTC)N#Simple_repeat	154	1	X.calcicola GTX0491	Simple_repeat
(GGATGG)N#Simple_repeat	154	4	X.calcicola GTX0491	Simple_repeat

(TGTTGCTGC)N#Simple_repeat	154	1	X.calcicola GTX0491	Simple_repeat
(TTCTAT)N#Simple_repeat	154	3	X.calcicola GTX0491	Simple_repeat
(AATATA)N#Simple_repeat	153	3	X.calcicola GTX0491	Simple_repeat
(CCATCCC)N#Simple_repeat	153	2	X.calcicola GTX0491	Simple_repeat
(CCTCAC)N#Simple_repeat	153	4	X.calcicola GTX0491	Simple_repeat
(GTGGAT)N#Simple_repeat	151	2	X.calcicola GTX0491	Simple_repeat
(TCCTCA)N#Simple_repeat	151	4	X.calcicola GTX0491	Simple_repeat
(GAAA)N#Simple_repeat	150	4	X.calcicola GTX0491	Simple_repeat
(GCAGTATCA)N#Simple_repeat	150	1	X.calcicola GTX0491	Simple_repeat
(CTTTC)N#Simple_repeat	149	4	X.calcicola GTX0491	Simple_repeat
(TACCT)N#Simple_repeat	148	5	X.calcicola GTX0491	Simple_repeat
(TTTTC)N#Simple_repeat	148	3	X.calcicola GTX0491	Simple_repeat
(ATTTA)N#Simple_repeat	147	3	X.calcicola GTX0491	Simple_repeat
(TATAAT)N#Simple_repeat	147	3	X.calcicola GTX0491	Simple_repeat
(TTGGATTGAA)N#Simple_repeat	147	1	X.calcicola GTX0491	Simple_repeat
(ATTTTTA)N#Simple_repeat	146	3	X.calcicola GTX0491	Simple_repeat
(CTAACC)N#Simple_repeat	146	1	X.calcicola GTX0491	Simple_repeat
(TATTAC)N#Simple_repeat	146	3	X.calcicola GTX0491	Simple_repeat
(TGAGAGGTGC)N#Simple_repeat	145	1	X.calcicola GTX0491	Simple_repeat
(TGGTG)N#Simple_repeat	145	3	X.calcicola GTX0491	Simple_repeat
(TTTATT)N#Simple_repeat	145	3	X.calcicola GTX0491	Simple_repeat
(CTCCAC)N#Simple_repeat	144	2	X.calcicola GTX0491	Simple_repeat
(TCCTC)N#Simple_repeat	144	4	X.calcicola GTX0491	Simple_repeat
(GGATGGA)N#Simple_repeat	142	3	X.calcicola GTX0491	Simple_repeat
(GGTG)N#Simple_repeat	142	4	X.calcicola GTX0491	Simple_repeat
(TCAC)N#Simple_repeat	142	3	X.calcicola GTX0491	Simple_repeat

(TTTAAT)N#Simple_repeat	142	4	X.calcicola GTX0491	Simple_repeat
(ATAATA)N#Simple_repeat	141	3	X.calcicola GTX0491	Simple_repeat
(CCTCAT)N#Simple_repeat	141	3	X.calcicola GTX0491	Simple_repeat
(GTGAGG)N#Simple_repeat	140	1	X.calcicola GTX0491	Simple_repeat
(TCCCCT)N#Simple_repeat	140	3	X.calcicola GTX0491	Simple_repeat
(TTCTT)N#Simple_repeat	140	5	X.calcicola GTX0491	Simple_repeat
(ATATA)N#Simple_repeat	139	3	X.calcicola GTX0491	Simple_repeat
(ATGAGG)N#Simple_repeat	139	3	X.calcicola GTX0491	Simple_repeat
(CTTCCT)N#Simple_repeat	138	3	X.calcicola GTX0491	Simple_repeat
(TTAATT)N#Simple_repeat	138	2	X.calcicola GTX0491	Simple_repeat
(TTTATC)N#Simple_repeat	138	2	X.calcicola GTX0491	Simple_repeat
(ACCCAC)N#Simple_repeat	137	3	X.calcicola GTX0491	Simple_repeat
(ATTTTA)N#Simple_repeat	137	2	X.calcicola GTX0491	Simple_repeat
(TGGAGG)N#Simple_repeat	137	3	X.calcicola GTX0491	Simple_repeat
(TGGAGGAGG)N#Simple_repeat	137	1	X.calcicola GTX0491	Simple_repeat
(ATCT)N#Simple_repeat	136	4	X.calcicola GTX0491	Simple_repeat
(TTATTTTTA)N#Simple_repeat	136	1	X.calcicola GTX0491	Simple_repeat
(ATTGG)N#Simple_repeat	135	3	X.calcicola GTX0491	Simple_repeat
(GGTTAG)N#Simple_repeat	135	1	X.calcicola GTX0491	Simple_repeat
(TAGACTA)N#Simple_repeat	135	3	X.calcicola GTX0491	Simple_repeat
(TTACC)N#Simple_repeat	135	4	X.calcicola GTX0491	Simple_repeat
(CCACTC)N#Simple_repeat	134	3	X.calcicola GTX0491	Simple_repeat
(CCTTCG)N#Simple_repeat	134	2	X.calcicola GTX0491	Simple_repeat
(CTCT)N#Simple_repeat	134	3	X.calcicola GTX0491	Simple_repeat
(GGTA)N#Simple_repeat	134	5	X.calcicola GTX0491	Simple_repeat
(TCTTTC)N#Simple_repeat	134	3	X.calcicola GTX0491	Simple_repeat

(TTCC)N#Simple_repeat	134	3	X.calcicola GTX0491	Simple_repeat
(ACCA)N#Simple_repeat	133	3	X.calcicola GTX0491	Simple_repeat
(TCTTT)N#Simple_repeat	133	3	X.calcicola GTX0491	Simple_repeat
(CCCATCC)N#Simple_repeat	132	2	X.calcicola GTX0491	Simple_repeat
(CCCTCCT)N#Simple_repeat	132	2	X.calcicola GTX0491	Simple_repeat
(CTTCTTG)N#Simple_repeat	132	2	X.calcicola GTX0491	Simple_repeat
(TTTTAT)N#Simple_repeat	132	3	X.calcicola GTX0491	Simple_repeat
(AATC)N#Simple_repeat	131	5	X.calcicola GTX0491	Simple_repeat
(ACTAACT)N#Simple_repeat	131	2	X.calcicola GTX0491	Simple_repeat
(CAGCAC)N#Simple_repeat	130	1	X.calcicola GTX0491	Simple_repeat
(CCACCG)N#Simple_repeat	130	2	X.calcicola GTX0491	Simple_repeat
(CTCTTC)N#Simple_repeat	130	3	X.calcicola GTX0491	Simple_repeat
(TAGTAT)N#Simple_repeat	130	3	X.calcicola GTX0491	Simple_repeat
(AATT)N#Simple_repeat	129	2	X.calcicola GTX0491	Simple_repeat
(ATCGCT)N#Simple_repeat	129	1	X.calcicola GTX0491	Simple_repeat
(CAAG)N#Simple_repeat	129	2	X.calcicola GTX0491	Simple_repeat
(CTCCGC)N#Simple_repeat	129	2	X.calcicola GTX0491	Simple_repeat
(GACGGA)N#Simple_repeat	129	3	X.calcicola GTX0491	Simple_repeat
(GGAA)N#Simple_repeat	129	4	X.calcicola GTX0491	Simple_repeat
(GGCGAT)N#Simple_repeat	129	4	X.calcicola GTX0491	Simple_repeat
(CCCTCCA)N#Simple_repeat	128	2	X.calcicola GTX0491	Simple_repeat
(AATGCC)N#Simple_repeat	127	3	X.calcicola GTX0491	Simple_repeat
(CCTCAG)N#Simple_repeat	127	1	X.calcicola GTX0491	Simple_repeat
(TCACC)N#Simple_repeat	127	3	X.calcicola GTX0491	Simple_repeat
(AAGATAT)N#Simple_repeat	124	2	X.calcicola GTX0491	Simple_repeat
(CCACAA)N#Simple_repeat	124	2	X.calcicola GTX0491	Simple_repeat

(CTATA)N#Simple_repeat	124	2	X.calcicola GTX0491	Simple_repeat
(GTGGTG)N#Simple_repeat	124	2	X.calcicola GTX0491	Simple_repeat
(TGACGG)N#Simple_repeat	124	1	X.calcicola GTX0491	Simple_repeat
(CTAT)N#Simple_repeat	123	3	X.calcicola GTX0491	Simple_repeat
(CTCA)N#Simple_repeat	123	3	X.calcicola GTX0491	Simple_repeat
(GAGCAG)N#Simple_repeat	123	3	X.calcicola GTX0491	Simple_repeat
(TAATAC)N#Simple_repeat	123	2	X.calcicola GTX0491	Simple_repeat
(ACCCC)N#Simple_repeat	122	2	X.calcicola GTX0491	Simple_repeat
(ATAATAT)N#Simple_repeat	122	2	X.calcicola GTX0491	Simple_repeat
(CCACAAC)N#Simple_repeat	122	1	X.calcicola GTX0491	Simple_repeat
(GTTAGG)N#Simple_repeat	122	1	X.calcicola GTX0491	Simple_repeat
(GTTGAT)N#Simple_repeat	122	2	X.calcicola GTX0491	Simple_repeat
(TCCTTT)N#Simple_repeat	122	2	X.calcicola GTX0491	Simple_repeat
(AGGTA)N#Simple_repeat	121	4	X.calcicola GTX0491	Simple_repeat
(CTCTGC)N#Simple_repeat	121	1	X.calcicola GTX0491	Simple_repeat
(GGATGTT)N#Simple_repeat	121	1	X.calcicola GTX0491	Simple_repeat
(CACCGC)N#Simple_repeat	120	3	X.calcicola GTX0491	Simple_repeat
(GGAGAT)N#Simple_repeat	120	2	X.calcicola GTX0491	Simple_repeat
(AGACGA)N#Simple_repeat	119	3	X.calcicola GTX0491	Simple_repeat
(ATATTTA)N#Simple_repeat	119	2	X.calcicola GTX0491	Simple_repeat
(CCAA)N#Simple_repeat	118	4	X.calcicola GTX0491	Simple_repeat
(CTTCC)N#Simple_repeat	118	3	X.calcicola GTX0491	Simple_repeat
(TTCTC)N#Simple_repeat	118	2	X.calcicola GTX0491	Simple_repeat
(GTTGTT)N#Simple_repeat	117	2	X.calcicola GTX0491	Simple_repeat
(TGACGA)N#Simple_repeat	117	3	X.calcicola GTX0491	Simple_repeat
(GACGAG)N#Simple_repeat	116	3	X.calcicola GTX0491	Simple_repeat

(AAAATG)N#Simple_repeat	115	1	X.calcicola GTX0491	Simple_repeat
(AGAGT)N#Simple_repeat	115	2	X.calcicola GTX0491	Simple_repeat
(ATTAA)N#Simple_repeat	115	2	X.calcicola GTX0491	Simple_repeat
(TATA)N#Simple_repeat	115	2	X.calcicola GTX0491	Simple_repeat
(TTCTAC)N#Simple_repeat	115	1	X.calcicola GTX0491	Simple_repeat
(TTTATAA)N#Simple_repeat	115	2	X.calcicola GTX0491	Simple_repeat
(GGAAGA)N#Simple_repeat	114	3	X.calcicola GTX0491	Simple_repeat
(GGGAT)N#Simple_repeat	114	3	X.calcicola GTX0491	Simple_repeat
(TCCGCC)N#Simple_repeat	114	3	X.calcicola GTX0491	Simple_repeat
(TGGTGGTGC)N#Simple_repeat	114	1	X.calcicola GTX0491	Simple_repeat
(ACACCC)N#Simple_repeat	113	2	X.calcicola GTX0491	Simple_repeat
(ACCCCC)N#Simple_repeat	113	2	X.calcicola GTX0491	Simple_repeat
(CCCCTCC)N#Simple_repeat	113	2	X.calcicola GTX0491	Simple_repeat
(GGAGGC)N#Simple_repeat	113	3	X.calcicola GTX0491	Simple_repeat
(CATCAC)N#Simple_repeat	112	3	X.calcicola GTX0491	Simple_repeat
(CTCATC)N#Simple_repeat	112	2	X.calcicola GTX0491	Simple_repeat
(CTTAC)N#Simple_repeat	112	3	X.calcicola GTX0491	Simple_repeat
(GTTGTC)N#Simple_repeat	112	2	X.calcicola GTX0491	Simple_repeat
(TAATATA)N#Simple_repeat	112	2	X.calcicola GTX0491	Simple_repeat
(TATATAT)N#Simple_repeat	112	2	X.calcicola GTX0491	Simple_repeat
(AGGAAAAG)N#Simple_repeat	111	1	X.calcicola GTX0491	Simple_repeat
(CACAC)N#Simple_repeat	111	3	X.calcicola GTX0491	Simple_repeat
(CACCCTT)N#Simple_repeat	111	2	X.calcicola GTX0491	Simple_repeat
(CCCCTT)N#Simple_repeat	111	3	X.calcicola GTX0491	Simple_repeat
(CAAC)N#Simple_repeat	110	3	X.calcicola GTX0491	Simple_repeat
(CGAGAA)N#Simple_repeat	110	1	X.calcicola GTX0491	Simple_repeat

(GAGAGT)N#Simple_repeat	110	1	X.calcicola GTX0491	Simple_repeat
(GATGTG)N#Simple_repeat	110	3	X.calcicola GTX0491	Simple_repeat
(GGTGCG)N#Simple_repeat	110	2	X.calcicola GTX0491	Simple_repeat
(TTAATAG)N#Simple_repeat	110	2	X.calcicola GTX0491	Simple_repeat
(TTTATA)N#Simple_repeat	110	2	X.calcicola GTX0491	Simple_repeat
(AGCCCT)N#Simple_repeat	109	3	X.calcicola GTX0491	Simple_repeat
(CCTCAAC)N#Simple_repeat	109	1	X.calcicola GTX0491	Simple_repeat
(GTGGAAG)N#Simple_repeat	109	2	X.calcicola GTX0491	Simple_repeat
(TCCAGC)N#Simple_repeat	109	2	X.calcicola GTX0491	Simple_repeat
(TGAAGA)N#Simple_repeat	109	2	X.calcicola GTX0491	Simple_repeat
(CCCAGT)N#Simple_repeat	108	1	X.calcicola GTX0491	Simple_repeat
(TAGCTATA)N#Simple_repeat	108	2	X.calcicola GTX0491	Simple_repeat
(TCTGCA)N#Simple_repeat	108	2	X.calcicola GTX0491	Simple_repeat
(ATATTA)N#Simple_repeat	107	2	X.calcicola GTX0491	Simple_repeat
(AAGAAT)N#Simple_repeat	106	1	X.calcicola GTX0491	Simple_repeat
(AGGT)N#Simple_repeat	106	4	X.calcicola GTX0491	Simple_repeat
(CTATTA)N#Simple_repeat	106	2	X.calcicola GTX0491	Simple_repeat
(GGTTGT)N#Simple_repeat	106	2	X.calcicola GTX0491	Simple_repeat
(TATTTC)N#Simple_repeat	106	2	X.calcicola GTX0491	Simple_repeat
(AGGATG)N#Simple_repeat	105	2	X.calcicola GTX0491	Simple_repeat
(CCCTTC)N#Simple_repeat	105	2	X.calcicola GTX0491	Simple_repeat
(GAGGGAGGA)N#Simple_repeat	105	1	X.calcicola GTX0491	Simple_repeat
(TAATT)N#Simple_repeat	105	3	X.calcicola GTX0491	Simple_repeat
(ATCCAA)N#Simple_repeat	104	2	X.calcicola GTX0491	Simple_repeat
(CTTCT)N#Simple_repeat	104	3	X.calcicola GTX0491	Simple_repeat
(GATGCA)N#Simple_repeat	104	2	X.calcicola GTX0491	Simple_repeat

(TCACCA)N#Simple_repeat	104	2	X.calcicola GTX0491	Simple_repeat
(TTTCC)N#Simple_repeat	104	3	X.calcicola GTX0491	Simple_repeat
(ACACC)N#Simple_repeat	103	1	X.calcicola GTX0491	Simple_repeat
(CCGCTC)N#Simple_repeat	103	2	X.calcicola GTX0491	Simple_repeat
(CACCATC)N#Simple_repeat	102	2	X.calcicola GTX0491	Simple_repeat
(GCCGTC)N#Simple_repeat	101	2	X.calcicola GTX0491	Simple_repeat
(TAATAT)N#Simple_repeat	101	2	X.calcicola GTX0491	Simple_repeat
(TATTTTA)N#Simple_repeat	101	2	X.calcicola GTX0491	Simple_repeat
(TCTC)N#Simple_repeat	101	2	X.calcicola GTX0491	Simple_repeat
(TTGG)N#Simple_repeat	101	2	X.calcicola GTX0491	Simple_repeat
(TATATT)N#Simple_repeat	100	1	X.calcicola GTX0491	Simple_repeat
(TCATC)N#Simple_repeat	100	3	X.calcicola GTX0491	Simple_repeat
(TCATCT)N#Simple_repeat	100	2	X.calcicola GTX0491	Simple_repeat
(AAAG)N#Simple_repeat	99	4	X.calcicola GTX0491	Simple_repeat
(CTCCAT)N#Simple_repeat	99	2	X.calcicola GTX0491	Simple_repeat
(GAGATGT)N#Simple_repeat	99	1	X.calcicola GTX0491	Simple_repeat
(TTAACTA)N#Simple_repeat	99	2	X.calcicola GTX0491	Simple_repeat
(TTCCT)N#Simple_repeat	99	3	X.calcicola GTX0491	Simple_repeat
(AACTATAT)N#Simple_repeat	98	1	X.calcicola GTX0491	Simple_repeat
(AAGGG)N#Simple_repeat	98	3	X.calcicola GTX0491	Simple_repeat
(ACT)N#Simple_repeat	98	3	X.calcicola GTX0491	Simple_repeat
(ATATATT)N#Simple_repeat	98	1	X.calcicola GTX0491	Simple_repeat
(CAACCC)N#Simple_repeat	98	2	X.calcicola GTX0491	Simple_repeat
(CACCAT)N#Simple_repeat	98	2	X.calcicola GTX0491	Simple_repeat
(CTATTAT)N#Simple_repeat	98	2	X.calcicola GTX0491	Simple_repeat
(GCGAGG)N#Simple_repeat	98	2	X.calcicola GTX0491	Simple_repeat

(GCGCCA)N#Simple_repeat	98	2	X.calcicola GTX0491	Simple_repeat
(TGTCGAGG)N#Simple_repeat	98	1	X.calcicola GTX0491	Simple_repeat
(GGTATG)N#Simple_repeat	97	1	X.calcicola GTX0491	Simple_repeat
(TCGTTC)N#Simple_repeat	97	2	X.calcicola GTX0491	Simple_repeat
(TGGTGC)N#Simple_repeat	97	2	X.calcicola GTX0491	Simple_repeat
(ACGACG)N#Simple_repeat	96	1	X.calcicola GTX0491	Simple_repeat
(ATCC)N#Simple_repeat	96	1	X.calcicola GTX0491	Simple_repeat
(CTTCTATTG)N#Simple_repeat	96	1	X.calcicola GTX0491	Simple_repeat
(GGCGTTT)N#Simple_repeat	96	1	X.calcicola GTX0491	Simple_repeat
(GGTT)N#Simple_repeat	96	2	X.calcicola GTX0491	Simple_repeat
(TATAGA)N#Simple_repeat	96	3	X.calcicola GTX0491	Simple_repeat
(TATTTAA)N#Simple_repeat	96	2	X.calcicola GTX0491	Simple_repeat
(TCCCCA)N#Simple_repeat	96	2	X.calcicola GTX0491	Simple_repeat
(CCCGCCA)N#Simple_repeat	95	1	X.calcicola GTX0491	Simple_repeat
(GAGGCG)N#Simple_repeat	95	2	X.calcicola GTX0491	Simple_repeat
(GGGA)N#Simple_repeat	95	3	X.calcicola GTX0491	Simple_repeat
(TGTA)N#Simple_repeat	95	3	X.calcicola GTX0491	Simple_repeat
(AATTT)N#Simple_repeat	94	2	X.calcicola GTX0491	Simple_repeat
(CGAAGA)N#Simple_repeat	94	3	X.calcicola GTX0491	Simple_repeat
(CGCCCC)N#Simple_repeat	94	1	X.calcicola GTX0491	Simple_repeat
(TGCTA)N#Simple_repeat	94	3	X.calcicola GTX0491	Simple_repeat
(TGGTTT)N#Simple_repeat	94	1	X.calcicola GTX0491	Simple_repeat
(AATAGT)N#Simple_repeat	93	2	X.calcicola GTX0491	Simple_repeat
(ATAGTAT)N#Simple_repeat	93	1	X.calcicola GTX0491	Simple_repeat
(ATTG)N#Simple_repeat	93	2	X.calcicola GTX0491	Simple_repeat
(CCGCCG)N#Simple_repeat	93	2	X.calcicola GTX0491	Simple_repeat

(CTCCT)N#Simple_repeat	93	2	X.calcicola GTX0491	Simple_repeat
(GAACAA)N#Simple_repeat	93	1	X.calcicola GTX0491	Simple_repeat
(GGAAAAGA)N#Simple_repeat	93	1	X.calcicola GTX0491	Simple_repeat
(GGTGGC)N#Simple_repeat	93	2	X.calcicola GTX0491	Simple_repeat
(GTGA)N#Simple_repeat	93	3	X.calcicola GTX0491	Simple_repeat
(TAGATAA)N#Simple_repeat	93	2	X.calcicola GTX0491	Simple_repeat
(TTGCGG)N#Simple_repeat	93	1	X.calcicola GTX0491	Simple_repeat
(ATAGTA)N#Simple_repeat	92	2	X.calcicola GTX0491	Simple_repeat
(ATCAC)N#Simple_repeat	92	2	X.calcicola GTX0491	Simple_repeat
(CCACCCC)N#Simple_repeat	92	2	X.calcicola GTX0491	Simple_repeat
(GGTGGT)N#Simple_repeat	92	2	X.calcicola GTX0491	Simple_repeat
(TTATATA)N#Simple_repeat	92	2	X.calcicola GTX0491	Simple_repeat
(ATTACT)N#Simple_repeat	91	2	X.calcicola GTX0491	Simple_repeat
(TTATCT)N#Simple_repeat	91	2	X.calcicola GTX0491	Simple_repeat
(TTCCTC)N#Simple_repeat	91	2	X.calcicola GTX0491	Simple_repeat
(CGCTGAA)N#Simple_repeat	90	1	X.calcicola GTX0491	Simple_repeat
(TAGG)N#Simple_repeat	90	3	X.calcicola GTX0491	Simple_repeat
(TCCTCCC)N#Simple_repeat	90	2	X.calcicola GTX0491	Simple_repeat
(TTTTTA)N#Simple_repeat	90	3	X.calcicola GTX0491	Simple_repeat
(ACCATC)N#Simple_repeat	89	2	X.calcicola GTX0491	Simple_repeat
(ACCTCA)N#Simple_repeat	89	3	X.calcicola GTX0491	Simple_repeat
(CCGAAG)N#Simple_repeat	89	2	X.calcicola GTX0491	Simple_repeat
(CCTTCCC)N#Simple_repeat	89	2	X.calcicola GTX0491	Simple_repeat
(CTAATA)N#Simple_repeat	89	2	X.calcicola GTX0491	Simple_repeat
(GGATA)N#Simple_repeat	89	1	X.calcicola GTX0491	Simple_repeat
(TATACTA)N#Simple_repeat	89	1	X.calcicola GTX0491	Simple_repeat

(TCCCCCT)N#Simple_repeat	89	2	X.calcicola GTX0491	Simple_repeat
(TCTTC)N#Simple_repeat	89	2	X.calcicola GTX0491	Simple_repeat
(TTTCTT)N#Simple_repeat	89	2	X.calcicola GTX0491	Simple_repeat
(ATATAGCT)N#Simple_repeat	88	2	X.calcicola GTX0491	Simple_repeat
(CCACCC)N#Simple_repeat	88	2	X.calcicola GTX0491	Simple_repeat
(CGTGATCCAA)N#Simple_repeat	88	1	X.calcicola GTX0491	Simple_repeat
(GGATT)N#Simple_repeat	88	2	X.calcicola GTX0491	Simple_repeat
(GGCAAC)N#Simple_repeat	88	1	X.calcicola GTX0491	Simple_repeat
(TCCACA)N#Simple_repeat	88	2	X.calcicola GTX0491	Simple_repeat
(TGATG)N#Simple_repeat	88	2	X.calcicola GTX0491	Simple_repeat
(AAAGGGA)N#Simple_repeat	87	1	X.calcicola GTX0491	Simple_repeat
(AATAATT)N#Simple_repeat	87	1	X.calcicola GTX0491	Simple_repeat
(AGCG)N#Simple_repeat	87	3	X.calcicola GTX0491	Simple_repeat
(ATCACC)N#Simple_repeat	87	1	X.calcicola GTX0491	Simple_repeat
(ATCTC)N#Simple_repeat	87	2	X.calcicola GTX0491	Simple_repeat
(CAACGC)N#Simple_repeat	87	2	X.calcicola GTX0491	Simple_repeat
(CCTATC)N#Simple_repeat	87	2	X.calcicola GTX0491	Simple_repeat
(CGGTCG)N#Simple_repeat	87	2	X.calcicola GTX0491	Simple_repeat
(GGAAG)N#Simple_repeat	87	2	X.calcicola GTX0491	Simple_repeat
(TAGCTG)N#Simple_repeat	87	1	X.calcicola GTX0491	Simple_repeat
(TGATGC)N#Simple_repeat	87	2	X.calcicola GTX0491	Simple_repeat
(AGGAGA)N#Simple_repeat	86	2	X.calcicola GTX0491	Simple_repeat
(CCTCCCC)N#Simple_repeat	86	2	X.calcicola GTX0491	Simple_repeat
(CGCCGC)N#Simple_repeat	86	2	X.calcicola GTX0491	Simple_repeat
(CTCCTG)N#Simple_repeat	86	1	X.calcicola GTX0491	Simple_repeat
(GAAG)N#Simple_repeat	86	3	X.calcicola GTX0491	Simple_repeat

(GAATA)N#Simple_repeat	86	3	X.calcicola GTX0491	Simple_repeat
(GGCATGAAC)N#Simple_repeat	86	1	X.calcicola GTX0491	Simple_repeat
(TTATTAC)N#Simple_repeat	86	1	X.calcicola GTX0491	Simple_repeat
(TTCTATA)N#Simple_repeat	86	1	X.calcicola GTX0491	Simple_repeat
(ATCCCC)N#Simple_repeat	85	1	X.calcicola GTX0491	Simple_repeat
(CCTCTCC)N#Simple_repeat	85	2	X.calcicola GTX0491	Simple_repeat
(CTAC)N#Simple_repeat	85	3	X.calcicola GTX0491	Simple_repeat
(CTCGCC)N#Simple_repeat	85	1	X.calcicola GTX0491	Simple_repeat
(GATGAG)N#Simple_repeat	85	2	X.calcicola GTX0491	Simple_repeat
(GTGT)N#Simple_repeat	85	1	X.calcicola GTX0491	Simple_repeat
(TAAGTAT)N#Simple_repeat	85	2	X.calcicola GTX0491	Simple_repeat
(TTAA)N#Simple_repeat	85	2	X.calcicola GTX0491	Simple_repeat
(CCTCGT)N#Simple_repeat	84	2	X.calcicola GTX0491	Simple_repeat
(CCTCTA)N#Simple_repeat	84	2	X.calcicola GTX0491	Simple_repeat
(CTCCTTCTC)N#Simple_repeat	84	1	X.calcicola GTX0491	Simple_repeat
(GACC)N#Simple_repeat	84	3	X.calcicola GTX0491	Simple_repeat
(GGTGGTG)N#Simple_repeat	84	1	X.calcicola GTX0491	Simple_repeat
(TAAC)N#Simple_repeat	84	2	X.calcicola GTX0491	Simple_repeat
(TATCT)N#Simple_repeat	84	2	X.calcicola GTX0491	Simple_repeat
(TTAATAA)N#Simple_repeat	84	1	X.calcicola GTX0491	Simple_repeat
(AGGTGA)N#Simple_repeat	83	2	X.calcicola GTX0491	Simple_repeat
(CCCTTAAT)N#Simple_repeat	83	1	X.calcicola GTX0491	Simple_repeat
(CGCGAT)N#Simple_repeat	83	1	X.calcicola GTX0491	Simple_repeat
(CTAGGGCCG)N#Simple_repeat	83	2	X.calcicola GTX0491	Simple_repeat
(GACGAA)N#Simple_repeat	83	2	X.calcicola GTX0491	Simple_repeat
(GCCACC)N#Simple_repeat	83	2	X.calcicola GTX0491	Simple_repeat

(GGGGAAAG)N#Simple_repeat	83	1	X.calcicola GTX0491	Simple_repeat
(CCACTCA)N#Simple_repeat	82	1	X.calcicola GTX0491	Simple_repeat
(GAAGTG)N#Simple_repeat	82	2	X.calcicola GTX0491	Simple_repeat
(GCTGCT)N#Simple_repeat	82	1	X.calcicola GTX0491	Simple_repeat
(GGGGAT)N#Simple_repeat	82	2	X.calcicola GTX0491	Simple_repeat
(TCTCT)N#Simple_repeat	82	2	X.calcicola GTX0491	Simple_repeat
(TGGTGA)N#Simple_repeat	82	1	X.calcicola GTX0491	Simple_repeat
(TGTTGC)N#Simple_repeat	82	3	X.calcicola GTX0491	Simple_repeat
(TTAAT)N#Simple_repeat	82	2	X.calcicola GTX0491	Simple_repeat
(ATAGT)N#Simple_repeat	81	2	X.calcicola GTX0491	Simple_repeat
(ATCTAG)N#Simple_repeat	81	1	X.calcicola GTX0491	Simple_repeat
(CAAGC)N#Simple_repeat	81	2	X.calcicola GTX0491	Simple_repeat
(CCTCTCCA)N#Simple_repeat	81	1	X.calcicola GTX0491	Simple_repeat
(CTTCAT)N#Simple_repeat	81	2	X.calcicola GTX0491	Simple_repeat
(CTTCTT)N#Simple_repeat	81	2	X.calcicola GTX0491	Simple_repeat
(GAAGACGG)N#Simple_repeat	81	1	X.calcicola GTX0491	Simple_repeat
(GACT)N#Simple_repeat	81	3	X.calcicola GTX0491	Simple_repeat
(TAATAA)N#Simple_repeat	81	2	X.calcicola GTX0491	Simple_repeat
(TACT)N#Simple_repeat	81	3	X.calcicola GTX0491	Simple_repeat
(CCATCAA)N#Simple_repeat	80	1	X.calcicola GTX0491	Simple_repeat
(GGCGAG)N#Simple_repeat	80	2	X.calcicola GTX0491	Simple_repeat
(TCCTCG)N#Simple_repeat	80	2	X.calcicola GTX0491	Simple_repeat
(AAGCCA)N#Simple_repeat	79	2	X.calcicola GTX0491	Simple_repeat
(AGGAA)N#Simple_repeat	79	2	X.calcicola GTX0491	Simple_repeat
(ATCTTC)N#Simple_repeat	79	2	X.calcicola GTX0491	Simple_repeat
(CAGAC)N#Simple_repeat	79	2	X.calcicola GTX0491	Simple_repeat

(CTCACT)N#Simple_repeat	79	1	X.calcicola GTX0491	Simple_repeat
(GATGATG)N#Simple_repeat	79	1	X.calcicola GTX0491	Simple_repeat
(TCCTCTC)N#Simple_repeat	79	1	X.calcicola GTX0491	Simple_repeat
(ATACTATT)N#Simple_repeat	78	1	X.calcicola GTX0491	Simple_repeat
(ATCCT)N#Simple_repeat	78	2	X.calcicola GTX0491	Simple_repeat
(GCAACTT)N#Simple_repeat	78	2	X.calcicola GTX0491	Simple_repeat
(GCCA)N#Simple_repeat	78	3	X.calcicola GTX0491	Simple_repeat
(TTACTA)N#Simple_repeat	78	2	X.calcicola GTX0491	Simple_repeat
(ATTATAG)N#Simple_repeat	77	1	X.calcicola GTX0491	Simple_repeat
(CCTCAA)N#Simple_repeat	77	1	X.calcicola GTX0491	Simple_repeat
(GCACCA)N#Simple_repeat	77	2	X.calcicola GTX0491	Simple_repeat
(GGGAGA)N#Simple_repeat	77	2	X.calcicola GTX0491	Simple_repeat
(GGTGAT)N#Simple_repeat	77	2	X.calcicola GTX0491	Simple_repeat
(AAAGA)N#Simple_repeat	76	2	X.calcicola GTX0491	Simple_repeat
(AGCAGT)N#Simple_repeat	76	1	X.calcicola GTX0491	Simple_repeat
(ATGGA)N#Simple_repeat	76	2	X.calcicola GTX0491	Simple_repeat
(GACCGC)N#Simple_repeat	76	1	X.calcicola GTX0491	Simple_repeat
(TCCAA)N#Simple_repeat	76	2	X.calcicola GTX0491	Simple_repeat
(TCTTTAA)N#Simple_repeat	76	1	X.calcicola GTX0491	Simple_repeat
(AAGAA)N#Simple_repeat	75	1	X.calcicola GTX0491	Simple_repeat
(AAGATA)N#Simple_repeat	75	1	X.calcicola GTX0491	Simple_repeat
(CTCGGA)N#Simple_repeat	75	2	X.calcicola GTX0491	Simple_repeat
(GTACGA)N#Simple_repeat	75	1	X.calcicola GTX0491	Simple_repeat
(TACTAT)N#Simple_repeat	75	2	X.calcicola GTX0491	Simple_repeat
(TCCATC)N#Simple_repeat	75	2	X.calcicola GTX0491	Simple_repeat
(ACCCCGC)N#Simple_repeat	74	1	X.calcicola GTX0491	Simple_repeat

(ATTTTAA)N#Simple_repeat	74	2	X.calcicola GTX0491	Simple_repeat
(CAGGGTTG)N#Simple_repeat	74	1	X.calcicola GTX0491	Simple_repeat
(CCTCTCT)N#Simple_repeat	74	1	X.calcicola GTX0491	Simple_repeat
(CGACAA)N#Simple_repeat	74	2	X.calcicola GTX0491	Simple_repeat
(CTTTAT)N#Simple_repeat	74	2	X.calcicola GTX0491	Simple_repeat
(GAGGAC)N#Simple_repeat	74	2	X.calcicola GTX0491	Simple_repeat
(GCAGGA)N#Simple_repeat	74	2	X.calcicola GTX0491	Simple_repeat
(TGGCGC)N#Simple_repeat	74	1	X.calcicola GTX0491	Simple_repeat
(TT)N#Simple_repeat	74	1	X.calcicola GTX0491	Simple_repeat
(AACGACA)N#Simple_repeat	73	1	X.calcicola GTX0491	Simple_repeat
(ATCGTC)N#Simple_repeat	73	2	X.calcicola GTX0491	Simple_repeat
(CATCCA)N#Simple_repeat	73	1	X.calcicola GTX0491	Simple_repeat
(CCGTGT)N#Simple_repeat	73	1	X.calcicola GTX0491	Simple_repeat
(GATTA)N#Simple_repeat	73	1	X.calcicola GTX0491	Simple_repeat
(GCTGTCGG)N#Simple_repeat	73	1	X.calcicola GTX0491	Simple_repeat
(GGCGAA)N#Simple_repeat	73	2	X.calcicola GTX0491	Simple_repeat
(GTTTTA)N#Simple_repeat	73	1	X.calcicola GTX0491	Simple_repeat
(TATTATAG)N#Simple_repeat	73	1	X.calcicola GTX0491	Simple_repeat
(TCCACC)N#Simple_repeat	73	2	X.calcicola GTX0491	Simple_repeat
(TCCCTCT)N#Simple_repeat	73	1	X.calcicola GTX0491	Simple_repeat
(AAGACG)N#Simple_repeat	72	2	X.calcicola GTX0491	Simple_repeat
(ACAA)N#Simple_repeat	72	2	X.calcicola GTX0491	Simple_repeat
(AGGG)N#Simple_repeat	72	3	X.calcicola GTX0491	Simple_repeat
(CGTCC)N#Simple_repeat	72	2	X.calcicola GTX0491	Simple_repeat
(GGAGTA)N#Simple_repeat	72	2	X.calcicola GTX0491	Simple_repeat
(GGTGCCG)N#Simple_repeat	72	1	X.calcicola GTX0491	Simple_repeat

(TATATATT)N#Simple_repeat	72	1	X.calcicola GTX0491	Simple_repeat
(TCGC)N#Simple_repeat	72	2	X.calcicola GTX0491	Simple_repeat
(TCTGCT)N#Simple_repeat	72	2	X.calcicola GTX0491	Simple_repeat
(TTCGTC)N#Simple_repeat	72	2	X.calcicola GTX0491	Simple_repeat
(ACTAGTT)N#Simple_repeat	71	1	X.calcicola GTX0491	Simple_repeat
(ATTTCT)N#Simple_repeat	71	1	X.calcicola GTX0491	Simple_repeat
(CCCAC)N#Simple_repeat	71	1	X.calcicola GTX0491	Simple_repeat
(CTTCTCA)N#Simple_repeat	71	1	X.calcicola GTX0491	Simple_repeat
(CTTTTA)N#Simple_repeat	71	2	X.calcicola GTX0491	Simple_repeat
(GATGA)N#Simple_repeat	71	2	X.calcicola GTX0491	Simple_repeat
(GCTGGT)N#Simple_repeat	71	2	X.calcicola GTX0491	Simple_repeat
(GCTGTT)N#Simple_repeat	71	2	X.calcicola GTX0491	Simple_repeat
(GGGGAAG)N#Simple_repeat	71	2	X.calcicola GTX0491	Simple_repeat
(GGTGTC)N#Simple_repeat	71	2	X.calcicola GTX0491	Simple_repeat
(TCCACGC)N#Simple_repeat	71	1	X.calcicola GTX0491	Simple_repeat
(TCCTCAACTC)N#Simple_repeat	71	1	X.calcicola GTX0491	Simple_repeat
(TCCTT)N#Simple_repeat	71	2	X.calcicola GTX0491	Simple_repeat
(TCTCCC)N#Simple_repeat	71	1	X.calcicola GTX0491	Simple_repeat
(TTTTCTT)N#Simple_repeat	71	2	X.calcicola GTX0491	Simple_repeat
(AGAGA)N#Simple_repeat	70	2	X.calcicola GTX0491	Simple_repeat
(ATCA)N#Simple_repeat	70	3	X.calcicola GTX0491	Simple_repeat
(CCAACC)N#Simple_repeat	70	2	X.calcicola GTX0491	Simple_repeat
(GCAGAA)N#Simple_repeat	70	1	X.calcicola GTX0491	Simple_repeat
(GCGATA)N#Simple_repeat	70	2	X.calcicola GTX0491	Simple_repeat
(GGGATG)N#Simple_repeat	70	2	X.calcicola GTX0491	Simple_repeat
(GTGAG)N#Simple_repeat	70	2	X.calcicola GTX0491	Simple_repeat

(TGCT)N#Simple_repeat	70	3	X.calcicola GTX0491	Simple_repeat
(TTATAAC)N#Simple_repeat	70	1	X.calcicola GTX0491	Simple_repeat
(ATAGTAA)N#Simple_repeat	69	1	X.calcicola GTX0491	Simple_repeat
(ATCATG)N#Simple_repeat	69	2	X.calcicola GTX0491	Simple_repeat
(ATGT)N#Simple_repeat	69	3	X.calcicola GTX0491	Simple_repeat
(ATTCT)N#Simple_repeat	69	2	X.calcicola GTX0491	Simple_repeat
(CCCAACC)N#Simple_repeat	69	1	X.calcicola GTX0491	Simple_repeat
(CCTCA)N#Simple_repeat	69	1	X.calcicola GTX0491	Simple_repeat
(CGGCGA)N#Simple_repeat	69	1	X.calcicola GTX0491	Simple_repeat
(GAGTCGGA)N#Simple_repeat	69	1	X.calcicola GTX0491	Simple_repeat
(GATGGT)N#Simple_repeat	69	2	X.calcicola GTX0491	Simple_repeat
(GCCCCA)N#Simple_repeat	69	2	X.calcicola GTX0491	Simple_repeat
(GGTGGCG)N#Simple_repeat	69	1	X.calcicola GTX0491	Simple_repeat
(TGTTGG)N#Simple_repeat	69	2	X.calcicola GTX0491	Simple_repeat
(AAGATG)N#Simple_repeat	68	2	X.calcicola GTX0491	Simple_repeat
(AAGATT)N#Simple_repeat	68	1	X.calcicola GTX0491	Simple_repeat
(CTCTT)N#Simple_repeat	68	2	X.calcicola GTX0491	Simple_repeat
(GTAATA)N#Simple_repeat	68	2	X.calcicola GTX0491	Simple_repeat
(TCGTTTCC)N#Simple_repeat	68	1	X.calcicola GTX0491	Simple_repeat
(CAGC)N#Simple_repeat	67	2	X.calcicola GTX0491	Simple_repeat
(CCAAC)N#Simple_repeat	67	2	X.calcicola GTX0491	Simple_repeat
(CTACT)N#Simple_repeat	67	1	X.calcicola GTX0491	Simple_repeat
(TCCTCC)N#Simple_repeat	67	2	X.calcicola GTX0491	Simple_repeat
(TCGCCA)N#Simple_repeat	67	1	X.calcicola GTX0491	Simple_repeat
(TTACTAG)N#Simple_repeat	67	1	X.calcicola GTX0491	Simple_repeat
(ATCTTT)N#Simple_repeat	66	2	X.calcicola GTX0491	Simple_repeat

(ATGAAG)N#Simple_repeat	66	1	X.calcicola GTX0491	Simple_repeat
(CATCGT)N#Simple_repeat	66	1	X.calcicola GTX0491	Simple_repeat
(CATCT)N#Simple_repeat	66	2	X.calcicola GTX0491	Simple_repeat
(CCAACG)N#Simple_repeat	66	2	X.calcicola GTX0491	Simple_repeat
(CCCATA)N#Simple_repeat	66	1	X.calcicola GTX0491	Simple_repeat
(CCTTCCTTT)N#Simple_repeat	66	1	X.calcicola GTX0491	Simple_repeat
(CTCCAAC)N#Simple_repeat	66	1	X.calcicola GTX0491	Simple_repeat
(CTTCTCC)N#Simple_repeat	66	1	X.calcicola GTX0491	Simple_repeat
(GAAGTT)N#Simple_repeat	66	1	X.calcicola GTX0491	Simple_repeat
(TAATTT)N#Simple_repeat	66	1	X.calcicola GTX0491	Simple_repeat
(TACTTC)N#Simple_repeat	66	2	X.calcicola GTX0491	Simple_repeat
(TCCTA)N#Simple_repeat	66	2	X.calcicola GTX0491	Simple_repeat
(TCTAATT)N#Simple_repeat	66	1	X.calcicola GTX0491	Simple_repeat
(TGCTT)N#Simple_repeat	66	2	X.calcicola GTX0491	Simple_repeat
(TTTTATAG)N#Simple_repeat	66	1	X.calcicola GTX0491	Simple_repeat
(ATCATA)N#Simple_repeat	65	1	X.calcicola GTX0491	Simple_repeat
(ATCGAT)N#Simple_repeat	65	1	X.calcicola GTX0491	Simple_repeat
(ATGG)N#Simple_repeat	65	2	X.calcicola GTX0491	Simple_repeat
(ATTATAT)N#Simple_repeat	65	2	X.calcicola GTX0491	Simple_repeat
(ATTATTA)N#Simple_repeat	65	1	X.calcicola GTX0491	Simple_repeat
(CAACA)N#Simple_repeat	65	2	X.calcicola GTX0491	Simple_repeat
(CAGTACCAC)N#Simple_repeat	65	1	X.calcicola GTX0491	Simple_repeat
(CCACCACA)N#Simple_repeat	65	1	X.calcicola GTX0491	Simple_repeat
(CCAGCA)N#Simple_repeat	65	2	X.calcicola GTX0491	Simple_repeat
(GGTGGTA)N#Simple_repeat	65	1	X.calcicola GTX0491	Simple_repeat
(TCTAC)N#Simple_repeat	65	2	X.calcicola GTX0491	Simple_repeat

(TCTCACTC)N#Simple_repeat	65	1	X.calcicola GTX0491	Simple_repeat
(TCTTCCC)N#Simple_repeat	65	1	X.calcicola GTX0491	Simple_repeat
(TCTTCG)N#Simple_repeat	65	1	X.calcicola GTX0491	Simple_repeat
(TTATAGT)N#Simple_repeat	65	1	X.calcicola GTX0491	Simple_repeat
(TTTAC)N#Simple_repeat	65	2	X.calcicola GTX0491	Simple_repeat
(ATCCA)N#Simple_repeat	64	2	X.calcicola GTX0491	Simple_repeat
(CCAACGC)N#Simple_repeat	64	1	X.calcicola GTX0491	Simple_repeat
(CGGTTG)N#Simple_repeat	64	1	X.calcicola GTX0491	Simple_repeat
(CTCAT)N#Simple_repeat	64	1	X.calcicola GTX0491	Simple_repeat
(CTCCGT)N#Simple_repeat	64	1	X.calcicola GTX0491	Simple_repeat
(GACAAC)N#Simple_repeat	64	1	X.calcicola GTX0491	Simple_repeat
(GTAAG)N#Simple_repeat	64	2	X.calcicola GTX0491	Simple_repeat
(TTGATTTGC)N#Simple_repeat	64	1	X.calcicola GTX0491	Simple_repeat
(TTGCCG)N#Simple_repeat	64	1	X.calcicola GTX0491	Simple_repeat
(AGGGAG)N#Simple_repeat	63	2	X.calcicola GTX0491	Simple_repeat
(CAGCA)N#Simple_repeat	63	1	X.calcicola GTX0491	Simple_repeat
(GAAGAT)N#Simple_repeat	63	1	X.calcicola GTX0491	Simple_repeat
(GACAAT)N#Simple_repeat	63	1	X.calcicola GTX0491	Simple_repeat
(TACCTA)N#Simple_repeat	63	1	X.calcicola GTX0491	Simple_repeat
(TCTCCAC)N#Simple_repeat	63	1	X.calcicola GTX0491	Simple_repeat
(TTCCCCT)N#Simple_repeat	63	1	X.calcicola GTX0491	Simple_repeat
(TTCTGC)N#Simple_repeat	63	2	X.calcicola GTX0491	Simple_repeat
(TTGCTT)N#Simple_repeat	63	1	X.calcicola GTX0491	Simple_repeat
(TTGTTG)N#Simple_repeat	63	1	X.calcicola GTX0491	Simple_repeat
(AATAG)N#Simple_repeat	62	2	X.calcicola GTX0491	Simple_repeat
(AGGTG)N#Simple_repeat	62	2	X.calcicola GTX0491	Simple_repeat

(CGTCCG)N#Simple_repeat	62	1	X.calcicola GTX0491	Simple_repeat
(GAGGAA)N#Simple_repeat	62	2	X.calcicola GTX0491	Simple_repeat
(GGATGC)N#Simple_repeat	62	2	X.calcicola GTX0491	Simple_repeat
(TAGTAAAGA)N#Simple_repeat	62	1	X.calcicola GTX0491	Simple_repeat
(TATCC)N#Simple_repeat	62	2	X.calcicola GTX0491	Simple_repeat
(TCCCCCC)N#Simple_repeat	62	2	X.calcicola GTX0491	Simple_repeat
(TGCA)N#Simple_repeat	62	2	X.calcicola GTX0491	Simple_repeat
(AGGATA)N#Simple_repeat	61	1	X.calcicola GTX0491	Simple_repeat
(CACCGG)N#Simple_repeat	61	1	X.calcicola GTX0491	Simple_repeat
(CATGAG)N#Simple_repeat	61	1	X.calcicola GTX0491	Simple_repeat
(CCCCACC)N#Simple_repeat	61	1	X.calcicola GTX0491	Simple_repeat
(CGCCTT)N#Simple_repeat	61	1	X.calcicola GTX0491	Simple_repeat
(CTACCTA)N#Simple_repeat	61	1	X.calcicola GTX0491	Simple_repeat
(CTATCTA)N#Simple_repeat	61	1	X.calcicola GTX0491	Simple_repeat
(CTTCTA)N#Simple_repeat	61	2	X.calcicola GTX0491	Simple_repeat
(CTTTTTTTT)N#Simple_repeat	61	1	X.calcicola GTX0491	Simple_repeat
(GATCTT)N#Simple_repeat	61	2	X.calcicola GTX0491	Simple_repeat
(GGGAAT)N#Simple_repeat	61	1	X.calcicola GTX0491	Simple_repeat
(GGTGAGG)N#Simple_repeat	61	1	X.calcicola GTX0491	Simple_repeat
(TAATCTC)N#Simple_repeat	61	1	X.calcicola GTX0491	Simple_repeat
(TCCCTCC)N#Simple_repeat	61	1	X.calcicola GTX0491	Simple_repeat
(TGAGCT)N#Simple_repeat	61	1	X.calcicola GTX0491	Simple_repeat
(TGGCGG)N#Simple_repeat	61	2	X.calcicola GTX0491	Simple_repeat
(ACGGTAA)N#Simple_repeat	60	1	X.calcicola GTX0491	Simple_repeat
(AGCACGA)N#Simple_repeat	60	1	X.calcicola GTX0491	Simple_repeat
(AGTAT)N#Simple_repeat	60	1	X.calcicola GTX0491	Simple_repeat

(ATTTAG)N#Simple_repeat	60	1	X.calcicola GTX0491	Simple_repeat
(CCCCTTCA)N#Simple_repeat	60	1	X.calcicola GTX0491	Simple_repeat
(CTCTTCG)N#Simple_repeat	60	1	X.calcicola GTX0491	Simple_repeat
(GCCTGA)N#Simple_repeat	60	1	X.calcicola GTX0491	Simple_repeat
(GCTTGA)N#Simple_repeat	60	1	X.calcicola GTX0491	Simple_repeat
(GGACGA)N#Simple_repeat	60	2	X.calcicola GTX0491	Simple_repeat
(GGTAGGGAGT)N#Simple_repeat	60	1	X.calcicola GTX0491	Simple_repeat
(GTTGCT)N#Simple_repeat	60	2	X.calcicola GTX0491	Simple_repeat
(TCCCA)N#Simple_repeat	60	1	X.calcicola GTX0491	Simple_repeat
(ATACT)N#Simple_repeat	59	1	X.calcicola GTX0491	Simple_repeat
(CAGAT)N#Simple_repeat	59	2	X.calcicola GTX0491	Simple_repeat
(GATCTCC)N#Simple_repeat	59	1	X.calcicola GTX0491	Simple_repeat
(TACTTAT)N#Simple_repeat	59	1	X.calcicola GTX0491	Simple_repeat
(TAGGTAA)N#Simple_repeat	59	1	X.calcicola GTX0491	Simple_repeat
(TATAAGTA)N#Simple_repeat	59	1	X.calcicola GTX0491	Simple_repeat
(TGGGA)N#Simple_repeat	59	2	X.calcicola GTX0491	Simple_repeat
(ACTAAT)N#Simple_repeat	58	1	X.calcicola GTX0491	Simple_repeat
(CATGCTC)N#Simple_repeat	58	1	X.calcicola GTX0491	Simple_repeat
(CTAG)N#Simple_repeat	58	1	X.calcicola GTX0491	Simple_repeat
(CTCTC)N#Simple_repeat	58	2	X.calcicola GTX0491	Simple_repeat
(GACTTG)N#Simple_repeat	58	1	X.calcicola GTX0491	Simple_repeat
(GAGCAGA)N#Simple_repeat	58	1	X.calcicola GTX0491	Simple_repeat
(GCCTC)N#Simple_repeat	58	1	X.calcicola GTX0491	Simple_repeat
(TAGA)N#Simple_repeat	58	1	X.calcicola GTX0491	Simple_repeat
(TATTAA)N#Simple_repeat	58	1	X.calcicola GTX0491	Simple_repeat
(TATTTTAACT)N#Simple_repeat	58	1	X.calcicola GTX0491	Simple_repeat

(TGATGT)N#Simple_repeat	58	1	X.calcicola GTX0491	Simple_repeat
(ATAGA)N#Simple_repeat	57	1	X.calcicola GTX0491	Simple_repeat
(CAGCACTAC)N#Simple_repeat	57	1	X.calcicola GTX0491	Simple_repeat
(CCATCT)N#Simple_repeat	57	1	X.calcicola GTX0491	Simple_repeat
(CGACGA)N#Simple_repeat	57	1	X.calcicola GTX0491	Simple_repeat
(GCCGAG)N#Simple_repeat	57	1	X.calcicola GTX0491	Simple_repeat
(GCTGACT)N#Simple_repeat	57	1	X.calcicola GTX0491	Simple_repeat
(GGTGGAG)N#Simple_repeat	57	1	X.calcicola GTX0491	Simple_repeat
(TACTCCTCG)N#Simple_repeat	57	1	X.calcicola GTX0491	Simple_repeat
(TGCCCGA)N#Simple_repeat	57	1	X.calcicola GTX0491	Simple_repeat
(TTTATAT)N#Simple_repeat	57	1	X.calcicola GTX0491	Simple_repeat
(CAACAC)N#Simple_repeat	56	1	X.calcicola GTX0491	Simple_repeat
(CCCCCC)N#Simple_repeat	56	1	X.calcicola GTX0491	Simple_repeat
(CCCTCAC)N#Simple_repeat	56	1	X.calcicola GTX0491	Simple_repeat
(CTAAGAA)N#Simple_repeat	56	1	X.calcicola GTX0491	Simple_repeat
(CTTCGC)N#Simple_repeat	56	1	X.calcicola GTX0491	Simple_repeat
(GACGTC)N#Simple_repeat	56	1	X.calcicola GTX0491	Simple_repeat
(GCTC)N#Simple_repeat	56	2	X.calcicola GTX0491	Simple_repeat
(GTAGAA)N#Simple_repeat	56	1	X.calcicola GTX0491	Simple_repeat
(TAATCTA)N#Simple_repeat	56	1	X.calcicola GTX0491	Simple_repeat
(TCTACC)N#Simple_repeat	56	1	X.calcicola GTX0491	Simple_repeat
(TTTGCA)N#Simple_repeat	56	1	X.calcicola GTX0491	Simple_repeat
(TTTGTG)N#Simple_repeat	56	1	X.calcicola GTX0491	Simple_repeat
(ATATAAG)N#Simple_repeat	55	1	X.calcicola GTX0491	Simple_repeat
(ATTAGAT)N#Simple_repeat	55	1	X.calcicola GTX0491	Simple_repeat
(CACTCCC)N#Simple_repeat	55	1	X.calcicola GTX0491	Simple_repeat

(CATCAT)N#Simple_repeat	55	1	X.calcicola GTX0491	Simple_repeat
(CTAAT)N#Simple_repeat	55	2	X.calcicola GTX0491	Simple_repeat
(CTCCCCC)N#Simple_repeat	55	1	X.calcicola GTX0491	Simple_repeat
(CTGCTT)N#Simple_repeat	55	1	X.calcicola GTX0491	Simple_repeat
(GACGGAA)N#Simple_repeat	55	1	X.calcicola GTX0491	Simple_repeat
(GGAG)N#Simple_repeat	55	2	X.calcicola GTX0491	Simple_repeat
(GGGTAT)N#Simple_repeat	55	1	X.calcicola GTX0491	Simple_repeat
(TAGATAC)N#Simple_repeat	55	1	X.calcicola GTX0491	Simple_repeat
(TATCTAA)N#Simple_repeat	55	1	X.calcicola GTX0491	Simple_repeat
(TATTACG)N#Simple_repeat	55	1	X.calcicola GTX0491	Simple_repeat
(TCCACT)N#Simple_repeat	55	1	X.calcicola GTX0491	Simple_repeat
(TCTCCCC)N#Simple_repeat	55	1	X.calcicola GTX0491	Simple_repeat
(TCTCTCCC)N#Simple_repeat	55	1	X.calcicola GTX0491	Simple_repeat
(TCTTCCG)N#Simple_repeat	55	1	X.calcicola GTX0491	Simple_repeat
(TGAT)N#Simple_repeat	55	2	X.calcicola GTX0491	Simple_repeat
(TGTC)N#Simple_repeat	55	2	X.calcicola GTX0491	Simple_repeat
(TTAGTTA)N#Simple_repeat	55	1	X.calcicola GTX0491	Simple_repeat
(TTATCTA)N#Simple_repeat	55	1	X.calcicola GTX0491	Simple_repeat
(AATAGGA)N#Simple_repeat	54	1	X.calcicola GTX0491	Simple_repeat
(ACCACCA)N#Simple_repeat	54	1	X.calcicola GTX0491	Simple_repeat
(ACCCCA)N#Simple_repeat	54	1	X.calcicola GTX0491	Simple_repeat
(AGAATTT)N#Simple_repeat	54	1	X.calcicola GTX0491	Simple_repeat
(GAGAC)N#Simple_repeat	54	2	X.calcicola GTX0491	Simple_repeat
(TATAACT)N#Simple_repeat	54	1	X.calcicola GTX0491	Simple_repeat
(TATATTA)N#Simple_repeat	54	1	X.calcicola GTX0491	Simple_repeat
(TGAGGTA)N#Simple_repeat	54	1	X.calcicola GTX0491	Simple_repeat

(AAAAG)N#Simple_repeat	53	1	X.calcicola GTX0491	Simple_repeat
(AATTAT)N#Simple_repeat	53	1	X.calcicola GTX0491	Simple_repeat
(AGAGGGAGA)N#Simple_repeat	53	1	X.calcicola GTX0491	Simple_repeat
(ATGTGGAG)N#Simple_repeat	53	1	X.calcicola GTX0491	Simple_repeat
(CCCCAC)N#Simple_repeat	53	1	X.calcicola GTX0491	Simple_repeat
(GAAGCC)N#Simple_repeat	53	1	X.calcicola GTX0491	Simple_repeat
(GACCCG)N#Simple_repeat	53	1	X.calcicola GTX0491	Simple_repeat
(GGGAA)N#Simple_repeat	53	1	X.calcicola GTX0491	Simple_repeat
(GTTTTTT)N#Simple_repeat	53	1	X.calcicola GTX0491	Simple_repeat
(TAATTAA)N#Simple_repeat	53	1	X.calcicola GTX0491	Simple_repeat
(TACCTC)N#Simple_repeat	53	1	X.calcicola GTX0491	Simple_repeat
(TATATCC)N#Simple_repeat	53	1	X.calcicola GTX0491	Simple_repeat
(TATTATT)N#Simple_repeat	53	1	X.calcicola GTX0491	Simple_repeat
(TGCTGCATC)N#Simple_repeat	53	1	X.calcicola GTX0491	Simple_repeat
(TTTTCCG)N#Simple_repeat	53	1	X.calcicola GTX0491	Simple_repeat
(ACCT)N#Simple_repeat	52	2	X.calcicola GTX0491	Simple_repeat
(AGGAAGTG)N#Simple_repeat	52	1	X.calcicola GTX0491	Simple_repeat
(CAGGAG)N#Simple_repeat	52	1	X.calcicola GTX0491	Simple_repeat
(CCCCGC)N#Simple_repeat	52	1	X.calcicola GTX0491	Simple_repeat
(CGCCTCC)N#Simple_repeat	52	1	X.calcicola GTX0491	Simple_repeat
(CTCGTC)N#Simple_repeat	52	1	X.calcicola GTX0491	Simple_repeat
(GATCTG)N#Simple_repeat	52	1	X.calcicola GTX0491	Simple_repeat
(GTTGATG)N#Simple_repeat	52	1	X.calcicola GTX0491	Simple_repeat
(TAATTTA)N#Simple_repeat	52	1	X.calcicola GTX0491	Simple_repeat
(TATACT)N#Simple_repeat	52	1	X.calcicola GTX0491	Simple_repeat
(TATCA)N#Simple_repeat	52	2	X.calcicola GTX0491	Simple_repeat

(TATTTA)N#Simple_repeat	52	1	X.calcicola GTX0491	Simple_repeat
(TCCCTTT)N#Simple_repeat	52	1	X.calcicola GTX0491	Simple_repeat
(TCGACGATC)N#Simple_repeat	52	1	X.calcicola GTX0491	Simple_repeat
(TGGTGGA)N#Simple_repeat	52	1	X.calcicola GTX0491	Simple_repeat
(TTCTTTC)N#Simple_repeat	52	1	X.calcicola GTX0491	Simple_repeat
(AACACC)N#Simple_repeat	51	1	X.calcicola GTX0491	Simple_repeat
(AAGG)N#Simple_repeat	51	2	X.calcicola GTX0491	Simple_repeat
(ACCAAACC)N#Simple_repeat	51	1	X.calcicola GTX0491	Simple_repeat
(ACCATTC)N#Simple_repeat	51	1	X.calcicola GTX0491	Simple_repeat
(AGAA)N#Simple_repeat	51	2	X.calcicola GTX0491	Simple_repeat
(AGCC)N#Simple_repeat	51	2	X.calcicola GTX0491	Simple_repeat
(AGTG)N#Simple_repeat	51	2	X.calcicola GTX0491	Simple_repeat
(ATGATTT)N#Simple_repeat	51	1	X.calcicola GTX0491	Simple_repeat
(CAAT)N#Simple_repeat	51	2	X.calcicola GTX0491	Simple_repeat
(CATCCCTA)N#Simple_repeat	51	1	X.calcicola GTX0491	Simple_repeat
(CCACAAG)N#Simple_repeat	51	1	X.calcicola GTX0491	Simple_repeat
(CCCACTC)N#Simple_repeat	51	1	X.calcicola GTX0491	Simple_repeat
(CCGCTG)N#Simple_repeat	51	1	X.calcicola GTX0491	Simple_repeat
(CGGCGC)N#Simple_repeat	51	1	X.calcicola GTX0491	Simple_repeat
(CTGGTG)N#Simple_repeat	51	1	X.calcicola GTX0491	Simple_repeat
(GCCGAC)N#Simple_repeat	51	1	X.calcicola GTX0491	Simple_repeat
(TCCCATTC)N#Simple_repeat	51	1	X.calcicola GTX0491	Simple_repeat
(TGATCA)N#Simple_repeat	51	1	X.calcicola GTX0491	Simple_repeat
(TTAGTA)N#Simple_repeat	51	1	X.calcicola GTX0491	Simple_repeat
(TTCAATA)N#Simple_repeat	51	1	X.calcicola GTX0491	Simple_repeat
(TTCCAC)N#Simple_repeat	51	1	X.calcicola GTX0491	Simple_repeat

(TTTCTA)N#Simple_repeat	51	1	X.calcicola GTX0491	Simple_repeat
(AACGAC)N#Simple_repeat	50	1	X.calcicola GTX0491	Simple_repeat
(AGAGGAGA)N#Simple_repeat	50	1	X.calcicola GTX0491	Simple_repeat
(ATATACTT)N#Simple_repeat	50	1	X.calcicola GTX0491	Simple_repeat
(CAACTCCC)N#Simple_repeat	50	1	X.calcicola GTX0491	Simple_repeat
(CCCTCCC)N#Simple_repeat	50	1	X.calcicola GTX0491	Simple_repeat
(CGTGGC)N#Simple_repeat	50	1	X.calcicola GTX0491	Simple_repeat
(GGTTG)N#Simple_repeat	50	1	X.calcicola GTX0491	Simple_repeat
(GTCGGGA)N#Simple_repeat	50	1	X.calcicola GTX0491	Simple_repeat
(TTACTTC)N#Simple_repeat	50	1	X.calcicola GTX0491	Simple_repeat
(AATATTA)N#Simple_repeat	49	1	X.calcicola GTX0491	Simple_repeat
(ACGGAC)N#Simple_repeat	49	1	X.calcicola GTX0491	Simple_repeat
(ATCTA)N#Simple_repeat	49	1	X.calcicola GTX0491	Simple_repeat
(CAGAGA)N#Simple_repeat	49	1	X.calcicola GTX0491	Simple_repeat
(CATA)N#Simple_repeat	49	2	X.calcicola GTX0491	Simple_repeat
(CCACTT)N#Simple_repeat	49	1	X.calcicola GTX0491	Simple_repeat
(CCGCAA)N#Simple_repeat	49	1	X.calcicola GTX0491	Simple_repeat
(CCGCAG)N#Simple_repeat	49	1	X.calcicola GTX0491	Simple_repeat
(CCTGT)N#Simple_repeat	49	1	X.calcicola GTX0491	Simple_repeat
(CCTTA)N#Simple_repeat	49	2	X.calcicola GTX0491	Simple_repeat
(CTACCT)N#Simple_repeat	49	1	X.calcicola GTX0491	Simple_repeat
(GGAAGT)N#Simple_repeat	49	1	X.calcicola GTX0491	Simple_repeat
(GGAGC)N#Simple_repeat	49	1	X.calcicola GTX0491	Simple_repeat
(GGAGCT)N#Simple_repeat	49	1	X.calcicola GTX0491	Simple_repeat
(GGGTTAG)N#Simple_repeat	49	1	X.calcicola GTX0491	Simple_repeat
(GTTTTG)N#Simple_repeat	49	1	X.calcicola GTX0491	Simple_repeat

(TCCATCA)N#Simple_repeat	49	1	X.calcicola GTX0491	Simple_repeat
(TCTAA)N#Simple_repeat	49	1	X.calcicola GTX0491	Simple_repeat
(TGAG)N#Simple_repeat	49	2	X.calcicola GTX0491	Simple_repeat
(TGCATT)N#Simple_repeat	49	1	X.calcicola GTX0491	Simple_repeat
(ACGGCTACTA)N#Simple_repeat	48	1	X.calcicola GTX0491	Simple_repeat
(AGTCGG)N#Simple_repeat	48	1	X.calcicola GTX0491	Simple_repeat
(ATTTCTA)N#Simple_repeat	48	1	X.calcicola GTX0491	Simple_repeat
(CCACCA)N#Simple_repeat	48	1	X.calcicola GTX0491	Simple_repeat
(CCACTCC)N#Simple_repeat	48	1	X.calcicola GTX0491	Simple_repeat
(CCCCACG)N#Simple_repeat	48	1	X.calcicola GTX0491	Simple_repeat
(CCCTTCC)N#Simple_repeat	48	1	X.calcicola GTX0491	Simple_repeat
(CGACCT)N#Simple_repeat	48	1	X.calcicola GTX0491	Simple_repeat
(CTAGATATAGA)N#Simple_repeat	48	1	X.calcicola GTX0491	Simple_repeat
(CTCACC)N#Simple_repeat	48	1	X.calcicola GTX0491	Simple_repeat
(CTCATG)N#Simple_repeat	48	1	X.calcicola GTX0491	Simple_repeat
(GATATA)N#Simple_repeat	48	1	X.calcicola GTX0491	Simple_repeat
(GATT)N#Simple_repeat	48	2	X.calcicola GTX0491	Simple_repeat
(GTAGTG)N#Simple_repeat	48	1	X.calcicola GTX0491	Simple_repeat
(TCCTCCA)N#Simple_repeat	48	1	X.calcicola GTX0491	Simple_repeat
(TCCTTTTAT)N#Simple_repeat	48	1	X.calcicola GTX0491	Simple_repeat
(TCGAA)N#Simple_repeat	48	1	X.calcicola GTX0491	Simple_repeat
(TGGTCG)N#Simple_repeat	48	1	X.calcicola GTX0491	Simple_repeat
(TGGTGCTG)N#Simple_repeat	48	1	X.calcicola GTX0491	Simple_repeat
(TTATTT)N#Simple_repeat	48	1	X.calcicola GTX0491	Simple_repeat
(TTTAA)N#Simple_repeat	48	1	X.calcicola GTX0491	Simple_repeat
(TTTCATG)N#Simple_repeat	48	1	X.calcicola GTX0491	Simple_repeat

(TTTTTT)N#Simple_repeat	48	1	X.calcicola GTX0491	Simple_repeat
(AATATAT)N#Simple_repeat	47	1	X.calcicola GTX0491	Simple_repeat
(AATATCTT)N#Simple_repeat	47	1	X.calcicola GTX0491	Simple_repeat
(ACGCCA)N#Simple_repeat	47	1	X.calcicola GTX0491	Simple_repeat
(AGCTCC)N#Simple_repeat	47	1	X.calcicola GTX0491	Simple_repeat
(AGGTAAT)N#Simple_repeat	47	1	X.calcicola GTX0491	Simple_repeat
(AGTA)N#Simple_repeat	47	2	X.calcicola GTX0491	Simple_repeat
(ATACTA)N#Simple_repeat	47	1	X.calcicola GTX0491	Simple_repeat
(ATATAGC)N#Simple_repeat	47	1	X.calcicola GTX0491	Simple_repeat
(ATTAAT)N#Simple_repeat	47	1	X.calcicola GTX0491	Simple_repeat
(ATTATCT)N#Simple_repeat	47	1	X.calcicola GTX0491	Simple_repeat
(CATAGT)N#Simple_repeat	47	1	X.calcicola GTX0491	Simple_repeat
(CATCCGC)N#Simple_repeat	47	1	X.calcicola GTX0491	Simple_repeat
(CCATCCCA)N#Simple_repeat	47	1	X.calcicola GTX0491	Simple_repeat
(CCTAAT)N#Simple_repeat	47	1	X.calcicola GTX0491	Simple_repeat
(CTTAGA)N#Simple_repeat	47	1	X.calcicola GTX0491	Simple_repeat
(CTTCCG)N#Simple_repeat	47	1	X.calcicola GTX0491	Simple_repeat
(CTTTTT)N#Simple_repeat	47	1	X.calcicola GTX0491	Simple_repeat
(GATTTG)N#Simple_repeat	47	1	X.calcicola GTX0491	Simple_repeat
(GGAAGGT)N#Simple_repeat	47	1	X.calcicola GTX0491	Simple_repeat
(GGACTTG)N#Simple_repeat	47	1	X.calcicola GTX0491	Simple_repeat
(GGATAA)N#Simple_repeat	47	1	X.calcicola GTX0491	Simple_repeat
(GTGG)N#Simple_repeat	47	1	X.calcicola GTX0491	Simple_repeat
(TACCTTC)N#Simple_repeat	47	1	X.calcicola GTX0491	Simple_repeat
(TCACCC)N#Simple_repeat	47	1	X.calcicola GTX0491	Simple_repeat
(TCTAGTCTAA)N#Simple_repeat	47	1	X.calcicola GTX0491	Simple_repeat

(TCTCCAA)N#Simple_repeat	47	1	X.calcicola GTX0491	Simple_repeat
(TCTCTA)N#Simple_repeat	47	1	X.calcicola GTX0491	Simple_repeat
(TGGTAG)N#Simple_repeat	47	1	X.calcicola GTX0491	Simple_repeat
(ACCTT)N#Simple_repeat	46	2	X.calcicola GTX0491	Simple_repeat
(ACGATC)N#Simple_repeat	46	1	X.calcicola GTX0491	Simple_repeat
(CAACTGTA)N#Simple_repeat	46	1	X.calcicola GTX0491	Simple_repeat
(CCGTCCA)N#Simple_repeat	46	1	X.calcicola GTX0491	Simple_repeat
(CCTCCAA)N#Simple_repeat	46	1	X.calcicola GTX0491	Simple_repeat
(CCTCGC)N#Simple_repeat	46	1	X.calcicola GTX0491	Simple_repeat
(CCTCTGC)N#Simple_repeat	46	1	X.calcicola GTX0491	Simple_repeat
(CGAG)N#Simple_repeat	46	1	X.calcicola GTX0491	Simple_repeat
(CTACTA)N#Simple_repeat	46	1	X.calcicola GTX0491	Simple_repeat
(CTTCCTCCTT)N#Simple_repeat	46	1	X.calcicola GTX0491	Simple_repeat
(GATGGAG)N#Simple_repeat	46	1	X.calcicola GTX0491	Simple_repeat
(GCCCCT)N#Simple_repeat	46	1	X.calcicola GTX0491	Simple_repeat
(GCCTTTT)N#Simple_repeat	46	1	X.calcicola GTX0491	Simple_repeat
(GGAGTCG)N#Simple_repeat	46	1	X.calcicola GTX0491	Simple_repeat
(GTCCTC)N#Simple_repeat	46	2	X.calcicola GTX0491	Simple_repeat
(GTCTTT)N#Simple_repeat	46	1	X.calcicola GTX0491	Simple_repeat
(GTTATA)N#Simple_repeat	46	1	X.calcicola GTX0491	Simple_repeat
(GTTGAGA)N#Simple_repeat	46	1	X.calcicola GTX0491	Simple_repeat
(GTTGCAA)N#Simple_repeat	46	1	X.calcicola GTX0491	Simple_repeat
(GTTGTG)N#Simple_repeat	46	1	X.calcicola GTX0491	Simple_repeat
(TAAA)N#Simple_repeat	46	2	X.calcicola GTX0491	Simple_repeat
(TAGGTAAGG)N#Simple_repeat	46	1	X.calcicola GTX0491	Simple_repeat
(TATAGTAT)N#Simple_repeat	46	1	X.calcicola GTX0491	Simple_repeat

(TCAACC)N#Simple_repeat	46	1	X.calcicola GTX0491	Simple_repeat
(TCCCGT)N#Simple_repeat	46	1	X.calcicola GTX0491	Simple_repeat
(TGCTGA)N#Simple_repeat	46	1	X.calcicola GTX0491	Simple_repeat
(TTCTTTT)N#Simple_repeat	46	1	X.calcicola GTX0491	Simple_repeat
(AGAGCA)N#Simple_repeat	45	1	X.calcicola GTX0491	Simple_repeat
(ATCACCTCT)N#Simple_repeat	45	1	X.calcicola GTX0491	Simple_repeat
(ATTACTT)N#Simple_repeat	45	1	X.calcicola GTX0491	Simple_repeat
(CAACCAA)N#Simple_repeat	45	1	X.calcicola GTX0491	Simple_repeat
(CATCCG)N#Simple_repeat	45	1	X.calcicola GTX0491	Simple_repeat
(CATCGAG)N#Simple_repeat	45	1	X.calcicola GTX0491	Simple_repeat
(CCCCACA)N#Simple_repeat	45	1	X.calcicola GTX0491	Simple_repeat
(CCGACG)N#Simple_repeat	45	1	X.calcicola GTX0491	Simple_repeat
(CCTCTATCTA)N#Simple_repeat	45	1	X.calcicola GTX0491	Simple_repeat
(CCTCTG)N#Simple_repeat	45	1	X.calcicola GTX0491	Simple_repeat
(CCTGA)N#Simple_repeat	45	1	X.calcicola GTX0491	Simple_repeat
(CCTTCAT)N#Simple_repeat	45	1	X.calcicola GTX0491	Simple_repeat
(CGCAAA)N#Simple_repeat	45	1	X.calcicola GTX0491	Simple_repeat
(CGCGGA)N#Simple_repeat	45	1	X.calcicola GTX0491	Simple_repeat
(CGTCGC)N#Simple_repeat	45	1	X.calcicola GTX0491	Simple_repeat
(GAGCGGA)N#Simple_repeat	45	1	X.calcicola GTX0491	Simple_repeat
(GAGGATGGC)N#Simple_repeat	45	1	X.calcicola GTX0491	Simple_repeat
(GATGAGG)N#Simple_repeat	45	1	X.calcicola GTX0491	Simple_repeat
(GTACCT)N#Simple_repeat	45	1	X.calcicola GTX0491	Simple_repeat
(TAATATC)N#Simple_repeat	45	1	X.calcicola GTX0491	Simple_repeat
(TAATTAT)N#Simple_repeat	45	1	X.calcicola GTX0491	Simple_repeat
(TAGTATT)N#Simple_repeat	45	1	X.calcicola GTX0491	Simple_repeat

(TATAGC)N#Simple_repeat	45	1	X.calcicola GTX0491	Simple_repeat
(TGCTGTTGC)N#Simple_repeat	45	1	X.calcicola GTX0491	Simple_repeat
(TTT)N#Simple_repeat	45	1	X.calcicola GTX0491	Simple_repeat
(TTTTTCT)N#Simple_repeat	45	1	X.calcicola GTX0491	Simple_repeat
(AAAATAA)N#Simple_repeat	44	1	X.calcicola GTX0491	Simple_repeat
(AATGCGG)N#Simple_repeat	44	1	X.calcicola GTX0491	Simple_repeat
(ACCGTC)N#Simple_repeat	44	1	X.calcicola GTX0491	Simple_repeat
(AGATAA)N#Simple_repeat	44	1	X.calcicola GTX0491	Simple_repeat
(AGCTGA)N#Simple_repeat	44	1	X.calcicola GTX0491	Simple_repeat
(AGCTTC)N#Simple_repeat	44	1	X.calcicola GTX0491	Simple_repeat
(AGTATTA)N#Simple_repeat	44	1	X.calcicola GTX0491	Simple_repeat
(AGTGTCC)N#Simple_repeat	44	1	X.calcicola GTX0491	Simple_repeat
(ATAAGTA)N#Simple_repeat	44	1	X.calcicola GTX0491	Simple_repeat
(ATAGAAT)N#Simple_repeat	44	1	X.calcicola GTX0491	Simple_repeat
(ATATAT)N#Simple_repeat	44	1	X.calcicola GTX0491	Simple_repeat
(ATCCCA)N#Simple_repeat	44	1	X.calcicola GTX0491	Simple_repeat
(ATCTGCATCT)N#Simple_repeat	44	1	X.calcicola GTX0491	Simple_repeat
(CCATAACA)N#Simple_repeat	44	1	X.calcicola GTX0491	Simple_repeat
(CCATATT)N#Simple_repeat	44	1	X.calcicola GTX0491	Simple_repeat
(CCCGACT)N#Simple_repeat	44	1	X.calcicola GTX0491	Simple_repeat
(GACACA)N#Simple_repeat	44	1	X.calcicola GTX0491	Simple_repeat
(GAGAAAG)N#Simple_repeat	44	1	X.calcicola GTX0491	Simple_repeat
(GAGTTTT)N#Simple_repeat	44	1	X.calcicola GTX0491	Simple_repeat
(GATTATAGA)N#Simple_repeat	44	1	X.calcicola GTX0491	Simple_repeat
(GGACA)N#Simple_repeat	44	1	X.calcicola GTX0491	Simple_repeat
(GGAGGTG)N#Simple_repeat	44	1	X.calcicola GTX0491	Simple_repeat

(GGTTGGA)N#Simple_repeat	44	1	X.calcicola GTX0491	Simple_repeat
(GTCAA)N#Simple_repeat	44	1	X.calcicola GTX0491	Simple_repeat
(TAGTA)N#Simple_repeat	44	1	X.calcicola GTX0491	Simple_repeat
(TAGTTA)N#Simple_repeat	44	1	X.calcicola GTX0491	Simple_repeat
(TATAGTT)N#Simple_repeat	44	1	X.calcicola GTX0491	Simple_repeat
(TCTGCC)N#Simple_repeat	44	1	X.calcicola GTX0491	Simple_repeat
(TCTGCCG)N#Simple_repeat	44	1	X.calcicola GTX0491	Simple_repeat
(TCTGGA)N#Simple_repeat	44	1	X.calcicola GTX0491	Simple_repeat
(TGTCCT)N#Simple_repeat	44	1	X.calcicola GTX0491	Simple_repeat
(AATAAC)N#Simple_repeat	43	1	X.calcicola GTX0491	Simple_repeat
(AATGAG)N#Simple_repeat	43	1	X.calcicola GTX0491	Simple_repeat
(ACCTTC)N#Simple_repeat	43	1	X.calcicola GTX0491	Simple_repeat
(AGTTACT)N#Simple_repeat	43	1	X.calcicola GTX0491	Simple_repeat
(ATGGAA)N#Simple_repeat	43	1	X.calcicola GTX0491	Simple_repeat
(ATTCGG)N#Simple_repeat	43	1	X.calcicola GTX0491	Simple_repeat
(CAACC)N#Simple_repeat	43	1	X.calcicola GTX0491	Simple_repeat
(CACTTCAT)N#Simple_repeat	43	1	X.calcicola GTX0491	Simple_repeat
(CCAATCA)N#Simple_repeat	43	1	X.calcicola GTX0491	Simple_repeat
(CCCCCG)N#Simple_repeat	43	1	X.calcicola GTX0491	Simple_repeat
(CCGCTT)N#Simple_repeat	43	1	X.calcicola GTX0491	Simple_repeat
(CGCCACCAT)N#Simple_repeat	43	1	X.calcicola GTX0491	Simple_repeat
(CTCCCTC)N#Simple_repeat	43	1	X.calcicola GTX0491	Simple_repeat
(GAGACA)N#Simple_repeat	43	1	X.calcicola GTX0491	Simple_repeat
(GAGGCA)N#Simple_repeat	43	1	X.calcicola GTX0491	Simple_repeat
(GAGGCGTTG)N#Simple_repeat	43	1	X.calcicola GTX0491	Simple_repeat
(GAGGGT)N#Simple_repeat	43	1	X.calcicola GTX0491	Simple_repeat

(GAGGTA)N#Simple_repeat	43	1	X.calcicola GTX0491	Simple_repeat
(GATGCC)N#Simple_repeat	43	1	X.calcicola GTX0491	Simple_repeat
(GCTTT)N#Simple_repeat	43	1	X.calcicola GTX0491	Simple_repeat
(GGGT)N#Simple_repeat	43	2	X.calcicola GTX0491	Simple_repeat
(GGTTCG)N#Simple_repeat	43	1	X.calcicola GTX0491	Simple_repeat
(GTATGT)N#Simple_repeat	43	1	X.calcicola GTX0491	Simple_repeat
(TAATATT)N#Simple_repeat	43	1	X.calcicola GTX0491	Simple_repeat
(TACTCT)N#Simple_repeat	43	1	X.calcicola GTX0491	Simple_repeat
(TATATTC)N#Simple_repeat	43	1	X.calcicola GTX0491	Simple_repeat
(TCATCC)N#Simple_repeat	43	1	X.calcicola GTX0491	Simple_repeat
(TCATCTT)N#Simple_repeat	43	1	X.calcicola GTX0491	Simple_repeat
(TCGTCC)N#Simple_repeat	43	1	X.calcicola GTX0491	Simple_repeat
(TCTAATA)N#Simple_repeat	43	1	X.calcicola GTX0491	Simple_repeat
(TGATTT)N#Simple_repeat	43	1	X.calcicola GTX0491	Simple_repeat
(TTATATT)N#Simple_repeat	43	1	X.calcicola GTX0491	Simple_repeat
(TTATTTA)N#Simple_repeat	43	1	X.calcicola GTX0491	Simple_repeat
(TTCTTTA)N#Simple_repeat	43	1	X.calcicola GTX0491	Simple_repeat
(TTGA)N#Simple_repeat	43	2	X.calcicola GTX0491	Simple_repeat
(TTTGTA)N#Simple_repeat	43	1	X.calcicola GTX0491	Simple_repeat
(AAATCCG)N#Simple_repeat	42	1	X.calcicola GTX0491	Simple_repeat
(AATCC)N#Simple_repeat	42	1	X.calcicola GTX0491	Simple_repeat
(ACAAG)N#Simple_repeat	42	1	X.calcicola GTX0491	Simple_repeat
(ACACA)N#Simple_repeat	42	1	X.calcicola GTX0491	Simple_repeat
(ACGAT)N#Simple_repeat	42	1	X.calcicola GTX0491	Simple_repeat
(AGATAT)N#Simple_repeat	42	1	X.calcicola GTX0491	Simple_repeat
(AGATGA)N#Simple_repeat	42	1	X.calcicola GTX0491	Simple_repeat

(AGCAATC)N#Simple_repeat	42	1	X.calcicola GTX0491	Simple_repeat
(AGGAAG)N#Simple_repeat	42	1	X.calcicola GTX0491	Simple_repeat
(AGGCAGA)N#Simple_repeat	42	1	X.calcicola GTX0491	Simple_repeat
(AGTATAT)N#Simple_repeat	42	1	X.calcicola GTX0491	Simple_repeat
(ATATTCT)N#Simple_repeat	42	1	X.calcicola GTX0491	Simple_repeat
(ATGCTG)N#Simple_repeat	42	1	X.calcicola GTX0491	Simple_repeat
(CCAACCC)N#Simple_repeat	42	1	X.calcicola GTX0491	Simple_repeat
(CCCCCTA)N#Simple_repeat	42	1	X.calcicola GTX0491	Simple_repeat
(CGGAGG)N#Simple_repeat	42	1	X.calcicola GTX0491	Simple_repeat
(CTATATTT)N#Simple_repeat	42	1	X.calcicola GTX0491	Simple_repeat
(CTCCC)N#Simple_repeat	42	1	X.calcicola GTX0491	Simple_repeat
(CTGATT)N#Simple_repeat	42	1	X.calcicola GTX0491	Simple_repeat
(CTGCTG)N#Simple_repeat	42	1	X.calcicola GTX0491	Simple_repeat
(GAGCCA)N#Simple_repeat	42	1	X.calcicola GTX0491	Simple_repeat
(GAGCGG)N#Simple_repeat	42	1	X.calcicola GTX0491	Simple_repeat
(GAGGAGGGA)N#Simple_repeat	42	1	X.calcicola GTX0491	Simple_repeat
(GAGGTTA)N#Simple_repeat	42	1	X.calcicola GTX0491	Simple_repeat
(GAGTGA)N#Simple_repeat	42	1	X.calcicola GTX0491	Simple_repeat
(GCATCA)N#Simple_repeat	42	1	X.calcicola GTX0491	Simple_repeat
(GCCAAC)N#Simple_repeat	42	1	X.calcicola GTX0491	Simple_repeat
(GGCATC)N#Simple_repeat	42	1	X.calcicola GTX0491	Simple_repeat
(GGCGGC)N#Simple_repeat	42	1	X.calcicola GTX0491	Simple_repeat
(GTTGAG)N#Simple_repeat	42	1	X.calcicola GTX0491	Simple_repeat
(TAGTAC)N#Simple_repeat	42	1	X.calcicola GTX0491	Simple_repeat
(TCTTGTGCT)N#Simple_repeat	42	1	X.calcicola GTX0491	Simple_repeat
(TGAGG)N#Simple_repeat	42	1	X.calcicola GTX0491	Simple_repeat

(TGCAGG)N#Simple_repeat	42	1	X.calcicola GTX0491	Simple_repeat
(TGGAC)N#Simple_repeat	42	1	X.calcicola GTX0491	Simple_repeat
(TGTCCAGTCC)N#Simple_repeat	42	1	X.calcicola GTX0491	Simple_repeat
(TTACG)N#Simple_repeat	42	1	X.calcicola GTX0491	Simple_repeat
(TTGTTGC)N#Simple_repeat	42	1	X.calcicola GTX0491	Simple_repeat
(TTTGAT)N#Simple_repeat	42	1	X.calcicola GTX0491	Simple_repeat
(TTTTAA)N#Simple_repeat	42	1	X.calcicola GTX0491	Simple_repeat
(TTTTTAC)N#Simple_repeat	42	1	X.calcicola GTX0491	Simple_repeat
(AATTAGT)N#Simple_repeat	41	1	X.calcicola GTX0491	Simple_repeat
(AATTTTT)N#Simple_repeat	41	1	X.calcicola GTX0491	Simple_repeat
(AGGAGC)N#Simple_repeat	41	1	X.calcicola GTX0491	Simple_repeat
(AGTTT)N#Simple_repeat	41	1	X.calcicola GTX0491	Simple_repeat
(ATATATA)N#Simple_repeat	41	1	X.calcicola GTX0491	Simple_repeat
(ATATTT)N#Simple_repeat	41	1	X.calcicola GTX0491	Simple_repeat
(ATCCAT)N#Simple_repeat	41	1	X.calcicola GTX0491	Simple_repeat
(CATCCCC)N#Simple_repeat	41	1	X.calcicola GTX0491	Simple_repeat
(CCATA)N#Simple_repeat	41	1	X.calcicola GTX0491	Simple_repeat
(CCATC)N#Simple_repeat	41	1	X.calcicola GTX0491	Simple_repeat
(CCCGCG)N#Simple_repeat	41	1	X.calcicola GTX0491	Simple_repeat
(CTAACCC)N#Simple_repeat	41	1	X.calcicola GTX0491	Simple_repeat
(CTCCATA)N#Simple_repeat	41	1	X.calcicola GTX0491	Simple_repeat
(CTTACC)N#Simple_repeat	41	1	X.calcicola GTX0491	Simple_repeat
(CTTTCT)N#Simple_repeat	41	1	X.calcicola GTX0491	Simple_repeat
(GAGGAAC)N#Simple_repeat	41	1	X.calcicola GTX0491	Simple_repeat
(GATTCG)N#Simple_repeat	41	1	X.calcicola GTX0491	Simple_repeat
(GATTTC)N#Simple_repeat	41	1	X.calcicola GTX0491	Simple_repeat

(GGACCA)N#Simple_repeat	41	1	X.calcicola GTX0491	Simple_repeat
(GGACGT)N#Simple_repeat	41	1	X.calcicola GTX0491	Simple_repeat
(GGGAAGAG)N#Simple_repeat	41	1	X.calcicola GTX0491	Simple_repeat
(GGTGT)N#Simple_repeat	41	1	X.calcicola GTX0491	Simple_repeat
(GTCATC)N#Simple_repeat	41	1	X.calcicola GTX0491	Simple_repeat
(GTTATT)N#Simple_repeat	41	1	X.calcicola GTX0491	Simple_repeat
(TATTATATAT)N#Simple_repeat	41	1	X.calcicola GTX0491	Simple_repeat
(TTATATAA)N#Simple_repeat	41	1	X.calcicola GTX0491	Simple_repeat
(AAAACC)N#Simple_repeat	40	1	X.calcicola GTX0491	Simple_repeat
(AATAATA)N#Simple_repeat	40	1	X.calcicola GTX0491	Simple_repeat
(AATATAG)N#Simple_repeat	40	1	X.calcicola GTX0491	Simple_repeat
(AGAGGT)N#Simple_repeat	40	1	X.calcicola GTX0491	Simple_repeat
(AGCTTCG)N#Simple_repeat	40	1	X.calcicola GTX0491	Simple_repeat
(AGGGCA)N#Simple_repeat	40	1	X.calcicola GTX0491	Simple_repeat
(ATACTTT)N#Simple_repeat	40	1	X.calcicola GTX0491	Simple_repeat
(ATAGAG)N#Simple_repeat	40	1	X.calcicola GTX0491	Simple_repeat
(ATAGCC)N#Simple_repeat	40	1	X.calcicola GTX0491	Simple_repeat
(ATCGAA)N#Simple_repeat	40	1	X.calcicola GTX0491	Simple_repeat
(ATCGC)N#Simple_repeat	40	1	X.calcicola GTX0491	Simple_repeat
(CATTCTT)N#Simple_repeat	40	1	X.calcicola GTX0491	Simple_repeat
(CCCACTA)N#Simple_repeat	40	1	X.calcicola GTX0491	Simple_repeat
(CCCATTC)N#Simple_repeat	40	1	X.calcicola GTX0491	Simple_repeat
(CCCCATC)N#Simple_repeat	40	1	X.calcicola GTX0491	Simple_repeat
(CCGGCC)N#Simple_repeat	40	1	X.calcicola GTX0491	Simple_repeat
(CGACAT)N#Simple_repeat	40	1	X.calcicola GTX0491	Simple_repeat
(CTACGTATAT)N#Simple_repeat	40	1	X.calcicola GTX0491	Simple_repeat

(CTTATA)N#Simple_repeat	40	1	X.calcicola GTX0491	Simple_repeat
(CTTCCCCTT)N#Simple_repeat	40	1	X.calcicola GTX0491	Simple_repeat
(GAGAGGT)N#Simple_repeat	40	1	X.calcicola GTX0491	Simple_repeat
(GAGGACG)N#Simple_repeat	40	1	X.calcicola GTX0491	Simple_repeat
(GATGGTGAT)N#Simple_repeat	40	1	X.calcicola GTX0491	Simple_repeat
(GCAT)N#Simple_repeat	40	1	X.calcicola GTX0491	Simple_repeat
(GGAGGGTGA)N#Simple_repeat	40	1	X.calcicola GTX0491	Simple_repeat
(GTCAT)N#Simple_repeat	40	1	X.calcicola GTX0491	Simple_repeat
(GTGGCGA)N#Simple_repeat	40	1	X.calcicola GTX0491	Simple_repeat
(GTGGTT)N#Simple_repeat	40	1	X.calcicola GTX0491	Simple_repeat
(TAATC)N#Simple_repeat	40	1	X.calcicola GTX0491	Simple_repeat
(TAGAA)N#Simple_repeat	40	1	X.calcicola GTX0491	Simple_repeat
(TAGAGAC)N#Simple_repeat	40	1	X.calcicola GTX0491	Simple_repeat
(TATTAGC)N#Simple_repeat	40	1	X.calcicola GTX0491	Simple_repeat
(TCGGCT)N#Simple_repeat	40	1	X.calcicola GTX0491	Simple_repeat
(TCTGGT)N#Simple_repeat	40	1	X.calcicola GTX0491	Simple_repeat
(TGCTGT)N#Simple_repeat	40	1	X.calcicola GTX0491	Simple_repeat
(TGGAGA)N#Simple_repeat	40	1	X.calcicola GTX0491	Simple_repeat
(TGGTAA)N#Simple_repeat	40	1	X.calcicola GTX0491	Simple_repeat
(TTATAGA)N#Simple_repeat	40	1	X.calcicola GTX0491	Simple_repeat
(TTCATC)N#Simple_repeat	40	1	X.calcicola GTX0491	Simple_repeat
(TTCGTGG)N#Simple_repeat	40	1	X.calcicola GTX0491	Simple_repeat
(TTTCCCA)N#Simple_repeat	40	1	X.calcicola GTX0491	Simple_repeat
(AACAGC)N#Simple_repeat	39	1	X.calcicola GTX0491	Simple_repeat
(AACGCTCC)N#Simple_repeat	39	1	X.calcicola GTX0491	Simple_repeat
(ACCAGCT)N#Simple_repeat	39	1	X.calcicola GTX0491	Simple_repeat

(ACCCTCA)N#Simple_repeat	39	1	X.calcicola GTX0491	Simple_repeat
(ACCTCC)N#Simple_repeat	39	1	X.calcicola GTX0491	Simple_repeat
(AGACGAG)N#Simple_repeat	39	1	X.calcicola GTX0491	Simple_repeat
(AGGACG)N#Simple_repeat	39	1	X.calcicola GTX0491	Simple_repeat
(AGGATTG)N#Simple_repeat	39	1	X.calcicola GTX0491	Simple_repeat
(ATAACGAAG)N#Simple_repeat	39	1	X.calcicola GTX0491	Simple_repeat
(ATGATG)N#Simple_repeat	39	1	X.calcicola GTX0491	Simple_repeat
(ATTATGT)N#Simple_repeat	39	1	X.calcicola GTX0491	Simple_repeat
(CAAGAA)N#Simple_repeat	39	1	X.calcicola GTX0491	Simple_repeat
(CCAGCAA)N#Simple_repeat	39	1	X.calcicola GTX0491	Simple_repeat
(CCAGCG)N#Simple_repeat	39	1	X.calcicola GTX0491	Simple_repeat
(CCATAC)N#Simple_repeat	39	1	X.calcicola GTX0491	Simple_repeat
(CCCCCTAT)N#Simple_repeat	39	1	X.calcicola GTX0491	Simple_repeat
(CCTACTA)N#Simple_repeat	39	1	X.calcicola GTX0491	Simple_repeat
(CCTCAAT)N#Simple_repeat	39	1	X.calcicola GTX0491	Simple_repeat
(CCTCCTCCA)N#Simple_repeat	39	1	X.calcicola GTX0491	Simple_repeat
(CCTCGA)N#Simple_repeat	39	1	X.calcicola GTX0491	Simple_repeat
(CGCCTC)N#Simple_repeat	39	1	X.calcicola GTX0491	Simple_repeat
(CGGGGCAAC)N#Simple_repeat	39	1	X.calcicola GTX0491	Simple_repeat
(CTAATATAT)N#Simple_repeat	39	1	X.calcicola GTX0491	Simple_repeat
(CTCCGA)N#Simple_repeat	39	1	X.calcicola GTX0491	Simple_repeat
(CTTCCAC)N#Simple_repeat	39	1	X.calcicola GTX0491	Simple_repeat
(GACCAG)N#Simple_repeat	39	1	X.calcicola GTX0491	Simple_repeat
(GAGGTTG)N#Simple_repeat	39	1	X.calcicola GTX0491	Simple_repeat
(GCTGT)N#Simple_repeat	39	1	X.calcicola GTX0491	Simple_repeat
(GGATTTT)N#Simple_repeat	39	1	X.calcicola GTX0491	Simple_repeat

(GGCAGGAG)N#Simple_repeat	39	1	X.calcicola GTX0491	Simple_repeat
(GGGTGCGT)N#Simple_repeat	39	1	X.calcicola GTX0491	Simple_repeat
(GTATG)N#Simple_repeat	39	1	X.calcicola GTX0491	Simple_repeat
(GTCAC)N#Simple_repeat	39	1	X.calcicola GTX0491	Simple_repeat
(TACCTT)N#Simple_repeat	39	1	X.calcicola GTX0491	Simple_repeat
(TAGAATTT)N#Simple_repeat	39	1	X.calcicola GTX0491	Simple_repeat
(TAGAC)N#Simple_repeat	39	1	X.calcicola GTX0491	Simple_repeat
(TCTCGCC)N#Simple_repeat	39	1	X.calcicola GTX0491	Simple_repeat
(TCTTTTG)N#Simple_repeat	39	1	X.calcicola GTX0491	Simple_repeat
(TGCCGC)N#Simple_repeat	39	1	X.calcicola GTX0491	Simple_repeat
(TGTGC)N#Simple_repeat	39	1	X.calcicola GTX0491	Simple_repeat
(TTATGCA)N#Simple_repeat	39	1	X.calcicola GTX0491	Simple_repeat
(TTGGTT)N#Simple_repeat	39	1	X.calcicola GTX0491	Simple_repeat
(TTTGTTT)N#Simple_repeat	39	1	X.calcicola GTX0491	Simple_repeat
(AAGGA)N#Simple_repeat	38	1	X.calcicola GTX0491	Simple_repeat
(AATCTTG)N#Simple_repeat	38	1	X.calcicola GTX0491	Simple_repeat
(AATTCAC)N#Simple_repeat	38	1	X.calcicola GTX0491	Simple_repeat
(ACACCCC)N#Simple_repeat	38	1	X.calcicola GTX0491	Simple_repeat
(ACCAGT)N#Simple_repeat	38	1	X.calcicola GTX0491	Simple_repeat
(AGCTCG)N#Simple_repeat	38	1	X.calcicola GTX0491	Simple_repeat
(ATCCAC)N#Simple_repeat	38	1	X.calcicola GTX0491	Simple_repeat
(ATGGCC)N#Simple_repeat	38	1	X.calcicola GTX0491	Simple_repeat
(CACAT)N#Simple_repeat	38	1	X.calcicola GTX0491	Simple_repeat
(CACATG)N#Simple_repeat	38	1	X.calcicola GTX0491	Simple_repeat
(CAGAAT)N#Simple_repeat	38	1	X.calcicola GTX0491	Simple_repeat
(CATAT)N#Simple_repeat	38	1	X.calcicola GTX0491	Simple_repeat

(CATCGG)N#Simple_repeat	38	1	X.calcicola GTX0491	Simple_repeat
(CATGGA)N#Simple_repeat	38	1	X.calcicola GTX0491	Simple_repeat
(CCAGT)N#Simple_repeat	38	1	X.calcicola GTX0491	Simple_repeat
(CCCGCCTA)N#Simple_repeat	38	1	X.calcicola GTX0491	Simple_repeat
(CCGATCCA)N#Simple_repeat	38	1	X.calcicola GTX0491	Simple_repeat
(CTATATA)N#Simple_repeat	38	1	X.calcicola GTX0491	Simple_repeat
(CTCCCCA)N#Simple_repeat	38	1	X.calcicola GTX0491	Simple_repeat
(CTTTTAG)N#Simple_repeat	38	1	X.calcicola GTX0491	Simple_repeat
(GAAGGGG)N#Simple_repeat	38	1	X.calcicola GTX0491	Simple_repeat
(GAATG)N#Simple_repeat	38	1	X.calcicola GTX0491	Simple_repeat
(GAGCAA)N#Simple_repeat	38	1	X.calcicola GTX0491	Simple_repeat
(GATACCCTA)N#Simple_repeat	38	1	X.calcicola GTX0491	Simple_repeat
(GCCAAT)N#Simple_repeat	38	1	X.calcicola GTX0491	Simple_repeat
(GGTATT)N#Simple_repeat	38	1	X.calcicola GTX0491	Simple_repeat
(GTCCACG)N#Simple_repeat	38	1	X.calcicola GTX0491	Simple_repeat
(GTCGTC)N#Simple_repeat	38	1	X.calcicola GTX0491	Simple_repeat
(GTCT)N#Simple_repeat	38	1	X.calcicola GTX0491	Simple_repeat
(GTGTTG)N#Simple_repeat	38	1	X.calcicola GTX0491	Simple_repeat
(TATCCTCAC)N#Simple_repeat	38	1	X.calcicola GTX0491	Simple_repeat
(TATCG)N#Simple_repeat	38	1	X.calcicola GTX0491	Simple_repeat
(TCCCAGC)N#Simple_repeat	38	1	X.calcicola GTX0491	Simple_repeat
(TCGATC)N#Simple_repeat	38	1	X.calcicola GTX0491	Simple_repeat
(TGATGG)N#Simple_repeat	38	1	X.calcicola GTX0491	Simple_repeat
(TGGATA)N#Simple_repeat	38	1	X.calcicola GTX0491	Simple_repeat
(TGTGAG)N#Simple_repeat	38	1	X.calcicola GTX0491	Simple_repeat
(TTCCTGC)N#Simple_repeat	38	1	X.calcicola GTX0491	Simple_repeat

(TTCTCT)N#Simple_repeat	38	1	X.calcicola GTX0491	Simple_repeat
(TTGAA)N#Simple_repeat	38	1	X.calcicola GTX0491	Simple_repeat
(TTGGTGG)N#Simple_repeat	38	1	X.calcicola GTX0491	Simple_repeat
(AAATTC)N#Simple_repeat	37	1	X.calcicola GTX0491	Simple_repeat
(AAGCA)N#Simple_repeat	37	1	X.calcicola GTX0491	Simple_repeat
(AAGCGG)N#Simple_repeat	37	1	X.calcicola GTX0491	Simple_repeat
(AAGGCA)N#Simple_repeat	37	1	X.calcicola GTX0491	Simple_repeat
(AATCCCC)N#Simple_repeat	37	1	X.calcicola GTX0491	Simple_repeat
(AGATC)N#Simple_repeat	37	1	X.calcicola GTX0491	Simple_repeat
(AGGAG)N#Simple_repeat	37	1	X.calcicola GTX0491	Simple_repeat
(ATCCTAC)N#Simple_repeat	37	1	X.calcicola GTX0491	Simple_repeat
(ATGATA)N#Simple_repeat	37	1	X.calcicola GTX0491	Simple_repeat
(ATTGCCTC)N#Simple_repeat	37	1	X.calcicola GTX0491	Simple_repeat
(CACCCTC)N#Simple_repeat	37	1	X.calcicola GTX0491	Simple_repeat
(CCACGA)N#Simple_repeat	37	1	X.calcicola GTX0491	Simple_repeat
(CCCACCC)N#Simple_repeat	37	1	X.calcicola GTX0491	Simple_repeat
(CTATCAT)N#Simple_repeat	37	1	X.calcicola GTX0491	Simple_repeat
(GACAGCCG)N#Simple_repeat	37	1	X.calcicola GTX0491	Simple_repeat
(GAGAT)N#Simple_repeat	37	1	X.calcicola GTX0491	Simple_repeat
(GATGAGC)N#Simple_repeat	37	1	X.calcicola GTX0491	Simple_repeat
(GCCGAGA)N#Simple_repeat	37	1	X.calcicola GTX0491	Simple_repeat
(GGACAGAAG)N#Simple_repeat	37	1	X.calcicola GTX0491	Simple_repeat
(GGAGATG)N#Simple_repeat	37	1	X.calcicola GTX0491	Simple_repeat
(GTAGCTA)N#Simple_repeat	37	1	X.calcicola GTX0491	Simple_repeat
(GTCGC)N#Simple_repeat	37	1	X.calcicola GTX0491	Simple_repeat
(GTGGAGG)N#Simple_repeat	37	1	X.calcicola GTX0491	Simple_repeat

(GTTCTC)N#Simple_repeat	37	1	X.calcicola GTX0491	Simple_repeat
(TAGATT)N#Simple_repeat	37	1	X.calcicola GTX0491	Simple_repeat
(TAGCTA)N#Simple_repeat	37	1	X.calcicola GTX0491	Simple_repeat
(TATAAC)N#Simple_repeat	37	1	X.calcicola GTX0491	Simple_repeat
(TCATG)N#Simple_repeat	37	1	X.calcicola GTX0491	Simple_repeat
(TCCAACC)N#Simple_repeat	37	1	X.calcicola GTX0491	Simple_repeat
(TCCCAT)N#Simple_repeat	37	1	X.calcicola GTX0491	Simple_repeat
(TCCCTGG)N#Simple_repeat	37	1	X.calcicola GTX0491	Simple_repeat
(TCGATA)N#Simple_repeat	37	1	X.calcicola GTX0491	Simple_repeat
(TCGCTC)N#Simple_repeat	37	1	X.calcicola GTX0491	Simple_repeat
(TCTCCCA)N#Simple_repeat	37	1	X.calcicola GTX0491	Simple_repeat
(TGATGAA)N#Simple_repeat	37	1	X.calcicola GTX0491	Simple_repeat
(TGGCTC)N#Simple_repeat	37	1	X.calcicola GTX0491	Simple_repeat
(TGGTA)N#Simple_repeat	37	1	X.calcicola GTX0491	Simple_repeat
(TTATT)N#Simple_repeat	37	1	X.calcicola GTX0491	Simple_repeat
(TTCCTTG)N#Simple_repeat	37	1	X.calcicola GTX0491	Simple_repeat
(TTTT)N#Simple_repeat	37	1	X.calcicola GTX0491	Simple_repeat
(TTTTCA)N#Simple_repeat	37	1	X.calcicola GTX0491	Simple_repeat
(TTTTCC)N#Simple_repeat	37	1	X.calcicola GTX0491	Simple_repeat
(AACAC)N#Simple_repeat	36	1	X.calcicola GTX0491	Simple_repeat
(AATTTAA)N#Simple_repeat	36	1	X.calcicola GTX0491	Simple_repeat
(ACCCT)N#Simple_repeat	36	1	X.calcicola GTX0491	Simple_repeat
(ACGAAG)N#Simple_repeat	36	1	X.calcicola GTX0491	Simple_repeat
(AGGCGG)N#Simple_repeat	36	1	X.calcicola GTX0491	Simple_repeat
(AGTCGCA)N#Simple_repeat	36	1	X.calcicola GTX0491	Simple_repeat
(ATATCTA)N#Simple_repeat	36	1	X.calcicola GTX0491	Simple_repeat

(ATATGT)N#Simple_repeat	36	1	X.calcicola GTX0491	Simple_repeat
(ATCAGC)N#Simple_repeat	36	1	X.calcicola GTX0491	Simple_repeat
(ATGGG)N#Simple_repeat	36	1	X.calcicola GTX0491	Simple_repeat
(ATTAATAAA)N#Simple_repeat	36	1	X.calcicola GTX0491	Simple_repeat
(CAAGGC)N#Simple_repeat	36	1	X.calcicola GTX0491	Simple_repeat
(CACCATCA)N#Simple_repeat	36	1	X.calcicola GTX0491	Simple_repeat
(CACCTTC)N#Simple_repeat	36	1	X.calcicola GTX0491	Simple_repeat
(CAGGAC)N#Simple_repeat	36	1	X.calcicola GTX0491	Simple_repeat
(CCAGAA)N#Simple_repeat	36	1	X.calcicola GTX0491	Simple_repeat
(CCCCTTC)N#Simple_repeat	36	1	X.calcicola GTX0491	Simple_repeat
(CCTCGCT)N#Simple_repeat	36	1	X.calcicola GTX0491	Simple_repeat
(CGCTGC)N#Simple_repeat	36	1	X.calcicola GTX0491	Simple_repeat
(CTATAA)N#Simple_repeat	36	1	X.calcicola GTX0491	Simple_repeat
(CTTATC)N#Simple_repeat	36	1	X.calcicola GTX0491	Simple_repeat
(GACGAC)N#Simple_repeat	36	1	X.calcicola GTX0491	Simple_repeat
(GAGGCGG)N#Simple_repeat	36	1	X.calcicola GTX0491	Simple_repeat
(GAGGTT)N#Simple_repeat	36	1	X.calcicola GTX0491	Simple_repeat
(GCGATG)N#Simple_repeat	36	1	X.calcicola GTX0491	Simple_repeat
(GCGGAGG)N#Simple_repeat	36	1	X.calcicola GTX0491	Simple_repeat
(GCTTCGA)N#Simple_repeat	36	1	X.calcicola GTX0491	Simple_repeat
(GGAACGA)N#Simple_repeat	36	1	X.calcicola GTX0491	Simple_repeat
(GGCTGCT)N#Simple_repeat	36	1	X.calcicola GTX0491	Simple_repeat
(GGTGTAG)N#Simple_repeat	36	1	X.calcicola GTX0491	Simple_repeat
(GGTTGG)N#Simple_repeat	36	1	X.calcicola GTX0491	Simple_repeat
(GTACGG)N#Simple_repeat	36	1	X.calcicola GTX0491	Simple_repeat
(TAATAG)N#Simple_repeat	36	1	X.calcicola GTX0491	Simple_repeat

(TAGAGAA)N#Simple_repeat	36	1	X.calcicola GTX0491	Simple_repeat
(TATAGTA)N#Simple_repeat	36	1	X.calcicola GTX0491	Simple_repeat
(TCAGGGGC)N#Simple_repeat	36	1	X.calcicola GTX0491	Simple_repeat
(TCATCG)N#Simple_repeat	36	2	X.calcicola GTX0491	Simple_repeat
(TCTAAC)N#Simple_repeat	36	1	X.calcicola GTX0491	Simple_repeat
(TCTGGAT)N#Simple_repeat	36	1	X.calcicola GTX0491	Simple_repeat
(TGCAAGC)N#Simple_repeat	36	1	X.calcicola GTX0491	Simple_repeat
(TGCGAG)N#Simple_repeat	36	1	X.calcicola GTX0491	Simple_repeat
(TGCTCA)N#Simple_repeat	36	1	X.calcicola GTX0491	Simple_repeat
(TGGACC)N#Simple_repeat	36	1	X.calcicola GTX0491	Simple_repeat
(TGTCG)N#Simple_repeat	36	1	X.calcicola GTX0491	Simple_repeat
(TTACT)N#Simple_repeat	36	1	X.calcicola GTX0491	Simple_repeat
(TTATCG)N#Simple_repeat	36	1	X.calcicola GTX0491	Simple_repeat
(TTGTGA)N#Simple_repeat	36	1	X.calcicola GTX0491	Simple_repeat
(AAGGTA)N#Simple_repeat	35	1	X.calcicola GTX0491	Simple_repeat
(AATATAA)N#Simple_repeat	35	1	X.calcicola GTX0491	Simple_repeat
(ACAAGAA)N#Simple_repeat	35	1	X.calcicola GTX0491	Simple_repeat
(ACCCACG)N#Simple_repeat	35	1	X.calcicola GTX0491	Simple_repeat
(ACGAAT)N#Simple_repeat	35	1	X.calcicola GTX0491	Simple_repeat
(ACTAGT)N#Simple_repeat	35	1	X.calcicola GTX0491	Simple_repeat
(AGATG)N#Simple_repeat	35	1	X.calcicola GTX0491	Simple_repeat
(AGCACC)N#Simple_repeat	35	1	X.calcicola GTX0491	Simple_repeat
(AGTAA)N#Simple_repeat	35	1	X.calcicola GTX0491	Simple_repeat
(AGTGGTC)N#Simple_repeat	35	1	X.calcicola GTX0491	Simple_repeat
(ATAATT)N#Simple_repeat	35	1	X.calcicola GTX0491	Simple_repeat
(ATACAAC)N#Simple_repeat	35	1	X.calcicola GTX0491	Simple_repeat

(ATCACA)N#Simple_repeat	35	1	X.calcicola GTX0491	Simple_repeat
(ATCTATA)N#Simple_repeat	35	1	X.calcicola GTX0491	Simple_repeat
(ATGGATG)N#Simple_repeat	35	1	X.calcicola GTX0491	Simple_repeat
(ATTGA)N#Simple_repeat	35	1	X.calcicola GTX0491	Simple_repeat
(CAACCGC)N#Simple_repeat	35	1	X.calcicola GTX0491	Simple_repeat
(CAGACG)N#Simple_repeat	35	1	X.calcicola GTX0491	Simple_repeat
(CATCCAC)N#Simple_repeat	35	1	X.calcicola GTX0491	Simple_repeat
(CATCCCT)N#Simple_repeat	35	1	X.calcicola GTX0491	Simple_repeat
(CATGA)N#Simple_repeat	35	1	X.calcicola GTX0491	Simple_repeat
(CCAGAG)N#Simple_repeat	35	1	X.calcicola GTX0491	Simple_repeat
(CCATT)N#Simple_repeat	35	1	X.calcicola GTX0491	Simple_repeat
(CCCAG)N#Simple_repeat	35	1	X.calcicola GTX0491	Simple_repeat
(CCGGAG)N#Simple_repeat	35	1	X.calcicola GTX0491	Simple_repeat
(CCGTACT)N#Simple_repeat	35	1	X.calcicola GTX0491	Simple_repeat
(CCGTCC)N#Simple_repeat	35	1	X.calcicola GTX0491	Simple_repeat
(CCGTCCT)N#Simple_repeat	35	1	X.calcicola GTX0491	Simple_repeat
(CCTCCTG)N#Simple_repeat	35	1	X.calcicola GTX0491	Simple_repeat
(CCTCTTT)N#Simple_repeat	35	1	X.calcicola GTX0491	Simple_repeat
(CTTCACC)N#Simple_repeat	35	1	X.calcicola GTX0491	Simple_repeat
(GATACTTC)N#Simple_repeat	35	1	X.calcicola GTX0491	Simple_repeat
(GATGAT)N#Simple_repeat	35	1	X.calcicola GTX0491	Simple_repeat
(GCCGAA)N#Simple_repeat	35	1	X.calcicola GTX0491	Simple_repeat
(GCTCGT)N#Simple_repeat	35	1	X.calcicola GTX0491	Simple_repeat
(GCTGAAA)N#Simple_repeat	35	1	X.calcicola GTX0491	Simple_repeat
(GTGTA)N#Simple_repeat	35	1	X.calcicola GTX0491	Simple_repeat
(GTTAAT)N#Simple_repeat	35	1	X.calcicola GTX0491	Simple_repeat

(GTTGCG)N#Simple_repeat	35	1	X.calcicola GTX0491	Simple_repeat
(GTTTA)N#Simple_repeat	35	1	X.calcicola GTX0491	Simple_repeat
(TAGATAT)N#Simple_repeat	35	1	X.calcicola GTX0491	Simple_repeat
(TATAAG)N#Simple_repeat	35	1	X.calcicola GTX0491	Simple_repeat
(TCACGT)N#Simple_repeat	35	1	X.calcicola GTX0491	Simple_repeat
(TCCATTC)N#Simple_repeat	35	1	X.calcicola GTX0491	Simple_repeat
(TCCTAT)N#Simple_repeat	35	1	X.calcicola GTX0491	Simple_repeat
(TCTAT)N#Simple_repeat	35	1	X.calcicola GTX0491	Simple_repeat
(TCTTCT)N#Simple_repeat	35	1	X.calcicola GTX0491	Simple_repeat
(TGTGAA)N#Simple_repeat	35	1	X.calcicola GTX0491	Simple_repeat
(TTCACC)N#Simple_repeat	35	1	X.calcicola GTX0491	Simple_repeat
(TTCGGAG)N#Simple_repeat	35	1	X.calcicola GTX0491	Simple_repeat
(TTGTCGA)N#Simple_repeat	35	1	X.calcicola GTX0491	Simple_repeat
(TTGTTC)N#Simple_repeat	35	1	X.calcicola GTX0491	Simple_repeat
(TTTGCT)N#Simple_repeat	35	1	X.calcicola GTX0491	Simple_repeat
(AAAACA)N#Simple_repeat	34	1	X.calcicola GTX0491	Simple_repeat
(AAGGTC)N#Simple_repeat	34	1	X.calcicola GTX0491	Simple_repeat
(ACAAGG)N#Simple_repeat	34	1	X.calcicola GTX0491	Simple_repeat
(ACATC)N#Simple_repeat	34	1	X.calcicola GTX0491	Simple_repeat
(ACCTCCA)N#Simple_repeat	34	1	X.calcicola GTX0491	Simple_repeat
(ACTGCG)N#Simple_repeat	34	1	X.calcicola GTX0491	Simple_repeat
(ATGTG)N#Simple_repeat	34	1	X.calcicola GTX0491	Simple_repeat
(CAACCT)N#Simple_repeat	34	1	X.calcicola GTX0491	Simple_repeat
(CATCA)N#Simple_repeat	34	1	X.calcicola GTX0491	Simple_repeat
(CCTGCCT)N#Simple_repeat	34	1	X.calcicola GTX0491	Simple_repeat
(CGTACTC)N#Simple_repeat	34	1	X.calcicola GTX0491	Simple_repeat

(CGTGAG)N#Simple_repeat	34	1	X.calcicola GTX0491	Simple_repeat
(CTCATT)N#Simple_repeat	34	1	X.calcicola GTX0491	Simple_repeat
(CTTGCT)N#Simple_repeat	34	1	X.calcicola GTX0491	Simple_repeat
(CTTTCTTG)N#Simple_repeat	34	1	X.calcicola GTX0491	Simple_repeat
(GAAAAGG)N#Simple_repeat	34	1	X.calcicola GTX0491	Simple_repeat
(GAATT)N#Simple_repeat	34	1	X.calcicola GTX0491	Simple_repeat
(GAGAACGG)N#Simple_repeat	34	1	X.calcicola GTX0491	Simple_repeat
(GAGCTTG)N#Simple_repeat	34	1	X.calcicola GTX0491	Simple_repeat
(GCGGA)N#Simple_repeat	34	1	X.calcicola GTX0491	Simple_repeat
(GGCAC)N#Simple_repeat	34	1	X.calcicola GTX0491	Simple_repeat
(TACTA)N#Simple_repeat	34	1	X.calcicola GTX0491	Simple_repeat
(TATTAAT)N#Simple_repeat	34	1	X.calcicola GTX0491	Simple_repeat
(TCCTG)N#Simple_repeat	34	1	X.calcicola GTX0491	Simple_repeat
(TCGCAC)N#Simple_repeat	34	1	X.calcicola GTX0491	Simple_repeat
(TCTACTA)N#Simple_repeat	34	1	X.calcicola GTX0491	Simple_repeat
(TCTCCA)N#Simple_repeat	34	1	X.calcicola GTX0491	Simple_repeat
(TCTCGA)N#Simple_repeat	34	1	X.calcicola GTX0491	Simple_repeat
(TGAAGG)N#Simple_repeat	34	1	X.calcicola GTX0491	Simple_repeat
(TGATGA)N#Simple_repeat	34	1	X.calcicola GTX0491	Simple_repeat
(TGGGAG)N#Simple_repeat	34	1	X.calcicola GTX0491	Simple_repeat
(TTAAAA)N#Simple_repeat	34	1	X.calcicola GTX0491	Simple_repeat
(TTAATTA)N#Simple_repeat	34	1	X.calcicola GTX0491	Simple_repeat
(TTACTC)N#Simple_repeat	34	1	X.calcicola GTX0491	Simple_repeat
(TTGCTCT)N#Simple_repeat	34	1	X.calcicola GTX0491	Simple_repeat
(TTGGCAT)N#Simple_repeat	34	1	X.calcicola GTX0491	Simple_repeat
(TTTGCC)N#Simple_repeat	34	1	X.calcicola GTX0491	Simple_repeat

(AAAAAG)N#Simple_repeat	33	1	X.calcicola GTX0491	Simple_repeat
(AACAGAT)N#Simple_repeat	33	1	X.calcicola GTX0491	Simple_repeat
(ACAACA)N#Simple_repeat	33	1	X.calcicola GTX0491	Simple_repeat
(AGCCGA)N#Simple_repeat	33	1	X.calcicola GTX0491	Simple_repeat
(AGGA)N#Simple_repeat	33	1	X.calcicola GTX0491	Simple_repeat
(AGTCAC)N#Simple_repeat	33	1	X.calcicola GTX0491	Simple_repeat
(CACCCA)N#Simple_repeat	33	1	X.calcicola GTX0491	Simple_repeat
(CACTCCG)N#Simple_repeat	33	1	X.calcicola GTX0491	Simple_repeat
(CAGTA)N#Simple_repeat	33	1	X.calcicola GTX0491	Simple_repeat
(CATGGC)N#Simple_repeat	33	1	X.calcicola GTX0491	Simple_repeat
(CCACA)N#Simple_repeat	33	1	X.calcicola GTX0491	Simple_repeat
(CCGCAC)N#Simple_repeat	33	1	X.calcicola GTX0491	Simple_repeat
(CCTTTC)N#Simple_repeat	33	1	X.calcicola GTX0491	Simple_repeat
(CGCCAA)N#Simple_repeat	33	1	X.calcicola GTX0491	Simple_repeat
(CGCCAC)N#Simple_repeat	33	1	X.calcicola GTX0491	Simple_repeat
(CGTCCT)N#Simple_repeat	33	1	X.calcicola GTX0491	Simple_repeat
(CGTCTT)N#Simple_repeat	33	1	X.calcicola GTX0491	Simple_repeat
(CTATAT)N#Simple_repeat	33	1	X.calcicola GTX0491	Simple_repeat
(CTGC)N#Simple_repeat	33	1	X.calcicola GTX0491	Simple_repeat
(GACGCT)N#Simple_repeat	33	1	X.calcicola GTX0491	Simple_repeat
(GAGAGC)N#Simple_repeat	33	1	X.calcicola GTX0491	Simple_repeat
(GAGGGG)N#Simple_repeat	33	1	X.calcicola GTX0491	Simple_repeat
(GATCGA)N#Simple_repeat	33	1	X.calcicola GTX0491	Simple_repeat
(GATGGA)N#Simple_repeat	33	1	X.calcicola GTX0491	Simple_repeat
(GCTTCG)N#Simple_repeat	33	1	X.calcicola GTX0491	Simple_repeat
(GGTAAT)N#Simple_repeat	33	1	X.calcicola GTX0491	Simple_repeat

(GGTTGC)N#Simple_repeat	33	1	X.calcicola GTX0491	Simple_repeat
(TAATTC)N#Simple_repeat	33	1	X.calcicola GTX0491	Simple_repeat
(TCATGC)N#Simple_repeat	33	1	X.calcicola GTX0491	Simple_repeat
(TCCCCG)N#Simple_repeat	33	1	X.calcicola GTX0491	Simple_repeat
(TCCCCTA)N#Simple_repeat	33	1	X.calcicola GTX0491	Simple_repeat
(TCCCG)N#Simple_repeat	33	1	X.calcicola GTX0491	Simple_repeat
(TCGAGA)N#Simple_repeat	33	1	X.calcicola GTX0491	Simple_repeat
(TGACCG)N#Simple_repeat	33	1	X.calcicola GTX0491	Simple_repeat
(TGGGAGA)N#Simple_repeat	33	1	X.calcicola GTX0491	Simple_repeat
(TGTACGG)N#Simple_repeat	33	1	X.calcicola GTX0491	Simple_repeat
(TTCCCA)N#Simple_repeat	33	1	X.calcicola GTX0491	Simple_repeat
(TTTAAGA)N#Simple_repeat	33	1	X.calcicola GTX0491	Simple_repeat
(AACAAC)N#Simple_repeat	32	1	X.calcicola GTX0491	Simple_repeat
(AAGTC)N#Simple_repeat	32	1	X.calcicola GTX0491	Simple_repeat
(ACCAT)N#Simple_repeat	32	1	X.calcicola GTX0491	Simple_repeat
(AGCTTT)N#Simple_repeat	32	1	X.calcicola GTX0491	Simple_repeat
(ATCTTA)N#Simple_repeat	32	1	X.calcicola GTX0491	Simple_repeat
(ATGGC)N#Simple_repeat	32	1	X.calcicola GTX0491	Simple_repeat
(ATGGCG)N#Simple_repeat	32	1	X.calcicola GTX0491	Simple_repeat
(ATTATC)N#Simple_repeat	32	1	X.calcicola GTX0491	Simple_repeat
(ATTTGG)N#Simple_repeat	32	1	X.calcicola GTX0491	Simple_repeat
(CAAACT)N#Simple_repeat	32	1	X.calcicola GTX0491	Simple_repeat
(CACCAG)N#Simple_repeat	32	1	X.calcicola GTX0491	Simple_repeat
(CCAGGA)N#Simple_repeat	32	1	X.calcicola GTX0491	Simple_repeat
(CCCGAC)N#Simple_repeat	32	1	X.calcicola GTX0491	Simple_repeat
(CCCTCTT)N#Simple_repeat	32	1	X.calcicola GTX0491	Simple_repeat

(CCGATC)N#Simple_repeat	32	1	X.calcicola GTX0491	Simple_repeat
(CGGTA)N#Simple_repeat	32	1	X.calcicola GTX0491	Simple_repeat
(CTATT)N#Simple_repeat	32	1	X.calcicola GTX0491	Simple_repeat
(CTGCCA)N#Simple_repeat	32	1	X.calcicola GTX0491	Simple_repeat
(GAAGT)N#Simple_repeat	32	1	X.calcicola GTX0491	Simple_repeat
(GAGGG)N#Simple_repeat	32	1	X.calcicola GTX0491	Simple_repeat
(GCGCAG)N#Simple_repeat	32	1	X.calcicola GTX0491	Simple_repeat
(GCTGGG)N#Simple_repeat	32	1	X.calcicola GTX0491	Simple_repeat
(GGAGCG)N#Simple_repeat	32	1	X.calcicola GTX0491	Simple_repeat
(GGGACA)N#Simple_repeat	32	1	X.calcicola GTX0491	Simple_repeat
(GTCTTC)N#Simple_repeat	32	1	X.calcicola GTX0491	Simple_repeat
(GTTTT)N#Simple_repeat	32	1	X.calcicola GTX0491	Simple_repeat
(TATTTT)N#Simple_repeat	32	1	X.calcicola GTX0491	Simple_repeat
(TGGCA)N#Simple_repeat	32	1	X.calcicola GTX0491	Simple_repeat
(TGTTG)N#Simple_repeat	32	1	X.calcicola GTX0491	Simple_repeat
(TGTTTT)N#Simple_repeat	32	1	X.calcicola GTX0491	Simple_repeat
(TTGCCA)N#Simple_repeat	32	1	X.calcicola GTX0491	Simple_repeat
(TTGTTA)N#Simple_repeat	32	1	X.calcicola GTX0491	Simple_repeat
(AAAGCTA)N#Simple_repeat	31	1	X.calcicola GTX0491	Simple_repeat
(AATAAT)N#Simple_repeat	31	1	X.calcicola GTX0491	Simple_repeat
(ACATCC)N#Simple_repeat	31	1	X.calcicola GTX0491	Simple_repeat
(ACATCG)N#Simple_repeat	31	1	X.calcicola GTX0491	Simple_repeat
(ACCACC)N#Simple_repeat	31	1	X.calcicola GTX0491	Simple_repeat
(ACCTCT)N#Simple_repeat	31	1	X.calcicola GTX0491	Simple_repeat
(ACGACT)N#Simple_repeat	31	1	X.calcicola GTX0491	Simple_repeat
(ACGAGG)N#Simple_repeat	31	1	X.calcicola GTX0491	Simple_repeat

(AGACGC)N#Simple_repeat	31	1	X.calcicola GTX0491	Simple_repeat
(ATCTCG)N#Simple_repeat	31	1	X.calcicola GTX0491	Simple_repeat
(CAAATC)N#Simple_repeat	31	1	X.calcicola GTX0491	Simple_repeat
(CAGCTTC)N#Simple_repeat	31	1	X.calcicola GTX0491	Simple_repeat
(CATTC)N#Simple_repeat	31	1	X.calcicola GTX0491	Simple_repeat
(CCAATA)N#Simple_repeat	31	1	X.calcicola GTX0491	Simple_repeat
(CCGTCA)N#Simple_repeat	31	1	X.calcicola GTX0491	Simple_repeat
(CCTTGC)N#Simple_repeat	31	1	X.calcicola GTX0491	Simple_repeat
(CGGATT)N#Simple_repeat	31	1	X.calcicola GTX0491	Simple_repeat
(CGGTGC)N#Simple_repeat	31	1	X.calcicola GTX0491	Simple_repeat
(CTATCT)N#Simple_repeat	31	1	X.calcicola GTX0491	Simple_repeat
(CTGTT)N#Simple_repeat	31	1	X.calcicola GTX0491	Simple_repeat
(GAGACG)N#Simple_repeat	31	1	X.calcicola GTX0491	Simple_repeat
(GCCCTA)N#Simple_repeat	31	1	X.calcicola GTX0491	Simple_repeat
(GCCT)N#Simple_repeat	31	1	X.calcicola GTX0491	Simple_repeat
(GCCTCC)N#Simple_repeat	31	1	X.calcicola GTX0491	Simple_repeat
(GCGAAG)N#Simple_repeat	31	1	X.calcicola GTX0491	Simple_repeat
(GCGGAA)N#Simple_repeat	31	1	X.calcicola GTX0491	Simple_repeat
(GCGGCA)N#Simple_repeat	31	1	X.calcicola GTX0491	Simple_repeat
(GTGAGCG)N#Simple_repeat	31	1	X.calcicola GTX0491	Simple_repeat
(GTGTTC)N#Simple_repeat	31	1	X.calcicola GTX0491	Simple_repeat
(TAAGTT)N#Simple_repeat	31	1	X.calcicola GTX0491	Simple_repeat
(TCCTGT)N#Simple_repeat	31	1	X.calcicola GTX0491	Simple_repeat
(TGAGA)N#Simple_repeat	31	1	X.calcicola GTX0491	Simple_repeat
(TTCCCT)N#Simple_repeat	31	1	X.calcicola GTX0491	Simple_repeat
(TTGTCG)N#Simple_repeat	31	1	X.calcicola GTX0491	Simple_repeat

(TTTCG)N#Simple_repeat	31	1	X.calcicola GTX0491	Simple_repeat
(TTTGA)N#Simple_repeat	31	1	X.calcicola GTX0491	Simple_repeat
(TTTTTC)N#Simple_repeat	31	1	X.calcicola GTX0491	Simple_repeat
(AACGAG)N#Simple_repeat	30	1	X.calcicola GTX0491	Simple_repeat
(AACTTA)N#Simple_repeat	30	1	X.calcicola GTX0491	Simple_repeat
(ACGGG)N#Simple_repeat	30	1	X.calcicola GTX0491	Simple_repeat
(AGAAGG)N#Simple_repeat	30	1	X.calcicola GTX0491	Simple_repeat
(AGAATT)N#Simple_repeat	30	1	X.calcicola GTX0491	Simple_repeat
(AGAGTC)N#Simple_repeat	30	1	X.calcicola GTX0491	Simple_repeat
(AGCTGC)N#Simple_repeat	30	1	X.calcicola GTX0491	Simple_repeat
(AGGGA)N#Simple_repeat	30	1	X.calcicola GTX0491	Simple_repeat
(AGTGAG)N#Simple_repeat	30	1	X.calcicola GTX0491	Simple_repeat
(AGTTAA)N#Simple_repeat	30	1	X.calcicola GTX0491	Simple_repeat
(ATCAT)N#Simple_repeat	30	1	X.calcicola GTX0491	Simple_repeat
(ATCGGG)N#Simple_repeat	30	1	X.calcicola GTX0491	Simple_repeat
(ATCGGT)N#Simple_repeat	30	1	X.calcicola GTX0491	Simple_repeat
(CCAGGGT)N#Simple_repeat	30	1	X.calcicola GTX0491	Simple_repeat
(CCGATG)N#Simple_repeat	30	1	X.calcicola GTX0491	Simple_repeat
(CCTAC)N#Simple_repeat	30	1	X.calcicola GTX0491	Simple_repeat
(CGACTC)N#Simple_repeat	30	1	X.calcicola GTX0491	Simple_repeat
(CGTTCC)N#Simple_repeat	30	1	X.calcicola GTX0491	Simple_repeat
(CTACA)N#Simple_repeat	30	1	X.calcicola GTX0491	Simple_repeat
(CTGGTT)N#Simple_repeat	30	1	X.calcicola GTX0491	Simple_repeat
(CTGTATC)N#Simple_repeat	30	1	X.calcicola GTX0491	Simple_repeat
(CTTCG)N#Simple_repeat	30	1	X.calcicola GTX0491	Simple_repeat
(CTTTAC)N#Simple_repeat	30	1	X.calcicola GTX0491	Simple_repeat

(GATTGG)N#Simple_repeat	30	1	X.calcicola GTX0491	Simple_repeat
(GCCCA)N#Simple_repeat	30	1	X.calcicola GTX0491	Simple_repeat
(GCCGCA)N#Simple_repeat	30	1	X.calcicola GTX0491	Simple_repeat
(GGCAA)N#Simple_repeat	30	1	X.calcicola GTX0491	Simple_repeat
(GGCGA)N#Simple_repeat	30	1	X.calcicola GTX0491	Simple_repeat
(GGGGAAA)N#Simple_repeat	30	1	X.calcicola GTX0491	Simple_repeat
(GGGTTC)N#Simple_repeat	30	1	X.calcicola GTX0491	Simple_repeat
(TAAGTTA)N#Simple_repeat	30	1	X.calcicola GTX0491	Simple_repeat
(TCCAAA)N#Simple_repeat	30	1	X.calcicola GTX0491	Simple_repeat
(TCCTGG)N#Simple_repeat	30	1	X.calcicola GTX0491	Simple_repeat
(TCTGTT)N#Simple_repeat	30	1	X.calcicola GTX0491	Simple_repeat
(TCTTTT)N#Simple_repeat	30	1	X.calcicola GTX0491	Simple_repeat
(TGAC)N#Simple_repeat	30	1	X.calcicola GTX0491	Simple_repeat
(TTCCTT)N#Simple_repeat	30	1	X.calcicola GTX0491	Simple_repeat
(TTCGGA)N#Simple_repeat	30	1	X.calcicola GTX0491	Simple_repeat
(TTGAGT)N#Simple_repeat	30	1	X.calcicola GTX0491	Simple_repeat
(AAGAGAG)N#Simple_repeat	29	1	X.calcicola GTX0491	Simple_repeat
(AGGGCT)N#Simple_repeat	29	1	X.calcicola GTX0491	Simple_repeat
(AGTGAT)N#Simple_repeat	29	1	X.calcicola GTX0491	Simple_repeat
(ATCCCTC)N#Simple_repeat	29	1	X.calcicola GTX0491	Simple_repeat
(ATGGT)N#Simple_repeat	29	1	X.calcicola GTX0491	Simple_repeat
(CAACCCT)N#Simple_repeat	29	1	X.calcicola GTX0491	Simple_repeat
(CACCC)N#Simple_repeat	29	1	X.calcicola GTX0491	Simple_repeat
(CAGCC)N#Simple_repeat	29	1	X.calcicola GTX0491	Simple_repeat
(CCATCCT)N#Simple_repeat	29	1	X.calcicola GTX0491	Simple_repeat
(CCATG)N#Simple_repeat	29	1	X.calcicola GTX0491	Simple_repeat

(CCCAAG)N#Simple_repeat	29	1	X.calcicola GTX0491	Simple_repeat
(CCCAAT)N#Simple_repeat	29	1	X.calcicola GTX0491	Simple_repeat
(CCGCAGT)N#Simple_repeat	29	1	X.calcicola GTX0491	Simple_repeat
(CGAGAG)N#Simple_repeat	29	1	X.calcicola GTX0491	Simple_repeat
(CGCT)N#Simple_repeat	29	1	X.calcicola GTX0491	Simple_repeat
(CTCTTT)N#Simple_repeat	29	1	X.calcicola GTX0491	Simple_repeat
(CTTCCA)N#Simple_repeat	29	1	X.calcicola GTX0491	Simple_repeat
(GACAGG)N#Simple_repeat	29	1	X.calcicola GTX0491	Simple_repeat
(GAGGA)N#Simple_repeat	29	1	X.calcicola GTX0491	Simple_repeat
(GATACC)N#Simple_repeat	29	1	X.calcicola GTX0491	Simple_repeat
(GCAAA)N#Simple_repeat	29	1	X.calcicola GTX0491	Simple_repeat
(GCCTTT)N#Simple_repeat	29	1	X.calcicola GTX0491	Simple_repeat
(GCTTTCA)N#Simple_repeat	29	1	X.calcicola GTX0491	Simple_repeat
(GGAAAGG)N#Simple_repeat	29	1	X.calcicola GTX0491	Simple_repeat
(GGCACC)N#Simple_repeat	29	1	X.calcicola GTX0491	Simple_repeat
(GGCAT)N#Simple_repeat	29	1	X.calcicola GTX0491	Simple_repeat
(GGGAG)N#Simple_repeat	29	1	X.calcicola GTX0491	Simple_repeat
(GTATTT)N#Simple_repeat	29	1	X.calcicola GTX0491	Simple_repeat
(GTGGA)N#Simple_repeat	29	1	X.calcicola GTX0491	Simple_repeat
(GTTT)N#Simple_repeat	29	1	X.calcicola GTX0491	Simple_repeat
(TAAAGG)N#Simple_repeat	29	1	X.calcicola GTX0491	Simple_repeat
(TAACCTA)N#Simple_repeat	29	1	X.calcicola GTX0491	Simple_repeat
(TAGATA)N#Simple_repeat	29	1	X.calcicola GTX0491	Simple_repeat
(TCCCAC)N#Simple_repeat	29	1	X.calcicola GTX0491	Simple_repeat
(TCGAGG)N#Simple_repeat	29	1	X.calcicola GTX0491	Simple_repeat
(AATGGA)N#Simple_repeat	28	1	X.calcicola GTX0491	Simple_repeat

(ACCC)N#Simple_repeat	28	1	X.calcicola GTX0491	Simple_repeat
(ATAGAT)N#Simple_repeat	28	1	X.calcicola GTX0491	Simple_repeat
(ATAGC)N#Simple_repeat	28	1	X.calcicola GTX0491	Simple_repeat
(CCAAAC)N#Simple_repeat	28	1	X.calcicola GTX0491	Simple_repeat
(CCCG)N#Simple_repeat	28	1	X.calcicola GTX0491	Simple_repeat
(CCGC)N#Simple_repeat	28	1	X.calcicola GTX0491	Simple_repeat
(CCGTCCG)N#Simple_repeat	28	1	X.calcicola GTX0491	Simple_repeat
(CGGCTT)N#Simple_repeat	28	1	X.calcicola GTX0491	Simple_repeat
(CTGCTA)N#Simple_repeat	28	1	X.calcicola GTX0491	Simple_repeat
(CTGTG)N#Simple_repeat	28	1	X.calcicola GTX0491	Simple_repeat
(GCTGAC)N#Simple_repeat	28	1	X.calcicola GTX0491	Simple_repeat
(GCTTC)N#Simple_repeat	28	1	X.calcicola GTX0491	Simple_repeat
(GTCA)N#Simple_repeat	28	1	X.calcicola GTX0491	Simple_repeat
(GTGGAA)N#Simple_repeat	28	1	X.calcicola GTX0491	Simple_repeat
(TAACT)N#Simple_repeat	28	1	X.calcicola GTX0491	Simple_repeat
(TAAGT)N#Simple_repeat	28	1	X.calcicola GTX0491	Simple_repeat
(TAGAT)N#Simple_repeat	28	1	X.calcicola GTX0491	Simple_repeat
(TCCGTT)N#Simple_repeat	28	1	X.calcicola GTX0491	Simple_repeat
(TGGC)N#Simple_repeat	28	1	X.calcicola GTX0491	Simple_repeat
(TTGT)N#Simple_repeat	28	1	X.calcicola GTX0491	Simple_repeat
(TTTTG)N#Simple_repeat	28	1	X.calcicola GTX0491	Simple_repeat
(AAATC)N#Simple_repeat	27	1	X.calcicola GTX0491	Simple_repeat
(ACGGC)N#Simple_repeat	27	1	X.calcicola GTX0491	Simple_repeat
(ACTTC)N#Simple_repeat	27	1	X.calcicola GTX0491	Simple_repeat
(AGAGC)N#Simple_repeat	27	1	X.calcicola GTX0491	Simple_repeat
(AGGAT)N#Simple_repeat	27	1	X.calcicola GTX0491	Simple_repeat

(AGGGG)N#Simple_repeat	27	1	X.calcicola GTX0491	Simple_repeat
(CAGT)N#Simple_repeat	27	1	X.calcicola GTX0491	Simple_repeat
(CCTG)N#Simple_repeat	27	1	X.calcicola GTX0491	Simple_repeat
(GAAAA)N#Simple_repeat	27	1	X.calcicola GTX0491	Simple_repeat
(GAAAAT)N#Simple_repeat	27	1	X.calcicola GTX0491	Simple_repeat
(GAGCG)N#Simple_repeat	27	1	X.calcicola GTX0491	Simple_repeat
(GAGTC)N#Simple_repeat	27	1	X.calcicola GTX0491	Simple_repeat
(GCCAT)N#Simple_repeat	27	1	X.calcicola GTX0491	Simple_repeat
(GGCA)N#Simple_repeat	27	1	X.calcicola GTX0491	Simple_repeat
(GGTC)N#Simple_repeat	27	1	X.calcicola GTX0491	Simple_repeat
(GTACT)N#Simple_repeat	27	1	X.calcicola GTX0491	Simple_repeat
(TACCA)N#Simple_repeat	27	1	X.calcicola GTX0491	Simple_repeat
(TCAG)N#Simple_repeat	27	1	X.calcicola GTX0491	Simple_repeat
(TTTGG)N#Simple_repeat	27	1	X.calcicola GTX0491	Simple_repeat
(AACA)N#Simple_repeat	26	1	X.calcicola GTX0491	Simple_repeat
(AACAA)N#Simple_repeat	26	1	X.calcicola GTX0491	Simple_repeat
(AGAGAC)N#Simple_repeat	26	1	X.calcicola GTX0491	Simple_repeat
(AGCAG)N#Simple_repeat	26	1	X.calcicola GTX0491	Simple_repeat
(CAGCG)N#Simple_repeat	26	1	X.calcicola GTX0491	Simple_repeat
(CCAG)N#Simple_repeat	26	1	X.calcicola GTX0491	Simple_repeat
(GAAGGG)N#Simple_repeat	26	1	X.calcicola GTX0491	Simple_repeat
(GAGAA)N#Simple_repeat	26	1	X.calcicola GTX0491	Simple_repeat
(GCAG)N#Simple_repeat	26	1	X.calcicola GTX0491	Simple_repeat
(GGAAA)N#Simple_repeat	26	1	X.calcicola GTX0491	Simple_repeat
(GGGGA)N#Simple_repeat	26	1	X.calcicola GTX0491	Simple_repeat
(GGTAG)N#Simple_repeat	26	1	X.calcicola GTX0491	Simple_repeat

(GGTGC)N#Simple_repeat	26	1	X.calcicola GTX0491	Simple_repeat
(TACA)N#Simple_repeat	26	1	X.calcicola GTX0491	Simple_repeat
(TATG)N#Simple_repeat	26	1	X.calcicola GTX0491	Simple_repeat
(TTGCT)N#Simple_repeat	26	1	X.calcicola GTX0491	Simple_repeat
(ACTGAG)N#Simple_repeat	25	1	X.calcicola GTX0491	Simple_repeat
(ATTCG)N#Simple_repeat	25	1	X.calcicola GTX0491	Simple_repeat
(CAAAG)N#Simple_repeat	25	1	X.calcicola GTX0491	Simple_repeat
(CAGCTC)N#Simple_repeat	25	1	X.calcicola GTX0491	Simple_repeat
(CATGG)N#Simple_repeat	25	1	X.calcicola GTX0491	Simple_repeat
(CCACAC)N#Simple_repeat	25	1	X.calcicola GTX0491	Simple_repeat
(CGAGT)N#Simple_repeat	25	1	X.calcicola GTX0491	Simple_repeat
(CGATCC)N#Simple_repeat	25	1	X.calcicola GTX0491	Simple_repeat
(CGGCGT)N#Simple_repeat	25	1	X.calcicola GTX0491	Simple_repeat
(CTTG)N#Simple_repeat	25	1	X.calcicola GTX0491	Simple_repeat
(GACGG)N#Simple_repeat	25	1	X.calcicola GTX0491	Simple_repeat
(GAGGT)N#Simple_repeat	25	1	X.calcicola GTX0491	Simple_repeat
(GCTGA)N#Simple_repeat	25	1	X.calcicola GTX0491	Simple_repeat
(GCTGGA)N#Simple_repeat	25	1	X.calcicola GTX0491	Simple_repeat
(GTCGCT)N#Simple_repeat	25	1	X.calcicola GTX0491	Simple_repeat
(TCCGCA)N#Simple_repeat	25	1	X.calcicola GTX0491	Simple_repeat
(TCTGAG)N#Simple_repeat	25	1	X.calcicola GTX0491	Simple_repeat
(TGCC)N#Simple_repeat	25	1	X.calcicola GTX0491	Simple_repeat
(TGCTG)N#Simple_repeat	25	1	X.calcicola GTX0491	Simple_repeat
(TGTTT)N#Simple_repeat	25	1	X.calcicola GTX0491	Simple_repeat
(TTAATATA)N#Simple_repeat	25	1	X.calcicola GTX0491	Simple_repeat
(TTCGT)N#Simple_repeat	25	1	X.calcicola GTX0491	Simple_repeat

(AATGGG)N#Simple_repeat	24	1	X.calcicola GTX0491	Simple_repeat
(ACGA)N#Simple_repeat	24	1	X.calcicola GTX0491	Simple_repeat
(ATAAAA)N#Simple_repeat	24	1	X.calcicola GTX0491	Simple_repeat
(CACACC)N#Simple_repeat	24	1	X.calcicola GTX0491	Simple_repeat
(CACCCC)N#Simple_repeat	24	1	X.calcicola GTX0491	Simple_repeat
(CAGAG)N#Simple_repeat	24	1	X.calcicola GTX0491	Simple_repeat
(CGATC)N#Simple_repeat	24	1	X.calcicola GTX0491	Simple_repeat
(CTTA)N#Simple_repeat	24	1	X.calcicola GTX0491	Simple_repeat
(GCATT)N#Simple_repeat	24	1	X.calcicola GTX0491	Simple_repeat
(GTACA)N#Simple_repeat	24	1	X.calcicola GTX0491	Simple_repeat
(GTCC)N#Simple_repeat	24	1	X.calcicola GTX0491	Simple_repeat
(TGAGC)N#Simple_repeat	24	1	X.calcicola GTX0491	Simple_repeat
(TTGGG)N#Simple_repeat	24	1	X.calcicola GTX0491	Simple_repeat
(AAGA)N#Simple_repeat	23	1	X.calcicola GTX0491	Simple_repeat
(ACTC)N#Simple_repeat	23	1	X.calcicola GTX0491	Simple_repeat
(ACTCC)N#Simple_repeat	23	1	X.calcicola GTX0491	Simple_repeat
(AGCA)N#Simple_repeat	23	1	X.calcicola GTX0491	Simple_repeat
(ATGC)N#Simple_repeat	23	1	X.calcicola GTX0491	Simple_repeat
(GACTAC)N#Simple_repeat	23	1	X.calcicola GTX0491	Simple_repeat
(GAGG)N#Simple_repeat	23	1	X.calcicola GTX0491	Simple_repeat
(GGAC)N#Simple_repeat	23	1	X.calcicola GTX0491	Simple_repeat
(TCAA)N#Simple_repeat	23	1	X.calcicola GTX0491	Simple_repeat
(TTAC)N#Simple_repeat	23	1	X.calcicola GTX0491	Simple_repeat
(ATAC)N#Simple_repeat	22	1	X.calcicola GTX0491	Simple_repeat
(GTAG)N#Simple_repeat	22	1	X.calcicola GTX0491	Simple_repeat
(GTAT)N#Simple_repeat	22	1	X.calcicola GTX0491	Simple_repeat

(AAAT)N#Simple_repeat	21	1	X.calcicola GTX0491	Simple_repeat
(GCTT)N#Simple_repeat	21	1	X.calcicola GTX0491	Simple_repeat
(TTTCGG)N#Simple_repeat	20	1	X.calcicola GTX0491	Simple_repeat
(CATATC)N#Simple_repeat	18	1	X.calcicola GTX0491	Simple_repeat
(GAGGATG)N#Simple_repeat	16	1	X.calcicola GTX0491	Simple_repeat
(AGGCAA)N#Simple_repeat	15	1	X.calcicola GTX0491	Simple_repeat
(TACCCG)N#Simple_repeat	12	1	X.calcicola GTX0491	Simple_repeat
(CTTCTTT)N#Simple_repeat	10	1	X.calcicola GTX0491	Simple_repeat
(TGCTC)N#Simple_repeat	7	1	X.calcicola GTX0491	Simple_repeat
(TTATAT)N#Simple_repeat	7	1	X.calcicola GTX0491	Simple_repeat

Table S3. Mobile elements identified in the four genomes of Xanthoria fungi by Starfish. For each element, we show informatin about it position and the position of corresponding 'empty' site. When availble, coordinates of direct repeats (DR) and tandem inverted repeats (TIR) are shown. The elements correponding to Tangerine are highlighted in bold.

elementID	elementGenome	elementCaptainID	elementContigID	element Begin	element End	element Length	element Strand	emptySiteID
**OZ2100_s00001**	**Xanthoria parietina isolate glXanPari1**	**OZ2100_002162-T1**	**OZ2100_30**	**304726**	**406787**	**102062**	**"+"**	**XcGTX0491_site001**
**scaffold_s00008**	**Xanthoria parietina isolate glXanPari20**	**scaffold_008752-T1**	**scaffold_12**	**662355**	**747238**	**84884**	**-**	**XcGTX0491_site001**
OZ2100_s00003	Xanthoria parietina isolate glXanPari1	OZ2100_tyr4	OZ2100_35	343880	361790	17911	-	scaffold_site001

elementID	emptySiteID	emptyContigID	emptyBegin	emptyEnd	emptyLength	emptyStrand	emptySiteSeq	quality	upDR
**OZ2100_s00001**	**XcGTX0491_site001**	**XcGTX0491_4**	**1852373**	**1852376**	**4**	**"+"**	**gcgg**	**ref**	**cccgc**
**scaffold_s00008**	**XcGTX0491_site001**	**XcGTX0491_4**	**1852373**	**1852376**	**4**	**"+"**	**ccgc**	**ref**	**ccgcc**
OZ2100_s00003	scaffold_site001	scaffold_4	1106074	1108028	1955	-	*empty site > 40 bp	ref	

elementID	upDRbegin	upDRend	downDR	downDRbegin	downDRend	upTIR	upTIRbegin	upTIRend	downTIR	downTIRbegin	downTIRend
**OZ2100_s00001**	**747238**	**747242**	**ccgc**	**662352**	**662355**	**.**	**.**	**.**	**.**	**.**	**.**
**scaffold_s00008**	**304723**	**304727**	**ccgc**	**406787**	**406790**	**tccaaatgga**	**304741**	**304750**	**tccacttatgga**	**406748**	**406759**
OZ2100_s00003											

Table S4. Tyrosine recombinase genes identified in the four genomes of Xanthoria fungi by Starfish. The genes are grouped into naves (orthologous groups) based on sequence similarity and assigned to a family/clade based on hmmsearch search. The genes correponding to Tangerine captains are highlighted in bold.

geneID	Genome	contig	Start	End	Strand	navis	family hit	evalue	Associated with a predicted element
XANPAOZ2100_003971-T1	Xanthoria parietina isolate glXanPari1	OZ2100_32	1910251	1912801	-	navis0001	phoenixFam	1.10E-85	No, but has possible auxillary genes in a vicnitiy (an NLR within 40 kb)
XANCAGTX0491_009866-T1	Xanthoria calcicola isolate GTX0491	XcGTX0491_15	853712	856255	"+"	navis0001	phoenixFam	4.80E-85	No
XANPAGTX0501_002936-T1	Xanthoria parietina isolate GTX0501	XpGTX0501_4	1205340	1208010	-	navis0001	phoenixFam	1.90E-73	No
XANPAGTX0501_007307-T1	Xanthoria parietina isolate GTX0501	XpGTX0501_12	129424	131974	"+"	navis0001	phoenixFam	1.10E-85	No, but has possible auxillary genes in a vicnitiy (an NLR within 38 kb)
glXanPari20_005633-T1	Xanthoria parietina isolate glXanPari20	scaffold_5	2024608	2027212	-	navis0001	phoenixFam	3.70E-86	No, but has possible auxillary genes in a vicnitiy (an NLR within 40 kb)
**XANPAOZ2100_002162-T1**	**Xanthoria parietina isolate glXanPari1**	**OZ2100_30**	**305825**	**307015**	**"+"**	**navis0002**	**clade1**	**2.30E-65**	**Yes, Tangerine**
**XANPAGTX0501_001716-T1**	**Xanthoria parietina isolate GTX0501**	**XpGTX0501_3**	**6660**	**7805**	**"+"**	**navis0002**	**clade1**	**6.10E-59**	**Yes, Tangerine**
**XANPAGTX0501_010272-T1**	**Xanthoria parietina isolate GTX0501**	**XpGTX0501_31**	**125644**	**126848**	**"+"**	**navis0002**	**clade1**	**4.80E-43**	**Yes, Tangerine**

**glXanPari20_008752-T1**	**Xanthoria parietina isolate glXanPari20**	**scaffold_12**	**744949**	**746139**	**-**	**navis0002**	**clade1**	**2.30E-65**	**Yes, Tangerine**
XANPAOZ2100_008954-T1	Xanthoria parietina isolate glXanPari1	OZ2100_43	931278	932679	-	navis0003	galacticaFam	6.60E-62	No
XANPAGTX0501_010485-T1	Xanthoria parietina isolate GTX0501	XpGTX0501_37	760	1759	"+"	navis0003	galacticaFam	8.50E-90	No, but has possible auxillary genes in a vicnitiy (PLP in 22 kb; Ferric Reductase in 41 kb)
glXanPari20_007362-T1	Xanthoria parietina isolate glXanPari20	scaffold_8	1503178	1505738	-	navis0003	galacticaFam	1.30E-144	No, but has possible auxillary genes in a vicnitiy (PLP in 18 kb; DUF3723 in 15 kb)
XANPAGTX0501_tyr12	Xanthoria parietina isolate GTX0501	XpGTX0501_5	1670285	1672933	-	navis0004	prometheusFam	4.10E-61	No
XANPAGTX0501_010572-T1	Xanthoria parietina isolate GTX0501	XpGTX0501_39	70667	72714	-	navis0004	prometheusFam	1.20E-82	No
XANCAGTX0491_tyr8	Xanthoria calcicola isolate GTX0491	XcGTX0491_12	23500	23784	"+"	navis0005	arwingFam	4.40E-11	No
XANCAGTX0491_008785-T1	Xanthoria calcicola isolate GTX0491	XcGTX0491_12	1478908	1481335	-	navis0006	phoenixFam	5.00E-128	No
glXanPari20_tyr17	Xanthoria parietina isolate glXanPari20	scaffold_4	1310219	1310743	-	navis0007	phoenixFam	2.00E-17	No
XANPAOZ2100_tyr3	Xanthoria parietina isolate glXanPari1	OZ2100_35	359121	359324	-	navis0008	moyaFam	1.40E-06	No
XANPAOZ2100_tyr4	Xanthoria parietina isolate glXanPari1	OZ2100_35	359761	360277	-	navis0009	clade2	1.40E-34	Yes, likely a false positive (lacks any other gene model, and shorter than expected with only 18 kb, associated with an empty site of 2kb)

XANCAGTX0491_002172-T1	Xanthoria calcicola isolate GTX0491	XcGTX0491_2	2632559	2633446	"+"	navis0010	clade3	1.70E-44	No, but has possible auxillary genes in a vicnitiy (NLR in 71 kb)

Table S5. Gene models present within the Tangerine element in the three genomes of X. parietina.

Genome	GeneID	TranscriptID	Contig	Start	Stop	InterPro
Xanthoria parietina isolate glXanPari1	XANPAOZ2100_002162	XANPAOZ2100_002162-T1	OZ210030	306115	307018	IPR021842 Protein of unknown function DUF3435
Xanthoria parietina isolate glXanPari1	XANPAOZ2100_002163	XANPAOZ2100_002163-T1	OZ210030	361239	362028	
Xanthoria parietina isolate glXanPari1	XANPAOZ2100_002164	XANPAOZ2100_002164-T1	OZ210030	362108	363397	IPR022198 Protein of unknown function DUF3723
Xanthoria parietina isolate glXanPari1	XANPAOZ2100_002165	XANPAOZ2100_002165-T1	OZ210030	363598	366226	IPR002641 Patatin-like phospholipase domain;IPR016035 Acyl transferase/acyl hydrolase/lysophospholipase
Xanthoria parietina isolate glXanPari1	XANPAOZ2100_002166	XANPAOZ2100_002166-T1	OZ210030	369245	370049	IPR013216 Methyltransferase type 11;IPR029063 S-adenosyl-L-methionine-dependent methyltransferase superfamily
Xanthoria parietina isolate glXanPari1	XANPAOZ2100_002167	XANPAOZ2100_002167-T1	OZ210030	370399	374238	IPR002110 Ankyrin repeat;IPR007111 NACHT nucleoside triphosphatase;IPR027417 P-loop containing nucleoside triphosphate hydrolase;IPR036770 Ankyrin repeat-containing domain superfamily
Xanthoria parietina isolate glXanPari20	glXanPari20_008745	glXanPari20_008745-T1	scaffold_12	678924	679379	IPR002110 Ankyrin repeat;IPR036770 Ankyrin repeat-containing domain superfamily
Xanthoria parietina isolate glXanPari20	glXanPari20_008746	glXanPari20_008746-T1	scaffold_12	688939	692778	IPR002110 Ankyrin repeat;IPR007111 NACHT nucleoside triphosphatase;IPR027417 P-loop containing nucleoside triphosphate hydrolase;IPR036770 Ankyrin repeat-containing domain superfamily
Xanthoria parietina isolate glXanPari20	glXanPari20_008747	glXanPari20_008747-T1	scaffold_12	693128	693932	IPR013216 Methyltransferase type 11;IPR029063 S-adenosyl-L-methionine-dependent methyltransferase superfamily
Xanthoria parietina isolate glXanPari20	glXanPari20_008748	glXanPari20_008748-T1	scaffold_12	696951	699579	IPR002641 Patatin-like phospholipase domain;IPR016035 Acyl transferase/acyl hydrolase/lysophospholipase
Xanthoria parietina isolate glXanPari20	glXanPari20_008749	glXanPari20_008749-T1	scaffold_12	699780	702351	IPR022198 Protein of unknown function DUF3723

Xanthoria parietina isolate glXanPari20	glXanPari20_008750	glXanPari20_008750-T1	scaffold_12	702431	703970	
Xanthoria parietina isolate glXanPari20	glXanPari20_008751	glXanPari20_008751-T1	scaffold_12	704329	704922	
Xanthoria parietina isolate glXanPari20	glXanPari20_008752	glXanPari20_008752-T1	scaffold_12	744946	745849	IPR002104 Integrase, catalytic domain;IPR011010 DNA breaking-rejoining enzyme, catalytic core;IPR013762 Integrase-like, catalytic domain superfamily;IPR021842 Protein of unknown function DUF3435
Xanthoria parietina isolate GTX0501	XANPAGTX0501_001716	XANPAGTX0501_001716-T1	Xp_GTX0501_3	6803	8423	IPR011010 DNA breaking-rejoining enzyme, catalytic core;IPR013762 Integrase-like, catalytic domain superfamily;IPR021842 Protein of unknown function DUF3435
Xanthoria parietina isolate GTX0501	XANPAGTX0501_001717	XANPAGTX0501_001717-T1	Xp_GTX0501_3	46309	46697	
Xanthoria parietina isolate GTX0501	XANPAGTX0501_001718	XANPAGTX0501_001718-T1	Xp_GTX0501_3	47056	47545	
Xanthoria parietina isolate GTX0501	XANPAGTX0501_001720	XANPAGTX0501_001720-T1	Xp_GTX0501_3	47350	49964	IPR022198 Protein of unknown function DUF3723
Xanthoria parietina isolate GTX0501	XANPAGTX0501_001720	XANPAGTX0501_001720-T2	Xp_GTX0501_3	47350	49964	IPR022198 Protein of unknown function DUF3723
Xanthoria parietina isolate GTX0501	XANPAGTX0501_001719	XANPAGTX0501_001719-T1	Xp_GTX0501_3	47806	48595	
Xanthoria parietina isolate GTX0501	XANPAGTX0501_001721	XANPAGTX0501_001721-T1	Xp_GTX0501_3	50165	52793	IPR001841 Zinc finger, RING-type;IPR002641 Patatin-like phospholipase domain;IPR016035 Acyl transferase/acyl hydrolase/lysophospholipase;IPR017907 Zinc finger, RING-type, conserved site
Xanthoria parietina isolate GTX0501	XANPAGTX0501_001722	XANPAGTX0501_001722-T1	Xp_GTX0501_3	53007	53381	
Xanthoria parietina isolate GTX0501	XANPAGTX0501_001723	XANPAGTX0501_001723-T1	Xp_GTX0501_3	55812	56616	IPR013216 Methyltransferase type 11;IPR029063 S-adenosyl-L-methionine-dependent methyltransferase superfamily

Xanthoria parietina isolate GTX0501	XANPAGTX0501_001724	XANPAGTX0501_001724-T1	Xp_GTX0501_3	56966	60805	IPR002110 Ankyrin repeat;IPR007111 NACHT nucleoside triphosphatase;IPR020683 Domain of unknown function DUF3447;IPR027417 P-loop containing nucleoside triphosphate hydrolase;IPR036770 Ankyrin repeat-containing domain superfamily
Xanthoria parietina isolate GTX0501	XANPAGTX0501_001725	XANPAGTX0501_001725-T1	Xp_GTX0501_3	61724	62779	
Xanthoria parietina isolate GTX0501	XANPAGTX0501_001726	XANPAGTX0501_001726-T1	Xp_GTX0501_3	73434	73812	
Xanthoria parietina isolate GTX0501	XANPAGTX0501_001727	XANPAGTX0501_001727-T1	Xp_GTX0501_3	75168	76047	
Xanthoria parietina isolate GTX0501	XANPAGTX0501_001728	XANPAGTX0501_001728-T1	Xp_GTX0501_3	76066	76559	IPR002110 Ankyrin repeat;IPR020683 Domain of unknown function DUF3447;IPR036770 Ankyrin repeat-containing domain superfamily

Table S6. Repeat family-level summaries for the Tangerine element in two X. parietina genomes (glXanPari20 and glXanPari1), for the LRAR (large RIP-affected region) downstream of Tangerine in X. parietina, and the corresponding LRAR in X. calcicola downstream of the empty site. Repeats were annotated with Earl Grey.

**Region**	**Superfamily**	**Family**	**Total bp in the region**
Tangerine, X. parietina glXanPari20	LINE/Tad1	RND-1_FAMILY-1	17153
Tangerine, X. parietina glXanPari20	LTR/Ty3	RND-2_FAMILY-14	10444
Tangerine, X. parietina glXanPari20	LTR/Ty3	RND-1_FAMILY-57	6688
Tangerine, X. parietina glXanPari20	LTR/Ty3	RND-1_FAMILY-24	6629
Tangerine, X. parietina glXanPari20	LINE/Tad1	RND-1_FAMILY-82	1998
Tangerine, X. parietina glXanPari20	LINE/Tad1	RND-1_FAMILY-2	1645
Tangerine, X. parietina glXanPari20	Unknown	RND-1_FAMILY-63	565
Tangerine, X. parietina glXanPari20	LTR/Ty3	RND-3_FAMILY-123	168
Tangerine, X. parietina glXanPari20	Unknown	RND-1_FAMILY-8	93
Tangerine, X. parietina glXanPari20	Simple_repeat	(CCTCT)N	39
Tangerine, X. parietina glXanPari20	Simple_repeat	(AATTATC)N	37
Tangerine, X. parietina glXanPari20	Simple_repeat	(CAAC)N	35
Tangerine, X. parietina glXanPari20	Simple_repeat	(GAT)N	35
Tangerine, X. parietina glXanPari20	Simple_repeat	(ATCAA)N	27
Tangerine, X. parietina glXanPari20	Simple_repeat	(GGT)N	23
Tangerine, X. parietina glXanPari1	LINE/Tad1	RND-1_FAMILY-1	22490
Tangerine, X. parietina glXanPari1	LTR/Ty3	RND-3_FAMILY-139	8323
Tangerine, X. parietina glXanPari1	LTR/Ty3	RND-1_FAMILY-66	6902
Tangerine, X. parietina glXanPari1	LTR/Ty3	RND-1_FAMILY-67	6684
Tangerine, X. parietina glXanPari1	LTR/Ty3	RND-1_FAMILY-7	6631
Tangerine, X. parietina glXanPari1	LTR/Ty3	RND-1_FAMILY-29	5952
Tangerine, X. parietina glXanPari1	LINE/I	RND-1_FAMILY-32	1998

Tangerine, X. parietina glXanPari1	Unknown	RND-1_FAMILY-20	1644
Tangerine, X. parietina glXanPari1	LTR/Ty3	RND-2_FAMILY-34	1238
Tangerine, X. parietina glXanPari1	LTR/Ty3	RND-3_FAMILY-138	879
Tangerine, X. parietina glXanPari1	Unknown	RND-1_FAMILY-64	494
Tangerine, X. parietina glXanPari1	LTR/Copia	RND-1_FAMILY-36	217
Tangerine, X. parietina glXanPari1	Unknown	RND-1_FAMILY-60	69
Tangerine, X. parietina glXanPari1	Low_complexity	GA-RICH	39
Tangerine, X. parietina glXanPari1	Simple_repeat	(ATTGATA)N	37
Tangerine, X. parietina glXanPari1	Unknown	RND-3_FAMILY-840	37
Tangerine, X. parietina glXanPari1	Simple_repeat	(ATC)N	35
Tangerine, X. parietina glXanPari1	Simple_repeat	(GTTG)N	35
Tangerine, X. parietina glXanPari1	Simple_repeat	(TTGAT)N	27
Tangerine, X. parietina glXanPari1	Simple_repeat	(CCA)N	23
LRAR downstream of the empty site, X. calcicola GTX0491	LTR/Ty3	RND-1_FAMILY-203	11773
LRAR downstream of the empty site, X. calcicola GTX0491	LTR/Copia	RND-1_FAMILY-109	8023
LRAR downstream of the empty site, X. calcicola GTX0491	LTR/Ty3	RND-1_FAMILY-147	7284
LRAR downstream of the empty site, X. calcicola GTX0491	Unknown	RND-1_FAMILY-55	6749
LRAR downstream of the empty site, X. calcicola GTX0491	LTR/Ty3	RND-1_FAMILY-104	6667
LRAR downstream of the empty site, X. calcicola GTX0491	LTR/Ty3	RND-1_FAMILY-155	6646
LRAR downstream of the empty site, X. calcicola GTX0491	LTR/Ty3	RND-2_FAMILY-17	6084
LRAR downstream of the empty site, X. calcicola GTX0491	Unknown	RND-1_FAMILY-119	4180
LRAR downstream of the empty site, X. calcicola GTX0491	Unknown	RND-1_FAMILY-118	3528
LRAR downstream of the empty site, X. calcicola GTX0491	Unknown	RND-1_FAMILY-29	2882
LRAR downstream of the empty site, X. calcicola GTX0491	SINE/B2	RND-1_FAMILY-121	2505
LRAR downstream of the empty site, X. calcicola GTX0491	DNA/TcMar-Fot1	RND-1_FAMILY-106	2341
LRAR downstream of the empty site, X. calcicola GTX0491	Unknown	RND-1_FAMILY-11	1682

LRAR downstream of the empty site, X. calcicola GTX0491	Unknown	RND-1_FAMILY-150	1377
LRAR downstream of the empty site, X. calcicola GTX0491	LTR/Ty3	RND-1_FAMILY-185	1209
LRAR downstream of the empty site, X. calcicola GTX0491	LTR/Copia	RND-3_FAMILY-138	1205
LRAR downstream of the empty site, X. calcicola GTX0491	Unknown	RND-1_FAMILY-184	1115
LRAR downstream of the empty site, X. calcicola GTX0491	Unknown	RND-1_FAMILY-165	895
LRAR downstream of the empty site, X. calcicola GTX0491	Unknown	RND-1_FAMILY-194	791
LRAR downstream of the empty site, X. calcicola GTX0491	Unknown	RND-1_FAMILY-17	734
LRAR downstream of the empty site, X. calcicola GTX0491	Unknown	RND-1_FAMILY-158	717
LRAR downstream of the empty site, X. calcicola GTX0491	Unknown	RND-1_FAMILY-70	701
LRAR downstream of the empty site, X. calcicola GTX0491	LTR/Ty3	RND-1_FAMILY-45	610
LRAR downstream of the empty site, X. calcicola GTX0491	Unknown	RND-1_FAMILY-174	486
LRAR downstream of the empty site, X. calcicola GTX0491	Unknown	RND-1_FAMILY-141	249
LRAR downstream of the empty site, X. calcicola GTX0491	Unknown	RND-1_FAMILY-115	232
LRAR downstream of the empty site, X. calcicola GTX0491	LTR/Ty3	RND-1_FAMILY-100	218
LRAR downstream of the empty site, X. calcicola GTX0491	Unknown	RND-1_FAMILY-130	143
LRAR downstream of the empty site, X. calcicola GTX0491	Unknown	RND-1_FAMILY-133	135
LRAR downstream of the empty site, X. calcicola GTX0491	Simple_repeat	(TA)N	129
LRAR downstream of the empty site, X. calcicola GTX0491	Simple_repeat	(TTAT)N	115
LRAR downstream of the empty site, X. calcicola GTX0491	Simple_repeat	(ATTT)N	81
LRAR downstream of the empty site, X. calcicola GTX0491	Simple_repeat	(TAAT)N	75
LRAR downstream of the empty site, X. calcicola GTX0491	Unknown	RND-1_FAMILY-50	66
LRAR downstream of the empty site, X. calcicola GTX0491	Simple_repeat	(TTA)N	65
LRAR downstream of the empty site, X. calcicola GTX0491	Simple_repeat	(ATTTAT)N	55
LRAR downstream of the empty site, X. calcicola GTX0491	Simple_repeat	(CTTTAT)N	45
LRAR downstream of the empty site, X. calcicola GTX0491	Simple_repeat	(TACTCT)N	43
LRAR downstream of the empty site, X. calcicola GTX0491	Simple_repeat	(ATTTA)N	41

LRAR downstream of the empty site, X. calcicola GTX0491	Simple_repeat	(TATTATATAT)N	41
LRAR downstream of the empty site, X. calcicola GTX0491	Simple_repeat	(CTAATATAT)N	39
LRAR downstream of the empty site, X. calcicola GTX0491	Simple_repeat	(TTTAC)N	37
LRAR downstream of the empty site, X. calcicola GTX0491	Simple_repeat	(CTTATC)N	36
LRAR downstream of the empty site, X. calcicola GTX0491	Simple_repeat	(TAA)N	33
LRAR downstream of Tangerine, X. parietina glXanPari20	LTR/Ty3	RND-1_FAMILY-60	6752
LRAR downstream of Tangerine, X. parietina glXanPari20	LTR/Ty3	RND-1_FAMILY-57	6504
LRAR downstream of Tangerine, X. parietina glXanPari20	LINE/Tad1	RND-1_FAMILY-1	5896
LRAR downstream of Tangerine, X. parietina glXanPari20	LINE/Tad1	RND-1_FAMILY-43	5714
LRAR downstream of Tangerine, X. parietina glXanPari20	LINE/Tad1	RND-1_FAMILY-75	5361
LRAR downstream of Tangerine, X. parietina glXanPari20	LINE/Tad1	RND-1_FAMILY-2	3050
LRAR downstream of Tangerine, X. parietina glXanPari20	LTR/Ty3	RND-1_FAMILY-97	1385
LRAR downstream of Tangerine, X. parietina glXanPari20	LTR/Ty3	RND-1_FAMILY-92	169
LRAR downstream of Tangerine, X. parietina glXanPari20	Simple_repeat	(TAAATC)N	34
LRAR downstream of Tangerine, X. parietina glXanPari20	Simple_repeat	(AATATT)N	33
LRAR downstream of Tangerine, X. parietina glXanPari20	Simple_repeat	(TTA)N	21
LRAR downstream of Tangerine, X. parietina glXanPari1	LINE/Tad1	RND-1_FAMILY-48	17197
LRAR downstream of Tangerine, X. parietina glXanPari1	LINE/Tad1	RND-1_FAMILY-1	9137
LRAR downstream of Tangerine, X. parietina glXanPari1	LTR/Ty3	RND-1_FAMILY-7	6730
LRAR downstream of Tangerine, X. parietina glXanPari1	LTR/Ty3	RND-1_FAMILY-22	6555
LRAR downstream of Tangerine, X. parietina glXanPari1	LINE/Tad1	RND-1_FAMILY-57	5403
LRAR downstream of Tangerine, X. parietina glXanPari1	LTR/Copia	RND-1_FAMILY-37	2413
LRAR downstream of Tangerine, X. parietina glXanPari1	LTR/Ty3	RND-1_FAMILY-58	666
LRAR downstream of Tangerine, X. parietina glXanPari1	LTR/Ty3	RND-1_FAMILY-67	399
LRAR downstream of Tangerine, X. parietina glXanPari1	LTR/Ty3	RND-2_FAMILY-86	174
LRAR downstream of Tangerine, X. parietina glXanPari1	Unknown	RND-1_FAMILY-46	171

LRAR downstream of Tangerine, X. parietina glXanPari1	LTR/Ty3	RND-1_FAMILY-29	85
LRAR downstream of Tangerine, X. parietina glXanPari1	Simple_repeat	(TTTATA)N	77
LRAR downstream of Tangerine, X. parietina glXanPari1	LTR/Ty3	RND-1_FAMILY-66	56
LRAR downstream of Tangerine, X. parietina glXanPari1	Simple_repeat	(ATTAAG)N	34
LRAR downstream of Tangerine, X. parietina glXanPari1	Simple_repeat	(TAG)N	22

Table S7. Structural variants (SV) in Tangerine and in 200 kb flanking regions. SV were annotated using Sniffles.

ID	Position	Position relative Starship	Filter	Quality	SV type	SV length	Supporting reads	Variant Allele Frequency
Sniffles2.DEL.32S2	101361	Upstream	GT: low number of supporting reads	60	DEL (deletion)	-106	79	0.081
Sniffles2.DEL.34S2	104064	Upstream	GT: low number of supporting reads	60	DEL (deletion)	-67	123	0.077
Sniffles2.DEL.36S2	120861	Upstream	GT: low number of supporting reads	60	DEL (deletion)	-99	135	0.074
Sniffles2.DEL.5AS2	124896	Upstream	GT: low number of supporting reads	60	DEL (deletion)	-55297	122	0.088
Sniffles2.INS.12S2	307925	Inside the element	PASS	52	INS (insertion)	5836	70	0.95
Sniffles2.DEL.65S2	314828	Inside the element	PASS	59	DEL (deletion)	-227	1344	0.484
Sniffles2.INS.15S2	316279	Inside the element	PASS	56	INS (insertion)	444	711	0.29
Sniffles2.BND.29BS2	318563	Inside the element	PASS	59	BND (break point)	NA	1249	0.973
Sniffles2.DEL.6FS2	322802	Inside the element	PASS	57	DEL (deletion)	-6905	625	0.48
Sniffles2.DEL.76S2	335206	Inside the element	PASS	58	DEL (deletion)	-5361	809	0.501
Sniffles2.INS.2BS2	362686	Inside the element	PASS	59	INS (insertion)	1282	1068	0.926
Sniffles2.DEL.89S2	399016	Inside the element	PASS	60	DEL (deletion)	-252	1335	0.478
Sniffles2.DEL.8BS2	399686	Inside the element	PASS	59	DEL (deletion)	-5787	1417	0.419
Sniffles2.INS.2CS2	405201	Inside the element	PASS	58	INS (insertion)	50	1266	0.984
Sniffles2.INS.2DS2	426834	Downstream	PASS	58	INS (insertion)	2987	562	1
Sniffles2.BND.38AS2	426846	Downstream	PASS	60	BND (break point)	NA	322	0.363

Table S8. Tyrosine recombinases (tyrRs) included in the structure-based phylogeny. The set included manually selected representative set of captains from Gluck-Thaler&Vogan (2024), predicted tyrRs from a larger set of lichen-associated fungi, and Tangerine captains. The family assignments correspond to that from Gluck-Thaler&Vogan (2024).

Name	Genome	contig	Start	End	Source	family
cladophialophora.endolichena.1_tyr5	cladophialophora.endolichena.1	cladophialophora.endolichena.1_5	6540980	6543177	Newly-annotated lichen genomes	
cladophialophora.sp..2_tyr403	cladophialophora.sp..2	cladophialophora.sp..2_10	2771492	2774932	Newly-annotated lichen genomes	
cladophialophora.sp..1_tyr3153	cladophialophora.sp..1	cladophialophora.sp..1_8	3134109	3136009	Newly-annotated lichen genomes	
rhimac1_11078					Gluck-Thaler and Vogan (2024)	fam07
elagra2_tyr4830					Gluck-Thaler and Vogan (2024)	fam07
Starship-1_Cpsa-Captain					Gluck-Thaler and Vogan (2024)	fam07
clapsa1_10577					Gluck-Thaler and Vogan (2024)	fam07
phachl1_4316					Gluck-Thaler and Vogan (2024)	fam07
melanina.sp..1_tyr579	melanina.sp..1	melanina.sp..1_4	920878	923632	Newly-annotated lichen genomes	
exospi1_9399					Gluck-Thaler and Vogan (2024)	fam07
botryolepraria.lesdainii.1_tyr1357	botryolepraria.lesdainii.1	botryolepraria.lesdainii.1_1045	3502	6228	Newly-annotated lichen genomes	
chaetothyriales.sp..1_tyr954	chaetothyriales.sp..1	chaetothyriales.sp..1_9	2353784	2355801	Newly-annotated lichen genomes	
cladophialophora.sp..2_tyr3780	cladophialophora.sp..2	cladophialophora.sp..2_6	4157374	4159124	Newly-annotated lichen genomes	
fonmon1_2411_2412					Gluck-Thaler and Vogan (2024)	n.d.
endocarpon.pusillum.2_tyr109	endocarpon.pusillum.2	endocarpon.pusillum.2_20	670433	672548	Newly-annotated lichen genomes	
endocarpon.pusillum.2_tyr1874	endocarpon.pusillum.2	endocarpon.pusillum.2_17	432007	435941	Newly-annotated lichen genomes	
gyalolechia.sp..2_tyr1934	gyalolechia.sp..2	gyalolechia.sp..2_825	5588	6707	Newly-annotated lichen genomes	

endocarpon.pusillum.3_tyr187	endocarpon.pusillum.3	endocarpon.pusillum.3_891	45672	48166	Newly-annotated lichen genomes	
endocarpon.pusillum.2_tyr350	endocarpon.pusillum.2	endocarpon.pusillum.2_14	202029	204583	Newly-annotated lichen genomes	
cypeur1_114146					Gluck-Thaler and Vogan (2024)	fam07
umbilicaria.polyphylla.2_tyr2880	umbilicaria.polyphylla.2	umbilicaria.polyphylla.2_24	59470	60901	Newly-annotated lichen genomes	
rhizocarpon.geographicum.1_tyr3008	rhizocarpon.geographicum.1	rhizocarpon.geographicum.1_869	7598	10178	Newly-annotated lichen genomes	
pseudosagedia.isidiata.1_tyr707	pseudosagedia.isidiata.1	pseudosagedia.isidiata.1_220	16982	19411	Newly-annotated lichen genomes	
endocarpon.pusillum.3_tyr1094	endocarpon.pusillum.3	endocarpon.pusillum.3_413	54996	56997	Newly-annotated lichen genomes	
colnym1_1013311					Gluck-Thaler and Vogan (2024)	fam07
phachl1_5955					Gluck-Thaler and Vogan (2024)	fam07
umbilicaria.freyi.1_tyr3723	umbilicaria.freyi.1	umbilicaria.freyi.1_97	34586	36683	Newly-annotated lichen genomes	
elagra2_tyr4812					Gluck-Thaler and Vogan (2024)	fam07
gluame1_KAH0543790.1					Gluck-Thaler and Vogan (2024)	fam07
amores1_PSS18507.1					Gluck-Thaler and Vogan (2024)	fam07
beabas1_10106					Gluck-Thaler and Vogan (2024)	fam07
mictri1_252258					Gluck-Thaler and Vogan (2024)	fam07
torhem1_9920					Gluck-Thaler and Vogan (2024)	fam07
stacha1_4873					Gluck-Thaler and Vogan (2024)	fam07
metacr1_2241					Gluck-Thaler and Vogan (2024)	fam07
pocchl3_OAQ57905.2					Gluck-Thaler and Vogan (2024)	fam07
fusneo1_tyr6117					Gluck-Thaler and Vogan (2024)	fam07

fussp.8_RSL81990.1					Gluck-Thaler and Vogan (2024)	fam07
dacest1_679540					Gluck-Thaler and Vogan (2024)	fam07
fusfus45_tyr5792					Gluck-Thaler and Vogan (2024)	fam07
fusman3_tyr6063					Gluck-Thaler and Vogan (2024)	fam07
fusfus45_tyr5796					Gluck-Thaler and Vogan (2024)	fam07
fusver2_EWG46573.1					Gluck-Thaler and Vogan (2024)	fam07
metmaj1_tyr8185					Gluck-Thaler and Vogan (2024)	fam07
fusoxy44_KAG7415401.1_KAG7415409.1					Gluck-Thaler and Vogan (2024)	n.d.
fusoxy36_RKK64490.1					Gluck-Thaler and Vogan (2024)	fam07
dacmac1_17988					Gluck-Thaler and Vogan (2024)	fam07
ilysp.1_683701					Gluck-Thaler and Vogan (2024)	fam07
chaglo1_574115					Gluck-Thaler and Vogan (2024)	fam07
colfru5_tyr4312					Gluck-Thaler and Vogan (2024)	fam07
coltof2_tyr4402					Gluck-Thaler and Vogan (2024)	fam07
coltri1_tyr4416					Gluck-Thaler and Vogan (2024)	fam07
coltru1_KAF6782444.1					Gluck-Thaler and Vogan (2024)	fam07
colnym1_1020188					Gluck-Thaler and Vogan (2024)	fam07
coltof2_2047					Gluck-Thaler and Vogan (2024)	fam07
muellerella.sp..1_tyr85	muellerella.sp..1	muellerella.sp..1_5	2664617	2667065	Newly-annotated lichen genomes	
chasp.7_RMZ91819.1					Gluck-Thaler and Vogan (2024)	fam07

platismatia.glauca.2_tyr27	platismatia.glauca.2	platismatia.glauca.2_169	14733	15630	Newly-annotated lichen genomes
rhizocarpon.geographicum.1_tyr1674	rhizocarpon.geographicum.1	rhizocarpon.geographicum.1_336	9113	10967	Newly-annotated lichen genomes
protoblastenia.rupestris.2_tyr3986	protoblastenia.rupestris.2	protoblastenia.rupestris.2_145	10257	11828	Newly-annotated lichen genomes
micareopsis.irriguata.1_tyr744	micareopsis.irriguata.1	micareopsis.irriguata.1_860	5908	7861	Newly-annotated lichen genomes
parmeliaceae.sp..16_tyr251	parmeliaceae.sp..16	parmeliaceae.sp..16_2201	2	946	Newly-annotated lichen genomes
hypogymnia.physodes.1_tyr649	hypogymnia.physodes.1	hypogymnia.physodes.1_1281	398	1967	Newly-annotated lichen genomes
usnea.ceratina.2_tyr3511	usnea.ceratina.2	usnea.ceratina.2_993	5416	6157	Newly-annotated lichen genomes
lecanora.oreinoides.1_tyr3125	lecanora.oreinoides.1	lecanora.oreinoides.1_625	10943	12289	Newly-annotated lichen genomes
cladonia.kurokawae.1_tyr1067	cladonia.kurokawae.1	cladonia.kurokawae.1_229	13025	14534	Newly-annotated lichen genomes
lecanora.helva.1_tyr1660	lecanora.helva.1	lecanora.helva.1_2	135861	138331	Newly-annotated lichen genomes
buellia.spuria.2_tyr2143	buellia.spuria.2	buellia.spuria.2_4	69914	72187	Newly-annotated lichen genomes
cladonia.kurokawae.1_tyr218	cladonia.kurokawae.1	cladonia.kurokawae.1_111	147111	148864	Newly-annotated lichen genomes
lecanorales.sp..6_tyr3950	lecanorales.sp..6	lecanorales.sp..6_1226	235	1768	Newly-annotated lichen genomes
psora.testacea.1_tyr2544	psora.testacea.1	psora.testacea.1_338	7180	9182	Newly-annotated lichen genomes
psora.testacea.1_tyr1362	psora.testacea.1	psora.testacea.1_211	2481	4706	Newly-annotated lichen genomes
bulbothrix.scortella.1_tyr3897	bulbothrix.scortella.1	bulbothrix.scortella.1_751	6193	8270	Newly-annotated lichen genomes
mycoblastus.sanguinarius.2_tyr1893	mycoblastus.sanguinarius.2	mycoblastus.sanguinarius.2_180	12783	15523	Newly-annotated lichen genomes
melanelia.culbersonii.1_tyr1605	melanelia.culbersonii.1	melanelia.culbersonii.1_295	26093	27253	Newly-annotated lichen genomes
peltigera.leucophlebia.1_tyr2605	peltigera.leucophlebia.1	peltigera.leucophlebia.1_2359	1274	2251	Newly-annotated lichen genomes
umbilicaria.crustulosa.2_tyr2752	umbilicaria.crustulosa.2	umbilicaria.crustulosa.2_4	1642952	1644075	Newly-annotated lichen genomes

umbilicaria.polyphylla.1_tyr2011	umbilicaria.polyphylla.1	umbilicaria.polyphylla.1_14	443162	444149	Newly-annotated lichen genomes	
umbilicaria.crustulosa.2_tyr1336	umbilicaria.crustulosa.2	umbilicaria.crustulosa.2_6	113198	114886	Newly-annotated lichen genomes	
umbilicaria.crustulosa.2_tyr1381	umbilicaria.crustulosa.2	umbilicaria.crustulosa.2_1	427784	429505	Newly-annotated lichen genomes	
umbilicaria.crustulosa.2_tyr2366	umbilicaria.crustulosa.2	umbilicaria.crustulosa.2_2	398661	400574	Newly-annotated lichen genomes	
puttea.exsequens.2_tyr973	puttea.exsequens.2	puttea.exsequens.2_4	331770	333923	Newly-annotated lichen genomes	
massalongia.carnosa.1_tyr3311	massalongia.carnosa.1	massalongia.carnosa.1_11	1168182	1169261	Newly-annotated lichen genomes	
melanelia.culbersonii.1_tyr901	melanelia.culbersonii.1	melanelia.culbersonii.1_807	11280	13376	Newly-annotated lichen genomes	
alectoria.sarmentosa.4_tyr2248	alectoria.sarmentosa.4	alectoria.sarmentosa.4_334	24167	26684	Newly-annotated lichen genomes	
gyalecta.farlowii.1_tyr3918	gyalecta.farlowii.1	gyalecta.farlowii.1_648	3263	4791	Newly-annotated lichen genomes	
puttea.exsequens.2_tyr972	puttea.exsequens.2	puttea.exsequens.2_4	454247	456271	Newly-annotated lichen genomes	
biatora.appalachensis.1_tyr3378	biatora.appalachensis.1	biatora.appalachensis.1_880	130	816	Newly-annotated lichen genomes	
gluame1_KAH0547751.1					Gluck-Thaler and Vogan (2024)	fam07
puttea.exsequens.1_tyr3752	puttea.exsequens.1	puttea.exsequens.1_55	10847	13215	Newly-annotated lichen genomes	
dirinaria.sp..2_tyr3979	dirinaria.sp..2	dirinaria.sp..2_4405	274	2793	Newly-annotated lichen genomes	
squamulea.squamosa.1_tyr3214	squamulea.squamosa.1	squamulea.squamosa.1_29	133862	136470	Newly-annotated lichen genomes	
xanthoria.mediterranea.1_tyr1163	xanthoria.mediterranea.1	xanthoria.mediterranea.1_131	39313	41617	Newly-annotated lichen genomes	
XpGTX0501_010485-T1					Gluck-Thaler and Vogan (2024)	
OZ2100_008954-T1					Gluck-Thaler and Vogan (2024)	
thelotrema.lepadinum.1_tyr2472	thelotrema.lepadinum.1	thelotrema.lepadinum.1_57	905	3082	Newly-annotated lichen genomes	
gomphillus.americanus.2_tyr1204	gomphillus.americanus.2	gomphillus.americanus.2_5	411169	412412	Newly-annotated lichen genomes	
gomame1_CAF9912407.1					Gluck-Thaler and Vogan (2024)	fam07

gomphillus.calycioides.1_tyr3850	gomphillus.calycioides.1	gomphillus.calycioides.1_345	12366	14687	Newly-annotated lichen genomes	
gomame1_CAF9918205.1					Gluck-Thaler and Vogan (2024)	fam07
agyrium.rufum.1_tyr1948	agyrium.rufum.1	agyrium.rufum.1_113	129501	131651	Newly-annotated lichen genomes	
gomphillus.americanus.1_tyr3818	gomphillus.americanus.1	gomphillus.americanus.1_9	49315	51226	Newly-annotated lichen genomes	
gomame1_CAF9914209.1					Gluck-Thaler and Vogan (2024)	fam07
Galactica_Afla-Captain					Gluck-Thaler and Vogan (2024)	fam07
asppse8_286872					Gluck-Thaler and Vogan (2024)	fam07
asplep2_tyr1813					Gluck-Thaler and Vogan (2024)	fam07
aspche2_tyr868					Gluck-Thaler and Vogan (2024)	fam07
aspfum5_EAL87801.2					Gluck-Thaler and Vogan (2024)	fam07
Starship-1_Asoj-Captain					Gluck-Thaler and Vogan (2024)	fam07
asppse8_278758					Gluck-Thaler and Vogan (2024)	fam07
Starship-2_Pvar-Captain					Gluck-Thaler and Vogan (2024)	fam07
aspacu4_tyr350					Gluck-Thaler and Vogan (2024)	fam07
aspell2_tyr1081					Gluck-Thaler and Vogan (2024)	fam07
aspcar1_tyr828					Gluck-Thaler and Vogan (2024)	fam07
aspcla3_EAW09702.1					Gluck-Thaler and Vogan (2024)	fam07
aspoli1_tyr2253					Gluck-Thaler and Vogan (2024)	fam07
aspfum5_EAL87819.1_EAL87820.1_EAL87821.1					Gluck-Thaler and Vogan (2024)	n.d.
aspthe1_tyr2953					Gluck-Thaler and Vogan (2024)	fam07

aspasp3_tyr578				Gluck-Thaler and Vogan (2024)	fam07
aspche2_tyr882				Gluck-Thaler and Vogan (2024)	fam07
aspqua1_tyr2559				Gluck-Thaler and Vogan (2024)	fam07
aspaff1_tyr365				Gluck-Thaler and Vogan (2024)	fam07
aspter10_tyr2883				Gluck-Thaler and Vogan (2024)	fam07
aspust1_tyr3097				Gluck-Thaler and Vogan (2024)	fam07
aspamo1_tyr433				Gluck-Thaler and Vogan (2024)	fam07
aspnan1_KAF9890711.1				Gluck-Thaler and Vogan (2024)	fam07
aspcri1_6849				Gluck-Thaler and Vogan (2024)	fam07
aspcri5_tyr1036				Gluck-Thaler and Vogan (2024)	fam07
aspgla1_OJJ85450.1_OJJ85451.1				Gluck-Thaler and Vogan (2024)	n.d.
aspmel1_tyr1930				Gluck-Thaler and Vogan (2024)	fam07
aspscl5_tyr2626				Gluck-Thaler and Vogan (2024)	fam07
aspara2_tyr467				Gluck-Thaler and Vogan (2024)	fam07
penant1_11477				Gluck-Thaler and Vogan (2024)	fam07
penrub1_CAP95304.1_CAP95306.1				Gluck-Thaler and Vogan (2024)	n.d.
penari1_5287				Gluck-Thaler and Vogan (2024)	fam07
penthy1_187711				Gluck-Thaler and Vogan (2024)	fam07
penbra1_9248				Gluck-Thaler and Vogan (2024)	fam07
penfre1_tyr8819				Gluck-Thaler and Vogan (2024)	fam07

pencam1_3517					Gluck-Thaler and Vogan (2024)	fam07
penrub2_KAF3027235.1					Gluck-Thaler and Vogan (2024)	fam07
pensp.9_KAF4765341.1					Gluck-Thaler and Vogan (2024)	fam07
paevar1_289487					Gluck-Thaler and Vogan (2024)	fam07
solorina.crocea.1_tyr2839	solorina.crocea.1	solorina.crocea.1_280	24858	27157	Newly-annotated lichen genomes	
aspasp6_tyr598					Gluck-Thaler and Vogan (2024)	fam07
aspasp6_tyr601					Gluck-Thaler and Vogan (2024)	fam07
aspsyd4_tyr2794					Gluck-Thaler and Vogan (2024)	fam07
aspver1_OJI99576.1					Gluck-Thaler and Vogan (2024)	fam07
aspsyd4_tyr2807					Gluck-Thaler and Vogan (2024)	fam07
aspscl4_tyr2611					Gluck-Thaler and Vogan (2024)	fam07
aspthe1_tyr2959					Gluck-Thaler and Vogan (2024)	fam07
asphir2_tyr1515					Gluck-Thaler and Vogan (2024)	fam07
aspnan1_KAF9885275.1					Gluck-Thaler and Vogan (2024)	fam07
paeniv1_tyr8569					Gluck-Thaler and Vogan (2024)	fam07
raseme1_KKA23990.1					Gluck-Thaler and Vogan (2024)	fam07
pectenia.cyanoloma.1_tyr365	pectenia.cyanoloma.1	pectenia.cyanoloma.1_46	48518	50366	Newly-annotated lichen genomes	
leptogium.corticola.1_tyr1821	leptogium.corticola.1	leptogium.corticola.1_392	7871	10283	Newly-annotated lichen genomes	
endocarpon.pusillum.1_tyr2774	endocarpon.pusillum.1	endocarpon.pusillum.1_856	40703	45099	Newly-annotated lichen genomes	
endpus1_8492					Gluck-Thaler and Vogan (2024)	fam07

phlyctis.petraea.1_tyr677	phlyctis.petraea.1	phlyctis.petraea.1_86	7590	9386	Newly-annotated lichen genomes	
umbilicaria.crustulosa.3_tyr2056	umbilicaria.crustulosa.3	umbilicaria.crustulosa.3_2	1906709	1909451	Newly-annotated lichen genomes	
umbilicaria.muhlenbergii.1_tyr4002	umbilicaria.muhlenbergii.1	umbilicaria.muhlenbergii.1_2	2643121	2646240	Newly-annotated lichen genomes	
dimelaena.oreina.1_tyr4063	dimelaena.oreina.1	dimelaena.oreina.1_1155	753	4674	Newly-annotated lichen genomes	
chasp.4_RMD40828.1					Gluck-Thaler and Vogan (2024)	clade02
chasp.7_RMZ90284.1					Gluck-Thaler and Vogan (2024)	clade02
teloschistes.exilis.3_tyr1430	teloschistes.exilis.3	teloschistes.exilis.3_1034	2441	3774	Newly-annotated lichen genomes	
teloschistes.exilis.2_tyr3635	teloschistes.exilis.2	teloschistes.exilis.2_108	25505	26498	Newly-annotated lichen genomes	
teloschistes.exilis.3_tyr2165	teloschistes.exilis.3	teloschistes.exilis.3_827	7588	9590	Newly-annotated lichen genomes	
brigantiaea.leucoxantha.1_tyr3736	brigantiaea.leucoxantha.1	brigantiaea.leucoxantha.1_563	13347	15305	Newly-annotated lichen genomes	
lecanora.subimmergens.1_tyr3208	lecanora.subimmergens.1	lecanora.subimmergens.1_4	13664	15636	Newly-annotated lichen genomes	
parmelia.squarrosa.1_tyr1871	parmelia.squarrosa.1	parmelia.squarrosa.1_1078	77	2122	Newly-annotated lichen genomes	
letharia.lupina.1_tyr2135	letharia.lupina.1	letharia.lupina.1_23	2293602	2294496	Newly-annotated lichen genomes	
letharia.vulpina.1_tyr3504	letharia.vulpina.1	letharia.vulpina.1_38	112445	113715	Newly-annotated lichen genomes	
stictis.urceolata.2_tyr2470	stictis.urceolata.2	stictis.urceolata.2_1739	569	1411	Newly-annotated lichen genomes	
nadvornikia.sorediata.1_tyr544	nadvornikia.sorediata.1	nadvornikia.sorediata.1_2278	704	2515	Newly-annotated lichen genomes	
grascr1_11513					Gluck-Thaler and Vogan (2024)	fam06
grascr1_5194_5195					Gluck-Thaler and Vogan (2024)	n.d.
stictis.urceolata.1_tyr685	stictis.urceolata.1	stictis.urceolata.1_513	31417	33191	Newly-annotated lichen genomes	
gomphillus.calycioides.1_tyr2044	gomphillus.calycioides.1	gomphillus.calycioides.1_786	3757	5552	Newly-annotated lichen genomes	

fissurina.insidiosa.1_tyr2126	fissurina.insidiosa.1	fissurina.insidiosa.1_371	10641	12445	Newly-annotated lichen genomes	
aspasp11_tyr514					Gluck-Thaler and Vogan (2024)	fam06
aspter10_tyr2888					Gluck-Thaler and Vogan (2024)	fam06
asplen8_tyr1775					Gluck-Thaler and Vogan (2024)	fam06
aspgla1_OJJ81546.1					Gluck-Thaler and Vogan (2024)	fam06
aspfum5_tyr1395					Gluck-Thaler and Vogan (2024)	fam06
asplat7_tyr1723					Gluck-Thaler and Vogan (2024)	fam06
aspter10_tyr2885					Gluck-Thaler and Vogan (2024)	fam06
asplen8_tyr1773					Gluck-Thaler and Vogan (2024)	fam06
aspoch5_tyr2231					Gluck-Thaler and Vogan (2024)	fam06
aspnig5_tyr2095					Gluck-Thaler and Vogan (2024)	fam06
hiscap1_5928					Gluck-Thaler and Vogan (2024)	fam06
penbra1_3631					Gluck-Thaler and Vogan (2024)	fam06
penroq1_7137_7138					Gluck-Thaler and Vogan (2024)	n.d.
penthy1_174382					Gluck-Thaler and Vogan (2024)	fam06
aspcor3_tyr920					Gluck-Thaler and Vogan (2024)	fam06
asppuu1_tyr2548					Gluck-Thaler and Vogan (2024)	fam06
aspscl6_tyr2634					Gluck-Thaler and Vogan (2024)	fam06
aspfis5_tyr1170					Gluck-Thaler and Vogan (2024)	fam06
aspuda4_tyr3016					Gluck-Thaler and Vogan (2024)	fam06

asplat6_tyr1698					Gluck-Thaler and Vogan (2024)	fam06
aspnid1_CBF75942.1_CBF75944.1_CBF75946.1					Gluck-Thaler and Vogan (2024)	n.d.
aspche2_tyr892					Gluck-Thaler and Vogan (2024)	fam06
emmcre2_KKZ61741.1					Gluck-Thaler and Vogan (2024)	fam06
psesp.11_tyr9301					Gluck-Thaler and Vogan (2024)	fam06
psesp.14_KFY20107.1					Gluck-Thaler and Vogan (2024)	fam06
psesp.5_187					Gluck-Thaler and Vogan (2024)	fam06
psesp.20_KFY66944.1					Gluck-Thaler and Vogan (2024)	fam06
psesp.22_KFZ20555.1					Gluck-Thaler and Vogan (2024)	fam06
psesp.5_1992					Gluck-Thaler and Vogan (2024)	fam06
psesp.17_KFZ02619.1					Gluck-Thaler and Vogan (2024)	fam06
psesp.3_1735					Gluck-Thaler and Vogan (2024)	fam06
psesp.4_1827_1828					Gluck-Thaler and Vogan (2024)	n.d.
psesp.4_tyr9584					Gluck-Thaler and Vogan (2024)	fam06
psesp.5_712					Gluck-Thaler and Vogan (2024)	fam06
psesp.14_KFY31799.1					Gluck-Thaler and Vogan (2024)	fam06
psesp.15_KFY57135.1_KFY57136.1					Gluck-Thaler and Vogan (2024)	n.d.
psesp.4_tyr9579					Gluck-Thaler and Vogan (2024)	fam06
phlyctis.speirea.1_tyr496	phlyctis.speirea.1	phlyctis.speirea.1_157	10607	12586	Newly-annotated lichen genomes	
cyanodermella.asteris.1_tyr1868	cyanodermella.asteris.1	cyanodermella.asteris.1_28	416695	418706	Newly-annotated lichen genomes	

sticta.canariensis.1_tyr72	sticta.canariensis.1	sticta.canariensis.1_2998	9207	11476	Newly-annotated lichen genomes	
lepra.amara.1_tyr2731	lepra.amara.1	lepra.amara.1_1844	3521	5244	Newly-annotated lichen genomes	
icmadophila.ericetorum.1_tyr613	icmadophila.ericetorum.1	icmadophila.ericetorum.1_724	1997	3974	Newly-annotated lichen genomes	
lecanoromycetes.sp..3_tyr106	lecanoromycetes.sp..3	lecanoromycetes.sp..3_3150	810	2952	Newly-annotated lichen genomes	
ramalina.farinacea.1_tyr4179	ramalina.farinacea.1	ramalina.farinacea.1_8	744403	746136	Newly-annotated lichen genomes	
bulbothrix.scortella.1_tyr1496	bulbothrix.scortella.1	bulbothrix.scortella.1_317	9417	10756	Newly-annotated lichen genomes	
parmotrema.hypotropum.1_tyr1608	parmotrema.hypotropum.1	parmotrema.hypotropum.1_1734	1202	2204	Newly-annotated lichen genomes	
mycoblastus.sanguinarius.1_tyr806	mycoblastus.sanguinarius.1	mycoblastus.sanguinarius.1_944	7965	9790	Newly-annotated lichen genomes	
puttea.exsequens.1_tyr4125	puttea.exsequens.1	puttea.exsequens.1_7	648	2443	Newly-annotated lichen genomes	
melanohalea.halei.1_tyr3440	melanohalea.halei.1	melanohalea.halei.1_2816	730	2238	Newly-annotated lichen genomes	
alectoria.sarmentosa.3_tyr1179	alectoria.sarmentosa.3	alectoria.sarmentosa.3_20	199184	201597	Newly-annotated lichen genomes	
alefal1_CAF9915422.1					Gluck-Thaler and Vogan (2024)	fam06
aspthe1_tyr2958					Gluck-Thaler and Vogan (2024)	fam06
buellia.spuria.2_tyr979	buellia.spuria.2	buellia.spuria.2_1577	3347	6689	Newly-annotated lichen genomes	
exomes1_113733_113734_113735_113736_113737_113738					Gluck-Thaler and Vogan (2024)	n.d.
amyenc1_432340_521054					Gluck-Thaler and Vogan (2024)	n.d.
aspasp7_tyr618					Gluck-Thaler and Vogan (2024)	fam06
gluame1_KAH0541585.1					Gluck-Thaler and Vogan (2024)	fam06
punctelia.appalachensis.1_tyr157	punctelia.appalachensis.1	punctelia.appalachensis.1_626	4196	6172	Newly-annotated lichen genomes	
buellia.spuria.2_tyr1348	buellia.spuria.2	buellia.spuria.2_988	6386	8510	Newly-annotated lichen genomes	

punctelia.appalachensis.1_tyr2004	punctelia.appalachensis.1	punctelia.appalachensis.1_481	13886	15751	Newly-annotated lichen genomes	
porpidia.subsimplex.1_tyr2619	porpidia.subsimplex.1	porpidia.subsimplex.1_138	262	1453	Newly-annotated lichen genomes	
acarospora.socialis.1_tyr3130	acarospora.socialis.1	acarospora.socialis.1_14	162470	164674	Newly-annotated lichen genomes	
aspicilia.laevata.1_tyr3361	aspicilia.laevata.1	aspicilia.laevata.1_963	6737	8810	Newly-annotated lichen genomes	
laccf.1_1280066					Gluck-Thaler and Vogan (2024)	fam06
phlsp.1_1968385					Gluck-Thaler and Vogan (2024)	fam06
phlsp.1_tyr9122					Gluck-Thaler and Vogan (2024)	fam06
rhoros1_675464_701269					Gluck-Thaler and Vogan (2024)	n.d.
cladonia.peziziformis.1_tyr751	cladonia.peziziformis.1	cladonia.peziziformis.1_2576	1463	2531	Newly-annotated lichen genomes	
tersp.5_KAG6901097.1					Gluck-Thaler and Vogan (2024)	fam06
tulsp.7_KAG8994973.1					Gluck-Thaler and Vogan (2024)	fam06
lecidea.sp..1_tyr2799	lecidea.sp..1	lecidea.sp..1_57	2170	4487	Newly-annotated lichen genomes	
grascr1_10749_10750					Gluck-Thaler and Vogan (2024)	n.d.
psevol1_tyr9664					Gluck-Thaler and Vogan (2024)	fam06
leosp.1_712301					Gluck-Thaler and Vogan (2024)	fam06
peltigera.hydrothyria.1_tyr591	peltigera.hydrothyria.1	peltigera.hydrothyria.1_1090	15091	16612	Newly-annotated lichen genomes	
diploschistes.diacapsis.1_tyr1354	diploschistes.diacapsis.1	diploschistes.diacapsis.1_802	1683	3642	Newly-annotated lichen genomes	
lecidea.sp..1_tyr684	lecidea.sp..1	lecidea.sp..1_1261	3092	4800	Newly-annotated lichen genomes	
phlyctis.petraea.1_tyr1522	phlyctis.petraea.1	phlyctis.petraea.1_622	13128	14918	Newly-annotated lichen genomes	
umbilicaria.mammulata.1_tyr624	umbilicaria.mammulata.1	umbilicaria.mammulata.1_1348	3103	5579	Newly-annotated lichen genomes	

phlyctis.petraea.1_tyr3168	phlyctis.petraea.1	phlyctis.petraea.1_602	1756	3309	Newly-annotated lichen genomes	
stereocaulon.virgatum.1_tyr4122	stereocaulon.virgatum.1	stereocaulon.virgatum.1_113	423854	425876	Newly-annotated lichen genomes	
Starship-1_Llup-Captain					Gluck-Thaler and Vogan (2024)	fam06
letlup1_KAF6222261.1					Gluck-Thaler and Vogan (2024)	fam06
lecanorales.sp..2_tyr2300	lecanorales.sp..2	lecanorales.sp..2_16	38176	41008	Newly-annotated lichen genomes	
umbilicaria.vellea.1_tyr1644	umbilicaria.vellea.1	umbilicaria.vellea.1_272	5298	7164	Newly-annotated lichen genomes	
umbilicaria.crustulosa.2_tyr1221	umbilicaria.crustulosa.2	umbilicaria.crustulosa.2_12	1318585	1320020	Newly-annotated lichen genomes	
umbilicaria.crustulosa.1_tyr446	umbilicaria.crustulosa.1	umbilicaria.crustulosa.1_8	243572	244582	Newly-annotated lichen genomes	
pilophorus.fibula.1_tyr2273	pilophorus.fibula.1	pilophorus.fibula.1_76	17627	19884	Newly-annotated lichen genomes	
umbilicaria.deusta.2_tyr762	umbilicaria.deusta.2	umbilicaria.deusta.2_1	275379	279030	Newly-annotated lichen genomes	
diploschistes.diacapsis.1_tyr1051	diploschistes.diacapsis.1	diploschistes.diacapsis.1_1757	7724	9254	Newly-annotated lichen genomes	
exooli1_142270_142271					Gluck-Thaler and Vogan (2024)	n.d.
bismed2_654338					Gluck-Thaler and Vogan (2024)	fam06
pocchl2_RZR64022.1					Gluck-Thaler and Vogan (2024)	fam06
tolpar1_tyr10471					Gluck-Thaler and Vogan (2024)	fam06
verlon1_10996_10997					Gluck-Thaler and Vogan (2024)	n.d.
xylbam1_587822					Gluck-Thaler and Vogan (2024)	fam06
xylpal1_613315					Gluck-Thaler and Vogan (2024)	fam06
psevex1_489986					Gluck-Thaler and Vogan (2024)	fam06
clorhi1_VUC09346.1					Gluck-Thaler and Vogan (2024)	fam06

mictri1_288833					Gluck-Thaler and Vogan (2024)	fam06
colaen1_KAF5522205.1					Gluck-Thaler and Vogan (2024)	fam06
colsco1_tyr4368					Gluck-Thaler and Vogan (2024)	fam06
talrug1_QKX61611.1					Gluck-Thaler and Vogan (2024)	fam06
blaper1_810					Gluck-Thaler and Vogan (2024)	fam06
talsti1_4992					Gluck-Thaler and Vogan (2024)	fam06
baeomycetales.sp..4_tyr1180	baeomycetales.sp..4	baeomycetales.sp..4_493	6584	8991	Newly-annotated lichen genomes	
Enterprise_Pans-Captain					Gluck-Thaler and Vogan (2024)	fam06
fusequ9_tyr5516					Gluck-Thaler and Vogan (2024)	fam06
fusfus65_tyr5879					Gluck-Thaler and Vogan (2024)	fam06
fustri7_tyr6779					Gluck-Thaler and Vogan (2024)	fam06
gaetri1_EJT68898.1					Gluck-Thaler and Vogan (2024)	fam06
gaetri1_EJT69642.1					Gluck-Thaler and Vogan (2024)	fam06
cudaci1_KAF4627340.1					Gluck-Thaler and Vogan (2024)	fam06
mictri1_249983					Gluck-Thaler and Vogan (2024)	fam06
endocarpon.pusillum.1_tyr2609	endocarpon.pusillum.1	endocarpon.pusillum.1_222	295282	297341	Newly-annotated lichen genomes	
fuscor2_tyr5424					Gluck-Thaler and Vogan (2024)	fam06
fushos2_tyr5966					Gluck-Thaler and Vogan (2024)	fam06
fussp.4_KAF9773401.1_KAF9773402.1					Gluck-Thaler and Vogan (2024)	n.d.
fusfus37_tyr5753					Gluck-Thaler and Vogan (2024)	fam06

fusfus35_tyr5744					Gluck-Thaler and Vogan (2024)	fam06
fusnem1_tyr6108					Gluck-Thaler and Vogan (2024)	fam06
phamin1_tyr9087					Gluck-Thaler and Vogan (2024)	fam06
Starship-2_Cpsa-Captain					Gluck-Thaler and Vogan (2024)	fam06
umbilicaria.iberica.1_tyr1168	umbilicaria.iberica.1	umbilicaria.iberica.1_19	1052981	1053947	Newly-annotated lichen genomes	
leucodecton.sp..1_tyr3879	leucodecton.sp..1	leucodecton.sp..1_69	36146	39005	Newly-annotated lichen genomes	
micmic1_140246					Gluck-Thaler and Vogan (2024)	fam06
rutsp.3_PQE10464.1_PQE10450.1					Gluck-Thaler and Vogan (2024)	n.d.
rhizocarpon.subgeminatum.1_tyr3407	rhizocarpon.subgeminatum.1	rhizocarpon.subgeminatum.1_110	21474	23289	Newly-annotated lichen genomes	
altgan2_tyr166					Gluck-Thaler and Vogan (2024)	fam06
mytres1_479663					Gluck-Thaler and Vogan (2024)	fam06
pesfic1_1296					Gluck-Thaler and Vogan (2024)	fam06
emeafr1_OAX79438.1					Gluck-Thaler and Vogan (2024)	fam06
helgri1_1499					Gluck-Thaler and Vogan (2024)	fam06
trimen1_4468					Gluck-Thaler and Vogan (2024)	fam06
trisou1_9405					Gluck-Thaler and Vogan (2024)	fam06
talrug1_QKX60504.1					Gluck-Thaler and Vogan (2024)	fam06
zoprhi1_KAF2195618.1					Gluck-Thaler and Vogan (2024)	fam06
micareopsis.irriguata.1_tyr1708	micareopsis.irriguata.1	micareopsis.irriguata.1_520	4935	6895	Newly-annotated lichen genomes	
micareopsis.irriguata.1_tyr4069	micareopsis.irriguata.1	micareopsis.irriguata.1_993	8458	9890	Newly-annotated lichen genomes	

cladonia.macilenta.1_tyr1721	cladonia.macilenta.1	cladonia.macilenta.1_518	14751	15977	Newly-annotated lichen genomes	
umbilicaria.deusta.1_tyr455	umbilicaria.deusta.1	umbilicaria.deusta.1_6	170882	172680	Newly-annotated lichen genomes	
gluame1_tyr7131					Gluck-Thaler and Vogan (2024)	fam06
stictis.urceolata.2_tyr2769	stictis.urceolata.2	stictis.urceolata.2_394	20586	23082	Newly-annotated lichen genomes	
corcas2_PSN61909.1					Gluck-Thaler and Vogan (2024)	fam06
coltof2_tyr4409					Gluck-Thaler and Vogan (2024)	fam06
Starship-1_Fnub-Captain					Gluck-Thaler and Vogan (2024)	fam06
cypeur1_118335					Gluck-Thaler and Vogan (2024)	fam06
chlaer1_TAQ83756.1					Gluck-Thaler and Vogan (2024)	fam06
gloste1_228597					Gluck-Thaler and Vogan (2024)	fam06
lacwil1_tyr7768					Gluck-Thaler and Vogan (2024)	fam06
rhieri1_658879					Gluck-Thaler and Vogan (2024)	fam06
psesp.2_9155					Gluck-Thaler and Vogan (2024)	fam06
psesp.5_10696					Gluck-Thaler and Vogan (2024)	fam06
psesp.8_7699					Gluck-Thaler and Vogan (2024)	fam06
psesp.4_1253_1254					Gluck-Thaler and Vogan (2024)	n.d.
fonere1_5544_5545					Gluck-Thaler and Vogan (2024)	n.d.
consp.1_1014526					Gluck-Thaler and Vogan (2024)	fam06
xylcf.1_280771_507006					Gluck-Thaler and Vogan (2024)	n.d.
xyltel1_515037_515045					Gluck-Thaler and Vogan (2024)	n.d.

fusalb1_KAF4458660.1					Gluck-Thaler and Vogan (2024)	fam06
magory1_2081_2080					Gluck-Thaler and Vogan (2024)	n.d.
pyrsp.2_TLD09608.1_TLD09609.1					Gluck-Thaler and Vogan (2024)	n.d.
pyrpen1_TLS25962.1					Gluck-Thaler and Vogan (2024)	fam06
fuscor2_tyr5423					Gluck-Thaler and Vogan (2024)	fam06
fusfus25_tyr5667					Gluck-Thaler and Vogan (2024)	fam06
fusmet1_tyr6076					Gluck-Thaler and Vogan (2024)	fam06
purlil1_tyr9695					Gluck-Thaler and Vogan (2024)	fam06
ilysp.1_592848_824996					Gluck-Thaler and Vogan (2024)	n.d.
stalon1_KAG7284893.1					Gluck-Thaler and Vogan (2024)	fam06
sceapi1_tyr10060					Gluck-Thaler and Vogan (2024)	fam06
acarospora.socialis.1_tyr3129	acarospora.socialis.1	acarospora.socialis.1_14	359000	362134	Newly-annotated lichen genomes	
xyldig1_515723					Gluck-Thaler and Vogan (2024)	fam06
ophpic1_tyr8517					Gluck-Thaler and Vogan (2024)	fam06
pyrory1_EHA47044.1					Gluck-Thaler and Vogan (2024)	fam06
pyrory3_ELQ32919.1					Gluck-Thaler and Vogan (2024)	fam06
pyrory1_EHA55112.1_EHA55113.1					Gluck-Thaler and Vogan (2024)	n.d.
acemac1_478033					Gluck-Thaler and Vogan (2024)	fam06
acemac1_585016					Gluck-Thaler and Vogan (2024)	fam06
chaetothyriales.sp..1_tyr1264	chaetothyriales.sp..1	chaetothyriales.sp..1_10	3112814	3114993	Newly-annotated lichen genomes	

cladophialophora.sp..1_tyr1003	cladophialophora.sp..1	cladophialophora.sp..1_5	5217013	5219376	Newly-annotated lichen genomes	
cladophialophora.sp..1_tyr1004	cladophialophora.sp..1	cladophialophora.sp..1_5	5007154	5008482	Newly-annotated lichen genomes	
cladophialophora.sp..1_tyr4136	cladophialophora.sp..1	cladophialophora.sp..1_14	4675150	4678368	Newly-annotated lichen genomes	
cladophialophora.sp..2_tyr400	cladophialophora.sp..2	cladophialophora.sp..2_10	3416696	3417421	Newly-annotated lichen genomes	
claimm1_tyr4064					Gluck-Thaler and Vogan (2024)	fam06
exooli1_137898					Gluck-Thaler and Vogan (2024)	fam06
cladophialophora.endolichena.1_tyr2203	cladophialophora.endolichena.1	cladophialophora.endolichena.1_1	3382789	3384663	Newly-annotated lichen genomes	
cypeur1_111219					Gluck-Thaler and Vogan (2024)	fam06
exooli1_137943_137944					Gluck-Thaler and Vogan (2024)	n.d.
aspasp2_tyr558					Gluck-Thaler and Vogan (2024)	fam06
aspibe2_tyr1544					Gluck-Thaler and Vogan (2024)	fam06
aspawa2_tyr663					Gluck-Thaler and Vogan (2024)	fam06
aspcej1_tyr842					Gluck-Thaler and Vogan (2024)	fam06
aspnid1_CBF82971.1					Gluck-Thaler and Vogan (2024)	fam06
aspspi1_tyr2725					Gluck-Thaler and Vogan (2024)	fam06
elagra2_tyr4827					Gluck-Thaler and Vogan (2024)	fam06
elagra2_tyr4839					Gluck-Thaler and Vogan (2024)	fam06
penexp2_2586					Gluck-Thaler and Vogan (2024)	fam06
hiscap1_5913					Gluck-Thaler and Vogan (2024)	fam06
polhys1_3689					Gluck-Thaler and Vogan (2024)	fam06

uncree2_EEP83037.1					Gluck-Thaler and Vogan (2024)	fam06
talmar2_KFX50176.1					Gluck-Thaler and Vogan (2024)	fam06
agyrium.rufum.1_tyr1892	agyrium.rufum.1	agyrium.rufum.1_10	57164	59145	Newly-annotated lichen genomes	
arthonia.susa.1_tyr48	arthonia.susa.1	arthonia.susa.1_10	25766	27939	Newly-annotated lichen genomes	
arthonia.radiata.1_tyr1680	arthonia.radiata.1	arthonia.radiata.1_14	1789879	1791298	Newly-annotated lichen genomes	
coniocarpon.cinnabarinum.1_tyr976	coniocarpon.cinnabarinum.1	coniocarpon.cinnabarinum.1_4	164741	166150	Newly-annotated lichen genomes	
vergal1_120558_120559					Gluck-Thaler and Vogan (2024)	n.d.
bipmay2_EMD97612.1					Gluck-Thaler and Vogan (2024)	clade02
lining1_19457					Gluck-Thaler and Vogan (2024)	clade03
polfus1_563474					Gluck-Thaler and Vogan (2024)	clade02
gluame1_KAH0542452.1					Gluck-Thaler and Vogan (2024)	clade02
orboli10_KAF3190727.1					Gluck-Thaler and Vogan (2024)	clade02
orboli20_KAF3221117.1					Gluck-Thaler and Vogan (2024)	clade02
orboli7_KAF3157311.1					Gluck-Thaler and Vogan (2024)	clade02
acemac1_681948					Gluck-Thaler and Vogan (2024)	fam08
ascsar1_9624					Gluck-Thaler and Vogan (2024)	fam08
venech1_RDL41173.1					Gluck-Thaler and Vogan (2024)	fam08
psever1_9422					Gluck-Thaler and Vogan (2024)	fam08
lacari1_tyr7740					Gluck-Thaler and Vogan (2024)	fam08
botcal1_tyr3614					Gluck-Thaler and Vogan (2024)	fam08

botcal1_tyr3618				Gluck-Thaler and Vogan (2024)	fam08
sclbor1_tyr10062				Gluck-Thaler and Vogan (2024)	fam08
monfru2_RAL60102.1				Gluck-Thaler and Vogan (2024)	fam08
monfru2_RAL66735.1				Gluck-Thaler and Vogan (2024)	fam08
monlax1_tyr8314				Gluck-Thaler and Vogan (2024)	fam08
sclcep1_KAF7877687.1				Gluck-Thaler and Vogan (2024)	fam08
botpor2_TGO90359.1				Gluck-Thaler and Vogan (2024)	fam08
cudaci1_KAF4629989.1				Gluck-Thaler and Vogan (2024)	fam08
lepsp.1_707367				Gluck-Thaler and Vogan (2024)	fam08
amyenc1_389645				Gluck-Thaler and Vogan (2024)	fam08
hymalb1_CAG8976161.1_CAG8976162.1				Gluck-Thaler and Vogan (2024)	n.d.
rutsp.2_tyr10042				Gluck-Thaler and Vogan (2024)	fam08
sclscl1_APA09337.1				Gluck-Thaler and Vogan (2024)	fam08
helsp.4_KAE8441678.1				Gluck-Thaler and Vogan (2024)	fam08
phisco1_737160_737161				Gluck-Thaler and Vogan (2024)	n.d.
phisub1_17707				Gluck-Thaler and Vogan (2024)	fam08
hyphym1_tyr7592				Gluck-Thaler and Vogan (2024)	fam08
lepsp.1_482229				Gluck-Thaler and Vogan (2024)	fam08
lacari1_tyr7741				Gluck-Thaler and Vogan (2024)	fam08
psever1_8338				Gluck-Thaler and Vogan (2024)	fam08

trihyb1_905659					Gluck-Thaler and Vogan (2024)	fam08
mycosphaerellales.sp..1_tyr2340	mycosphaerellales.sp..1	mycosphaerellales.sp..1_24	641563	643654	Newly-annotated lichen genomes	
cerkik1_GIZ36574.1					Gluck-Thaler and Vogan (2024)	fam10
zascel1_17254					Gluck-Thaler and Vogan (2024)	fam10
cerber1_4073					Gluck-Thaler and Vogan (2024)	fam10
cerbet1_PIA93163.1					Gluck-Thaler and Vogan (2024)	fam10
cerkik1_GIZ46179.1					Gluck-Thaler and Vogan (2024)	fam10
pasful1_KAH3649967.1_KAH3649832.1					Gluck-Thaler and Vogan (2024)	n.d.
disaci1_KAF1825875.1					Gluck-Thaler and Vogan (2024)	fam10
elsamp1_256687					Gluck-Thaler and Vogan (2024)	fam10
elsbat1_KAG8628150.1					Gluck-Thaler and Vogan (2024)	fam10
elsaus1_tyr4897					Gluck-Thaler and Vogan (2024)	fam10
elsbat1_KAG8623403.1_KAG8623701.1					Gluck-Thaler and Vogan (2024)	n.d.
elsaus2_TKX19097.1					Gluck-Thaler and Vogan (2024)	fam10
elsfaw1_KAF4556065.1					Gluck-Thaler and Vogan (2024)	fam10
aurpul6_KEQ83187.1_KEQ83188.1					Gluck-Thaler and Vogan (2024)	n.d.
delstr1_204501					Gluck-Thaler and Vogan (2024)	fam10
delstr1_369760					Gluck-Thaler and Vogan (2024)	fam10
delstr1_282815					Gluck-Thaler and Vogan (2024)	fam10
aaoarx1_319010					Gluck-Thaler and Vogan (2024)	fam10

maspha1_203713				Gluck-Thaler and Vogan (2024)	fam10
wesorn1_tyr10759				Gluck-Thaler and Vogan (2024)	fam10
didexi1_10501				Gluck-Thaler and Vogan (2024)	fam10
macano1_420158				Gluck-Thaler and Vogan (2024)	fam10
claely1_437966				Gluck-Thaler and Vogan (2024)	fam10
sethol1_15133				Gluck-Thaler and Vogan (2024)	fam10
stasp.1_299864				Gluck-Thaler and Vogan (2024)	fam10
lenflu1_KAF2680563.1				Gluck-Thaler and Vogan (2024)	fam10
cloaqu1_482612				Gluck-Thaler and Vogan (2024)	fam10
polfus1_408015				Gluck-Thaler and Vogan (2024)	fam10
polfus1_595134				Gluck-Thaler and Vogan (2024)	fam10
polfus1_594435_594441				Gluck-Thaler and Vogan (2024)	n.d.
parmin1_KAF9733691.1				Gluck-Thaler and Vogan (2024)	fam10
wesorn1_504387				Gluck-Thaler and Vogan (2024)	fam10
lenflu1_KAF2680670.1				Gluck-Thaler and Vogan (2024)	fam10
bimnov1_564002				Gluck-Thaler and Vogan (2024)	fam10
rhyruf1_112818				Gluck-Thaler and Vogan (2024)	fam10
gloste1_250357_290901				Gluck-Thaler and Vogan (2024)	n.d.
hyspul1_118805				Gluck-Thaler and Vogan (2024)	fam10
rhyruf1_tyr9994				Gluck-Thaler and Vogan (2024)	fam10

mytres1_483318				Gluck-Thaler and Vogan (2024)	fam10
mytres1_516471_225833				Gluck-Thaler and Vogan (2024)	n.d.
mytres1_569544				Gluck-Thaler and Vogan (2024)	fam10
rhyruf1_tyr9997				Gluck-Thaler and Vogan (2024)	fam10
Voyager_Mpha_mp102-Captain				Gluck-Thaler and Vogan (2024)	fam10
macpha1_756324				Gluck-Thaler and Vogan (2024)	fam10
neopar1_tyr8483				Gluck-Thaler and Vogan (2024)	fam10
neopar1_tyr8484				Gluck-Thaler and Vogan (2024)	fam10
hyspul1_120259				Gluck-Thaler and Vogan (2024)	fam10
patatr1_KAF2840256.1_KAF2840257.1				Gluck-Thaler and Vogan (2024)	n.d.
patatr1_tyr8666				Gluck-Thaler and Vogan (2024)	fam10
zoprhi1_KAF2178190.1				Gluck-Thaler and Vogan (2024)	fam10
Starship-1_Aalt-Captain				Gluck-Thaler and Vogan (2024)	fam10
Starship-1_Agai-Captain				Gluck-Thaler and Vogan (2024)	fam10
altgai1_tyr155				Gluck-Thaler and Vogan (2024)	fam10
Starship-1_Aten-Captain				Gluck-Thaler and Vogan (2024)	fam10
Starship-2_Aten-Captain				Gluck-Thaler and Vogan (2024)	fam10
altatr1_tyr121				Gluck-Thaler and Vogan (2024)	fam10
altatr2_tyr125				Gluck-Thaler and Vogan (2024)	fam10
lizemp1_118025				Gluck-Thaler and Vogan (2024)	fam10

pyrter3_EFQ93944.1				Gluck-Thaler and Vogan (2024)	fam10
pyrtri10_tyr9789				Gluck-Thaler and Vogan (2024)	fam10
pyrtri6_tyr9856				Gluck-Thaler and Vogan (2024)	fam10
pyrtri11_KAG9375895.1				Gluck-Thaler and Vogan (2024)	fam10
lizemp1_103933				Gluck-Thaler and Vogan (2024)	fam10
lizemp1_148783				Gluck-Thaler and Vogan (2024)	fam10
macano1_413805_348491				Gluck-Thaler and Vogan (2024)	n.d.
lizemp1_143644				Gluck-Thaler and Vogan (2024)	fam10
pletra1_KAF2849805.1				Gluck-Thaler and Vogan (2024)	fam10
claely1_463329				Gluck-Thaler and Vogan (2024)	fam10
coccar1_9885_111421				Gluck-Thaler and Vogan (2024)	n.d.
cocmiy1_41893_111066				Gluck-Thaler and Vogan (2024)	n.d.
maspha1_259090_259074				Gluck-Thaler and Vogan (2024)	n.d.
corcas2_PSN65677.1				Gluck-Thaler and Vogan (2024)	fam10
permac1_700065				Gluck-Thaler and Vogan (2024)	fam10
altgan2_tyr165				Gluck-Thaler and Vogan (2024)	fam10
delcon1_29688_29730_486811				Gluck-Thaler and Vogan (2024)	n.d.
racsp.1_tyr9897				Gluck-Thaler and Vogan (2024)	fam10
orboli10_KAF3191570.1				Gluck-Thaler and Vogan (2024)	fam08
orboli15_KAF3089738.1				Gluck-Thaler and Vogan (2024)	fam08

orboli19_KAF3231896.1					Gluck-Thaler and Vogan (2024)	fam08
orboli16_KAF3107618.1					Gluck-Thaler and Vogan (2024)	fam08
lacari1_tyr7736					Gluck-Thaler and Vogan (2024)	clade03
phisub1_17749					Gluck-Thaler and Vogan (2024)	clade03
zalvar1_678354					Gluck-Thaler and Vogan (2024)	clade03
lachya1_TVY25302.1					Gluck-Thaler and Vogan (2024)	clade02
rhesp.1_tyr9923					Gluck-Thaler and Vogan (2024)	clade02
psedes1_tyr9230					Gluck-Thaler and Vogan (2024)	clade03
psesp.4_4487_4488					Gluck-Thaler and Vogan (2024)	n.d.
psesp.12_tyr9318					Gluck-Thaler and Vogan (2024)	clade02
psesp.4_6788_6789					Gluck-Thaler and Vogan (2024)	n.d.
psesp.15_tyr9392					Gluck-Thaler and Vogan (2024)	clade03
psesp.16_KFY89749.1					Gluck-Thaler and Vogan (2024)	clade03
tubaes1_5336					Gluck-Thaler and Vogan (2024)	clade03
tubmag1_343032					Gluck-Thaler and Vogan (2024)	clade01
tubmag1_tyr10602					Gluck-Thaler and Vogan (2024)	clade02
delcon1_470772					Gluck-Thaler and Vogan (2024)	clade03
cladophialophora.endolichena.1_tyr8	cladophialophora.endolichena.1	cladophialophora.endolichena.1_5	6550313	6552743	Newly-annotated lichen genomes	
melanina.sp..2_tyr2115	melanina.sp..2	melanina.sp..2_15	26265	27817	Newly-annotated lichen genomes	
claban1_134700					Gluck-Thaler and Vogan (2024)	fam04

lecanorales.sp..6_tyr1531	lecanorales.sp..6	lecanorales.sp..6_93	7398	8826	Newly-annotated lichen genomes	
umbilicaria.muhlenbergii.1_tyr3052	umbilicaria.muhlenbergii.1	umbilicaria.muhlenbergii.1_3	285805	288332	Newly-annotated lichen genomes	
pyrtri9_PZD23847.1					Gluck-Thaler and Vogan (2024)	fam04
lizemp1_160652					Gluck-Thaler and Vogan (2024)	fam04
lizemp1_186564					Gluck-Thaler and Vogan (2024)	fam04
pletra1_KAF2844130.1					Gluck-Thaler and Vogan (2024)	fam04
altgai1_tyr159					Gluck-Thaler and Vogan (2024)	fam04
didker1_KAF3049100.1					Gluck-Thaler and Vogan (2024)	fam04
cloaqu1_tyr4087					Gluck-Thaler and Vogan (2024)	fam04
polfus1_606100					Gluck-Thaler and Vogan (2024)	fam04
lenflu1_KAF2679993.1_KAF2679994.1					Gluck-Thaler and Vogan (2024)	n.d.
gyalolechia.ehrenbergii.1_tyr1689	gyalolechia.ehrenbergii.1	gyalolechia.ehrenbergii.1_265	144906	146904	Newly-annotated lichen genomes	
amnlig1_424037					Gluck-Thaler and Vogan (2024)	fam04
polfus1_599705					Gluck-Thaler and Vogan (2024)	fam04
verena1_584247					Gluck-Thaler and Vogan (2024)	fam04
zoprhi1_tyr10970					Gluck-Thaler and Vogan (2024)	fam04
bimnov1_554665_388559					Gluck-Thaler and Vogan (2024)	n.d.
bimnov1_60159_363215					Gluck-Thaler and Vogan (2024)	n.d.
lopmac1_KAF2650664.1					Gluck-Thaler and Vogan (2024)	fam04
permac1_607348_577860					Gluck-Thaler and Vogan (2024)	n.d.

moncan1_RYO85021.1					Gluck-Thaler and Vogan (2024)	fam04
cengeo1_542205					Gluck-Thaler and Vogan (2024)	fam04
cengeo1_589484_589527_521781					Gluck-Thaler and Vogan (2024)	n.d.
cengeo1_tyr3818					Gluck-Thaler and Vogan (2024)	fam04
cengeo1_566735					Gluck-Thaler and Vogan (2024)	fam04
cengeo1_591310					Gluck-Thaler and Vogan (2024)	fam04
cengeo1_tyr3807					Gluck-Thaler and Vogan (2024)	fam04
cengeo1_tyr3819					Gluck-Thaler and Vogan (2024)	fam04
leppal1_OCK79459.1					Gluck-Thaler and Vogan (2024)	fam04
leppal1_tyr7885					Gluck-Thaler and Vogan (2024)	fam04
pyrtri1_EDU47821.1					Gluck-Thaler and Vogan (2024)	fam04
chrysothrix.sp..1_tyr2503	chrysothrix.sp..1	chrysothrix.sp..1_182	34494	36172	Newly-annotated lichen genomes	
micareopsis.irriguata.1_tyr1039	micareopsis.irriguata.1	micareopsis.irriguata.1_1565	3860	5454	Newly-annotated lichen genomes	
icmadophila.ericetorum.1_tyr4094	icmadophila.ericetorum.1	icmadophila.ericetorum.1_539	5552	7630	Newly-annotated lichen genomes	
amyenc1_547861					Gluck-Thaler and Vogan (2024)	fam04
cadmal1_KAG4411212.1					Gluck-Thaler and Vogan (2024)	fam04
botpor2_tyr3657					Gluck-Thaler and Vogan (2024)	fam04
sclcep1_KAF7866863.1					Gluck-Thaler and Vogan (2024)	fam04
sclcep1_KAF7870603.1					Gluck-Thaler and Vogan (2024)	fam04
psesp.14_KFY31966.1					Gluck-Thaler and Vogan (2024)	fam04

psesp.14_tyr9380				Gluck-Thaler and Vogan (2024)	fam04
psesp.5_10301				Gluck-Thaler and Vogan (2024)	fam04
amores1_PSS18287.1				Gluck-Thaler and Vogan (2024)	fam04
oidmai1_93661				Gluck-Thaler and Vogan (2024)	fam04
cadsp.2_KAG4439199.1				Gluck-Thaler and Vogan (2024)	fam04
lepsp.1_529980_636753				Gluck-Thaler and Vogan (2024)	n.d.
rhesp.1_14923				Gluck-Thaler and Vogan (2024)	fam04
hymalb1_CAG8977711.1				Gluck-Thaler and Vogan (2024)	fam04
cyloli1_552812				Gluck-Thaler and Vogan (2024)	fam04
stacha2_KFA51963.1				Gluck-Thaler and Vogan (2024)	fam04
staele1_490642				Gluck-Thaler and Vogan (2024)	fam04
plecuc2_353822				Gluck-Thaler and Vogan (2024)	fam04
verdah2_tyr10676				Gluck-Thaler and Vogan (2024)	fam04
verlon1_20430				Gluck-Thaler and Vogan (2024)	fam04
monsp.2_RYP32009.1				Gluck-Thaler and Vogan (2024)	fam04
monsp.8_tyr8373				Gluck-Thaler and Vogan (2024)	fam04
madmyc1_9294				Gluck-Thaler and Vogan (2024)	fam04
monsp.4_RYP58605.1				Gluck-Thaler and Vogan (2024)	fam04
fussub5_tyr6719				Gluck-Thaler and Vogan (2024)	fam04
fustan1_tyr6732				Gluck-Thaler and Vogan (2024)	fam04

hirrho1_KAH0968669.1					Gluck-Thaler and Vogan (2024)	fam04
hirrho1_KAH0968736.1					Gluck-Thaler and Vogan (2024)	fam04
neodit1_2170					Gluck-Thaler and Vogan (2024)	fam04
leclec1_6727					Gluck-Thaler and Vogan (2024)	fam04
madmyc1_9889					Gluck-Thaler and Vogan (2024)	fam04
micbol1_109359					Gluck-Thaler and Vogan (2024)	fam04
pesfic1_5930					Gluck-Thaler and Vogan (2024)	fam04
xylcur2_595032					Gluck-Thaler and Vogan (2024)	fam04
helsp.4_KAE8442194.1					Gluck-Thaler and Vogan (2024)	fam04
oidmai1_137687					Gluck-Thaler and Vogan (2024)	fam04
trapelia.coarctata.1_tyr166	trapelia.coarctata.1	trapelia.coarctata.1_12	21672	22879	Newly-annotated lichen genomes	
trapelia.coarctata.1_tyr3016	trapelia.coarctata.1	trapelia.coarctata.1_80	10620	11770	Newly-annotated lichen genomes	
stictis.urceolata.1_tyr1403	stictis.urceolata.1	stictis.urceolata.1_220	2430	4486	Newly-annotated lichen genomes	
stictis.urceolata.2_tyr1426	stictis.urceolata.2	stictis.urceolata.2_1483	2308	4424	Newly-annotated lichen genomes	
umbilicaria.spodochroa.4_tyr2482	umbilicaria.spodochroa.4	umbilicaria.spodochroa.4_7	584965	586903	Newly-annotated lichen genomes	
gyalolechia.marmorata.1_tyr2287	gyalolechia.marmorata.1	gyalolechia.marmorata.1_133	18549	19448	Newly-annotated lichen genomes	
cladonia.diversa.2_tyr1126	cladonia.diversa.2	cladonia.diversa.2_145	18621	20672	Newly-annotated lichen genomes	
stereocaulon.virgatum.1_tyr1769	stereocaulon.virgatum.1	stereocaulon.virgatum.1_1	202993	204458	Newly-annotated lichen genomes	
stereocaulon.dactylophyllum.1_tyr2212	stereocaulon.dactylophyllum.1	stereocaulon.dactylophyllum.1_842	798	2132	Newly-annotated lichen genomes	
lecanoromycetes.sp..3_tyr3889	lecanoromycetes.sp..3	lecanoromycetes.sp..3_5310	3	1401	Newly-annotated lichen genomes	

flavoplaca.austrocitrina.1_tyr2357	flavoplaca.austrocitrina.1	flavoplaca.austrocitrina.1_4917	1501	2585	Newly-annotated lichen genomes	
heterodermia.speciosa.2_tyr3362	heterodermia.speciosa.2	heterodermia.speciosa.2_202	817	2850	Newly-annotated lichen genomes	
hetspe1_CAF9942110.1					Gluck-Thaler and Vogan (2024)	fam05
lecanoromycetes.sp..3_tyr978	lecanoromycetes.sp..3	lecanoromycetes.sp..3_2220	4508	5656	Newly-annotated lichen genomes	
ramalina.siliquosa.1_tyr3935	ramalina.siliquosa.1	ramalina.siliquosa.1_24	43034	44299	Newly-annotated lichen genomes	
massalongia.carnosa.1_tyr2628	massalongia.carnosa.1	massalongia.carnosa.1_8	2216479	2218149	Newly-annotated lichen genomes	
xylographa.bjoerkii.1_tyr2039	xylographa.bjoerkii.1	xylographa.bjoerkii.1_403	4530	5871	Newly-annotated lichen genomes	
solorina.crocea.1_tyr3472	solorina.crocea.1	solorina.crocea.1_923	20895	22610	Newly-annotated lichen genomes	
umbilicaria.iberica.1_tyr2631	umbilicaria.iberica.1	umbilicaria.iberica.1_15	1039649	1041708	Newly-annotated lichen genomes	
umbilicaria.polyphylla.3_tyr3786	umbilicaria.polyphylla.3	umbilicaria.polyphylla.3_6	496146	497191	Newly-annotated lichen genomes	
lecanorales.sp..5_tyr406	lecanorales.sp..5	lecanorales.sp..5_8	80606	82613	Newly-annotated lichen genomes	
nadvornikia.sorediata.1_tyr3380	nadvornikia.sorediata.1	nadvornikia.sorediata.1_742	6350	8338	Newly-annotated lichen genomes	
thelotrema.lepadinum.1_tyr3674	thelotrema.lepadinum.1	thelotrema.lepadinum.1_20	115988	116880	Newly-annotated lichen genomes	
circinaria.calcarea.1_tyr3668	circinaria.calcarea.1	circinaria.calcarea.1_363	595	2648	Newly-annotated lichen genomes	
psehyp1_505659					Gluck-Thaler and Vogan (2024)	fam05
teratosphaeriaceae.sp..1_tyr277	teratosphaeriaceae.sp..1	teratosphaeriaceae.sp..1_1	1923210	1925271	Newly-annotated lichen genomes	
teratosphaeriaceae.sp..1_tyr2580	teratosphaeriaceae.sp..1	teratosphaeriaceae.sp..1_15	1451836	1452817	Newly-annotated lichen genomes	
cerber1_tyr3938					Gluck-Thaler and Vogan (2024)	fam05
cerber1_6038					Gluck-Thaler and Vogan (2024)	fam05
teratosphaeriaceae.sp..1_tyr929	teratosphaeriaceae.sp..1	teratosphaeriaceae.sp..1_23	406676	408205	Newly-annotated lichen genomes	

frisim1_TKA68777.1					Gluck-Thaler and Vogan (2024)	fam05
frisim1_TKA79242.1					Gluck-Thaler and Vogan (2024)	fam05
racant1_OQN95408.1					Gluck-Thaler and Vogan (2024)	fam05
baupan1_EMC97231.1					Gluck-Thaler and Vogan (2024)	fam05
mycosphaerellales.sp..2_tyr758	mycosphaerellales.sp..2	mycosphaerellales.sp..2_4	3114842	3117164	Newly-annotated lichen genomes	
neoaci1_KAF2486406.1					Gluck-Thaler and Vogan (2024)	fam05
mycosphaerellales.sp..1_tyr2339	mycosphaerellales.sp..1	mycosphaerellales.sp..1_24	731792	732788	Newly-annotated lichen genomes	
neoaci1_KAF2478441.1_KAF2478442.1					Gluck-Thaler and Vogan (2024)	n.d.
racant1_OQN95439.1					Gluck-Thaler and Vogan (2024)	fam05
zascel1_30718					Gluck-Thaler and Vogan (2024)	fam05
zascel1_tyr10949					Gluck-Thaler and Vogan (2024)	fam05
ramalina.thrausta.1_tyr185	ramalina.thrausta.1	ramalina.thrausta.1_529	3265	4237	Newly-annotated lichen genomes	
ramalina.petrina.1_tyr3342	ramalina.petrina.1	ramalina.petrina.1_47	31202	32823	Newly-annotated lichen genomes	
protoblastenia.rupestris.2_tyr2177	protoblastenia.rupestris.2	protoblastenia.rupestris.2_721	8687	9665	Newly-annotated lichen genomes	
aspasp7_tyr614					Gluck-Thaler and Vogan (2024)	fam05
pensp.11_tyr8960					Gluck-Thaler and Vogan (2024)	fam05
talcel1_GAM41794.1					Gluck-Thaler and Vogan (2024)	fam05
talver1_KUL81733.1					Gluck-Thaler and Vogan (2024)	fam05
aspory7_tyr2327					Gluck-Thaler and Vogan (2024)	fam05
aspwen1_OJJ29631.1					Gluck-Thaler and Vogan (2024)	fam05

asppho1_RDK41012.1					Gluck-Thaler and Vogan (2024)	fam05
asptan1_tyr2879					Gluck-Thaler and Vogan (2024)	fam05
aspsoj2_tyr2667					Gluck-Thaler and Vogan (2024)	fam05
aspust1_tyr3098					Gluck-Thaler and Vogan (2024)	fam05
aulhed1_KAF1980648.1					Gluck-Thaler and Vogan (2024)	fam05
rhilig1_72239					Gluck-Thaler and Vogan (2024)	fam05
lasallia.pustulata.7_tyr482	lasallia.pustulata.7	lasallia.pustulata.7_3489	436749	438358	Newly-annotated lichen genomes	
lasallia.pustulata.1_tyr3965	lasallia.pustulata.1	lasallia.pustulata.1_41	1085618	1086795	Newly-annotated lichen genomes	
rhieri1_750159					Gluck-Thaler and Vogan (2024)	fam05
cudaci1_KAF4625152.1					Gluck-Thaler and Vogan (2024)	fam05
rutsp.1_PQE27284.1					Gluck-Thaler and Vogan (2024)	fam05
psesp.16_tyr9418					Gluck-Thaler and Vogan (2024)	fam05
psesp.18_KFY46141.1_KFY46142.1					Gluck-Thaler and Vogan (2024)	n.d.
blader5_EQL32851.1					Gluck-Thaler and Vogan (2024)	fam05
polhys1_6343					Gluck-Thaler and Vogan (2024)	fam05
blaper1_3939					Gluck-Thaler and Vogan (2024)	fam05
emepas2_OJD09878.1_OJD09879.1					Gluck-Thaler and Vogan (2024)	n.d.
emepas2_OJD10625.1					Gluck-Thaler and Vogan (2024)	fam05
hiscap4_ncRNA10775_ncRNA10776					Gluck-Thaler and Vogan (2024)	n.d.
parlut1_7852					Gluck-Thaler and Vogan (2024)	fam05

hiscap10_KAG5294641.1				Gluck-Thaler and Vogan (2024)	fam05
parbra1_2719				Gluck-Thaler and Vogan (2024)	fam05
parbra2_EEH48327.2				Gluck-Thaler and Vogan (2024)	fam05
hiscap10_KAG5292110.1_KAG5292111.1_KAG5292112.1				Gluck-Thaler and Vogan (2024)	n.d.
blaper1_5932				Gluck-Thaler and Vogan (2024)	fam05
emmcre2_KKZ61922.1				Gluck-Thaler and Vogan (2024)	fam05
aspasp12_tyr525				Gluck-Thaler and Vogan (2024)	fam05
aspnov3_tyr2189				Gluck-Thaler and Vogan (2024)	fam05
asplat4_tyr1663				Gluck-Thaler and Vogan (2024)	fam05
aspqua1_tyr2555				Gluck-Thaler and Vogan (2024)	fam05
asplep2_tyr1824				Gluck-Thaler and Vogan (2024)	fam05
aspnig13_tyr2056				Gluck-Thaler and Vogan (2024)	fam05
asppse7_288767_264632				Gluck-Thaler and Vogan (2024)	n.d.
aspfum12_tyr1266				Gluck-Thaler and Vogan (2024)	fam05
asphir2_tyr1511				Gluck-Thaler and Vogan (2024)	fam05
aspfla13_tyr1195				Gluck-Thaler and Vogan (2024)	fam05
penoxa1_6225				Gluck-Thaler and Vogan (2024)	fam05
penroq1_4089				Gluck-Thaler and Vogan (2024)	fam05
penrub1_CAP94151.1				Gluck-Thaler and Vogan (2024)	fam05
asphan1_tyr1478				Gluck-Thaler and Vogan (2024)	fam05

asplat5_tyr1682				Gluck-Thaler and Vogan (2024)	fam05
aspnid3_tyr2023				Gluck-Thaler and Vogan (2024)	fam05
aspqua1_tyr2553				Gluck-Thaler and Vogan (2024)	fam05
pesfic1_tyr9064				Gluck-Thaler and Vogan (2024)	fam05
blaper1_1788				Gluck-Thaler and Vogan (2024)	fam05
blaper1_3581				Gluck-Thaler and Vogan (2024)	fam05
parbra1_3446				Gluck-Thaler and Vogan (2024)	fam05
parbra2_EEH47566.2				Gluck-Thaler and Vogan (2024)	fam05
parbra2_KGM92451.1_KGM92452.1				Gluck-Thaler and Vogan (2024)	n.d.
emmcre2_KKZ63868.1_KKZ63869.1				Gluck-Thaler and Vogan (2024)	n.d.
penbra1_tyr8704				Gluck-Thaler and Vogan (2024)	fam05
penbra1_tyr8712				Gluck-Thaler and Vogan (2024)	fam05
talpin1_KAF3401258.1				Gluck-Thaler and Vogan (2024)	fam05
talmar2_KFX47975.1				Gluck-Thaler and Vogan (2024)	fam05
elagra2_5163_5164				Gluck-Thaler and Vogan (2024)	n.d.
elagra2_tyr4808				Gluck-Thaler and Vogan (2024)	fam05
aspter9_tyr2940				Gluck-Thaler and Vogan (2024)	fam05
psevol1_tyr9689				Gluck-Thaler and Vogan (2024)	fam05
blader3_7891_7892				Gluck-Thaler and Vogan (2024)	n.d.
cadsp.1_471581				Gluck-Thaler and Vogan (2024)	fam05

fuscopannaria.leucosticta.1_tyr648	fuscopannaria.leucosticta.1	fuscopannaria.leucosticta.1_2216	2489	4065	Newly-annotated lichen genomes	
altatr1_CAG5179564.1					Gluck-Thaler and Vogan (2024)	fam05
altlon1_tyr172					Gluck-Thaler and Vogan (2024)	fam05
bipmay2_EMD88483.1					Gluck-Thaler and Vogan (2024)	fam05
zoprhi1_KAF2184210.1					Gluck-Thaler and Vogan (2024)	fam05
zoprhi1_tyr11022					Gluck-Thaler and Vogan (2024)	fam05
linrhi1_417195					Gluck-Thaler and Vogan (2024)	fam05
treper1_542511					Gluck-Thaler and Vogan (2024)	fam05
xylhev1_246969_246970					Gluck-Thaler and Vogan (2024)	n.d.
lopnuc1_509612_608368_655764					Gluck-Thaler and Vogan (2024)	n.d.
flavoplaca.sp..1_tyr2392	flavoplaca.sp..1	flavoplaca.sp..1_926	7831	9998	Newly-annotated lichen genomes	
pseudevernia.furfuracea.3_tyr1235	pseudevernia.furfuracea.3	pseudevernia.furfuracea.3_17	527450	529232	Newly-annotated lichen genomes	
lachya1_TVY25215.1					Gluck-Thaler and Vogan (2024)	fam05
melbic1_tyr8098					Gluck-Thaler and Vogan (2024)	fam05
trihir1_KAH0563164.1_KAH0563163.1					Gluck-Thaler and Vogan (2024)	n.d.
lacari1_TVY13717.1					Gluck-Thaler and Vogan (2024)	fam05
acemac1_707798					Gluck-Thaler and Vogan (2024)	fam05
rhysec1_tyr10005					Gluck-Thaler and Vogan (2024)	fam05
cadsp.2_tyr3757					Gluck-Thaler and Vogan (2024)	fam05
lepsp.1_550109_673825					Gluck-Thaler and Vogan (2024)	n.d.

phisco1_133811					Gluck-Thaler and Vogan (2024)	fam05
rhesp.1_201867_520848_520851					Gluck-Thaler and Vogan (2024)	n.d.
rhesp.1_414138_414213					Gluck-Thaler and Vogan (2024)	n.d.
rhysec1_tyr10006					Gluck-Thaler and Vogan (2024)	fam05
calmar1_178488					Gluck-Thaler and Vogan (2024)	fam05
trihir1_KAH0563021.1					Gluck-Thaler and Vogan (2024)	fam05
lacari1_TVY12806.1					Gluck-Thaler and Vogan (2024)	fam05
lachya1_TVY22339.1					Gluck-Thaler and Vogan (2024)	fam05
oidmai1_62373_62374					Gluck-Thaler and Vogan (2024)	n.d.
peltigera.leucophlebia.1_tyr4120	peltigera.leucophlebia.1	peltigera.leucophlebia.1_2048	3311	4744	Newly-annotated lichen genomes	
calmar1_577054					Gluck-Thaler and Vogan (2024)	fam05
cengeo1_tyr3834					Gluck-Thaler and Vogan (2024)	fam05
cengeo1_tyr3835					Gluck-Thaler and Vogan (2024)	fam05
pseudopyrenochaetaceae.sp..1_tyr1768	pseudopyrenochaetaceae.sp..1	pseudopyrenochaetaceae.sp..1_8	1168147	1170268	Newly-annotated lichen genomes	
altarb7_tyr109					Gluck-Thaler and Vogan (2024)	fam05
altgai1_tyr158					Gluck-Thaler and Vogan (2024)	fam05
pyrtri5_tyr9846					Gluck-Thaler and Vogan (2024)	fam05
ascrab1_tyr338					Gluck-Thaler and Vogan (2024)	fam05
lizemp1_168641					Gluck-Thaler and Vogan (2024)	fam05
bipmay1_ENH98990.1					Gluck-Thaler and Vogan (2024)	fam05

cocmiy1_41658				Gluck-Thaler and Vogan (2024)	fam05
ascrab1_tyr340				Gluck-Thaler and Vogan (2024)	fam05
bimnov1_585611				Gluck-Thaler and Vogan (2024)	fam05
bimnov1_tyr3321				Gluck-Thaler and Vogan (2024)	fam05
byscir1_tyr3672				Gluck-Thaler and Vogan (2024)	fam05
cloaqu1_495597				Gluck-Thaler and Vogan (2024)	fam05
mytres1_375477				Gluck-Thaler and Vogan (2024)	fam05
mytres1_384697				Gluck-Thaler and Vogan (2024)	fam05
mytres1_388358_388424				Gluck-Thaler and Vogan (2024)	n.d.
mytres1_491224				Gluck-Thaler and Vogan (2024)	fam05
cloaqu1_tyr4081				Gluck-Thaler and Vogan (2024)	fam05
zoprhi1_KAF2184180.1_KAF2184181.1				Gluck-Thaler and Vogan (2024)	n.d.
lenflu1_KAF2676861.1				Gluck-Thaler and Vogan (2024)	fam05
leppal1_OCK73338.1				Gluck-Thaler and Vogan (2024)	fam05
gloste1_222527				Gluck-Thaler and Vogan (2024)	fam05
cengeo1_tyr3876				Gluck-Thaler and Vogan (2024)	fam05
linrhi1_396397				Gluck-Thaler and Vogan (2024)	fam05
totfus1_729837_333907_645645				Gluck-Thaler and Vogan (2024)	n.d.
virvir1_tyr10748				Gluck-Thaler and Vogan (2024)	fam05
Starship-2_Afla-Captain				Gluck-Thaler and Vogan (2024)	fam05

aspfum15_tyr1314				Gluck-Thaler and Vogan (2024)	fam05
aspscl6_tyr2635				Gluck-Thaler and Vogan (2024)	fam05
aspfum16_tyr1326				Gluck-Thaler and Vogan (2024)	fam05
asplen8_tyr1771				Gluck-Thaler and Vogan (2024)	fam05
aspoer1_tyr2236				Gluck-Thaler and Vogan (2024)	fam05
asplep2_tyr1825				Gluck-Thaler and Vogan (2024)	fam05
aspnid4_tyr2028				Gluck-Thaler and Vogan (2024)	fam05
pennor1_tyr8865				Gluck-Thaler and Vogan (2024)	fam05
raseme1_KKA16204.1				Gluck-Thaler and Vogan (2024)	fam05
blaper1_2381				Gluck-Thaler and Vogan (2024)	fam05
blaper1_9954				Gluck-Thaler and Vogan (2024)	fam05
hiscap1_tyr7335				Gluck-Thaler and Vogan (2024)	fam05
cocimm3_KMU92327.1_KMU92328.1				Gluck-Thaler and Vogan (2024)	n.d.
emmcre2_KKZ60871.1				Gluck-Thaler and Vogan (2024)	fam05
pensp.11_PCG88942.1				Gluck-Thaler and Vogan (2024)	fam05
Starship-1_Foxy_Fo5176-Captain				Gluck-Thaler and Vogan (2024)	fam05
fusequ4_tyr5487				Gluck-Thaler and Vogan (2024)	fam05
fusave8_tyr5272				Gluck-Thaler and Vogan (2024)	fam05
fusmus1_tyr6092				Gluck-Thaler and Vogan (2024)	fam05
fusfus65_tyr5881				Gluck-Thaler and Vogan (2024)	fam05

fusoxy29_474803				Gluck-Thaler and Vogan (2024)	fam05
fusinc2_tyr6003				Gluck-Thaler and Vogan (2024)	fam05
fususs4_tyr6846				Gluck-Thaler and Vogan (2024)	fam05
fuscer2_tyr5355				Gluck-Thaler and Vogan (2024)	fam05
fusequ5_tyr5495				Gluck-Thaler and Vogan (2024)	fam05
metmaj1_2504_2505				Gluck-Thaler and Vogan (2024)	n.d.
fusasi4_tyr5247				Gluck-Thaler and Vogan (2024)	fam05
fussp.5_KAF5611984.1				Gluck-Thaler and Vogan (2024)	fam05
fuscir3_tyr5363				Gluck-Thaler and Vogan (2024)	fam05
fuspro7_tyr6510				Gluck-Thaler and Vogan (2024)	fam05
fusste1_tyr6701				Gluck-Thaler and Vogan (2024)	fam05
fustup1_tyr6815				Gluck-Thaler and Vogan (2024)	fam05
fusfus29_tyr5699				Gluck-Thaler and Vogan (2024)	fam05
fusfus31_tyr5729				Gluck-Thaler and Vogan (2024)	fam05
dacmac1_659788				Gluck-Thaler and Vogan (2024)	fam05
beabas2_KGQ12276.1				Gluck-Thaler and Vogan (2024)	fam05
crypar1_KAF3768443.1				Gluck-Thaler and Vogan (2024)	fam05
diaamp1_10693				Gluck-Thaler and Vogan (2024)	fam05
diabat1_KAG8157681.1_KAG8157653.1				Gluck-Thaler and Vogan (2024)	n.d.
xylpal1_601133				Gluck-Thaler and Vogan (2024)	fam05

diaamp1_tyr4650				Gluck-Thaler and Vogan (2024)	fam05
xylsp.15_432324				Gluck-Thaler and Vogan (2024)	fam05
neocla1_KAF7521542.1				Gluck-Thaler and Vogan (2024)	fam05
xylcf.1_507826				Gluck-Thaler and Vogan (2024)	fam05
xylcub1_494674				Gluck-Thaler and Vogan (2024)	fam05
xylsp.2_355315				Gluck-Thaler and Vogan (2024)	fam05
diacit1_KAG6358485.1				Gluck-Thaler and Vogan (2024)	fam05
xyllon1_530278				Gluck-Thaler and Vogan (2024)	fam05
xylsp.15_427122				Gluck-Thaler and Vogan (2024)	fam05
xylsp.11_322789				Gluck-Thaler and Vogan (2024)	fam05
funsp.2_GAW15977.1				Gluck-Thaler and Vogan (2024)	fam05
xylgra1_359715				Gluck-Thaler and Vogan (2024)	fam05
xylcf.2_551827				Gluck-Thaler and Vogan (2024)	fam05
kredeu1_621827				Gluck-Thaler and Vogan (2024)	fam05
xylarb1_430476_471988				Gluck-Thaler and Vogan (2024)	n.d.
xylacu1_521853				Gluck-Thaler and Vogan (2024)	fam05
xylsp.9_38763_718660				Gluck-Thaler and Vogan (2024)	n.d.
xylcf.2_534528_534527				Gluck-Thaler and Vogan (2024)	n.d.
xyltel1_567555				Gluck-Thaler and Vogan (2024)	fam05
staele1_500552				Gluck-Thaler and Vogan (2024)	fam05

xylsp.18_tyr10859				Gluck-Thaler and Vogan (2024)	fam05
colcam1_tyr4276				Gluck-Thaler and Vogan (2024)	fam05
kredeu2_517727				Gluck-Thaler and Vogan (2024)	fam05
colspi1_tyr4386				Gluck-Thaler and Vogan (2024)	fam05
coltof2_11826_11827				Gluck-Thaler and Vogan (2024)	n.d.
xylsp.19_237375				Gluck-Thaler and Vogan (2024)	fam05
fusrus1_tyr6576				Gluck-Thaler and Vogan (2024)	fam05
hirrho1_KAH0961187.1				Gluck-Thaler and Vogan (2024)	fam05
toloph1_1610				Gluck-Thaler and Vogan (2024)	fam05
metbru1_tyr8161				Gluck-Thaler and Vogan (2024)	fam05
pocchl2_RZR61296.1				Gluck-Thaler and Vogan (2024)	fam05
toloph1_3948				Gluck-Thaler and Vogan (2024)	fam05
mictri1_425164				Gluck-Thaler and Vogan (2024)	fam05
artunc1_KAF3484272.1				Gluck-Thaler and Vogan (2024)	fam05
artunc1_KAF3492095.1				Gluck-Thaler and Vogan (2024)	fam05
aspara2_tyr470				Gluck-Thaler and Vogan (2024)	fam05
aspuda6_tyr3047				Gluck-Thaler and Vogan (2024)	fam05
aspflo1_tyr1229				Gluck-Thaler and Vogan (2024)	fam05
penroq1_3856				Gluck-Thaler and Vogan (2024)	fam05
aspwes1_tyr3212				Gluck-Thaler and Vogan (2024)	fam05

pencam1_9182_9183				Gluck-Thaler and Vogan (2024)	n.d.
pencam1_5738				Gluck-Thaler and Vogan (2024)	fam05
pennal1_11775				Gluck-Thaler and Vogan (2024)	fam05
penzon1_tyr9052				Gluck-Thaler and Vogan (2024)	fam05
paeniv1_62125				Gluck-Thaler and Vogan (2024)	fam05
aspasp7_tyr613				Gluck-Thaler and Vogan (2024)	fam05
paeniv1_60818				Gluck-Thaler and Vogan (2024)	fam05
fussp.6_KAF5007163.1				Gluck-Thaler and Vogan (2024)	fam05
talmar2_KFX41117.1_lncRNA0700070				Gluck-Thaler and Vogan (2024)	n.d.
talatr1_4403				Gluck-Thaler and Vogan (2024)	fam05
talsti1_5901				Gluck-Thaler and Vogan (2024)	fam05
talmar2_KFX41220.1				Gluck-Thaler and Vogan (2024)	fam05
cocimm1_tyr4125				Gluck-Thaler and Vogan (2024)	fam05
cocpos5_KMM68959.1				Gluck-Thaler and Vogan (2024)	fam05
uncree2_tyr10647				Gluck-Thaler and Vogan (2024)	fam05
emmcre2_KKZ61489.1				Gluck-Thaler and Vogan (2024)	fam05
hiscap1_tyr7384				Gluck-Thaler and Vogan (2024)	fam05
hiscap3_QSS69343.1_ncRNA10612				Gluck-Thaler and Vogan (2024)	n.d.
hiscap5_EER37235.1				Gluck-Thaler and Vogan (2024)	fam05
hiscap4_tyr7429				Gluck-Thaler and Vogan (2024)	fam05

triequ1_7282				Gluck-Thaler and Vogan (2024)	fam05
triton1_5425				Gluck-Thaler and Vogan (2024)	fam05
aspell2_tyr1085				Gluck-Thaler and Vogan (2024)	fam05
aspuda6_tyr3046				Gluck-Thaler and Vogan (2024)	fam05
aspnom1_125579_139535				Gluck-Thaler and Vogan (2024)	n.d.
aspory2_EIT83020.1_EIT83023.1				Gluck-Thaler and Vogan (2024)	n.d.
aspory7_tyr2325				Gluck-Thaler and Vogan (2024)	fam05
asppho1_RDK41021.1				Gluck-Thaler and Vogan (2024)	fam05
aspber2_KAE8379024.1				Gluck-Thaler and Vogan (2024)	fam05
eurrub1_405644_415988				Gluck-Thaler and Vogan (2024)	n.d.
pensp.9_tyr8981				Gluck-Thaler and Vogan (2024)	fam05
pensol2_2141_2142				Gluck-Thaler and Vogan (2024)	n.d.
asptam4_tyr2840				Gluck-Thaler and Vogan (2024)	fam05
blader3_tyr3427				Gluck-Thaler and Vogan (2024)	fam05
cocpos2_6003				Gluck-Thaler and Vogan (2024)	fam05
elagra2_2112				Gluck-Thaler and Vogan (2024)	fam05
aspasp2_tyr555				Gluck-Thaler and Vogan (2024)	fam05
asppar5_tyr2394				Gluck-Thaler and Vogan (2024)	fam05
asppar13_tyr2371				Gluck-Thaler and Vogan (2024)	fam05
aspnom1_155723				Gluck-Thaler and Vogan (2024)	fam05

aspter9_tyr2936					Gluck-Thaler and Vogan (2024)	fam05
pensub1_OKP09913.1_OKP09914.1					Gluck-Thaler and Vogan (2024)	n.d.
penthy1_208992					Gluck-Thaler and Vogan (2024)	fam05
talrug1_QKX60769.1					Gluck-Thaler and Vogan (2024)	fam05
blader5_EQL29727.1					Gluck-Thaler and Vogan (2024)	fam05
hiscap1_tyr7371					Gluck-Thaler and Vogan (2024)	fam05
lambiella.insularis.1_tyr3854	lambiella.insularis.1	lambiella.insularis.1_92	37812	39865	Newly-annotated lichen genomes	
botdot1_290492					Gluck-Thaler and Vogan (2024)	fam05
lasthe1_tyr7811					Gluck-Thaler and Vogan (2024)	fam05
lasthe3_KAB2568541.1					Gluck-Thaler and Vogan (2024)	fam05
coniocarpon.cinnabarinum.2_tyr169	coniocarpon.cinnabarinum.2	coniocarpon.cinnabarinum.2_522	1822	3620	Newly-annotated lichen genomes	
chrysothrix.sp..1_tyr3844	chrysothrix.sp..1	chrysothrix.sp..1_201	47006	49002	Newly-annotated lichen genomes	
coniocarpon.cinnabarinum.2_tyr3740	coniocarpon.cinnabarinum.2	coniocarpon.cinnabarinum.2_297	15358	17452	Newly-annotated lichen genomes	
cornicularia.normoerica.2_tyr176	cornicularia.normoerica.2	cornicularia.normoerica.2_1089	6114	7077	Newly-annotated lichen genomes	
letrouitia.transgressa.2_tyr670	letrouitia.transgressa.2	letrouitia.transgressa.2_74	28782	30014	Newly-annotated lichen genomes	
cladonia.uncialis.1_tyr1299	cladonia.uncialis.1	cladonia.uncialis.1_28	506878	507856	Newly-annotated lichen genomes	
cladonia.rangiferina.2_tyr2351	cladonia.rangiferina.2	cladonia.rangiferina.2_399	81562	83187	Newly-annotated lichen genomes	
pseudevernia.furfuracea.1_tyr327	pseudevernia.furfuracea.1	pseudevernia.furfuracea.1_7	493347	494212	Newly-annotated lichen genomes	
imsale1_tyr7676					Gluck-Thaler and Vogan (2024)	fam05
usnea.hakonensis.1_tyr1050	usnea.hakonensis.1	usnea.hakonensis.1_312	332	2121	Newly-annotated lichen genomes	

usnea.hakonensis.1_tyr513	usnea.hakonensis.1	usnea.hakonensis.1_373	1075	2157	Newly-annotated lichen genomes	
usnea.hakonensis.1_tyr3823	usnea.hakonensis.1	usnea.hakonensis.1_399	541	1317	Newly-annotated lichen genomes	
flavopunctelia.flaventior.1_tyr3604	flavopunctelia.flaventior.1	flavopunctelia.flaventior.1_127	7389	8811	Newly-annotated lichen genomes	
umbilicaria.hispanica.1_tyr1614	umbilicaria.hispanica.1	umbilicaria.hispanica.1_15	434276	436346	Newly-annotated lichen genomes	
trapelia.coarctata.2_tyr1443	trapelia.coarctata.2	trapelia.coarctata.2_1221	3245	4669	Newly-annotated lichen genomes	
clagra1_11292					Gluck-Thaler and Vogan (2024)	fam05
lecanoromycetes.sp..2_tyr232	lecanoromycetes.sp..2	lecanoromycetes.sp..2_1446	516	1528	Newly-annotated lichen genomes	
brianaria.bauschiana.1_tyr2644	brianaria.bauschiana.1	brianaria.bauschiana.1_1911	462	2583	Newly-annotated lichen genomes	
ramalina.siliquosa.1_tyr1191	ramalina.siliquosa.1	ramalina.siliquosa.1_26	890500	892528	Newly-annotated lichen genomes	
letrouitia.transgressa.1_tyr1836	letrouitia.transgressa.1	letrouitia.transgressa.1_21	683	2092	Newly-annotated lichen genomes	
porpidia.macrocarpa.1_tyr578	porpidia.macrocarpa.1	porpidia.macrocarpa.1_1220	8006	8956	Newly-annotated lichen genomes	
umbilicaria.crustulosa.1_tyr1108	umbilicaria.crustulosa.1	umbilicaria.crustulosa.1_2	1328972	1331034	Newly-annotated lichen genomes	
grascr1_10188					Gluck-Thaler and Vogan (2024)	fam05
pseudosagedia.sp..1_tyr3004	pseudosagedia.sp..1	pseudosagedia.sp..1_1180	743	2829	Newly-annotated lichen genomes	
dermiscellum.oulocheilum.1_tyr3730	dermiscellum.oulocheilum.1	dermiscellum.oulocheilum.1_1174	1451	3455	Newly-annotated lichen genomes	
umbilicaria.vellea.1_tyr4141	umbilicaria.vellea.1	umbilicaria.vellea.1_1236	15299	17611	Newly-annotated lichen genomes	
exooli1_139439_139440_139441_139442_139443					Gluck-Thaler and Vogan (2024)	n.d.
exosid1_tyr5117					Gluck-Thaler and Vogan (2024)	fam05
exoxen1_tyr5127					Gluck-Thaler and Vogan (2024)	fam05
exooli1_139457_139458_139459_139460					Gluck-Thaler and Vogan (2024)	n.d.

elagra2_6724					Gluck-Thaler and Vogan (2024)	fam05
endpal1_11393_11394					Gluck-Thaler and Vogan (2024)	n.d.
talpin1_KAF3392106.1					Gluck-Thaler and Vogan (2024)	fam05
talrug1_tyr10331					Gluck-Thaler and Vogan (2024)	fam05
solorina.crocea.1_tyr3630	solorina.crocea.1	solorina.crocea.1_1347	10608	12595	Newly-annotated lichen genomes	
endocarpon.pusillum.2_tyr3372	endocarpon.pusillum.2	endocarpon.pusillum.2_39	50463	52328	Newly-annotated lichen genomes	
muellerella.sp..1_tyr1789	muellerella.sp..1	muellerella.sp..1_3	170083	171682	Newly-annotated lichen genomes	
lambiella.insularis.1_tyr2509	lambiella.insularis.1	lambiella.insularis.1_53	398136	400493	Newly-annotated lichen genomes	
porpidia.macrocarpa.1_tyr3848	porpidia.macrocarpa.1	porpidia.macrocarpa.1_793	1385	2739	Newly-annotated lichen genomes	
acarospora.sp..1_tyr552	acarospora.sp..1	acarospora.sp..1_745	7357	10089	Newly-annotated lichen genomes	
erebil1_463208					Gluck-Thaler and Vogan (2024)	fam02
erebil1_472933_308236					Gluck-Thaler and Vogan (2024)	n.d.
HEPHAESTUS_Pvar-Captain					Gluck-Thaler and Vogan (2024)	fam02
aspasp7_tyr611					Gluck-Thaler and Vogan (2024)	fam02
asplep2_tyr1807					Gluck-Thaler and Vogan (2024)	fam02
aspasp3_tyr574					Gluck-Thaler and Vogan (2024)	fam02
emeafr1_OAX78725.1_OAX78726.1					Gluck-Thaler and Vogan (2024)	n.d.
penant1_1867_1868					Gluck-Thaler and Vogan (2024)	n.d.
Starship-1_Afel-Captain					Gluck-Thaler and Vogan (2024)	fam02
Starship-1_Afum-Captain					Gluck-Thaler and Vogan (2024)	fam02

aspfum4_KMK55507.1					Gluck-Thaler and Vogan (2024)	fam02
aspnig10_tyr2036					Gluck-Thaler and Vogan (2024)	fam02
aspche2_tyr890					Gluck-Thaler and Vogan (2024)	fam02
aspgla1_OJJ83717.1					Gluck-Thaler and Vogan (2024)	fam02
asplat6_tyr1695					Gluck-Thaler and Vogan (2024)	fam02
aspsyd2_tyr2767					Gluck-Thaler and Vogan (2024)	fam02
Starship-1_Tmar-Captain					Gluck-Thaler and Vogan (2024)	fam02
talver1_KUL81284.1					Gluck-Thaler and Vogan (2024)	fam02
talisl1_2669					Gluck-Thaler and Vogan (2024)	fam02
talsti1_3815_3816					Gluck-Thaler and Vogan (2024)	n.d.
aspaff1_tyr364					Gluck-Thaler and Vogan (2024)	fam02
aspbru4_tyr759					Gluck-Thaler and Vogan (2024)	fam02
blader3_11177					Gluck-Thaler and Vogan (2024)	fam02
cocimm1_628					Gluck-Thaler and Vogan (2024)	fam02
muellerella.sp..1_tyr1324	muellerella.sp..1	muellerella.sp..1_2	1230908	1233361	Newly-annotated lichen genomes	
evernia.prunastri.2_tyr1283	evernia.prunastri.2	evernia.prunastri.2_897	5926	8332	Newly-annotated lichen genomes	
peltigera.extenuata.1_tyr2566	peltigera.extenuata.1	peltigera.extenuata.1_381	23210	24003	Newly-annotated lichen genomes	
solorina.crocea.1_tyr2911	solorina.crocea.1	solorina.crocea.1_514	50758	52012	Newly-annotated lichen genomes	
gluame1_KAH0542861.1					Gluck-Thaler and Vogan (2024)	clade01
sticta.sp..1_tyr2883	sticta.sp..1	sticta.sp..1_1714	2946	4573	Newly-annotated lichen genomes	

opegrapha.moroziana.1_tyr1878	opegrapha.moroziana.1	opegrapha.moroziana.1_893	2578	4303	Newly-annotated lichen genomes	
arthonia.rubella.1_tyr714	arthonia.rubella.1	arthonia.rubella.1_3009	864	2631	Newly-annotated lichen genomes	
umbilicaria.crustulosa.2_tyr13	umbilicaria.crustulosa.2	umbilicaria.crustulosa.2_8	621938	624274	Newly-annotated lichen genomes	
stictis.urceolata.2_tyr390	stictis.urceolata.2	stictis.urceolata.2_902	9248	10905	Newly-annotated lichen genomes	
stictis.urceolata.1_tyr4160	stictis.urceolata.1	stictis.urceolata.1_301	849	2914	Newly-annotated lichen genomes	
porpidia.subsimplex.1_tyr2738	porpidia.subsimplex.1	porpidia.subsimplex.1_1194	1209	3375	Newly-annotated lichen genomes	
pertusaria.obruta.1_tyr2294	pertusaria.obruta.1	pertusaria.obruta.1_562	6194	7736	Newly-annotated lichen genomes	
lobaria.immixta.1_tyr2979	lobaria.immixta.1	lobaria.immixta.1_700	21179	22318	Newly-annotated lichen genomes	
lecidea.sp..1_tyr76	lecidea.sp..1	lecidea.sp..1_284	6256	7878	Newly-annotated lichen genomes	
guynec1_997316_937336					Gluck-Thaler and Vogan (2024)	n.d.
hypsp.2_644004					Gluck-Thaler and Vogan (2024)	fam01
leisp.1_1338902					Gluck-Thaler and Vogan (2024)	fam01
rusear1_1273445					Gluck-Thaler and Vogan (2024)	fam01
polsqu1_536378					Gluck-Thaler and Vogan (2024)	fam01
tepsp.1_KAG6829646.1_KAG6829647.1					Gluck-Thaler and Vogan (2024)	n.d.
consp.1_1039156					Gluck-Thaler and Vogan (2024)	fam01
crypar1_KAF3763489.1					Gluck-Thaler and Vogan (2024)	fam01
monsp.2_RYP22820.1					Gluck-Thaler and Vogan (2024)	fam01
consp.1_662629					Gluck-Thaler and Vogan (2024)	fam01
monsp.2_RYP34851.1					Gluck-Thaler and Vogan (2024)	fam01

monsp.3_RYP20312.1					Gluck-Thaler and Vogan (2024)	fam01
madmyc1_955					Gluck-Thaler and Vogan (2024)	fam01
spoins1_7186					Gluck-Thaler and Vogan (2024)	fam01
icmadophila.ericetorum.1_tyr1831	icmadophila.ericetorum.1	icmadophila.ericetorum.1_46	148459	150042	Newly-annotated lichen genomes	
fusver6_tyr6875					Gluck-Thaler and Vogan (2024)	fam01
fuscopannaria.leucosticta.1_tyr3928	fuscopannaria.leucosticta.1	fuscopannaria.leucosticta.1_110	14232	16251	Newly-annotated lichen genomes	
XcGTX0491_008785-T1					Gluck-Thaler and Vogan (2024)	
crydep1_ODN97795.1					Gluck-Thaler and Vogan (2024)	fam01
fusgad1_KAF4947046.1					Gluck-Thaler and Vogan (2024)	fam01
fustri4_tyr6766					Gluck-Thaler and Vogan (2024)	fam01
triasp2_28945					Gluck-Thaler and Vogan (2024)	fam01
moelib1_2937_2938					Gluck-Thaler and Vogan (2024)	n.d.
xylsp.2_523567_536667					Gluck-Thaler and Vogan (2024)	n.d.
monibe1_RYO96122.1					Gluck-Thaler and Vogan (2024)	fam01
hirmin1_tyr7282					Gluck-Thaler and Vogan (2024)	fam01
metacr1_8233					Gluck-Thaler and Vogan (2024)	fam01
metgui1_tyr8174					Gluck-Thaler and Vogan (2024)	fam01
valmal3_tyr10655					Gluck-Thaler and Vogan (2024)	fam01
durrog1_292270					Gluck-Thaler and Vogan (2024)	fam01
fusbur1_tyr5341					Gluck-Thaler and Vogan (2024)	fam01

stacha1_2942				Gluck-Thaler and Vogan (2024)	fam01
triaru1_7814				Gluck-Thaler and Vogan (2024)	fam01
triham1_3351				Gluck-Thaler and Vogan (2024)	fam01
moncan1_tyr8276				Gluck-Thaler and Vogan (2024)	fam01
monsp.3_tyr8335				Gluck-Thaler and Vogan (2024)	fam01
elagra2_tyr4880				Gluck-Thaler and Vogan (2024)	fam01
beabas1_7480				Gluck-Thaler and Vogan (2024)	fam01
metrob3_EFY94016.1				Gluck-Thaler and Vogan (2024)	fam01
trihar4_48485_396645				Gluck-Thaler and Vogan (2024)	n.d.
clorhi1_VUC06060.1				Gluck-Thaler and Vogan (2024)	fam01
fusbur1_tyr5345				Gluck-Thaler and Vogan (2024)	fam01
fusfus39_tyr5762				Gluck-Thaler and Vogan (2024)	fam01
fushae3_tyr5936				Gluck-Thaler and Vogan (2024)	fam01
fusfus28_tyr5695				Gluck-Thaler and Vogan (2024)	fam01
fusman2_tyr6048				Gluck-Thaler and Vogan (2024)	fam01
stacha2_KFA55155.1				Gluck-Thaler and Vogan (2024)	fam01
stacha3_KEY73861.1				Gluck-Thaler and Vogan (2024)	fam01
fusbra1_tyr5309				Gluck-Thaler and Vogan (2024)	fam01
fuskur1_RMI94325.1				Gluck-Thaler and Vogan (2024)	fam01
fussol2_tyr6622				Gluck-Thaler and Vogan (2024)	fam01

fusven3_tyr6863					Gluck-Thaler and Vogan (2024)	fam01
fustuc1_tyr6790					Gluck-Thaler and Vogan (2024)	fam01
fusoxy45_KAG7413195.1					Gluck-Thaler and Vogan (2024)	fam01
fusfus31_tyr5721					Gluck-Thaler and Vogan (2024)	fam01
fusfus24_tyr5659					Gluck-Thaler and Vogan (2024)	fam01
leclec1_6749					Gluck-Thaler and Vogan (2024)	fam01
dalsp.1_361367					Gluck-Thaler and Vogan (2024)	fam01
xylcf.1_526122					Gluck-Thaler and Vogan (2024)	fam01
fusfal1_tyr5526					Gluck-Thaler and Vogan (2024)	fam01
fusfus38_tyr5754					Gluck-Thaler and Vogan (2024)	fam01
golmag1_tyr7146					Gluck-Thaler and Vogan (2024)	fam01
colsoj1_KAF6812414.1					Gluck-Thaler and Vogan (2024)	fam01
dacmac1_34260					Gluck-Thaler and Vogan (2024)	fam01
micbol1_226188					Gluck-Thaler and Vogan (2024)	fam01
mictri1_300110					Gluck-Thaler and Vogan (2024)	fam01
mictri1_377953					Gluck-Thaler and Vogan (2024)	fam01
xylographa.bjoerkii.1_tyr163	xylographa.bjoerkii.1	xylographa.bjoerkii.1_163	25829	27746	Newly-annotated lichen genomes	
psedes1_5938					Gluck-Thaler and Vogan (2024)	fam01
psesp.11_OBT51882.1_OBT51880.1					Gluck-Thaler and Vogan (2024)	n.d.
psesp.13_KFX93134.1					Gluck-Thaler and Vogan (2024)	fam01

psesp.13_tyr9350				Gluck-Thaler and Vogan (2024)	fam01
psesp.11_OBT54041.1				Gluck-Thaler and Vogan (2024)	fam01
lepsp.1_tyr7902				Gluck-Thaler and Vogan (2024)	fam01
rhesp.1_195900				Gluck-Thaler and Vogan (2024)	fam01
parpol1_3559				Gluck-Thaler and Vogan (2024)	fam01
parpol1_tyr8652				Gluck-Thaler and Vogan (2024)	fam01
parpol1_tyr8651				Gluck-Thaler and Vogan (2024)	fam01
melbic1_634125				Gluck-Thaler and Vogan (2024)	fam01
cadsp.1_437647_620720_620728				Gluck-Thaler and Vogan (2024)	n.d.
cadsp.1_443130				Gluck-Thaler and Vogan (2024)	fam01
lepsp.1_451448				Gluck-Thaler and Vogan (2024)	fam01
phisub1_tyr9118				Gluck-Thaler and Vogan (2024)	fam01
rhesp.1_375297				Gluck-Thaler and Vogan (2024)	fam01
zalvar1_689006				Gluck-Thaler and Vogan (2024)	fam01
lepsp.1_560113_646570				Gluck-Thaler and Vogan (2024)	n.d.
lepsp.1_674126				Gluck-Thaler and Vogan (2024)	fam01
lepsp.1_739939				Gluck-Thaler and Vogan (2024)	fam01
leosp.1_206943_730274				Gluck-Thaler and Vogan (2024)	n.d.
lepsp.1_734986				Gluck-Thaler and Vogan (2024)	fam01
bothya1_tyr3649				Gluck-Thaler and Vogan (2024)	fam01

hypocenomyce.scalaris.3_tyr680	hypocenomyce.scalaris.3	hypocenomyce.scalaris.3_93	6779	9167	Newly-annotated lichen genomes	
leprocaulales.sp..1_tyr2957	leprocaulales.sp..1	leprocaulales.sp..1_2230	197	2828	Newly-annotated lichen genomes	
monfru1_KAA8574662.1					Gluck-Thaler and Vogan (2024)	fam01
monfru3_KAG4027417.1					Gluck-Thaler and Vogan (2024)	fam01
monlax1_KAB8302114.1					Gluck-Thaler and Vogan (2024)	fam01
solorina.crocea.1_tyr419	solorina.crocea.1	solorina.crocea.1_2709	4383	5954	Newly-annotated lichen genomes	
cadsp.1_440977_440967_440971_440978					Gluck-Thaler and Vogan (2024)	n.d.
lacari1_TVY17613.1_TVY17612.1					Gluck-Thaler and Vogan (2024)	n.d.
lachya1_TVY29336.1					Gluck-Thaler and Vogan (2024)	fam01
psesp.15_KFY65136.1					Gluck-Thaler and Vogan (2024)	fam01
venech1_RDL30427.1_RDL30426.1					Gluck-Thaler and Vogan (2024)	n.d.
scylig1_RFU28318.1					Gluck-Thaler and Vogan (2024)	fam01
zalvar1_538602					Gluck-Thaler and Vogan (2024)	fam01
amores1_PSS18505.1					Gluck-Thaler and Vogan (2024)	fam01
cudaci1_KAF4633412.1					Gluck-Thaler and Vogan (2024)	fam01
dacest1_591128_591130					Gluck-Thaler and Vogan (2024)	n.d.
phamin1_EOO01307.1					Gluck-Thaler and Vogan (2024)	fam01
stachl1_3593					Gluck-Thaler and Vogan (2024)	fam01
hirmin1_5623					Gluck-Thaler and Vogan (2024)	fam01
metacr1_9370					Gluck-Thaler and Vogan (2024)	fam01

metani1_5588					Gluck-Thaler and Vogan (2024)	fam01
metmaj1_1405					Gluck-Thaler and Vogan (2024)	fam01
metrob3_EFZ00634.1					Gluck-Thaler and Vogan (2024)	fam01
torhem1_1768					Gluck-Thaler and Vogan (2024)	fam01
fuscir3_tyr5364					Gluck-Thaler and Vogan (2024)	fam01
fusoxy42_ENH71189.1					Gluck-Thaler and Vogan (2024)	fam01
fusxyl4_KAG5801712.1					Gluck-Thaler and Vogan (2024)	fam01
fusxyl9_tyr7052					Gluck-Thaler and Vogan (2024)	fam01
fuscom2_tyr5400					Gluck-Thaler and Vogan (2024)	fam01
umbilicaria.spodochroa.3_tyr3907	umbilicaria.spodochroa.3	umbilicaria.spodochroa.3_18	612806	614876	Newly-annotated lichen genomes	
coltof2_tyr4412					Gluck-Thaler and Vogan (2024)	fam01
fusalb2_tyr5195					Gluck-Thaler and Vogan (2024)	fam01
moncan1_RYO82366.1					Gluck-Thaler and Vogan (2024)	fam01
fusvan1_EEU40131.1					Gluck-Thaler and Vogan (2024)	fam01
metgui1_4539					Gluck-Thaler and Vogan (2024)	fam01
dalgra1_449726					Gluck-Thaler and Vogan (2024)	fam01
xylsp.14_291070_291074					Gluck-Thaler and Vogan (2024)	n.d.
metrob3_EFZ04323.1					Gluck-Thaler and Vogan (2024)	fam01
isafum1_tyr7682					Gluck-Thaler and Vogan (2024)	fam01
dalgra1_438190					Gluck-Thaler and Vogan (2024)	fam01

kredeu2_484721					Gluck-Thaler and Vogan (2024)	fam01
kredeu2_tyr7716					Gluck-Thaler and Vogan (2024)	fam01
phicur1_TPX11403.1					Gluck-Thaler and Vogan (2024)	fam01
mictri1_377759					Gluck-Thaler and Vogan (2024)	fam01
spoins1_661					Gluck-Thaler and Vogan (2024)	fam01
spoins1_7220					Gluck-Thaler and Vogan (2024)	fam01
spobra1_10170_10169					Gluck-Thaler and Vogan (2024)	n.d.
monsp.8_RYP61190.1					Gluck-Thaler and Vogan (2024)	fam01
crypar1_KAF3763523.1					Gluck-Thaler and Vogan (2024)	fam01
variospora.velana.1_tyr1530	variospora.velana.1	variospora.velana.1_211	17899	20239	Newly-annotated lichen genomes	
botcon1_TGO45211.1_TGO45212.1					Gluck-Thaler and Vogan (2024)	n.d.
rutsp.2_PQE09269.1					Gluck-Thaler and Vogan (2024)	fam01
rutsp.2_PQE27491.1					Gluck-Thaler and Vogan (2024)	fam01
monfru1_KAA8565543.1					Gluck-Thaler and Vogan (2024)	fam01
rutsp.1_PQE11730.1					Gluck-Thaler and Vogan (2024)	fam01
rutsp.1_PQE28656.1					Gluck-Thaler and Vogan (2024)	fam01
rutsp.2_PQE07086.1					Gluck-Thaler and Vogan (2024)	fam01
sclcep1_KAF7865656.1					Gluck-Thaler and Vogan (2024)	fam01
psedes1_tyr9258					Gluck-Thaler and Vogan (2024)	fam01
psesp.4_tyr9585					Gluck-Thaler and Vogan (2024)	fam01

botacl1_KAF7955521.1					Gluck-Thaler and Vogan (2024)	fam01
botpae1_TGO22912.1					Gluck-Thaler and Vogan (2024)	fam01
icmadophila.ericetorum.1_tyr1705	icmadophila.ericetorum.1	icmadophila.ericetorum.1_599	8580	9599	Newly-annotated lichen genomes	
ptychographa.xylographoides.1_tyr2152	ptychographa.xylographoides.1	ptychographa.xylographoides.1_89	106292	108259	Newly-annotated lichen genomes	
buellia.spuria.1_tyr2629	buellia.spuria.1	buellia.spuria.1_946	1762	3111	Newly-annotated lichen genomes	
buellia.spuria.2_tyr1802	buellia.spuria.2	buellia.spuria.2_2092	2114	3282	Newly-annotated lichen genomes	
phlyctis.petraea.1_tyr3242	phlyctis.petraea.1	phlyctis.petraea.1_172	4234	5986	Newly-annotated lichen genomes	
rinodina.dolichospora.1_tyr2026	rinodina.dolichospora.1	rinodina.dolichospora.1_598	1343	3490	Newly-annotated lichen genomes	
lecanora.rugosella.1_tyr990	lecanora.rugosella.1	lecanora.rugosella.1_1219	2781	4916	Newly-annotated lichen genomes	
lecidea.sp..1_tyr236	lecidea.sp..1	lecidea.sp..1_200	7518	9364	Newly-annotated lichen genomes	
punctelia.appalachensis.1_tyr2219	punctelia.appalachensis.1	punctelia.appalachensis.1_191	13617	16064	Newly-annotated lichen genomes	
permac1_395901					Gluck-Thaler and Vogan (2024)	fam01
neopar1_6999					Gluck-Thaler and Vogan (2024)	fam01
endocarpon.pusillum.2_tyr2473	endocarpon.pusillum.2	endocarpon.pusillum.2_15	173106	175132	Newly-annotated lichen genomes	
endocarpon.pusillum.1_tyr3655	endocarpon.pusillum.1	endocarpon.pusillum.1_218	8821	10131	Newly-annotated lichen genomes	
endocarpon.pusillum.2_tyr1873	endocarpon.pusillum.2	endocarpon.pusillum.2_17	242829	245174	Newly-annotated lichen genomes	
botryolepraria.lesdainii.1_tyr1709	botryolepraria.lesdainii.1	botryolepraria.lesdainii.1_338	8590	10041	Newly-annotated lichen genomes	
zalvar1_685428					Gluck-Thaler and Vogan (2024)	fam01
aspasp2_tyr552					Gluck-Thaler and Vogan (2024)	fam01
paeniv1_69520					Gluck-Thaler and Vogan (2024)	fam01

penoxa1_tyr8890					Gluck-Thaler and Vogan (2024)	fam01
penrub2_KAF3021169.1					Gluck-Thaler and Vogan (2024)	fam01
leprocaulon.nicholsiae.1_tyr3801	leprocaulon.nicholsiae.1	leprocaulon.nicholsiae.1_1281	2432	4481	Newly-annotated lichen genomes	
chasp.7_RMZ87469.1					Gluck-Thaler and Vogan (2024)	fam01
exooli1_134015					Gluck-Thaler and Vogan (2024)	fam01
rhimac1_8905					Gluck-Thaler and Vogan (2024)	fam01
phachl1_4396					Gluck-Thaler and Vogan (2024)	fam01
Phoenix_Mpha_mp053-Captain					Gluck-Thaler and Vogan (2024)	fam01
macpha2_4023					Gluck-Thaler and Vogan (2024)	fam01
beabas1_10225					Gluck-Thaler and Vogan (2024)	fam01
metacr1_9181					Gluck-Thaler and Vogan (2024)	fam01
metrob3_tyr8212					Gluck-Thaler and Vogan (2024)	fam01
pocchl3_OAQ60539.2					Gluck-Thaler and Vogan (2024)	fam01
toloph1_tyr10453					Gluck-Thaler and Vogan (2024)	fam01
ilysp.2_681009					Gluck-Thaler and Vogan (2024)	fam01
metmaj1_10173					Gluck-Thaler and Vogan (2024)	fam01
podcom1_tyr9194					Gluck-Thaler and Vogan (2024)	fam01
tribis1_522931					Gluck-Thaler and Vogan (2024)	fam01
tribis1_534205_534208					Gluck-Thaler and Vogan (2024)	n.d.
teratosphaeriaceae.sp..1_tyr278	teratosphaeriaceae.sp..1	teratosphaeriaceae.sp..1_1	1617806	1619018	Newly-annotated lichen genomes	

friend1_TKA26544.1					Gluck-Thaler and Vogan (2024)	clade01
mycosphaerellales.sp..1_tyr1726	mycosphaerellales.sp..1	mycosphaerellales.sp..1_1	1359459	1360475	Newly-annotated lichen genomes	
aciric2_130832					Gluck-Thaler and Vogan (2024)	clade01
teratosphaeriaceae.sp..1_tyr2413	teratosphaeriaceae.sp..1	teratosphaeriaceae.sp..1_25	1206888	1208972	Newly-annotated lichen genomes	
racsp.1_tyr9896					Gluck-Thaler and Vogan (2024)	clade01
acisp.1_KXL50416.1					Gluck-Thaler and Vogan (2024)	clade01
pasful1_KAH3646784.1					Gluck-Thaler and Vogan (2024)	clade01
zascel1_66988					Gluck-Thaler and Vogan (2024)	clade01
chrysothrix.sp..1_tyr303	chrysothrix.sp..1	chrysothrix.sp..1_208	5665	7851	Newly-annotated lichen genomes	
zwackhia.viridis.1_tyr611	zwackhia.viridis.1	zwackhia.viridis.1_528	11431	13483	Newly-annotated lichen genomes	
virvir1_581593					Gluck-Thaler and Vogan (2024)	fam01
lecanora.albella.1_tyr331	lecanora.albella.1	lecanora.albella.1_770	10090	12369	Newly-annotated lichen genomes	
lecanora.strobilina.1_tyr1712	lecanora.strobilina.1	lecanora.strobilina.1_1156	3672	5865	Newly-annotated lichen genomes	
lecanora.subimmergens.1_tyr3478	lecanora.subimmergens.1	lecanora.subimmergens.1_62	33383	35433	Newly-annotated lichen genomes	
trapelia.coarctata.1_tyr378	trapelia.coarctata.1	trapelia.coarctata.1_193	1264	2583	Newly-annotated lichen genomes	
pleopsidium.flavum.1_tyr2871	pleopsidium.flavum.1	pleopsidium.flavum.1_3187	1	1426	Newly-annotated lichen genomes	
variospora.aurantia.1_tyr837	variospora.aurantia.1	variospora.aurantia.1_757	3695	4960	Newly-annotated lichen genomes	
mobergia.calculiformis.1_tyr3516	mobergia.calculiformis.1	mobergia.calculiformis.1_196	43637	45070	Newly-annotated lichen genomes	
phaeophyscia.squarrosa.1_tyr3355	phaeophyscia.squarrosa.1	phaeophyscia.squarrosa.1_949	10020	11618	Newly-annotated lichen genomes	
xanthoparmelia.verrucella.1_tyr431	xanthoparmelia.verrucella.1	xanthoparmelia.verrucella.1_25	416	2809	Newly-annotated lichen genomes	

gyalolechia.ehrenbergii.1_tyr2514	gyalolechia.ehrenbergii.1	gyalolechia.ehrenbergii.1_81	68743	70800	Newly-annotated lichen genomes	
gyalolechia.marmorata.1_tyr1245	gyalolechia.marmorata.1	gyalolechia.marmorata.1_114	24301	26224	Newly-annotated lichen genomes	
gyalolechia.gomerana.1_tyr3154	gyalolechia.gomerana.1	gyalolechia.gomerana.1_780	1584	3256	Newly-annotated lichen genomes	
diploschistes.diacapsis.1_tyr1681	diploschistes.diacapsis.1	diploschistes.diacapsis.1_3135	1786	2967	Newly-annotated lichen genomes	
porpidia.albocaerulescens.1_tyr3320	porpidia.albocaerulescens.1	porpidia.albocaerulescens.1_1024	8847	10896	Newly-annotated lichen genomes	
acarospora.socialis.1_tyr2216	acarospora.socialis.1	acarospora.socialis.1_1	583577	584299	Newly-annotated lichen genomes	
pleopsidium.flavum.1_tyr1541	pleopsidium.flavum.1	pleopsidium.flavum.1_725	450	1900	Newly-annotated lichen genomes	
xanthoparmelia.tasmanica.1_tyr2842	xanthoparmelia.tasmanica.1	xanthoparmelia.tasmanica.1_1472	5157	7305	Newly-annotated lichen genomes	
peltigera.hydrothyria.1_tyr3437	peltigera.hydrothyria.1	peltigera.hydrothyria.1_4255	21069	23446	Newly-annotated lichen genomes	
suisub3_tyr10267					Gluck-Thaler and Vogan (2024)	fam01
umbilicaria.muhlenbergii.1_tyr2675	umbilicaria.muhlenbergii.1	umbilicaria.muhlenbergii.1_1	5138583	5140836	Newly-annotated lichen genomes	
talsti1_4534					Gluck-Thaler and Vogan (2024)	fam01
aspasp13_tyr531					Gluck-Thaler and Vogan (2024)	fam01
asplat1_tyr1608					Gluck-Thaler and Vogan (2024)	fam01
aspawa1_tyr652					Gluck-Thaler and Vogan (2024)	fam01
aspcri1_2283					Gluck-Thaler and Vogan (2024)	fam01
aspluc3_tyr1878					Gluck-Thaler and Vogan (2024)	fam01
aspfis5_tyr1169					Gluck-Thaler and Vogan (2024)	fam01
asplen4_tyr1745					Gluck-Thaler and Vogan (2024)	fam01
asphir2_tyr1512					Gluck-Thaler and Vogan (2024)	fam01

artunc1_KAF3492431.1					Gluck-Thaler and Vogan (2024)	fam01
blaper1_3236					Gluck-Thaler and Vogan (2024)	fam01
blaper1_4705					Gluck-Thaler and Vogan (2024)	fam01
cocimm1_4716					Gluck-Thaler and Vogan (2024)	fam01
uncree2_EEP82143.1					Gluck-Thaler and Vogan (2024)	fam01
blagil1_OAT05240.1_OAT05241.1_OAT05242.1					Gluck-Thaler and Vogan (2024)	n.d.
parlut1_3075_3076					Gluck-Thaler and Vogan (2024)	n.d.
blaper1_4139_4140_4141					Gluck-Thaler and Vogan (2024)	n.d.
blaper1_5625					Gluck-Thaler and Vogan (2024)	fam01
psevol1_tyr9670					Gluck-Thaler and Vogan (2024)	fam01
cocimm2_KMU79836.1_KMU79837.1					Gluck-Thaler and Vogan (2024)	n.d.
blagil1_OAT07656.1					Gluck-Thaler and Vogan (2024)	fam01
pertusaria.ostiolata.1_tyr2466	pertusaria.ostiolata.1	pertusaria.ostiolata.1_1624	3461	5798	Newly-annotated lichen genomes	
lambiella.insularis.1_tyr2190	lambiella.insularis.1	lambiella.insularis.1_57	276883	278725	Newly-annotated lichen genomes	
lasallia.pustulata.1_tyr527	lasallia.pustulata.1	lasallia.pustulata.1_1	2115493	2117800	Newly-annotated lichen genomes	
umbpus1_102502					Gluck-Thaler and Vogan (2024)	fam01
lasallia.pustulata.7_tyr1579	lasallia.pustulata.7	lasallia.pustulata.7_893	36735	41976	Newly-annotated lichen genomes	
umbpus1_107339					Gluck-Thaler and Vogan (2024)	fam01
laspus1_KAA6408389.1_KAA6408390.1					Gluck-Thaler and Vogan (2024)	n.d.
xanthoparmelia.verrucella.1_tyr2858	xanthoparmelia.verrucella.1	xanthoparmelia.verrucella.1_1730	1296	2473	Newly-annotated lichen genomes	

wetmoreana.brouardii.1_tyr3494	wetmoreana.brouardii.1	wetmoreana.brouardii.1_768	7325	9422	Newly-annotated lichen genomes	
bacgig1_KAG8531634.1					Gluck-Thaler and Vogan (2024)	fam01
teloschistes.exilis.3_tyr3078	teloschistes.exilis.3	teloschistes.exilis.3_1065	4585	6139	Newly-annotated lichen genomes	
teloschistes.exilis.2_tyr3488	teloschistes.exilis.2	teloschistes.exilis.2_59	2699	4442	Newly-annotated lichen genomes	
flavoparmelia.baltimorensis.1_tyr3090	flavoparmelia.baltimorensis.1	flavoparmelia.baltimorensis.1_1658	4108	5742	Newly-annotated lichen genomes	
umbilicaria.polyphylla.3_tyr780	umbilicaria.polyphylla.3	umbilicaria.polyphylla.3_11	998932	1000288	Newly-annotated lichen genomes	
umbilicaria.polyphylla.4_tyr3789	umbilicaria.polyphylla.4	umbilicaria.polyphylla.4_12	1034965	1036321	Newly-annotated lichen genomes	
lepraria.sp..1_tyr3866	lepraria.sp..1	lepraria.sp..1_8345	3	1263	Newly-annotated lichen genomes	
umbilicaria.spodochroa.3_tyr1176	umbilicaria.spodochroa.3	umbilicaria.spodochroa.3_14	952265	953928	Newly-annotated lichen genomes	
umbilicaria.deusta.4_tyr3554	umbilicaria.deusta.4	umbilicaria.deusta.4_10	601661	603301	Newly-annotated lichen genomes	
flavoplaca.marina.2_tyr1816	flavoplaca.marina.2	flavoplaca.marina.2_2000	5451	7939	Newly-annotated lichen genomes	
lecanora.subimmergens.1_tyr2007	lecanora.subimmergens.1	lecanora.subimmergens.1_1741	3150	4496	Newly-annotated lichen genomes	
rusavskia.elegans.1_tyr3814	rusavskia.elegans.1	rusavskia.elegans.1_242	96436	98064	Newly-annotated lichen genomes	
arthonia.susa.1_tyr612	arthonia.susa.1	arthonia.susa.1_1209	866	3424	Newly-annotated lichen genomes	
gomphillus.calycioides.1_tyr2877	gomphillus.calycioides.1	gomphillus.calycioides.1_753	637	2209	Newly-annotated lichen genomes	
thelotrema.lepadinum.2_tyr2343	thelotrema.lepadinum.2	thelotrema.lepadinum.2_262	7906	9224	Newly-annotated lichen genomes	
leucodecton.sp..1_tyr1006	leucodecton.sp..1	leucodecton.sp..1_1376	893	2382	Newly-annotated lichen genomes	
baeomycetales.sp..4_tyr1767	baeomycetales.sp..4	baeomycetales.sp..4_280	555	2874	Newly-annotated lichen genomes	
leptogium.austroamericanum.2_tyr4156	leptogium.austroamericanum.2	leptogium.austroamericanum.2_1120	513	3141	Newly-annotated lichen genomes	
leptogium.corticola.1_tyr2099	leptogium.corticola.1	leptogium.corticola.1_2388	1790	3528	Newly-annotated lichen genomes	

acarospora.strigata.1_tyr1553	acarospora.strigata.1	acarospora.strigata.1_59	40226	42765	Newly-annotated lichen genomes	
arthonia.rubella.1_tyr891	arthonia.rubella.1	arthonia.rubella.1_1932	1042	2938	Newly-annotated lichen genomes	
parmin1_KAF9728395.1					Gluck-Thaler and Vogan (2024)	fam01
polfus1_527418					Gluck-Thaler and Vogan (2024)	fam01
zoprhi1_KAF2174473.1					Gluck-Thaler and Vogan (2024)	fam01
zoprhi1_KAF2179529.1					Gluck-Thaler and Vogan (2024)	fam01
pyrtri10_PZD03750.1					Gluck-Thaler and Vogan (2024)	fam01
phaeophyscia.squarrosa.1_tyr25	phaeophyscia.squarrosa.1	phaeophyscia.squarrosa.1_1163	6071	8148	Newly-annotated lichen genomes	
placynthium.petersii.1_tyr1421	placynthium.petersii.1	placynthium.petersii.1_2301	395	2473	Newly-annotated lichen genomes	
placynthium.petersii.1_tyr3666	placynthium.petersii.1	placynthium.petersii.1_363	4474	5997	Newly-annotated lichen genomes	
porpidia.macrocarpa.1_tyr3690	porpidia.macrocarpa.1	porpidia.macrocarpa.1_110	867	2854	Newly-annotated lichen genomes	
coniocarpon.cinnabarinum.2_tyr1581	coniocarpon.cinnabarinum.2	coniocarpon.cinnabarinum.2_535	2082	3912	Newly-annotated lichen genomes	
ochrolechia.trochophora.1_tyr194	ochrolechia.trochophora.1	ochrolechia.trochophora.1_1273	4903	7003	Newly-annotated lichen genomes	
pertusariales.sp..1_tyr553	pertusariales.sp..1	pertusariales.sp..1_221	30785	32818	Newly-annotated lichen genomes	
umbilicaria.grisea.2_tyr276	umbilicaria.grisea.2	umbilicaria.grisea.2_31	1042244	1044484	Newly-annotated lichen genomes	
umbilicaria.freyi.3_tyr2930	umbilicaria.freyi.3	umbilicaria.freyi.3_4	766539	767888	Newly-annotated lichen genomes	
umbilicaria.crustulosa.2_tyr1387	umbilicaria.crustulosa.2	umbilicaria.crustulosa.2_1	438893	440662	Newly-annotated lichen genomes	
umbilicaria.iberica.1_tyr3831	umbilicaria.iberica.1	umbilicaria.iberica.1_1	2461520	2463075	Newly-annotated lichen genomes	
umbilicaria.spodochroa.1_tyr827	umbilicaria.spodochroa.1	umbilicaria.spodochroa.1_118	507893	509873	Newly-annotated lichen genomes	
umbpus1_tyr10627					Gluck-Thaler and Vogan (2024)	clade02

heterodermia.speciosa.3_tyr1655	heterodermia.speciosa.3	heterodermia.speciosa.3_1694	42560	44033	Newly-annotated lichen genomes	
heterodermia.obscurata.1_tyr574	heterodermia.obscurata.1	heterodermia.obscurata.1_249	7548	9414	Newly-annotated lichen genomes	
hetspe1_CAF9941352.1					Gluck-Thaler and Vogan (2024)	clade01
mobergia.calculiformis.1_tyr1718	mobergia.calculiformis.1	mobergia.calculiformis.1_21	62217	64225	Newly-annotated lichen genomes	
phaeophyscia.hispidula.1_tyr3356	phaeophyscia.hispidula.1	phaeophyscia.hispidula.1_81	9272	11255	Newly-annotated lichen genomes	
trapelia.coarctata.1_tyr1301	trapelia.coarctata.1	trapelia.coarctata.1_203	3410	4636	Newly-annotated lichen genomes	
icmadophila.ericetorum.1_tyr2978	icmadophila.ericetorum.1	icmadophila.ericetorum.1_523	125128	126749	Newly-annotated lichen genomes	
umbilicaria.crustulosa.1_tyr2456	umbilicaria.crustulosa.1	umbilicaria.crustulosa.1_7	196604	198160	Newly-annotated lichen genomes	
umbilicaria.iberica.2_tyr1863	umbilicaria.iberica.2	umbilicaria.iberica.2_5	1384816	1386306	Newly-annotated lichen genomes	
umbpus1_103234					Gluck-Thaler and Vogan (2024)	fam03
umbilicaria.spodochroa.4_tyr3266	umbilicaria.spodochroa.4	umbilicaria.spodochroa.4_15	568970	570727	Newly-annotated lichen genomes	
lasallia.papulosa.1_tyr3891	lasallia.papulosa.1	lasallia.papulosa.1_1715	3307	4388	Newly-annotated lichen genomes	
pectenia.plumbea.1_tyr2272	pectenia.plumbea.1	pectenia.plumbea.1_569	8494	10167	Newly-annotated lichen genomes	
parmeliaceae.sp..15_tyr2654	parmeliaceae.sp..15	parmeliaceae.sp..15_4063	1866	2884	Newly-annotated lichen genomes	
pseudephebe.minuscula.1_tyr2975	pseudephebe.minuscula.1	pseudephebe.minuscula.1_115	7318	8063	Newly-annotated lichen genomes	
xanthoparmelia.taractica.1_tyr3364	xanthoparmelia.taractica.1	xanthoparmelia.taractica.1_331	13273	15112	Newly-annotated lichen genomes	
parmeliaceae.sp..1_tyr2917	parmeliaceae.sp..1	parmeliaceae.sp..1_1010	12825	13630	Newly-annotated lichen genomes	
hypotrachyna.osseoalba.1_tyr1699	hypotrachyna.osseoalba.1	hypotrachyna.osseoalba.1_269	9981	11749	Newly-annotated lichen genomes	
endocarpon.pusillum.1_tyr2645	endocarpon.pusillum.1	endocarpon.pusillum.1_424	17654	19474	Newly-annotated lichen genomes	
endpus1_4215					Gluck-Thaler and Vogan (2024)	fam03

endocarpon.pusillum.1_tyr746	endocarpon.pusillum.1	endocarpon.pusillum.1_764	74861	77250	Newly-annotated lichen genomes	
endpus1_7577					Gluck-Thaler and Vogan (2024)	fam03
rhizocarpon.subgeminatum.1_tyr3275	rhizocarpon.subgeminatum.1	rhizocarpon.subgeminatum.1_85	1939	3429	Newly-annotated lichen genomes	
umbilicaria.deusta.4_tyr2123	umbilicaria.deusta.4	umbilicaria.deusta.4_5	939200	940652	Newly-annotated lichen genomes	
endocarpon.pusillum.1_tyr3654	endocarpon.pusillum.1	endocarpon.pusillum.1_154	360145	361500	Newly-annotated lichen genomes	
porpidia.contraponenda.1_tyr415	porpidia.contraponenda.1	porpidia.contraponenda.1_1048	2745	4344	Newly-annotated lichen genomes	
phachl1_tyr9076					Gluck-Thaler and Vogan (2024)	fam03
capepi1_1814					Gluck-Thaler and Vogan (2024)	fam03
muellerella.sp..1_tyr1325	muellerella.sp..1	muellerella.sp..1_2	1512538	1514356	Newly-annotated lichen genomes	
melanina.sp..2_tyr3167	melanina.sp..2	melanina.sp..2_3	354186	355192	Newly-annotated lichen genomes	
aspcos3_tyr926					Gluck-Thaler and Vogan (2024)	fam03
aspscl4_RJE24476.1					Gluck-Thaler and Vogan (2024)	fam03
aspsp.1_RMJ24447.1					Gluck-Thaler and Vogan (2024)	fam03
aspcro1_tyr1048					Gluck-Thaler and Vogan (2024)	fam03
asptan1_tyr2858					Gluck-Thaler and Vogan (2024)	fam03
aspfis5_tyr1178					Gluck-Thaler and Vogan (2024)	fam03
asptam2_tyr2823					Gluck-Thaler and Vogan (2024)	fam03
asptra3_tyr2982					Gluck-Thaler and Vogan (2024)	fam03
aspell2_tyr1084					Gluck-Thaler and Vogan (2024)	fam03
penste1_6620_6621					Gluck-Thaler and Vogan (2024)	n.d.

penfla1_tyr8805					Gluck-Thaler and Vogan (2024)	fam03
aspell2_tyr1091					Gluck-Thaler and Vogan (2024)	fam03
penfla1_2705					Gluck-Thaler and Vogan (2024)	fam03
aspgla1_OJJ87743.1					Gluck-Thaler and Vogan (2024)	fam03
aspind3_tyr1556					Gluck-Thaler and Vogan (2024)	fam03
penchr2_30653					Gluck-Thaler and Vogan (2024)	fam03
pensub1_tyr9007					Gluck-Thaler and Vogan (2024)	fam03
eurrub1_525771					Gluck-Thaler and Vogan (2024)	fam03
blagil1_tyr3501					Gluck-Thaler and Vogan (2024)	fam03
hiscap4_QSS56076.1					Gluck-Thaler and Vogan (2024)	fam03
blaper1_3160_3159					Gluck-Thaler and Vogan (2024)	n.d.
cocimm2_KMU76246.1_KMU76247.1					Gluck-Thaler and Vogan (2024)	n.d.
emepas2_OJD09711.1					Gluck-Thaler and Vogan (2024)	fam03
hiscap1_5846					Gluck-Thaler and Vogan (2024)	fam03
trimen1_4445					Gluck-Thaler and Vogan (2024)	fam03
triton1_666					Gluck-Thaler and Vogan (2024)	fam03
emmcre2_KKZ67376.1					Gluck-Thaler and Vogan (2024)	fam03
arthonia.radiata.1_tyr3040	arthonia.radiata.1	arthonia.radiata.1_12	171334	173118	Newly-annotated lichen genomes	
opegrapha.moroziana.1_tyr4059	opegrapha.moroziana.1	opegrapha.moroziana.1_36	28764	30306	Newly-annotated lichen genomes	
coniocarpon.cinnabarinum.1_tyr1809	coniocarpon.cinnabarinum.1	coniocarpon.cinnabarinum.1_217	27761	29336	Newly-annotated lichen genomes	

coniocarpon.cinnabarinum.2_tyr2144	coniocarpon.cinnabarinum.2	coniocarpon.cinnabarinum.2_182	27752	28549	Newly-annotated lichen genomes	
coniocarpon.cinnabarinum.1_tyr1072	coniocarpon.cinnabarinum.1	coniocarpon.cinnabarinum.1_27	141132	143148	Newly-annotated lichen genomes	
solorina.crocea.1_tyr791	solorina.crocea.1	solorina.crocea.1_2510	4674	6576	Newly-annotated lichen genomes	
erebil1_192051					Gluck-Thaler and Vogan (2024)	fam03
erebil1_462223_462224_210623					Gluck-Thaler and Vogan (2024)	n.d.
delstr1_295949					Gluck-Thaler and Vogan (2024)	fam03
zymtri5_SMR62249.1					Gluck-Thaler and Vogan (2024)	fam03
umbilicaria.spodochroa.1_tyr602	umbilicaria.spodochroa.1	umbilicaria.spodochroa.1_113	491130	493103	Newly-annotated lichen genomes	
artoli1_7049					Gluck-Thaler and Vogan (2024)	fam03
orboli2_tyr8545					Gluck-Thaler and Vogan (2024)	fam03
daccio1_otherdc0103420					Gluck-Thaler and Vogan (2024)	fam03
orboli2_TGJ70084.1					Gluck-Thaler and Vogan (2024)	fam03
psora.crenata.1_tyr256	psora.crenata.1	psora.crenata.1_296	14785	15944	Newly-annotated lichen genomes	
blader3_6950_6951					Gluck-Thaler and Vogan (2024)	n.d.
blaper1_4528					Gluck-Thaler and Vogan (2024)	fam03
cocpos5_KMM73308.1					Gluck-Thaler and Vogan (2024)	fam03
parbra1_6508					Gluck-Thaler and Vogan (2024)	fam03
blaper1_5882					Gluck-Thaler and Vogan (2024)	fam03
blaper1_675					Gluck-Thaler and Vogan (2024)	fam03
blaper1_6736					Gluck-Thaler and Vogan (2024)	fam03

blaper1_6779					Gluck-Thaler and Vogan (2024)	fam03
cocimm1_tyr4120					Gluck-Thaler and Vogan (2024)	fam03
parbra1_581_582					Gluck-Thaler and Vogan (2024)	n.d.
parlut1_5370					Gluck-Thaler and Vogan (2024)	fam03
emeafr1_OAX85254.1					Gluck-Thaler and Vogan (2024)	fam03
parlut1_5348					Gluck-Thaler and Vogan (2024)	fam03
coniocarpon.cinnabarinum.1_tyr1808	coniocarpon.cinnabarinum.1	coniocarpon.cinnabarinum.1_153	24472	26564	Newly-annotated lichen genomes	
ascnig1_307582					Gluck-Thaler and Vogan (2024)	fam03
ascnig1_383553					Gluck-Thaler and Vogan (2024)	fam03
wilmik1_113864					Gluck-Thaler and Vogan (2024)	fam03
terbou1_tyr10393					Gluck-Thaler and Vogan (2024)	clade02
tercla1_1272127					Gluck-Thaler and Vogan (2024)	clade03
tercla1_tyr10405					Gluck-Thaler and Vogan (2024)	clade02
tercla1_tyr10408					Gluck-Thaler and Vogan (2024)	clade02
tirniv1_708397					Gluck-Thaler and Vogan (2024)	clade02
Starship-1_Mimp-Captain					Gluck-Thaler and Vogan (2024)	clade01
trihyb1_897299					Gluck-Thaler and Vogan (2024)	clade01
Starship-1_Ooli-Captain					Gluck-Thaler and Vogan (2024)	clade01
orboli11_KAF3077820.1					Gluck-Thaler and Vogan (2024)	clade01
choven1_786032					Gluck-Thaler and Vogan (2024)	clade01

tubmag1_tyr10581					Gluck-Thaler and Vogan (2024)	clade01
tubmag1_tyr10586					Gluck-Thaler and Vogan (2024)	clade01
tubmel2_tyr10605					Gluck-Thaler and Vogan (2024)	clade01
gomphillus.calycioides.1_tyr105	gomphillus.calycioides.1	gomphillus.calycioides.1_638	3816	5736	Newly-annotated lichen genomes	
fissurina.insidiosa.1_tyr1461	fissurina.insidiosa.1	fissurina.insidiosa.1_2171	855	2649	Newly-annotated lichen genomes	
acanthothecis.fontana.1_tyr1446	acanthothecis.fontana.1	acanthothecis.fontana.1_629	5766	7641	Newly-annotated lichen genomes	
acanthothecis.fontana.1_tyr2209	acanthothecis.fontana.1	acanthothecis.fontana.1_1919	735	1499	Newly-annotated lichen genomes	
nadvornikia.sorediata.1_tyr4112	nadvornikia.sorediata.1	nadvornikia.sorediata.1_2708	1168	2847	Newly-annotated lichen genomes	
grascr1_687					Gluck-Thaler and Vogan (2024)	clade01
leucodecton.sp..1_tyr2657	leucodecton.sp..1	leucodecton.sp..1_675	363	2082	Newly-annotated lichen genomes	
cyanodermella.asteris.1_tyr2814	cyanodermella.asteris.1	cyanodermella.asteris.1_16	766409	768208	Newly-annotated lichen genomes	
acarospora.sp..1_tyr498	acarospora.sp..1	acarospora.sp..1_55	516	1964	Newly-annotated lichen genomes	
icmadophila.ericetorum.1_tyr1628	icmadophila.ericetorum.1	icmadophila.ericetorum.1_114	2	757	Newly-annotated lichen genomes	
icmadophila.ericetorum.1_tyr3276	icmadophila.ericetorum.1	icmadophila.ericetorum.1_355	16752	17590	Newly-annotated lichen genomes	
dibbae1_5584_5585					Gluck-Thaler and Vogan (2024)	n.d.
pseudosagedia.sp..1_tyr1320	pseudosagedia.sp..1	pseudosagedia.sp..1_26	1498	3247	Newly-annotated lichen genomes	
lecanorales.sp..6_tyr1295	lecanorales.sp..6	lecanorales.sp..6_307	2799	4683	Newly-annotated lichen genomes	
teloschistes.exilis.3_tyr1984	teloschistes.exilis.3	teloschistes.exilis.3_1354	4273	5737	Newly-annotated lichen genomes	
buellia.spuria.2_tyr3704	buellia.spuria.2	buellia.spuria.2_30	39602	40605	Newly-annotated lichen genomes	
lecanorales.sp..3_tyr2207	lecanorales.sp..3	lecanorales.sp..3_1779	1358	2502	Newly-annotated lichen genomes	

variospora.aurantia.1_tyr2779	variospora.aurantia.1	variospora.aurantia.1_1561	3074	4582	Newly-annotated lichen genomes	
gallowayella.fulva.1_tyr2297	gallowayella.fulva.1	gallowayella.fulva.1_77	1977	3825	Newly-annotated lichen genomes	
calogaya.arnoldii.2_tyr1401	calogaya.arnoldii.2	calogaya.arnoldii.2_100	17430	18286	Newly-annotated lichen genomes	
xanthoria.sp..2_tyr633	xanthoria.sp..2	xanthoria.sp..2_79	18944	20361		
teloschistes.exilis.3_tyr394	teloschistes.exilis.3	teloschistes.exilis.3_80	30756	32505	Newly-annotated lichen genomes	
teloschistes.exilis.1_tyr3101	teloschistes.exilis.1	teloschistes.exilis.1_820	3727	4680	Newly-annotated lichen genomes	
teloschistes.peruensis.1_tyr3340	teloschistes.peruensis.1	teloschistes.peruensis.1_724	28	1761	Newly-annotated lichen genomes	
cyanodermella.asteris.1_tyr1869	cyanodermella.asteris.1	cyanodermella.asteris.1_28	813973	815811	Newly-annotated lichen genomes	
agyrium.rufum.1_tyr287	agyrium.rufum.1	agyrium.rufum.1_220	21229	22125	Newly-annotated lichen genomes	
pertusaria.plittiana.1_tyr1206	pertusaria.plittiana.1	pertusaria.plittiana.1_61	4247	6054	Newly-annotated lichen genomes	
pertusaria.obruta.1_tyr2146	pertusaria.obruta.1	pertusaria.obruta.1_2213	1127	1990	Newly-annotated lichen genomes	
stictis.urceolata.2_tyr224	stictis.urceolata.2	stictis.urceolata.2_492	8008	9769	Newly-annotated lichen genomes	
stictis.urceolata.1_tyr1442	stictis.urceolata.1	stictis.urceolata.1_296	8137	9947	Newly-annotated lichen genomes	
stictis.urceolata.2_tyr3715	stictis.urceolata.2	stictis.urceolata.2_971	1770	2744	Newly-annotated lichen genomes	
porina.heterospora.1_tyr2130	porina.heterospora.1	porina.heterospora.1_1129	4784	6570	Newly-annotated lichen genomes	
umbilicaria.iberica.1_tyr757	umbilicaria.iberica.1	umbilicaria.iberica.1_10	687098	689186	Newly-annotated lichen genomes	
umbpus1_103454					Gluck-Thaler and Vogan (2024)	clade01
morimp1_601763					Gluck-Thaler and Vogan (2024)	clade02
morimp1_644698					Gluck-Thaler and Vogan (2024)	clade02
flavoplaca.citrina.1_tyr34	flavoplaca.citrina.1	flavoplaca.citrina.1_166	32110	33267	Newly-annotated lichen genomes	

flavoplaca.marina.2_tyr1457	flavoplaca.marina.2	flavoplaca.marina.2_12090	27	944	Newly-annotated lichen genomes	
flavoplaca.austrocitrina.1_tyr4097	flavoplaca.austrocitrina.1	flavoplaca.austrocitrina.1_298	706	1887	Newly-annotated lichen genomes	
flavoplaca.sp..1_tyr2361	flavoplaca.sp..1	flavoplaca.sp..1_208	1954	3165	Newly-annotated lichen genomes	
kuettlingeria.erythrocarpa.1_tyr700	kuettlingeria.erythrocarpa.1	kuettlingeria.erythrocarpa.1_52	9995	10854	Newly-annotated lichen genomes	
lecanorales.sp..2_tyr388	lecanorales.sp..2	lecanorales.sp..2_471	6390	7493	Newly-annotated lichen genomes	
cerbet1_PIA90313.1					Gluck-Thaler and Vogan (2024)	clade03
lecidella.sp..1_tyr1820	lecidella.sp..1	lecidella.sp..1_5	2374	3676	Newly-annotated lichen genomes	
melanohalea.halei.1_tyr2427	melanohalea.halei.1	melanohalea.halei.1_1938	3744	4766	Newly-annotated lichen genomes	
phaeophyscia.hispidula.1_tyr2960	phaeophyscia.hispidula.1	phaeophyscia.hispidula.1_704	13596	14570	Newly-annotated lichen genomes	
pseudosagedia.isidiata.1_tyr477	pseudosagedia.isidiata.1	pseudosagedia.isidiata.1_230	12834	13820	Newly-annotated lichen genomes	
fissurina.insidiosa.1_tyr3590	fissurina.insidiosa.1	fissurina.insidiosa.1_1311	1845	2978	Newly-annotated lichen genomes	
imshaugia.aleurites.2_tyr2567	imshaugia.aleurites.2	imshaugia.aleurites.2_18	61439	62677	Newly-annotated lichen genomes	
xanthoparmelia.verrucella.1_tyr1823	xanthoparmelia.verrucella.1	xanthoparmelia.verrucella.1_138	10410	11057	Newly-annotated lichen genomes	
pyrrhospora.varians.1_tyr3775	pyrrhospora.varians.1	pyrrhospora.varians.1_80	12252	13499	Newly-annotated lichen genomes	
lobaria.pulmonaria.1_tyr474	lobaria.pulmonaria.1	lobaria.pulmonaria.1_610	4997	5899	Newly-annotated lichen genomes	
lobaria.immixta.1_tyr2349	lobaria.immixta.1	lobaria.immixta.1_1	93104	94201	Newly-annotated lichen genomes	
arthoniomycete.sp..1_tyr581	arthoniomycete.sp..1	arthoniomycete.sp..1_8	455121	456368	Newly-annotated lichen genomes	
umbilicaria.nylanderiana.2_tyr3926	umbilicaria.nylanderiana.2	umbilicaria.nylanderiana.2_12	263381	264442	Newly-annotated lichen genomes	
dendriscocaulon.intricatulum.1_tyr4096	dendriscocaulon.intricatulum.1	dendriscocaulon.intricatulum.1_26	695	1827	Newly-annotated lichen genomes	
ramalina.siliquosa.1_tyr3421	ramalina.siliquosa.1	ramalina.siliquosa.1_31	281820	282986	Newly-annotated lichen genomes	

elsfaw1_KAF4556931.1					Gluck-Thaler and Vogan (2024)	clade03
rutsp.1_tyr10026					Gluck-Thaler and Vogan (2024)	clade03
pertusaria.andersoniae.1_tyr225	pertusaria.andersoniae.1	pertusaria.andersoniae.1_286	5437	6216	Newly-annotated lichen genomes	
varicellaria.rhodocarpa.1_tyr3174	varicellaria.rhodocarpa.1	varicellaria.rhodocarpa.1_1890	31213	32444	Newly-annotated lichen genomes	
aspicilia.laevata.1_tyr1692	aspicilia.laevata.1	aspicilia.laevata.1_5	20857	22187	Newly-annotated lichen genomes	
pertusaria.ostiolata.1_tyr3427	pertusaria.ostiolata.1	pertusaria.ostiolata.1_1221	1777	2910	Newly-annotated lichen genomes	
zymtri1_EGP85669.1					Gluck-Thaler and Vogan (2024)	clade03
exophiala.sp..1_tyr2542	exophiala.sp..1	exophiala.sp..1_4	83476	84591	Newly-annotated lichen genomes	
exophiala.sp..1_tyr3133	exophiala.sp..1	exophiala.sp..1_1	856337	857392	Newly-annotated lichen genomes	
exospi1_6301					Gluck-Thaler and Vogan (2024)	clade03
aspasp1_tyr547					Gluck-Thaler and Vogan (2024)	clade03
aspcal3_tyr819					Gluck-Thaler and Vogan (2024)	clade03
lecanora.hybocarpa.1_tyr1670	lecanora.hybocarpa.1	lecanora.hybocarpa.1_859	928	2165	Newly-annotated lichen genomes	
trapeliopsis.flexuosa.1_tyr3499	trapeliopsis.flexuosa.1	trapeliopsis.flexuosa.1_160	1735	2970	Newly-annotated lichen genomes	
cladonia.coccifera.1_tyr2439	cladonia.coccifera.1	cladonia.coccifera.1_19	46022	47303	Newly-annotated lichen genomes	
collema.furfuraceum.1_tyr571	collema.furfuraceum.1	collema.furfuraceum.1_1304	2555	3810	Newly-annotated lichen genomes	
psesp.10_OBT66236.1					Gluck-Thaler and Vogan (2024)	clade03
psesp.14_KFY24325.1					Gluck-Thaler and Vogan (2024)	clade03
usnochroma.carphineum.1_tyr2888	usnochroma.carphineum.1	usnochroma.carphineum.1_362	4023	5034	Newly-annotated lichen genomes	
ramalina.farinacea.1_tyr3393	ramalina.farinacea.1	ramalina.farinacea.1_43	1492009	1492921	Newly-annotated lichen genomes	

bacidia.gigantensis.1_tyr4167	bacidia.gigantensis.1	bacidia.gigantensis.1_2	734681	735956	Newly-annotated lichen genomes	
bacgig1_KAG8533760.1					Gluck-Thaler and Vogan (2024)	clade03
botryolepraria.lesdainii.1_tyr3146	botryolepraria.lesdainii.1	botryolepraria.lesdainii.1_2727	1418	2515	Newly-annotated lichen genomes	
phisub1_19656					Gluck-Thaler and Vogan (2024)	clade03
lecanorales.sp..5_tyr3080	lecanorales.sp..5	lecanorales.sp..5_902	3376	4315	Newly-annotated lichen genomes	
ptychographa.xylographoides.2_tyr1503	ptychographa.xylographoides.2	ptychographa.xylographoides.2_122	10371	11587	Newly-annotated lichen genomes	
psesp.16_KFY82747.1					Gluck-Thaler and Vogan (2024)	clade03
parmotrema.reticulatum.1_tyr3664	parmotrema.reticulatum.1	parmotrema.reticulatum.1_368	13550	14698	Newly-annotated lichen genomes	
flavoplaca.marina.2_tyr2556	flavoplaca.marina.2	flavoplaca.marina.2_267	27156	28372	Newly-annotated lichen genomes	
lecanora.atra.1_tyr2332	lecanora.atra.1	lecanora.atra.1_2309	1821	3118	Newly-annotated lichen genomes	
lecanorales.sp..5_tyr2453	lecanorales.sp..5	lecanorales.sp..5_481	3234	4033	Newly-annotated lichen genomes	
leucodermia.leucomelos.1_tyr2768	leucodermia.leucomelos.1	leucodermia.leucomelos.1_862	1185	2186	Newly-annotated lichen genomes	
heterodermia.langdoniana.1_tyr1258	heterodermia.langdoniana.1	heterodermia.langdoniana.1_441	11622	14065	Newly-annotated lichen genomes	
heterodermia.obscurata.1_tyr2835	heterodermia.obscurata.1	heterodermia.obscurata.1_209	45944	48049	Newly-annotated lichen genomes	
polyblastidium.squamulosum.1_tyr4040	polyblastidium.squamulosum.1	polyblastidium.squamulosum.1_132	23862	25073	Newly-annotated lichen genomes	
lecanorales.sp..2_tyr530	lecanorales.sp..2	lecanorales.sp..2_769	2533	3762	Newly-annotated lichen genomes	
buellia.spuria.2_tyr3283	buellia.spuria.2	buellia.spuria.2_1713	3918	5246	Newly-annotated lichen genomes	
buellia.stillingiana.1_tyr3619	buellia.stillingiana.1	buellia.stillingiana.1_278	18267	19431	Newly-annotated lichen genomes	
melanohalea.halei.1_tyr1352	melanohalea.halei.1	melanohalea.halei.1_85	29641	31204	Newly-annotated lichen genomes	
xanthoparmelia.verrucella.1_tyr2252	xanthoparmelia.verrucella.1	xanthoparmelia.verrucella.1_437	19077	20137	Newly-annotated lichen genomes	

usnea.florida.1_tyr3426	usnea.florida.1	usnea.florida.1_45	90767	92058	Newly-annotated lichen genomes	
pucgra2_10909					Gluck-Thaler and Vogan (2024)	clade03
lecanora.hybocarpa.1_tyr316	lecanora.hybocarpa.1	lecanora.hybocarpa.1_580	10004	11268	Newly-annotated lichen genomes	
buellia.spuria.1_tyr1883	buellia.spuria.1	buellia.spuria.1_14	11953	13151	Newly-annotated lichen genomes	
heterodermia.langdoniana.1_tyr1257	heterodermia.langdoniana.1	heterodermia.langdoniana.1_1910	4215	5301	Newly-annotated lichen genomes	
heterodermia.sp..1_tyr3905	heterodermia.sp..1	heterodermia.sp..1_1058	6877	7635	Newly-annotated lichen genomes	
heterodermia.obscurata.1_tyr1259	heterodermia.obscurata.1	heterodermia.obscurata.1_7	88773	89986	Newly-annotated lichen genomes	
loekoesia.sp..1_tyr2499	loekoesia.sp..1	loekoesia.sp..1_693	2872	5053	Newly-annotated lichen genomes	
ionaspis.lacustris.1_tyr2819	ionaspis.lacustris.1	ionaspis.lacustris.1_1050	2270	3533	Newly-annotated lichen genomes	
fusfra1_tyr5556					Gluck-Thaler and Vogan (2024)	clade03
pertusaria.plittiana.1_tyr3414	pertusaria.plittiana.1	pertusaria.plittiana.1_150	21844	22892	Newly-annotated lichen genomes	
fusfus16_tyr5625					Gluck-Thaler and Vogan (2024)	clade03
fusfus29_tyr5704					Gluck-Thaler and Vogan (2024)	clade03
fusfus30_tyr5713					Gluck-Thaler and Vogan (2024)	clade03
ilydes1_KAH6980527.1					Gluck-Thaler and Vogan (2024)	clade03
fusvan1_EEU46504.1					Gluck-Thaler and Vogan (2024)	clade03
fusfus39_tyr5764					Gluck-Thaler and Vogan (2024)	clade03
micbol1_176150					Gluck-Thaler and Vogan (2024)	clade03
phicur1_TPX13777.1					Gluck-Thaler and Vogan (2024)	clade03
elsfaw1_KAF4547791.1					Gluck-Thaler and Vogan (2024)	clade03

lecanorales.sp..2_tyr529	lecanorales.sp..2	lecanorales.sp..2_673	6406	7370	Newly-annotated lichen genomes	
lecanorales.sp..2_tyr2350	lecanorales.sp..2	lecanorales.sp..2_211	1449	2572	Newly-annotated lichen genomes	
zwackhia.viridis.1_tyr2925	zwackhia.viridis.1	zwackhia.viridis.1_162	18091	18891	Newly-annotated lichen genomes	
pyrter2_CAA9956742.1					Gluck-Thaler and Vogan (2024)	clade03
pyrter3_EFQ88401.1					Gluck-Thaler and Vogan (2024)	clade03
mellar1_EGG09125.1					Gluck-Thaler and Vogan (2024)	clade03
caloplaca.sp..1_tyr2181	caloplaca.sp..1	caloplaca.sp..1_5	225313	227552	Newly-annotated lichen genomes	
dendriscocaulon.intricatulum.1_tyr934	dendriscocaulon.intricatulum.1	dendriscocaulon.intricatulum.1_1241	98	871	Newly-annotated lichen genomes	
moelib1_tyr8263					Gluck-Thaler and Vogan (2024)	fam09
tolinf1_199956					Gluck-Thaler and Vogan (2024)	clade01
pseudocyphellaria.aurata.1_tyr2535	pseudocyphellaria.aurata.1	pseudocyphellaria.aurata.1_40	81187	82048	Newly-annotated lichen genomes	
porpidia.subsimplex.1_tyr3915	porpidia.subsimplex.1	porpidia.subsimplex.1_609	3429	5640	Newly-annotated lichen genomes	
sticta.canariensis.1_tyr1454	sticta.canariensis.1	sticta.canariensis.1_90	3162	5195	Newly-annotated lichen genomes	
treper1_499963_134336					Gluck-Thaler and Vogan (2024)	n.d.
phlbre1_15608					Gluck-Thaler and Vogan (2024)	fam09
volvol2_KAF8645791.1					Gluck-Thaler and Vogan (2024)	fam09
acanthothecis.fontana.1_tyr3737	acanthothecis.fontana.1	acanthothecis.fontana.1_887	1470	2306	Newly-annotated lichen genomes	
punctelia.appalachensis.1_tyr3058	punctelia.appalachensis.1	punctelia.appalachensis.1_1227	1590	3664	Newly-annotated lichen genomes	
kredeu2_498028					Gluck-Thaler and Vogan (2024)	fam09
porpidia.subsimplex.1_tyr3802	porpidia.subsimplex.1	porpidia.subsimplex.1_1297	4877	7111	Newly-annotated lichen genomes	

tulsp.13_KAG8978541.1_KAG8978542.1					Gluck-Thaler and Vogan (2024)	n.d.
annsty1_339604_401957					Gluck-Thaler and Vogan (2024)	n.d.
kredeu2_220161_512558					Gluck-Thaler and Vogan (2024)	n.d.
nemser2_436945					Gluck-Thaler and Vogan (2024)	fam09
annsty1_404214					Gluck-Thaler and Vogan (2024)	fam09
hyptru1_435293					Gluck-Thaler and Vogan (2024)	fam09
annsty1_427123					Gluck-Thaler and Vogan (2024)	fam09
monsp.3_tyr8334					Gluck-Thaler and Vogan (2024)	fam09
monsp.6_tyr8358					Gluck-Thaler and Vogan (2024)	fam09
astsub1_437548					Gluck-Thaler and Vogan (2024)	fam09
astsub1_465646					Gluck-Thaler and Vogan (2024)	fam09
xylsp.5_451751					Gluck-Thaler and Vogan (2024)	fam09
xylsp.4_304735					Gluck-Thaler and Vogan (2024)	fam09
truang1_533773					Gluck-Thaler and Vogan (2024)	fam09
tolpar1_tyr10469					Gluck-Thaler and Vogan (2024)	fam09
chaglo1_159177					Gluck-Thaler and Vogan (2024)	fam09
chasp.2_300927					Gluck-Thaler and Vogan (2024)	fam09
stalon1_KAG7293987.1					Gluck-Thaler and Vogan (2024)	fam09
flavoplaca.austrocitrina.1_tyr1793	flavoplaca.austrocitrina.1	flavoplaca.austrocitrina.1_9429	217	1263	Newly-annotated lichen genomes	
cladophialophora.sp..2_tyr2183	cladophialophora.sp..2	cladophialophora.sp..2_9	4668127	4671427	Newly-annotated lichen genomes	

lopnuc1_601207					Gluck-Thaler and Vogan (2024)	fam09
zoprhi1_KAF2186083.1					Gluck-Thaler and Vogan (2024)	fam09
lacwil1_tyr7764					Gluck-Thaler and Vogan (2024)	fam09
xylbam1_657765					Gluck-Thaler and Vogan (2024)	fam09
erebil1_455447					Gluck-Thaler and Vogan (2024)	fam09
helsp.4_KAE8441012.1					Gluck-Thaler and Vogan (2024)	fam09
lepsp.1_603043_720900_734108					Gluck-Thaler and Vogan (2024)	n.d.
kuettlingeria.erythrocarpa.1_tyr2630	kuettlingeria.erythrocarpa.1	kuettlingeria.erythrocarpa.1_182	3750	4667	Newly-annotated lichen genomes	
pannaria.tavaresii.1_tyr3625	pannaria.tavaresii.1	pannaria.tavaresii.1_1403	2615	3842	Newly-annotated lichen genomes	
fissurina.insidiosa.1_tyr3001	fissurina.insidiosa.1	fissurina.insidiosa.1_288	13399	15115	Newly-annotated lichen genomes	
sticta.canariensis.1_tyr3468	sticta.canariensis.1	sticta.canariensis.1_2913	13925	16099	Newly-annotated lichen genomes	
lining1_125901_125887					Gluck-Thaler and Vogan (2024)	n.d.
polfus1_16250					Gluck-Thaler and Vogan (2024)	fam09
Starship-1_Foxy_Fol4287-Captain					Gluck-Thaler and Vogan (2024)	fam09
fussp.4_KAF9770092.1					Gluck-Thaler and Vogan (2024)	fam09
fusxyl9_tyr7051					Gluck-Thaler and Vogan (2024)	fam09
fusfus45_tyr5795					Gluck-Thaler and Vogan (2024)	fam09
fusdom1_tyr5472					Gluck-Thaler and Vogan (2024)	fam09
fusfus31_tyr5727					Gluck-Thaler and Vogan (2024)	fam09
fusfus39_tyr5763					Gluck-Thaler and Vogan (2024)	fam09

fusill1_tyr5996				Gluck-Thaler and Vogan (2024)	fam09
neodit1_1586				Gluck-Thaler and Vogan (2024)	fam09
fuspen1_tyr6438				Gluck-Thaler and Vogan (2024)	fam09
cyloli1_548470				Gluck-Thaler and Vogan (2024)	fam09
dacest1_612184				Gluck-Thaler and Vogan (2024)	fam09
fusbeo1_KAF4342966.1				Gluck-Thaler and Vogan (2024)	fam09
fusxyr1_tyr7062				Gluck-Thaler and Vogan (2024)	fam09
fuscon1_tyr5412				Gluck-Thaler and Vogan (2024)	fam09
fussp.6_tyr6673				Gluck-Thaler and Vogan (2024)	fam09
neodit1_181				Gluck-Thaler and Vogan (2024)	fam09
fusman2_tyr6057				Gluck-Thaler and Vogan (2024)	fam09
fusnem1_tyr6103				Gluck-Thaler and Vogan (2024)	fam09
fusnem1_tyr6106				Gluck-Thaler and Vogan (2024)	fam09
geomor1_KAF4119892.1				Gluck-Thaler and Vogan (2024)	fam09
pocchl2_RZR69165.1				Gluck-Thaler and Vogan (2024)	fam09
hirmin1_9210				Gluck-Thaler and Vogan (2024)	fam09
Starship-1_Tasp-Captain				Gluck-Thaler and Vogan (2024)	fam09
triasp2_43980				Gluck-Thaler and Vogan (2024)	fam09
triatr4_tyr10505				Gluck-Thaler and Vogan (2024)	fam09
beabas2_KGQ02280.1				Gluck-Thaler and Vogan (2024)	fam09

isafum1_tyr7678				Gluck-Thaler and Vogan (2024)	fam09
leclec1_10286				Gluck-Thaler and Vogan (2024)	fam09
pocchl2_RZR70293.1				Gluck-Thaler and Vogan (2024)	fam09
colfru1_KAE9565890.1				Gluck-Thaler and Vogan (2024)	fam09
colsub1_6891				Gluck-Thaler and Vogan (2024)	fam09
colsal1_941573				Gluck-Thaler and Vogan (2024)	fam09
coltri1_tyr4417				Gluck-Thaler and Vogan (2024)	fam09
colvin1_tyr4425				Gluck-Thaler and Vogan (2024)	fam09
coltof2_1585				Gluck-Thaler and Vogan (2024)	fam09
diabat1_KAG8162722.1				Gluck-Thaler and Vogan (2024)	fam09
hypsp.2_326193_642931				Gluck-Thaler and Vogan (2024)	n.d.
xylsp.19_30440				Gluck-Thaler and Vogan (2024)	fam09
mictri1_388981				Gluck-Thaler and Vogan (2024)	fam09
verdah3_tyr10695				Gluck-Thaler and Vogan (2024)	fam09
verlon1_1636				Gluck-Thaler and Vogan (2024)	fam09
Starship-2_Pans-Captain				Gluck-Thaler and Vogan (2024)	fam09
podans1_209680				Gluck-Thaler and Vogan (2024)	fam09
podcom1_tyr9195				Gluck-Thaler and Vogan (2024)	fam09
chasp.2_354242				Gluck-Thaler and Vogan (2024)	fam09
micbol1_140131				Gluck-Thaler and Vogan (2024)	fam09

mictri1_436685				Gluck-Thaler and Vogan (2024)	fam09
xylarb1_416827				Gluck-Thaler and Vogan (2024)	fam09
xylarb1_485319				Gluck-Thaler and Vogan (2024)	fam09
truang1_417612				Gluck-Thaler and Vogan (2024)	fam09
crypar1_KAF3763129.1				Gluck-Thaler and Vogan (2024)	fam09
diacit1_KAG6368733.1				Gluck-Thaler and Vogan (2024)	fam09
valmal3_664				Gluck-Thaler and Vogan (2024)	fam09
diahel1_9225				Gluck-Thaler and Vogan (2024)	fam09
podcom1_tyr9196				Gluck-Thaler and Vogan (2024)	fam09
neodit1_6628				Gluck-Thaler and Vogan (2024)	fam09
pyrgri1_TLD03770.1				Gluck-Thaler and Vogan (2024)	fam09
pyrory4_ELQ69711.1				Gluck-Thaler and Vogan (2024)	fam09
pyrsp.2_TLD25447.1				Gluck-Thaler and Vogan (2024)	fam09
pyrgri1_TLD05862.1				Gluck-Thaler and Vogan (2024)	fam09
pyrory2_QBZ54310.1				Gluck-Thaler and Vogan (2024)	fam09
sorsp.1_KAH7633559.1				Gluck-Thaler and Vogan (2024)	fam09
sorsp.1_KAH7633844.1				Gluck-Thaler and Vogan (2024)	fam09
colasi1_KAF0330480.1				Gluck-Thaler and Vogan (2024)	fam09
colsid1_tyr4377				Gluck-Thaler and Vogan (2024)	fam09
colsid1_tyr4379				Gluck-Thaler and Vogan (2024)	fam09

colplu1_KAF6806911.1					Gluck-Thaler and Vogan (2024)	fam09
colplu1_KAF6808160.1					Gluck-Thaler and Vogan (2024)	fam09
colplu1_KAF6821138.1					Gluck-Thaler and Vogan (2024)	fam09
erebil1_510855					Gluck-Thaler and Vogan (2024)	fam09
crypar1_tyr4503					Gluck-Thaler and Vogan (2024)	fam09
diacit1_KAG6354864.1					Gluck-Thaler and Vogan (2024)	fam09
choven1_1052954					Gluck-Thaler and Vogan (2024)	fam08
lecanoromycetidae.sp..1_tyr127	lecanoromycetidae.sp..1	lecanoromycetidae.sp..1_36	39702	41948	Newly-annotated lichen genomes	
dibaeis.baeomyces.1_tyr557	dibaeis.baeomyces.1	dibaeis.baeomyces.1_72	95330	96572	Newly-annotated lichen genomes	
umbilicaria.phaea.2_tyr434	umbilicaria.phaea.2	umbilicaria.phaea.2_12	274222	275333	Newly-annotated lichen genomes	
ascsar1_3734					Gluck-Thaler and Vogan (2024)	clade03
icmadophila.ericetorum.1_tyr3161	icmadophila.ericetorum.1	icmadophila.ericetorum.1_712	83545	85894	Newly-annotated lichen genomes	
icmadophila.ericetorum.2_tyr1798	icmadophila.ericetorum.2	icmadophila.ericetorum.2_4821	306	1373	Newly-annotated lichen genomes	
emepas2_OJD15817.1_OJD15818.1					Gluck-Thaler and Vogan (2024)	n.d.
teratosphaeriaceae.sp..1_tyr1571	teratosphaeriaceae.sp..1	teratosphaeriaceae.sp..1_24	934436	935296	Newly-annotated lichen genomes	
baupan1_EMC98256.1					Gluck-Thaler and Vogan (2024)	clade03
altgai1_tyr157					Gluck-Thaler and Vogan (2024)	clade03
altsol2_tyr198					Gluck-Thaler and Vogan (2024)	clade03
stasp.1_382781					Gluck-Thaler and Vogan (2024)	clade03
parnod1_QRC96480.1					Gluck-Thaler and Vogan (2024)	clade03

arthoniomycete.sp..1_tyr168	arthoniomycete.sp..1	arthoniomycete.sp..1_5	113697	114569	Newly-annotated lichen genomes	
aurpul6_KEQ79301.1					Gluck-Thaler and Vogan (2024)	clade03
hyspul1_112804					Gluck-Thaler and Vogan (2024)	clade03
zymtri3_SMQ45618.1					Gluck-Thaler and Vogan (2024)	clade03
zymtri4_SMY26934.1					Gluck-Thaler and Vogan (2024)	clade03
cyanodermella.asteris.1_tyr3589	cyanodermella.asteris.1	cyanodermella.asteris.1_29	1025004	1025804	Newly-annotated lichen genomes	
aurpul2_36615					Gluck-Thaler and Vogan (2024)	clade03
leprocaulon.nicholsiae.1_tyr3158	leprocaulon.nicholsiae.1	leprocaulon.nicholsiae.1_435	11191	12073	Newly-annotated lichen genomes	
mictri1_387286					Gluck-Thaler and Vogan (2024)	clade03
consp.1_1239454					Gluck-Thaler and Vogan (2024)	clade03
fusbux1_tyr5352					Gluck-Thaler and Vogan (2024)	clade03
fustha1_tyr6742					Gluck-Thaler and Vogan (2024)	clade03
ptychographa.xylographoides.1_tyr1363	ptychographa.xylographoides.1	ptychographa.xylographoides.1_110	2048	3243	Newly-annotated lichen genomes	
colcam1_KAH0438924.1					Gluck-Thaler and Vogan (2024)	clade03
colglo1_5357					Gluck-Thaler and Vogan (2024)	clade03
xylsp.8_760567					Gluck-Thaler and Vogan (2024)	clade03
colsia5_tyr4375					Gluck-Thaler and Vogan (2024)	clade03
fuspen1_tyr6433					Gluck-Thaler and Vogan (2024)	clade03
pertusaria.plittiana.1_tyr1285	pertusaria.plittiana.1	pertusaria.plittiana.1_2521	3226	4263	Newly-annotated lichen genomes	
pertusaria.propinqua.1_tyr4079	pertusaria.propinqua.1	pertusaria.propinqua.1_1494	5756	6585	Newly-annotated lichen genomes	

psesp.4_4572					Gluck-Thaler and Vogan (2024)	clade03
flavoplaca.limonia.1_tyr495	flavoplaca.limonia.1	flavoplaca.limonia.1_270	14324	15262	Newly-annotated lichen genomes	
flavoplaca.limonia.1_tyr3663	flavoplaca.limonia.1	flavoplaca.limonia.1_287	13838	14650	Newly-annotated lichen genomes	
lecanora.masana.1_tyr3081	lecanora.masana.1	lecanora.masana.1_16	47077	47763	Newly-annotated lichen genomes	
pertusaria.obruta.1_tyr1248	pertusaria.obruta.1	pertusaria.obruta.1_876	4929	5567	Newly-annotated lichen genomes	
cladonia.diversa.2_tyr1587	cladonia.diversa.2	cladonia.diversa.2_212	21139	22050	Newly-annotated lichen genomes	
umbilicaria.phaea.3_tyr2049	umbilicaria.phaea.3	umbilicaria.phaea.3_3	973993	974937	Newly-annotated lichen genomes	
exomes1_110667					Gluck-Thaler and Vogan (2024)	clade03
aurpul1_15872					Gluck-Thaler and Vogan (2024)	clade03
aursub2_KEQ98213.1					Gluck-Thaler and Vogan (2024)	clade03
psefij1_EME77334.1					Gluck-Thaler and Vogan (2024)	clade03
botdew1_KAF7921612.1					Gluck-Thaler and Vogan (2024)	clade03
racsp.1_tyr9894					Gluck-Thaler and Vogan (2024)	clade03
jimfla1_476449					Gluck-Thaler and Vogan (2024)	clade03
jimfla2_143193					Gluck-Thaler and Vogan (2024)	clade03
pertusaria.propinqua.1_tyr292	pertusaria.propinqua.1	pertusaria.propinqua.1_22	9254	11410	Newly-annotated lichen genomes	
pertusaria.ostiolata.1_tyr2780	pertusaria.ostiolata.1	pertusaria.ostiolata.1_524	9869	11039	Newly-annotated lichen genomes	
pertusaria.epixantha.1_tyr2831	pertusaria.epixantha.1	pertusaria.epixantha.1_503	6873	8310	Newly-annotated lichen genomes	
dibaeis.sorediata.1_tyr503	dibaeis.sorediata.1	dibaeis.sorediata.1_30	29116	29852	Newly-annotated lichen genomes	
leucodermia.leucomelos.1_tyr1351	leucodermia.leucomelos.1	leucodermia.leucomelos.1_2039	2137	4369	Newly-annotated lichen genomes	

heterodermia.sp..3_tyr1517	heterodermia.sp..3	heterodermia.sp..3_1361	36955	38175	Newly-annotated lichen genomes	
physciaceae.sp..4_tyr3794	physciaceae.sp..4	physciaceae.sp..4_1249	34518	36501	Newly-annotated lichen genomes	
vulpicida.juniperinus.1_tyr1524	vulpicida.juniperinus.1	vulpicida.juniperinus.1_24	121180	123803	Newly-annotated lichen genomes	
ascsar1_7037					Gluck-Thaler and Vogan (2024)	fam11
chlaer1_TAQ83553.1					Gluck-Thaler and Vogan (2024)	fam11
cadsp.2_KAG4436364.1					Gluck-Thaler and Vogan (2024)	fam11
leosp.1_775379					Gluck-Thaler and Vogan (2024)	fam11
diacit1_KAG6354360.1					Gluck-Thaler and Vogan (2024)	fam11
elsamp1_316789					Gluck-Thaler and Vogan (2024)	fam11
spobra1_10745					Gluck-Thaler and Vogan (2024)	fam11
glaloz2_EHL01589.1					Gluck-Thaler and Vogan (2024)	fam11
lachya1_TVY26861.1					Gluck-Thaler and Vogan (2024)	fam11
umbilicaria.americana.1_tyr1621	umbilicaria.americana.1	umbilicaria.americana.1_21	5667	8425	Newly-annotated lichen genomes	
psesp.14_KFY24906.1					Gluck-Thaler and Vogan (2024)	fam11
leosp.1_718556					Gluck-Thaler and Vogan (2024)	fam11
umbilicaria.freyi.3_tyr456	umbilicaria.freyi.3	umbilicaria.freyi.3_13	527127	528207	Newly-annotated lichen genomes	
umbilicaria.crustulosa.2_tyr632	umbilicaria.crustulosa.2	umbilicaria.crustulosa.2_10	852327	854576	Newly-annotated lichen genomes	
umbilicaria.crustulosa.3_tyr1110	umbilicaria.crustulosa.3	umbilicaria.crustulosa.3_12	821804	822688	Newly-annotated lichen genomes	
umbilicaria.vellea.1_tyr1419	umbilicaria.vellea.1	umbilicaria.vellea.1_128	1841	2777	Newly-annotated lichen genomes	
umbilicaria.phaea.1_tyr3806	umbilicaria.phaea.1	umbilicaria.phaea.1_20	529491	530626	Newly-annotated lichen genomes	

baeomycetales.sp..2_tyr423	baeomycetales.sp..2	baeomycetales.sp..2_40	148	2639	Newly-annotated lichen genomes	
conlig1_643740					Gluck-Thaler and Vogan (2024)	fam11
cytleu1_ROW05950.1					Gluck-Thaler and Vogan (2024)	fam11
melbic1_394156					Gluck-Thaler and Vogan (2024)	fam11
oidmai1_53117					Gluck-Thaler and Vogan (2024)	fam11
psesp.13_KFX99979.1					Gluck-Thaler and Vogan (2024)	clade03
usnea.ceratina.1_tyr3457	usnea.ceratina.1	usnea.ceratina.1_1245	237	1664	Newly-annotated lichen genomes	
usnea.florida.1_tyr2354	usnea.florida.1	usnea.florida.1_19	662503	665406	Newly-annotated lichen genomes	
aspicilia.laevata.1_tyr1038	aspicilia.laevata.1	aspicilia.laevata.1_129	9223	10248	Newly-annotated lichen genomes	
baeomycetales.sp..4_tyr1237	baeomycetales.sp..4	baeomycetales.sp..4_354	3893	11015	Newly-annotated lichen genomes	
xylographa.parallela.1_tyr3961	xylographa.parallela.1	xylographa.parallela.1_1	68438	71585	Newly-annotated lichen genomes	
chlaer1_TAQ85415.1					Gluck-Thaler and Vogan (2024)	fam11
rhyagr1_1870_1869					Gluck-Thaler and Vogan (2024)	n.d.
lacari1_TVY14354.1					Gluck-Thaler and Vogan (2024)	fam11
coccar1_81378					Gluck-Thaler and Vogan (2024)	fam11
dotsep1_32084					Gluck-Thaler and Vogan (2024)	fam11
neucra4_2977					Gluck-Thaler and Vogan (2024)	fam11
diaamp1_tyr4647					Gluck-Thaler and Vogan (2024)	fam11
fusnan1_tyr6095					Gluck-Thaler and Vogan (2024)	fam11
fustja1_KAF5616136.1					Gluck-Thaler and Vogan (2024)	fam11

fusxyl1_KAG5773589.1					Gluck-Thaler and Vogan (2024)	fam11
diabat1_KAG8161438.1					Gluck-Thaler and Vogan (2024)	fam11
psesp.4_703					Gluck-Thaler and Vogan (2024)	fam11
leprocaulon.nicholsiae.1_tyr3065	leprocaulon.nicholsiae.1	leprocaulon.nicholsiae.1_96	3486	4552	Newly-annotated lichen genomes	
cladonia.furcata.1_tyr1068	cladonia.furcata.1	cladonia.furcata.1_1079	4268	5381	Newly-annotated lichen genomes	
xylographa.bjoerkii.1_tyr1691	xylographa.bjoerkii.1	xylographa.bjoerkii.1_364	151655	152878	Newly-annotated lichen genomes	
cergua1_330854					Gluck-Thaler and Vogan (2024)	clade03
crycur1_tyr4496					Gluck-Thaler and Vogan (2024)	clade03
amapse1_tyr227					Gluck-Thaler and Vogan (2024)	fam11
antflo1_EPQ28709.1					Gluck-Thaler and Vogan (2024)	fam11
jamsp.1_185530					Gluck-Thaler and Vogan (2024)	fam11
meimil1_154462					Gluck-Thaler and Vogan (2024)	fam11
pseflo1_6738					Gluck-Thaler and Vogan (2024)	fam11
viopal1_370908					Gluck-Thaler and Vogan (2024)	fam11
wilmik1_644492					Gluck-Thaler and Vogan (2024)	clade02
aursub3_EJD38325.1					Gluck-Thaler and Vogan (2024)	fam11
monsp.5_RYP50627.1_RYP50628.1					Gluck-Thaler and Vogan (2024)	n.d.
mycchl1_tyr8389					Gluck-Thaler and Vogan (2024)	fam11
CryptonF-1_RhMu					Gluck-Thaler and Vogan (2024)	
CryptonF-2_PS_1p					Gluck-Thaler and Vogan (2024)	
CryptonF-5_PI_1p						

Table S9. Captain sequences assigned to the newly defined Tangerine family. The full set of captains (both representative and not) are listed.

Captain Sequence ID	Taxon	Genome	Source
acanthothecis.fontana.1_tyr1446	Ostropales	acanthothecis.fontana.1	Newly-annotated lichen genomes
acanthothecis.fontana.1_tyr2209	Ostropales	acanthothecis.fontana.1	Newly-annotated lichen genomes
acarospora.sp..1_tyr498	Acarosporales	acarospora.sp..1	Newly-annotated lichen genomes
agyrium.rufum.1_tyr287	Pertusariales	agyrium.rufum.1	Newly-annotated lichen genomes
buellia.spuria.2_tyr3704	Caliciales	buellia.spuria.2	Newly-annotated lichen genomes
byssoloma.meadii.1_tyr1250	Lecanorales	byssoloma.meadii.1	Newly-annotated lichen genomes
calogaya.arnoldii.1_tyr1881	Teloschistales	calogaya.arnoldii.1	Newly-annotated lichen genomes
calogaya.arnoldii.2_tyr1401	Teloschistales	calogaya.arnoldii.2	Newly-annotated lichen genomes
caloplaca.aetnensis.1_tyr1915	Teloschistales	caloplaca.aetnensis.1	Newly-annotated lichen genomes
caloplaca.sp..2_tyr2406	Teloschistales	caloplaca.sp..2	Newly-annotated lichen genomes
cladonia.rangiferina.1_tyr305	Lecanorales	cladonia.rangiferina.1	Newly-annotated lichen genomes
cyanodermella.asteris.1_tyr1869	Ostropales	cyanodermella.asteris.1	Newly-annotated lichen genomes
cyanodermella.asteris.1_tyr2814	Ostropales	cyanodermella.asteris.1	Newly-annotated lichen genomes
cyanodermella.asteris.1_tyr3466	Ostropales	cyanodermella.asteris.1	Newly-annotated lichen genomes
dibaeis.baeomyces.1_tyr3092	Pertusariales	dibaeis.baeomyces.1	Newly-annotated lichen genomes
dibaeis.sorediata.1_tyr2623	Pertusariales	dibaeis.sorediata.1	Newly-annotated lichen genomes
dibbae1_5584_5585	Pertusariales	dibbae1	Gluck-Thaler and Vogan (2024)
fissurina.insidiosa.1_tyr1461	Ostropales	fissurina.insidiosa.1	Newly-annotated lichen genomes
gallowayella.fulva.1_tyr2297	Teloschistales	gallowayella.fulva.1	Newly-annotated lichen genomes
gomphillus.americanus.1_tyr1053	Ostropales	gomphillus.americanus.1	Newly-annotated lichen genomes
gomphillus.americanus.2_tyr1205	Ostropales	gomphillus.americanus.2	Newly-annotated lichen genomes
gomphillus.calycioides.1_tyr105	Ostropales	gomphillus.calycioides.1	Newly-annotated lichen genomes
gomphillus.calycioides.1_tyr2843	Ostropales	gomphillus.calycioides.1	Newly-annotated lichen genomes
grascr1_11383	Ostropales	grascr1	Gluck-Thaler and Vogan (2024)
grascr1_687	Ostropales	grascr1	Gluck-Thaler and Vogan (2024)

gyalolechia.marmorata.1_tyr2081	Teloschistales	gyalolechia.marmorata.1	Newly-annotated lichen genomes
gyalolechia.marmorata.1_tyr3049	Teloschistales	gyalolechia.marmorata.1	Newly-annotated lichen genomes
icmadophila.ericetorum.1_tyr1628	Pertusariales	icmadophila.ericetorum.1	Newly-annotated lichen genomes
icmadophila.ericetorum.1_tyr3276	Pertusariales	icmadophila.ericetorum.1	Newly-annotated lichen genomes
icmadophila.ericetorum.1_tyr3921	Pertusariales	icmadophila.ericetorum.1	Newly-annotated lichen genomes
ionaspis.lacustris.1_tyr4127	Hymeneliales	ionaspis.lacustris.1	Newly-annotated lichen genomes
kuettlingeria.erythrocarpa.1_tyr701	Teloschistales	kuettlingeria.erythrocarpa.1	Newly-annotated lichen genomes
laspus2_SLM39506.1	Umbilicariales	laspus2	Gluck-Thaler and Vogan (2024)
lecanorales.sp..3_tyr2207	Lecanorales	lecanorales.sp..3	Newly-annotated lichen genomes
lecanorales.sp..6_tyr1295	Lecanorales	lecanorales.sp..6	Newly-annotated lichen genomes
letharia.columbiana.1_tyr1459	Lecanorales	letharia.columbiana.1	Newly-annotated lichen genomes
leucodecton.sp..1_tyr2657	Ostropales	leucodecton.sp..1	Newly-annotated lichen genomes
nadvornikia.sorediata.1_tyr2598	Ostropales	nadvornikia.sorediata.1	Newly-annotated lichen genomes
nadvornikia.sorediata.1_tyr3603	Ostropales	nadvornikia.sorediata.1	Newly-annotated lichen genomes
nadvornikia.sorediata.1_tyr4112	Ostropales	nadvornikia.sorediata.1	Newly-annotated lichen genomes
XANPAOZ2100_002162-T1	Teloschistales	X. parietina gXanPari1	Tangerine element from X. parietina
pertusaria.obruta.1_tyr2146	Pertusariales	pertusaria.obruta.1	Newly-annotated lichen genomes
pertusaria.plittiana.1_tyr1206	Pertusariales	pertusaria.plittiana.1	Newly-annotated lichen genomes
phlyctis.petraea.1_tyr16	Gyalectales	phlyctis.petraea.1	Newly-annotated lichen genomes
polycauliona.sp..2_tyr3658	Teloschistales	polycauliona.sp..2	Newly-annotated lichen genomes
porina.heterospora.1_tyr2130	Ostropales	porina.heterospora.1	Newly-annotated lichen genomes
pseudosagedia.sp..1_tyr1320	Pertusariales	pseudosagedia.sp..1	Newly-annotated lichen genomes
XANPARI20_008752-T1	Teloschistales	X. parietina gXanPari20	Tangerine element from X. parietina
squamulea.sp..1_tyr2986	Teloschistales	squamulea.sp..1	Newly-annotated lichen genomes
stictis.urceolata.1_tyr128	Ostropales	stictis.urceolata.1	Newly-annotated lichen genomes
stictis.urceolata.1_tyr1442	Ostropales	stictis.urceolata.1	Newly-annotated lichen genomes
stictis.urceolata.2_tyr224	Ostropales	stictis.urceolata.2	Newly-annotated lichen genomes
stictis.urceolata.2_tyr3715	Ostropales	stictis.urceolata.2	Newly-annotated lichen genomes
teloschistales.sp..1_tyr3330	Teloschistales	teloschistales.sp..1	Newly-annotated lichen genomes

teloschistes.chrysophthalmus.1_tyr1428	Teloschistales	teloschistes.chrysophthalmus.1	Newly-annotated lichen genomes
teloschistes.chrysophthalmus.1_tyr2102	Teloschistales	teloschistes.chrysophthalmus.1	Newly-annotated lichen genomes
teloschistes.chrysophthalmus.1_tyr980	Teloschistales	teloschistes.chrysophthalmus.1	Newly-annotated lichen genomes
teloschistes.chrysophthalmus.2_tyr2422	Teloschistales	teloschistes.chrysophthalmus.2	Newly-annotated lichen genomes
teloschistes.chrysophthalmus.2_tyr3795	Teloschistales	teloschistes.chrysophthalmus.2	Newly-annotated lichen genomes
teloschistes.exilis.1_tyr3101	Teloschistales	teloschistes.exilis.1	Newly-annotated lichen genomes
teloschistes.exilis.1_tyr3137	Teloschistales	teloschistes.exilis.1	Newly-annotated lichen genomes
teloschistes.exilis.1_tyr919	Teloschistales	teloschistes.exilis.1	Newly-annotated lichen genomes
teloschistes.exilis.2_tyr1555	Teloschistales	teloschistes.exilis.2	Newly-annotated lichen genomes
teloschistes.exilis.2_tyr2659	Teloschistales	teloschistes.exilis.2	Newly-annotated lichen genomes
teloschistes.exilis.2_tyr4051	Teloschistales	teloschistes.exilis.2	Newly-annotated lichen genomes
teloschistes.exilis.2_tyr4052	Teloschistales	teloschistes.exilis.2	Newly-annotated lichen genomes
teloschistes.exilis.3_tyr1984	Teloschistales	teloschistes.exilis.3	Newly-annotated lichen genomes
teloschistes.exilis.3_tyr2312	Teloschistales	teloschistes.exilis.3	Newly-annotated lichen genomes
teloschistes.exilis.3_tyr394	Teloschistales	teloschistes.exilis.3	Newly-annotated lichen genomes
teloschistes.exilis.3_tyr4193	Teloschistales	teloschistes.exilis.3	Newly-annotated lichen genomes
teloschistes.exilis.3_tyr560	Teloschistales	teloschistes.exilis.3	Newly-annotated lichen genomes
teloschistes.flavicans.2_tyr4102	Teloschistales	teloschistes.flavicans.2	Newly-annotated lichen genomes
teloschistes.hypoglaucus.1_tyr3519	Teloschistales	teloschistes.hypoglaucus.1	Newly-annotated lichen genomes
teloschistes.peruensis.1_tyr1536	Teloschistales	teloschistes.peruensis.1	Newly-annotated lichen genomes
teloschistes.peruensis.1_tyr2728	Teloschistales	teloschistes.peruensis.1	Newly-annotated lichen genomes
teloschistes.peruensis.1_tyr3340	Teloschistales	teloschistes.peruensis.1	Newly-annotated lichen genomes
teloschistes.peruensis.1_tyr3348	Teloschistales	teloschistes.peruensis.1	Newly-annotated lichen genomes
teloschistes.peruensis.1_tyr4017	Teloschistales	teloschistes.peruensis.1	Newly-annotated lichen genomes
teloschistes.peruensis.1_tyr623	Teloschistales	teloschistes.peruensis.1	Newly-annotated lichen genomes
umbilicaria.iberica.1_tyr757	Umbilicariales	umbilicaria.iberica.1	Newly-annotated lichen genomes
umbilicaria.spodochroa.4_tyr3267	Umbilicariales	umbilicaria.spodochroa.4	Newly-annotated lichen genomes
umbpus1_103454	Umbilicariales	umbpus1	Gluck-Thaler and Vogan (2024)
variospora.aurantia.1_tyr2779	Teloschistales	variospora.aurantia.1	Newly-annotated lichen genomes
xanthocarpia.lactea.1_tyr1907	Teloschistales	xanthocarpia.lactea.1	Newly-annotated lichen genomes
xanthocarpia.ochracea.1_tyr156	Teloschistales	xanthocarpia.ochracea.1	Newly-annotated lichen genomes

xanthoria.mediterranea.1_tyr2790	Teloschistales	xanthoria.mediterranea.1	Newly-annotated lichen genomes
xanthoria.sp..2_tyr633	Teloschistales	xanthoria.sp..2	Newly-annotated lichen genomes
XANPAGTX0501_001716-T1	Teloschistales	X. parietina GTX0501	Tangerine element from X. parietina

Table S10. Columns of annotated domains and active sites in the alignment files for the 'tangerine' and 'non-tangerine' sequence clusters.

Alignment File	CB Domain	CAT Domain	Active Site 1	Active Site 2	Active Site 3	Active Site 4	Active Site 5	Active Site 6
sub_reps_plus_non_tangerine_cluster_caps.mafft	302-1193	1664-5311	2065	3330	4597	4627	5124	5304
tangerine_cluster_caps.mafft	302-1193	1664-5311	2065	3330	4558	4561	5124	5304

Table S11. Results of Foldseek search of the Tangerine captain (XANPAOZ2100_002162-T1) against the PDB database.

query	target	Probability of homology	E-value	Tm-score	Sequence Identity	description
XANPAOZ2100_002162-T1	5hxy-assembly4_D	1	0.281	91	31.3	Crystal structure of XerA recombinase
XANPAOZ2100_002162-T1	5hxy-assembly3_C	0.99	0.467	84	31.8	Crystal structure of XerA recombinase
XANPAOZ2100_002162-T1	5u91-assembly1_F	0.97	0.314	77	18.2	Crystal structure of Tre/loxLTR complex
XANPAOZ2100_002162-T1	5j0i-assembly1_A	0.07	8.82	27	16.6	De novo design of protein homo-oligomers with modular hydrogen bond network-mediated specificity
XANPAOZ2100_002162-T1	8cok-assembly1_B	0.07	4.23	27	13.5	Structural analysis of ING3 protein and its binding to histone H3
XANPAOZ2100_002162-T1	2pih-assembly1_B	0.06	9.33	26	14.1	Crystal structure of Protein ymcA from Bacillus subtilis, NorthEast Structural Genomics target SR375
XANPAOZ2100_002162-T1	8btk-assembly1_TC	0.05	8.82	24	9.7	Structure of the TRAP complex with the Sec translocon and a translating ribosome

Table S12. Results of blast search for two HGT candidates: methyltransferase (XANPAOZ2100_002166-T1) and NLR (XANPAOZ2100_002167-T1). For each gene, we ran two searches: against core_nt database without any restriction and against nr/nt collection with results limited to Lecanoromycetes.

Query	Subject	Description	Taxonomy	Max Score	Total Score	Query Cover	E value	Per. ident	Acc. Len	Accession
XANPAOZ2100_002166-T1	core_nt	Cladophialophora yegresii CBS 114405 uncharacterized protein (A1O7_06482), partial mRNA	non-Lecanoromycete	360	360	0.96	3E-123	75.68	726	XM_007760484.1
XANPAOZ2100_002166-T1	core_nt	Fonsecaea erecta hypothetical protein (AYL99_06618), partial mRNA	non-Lecanoromycete	358	358	0.97	6E-123	74.11	675	XM_018838127.1
XANPAOZ2100_002166-T1	core_nt	Cladophialophora psammophila CBS 110553 uncharacterized protein (A1O5_08129), partial mRNA	non-Lecanoromycete	358	358	0.99	1E-122	73.68	699	XM_007748713.1
XANPAOZ2100_002166-T1	core_nt	Endocarpon pusillum Z07020 uncharacterized protein (EPUS_08988), partial mRNA	non-Lecanoromycete	355	355	0.98	3E-121	73.45	693	XM_007805357.1
XANPAOZ2100_002166-T1	core_nt	Fonsecaea multimorphosa CBS 102226 uncharacterized protein (Z520_07803), partial mRNA	non-Lecanoromycete	352	352	0.98	3E-120	73.45	687	XM_016778300.1
XANPAOZ2100_002166-T1	core_nt	Exophiala xenobiotica uncharacterized protein (PV05_08005), mRNA	non-Lecanoromycete	353	353	0.92	3E-119	76.78	975	XM_013457493.1
XANPAOZ2100_002166-T1	core_nt	Cladophialophora carrionii CBS 160.54 uncharacterized protein (G647_04799), partial mRNA	non-Lecanoromycete	346	346	1	7E-118	69.87	690	XM_008729136.1
XANPAOZ2100_002166-T1	core_nt	Exophiala mesophila uncharacterized protein (PV10_02689), partial mRNA	non-Lecanoromycete	348	348	1	8E-118	70.87	872	XM_016367035.1
XANPAOZ2100_002166-T1	core_nt	Cyphellophora europaea CBS 101466 uncharacterized protein (HMPREF1541_09139), partial mRNA	non-Lecanoromycete	344	344	0.91	3E-117	72.73	672	XM_008713814.1

XANPAOZ2100_002166-T1	core_nt	Exophiala oligosperma uncharacterized protein (PV06_08307), partial mRNA	non-Lecanoromycete	352	352	0.95	7E-117	73.64	1401	XM_016409626.1
XANPAOZ2100_002166-T1	core_nt	Exophiala aquamarina CBS 119918 uncharacterized protein (A1O9_03817), partial mRNA	non-Lecanoromycete	337	337	1	4E-114	69	774	XM_013406110.1
XANPAOZ2100_002166-T1	core_nt	Cladophialophora yegresii CBS 114405 uncharacterized protein (A1O7_03580), partial mRNA	non-Lecanoromycete	331	331	1	8E-112	67.53	699	XM_007757599.1
XANPAOZ2100_002166-T1	core_nt	Hypoxylon trugodes S-adenosyl-L-methionine-dependent methyltransferase (F4822DRAFT_430822), partial mRNA	non-Lecanoromycete	296	296	0.92	3E-98	63.68	648	XM_051461370.1
XANPAOZ2100_002166-T1	core_nt	Annulohypoxylon truncatum S-adenosyl-L-methionine-dependent methyltransferase (F4807DRAFT_144987), mRNA	non-Lecanoromycete	296	296	0.92	7E-97	62.09	947	XM_047993147.1
XANPAOZ2100_002166-T1	core_nt	Annulohypoxylon maeteangense S-adenosyl-L-methionine-dependent methyltransferase (GGS22DRAFT_156084), mRNA	non-Lecanoromycete	292	292	0.91	1E-95	64.76	900	XM_047960313.1
XANPAOZ2100_002166-T1	core_nt	Daldinia vernicosa S-adenosyl-L-methionine-dependent methyltransferase (F5Y00DRAFT_208395), mRNA	non-Lecanoromycete	293	293	0.93	3E-95	62.44	1047	XM_048005468.1
XANPAOZ2100_002166-T1	core_nt	Daldinia loculata S-adenosyl-L-methionine-dependent methyltransferase (F4817DRAFT_205109), mRNA	non-Lecanoromycete	291	291	0.93	5E-95	62.44	937	XM_049298629.1
XANPAOZ2100_002166-T1	core_nt	Daldinia childiae Endothelin-converting enzyme 2 (GL218_01013), partial mRNA	non-Lecanoromycete	287	287	0.93	8E-95	61.97	648	XM_033577459.1

XANPAOZ2100_002166-T1	core_nt	Daldinia decipiens S-adenosyl-L-methionine-dependent methyltransferase (F4813DRAFT_368903), mRNA	non-Lecanoromycete	291	291	0.93	7E-94	61.5	1152	XM_049244375.1
XANPAOZ2100_002166-T1	core_nt	Thyridium curvatum uncharacterized protein (E0L32_004868), partial mRNA	non-Lecanoromycete	283	283	0.93	4E-93	60.83	666	XM_031139326.1
XANPAOZ2100_002166-T1	core_nt	Verticillium nonalfalfae uncharacterized protein (D7B24_005222), partial mRNA	non-Lecanoromycete	281	281	0.97	8E-92	59.19	744	XM_028639381.1
XANPAOZ2100_002166-T1	core_nt	Verticillium alfalfae VaMs.102 conserved hypothetical protein mRNA	non-Lecanoromycete	281	281	0.97	1E-91	59.19	817	XM_003001469.1
XANPAOZ2100_002166-T1	core_nt	Verticillium dahliae VdLs.17 uncharacterized protein (VDAG_08232), mRNA	non-Lecanoromycete	279	279	0.93	1E-90	60.75	817	XM_009657609.1
XANPAOZ2100_002166-T1	core_nt	Daldinia caldariorum S-adenosyl-L-methionine-dependent methyltransferase (F4812DRAFT_353580), mRNA	non-Lecanoromycete	282	282	0.93	1E-90	59.62	1086	XM_047931756.1
XANPAOZ2100_002166-T1	core_nt	Colletotrichum truncatum methyltransferase type 11 (CTRU02_207930), partial mRNA	non-Lecanoromycete	275	275	0.9	3E-90	58.85	654	XM_070204993.1
XANPAOZ2100_002166-T1	core_nt	Colletotrichum chrysophilum uncharacterized protein (COL26b_011736), partial mRNA	non-Lecanoromycete	275	275	0.9	6E-90	59.81	654	XM_053184397.1
XANPAOZ2100_002166-T1	core_nt	Colletotrichum aenigma EEF1A lysine methyltransferase 4 (Eef1akmt4), partial mRNA	non-Lecanoromycete	275	275	0.9	6E-90	59.81	654	XM_037327971.1
XANPAOZ2100_002166-T1	core_nt	Colletotrichum siamense EEF1A lysine methyltransferase 4 (Eef1akmt4), partial mRNA	non-Lecanoromycete	273	273	0.9	3E-89	59.81	654	XM_036644624.1

XANPAOZ2100_002166-T1	core_nt	Colletotrichum gloeosporioides uncharacterized protein (GCG54_00013103), partial mRNA	non-Lecanoromycete	273	273	0.9	3E-89	59.81	654	XM_045412963.1
XANPAOZ2100_002166-T1	core_nt	Colletotrichum fructicola Nara gc5 EEF1A lysine methyltransferase 4 (CGGC5_v011631), partial mRNA	non-Lecanoromycete	272	272	0.9	6E-89	59.33	654	XM_032027884.1
XANPAOZ2100_002166-T1	core_nt	Colletotrichum higginsianum IMI 349063 uncharacterized protein (CH63R_09706), partial mRNA	non-Lecanoromycete	272	272	0.92	8E-89	56.81	684	XM_018304680.1
XANPAOZ2100_002166-T1	core_nt	Colletotrichum karsti uncharacterized protein (CkaCkLH20_13122), partial mRNA	non-Lecanoromycete	270	270	0.92	8E-88	57.28	654	XM_038895833.1
XANPAOZ2100_002166-T1	core_nt	Colletotrichum spaethianum EEF1A lysine methyltransferase 4 (ColSpa_12423), partial mRNA	non-Lecanoromycete	268	268	0.92	2E-87	57.75	654	XM_049278635.1
XANPAOZ2100_002166-T1	core_nt	Purpureocillium lilacinum uncharacterized protein (PLICBS_004226), partial mRNA	non-Lecanoromycete	268	268	0.95	2E-87	57.8	657	XM_018327050.1
XANPAOZ2100_002166-T1	core_nt	Boeremia exigua S-adenosyl-L-methionine-dependent methyltransferase (C7974DRAFT_394160), partial mRNA	non-Lecanoromycete	269	269	0.93	6E-87	57.94	819	XM_046141850.1
XANPAOZ2100_002166-T1	core_nt	Colletotrichum graminicola M1.001 uncharacterized protein (GLRG_11413), partial mRNA	non-Lecanoromycete	266	266	0.9	1E-86	57.89	660	XM_008102097.1
XANPAOZ2100_002166-T1	core_nt	Macroventuria anomochaeta uncharacterized protein (BU25DRAFT_345099), partial mRNA	non-Lecanoromycete	266	266	0.9	2E-86	59.13	645	XM_033703025.1

XANPAOZ2100_002166-T1	core_nt	Colletotrichum destructivum Putative methyltransferase type 11, S-adenosyl-L-methionine-dependent methyltransferase superfamily (CDEST_14275), mRNA	non-Lecanoromycete	273	273	0.92	3E-86	56.81	1269	XM_062930431.1
XANPAOZ2100_002166-T1	core_nt	Didymella exigua CBS 183.55 uncharacterized protein (M421DRAFT_73186), partial mRNA	non-Lecanoromycete	265	265	0.91	5E-86	59.24	654	XM_033596943.1
XANPAOZ2100_002166-T1	core_nt	Colletotrichum paranaense uncharacterized protein (CPAR01_14215), partial mRNA	non-Lecanoromycete	264	264	0.9	2E-85	57.89	660	XM_060498466.1
XANPAOZ2100_002166-T1	core_nt	Ascochyta rabiei uncharacterized protein (EKO05_0004562), partial mRNA	non-Lecanoromycete	263	263	0.9	2E-85	58.65	645	XM_038938776.1
XANPAOZ2100_002166-T1	core_nt	Colletotrichum fioriniae uncharacterized protein (N0V96_010881), partial mRNA	non-Lecanoromycete	261	261	0.9	2E-84	56.94	654	XM_053195826.1
XANPAOZ2100_002166-T1	core_nt	Fulvia fulva EEF1A lysine methyltransferase 4 (CLAFUR5_05086), partial mRNA	non-Lecanoromycete	261	261	0.94	2E-84	57.14	657	XM_047904234.1
XANPAOZ2100_002166-T1	core_nt	Colletotrichum costaricense uncharacterized protein (CCOS01_11420), partial mRNA	non-Lecanoromycete	260	260	0.9	4E-84	56.46	660	XM_060459573.1
XANPAOZ2100_002166-T1	core_nt	Colletotrichum tamarilloi uncharacterized protein (CTAM01_09877), partial mRNA	non-Lecanoromycete	260	260	0.9	4E-84	57.21	660	XM_060525893.1
XANPAOZ2100_002166-T1	core_nt	Lasiodiplodia theobromae Methyltransferase type 11 (LTHEOB_7030), partial mRNA	non-Lecanoromycete	260	260	0.9	5E-84	59.9	669	XM_035516352.1

XANPAOZ2100_002166-T1	core_nt	Colletotrichum lupini uncharacterized protein (CLUP02_13032), partial mRNA	non-Lecanoromycete	259	259	0.9	8E-84	56.73	660	XM_049291990.1
XANPAOZ2100_002166-T1	core_nt	Alternaria arborescens hypothetical protein (AA0111_g2457), partial mRNA	non-Lecanoromycete	258	258	0.91	3E-83	57.82	657	XM_028648230.1
XANPAOZ2100_002166-T1	core_nt	Alternaria postmessia uncharacterized protein (J4E82_001247), partial mRNA	non-Lecanoromycete	258	258	0.91	3E-83	57.82	657	XM_051727562.1
XANPAOZ2100_002166-T1	core_nt	Alternaria atra uncharacterized protein (ALTATR162_LOCUS11096), partial mRNA	non-Lecanoromycete	258	258	0.91	3E-83	58.29	657	XM_043318738.1
XANPAOZ2100_002166-T1	core_nt	Purpureocillium takamizusanense uncharacterized protein (JDV02_009070), mRNA	non-Lecanoromycete	264	264	0.91	5E-83	58.37	1212	XM_047990709.1
XANPAOZ2100_002166-T1	core_nt	Exserohilum turcica Et28A uncharacterized protein (SETTUDRAFT_137107), partial mRNA	non-Lecanoromycete	257	257	0.91	6E-83	55.45	677	XM_008029235.1
XANPAOZ2100_002166-T1	core_nt	Cercospora beticola uncharacterized protein (RHO25_008349), partial mRNA	non-Lecanoromycete	256	256	0.91	1E-82	55.98	645	XM_023600481.2
XANPAOZ2100_002166-T1	core_nt	Alternaria metachromatica uncharacterized protein (J4E83_008044), partial mRNA	non-Lecanoromycete	256	256	0.92	1E-82	56.81	657	XM_049334941.1
XANPAOZ2100_002166-T1	core_nt	Alternaria ethzedia uncharacterized protein (J4E87_001333), partial mRNA	non-Lecanoromycete	256	256	0.92	1E-82	57.28	657	XM_049372979.1
XANPAOZ2100_002166-T1	core_nt	Alternaria conjuncta uncharacterized protein (J4E85_004224), partial mRNA	non-Lecanoromycete	256	256	0.92	2E-82	56.81	657	XM_051468485.1

XANPAOZ2100_002166-T1	core_nt	Alternaria hordeiaustralica uncharacterized protein (J4E84_007788), partial mRNA	non-Lecanoromycete	256	256	0.92	2E-82	56.81	657	XM_049390368.1
XANPAOZ2100_002166-T1	core_nt	Dothidotthia symphoricarpi CBS 119687 uncharacterized protein (P153DRAFT_364327), mRNA	non-Lecanoromycete	262	262	0.96	2E-82	54.5	1200	XM_033667573.1
XANPAOZ2100_002166-T1	core_nt	Alternaria infectoria uncharacterized protein (J4E92_003793), partial mRNA	non-Lecanoromycete	256	256	0.92	2E-82	56.81	657	XM_051495187.1
XANPAOZ2100_002166-T1	core_nt	Parastagonospora nodorum SN15 hypothetical protein (SNOG_02149), partial mRNA	non-Lecanoromycete	266	266	0.96	3E-82	56.11	1632	XM_001792715.1
XANPAOZ2100_002166-T1	core_nt	Diplodia corticola endothelin-converting enzyme 2 (BKCO1_4000160), partial mRNA	non-Lecanoromycete	255	255	0.9	3E-82	57.97	651	XM_020275583.1
XANPAOZ2100_002166-T1	core_nt	Colletotrichum scovillei uncharacterized protein (HER10_EVM0005217), mRNA	non-Lecanoromycete	259	259	0.9	4E-82	56.94	1026	XM_035474839.1
XANPAOZ2100_002166-T1	core_nt	Colletotrichum navitas S-adenosyl-L-methionine-dependent methyltransferase (LY79DRAFT_618709), mRNA	non-Lecanoromycete	261	261	0.9	6E-82	56.6	1187	XM_060562026.1
XANPAOZ2100_002166-T1	core_nt	Colletotrichum phormii S-adenosyl-L-methionine-dependent methyltransferase (BDP81DRAFT_328824), partial mRNA	non-Lecanoromycete	254	254	0.9	8E-82	55.02	669	XM_060585252.1
XANPAOZ2100_002166-T1	core_nt	Alternaria novae-zelandiae uncharacterized protein (J4E88_008233), partial mRNA	non-Lecanoromycete	254	254	0.92	9E-82	56.81	657	XM_049401844.1
XANPAOZ2100_002166-T1	core_nt	Alternaria triticimaculans uncharacterized protein	non-Lecanoromycete	254	254	0.92	9E-82	56.34	657	XM_049366035.1

		(J4E78_005415), partial mRNA								
XANPAOZ2100_002166-T1	core_nt	Diplodia seriata uncharacterized protein (SLS55_004925), partial mRNA	non-Lecanoromycete	254	254	0.9	1E-81	58.65	663	XM_066776377.1
XANPAOZ2100_002166-T1	core_nt	Alternaria alternata hypothetical protein mRNA	non-Lecanoromycete	258	258	0.91	2E-81	57.82	1041	XM_018525700.1
XANPAOZ2100_002166-T1	core_nt	Alternaria ventricosa uncharacterized protein (J4E93_006292), partial mRNA	non-Lecanoromycete	253	253	0.92	2E-81	56.81	657	XM_049344209.1
XANPAOZ2100_002166-T1	core_nt	Colletotrichum acutatum S-adenosyl-L-methionine-dependent methyltransferase (BDZ83DRAFT_626511), mRNA	non-Lecanoromycete	256	256	0.9	2E-81	56.73	907	XM_060508568.1
XANPAOZ2100_002166-T1	core_nt	Cercospora kikuchii uncharacterized protein (CKM354_000597400), partial mRNA	non-Lecanoromycete	253	253	0.91	3E-81	55.98	645	XM_044801267.1
XANPAOZ2100_002166-T1	core_nt	Alternaria burnsii methyltransferase type 11 (GT037_008621), partial mRNA	non-Lecanoromycete	262	262	0.91	6E-81	58.29	1572	XM_038933668.1
XANPAOZ2100_002166-T1	core_nt	Alternaria rosae S-adenosyl-L-methionine-dependent methyltransferase (BKA58DRAFT_234400), mRNA	non-Lecanoromycete	254	254	0.96	7E-80	54.3	1091	XM_046166554.1
XANPAOZ2100_002166-T1	core_nt	Ramularia collo-cygni uncharacterized protein (RCC_03825), partial mRNA	non-Lecanoromycete	249	249	0.97	1E-79	52.21	684	XM_023769108.1
XANPAOZ2100_002166-T1	core_nt	Microdochium trichocladiopsis uncharacterized protein (B0I36DRAFT_319463), mRNA	non-Lecanoromycete	254	254	0.9	1E-79	55.26	1080	XM_046153421.1
XANPAOZ2100_002166-T1	core_nt	Madurella fahalii uncharacterized protein	non-Lecanoromycete	246	246	0.92	1E-78	55.61	657	XM_071059100.1

		(MFIFM68171_03679), partial mRNA								
XANPAOZ2100_002166-T1	core_nt	Aureobasidium melanogenum CBS 110374 uncharacterized protein (M437DRAFT_68118), partial mRNA	non-Lecanoromycete	246	246	0.88	1E-78	56.65	675	XM_041025059.1
XANPAOZ2100_002166-T1	core_nt	Trichoderma aggressivum f. europaeum uncharacterized protein (Triagg1_10803), partial mRNA	non-Lecanoromycete	247	247	0.92	1E-78	55.4	771	XM_062894915.1
XANPAOZ2100_002166-T1	core_nt	Trichoderma gamsii hypothetical protein (TGAM01_v200291), partial mRNA	non-Lecanoromycete	245	245	0.92	4E-78	55.87	669	XM_024548531.1
XANPAOZ2100_002166-T1	core_nt	Aureobasidium namibiae CBS 147.97 uncharacterized protein (M436DRAFT_55093), partial mRNA	non-Lecanoromycete	244	244	0.88	6E-78	57.14	672	XM_013568370.1
XANPAOZ2100_002166-T1	core_nt	Grosmannia clavigera kw1407 uncharacterized protein (CMQ_7880), partial mRNA	non-Lecanoromycete	244	244	0.88	7E-78	55.61	642	XM_014313520.1
XANPAOZ2100_002166-T1	core_nt	Phyllosticta capitalensis S-adenosyl-L-methionine-dependent methyltransferase (BKA78DRAFT_239229), partial mRNA	non-Lecanoromycete	243	243	0.91	7E-78	58.37	597	XM_066883896.1
XANPAOZ2100_002166-T1	core_nt	Colletotrichum abscissum uncharacterized protein (CABS01_07642), partial mRNA	non-Lecanoromycete	250	250	0.9	2E-77	53.92	1245	XM_060541004.1
XANPAOZ2100_002166-T1	core_nt	Alternaria dauci uncharacterized protein (ACET3X_007311), partial mRNA	non-Lecanoromycete	243	243	0.91	3E-77	54.98	669	XM_069454196.1
XANPAOZ2100_002166-T1	core_nt	Fusarium graminearum PH-1 uncharacterized protein (FGSG_11344), partial mRNA	non-Lecanoromycete	242	242	0.93	4E-77	51.39	648	XM_011327594.1

XANPAOZ2100_002166-T1	core_nt	Trichoderma atroviride uncharacterized protein (TrAtP1_006181), mRNA	non-Lecanoromycete	248	248	0.92	2E-76	56.34	1353	XM_014082818.2
XANPAOZ2100_002166-T1	core_nt	Fusarium flagelliforme S-adenosyl-L-methionine-dependent methyltransferase (B0J16DRAFT_290150), mRNA	non-Lecanoromycete	238	238	0.93	3E-75	51.85	707	XM_046124238.1
XANPAOZ2100_002166-T1	core_nt	Fusarium poae uncharacterized protein (FPOAC1_007521), partial mRNA	non-Lecanoromycete	237	237	0.93	3E-75	51.39	648	XM_044851977.1
XANPAOZ2100_002166-T1	core_nt	Fusarium coffeatum uncharacterized protein (FIESC28_07298), partial mRNA	non-Lecanoromycete	237	237	0.93	5E-75	51.39	648	XM_031161439.1
XANPAOZ2100_002166-T1	core_nt	Colletotrichum jinshuiense isolate EsH8 chromosome 8	non-Lecanoromycete	258	258	0.92	7E-75	51.39	4210165	CP150811.1
XANPAOZ2100_002166-T1	core_nt	Exophiala viscosa endothelin-converting enzyme (EDD37DRAFT_644373), partial mRNA	non-Lecanoromycete	236	236	0.88	1E-74	52.71	636	XM_062817505.1
XANPAOZ2100_002166-T1	core_nt	Pseudocercospora fijiensis CIRAD86 uncharacterized protein (MYCFIDRAFT_86729), partial mRNA	non-Lecanoromycete	236	236	0.9	9E-74	55.24	882	XM_007930874.1
XANPAOZ2100_002166-T1	core_nt	Alternaria viburni uncharacterized protein (J4E79_002089), partial mRNA	non-Lecanoromycete	233	233	0.9	2E-73	51.21	651	XM_049350992.1
XANPAOZ2100_002166-T1	core_nt	Fusarium subglutinans endothelin-converting enzyme 2 (FSUBG_12914), partial mRNA	non-Lecanoromycete	232	232	0.92	4E-73	52.58	642	XM_036677750.1
XANPAOZ2100_002166-T1	core_nt	Alternaria arbusti uncharacterized protein (J4E86_006517), partial mRNA	non-Lecanoromycete	231	231	0.89	8E-73	50.73	651	XM_051447045.1

XANPAOZ2100_002166-T1	core_nt	Sporothrix brasiliensis 5110 uncharacterized protein (SPBR_05262), partial mRNA	non-Lecanoromycete	232	232	0.88	1E-72	49.56	717	XM_040763539.1
XANPAOZ2100_002166-T1	core_nt	Trichoderma virens Gv29-8 uncharacterized protein (TRIVIDRAFT_194210), partial mRNA	non-Lecanoromycete	231	231	0.96	1E-72	51.79	672	XM_014097172.1
XANPAOZ2100_002166-T1	core_nt	Neohortaea acidophila S-adenosyl-L-methionine-dependent methyltransferase (BDY17DRAFT_288441), mRNA	non-Lecanoromycete	237	237	0.91	2E-72	51.43	1249	XM_033732194.1
XANPAOZ2100_002166-T1	core_nt	Saxophila tyrrhenica uncharacterized protein (LTR77_005914), partial mRNA	non-Lecanoromycete	229	229	0.91	3E-72	51.43	633	XM_064803156.1
XANPAOZ2100_002166-T1	core_nt	Sporothrix schenckii 1099-18 S-adenosyl-L-methionine-dependent methyltransferase-like protein (SPSK_06566), partial mRNA	non-Lecanoromycete	229	229	0.88	8E-72	48.89	717	XM_016733268.1
XANPAOZ2100_002167-T1	core_nt	Cladophialophora psammophila CBS 110553 uncharacterized protein (A1O5_08130), partial mRNA	non-Lecanoromycete	1428	1428	0.79	0	61.77	3636	XM_007748714.1
XANPAOZ2100_002167-T1	core_nt	Fonsecaea multimorphosa CBS 102226 uncharacterized protein (Z520_11930), partial mRNA	non-Lecanoromycete	1409	1409	0.76	0	61.87	3636	XM_016782417.1
XANPAOZ2100_002167-T1	core_nt	Fonsecaea erecta hypothetical protein (AYL99_06617), partial mRNA	non-Lecanoromycete	1395	1395	0.79	0	59.54	3627	XM_018838126.1
XANPAOZ2100_002167-T1	core_nt	Cladophialophora yegresii CBS 114405 uncharacterized protein (A1O7_06481), partial mRNA	non-Lecanoromycete	1182	1182	0.75	0	54.04	3378	XM_007760483.1
XANPAOZ2100_002167-T1	core_nt	Scedosporium apiospermum uncharacterized protein (SAPIO_CDS1618), partial mRNA	non-Lecanoromycete	867	867	0.75	0	43.14	3552	XM_016784845.1

XANPAOZ2100_002167-T1	core_nt	Botrytis porri uncharacterized protein (EAF01_003706), mRNA	non-Lecanoromycete	793	793	0.77	0	40.71	4097	XM_038911960.1
XANPAOZ2100_002167-T1	core_nt	Talaromyces rugulosus uncharacterized protein (TRUGW13939_03858), partial mRNA	non-Lecanoromycete	790	790	0.76	0	39.43	3657	XM_035487036.1
XANPAOZ2100_002167-T1	core_nt	Botrytis fragariae putative ankyrin repeat protein (Bfra_005064), partial mRNA	non-Lecanoromycete	789	789	0.77	0	40.57	3723	XM_037335451.1
XANPAOZ2100_002167-T1	core_nt	Talaromyces atroroseus hypothetical protein (UA08_06898), partial mRNA	non-Lecanoromycete	786	786	0.76	0	40.16	3543	XM_020261979.1
XANPAOZ2100_002167-T1	core_nt	Sporothrix schenckii 1099-18 uncharacterized protein (SPSK_01385), partial mRNA	non-Lecanoromycete	772	772	0.73	0	41.25	3666	XM_016728300.1
XANPAOZ2100_002167-T1	core_nt	Lindgomyces ingoldianus uncharacterized protein (BDR25DRAFT_341809), partial mRNA	non-Lecanoromycete	768	768	0.75	0	39.98	3501	XM_033693645.1
XANPAOZ2100_002167-T1	core_nt	Capronia epimyces CBS 606.96 uncharacterized protein (A1O3_03008), partial mRNA	non-Lecanoromycete	759	759	0.73	0	40.44	3504	XM_007733148.1
XANPAOZ2100_002167-T1	core_nt	Rhinocladiella mackenziei CBS 650.93 uncharacterized protein (Z518_07228), partial mRNA	non-Lecanoromycete	749	749	0.75	0	39.42	3489	XM_013415357.1
XANPAOZ2100_002167-T1	core_nt	Thyridium curvatum uncharacterized protein (E0L32_005475), partial mRNA	non-Lecanoromycete	746	746	0.74	0	39.47	3528	XM_031140000.1
XANPAOZ2100_002167-T1	core_nt	Botrytis byssoidea uncharacterized protein (EAE97_003805), partial mRNA	non-Lecanoromycete	741	741	0.74	0	39.86	3492	XM_038874317.1
XANPAOZ2100_002167-T1	core_nt	Aspergillus lucknowensis uncharacterized protein (BJX67DRAFT_367453), mRNA	non-Lecanoromycete	728	728	0.76	0	39.72	4026	XM_071030526.1

XANPAOZ2100_002167-T1	core_nt	Penicillium psychrosexuale uncharacterized protein (N7518_001079), partial mRNA	non-Lecanoromycete	728	728	0.73	0	38.47	3750	XM_057182007.1
XANPAOZ2100_002167-T1	core_nt	Fusarium mangiferae uncharacterized protein (FMAN_02144), partial mRNA	non-Lecanoromycete	726	726	0.73	0	39.58	3516	XM_041835464.1
XANPAOZ2100_002167-T1	core_nt	Lithohypha guttulata uncharacterized protein (LTR64_008794), partial mRNA	non-Lecanoromycete	722	722	0.74	0	38.65	3258	XM_064903099.1
XANPAOZ2100_002167-T1	core_nt	Fusarium verticillioides 7600 hypothetical protein (FVEG_16082), partial mRNA	non-Lecanoromycete	713	713	0.72	0	38.22	3570	XM_018905323.1
XANPAOZ2100_002167-T1	core_nt	Truncatella angustata uncharacterized protein (BKA67DRAFT_525676), partial mRNA	non-Lecanoromycete	706	706	0.72	0	37.35	3300	XM_046098685.1
XANPAOZ2100_002167-T1	core_nt	Penicillium brevicompactum uncharacterized protein (N7506_001908), partial mRNA	non-Lecanoromycete	704	704	0.74	0	38.3	3576	XM_056951204.1
XANPAOZ2100_002167-T1	core_nt	Talaromyces amestolkiae uncharacterized protein (BHQ10_001440), partial mRNA	non-Lecanoromycete	703	703	0.77	0	37.28	3498	XM_040873477.1
XANPAOZ2100_002167-T1	core_nt	Byssochlamys spectabilis hypothetical protein (C8Q69DRAFT_150824), mRNA	non-Lecanoromycete	702	702	0.66	0	39.72	3604	XM_028625542.1
XANPAOZ2100_002167-T1	core_nt	Emericellopsis atlantica wd-repeat protein (F5Z01DRAFT_621309), partial mRNA	non-Lecanoromycete	692	692	0.75	0	36.79	3630	XM_046261198.1
XANPAOZ2100_002167-T1	core_nt	Fusarium musae uncharacterized protein (J7337_010619), partial mRNA	non-Lecanoromycete	676	676	0.73	0	36.82	3441	XM_044828194.1
XANPAOZ2100_002167-T1	core_nt	Penicillium diatomitis uncharacterized protein	non-Lecanoromycete	672	672	0.7	0	37.5	3501	XM_056938945.1

		(N7539_009350), partial mRNA								
XANPAOZ2100_002167-T1	core_nt	Geosmithia morbida NACHT domain (GMORB2_5051), partial mRNA	non-Lecanoromycete	670	670	0.65	0	39.88	2913	XM_035467025.1
XANPAOZ2100_002167-T1	core_nt	Emericellopsis cladophorae Vegetative incompatibility protein HET-E-like protein (J7T54_002603), partial mRNA	non-Lecanoromycete	667	667	0.72	0	37.43	3408	XM_051509131.1
XANPAOZ2100_002167-T1	core_nt	Penicillium lagena uncharacterized protein (N7510_005905), partial mRNA	non-Lecanoromycete	662	662	0.66	0	39.37	3213	XM_056977831.1
XANPAOZ2100_002167-T1	core_nt	Penicillium atrosanguineum Protein of unknown function DUF3468 (N7443_010211), partial mRNA	non-Lecanoromycete	660	660	0.75	0	36.5	3753	XM_056890267.1
XANPAOZ2100_002167-T1	core_nt	Aspergillus karnatakaensis uncharacterized protein (BJY04DRAFT_218660), partial mRNA	non-Lecanoromycete	653	653	0.74	0	36.19	3429	XM_073716366.1
XANPAOZ2100_002167-T1	core_nt	Aspergillus welwitschiae uncharacterized protein (BDQ94DRAFT_139360), mRNA	non-Lecanoromycete	650	650	0.62	0	39.83	3331	XM_026765410.1
XANPAOZ2100_002167-T1	core_nt	Aspergillus vadensis CBS 113365 uncharacterized protein (BO88DRAFT_440435), mRNA	non-Lecanoromycete	647	647	0.62	0	39.83	3318	XM_025709785.1
XANPAOZ2100_002167-T1	core_nt	Alternaria dauci uncharacterized protein (ACET3X_006422), partial mRNA	non-Lecanoromycete	647	647	0.66	0	38.13	3247	XM_069453077.1
XANPAOZ2100_002167-T1	core_nt	Parastagonospora nodorum strain Sn79 chromosome 3	non-Lecanoromycete	637	637	0.73	0	35.32	2251062	CP039671.1
XANPAOZ2100_002167-T1	core_nt	Bipolaris sorokiniana ND90Pr uncharacterized protein (COCSADRAFT_34948), mRNA	non-Lecanoromycete	636	636	0.7	0	37.14	3611	XM_007699348.1

XANPAOZ2100_002167-T1	core_nt	Exophiala xenobiotica uncharacterized protein (PV05_08003), partial mRNA	non-Lecanoromycete	633	633	0.42	0	53.14	1881	XM_013457491.1
XANPAOZ2100_002167-T1	core_nt	Talaromyces proteolyticus uncharacterized protein (BGW36DRAFT_429550), partial mRNA	non-Lecanoromycete	630	630	0.61	0	39.78	3048	XM_046220711.1
XANPAOZ2100_002167-T1	core_nt	Cordyceps militaris CM01 wd-repeat protein (CCM_00812), partial mRNA	non-Lecanoromycete	624	624	0.74	0	35.23	3582	XM_006665971.1
XANPAOZ2100_002167-T1	core_nt	Penicillium verhagenii uncharacterized protein (N7466_003935), partial mRNA	non-Lecanoromycete	622	622	0.69	0	36.92	3264	XM_057163332.1
XANPAOZ2100_002167-T1	core_nt	Penicillium cinerascens uncharacterized protein (N7498_004301), partial mRNA	non-Lecanoromycete	618	618	0.64	0	39.25	3057	XM_058451363.1
XANPAOZ2100_002167-T1	core_nt	Microdochium trichocladiopsis uncharacterized protein (B0I36DRAFT_265433), mRNA	non-Lecanoromycete	612	612	0.72	0	35.48	3561	XM_046151198.1
XANPAOZ2100_002167-T1	core_nt	Dichotomopilus funicola uncharacterized protein (C8A04DRAFT_32311), partial mRNA	non-Lecanoromycete	605	605	0.63	0	38.93	3102	XM_062782051.1
XANPAOZ2100_002167-T1	core_nt	Apiospora marii NACHT domain (PG985_002975), partial mRNA	non-Lecanoromycete	601	601	0.75	0	33.73	3825	XM_066822744.1
XANPAOZ2100_002167-T1	core_nt	Thyridium curvatum uncharacterized protein (E0L32_004870), partial mRNA	non-Lecanoromycete	596	596	0.66	0	34.97	3012	XM_031139328.1
XANPAOZ2100_002167-T1	core_nt	Cladophialophora bantiana CBS 173.52 uncharacterized protein (Z519_09352), partial mRNA	non-Lecanoromycete	590	590	0.34	0	60.04	1422	XM_016767075.1
XANPAOZ2100_002167-T1	core_nt	Apiospora phragmitis NACHT domain (PG994_006603), partial mRNA	non-Lecanoromycete	590	590	0.71	0	34.08	3645	XM_066858012.1

XANPAOZ2100_002167-T1	core_nt	Scedosporium apiospermum NACHT nucleoside triphosphatase (SAPIO_CDS1428), partial mRNA	non-Lecanoromycete	588	588	0.63	0	36.74	3159	XM_016784704.1
XANPAOZ2100_002167-T1	core_nt	Ramularia collo-cygni uncharacterized protein (RCC_06734), partial mRNA	non-Lecanoromycete	587	587	0.62	0	37.01	3180	XM_023771995.1
XANPAOZ2100_002167-T1	core_nt	Isaria fumosorosea ARSEF 2679 NACHT domain protein (ISF_04581), partial mRNA	non-Lecanoromycete	585	585	0.68	0	34.91	3357	XM_018848187.1
XANPAOZ2100_002167-T1	core_nt	Hypoxylon trugodes uncharacterized protein (F4822DRAFT_445871), partial mRNA	non-Lecanoromycete	585	649	0.69	0	38.12	3091	XM_051463437.1
XANPAOZ2100_002167-T1	core_nt	Penicillium vulpinum uncharacterized protein (N7479_007329), partial mRNA	non-Lecanoromycete	583	583	0.58	0	37.36	2805	XM_057255023.1
XANPAOZ2100_002167-T1	core_nt	Aspergillus piperis CBS 112811 NACHT domain protein (BO85DRAFT_452962), mRNA	non-Lecanoromycete	577	577	0.55	0	39.73	2831	XM_025660956.1
XANPAOZ2100_002167-T1	core_nt	Metarhizium brunneum Transporter mfs1 (mfs1_2), partial mRNA	non-Lecanoromycete	580	580	0.66	3E-177	35.72	4362	XM_066129971.1
XANPAOZ2100_002167-T1	core_nt	Metarhizium robertsii ARSEF 23 NACHT domain protein (MAA_05266), partial mRNA	non-Lecanoromycete	572	572	0.63	5E-177	36.28	3540	XM_007823264.2
XANPAOZ2100_002167-T1	core_nt	Trichoderma atroviride uncharacterized protein (TrAtP1_006182), mRNA	non-Lecanoromycete	549	549	0.75	4E-166	32.02	4154	XM_014082819.2
XANPAOZ2100_002167-T1	core_nt	Penicillium coprophilum uncharacterized protein (N7500_006598), partial mRNA	non-Lecanoromycete	528	528	0.53	4E-165	38.25	2355	XM_056677742.1
XANPAOZ2100_002167-T1	core_nt	Trichoderma citrinoviride hypothetical protein (BBK36DRAFT_146201), partial mRNA	non-Lecanoromycete	527	527	0.75	9E-160	32.21	3678	XM_024897535.1

XANPAOZ2100_002167-T1	core_nt	Trichoderma ghanense uncharacterized protein (CCMA1212_006451), partial mRNA	non-Lecanoromycete	526	526	0.75	3E-159	32.51	3618	XM_073703672.1
XANPAOZ2100_002167-T1	core_nt	Talaromyces marneffei uncharacterized protein (EYB26_007038), partial mRNA	non-Lecanoromycete	539	539	0.63	4E-159	36.61	5484	XM_054266310.1
XANPAOZ2100_002167-T1	core_nt	Fusarium vanettenii 77-13-4 uncharacterized protein (NECHADRAFT_97420), partial mRNA	non-Lecanoromycete	500	500	0.48	2E-155	37.95	2121	XM_003041991.1
XANPAOZ2100_002167-T1	core_nt	Aspergillus novofumigatus IBT 16806 NACHT domain protein (P174DRAFT_464308), partial mRNA	non-Lecanoromycete	495	495	0.49	2E-153	37.57	2226	XM_024830374.1
XANPAOZ2100_002167-T1	core_nt	Aspergillus fumigatus CEA10 chromosome 4	non-Lecanoromycete	529	610	0.58	2E-152	37.96	3820116	CP097566.1
XANPAOZ2100_002167-T1	core_nt	Trichoderma reesei QM6a uncharacterized protein (TRIREDRAFT_109341), partial mRNA	non-Lecanoromycete	505	505	0.75	2E-151	32.13	3633	XM_006967044.1
XANPAOZ2100_002167-T1	core_nt	Thermothielavioides terrestris NRRL 8126 uncharacterized protein (THITE_2037841), partial mRNA	non-Lecanoromycete	488	488	0.46	6E-151	39.94	2127	XM_003649575.1
XANPAOZ2100_002167-T1	core_nt	Aspergillus steynii IBT 23096 uncharacterized protein (P170DRAFT_443267), partial mRNA	non-Lecanoromycete	484	484	0.61	2E-148	34.68	2430	XM_024850524.1
XANPAOZ2100_002167-T1	core_nt	Madurella fahalii uncharacterized protein (MFIFM68171_03680), partial mRNA	non-Lecanoromycete	477	477	0.74	8E-142	30.84	3438	XM_071059101.1
XANPAOZ2100_002167-T1	core_nt	Bipolaris maydis clone FNFP21-O24, complete sequence	non-Lecanoromycete	494	494	0.63	9E-141	34.29	32167	AC277308.1
XANPAOZ2100_002167-T1	core_nt	Bipolaris maydis clone FNFP197-D01, complete sequence	non-Lecanoromycete	494	494	0.63	9E-141	34.29	37257	AC277180.1

XANPAOZ2100_002167-T1	core_nt	Bipolaris maydis clone FNFP161-D18, complete sequence	non-Lecanoromycete	494	494	0.63	9E-141	34.29	41372	AC277363.1
XANPAOZ2100_002167-T1	core_nt	Aspergillus fumigatus CEA10 chromosome 3	non-Lecanoromycete	479	613	0.58	3E-139	38.14	4270437	CP097565.1
XANPAOZ2100_002167-T1	core_nt	Penicillium daleae uncharacterized protein (N7458_007455), partial mRNA	non-Lecanoromycete	450	450	0.42	9E-138	40.48	1908	XM_056910837.1
XANPAOZ2100_002167-T1	core_nt	Fusarium graminearum PH-1 uncharacterized protein (FGSG_10428), partial mRNA	non-Lecanoromycete	443	443	0.49	4E-134	35.92	2139	XM_011321101.1
XANPAOZ2100_002167-T1	core_nt	Penicillium brevicompactum uncharacterized protein (N7506_012228), partial mRNA	non-Lecanoromycete	445	445	0.51	4E-134	36.89	2331	XM_056961519.1
XANPAOZ2100_002167-T1	core_nt	Alternaria rosae uncharacterized protein (BKA58DRAFT_442933), partial mRNA	non-Lecanoromycete	456	456	0.75	5E-134	29.72	3513	XM_046173942.1
XANPAOZ2100_002167-T1	core_nt	Didymella exigua CBS 183.55 uncharacterized protein (M421DRAFT_403724), partial mRNA	non-Lecanoromycete	448	448	0.75	3E-131	28.92	3459	XM_033590267.1
XANPAOZ2100_002167-T1	core_nt	Verticillium nonalfalfae uncharacterized protein (D7B24_005221), partial mRNA	non-Lecanoromycete	449	449	0.77	4E-131	29.29	3606	XM_028639380.1
XANPAOZ2100_002167-T1	core_nt	Cercospora kikuchii uncharacterized protein (CKM354_000780200), partial mRNA	non-Lecanoromycete	434	434	0.56	9E-130	34.19	2466	XM_044803160.1
XANPAOZ2100_002167-T1	core_nt	Parastagonospora nodorum SN15 hypothetical protein (SNOG_05529), partial mRNA	non-Lecanoromycete	427	427	0.47	5E-129	37.9	1995	XM_001795882.1
XANPAOZ2100_002167-T1	core_nt	Chaetomium strumarium uncharacterized protein	non-Lecanoromycete	443	443	0.75	8E-129	30.04	3702	XM_062866645.1

		(B0T15DRAFT_490017), partial mRNA								
XANPAOZ2100_002167-T1	core_nt	Chaetomium fimeti uncharacterized protein (B0H64DRAFT_80083), mRNA	non-Lecanoromycete	443	443	0.73	4E-128	30.26	3964	XM_062808787.1
XANPAOZ2100_002167-T1	core_nt	Thermothielavioides terrestris NRRL 8126 uncharacterized protein (THITE_2149928), partial mRNA	non-Lecanoromycete	437	437	0.75	1E-126	31.79	3660	XM_003651804.1
XANPAOZ2100_002167-T1	core_nt	Cercospora beticola uncharacterized protein (RHO25_007085), partial mRNA	non-Lecanoromycete	420	420	0.55	3E-124	33.21	2490	XM_023597864.2
XANPAOZ2100_002167-T1	core_nt	Exophiala xenobiotica uncharacterized protein (PV05_08004), partial mRNA	non-Lecanoromycete	408	408	0.3	6E-124	49.89	1566	XM_013457492.1
XANPAOZ2100_002167-T1	core_nt	Wardomyces moseri uncharacterized protein (JN550_007857), partial mRNA	non-Lecanoromycete	422	592	0.68	1E-122	35.11	3183	XM_049319753.1
XANPAOZ2100_002167-T1	core_nt	Trichoderma atroviride strain CBMAI-0020 clone BAC ta20_023 genomic sequence, complete sequence	non-Lecanoromycete	439	439	0.59	2E-122	32.99	112507	OR643402.1
XANPAOZ2100_002167-T1	core_nt	Cladophialophora carrionii CBS 160.54 uncharacterized protein (G647_10274), partial mRNA	non-Lecanoromycete	412	412	0.55	4E-122	33.58	2292	XM_008726265.1
XANPAOZ2100_002167-T1	core_nt	Fusarium pseudograminearum CS3096 uncharacterized protein (FPSE_07444), partial mRNA	non-Lecanoromycete	411	411	0.42	2E-120	40.06	2649	XM_009260562.1
XANPAOZ2100_002167-T1	core_nt	Boeremia exigua uncharacterized protein (C7974DRAFT_464511), partial mRNA	non-Lecanoromycete	399	399	0.47	8E-119	35.58	1975	XM_046146266.1
XANPAOZ2100_002167-T1	core_nt	Aspergillus glaucus CBS 516.65 uncharacterized protein	non-Lecanoromycete	385	385	0.45	9E-114	35.49	1937	XM_022549907.1

		(ASPGLDRAFT_77654), partial mRNA								
XANPAOZ2100_002167-T1	core_nt	Trichoderma aggressivum f. europaeum uncharacterized protein (Triagg1_10804), partial mRNA	non-Lecanoromycete	394	491	0.68	2E-112	32.6	3222	XM_062894916.1
XANPAOZ2100_002167-T1	core_nt	Aspergillus sydowii CBS 593.65 uncharacterized protein (ASPSYDRAFT_91102), partial mRNA	non-Lecanoromycete	379	379	0.38	5E-112	39.86	1854	XM_040852434.1
XANPAOZ2100_002167-T1	core_nt	Pochonia chlamydosporia 170 NACHT domain-containing protein (VFPPC_06580), partial mRNA	non-Lecanoromycete	367	367	0.43	4E-107	34.58	1944	XM_018285591.1
XANPAOZ2100_002167-T1	core_nt	Isaria fumosorosea ARSEF 2679 Ankyrin repeat-containing domain protein (ISF_05692), partial mRNA	non-Lecanoromycete	330	330	0.7	5E-89	24.34	3714	XM_018849297.1
XANPAOZ2100_002167-T1	core_nt	Akanthomyces muscarius uncharacterized protein (LMH87_002791), partial mRNA	non-Lecanoromycete	324	324	0.75	1E-87	26.3	3438	XM_056194305.1
XANPAOZ2100_002167-T1	core_nt	Zymoseptoria tritici IPO323 uncharacterized protein (MYCGRDRAFT_50673), partial mRNA	non-Lecanoromycete	310	310	0.42	3E-87	32.75	1821	XM_003847902.1
XANPAOZ2100_002167-T1	core_nt	Lasiosphaeria miniovina uncharacterized protein (B0T26DRAFT_803888), partial mRNA	non-Lecanoromycete	310	395	0.39	3E-87	39.13	1804	XM_060447107.1
XANPAOZ2100_002167-T1	core_nt	Zymoseptoria tritici isolate M4_8 chromosome 6_12.03	non-Lecanoromycete	312	312	0.41	2E-81	33.01	2980394	CP062565.1
XANPAOZ2100_002167-T1	core_nt	Penicillium subrubescens uncharacterized protein (N7473_004961), partial mRNA	non-Lecanoromycete	296	409	0.52	1E-80	33.21	2334	XM_057150901.1
XANPAOZ2100_002166-T1	nt/nr limited to Lecanoromycetes	Flavoplaca marina genome assembly, chromosome: 1	Teloschistales (Lecanoromycete)	400	400	1	2E-127	74.81	3868129	OZ245337.1

XANPAOZ2100_002166-T1	nt/nr limited to Lecanoromycetes	Xanthoria parietina genome assembly, chromosome: 3	Teloschistales (Lecanoromycete)	329	471	1	1E-125	90.61	2273945	OZ210030.1
XANPAOZ2100_002166-T1	nt/nr limited to Lecanoromycetes	Letharia columbiana uncharacterized protein (HO173_000994), partial mRNA	non-Teloschistales Lecanoromycete	165	165	0.89	8E-50	43.41	705	XM_037302941.1
XANPAOZ2100_002166-T1	nt/nr limited to Lecanoromycetes	Letharia lupina uncharacterized protein (HO133_005206), partial mRNA	non-Teloschistales Lecanoromycete	164	164	0.89	1E-49	43.41	705	XM_037296117.1
XANPAOZ2100_002166-T1	nt/nr limited to Lecanoromycetes	Ramalina siliquosa genome assembly, chromosome: 7	non-Teloschistales Lecanoromycete	164	203	0.96	3E-45	45.26	2028249	OZ244603.1
XANPAOZ2100_002166-T1	nt/nr limited to Lecanoromycetes	Cladonia portentosa genome assembly, chromosome: 1	non-Teloschistales Lecanoromycete	95.9	156	0.7	2E-21	50	2177238	OZ232336.1
XANPAOZ2100_002167-T1	nt/nr limited to Lecanoromycetes	Xanthoria parietina genome assembly, chromosome: 3	Teloschistales (Lecanoromycete)	1743	2900	1	0	94.33	2273945	OZ210030.1
XANPAOZ2100_002167-T1	nt/nr limited to Lecanoromycetes	Flavoplaca marina genome assembly, chromosome: 1	Teloschistales (Lecanoromycete)	1451	1883	0.79	0	79.09	3868129	OZ245337.1
XANPAOZ2100_002167-T1	nt/nr limited to Lecanoromycetes	Platismatia glauca genome assembly, chromosome: 5	non-Teloschistales Lecanoromycete	718	860	0.74	0	40.83	1872535	OY745767.1
XANPAOZ2100_002167-T1	nt/nr limited to Lecanoromycetes	Platismatia glauca genome assembly, chromosome: 4	non-Teloschistales Lecanoromycete	541	723	0.59	8E-160	43.48	2095241	OY745766.1
XANPAOZ2100_002167-T1	nt/nr limited to Lecanoromycetes	Cladonia norvegica genome assembly, chromosome: 19	non-Teloschistales Lecanoromycete	89.4	138	0.28	2E-16	21.93	1115434	OZ020218.1
XANPAOZ2100_002167-T1	nt/nr limited to Lecanoromycetes	Letharia lupina uncharacterized protein (HO133_010583), partial mRNA	non-Teloschistales Lecanoromycete	87.8	137	0.34	5E-16	25.78	4236	XM_037301435.1
XANPAOZ2100_002167-T1	nt/nr limited to Lecanoromycetes	Letharia lupina uncharacterized protein (HO133_008519), partial mRNA	non-Teloschistales Lecanoromycete	87.4	87.4	0.3	7E-16	23.05	3942	XM_037299385.1
XANPAOZ2100_002167-T1	nt/nr limited to Lecanoromycetes	Umbilicaria deusta genome assembly, chromosome: 11	non-Teloschistales Lecanoromycete	87	87	0.32	1E-15	25.15	1688060	OZ203553.1
XANPAOZ2100_002167-T1	nt/nr limited to Lecanoromycetes	Letharia columbiana uncharacterized protein (HO173_003772), partial mRNA	non-Teloschistales Lecanoromycete	84.3	84.3	0.21	2E-15	26.13	1284	XM_037305697.1
XANPAOZ2100_002167-T1	nt/nr limited to Lecanoromycetes	Cladonia squamosa genome assembly, chromosome: 21	non-Teloschistales Lecanoromycete	86.3	86.3	0.27	2E-15	22.61	1102328	OX392392.1

XANPAOZ2100_002167-T1	nt/nr limited to Lecanoromycetes	Letharia columbiana uncharacterized protein (HO173_009837), partial mRNA	non-Teloschistales Lecanoromycete	85.1	85.1	0.28	2E-15	22.39	1932	XM_037311725.1
XANPAOZ2100_002167-T1	nt/nr limited to Lecanoromycetes	Bacidia gigantensis uncharacterized protein (KY384_003575), partial mRNA	non-Teloschistales Lecanoromycete	85.5	85.5	0.2	2E-15	24.56	4158	XM_044812089.1
XANPAOZ2100_002167-T1	nt/nr limited to Lecanoromycetes	Flavoplaca marina genome assembly, chromosome: 15	Teloschistales (Lecanoromycete)	85.9	85.9	0.28	3E-15	22.9	1338266	OZ245351.1
XANPAOZ2100_002167-T1	nt/nr limited to Lecanoromycetes	Cladonia diversa genome assembly, chromosome: 21	non-Teloschistales Lecanoromycete	64.7	105	0.26	3E-15	25.36	1008410	OZ208693.1
XANPAOZ2100_002167-T1	nt/nr limited to Lecanoromycetes	Xanthoria parietina genome assembly, chromosome: 10	Teloschistales (Lecanoromycete)	80.5	80.5	0.21	1E-13	24.54	1600061	OZ210037.1
XANPAOZ2100_002167-T1	nt/nr limited to Lecanoromycetes	Flavoplaca marina genome assembly, chromosome: 2	Teloschistales (Lecanoromycete)	79.7	134	0.34	2E-13	23.58	3435219	OZ245338.1
XANPAOZ2100_002167-T1	nt/nr limited to Lecanoromycetes	Platismatia glauca genome assembly, chromosome: 1	non-Teloschistales Lecanoromycete	79	79	0.29	4E-13	21.38	2237538	OY745763.1
XANPAOZ2100_002167-T1	nt/nr limited to Lecanoromycetes	Cladonia diversa genome assembly, chromosome: 8	non-Teloschistales Lecanoromycete	76.6	76.6	0.28	2E-12	23.61	1729717	OZ208680.1
XANPAOZ2100_002167-T1	nt/nr limited to Lecanoromycetes	Bacidia gigantensis uncharacterized protein (KY384_008400), partial mRNA	non-Teloschistales Lecanoromycete	75.5	75.5	0.35	2E-12	21.32	3648	XM_044816889.1
XANPAOZ2100_002167-T1	nt/nr limited to Lecanoromycetes	Platismatia glauca genome assembly, chromosome: 7	non-Teloschistales Lecanoromycete	75.1	75.1	0.25	5E-12	21.37	1844907	OY745769.1
XANPAOZ2100_002167-T1	nt/nr limited to Lecanoromycetes	Xanthoria parietina genome assembly, chromosome: 2	Teloschistales (Lecanoromycete)	74.3	119	0.3	8E-12	23.58	2742845	OZ210029.1
XANPAOZ2100_002167-T1	nt/nr limited to Lecanoromycetes	Platismatia glauca genome assembly, chromosome: 2	non-Teloschistales Lecanoromycete	70.9	137	0.28	9E-11	24.94	2216080	OY745764.1
XANPAOZ2100_002167-T1	nt/nr limited to Lecanoromycetes	Cladonia diversa genome assembly, chromosome: 23	non-Teloschistales Lecanoromycete	69.3	69.3	0.2	3E-10	26.73	508859	OZ208695.1
XANPAOZ2100_002167-T1	nt/nr limited to Lecanoromycetes	Cladonia squamosa genome assembly, chromosome: 3	non-Teloschistales Lecanoromycete	68.9	68.9	0.28	4E-10	21.06	2074597	OX392374.1
XANPAOZ2100_002167-T1	nt/nr limited to Lecanoromycetes	Cladonia portentosa genome assembly, chromosome: 6	non-Teloschistales Lecanoromycete	68.6	68.6	0.28	5E-10	21.41	1766384	OZ232341.1
XANPAOZ2100_002167-T1	nt/nr limited to Lecanoromycetes	Cladonia squamosa genome assembly, chromosome: 8	non-Teloschistales Lecanoromycete	68.6	111	0.27	5E-10	22.89	1658534	OX392379.1
XANPAOZ2100_002167-T1	nt/nr limited to Lecanoromycetes	Bacidia gigantensis uncharacterized protein	non-Teloschistales Lecanoromycete	65.5	65.5	0.29	3E-9	22.98	2694	XM_044809735.1

		(KY384_001203), partial mRNA								
XANPAOZ2100_002167-T1	nt/nr limited to Lecanoromycetes	Letharia lupina uncharacterized protein (HO133_010247), partial mRNA	non-Teloschistales Lecanoromycete	65.1	65.1	0.16	4E-9	26.05	3909	XM_037301102.1
XANPAOZ2100_002167-T1	nt/nr limited to Lecanoromycetes	Ramalina siliquosa genome assembly, chromosome: 13	non-Teloschistales Lecanoromycete	65.1	65.1	0.23	6E-9	23.12	1832005	OZ244609.1
XANPAOZ2100_002167-T1	nt/nr limited to Lecanoromycetes	Bacidia gigantensis uncharacterized protein (KY384_000134), partial mRNA	non-Teloschistales Lecanoromycete	64.3	64.3	0.25	6E-9	22.42	2904	XM_044808671.1
XANPAOZ2100_002167-T1	nt/nr limited to Lecanoromycetes	Cladonia squamosa genome assembly, chromosome: 26	non-Teloschistales Lecanoromycete	64.7	64.7	0.28	7E-9	23.61	465332	OX392397.1
XANPAOZ2100_002167-T1	nt/nr limited to Lecanoromycetes	Bacidia gigantensis uncharacterized protein (KY384_000400), partial mRNA	non-Teloschistales Lecanoromycete	63.9	63.9	0.19	9E-9	22.65	3042	XM_044808936.1
XANPAOZ2100_002167-T1	nt/nr limited to Lecanoromycetes	Bacidia gigantensis uncharacterized protein (KY384_008049), partial mRNA	non-Teloschistales Lecanoromycete	62.8	62.8	0.25	2E-8	23.54	2547	XM_044816538.1
XANPAOZ2100_002167-T1	nt/nr limited to Lecanoromycetes	Letharia lupina uncharacterized protein (HO133_002611), partial mRNA	non-Teloschistales Lecanoromycete	62	62	0.22	4E-8	24.7	5097	XM_037293536.1
XANPAOZ2100_002167-T1	nt/nr limited to Lecanoromycetes	Letharia lupina uncharacterized protein (HO133_002473), partial mRNA	non-Teloschistales Lecanoromycete	61.6	61.6	0.26	4E-8	22.76	2949	XM_037293399.1
XANPAOZ2100_002167-T1	nt/nr limited to Lecanoromycetes	Ramalina siliquosa genome assembly, chromosome: 11	non-Teloschistales Lecanoromycete	61.2	61.2	0.28	8E-8	23.46	1889834	OZ244607.1
XANPAOZ2100_002167-T1	nt/nr limited to Lecanoromycetes	Cladonia norvegica genome assembly, chromosome: 16	non-Teloschistales Lecanoromycete	60.8	60.8	0.26	1E-7	23.65	1243380	OZ020215.1
XANPAOZ2100_002167-T1	nt/nr limited to Lecanoromycetes	Bacidia gigantensis uncharacterized protein (KY384_001241), partial mRNA	non-Teloschistales Lecanoromycete	60.1	60.1	0.21	1E-7	24.61	3408	XM_044809772.1
XANPAOZ2100_002167-T1	nt/nr limited to Lecanoromycetes	Umbilicaria deusta genome assembly, chromosome: 6	non-Teloschistales Lecanoromycete	60.1	60.1	0.28	2E-7	19.08	1917118	OZ203548.1

XANPAOZ2100_002167-T1	nt/nr limited to Lecanoromycetes	Letharia lupina uncharacterized protein (HO133_007644), partial mRNA	non-Teloschistales Lecanoromycete	59.3	59.3	0.22	2E-7	20.71	5001	XM_037298514.1
XANPAOZ2100_002167-T1	nt/nr limited to Lecanoromycetes	Bacidia gigantensis uncharacterized protein (KY384_008222), partial mRNA	non-Teloschistales Lecanoromycete	59.3	59.3	0.25	3E-7	22.19	3996	XM_044816711.1
XANPAOZ2100_002167-T1	nt/nr limited to Lecanoromycetes	Bacidia gigantensis uncharacterized protein (KY384_001774), partial mRNA	non-Teloschistales Lecanoromycete	58.5	58.5	0.22	4E-7	21.87	3834	XM_044810301.1
XANPAOZ2100_002167-T1	nt/nr limited to Lecanoromycetes	Letharia lupina uncharacterized protein (HO133_006078), partial mRNA	non-Teloschistales Lecanoromycete	58.2	58.2	0.29	5E-7	22.39	4959	XM_037296981.1
XANPAOZ2100_002167-T1	nt/nr limited to Lecanoromycetes	Cladonia squamosa genome assembly, chromosome: 16	non-Teloschistales Lecanoromycete	58.2	58.2	0.26	7E-7	22.85	1302476	OX392387.1
XANPAOZ2100_002167-T1	nt/nr limited to Lecanoromycetes	Bacidia gigantensis uncharacterized protein (KY384_007485), partial mRNA	non-Teloschistales Lecanoromycete	57.4	57.4	0.19	8E-7	21.93	4473	XM_044815976.1
XANPAOZ2100_002167-T1	nt/nr limited to Lecanoromycetes	Cladonia diversa genome assembly, chromosome: 6	non-Teloschistales Lecanoromycete	57	143	0.28	1E-6	22.18	1817240	OZ208678.1
XANPAOZ2100_002167-T1	nt/nr limited to Lecanoromycetes	Cladonia portentosa genome assembly, chromosome: 8	non-Teloschistales Lecanoromycete	56.6	147	0.27	2E-6	20.95	1590851	OZ232343.1
XANPAOZ2100_002167-T1	nt/nr limited to Lecanoromycetes	Ramalina siliquosa genome assembly, chromosome: 7	non-Teloschistales Lecanoromycete	56.6	56.6	0.38	2E-6	21.05	2028249	OZ244603.1
XANPAOZ2100_002167-T1	nt/nr limited to Lecanoromycetes	Cladonia portentosa genome assembly, chromosome: 15	non-Teloschistales Lecanoromycete	56.2	56.2	0.28	3E-6	20.24	1391848	OZ232350.1
XANPAOZ2100_002167-T1	nt/nr limited to Lecanoromycetes	Letharia columbiana uncharacterized protein (HO173_005047), partial mRNA	non-Teloschistales Lecanoromycete	55.8	55.8	0.19	3E-6	23.87	6495	XM_037306965.1
XANPAOZ2100_002167-T1	nt/nr limited to Lecanoromycetes	Letharia columbiana uncharacterized protein (HO173_005442), partial mRNA	non-Teloschistales Lecanoromycete	55.1	55.1	0.29	4E-6	21.76	1758	XM_037307359.1
XANPAOZ2100_002167-T1	nt/nr limited to Lecanoromycetes	Bacidia gigantensis uncharacterized protein	non-Teloschistales Lecanoromycete	55.5	55.5	0.28	4E-6	22.57	4638	XM_044810951.1

		(KY384_002428), partial mRNA								
XANPAOZ2100_002167-T1	nt/nr limited to Lecanoromycetes	Cladonia uncialis subsp. uncialis clone SPAdes15 genomic sequence	non-Teloschistales Lecanoromycete	55.5	55.5	0.24	5E-6	21.63	106278	MG777483.1
XANPAOZ2100_002167-T1	nt/nr limited to Lecanoromycetes	Letharia columbiana uncharacterized protein (HO173_010921), partial mRNA	non-Teloschistales Lecanoromycete	54.7	54.7	0.22	5E-6	22.22	2403	XM_037312806.1
XANPAOZ2100_002167-T1	nt/nr limited to Lecanoromycetes	Cladonia squamosa genome assembly, chromosome: 6	non-Teloschistales Lecanoromycete	54.3	100	0.21	1E-5	21.82	1732432	OX392377.1
XANPAOZ2100_002167-T1	nt/nr limited to Lecanoromycetes	Letharia lupina uncharacterized protein (HO133_005708), partial mRNA	non-Teloschistales Lecanoromycete	53.5	53.5	0.17	1E-5	18.96	3714	XM_037296615.1
XANPAOZ2100_002167-T1	nt/nr limited to Lecanoromycetes	Ramalina siliquosa genome assembly, chromosome: 1	non-Teloschistales Lecanoromycete	53.5	106	0.21	2E-5	27.75	2850942	OZ244597.1
XANPAOZ2100_002167-T1	nt/nr limited to Lecanoromycetes	Cladonia norvegica genome assembly, chromosome: 9	non-Teloschistales Lecanoromycete	53.1	150	0.27	2E-5	22.81	1590203	OZ020208.1
XANPAOZ2100_002167-T1	nt/nr limited to Lecanoromycetes	Cladonia squamosa genome assembly, chromosome: 24	non-Teloschistales Lecanoromycete	52.4	97	0.15	4E-5	24.02	589747	OX392395.1
XANPAOZ2100_002167-T1	nt/nr limited to Lecanoromycetes	Cladonia squamosa genome assembly, chromosome: 18	non-Teloschistales Lecanoromycete	52	145	0.33	5E-5	20.79	1210831	OX392389.1
XANPAOZ2100_002167-T1	nt/nr limited to Lecanoromycetes	Xanthoria parietina genome assembly, chromosome: 16	Teloschistales (Lecanoromycete)	51.6	51.6	0.12	7E-5	27.6	1172400	OZ210043.1
XANPAOZ2100_002167-T1	nt/nr limited to Lecanoromycetes	Cladonia diversa genome assembly, chromosome: 16	non-Teloschistales Lecanoromycete	50.8	92.8	0.29	9E-5	24.56	1433162	OZ208688.1
XANPAOZ2100_002167-T1	nt/nr limited to Lecanoromycetes	Letharia columbiana uncharacterized protein (HO173_007601), partial mRNA	non-Teloschistales Lecanoromycete	50.1	50.1	0.13	0.0001	22.06	1521	XM_037309503.1
XANPAOZ2100_002167-T1	nt/nr limited to Lecanoromycetes	Letharia columbiana uncharacterized protein (HO173_012760), partial mRNA	non-Teloschistales Lecanoromycete	49.7	49.7	0.14	0.0001	24.42	1479	XM_037314594.1
XANPAOZ2100_002167-T1	nt/nr limited to Lecanoromycetes	Cladonia norvegica genome assembly, chromosome: 1	non-Teloschistales Lecanoromycete	50.4	96.7	0.27	0.0001	25.67	2477012	OZ020200.1
XANPAOZ2100_002167-T1	nt/nr limited to Lecanoromycetes	Cladonia norvegica genome assembly, chromosome: 4	non-Teloschistales Lecanoromycete	50.1	50.1	0.14	0.0002	23.85	2120184	OZ020203.1

XANPAOZ2100_002167-T1	nt/nr limited to Lecanoromycetes	Cladonia uncialis subsp. uncialis clone DNAstar32 genomic sequence	non-Teloschistales Lecanoromycete	49.7	49.7	0.21	0.0002	23.36	73373	KX264279.1
XANPAOZ2100_002167-T1	nt/nr limited to Lecanoromycetes	Cladonia uncialis subsp. uncialis clone SPAdes3 genomic sequence	non-Teloschistales Lecanoromycete	49.7	49.7	0.21	0.0002	23.36	113581	MG777471.1
XANPAOZ2100_002167-T1	nt/nr limited to Lecanoromycetes	Cladonia portentosa genome assembly, chromosome: 25	non-Teloschistales Lecanoromycete	49.7	49.7	0.12	0.0003	23.66	486778	OZ232360.1
XANPAOZ2100_002167-T1	nt/nr limited to Lecanoromycetes	Letharia columbiana uncharacterized protein (HO173_008429), partial mRNA	non-Teloschistales Lecanoromycete	48.1	48.1	0.17	0.0003	24.9	990	XM_037310329.1
XANPAOZ2100_002167-T1	nt/nr limited to Lecanoromycetes	Cladonia diversa genome assembly, chromosome: 3	non-Teloschistales Lecanoromycete	49.3	49.3	0.12	0.0003	21.43	2064425	OZ208675.1
XANPAOZ2100_002167-T1	nt/nr limited to Lecanoromycetes	Bacidia gigantensis uncharacterized protein (KY384_002258), partial mRNA	non-Teloschistales Lecanoromycete	48.9	48.9	0.24	0.0003	23.24	4167	XM_044810784.1
XANPAOZ2100_002167-T1	nt/nr limited to Lecanoromycetes	Cladonia squamosa genome assembly, chromosome: 20	non-Teloschistales Lecanoromycete	48.9	48.9	0.1	0.0005	29.56	1117100	OX392391.1
XANPAOZ2100_002167-T1	nt/nr limited to Lecanoromycetes	Letharia lupina uncharacterized protein (HO133_002603), partial mRNA	non-Teloschistales Lecanoromycete	48.5	48.5	0.17	0.0005	22.91	6093	XM_037293529.1
XANPAOZ2100_002167-T1	nt/nr limited to Lecanoromycetes	Cladonia diversa genome assembly, chromosome: 20	non-Teloschistales Lecanoromycete	48.5	48.5	0.11	0.0005	25.29	1120590	OZ208692.1
XANPAOZ2100_002167-T1	nt/nr limited to Lecanoromycetes	Cladonia norvegica genome assembly, chromosome: 17	non-Teloschistales Lecanoromycete	48.5	48.5	0.1	0.0005	29.75	1237962	OZ020216.1
XANPAOZ2100_002167-T1	nt/nr limited to Lecanoromycetes	Cladonia portentosa genome assembly, chromosome: 19	non-Teloschistales Lecanoromycete	48.5	48.5	0.03	0.0005	48.94	1134964	OZ232354.1
XANPAOZ2100_002167-T1	nt/nr limited to Lecanoromycetes	Flavoplaca marina genome assembly, chromosome: 14	Teloschistales (Lecanoromycete)	48.5	48.5	0.28	0.0006	22.49	1440631	OZ245350.1
XANPAOZ2100_002167-T1	nt/nr limited to Lecanoromycetes	Letharia columbiana uncharacterized protein (HO173_003771), partial mRNA	non-Teloschistales Lecanoromycete	47.8	47.8	0.09	0.0006	32.14	2295	XM_037305696.1
XANPAOZ2100_002167-T1	nt/nr limited to Lecanoromycetes	Umbilicaria deusta genome assembly, chromosome: 2	non-Teloschistales Lecanoromycete	48.1	48.1	0.07	0.0006	35.58	2353275	OZ203544.1
XANPAOZ2100_002167-T1	nt/nr limited to Lecanoromycetes	Cladonia squamosa genome assembly, chromosome: 17	non-Teloschistales Lecanoromycete	47.8	90.5	0.31	0.001	25.97	1275953	OX392388.1

XANPAOZ2100_002167-T1	nt/nr limited to Lecanoromycetes	Bacidia gigantensis uncharacterized protein (KY384_008477), partial mRNA	non-Teloschistales Lecanoromycete	47	47	0.31	0.001	17.72	3474	XM_044816966.1
XANPAOZ2100_002167-T1	nt/nr limited to Lecanoromycetes	Cladonia diversa genome assembly, chromosome: 5	non-Teloschistales Lecanoromycete	46.6	46.6	0.18	0.002	20.98	1889133	OZ208677.1
XANPAOZ2100_002167-T1	nt/nr limited to Lecanoromycetes	Cladonia norvegica genome assembly, chromosome: 7	non-Teloschistales Lecanoromycete	46.6	46.6	0.18	0.002	21.53	1646023	OZ020206.1
XANPAOZ2100_002167-T1	nt/nr limited to Lecanoromycetes	Letharia lupina uncharacterized protein (HO133_004309), partial mRNA	non-Teloschistales Lecanoromycete	45.8	45.8	0.13	0.003	27.23	2202	XM_037295227.1
XANPAOZ2100_002167-T1	nt/nr limited to Lecanoromycetes	Cladonia squamosa genome assembly, chromosome: 2	non-Teloschistales Lecanoromycete	46.2	91.6	0.23	0.003	21.59	2120710	OX392373.1
XANPAOZ2100_002167-T1	nt/nr limited to Lecanoromycetes	Cladonia portentosa genome assembly, chromosome: 9	non-Teloschistales Lecanoromycete	45.8	45.8	0.12	0.004	23.78	1560360	OZ232344.1
XANPAOZ2100_002167-T1	nt/nr limited to Lecanoromycetes	Cladonia norvegica genome assembly, chromosome: 18	non-Teloschistales Lecanoromycete	45.8	90.9	0.29	0.004	20.62	1188953	OZ020217.1
XANPAOZ2100_002167-T1	nt/nr limited to Lecanoromycetes	Cladonia portentosa genome assembly, chromosome: 10	non-Teloschistales Lecanoromycete	45.4	45.4	0.19	0.005	22.05	1541362	OZ232345.1
XANPAOZ2100_002167-T1	nt/nr limited to Lecanoromycetes	Letharia lupina uncharacterized protein (HO133_003854), partial mRNA	non-Teloschistales Lecanoromycete	45.1	45.1	0.12	0.005	23.5	4377	XM_037294776.1
XANPAOZ2100_002167-T1	nt/nr limited to Lecanoromycetes	Cladonia portentosa genome assembly, chromosome: 13	non-Teloschistales Lecanoromycete	45.1	45.1	0.13	0.005	21.03	1430520	OZ232348.1
XANPAOZ2100_002167-T1	nt/nr limited to Lecanoromycetes	Cladonia squamosa genome assembly, chromosome: 1	non-Teloschistales Lecanoromycete	45.1	45.1	0.05	0.006	34.78	2218899	OX392372.1
XANPAOZ2100_002167-T1	nt/nr limited to Lecanoromycetes	Platismatia glauca genome assembly, chromosome: 3	non-Teloschistales Lecanoromycete	45.1	45.1	0.1	0.006	23.45	2152978	OY745765.1
XANPAOZ2100_002167-T1	nt/nr limited to Lecanoromycetes	Cladonia diversa genome assembly, chromosome: 18	non-Teloschistales Lecanoromycete	44.7	44.7	0.13	0.008	25.98	1334447	OZ208690.1
XANPAOZ2100_002167-T1	nt/nr limited to Lecanoromycetes	Cladonia norvegica genome assembly, chromosome: 8	non-Teloschistales Lecanoromycete	44.7	44.7	0.05	0.008	30.26	1590646	OZ020207.1
XANPAOZ2100_002167-T1	nt/nr limited to Lecanoromycetes	Cladonia diversa genome assembly, chromosome: 22	non-Teloschistales Lecanoromycete	44.7	44.7	0.21	0.008	22.7	861413	OZ208694.1
XANPAOZ2100_002167-T1	nt/nr limited to Lecanoromycetes	Bacidia gigantensis uncharacterized protein (KY384_000509), partial mRNA	non-Teloschistales Lecanoromycete	44.3	44.3	0.27	0.009	20.63	3426	XM_044809045.1

XANPAOZ2100_002167-T1	nt/nr limited to Lecanoromycetes	Platismatia glauca genome assembly, chromosome: 16	non-Teloschistales Lecanoromycete	43.9	43.9	0.03	0.014	48.89	1246429	OY745778.1
XANPAOZ2100_002167-T1	nt/nr limited to Lecanoromycetes	Bacidia gigantensis uncharacterized protein (KY384_004765), partial mRNA	non-Teloschistales Lecanoromycete	43.5	43.5	0.1	0.016	31.17	2826	XM_044813269.1
XANPAOZ2100_002167-T1	nt/nr limited to Lecanoromycetes	Flavoplaca marina genome assembly, chromosome: 8	Teloschistales (Lecanoromycete)	43.5	43.5	0.13	0.017	21.84	1788174	OZ245344.1

Table S13. Alienness Index (AI) for Tangerine-associated genes, as well as five gene models immediately upstream and downstream of Tangerine, and ten randomly selected gene models. AI was caluclated as ln(best E-value of non-lecanoromycete hit + 1e-200) - ln(best E-value of non-Teloschistales lecanoromycete hit + 1e-200) using search results obtained from BLASTp search against the NCBI nr database.

Gene	Genome	Lowest e-value for Lecanoromycetes (excluding Teloschistales)	Lowest e-value for Teloschistales	Lowest e-value for any organism exclusing Lecanoromycetes	Alienness Index	Gene description
glXanPari20_008745-T1	Xanthoria parietina isolate glXanPari20	1.03E-44	3.96E-80	1.24E-40	-9.3958929	other Starship genes
glXanPari20_008746-T1	Xanthoria parietina isolate glXanPari20	1	0	0	460.517019	NLR
glXanPari20_008747-T1	Xanthoria parietina isolate glXanPari20	1	7.45E-169	5.51E-124	283.813987	Methyltransferase
glXanPari20_008748-T1	Xanthoria parietina isolate glXanPari20	3.32E-95	0	1.19E-94	-1.2765736	other Starship genes
glXanPari20_008749-T1	Xanthoria parietina isolate glXanPari20	8.17E-22	3.94E-133	1.02E-13	-18.6426	other Starship genes
glXanPari20_008750-T1	Xanthoria parietina isolate glXanPari20	1	9.33E-177	1	0	other Starship genes
glXanPari20_008751-T1	Xanthoria parietina isolate glXanPari20	1	3.63E-58	1	0	other Starship genes
glXanPari20_008752-T1	Xanthoria parietina isolate glXanPari20	4.69E-48	2.17E-176	2.25E-30	-40.712029	other Starship genes
XANPAOZ2100_000699-T1	Xanthoria parietina isolate glXanPari1	0	0	1.42E-167	-76.335965	Random genes
XANPAOZ2100_000979-T1	Xanthoria parietina isolate glXanPari1	0	0	0	0	Random genes
XANPAOZ2100_002117-T1	Xanthoria parietina isolate glXanPari1	1	0	1	0	Random genes
XANPAOZ2100_002157-T1	Xanthoria parietina isolate glXanPari1	9.28E-97	4.54E-132	8.24E-89	-18.30182	Flanking upstream
XANPAOZ2100_002158-T1	Xanthoria parietina isolate glXanPari1	5.99E-09	5.26E-131	1	-18.933174	Flanking upstream
XANPAOZ2100_002159-T1	Xanthoria parietina isolate glXanPari1	9.99E-53	0	6.89E-37	-36.469848	Flanking upstream
XANPAOZ2100_002160-T1	Xanthoria parietina isolate glXanPari1	3.48E-122	0	8.93E-102	-46.994086	Flanking upstream
XANPAOZ2100_002161-T1	Xanthoria parietina isolate glXanPari1	1.13E-122	0	2.48E-109	-30.719647	Flanking upstream

XANPAOZ2100_002162-T1	Xanthoria parietina isolate glXanPari1	1.18E-22	2.72E-114	5.57E-13	-22.275146	other Starship genes
XANPAOZ2100_002163-T1	Xanthoria parietina isolate glXanPari1	1	2.03E-178	1	0	other Starship genes
XANPAOZ2100_002164-T1	Xanthoria parietina isolate glXanPari1	3.77E-39	0	9.41E-36	-7.8224532	other Starship genes
XANPAOZ2100_002165-T1	Xanthoria parietina isolate glXanPari1	9E-76	0	3.81E-61	-33.679181	other Starship genes
XANPAOZ2100_002166-T1	Xanthoria parietina isolate glXanPari1	1	7.45E-169	5.51E-124	283.813987	Methyltransferase
XANPAOZ2100_002167-T1	Xanthoria parietina isolate glXanPari1	1	0	0	460.517019	NLR
XANPAOZ2100_002168-T1	Xanthoria parietina isolate glXanPari1	0	0	0	0	Flanking downstream
XANPAOZ2100_002169-T1	Xanthoria parietina isolate glXanPari1	1.34E-157	0	4.35E-156	-3.4800913	Flanking downstream
XANPAOZ2100_002170-T1	Xanthoria parietina isolate glXanPari1	0	0	0	0	Flanking downstream
XANPAOZ2100_002171-T1	Xanthoria parietina isolate glXanPari1	0	0	0	0	Flanking downstream
XANPAOZ2100_002172-T1	Xanthoria parietina isolate glXanPari1	1.61E-172	0	3.3E-169	-7.6254436	Flanking downstream
XANPAOZ2100_003656-T1	Xanthoria parietina isolate glXanPari1	0	0	0	0	Random genes
XANPAOZ2100_004401-T1	Xanthoria parietina isolate glXanPari1	4.64E-98	0	2.69E-74	-54.716869	Random genes
XANPAOZ2100_005914-T1	Xanthoria parietina isolate glXanPari1	0	0	0	0	Random genes
XANPAOZ2100_006094-T1	Xanthoria parietina isolate glXanPari1	0	0	0	0	Random genes
XANPAOZ2100_006140-T1	Xanthoria parietina isolate glXanPari1	1	3.62E-30	1	0	Random genes
XANPAOZ2100_007892-T1	Xanthoria parietina isolate glXanPari1	2.03E-74	1.85E-99	2.01E-72	-4.5952691	Random genes
XANPAOZ2100_008367-T1	Xanthoria parietina isolate glXanPari1	2.82E-134	3.61E-174	1.03E-122	-26.623843	Random genes

Table S14. Genomes used in the orthogroup analysis.

ID	Species	Taxonomy	Source	Protein annotation	Reference
XpEllen	Xanthoria parietina glXanPari20	Ascomycota; Lecanoromycetes; Teloschistales	This paper	This paper	
GTX0491	Xanthoria calcicola GTX0491	Ascomycota; Lecanoromycetes; Teloschistales	This paper	This paper	
glXanPari1	Xanthoria parietina glXanPari1	Ascomycota; Lecanoromycetes; Teloschistales	NCBI	This paper	
GTX0501	Xanthoria parietina GTX0501	Ascomycota; Lecanoromycetes; Teloschistales	NCBI	Tagirdzhanova et al. Curr Biol. 2025	Tagirdzhanova et al. Curr Biol. 2025
SacceM3707	Saccharomyces cerevisiae	Ascomycota; Saccaromycetes; Saccharomycetales	JGI	JGI	Brown et al. Genome Announc. 2013
Ascim1	Ascobolus immersus	Ascomycota; Pezizomycetes; Pezizales	JGI	JGI	Murat et al. Nat Ecol Evol. 2018
Morco1	Morchella importuna	Ascomycota; Pezizomycetes; Pezizales	JGI	JGI	Murat et al. Nat Ecol Evol. 2018
Neucr	Neurospora crassa trp3	Ascomycota; Sordariomycetes; Sordariales	JGI	JGI	Baker et al. Genome Announc. 2015
Podan3	Podospora anserina	Ascomycota; Sordariomycetes; Sordariales	JGI	JGI	Espagne et al. Genome Biol. 2008
Fusgr1	Fusarium graminearum	Ascomycota; Sordariomycetes; Hypocreales	JGI	JGI	Cuomo et al. Science. 2007
Aurpu	Aureobasidium pullulans var. melanogenum	Ascomycota; Dothideomycetes; Botryosphaeriales	JGI	JGI	Gostincar et al. BMC Genomics. 2014
Altal1	Alternaria alternata	Ascomycota; Dothideomycetes; Pleosporales	JGI	JGI	Zeiner et al. PLoS One. 2016
Claba1	Cladophialophora bantiana CBS 173.52	Ascomycota; Eurotiomycetes; Chaetothyriales	JGI	JGI	Teixeira et al. Stud Mycol. 2017
Capcor1	Capronia coronata	Ascomycota; Eurotiomycetes; Chaetothyriales	JGI	JGI	Teixeira et al. Stud Mycol. 2017
Exoaq1	Exophiala aquamarina	Ascomycota; Eurotiomycetes; Chaetothyriales	JGI	JGI	Teixeira et al. Stud Mycol. 2017
JAPDRQ01	Knufia sp. JES_112	Ascomycota; Eurotiomycetes; Chaetothyriales	NCBI	NCBI	Kurbessoian,T. and Stajich,J.E.
Fonmu1	Fonsecaea multimorphosa	Ascomycota; Eurotiomycetes; Chaetothyriales	JGI	JGI	Teixeira et al. Stud Mycol. 2017
AOBU01	Cyphellophora europaea	Ascomycota; Eurotiomycetes; Chaetothyriales	NCBI	NCBI	Broad Institute

Phach1	Phaeomoniella chlamydospora	Ascomycota; Eurotiomycetes; Phaeomoniellales	JGI	JGI	Morales-Cruz et al. BMC Genomics. 2015
EndpusZ1	Endocarpon pusillum	Ascomycota; Eurotiomycetes; Verrucariales	JGI	JGI	Wang et al. BMC Genomics. 2014
Aspni	Aspergillus niger CBS 513.88	Ascomycota; Eurotiomycetes; Eurotiales	JGI	JGI	Pel et al. Nat Biotechnol. 2007
Penexp1	Penicillium expansum	Ascomycota; Eurotiomycetes; Eurotiales	JGI	JGI	Ballester et al. Mol Plant Microbe Interact. 2015
Cocpo1	Coccidioides posadasii	Ascomycota; Eurotiomycetes; Onygenales	JGI	JGI	Sharpton et al. Genome Research. 2009
JAPETR01	Chrysothrix sp. TS-e1954	Ascomycota; Arthoniomycetes; Arthoniales	NCBI	NCBI	Diaz Escandon et al. Curr. Biol. 2022
JAPETI01	Alyxoria varia	Ascomycota; Arthoniomycetes; Arthoniales	NCBI	NCBI	Diaz Escandon et al. Curr. Biol. 2022
Sclsc1	Sclerotinia sclerotiorum	Ascomycota; Leotiomycetes; Helotiales	JGI	JGI	Amselem et al. PLoS Genet. 2011
PseVKM4520	Pseudogymnoascus sp. VKM F-4520	Ascomycota; Leotiomycetes; Thelebolales	JGI	JGI	Leushkin et al. BMC Genomics. 2015
Erypu1	Erysiphe pulchra	Ascomycota; Leotiomycetes; Erysiphales	JGI	JGI	Wadl et al. Plant Dis. 2019
Artol1	Arthrobotrys oligospora	Ascomycota; Orbiliomycetes; Orbiliales	JGI	JGI	Yang et al. PLoS Pathog. 7 e1002179 (2011)
Monha1	Monacrosporium haptotylum	Ascomycota; Orbiliomycetes; Orbiliales	JGI	JGI	Meerupati et al. PLoS Genet. 9 e1003909 (2013)
JAPETW01	Lichina confinis	Ascomycota; Lichinomycetes; Lichinales	NCBI	NCBI	Diaz Escandon et al. Curr. Biol. 2022
JAPETP01	Candelina mexicana	Ascomycota; Lichinomycetes; Candelariales	NCBI	NCBI	Diaz Escandon et al. Curr. Biol. 2022
JAPETN01	Candelaria pacifica	Ascomycota; Lichinomycetes; Candelariales	NCBI	NCBI	Diaz Escandon et al. Curr. Biol. 2022
JAPEUD01	Sarea resinae	Ascomycota; Lichinomycetes; Sareales	NCBI	NCBI	Diaz Escandon et al. Curr. Biol. 2022
JAPEUF01	Thelocarpon impressellum	Ascomycota; Lichinomycetes; Thelocarpales	NCBI	NCBI	Diaz Escandon et al. Curr. Biol. 2022
CAJPDR01	Alectoria fallacina	Ascomycota; Lecanoromycetes; Lecanorales	NCBI	NCBI	Tagirdzhanova et al. Mol Ecol. 2021
Clagr3	Cladonia grayi	Ascomycota; Lecanoromycetes; Lecanorales	JGI	JGI	Armaleo et al. BMC Genomics, 2019
JALAHW01	Teloschistes peruensis	Ascomycota; Lecanoromycetes; Teloschistales	NCBI	NCBI	Llewellyn et al. Genome Biol Evol. 2023

JALAII01	Xanthoria aureola	Ascomycota; Lecanoromycetes; Teloschistales	NCBI	NCBI	Llewellyn et al. Genome Biol Evol. 2023
JALAIK01	Xanthoria steineri	Ascomycota; Lecanoromycetes; Teloschistales	NCBI	NCBI	Llewellyn et al. Genome Biol Evol. 2023
JALAIA01	Xanthoria mediterranea	Ascomycota; Lecanoromycetes; Teloschistales	NCBI	NCBI	Llewellyn et al. Genome Biol Evol. 2023
JALAIP01	Caloplaca aegaea	Ascomycota; Lecanoromycetes; Teloschistales	NCBI	NCBI	Llewellyn et al. Genome Biol Evol. 2023
Grascr1	Graphis scripta	Ascomycota; Lecanoromycetes; Ostropales	JGI	JGI	McDonald et al. BMC Genomics. 2013
Umbpus1	Lassalia pustulata	Ascomycota; Lecanoromycetes; Umbilicariales	JGI	JGI	Dal Grande et al. Lichenologist. 2018
Acastr1	Acarospora strigata	Ascomycota; Lecanoromycetes; Acarosporales	JGI	JGI	McDonald et al. BMC Genomics. 2013
Dibbae1	Dibaeis baeomyces	Ascomycota; Lecanoromycetes; Pertusariales	JGI	JGI	McDonald et al. BMC Genomics. 2013
Cenge3	Cenococcum geophilum	Ascomycota; Dothideomycetes; Gloniales	JGI	JGI	Kuo et al. Nat Comun. 2016
JAXLPM01	Exophiala xenobiotica	Ascomycota; Eurotiomycetes; Chaetothyriales	NCBI	NCBI	Coleine et al. Fungal Diversity. 2024
JAPCWZ01	Apiospora arundinis	Ascomycota; Sordariomycetes; Amphisphaeriales	NCBI	NCBI	Sorensen et al. IMA fungus. 2024
JAQOWY01	Colletotrichum chrysophilum	Ascomycota; Sordariomycetes; Glomerellales	NCBI	NCBI	Amaral Carneiro et al. Journal of Plant Pathology. 2023
NCTX01	Epicoccum nigrum	Ascomycota; Dothideomycetes; Pleosporales	NCBI	NCBI	Fokin et al. Genome Announcements. 2017
JOWA01	Scedosporium apiospermum	Ascomycota; Sordariomycetes; Microascales	NCBI	NCBI	Vandeputte et al. Genome announcements. 2014
JAKUDF01	Eutypa lata	Ascomycota; Sordariomycetes; Xylariales	NCBI	NCBI	Onetto et al. PLoS genetics. 2022
CAWUON01	Sporothrix epigloea	Ascomycota; Sordariomycetes; Ophiostomatales	NCBI	NCBI	Allen et al. GBE. 2025
JAPDRN01	Knufia peltigerae	Ascomycota; Eurotiomycetes; Chaetothyriales	NCBI	NCBI	Kurbessoian,T. and Stajich,J.E.
JAPETQ01	Chaenotheca gracillima	Ascomycota; Lichinomycetes; Coniocybales	NCBI	NCBI	Diaz Escandon et al. Curr. Biol. 2022
JAJKKQ01	Hypoxylon rubiginosum	Ascomycota; Sordariomycetes; Xylariales	NCBI	NCBI	Franco et al. New Phytologist. 2022

APIU01	Stachybotrys chartarum	Ascomycota; Sordariomycetes; Hypocreales	NCBI	NCBI	Semeiks et al. BMC Genomics. 2014
JAFKAH01	Verticillium longisporum	Ascomycota; Sordariomycetes; Glomerellales	NCBI	NCBI	Harting et al. Molecular Plant Pathology. 2021
JBHFEH01	Lepraria finkii	Ascomycota; Lecanoromycetes; Lecanorales	NCBI	NCBI	Doellman et al. BMC genomics. 2024
JAPETU01	Geoglossum simile	Ascomycota; Lichinomycetes; Geoglossales	NCBI	NCBI	Diaz Escandon et al. Curr. Biol. 2022
JAAEIX01	Viridothelium virens	Ascomycota; Dothideomycetes; Trypetheliales	NCBI	NCBI	Haridas et al. Stud. Mycol. 2020
LGTZ01	Blastomyces percursus	Ascomycota; Eurotiomycetes; Onygenales	NCBI	NCBI	Dukik et al. Mycoses. 2017
JBBIWK01	Lipomyces starkeyi	Ascomycota; Lipomycetes; Lipomycetales	NCBI	NCBI	Czajka et al. Front Bioeng Biotechnol. 2024
JACCEH01	Tuber indicum	Ascomycota; Pezizomycetes; Pezizales	NCBI	NCBI	Morin et al. Microbiology Resource Announcements. 2021
LHQR01	Penicillium griseofulvum	Ascomycota; Eurotiomycetes; Eurotiales	NCBI	NCBI	Banani et al. BMC Genomics. 2016
JALDZD01	Icmadophila ericetorum	Ascomycota; Lecanoromycetes; Pertusariales	NCBI	NCBI	Resl et al. Nat. Comun. 2022
JALDZE01	Lambiella insularis	Ascomycota; Lecanoromycetes; Baeomycetales	NCBI	NCBI	Resl et al. Nat. Comun. 2022
JAAEJJ01	Mytilinidion resinicola	Ascomycota; Dothideomycetes; Mytilinidiales	NCBI	NCBI	Haridas et al. Stud. Mycol. 2020
RWIE02	Talaromyces pinophilus	Ascomycota; Eurotiomycetes; Eurotiales	NCBI	NCBI	Adlakha et al.
GCA_017310585	Histoplasma capsulatum	Ascomycota; Eurotiomycetes; Onygenales	NCBI	NCBI	Voorhies et al. MBio 13. 2022
JALDZQ01	Stictis urceolata	Ascomycota; Lecanoromycetes; Ostropales	NCBI	NCBI	Resl et al. Nat. Comun. 2022
JAKWBI02	Zalerion maritima	Ascomycota; Sordariomycetes; Lulworthiales	NCBI	NCBI	Paço et al. Microbiology Resource Announcements. 2023
CAJPDS01	Heterodermia speciosa	Ascomycota; Lecanoromycetes; Caliciales	NCBI	NCBI	Tagirdzhanova et al. Mol Ecol. 2021
CAJPDT01	Imshaugia aleurites	Ascomycota; Lecanoromycetes; Lecanorales	NCBI	NCBI	Tagirdzhanova et al. Mol Ecol. 2021
JALDZH01	Lobaria immixta	Ascomycota; Lecanoromycetes; Peltigerales	NCBI	NCBI	Resl et al. Nat. Comun. 2022

JALDZG01	Lignoscripta atroalba	Ascomycota; Lecanoromycetes; Trapeliales	NCBI	NCBI	Resl et al. Nat. Comun. 2022
JALDZS01	Toensbergia leucococca	Ascomycota; Lecanoromycetes; Rhizocarpales	NCBI	NCBI	Resl et al. Nat. Comun. 2022
JALDZL01	Pseudocyphellaria aurata	Ascomycota; Lecanoromycetes; Peltigerales	NCBI	NCBI	Resl et al. Nat. Comun. 2022
JALDZU01	Varicellaria rhodocarp	Ascomycota; Lecanoromycetes; Pertusariales	NCBI	NCBI	Resl et al. Nat. Comun. 2022
JALDZI01	Loxospora ochrophaea	Ascomycota; Lecanoromycetes; Sarrameanales	NCBI	NCBI	Resl et al. Nat. Comun. 2022
JALDZC01	Hypocenomyce scalaris	Ascomycota; Lecanoromycetes; Umbilicariales	NCBI	NCBI	Resl et al. Nat. Comun. 2022
JALEAC01	Xylographa vitiligo	Ascomycota; Lecanoromycetes; Baeomycetales	NCBI	NCBI	Resl et al. Nat. Comun. 2022
PDLN01	Coleophoma crateriformis	Ascomycota; Leotiomycetes; Helotiales	NCBI	NCBI	Wingfield et al. IMA Fungus. 2018
SDJM01	Elsinoe fawcettii	Ascomycota; Dothideomycetes; Myriangiales	NCBI	NCBI	Jeffress et al. PLoS ONE. 2020
JALDZR01	Thelotrema lepadinum	Ascomycota; Lecanoromycetes; Ostropales	NCBI	NCBI	Resl et al. Nat. Comun. 2022
JAPEUE01	Sclerophora amabilis	Ascomycota; Lichinomycetes; Coniocybales	NCBI	NCBI	Diaz Escandon et al. Curr. Biol. 2022
AYEN01	Aureobasidium melanogenum	Ascomycota; Dothideomycetes; Dothideales	NCBI	NCBI	Gostin et al. BMC Genomics. 2014
QGMK01	Lachnellula suecica	Ascomycota; Leotiomycetes; Helotiales	NCBI	NCBI	Giroux et al. Phytopathology. 2020
GCA_000227075	Botrytis cinerea	Ascomycota; Leotiomycetes; Helotiales	NCBI	NCBI	Amselem et al. PLoS genetics. 2011
JAPEVC01	Gnomoniopsis sp. IMI 355080	Ascomycota; Sordariomycetes; Diaporthales	NCBI	NCBI	Hill et al. GBE. 2023
JACCJB01	Letharia lupina	Ascomycota; Lecanoromycetes; Lecanorales	NCBI	NCBI	McKenzie et al. Genomics. 2020
ABKH02	Paracoccidioides lutzii	Ascomycota; Eurotiomycetes; Onygenales	NCBI	NCBI	Desjardins et al. PLoS Genet. 2011
PDNA01	Polytolypa hystricis	Ascomycota; Eurotiomycetes; Onygenales	NCBI	NCBI	Muñoz et al. Scientific reports. 2018.
JAIBSO01	Aspergillus fumigatus	Ascomycota; Eurotiomycetes; Eurotiales	NCBI	NCBI	Barber et al. Nature microbiology. 2021
NPHW01	Elaphomyces granulatus	Ascomycota; Eurotiomycetes; Eurotiales	NCBI	NCBI	Quandt et al. Environ. Microbiol. 2015

JAVRRQ01	Oleoguttula sp. CCFEE 6159	Ascomycota; Dothideomycetes; Mycosphaerellales	NCBI	NCBI	Coleine et al. Fungal Diversity. 2024
JAROPH01	Paecilomyces variotii	Ascomycota; Eurotiomycetes; Eurotiales	NCBI	NCBI	Van Den Brule et al.
VIFY01	Monascus purpureus	Ascomycota; Eurotiomycetes; Eurotiales	NCBI	NCBI	Geng,C. and Zhang,Y
JAJNNR01	Daldinia caldariorum	Ascomycota; Sordariomycetes; Xylariales	NCBI	NCBI	Franco et al. New Phytologist. 2022
SKBQ01	Thyridium curvatum	Ascomycota; Sordariomycetes; Thyridiales	NCBI	NCBI	Varaljay et al. Microbiology Resource Announcements. 2019
LFRF01	Tolypocladium ophioglossoides	Ascomycota; Sordariomycetes; Hypocreales	NCBI	NCBI	Quandt et al. BMC Genomics. 2015
JASJSX01	Parachaetomium inaequale	Ascomycota; Sordariomycetes; Sordariales	NCBI	NCBI	Hensen et al. Mol Phylogenet Evol. 2023
AACU03	Pyricularia oryzae	Ascomycota; Sordariomycetes; Magnaporthales	NCBI	NCBI	Dean et al. Nature. 2005
coassembly_bin_64	Chaetothyriales	Ascomycota; Eurotiomycetes; Chaetothyriales	NCBI	Tagirdzhanova et al. Curr Biol. 2025	Tagirdzhanova et al. Curr Biol. 2025
coassembly_bin_76	Chaetothyriales	Ascomycota; Eurotiomycetes; Chaetothyriales	NCBI	Tagirdzhanova et al. Curr Biol. 2025	Tagirdzhanova et al. Curr Biol. 2025
GTX0466_bin_15	Chaetothyriales	Ascomycota; Eurotiomycetes; Chaetothyriales	NCBI	Tagirdzhanova et al. Curr Biol. 2025	Tagirdzhanova et al. Curr Biol. 2025

Table S15. Sequences recovered as a part of the putative HGT clades in the tyrR phylogeny. Three clades were selected as putative lichen-related HGT based on the prevalence of tyrR sequences from lichen-forming (mycobionts) and lichen-associated (endolichenic) fungi, and on the discordance with the species tree. For each sequences, we list the clade (A-C), the taxonomic assignement for the fungus, and its lifestyle.

Captain Sequence	Genome	Source	taxon	lifestyle	Clade
chaetothyriales.sp..1_tyr3637	chaetothyriales.sp..1	Newly-annotated lichen genomes	Chaetothyriales	Non-mycobiont lichen-associated	Clade A
chaetothyriales.sp..1_tyr954	chaetothyriales.sp..1	Newly-annotated lichen genomes	Chaetothyriales	Non-mycobiont lichen-associated	Clade A
cladophialophora.endolichena.1_tyr5	cladophialophora.endolichena.1	Newly-annotated lichen genomes	Chaetothyriales	Non-mycobiont lichen-associated	Clade A
cladophialophora.endolichena.1_tyr969	cladophialophora.endolichena.1	Newly-annotated lichen genomes	Chaetothyriales	Non-mycobiont lichen-associated	Clade A
cladophialophora.endolichena.1_tyr7	cladophialophora.endolichena.1	Newly-annotated lichen genomes	Chaetothyriales	Non-mycobiont lichen-associated	Clade A
cladophialophora.sp..1_tyr3153	cladophialophora.sp..1	Newly-annotated lichen genomes	Chaetothyriales	Non-mycobiont lichen-associated	Clade A
cladophialophora.sp..2_tyr3779	cladophialophora.sp..2	Newly-annotated lichen genomes	Chaetothyriales	Non-mycobiont lichen-associated	Clade A
cladophialophora.sp..2_tyr3780	cladophialophora.sp..2	Newly-annotated lichen genomes	Chaetothyriales	Non-mycobiont lichen-associated	Clade A
cladophialophora.sp..2_tyr403	cladophialophora.sp..2	Newly-annotated lichen genomes	Chaetothyriales	Non-mycobiont lichen-associated	Clade A
clapsa1_10577	clapsa1	Gluck-Thaler and Vogan (2024)	Chaetothyriales	Non-Lichen	Clade A
clapsa1_9549	clapsa1	Gluck-Thaler and Vogan (2024)	Chaetothyriales	Non-Lichen	Clade A
cypeur1_114146	cypeur1	Gluck-Thaler and Vogan (2024)	Chaetothyriales	Non-Lichen	Clade A
exospi1_9399	exospi1	Gluck-Thaler and Vogan (2024)	Chaetothyriales	Non-Lichen	Clade A
melanina.sp..1_tyr579	melanina.sp..1	Newly-annotated lichen genomes	Chaetothyriales	Non-mycobiont lichen-associated	Clade A
melanina.sp..2_tyr2558	melanina.sp..2	Newly-annotated lichen genomes	Chaetothyriales	Non-mycobiont lichen-associated	Clade A
melanina.sp..2_tyr361	melanina.sp..2	Newly-annotated lichen genomes	Chaetothyriales	Non-mycobiont lichen-associated	Clade A
melanina.sp..2_tyr580	melanina.sp..2	Newly-annotated lichen genomes	Chaetothyriales	Non-mycobiont lichen-associated	Clade A

melanina.sp..2_tyr4104	melanina.sp..2	Newly-annotated lichen genomes	Chaetothyriales	Non-mycobiont lichen-associated	Clade A
rhimac1_11078	rhimac1		Chaetothyriales	Non-Lichen	Clade A
botryolepraria.lesdainii.1_tyr1357	botryolepraria.lesdainii.1	Newly-annotated lichen genomes	Lecanoromycetes	Lichen-forming	Clade A
dermiscellum.oulocheilum.1_tyr302	dermiscellum.oulocheilum.1	Newly-annotated lichen genomes	Lecanoromycetes	Lichen-forming	Clade A
gyalecta.farlowii.1_tyr1367	gyalecta.farlowii.1	Newly-annotated lichen genomes	Lecanoromycetes	Lichen-forming	Clade A
gyalolechia.sp..2_tyr1934	gyalolechia.sp..2	Newly-annotated lichen genomes	Lecanoromycetes	Lichen-forming	Clade A
gypsoplaca.macrophylla.1_tyr2626	gypsoplaca.macrophylla.1	Newly-annotated lichen genomes	Lecanoromycetes	Lichen-forming	Clade A
lambiella.insularis.1_tyr2208	lambiella.insularis.1	Newly-annotated lichen genomes	Lecanoromycetes	Lichen-forming	Clade A
lambiella.insularis.1_tyr2292	lambiella.insularis.1	Newly-annotated lichen genomes	Lecanoromycetes	Lichen-forming	Clade A
phlyctis.petraea.1_tyr3429	phlyctis.petraea.1	Newly-annotated lichen genomes	Lecanoromycetes	Lichen-forming	Clade A
pseudosagedia.isidiata.1_tyr707	pseudosagedia.isidiata.1	Newly-annotated lichen genomes	Lecanoromycetes	Lichen-forming	Clade A
rhizocarpon.geographicum.1_tyr3008	rhizocarpon.geographicum.1	Newly-annotated lichen genomes	Lecanoromycetes	Lichen-forming	Clade A
sticta.canariensis.1_tyr1098	sticta.canariensis.1	Newly-annotated lichen genomes	Lecanoromycetes	Lichen-forming	Clade A
umbilicaria.freyi.1_tyr3723	umbilicaria.freyi.1	Newly-annotated lichen genomes	Lecanoromycetes	Lichen-forming	Clade A
umbilicaria.grisea.1_tyr3321	umbilicaria.grisea.1	Newly-annotated lichen genomes	Lecanoromycetes	Lichen-forming	Clade A
umbilicaria.grisea.2_tyr2582	umbilicaria.grisea.2	Newly-annotated lichen genomes	Lecanoromycetes	Lichen-forming	Clade A
umbilicaria.grisea.3_tyr2971	umbilicaria.grisea.3	Newly-annotated lichen genomes	Lecanoromycetes	Lichen-forming	Clade A
umbilicaria.mammulata.1_tyr2778	umbilicaria.mammulata.1	Newly-annotated lichen genomes	Lecanoromycetes	Lichen-forming	Clade A
umbilicaria.polyphylla.2_tyr2880	umbilicaria.polyphylla.2	Newly-annotated lichen genomes	Lecanoromycetes	Lichen-forming	Clade A
umbilicaria.spodochroa.1_tyr3402	umbilicaria.spodochroa.1	Newly-annotated lichen genomes	Lecanoromycetes	Lichen-forming	Clade A

umbilicaria.spodochroa.3_tyr1175	umbilicaria.spodochroa.3	Newly-annotated lichen genomes	Lecanoromycetes	Lichen-forming	Clade A
umbilicaria.spodochroa.4_tyr2480	umbilicaria.spodochroa.4	Newly-annotated lichen genomes	Lecanoromycetes	Lichen-forming	Clade A
chasp.7_tyr3993	chasp.7	Gluck-Thaler and Vogan (2024)	Other Eurotiomycetes	Non-Lichen	Clade A
elagra1_tyr4783	elagra1	Gluck-Thaler and Vogan (2024)	Other Eurotiomycetes	Non-Lichen	Clade A
elagra2_tyr4830	elagra2	Gluck-Thaler and Vogan (2024)	Other Eurotiomycetes	Non-Lichen	Clade A
endocarpon.pusillum.2_tyr109	endocarpon.pusillum.2	Newly-annotated lichen genomes	Other Eurotiomycetes	Lichen-forming	Clade A
endocarpon.pusillum.2_tyr1208	endocarpon.pusillum.2	Newly-annotated lichen genomes	Other Eurotiomycetes	Lichen-forming	Clade A
endocarpon.pusillum.2_tyr1874	endocarpon.pusillum.2	Newly-annotated lichen genomes	Other Eurotiomycetes	Lichen-forming	Clade A
endocarpon.pusillum.2_tyr350	endocarpon.pusillum.2	Newly-annotated lichen genomes	Other Eurotiomycetes	Lichen-forming	Clade A
endocarpon.pusillum.3_tyr1094	endocarpon.pusillum.3	Newly-annotated lichen genomes	Other Eurotiomycetes	Lichen-forming	Clade A
endocarpon.pusillum.3_tyr187	endocarpon.pusillum.3	Newly-annotated lichen genomes	Other Eurotiomycetes	Lichen-forming	Clade A
muellerella.sp..1_tyr1791	muellerella.sp..1	Newly-annotated lichen genomes	Other Eurotiomycetes	Non-mycobiont lichen-associated	Clade A
phachl1_4316	phachl1	Gluck-Thaler and Vogan (2024)	Other Eurotiomycetes	Non-Lichen	Clade A
phachl1_5955	phachl1	Gluck-Thaler and Vogan (2024)	Other Eurotiomycetes	Non-Lichen	Clade A
beabas1_10106	beabas1	Gluck-Thaler and Vogan (2024)	Outgroup	Outgroup	Clade A
colnym1_1013311	colnym1	Gluck-Thaler and Vogan (2024)	Sordariomycetes	Non-Lichen	Clade A
capepi1_1814	capepi1	Gluck-Thaler and Vogan (2024)	Chaetothyriales	Non-Lichen	Clade B
chaetothyriales.sp..1_tyr955	chaetothyriales.sp..1	Newly-annotated lichen genomes	Chaetothyriales	Non-mycobiont lichen-associated	Clade B
melanina.sp..2_tyr3167	melanina.sp..2	Newly-annotated lichen genomes	Chaetothyriales	Non-mycobiont lichen-associated	Clade B
dimelaena.oreina.1_tyr2942	dimelaena.oreina.1	Newly-annotated lichen genomes	Lecanoromycetes	Lichen-forming	Clade B

leprocaulales.sp..1_tyr1393	leprocaulales.sp..1	Newly-annotated lichen genomes	Lecanoromycetes	Lichen-forming	Clade B
porpidia.contraponenda.1_tyr415	porpidia.contraponenda.1	Newly-annotated lichen genomes	Lecanoromycetes	Lichen-forming	Clade B
rhizocarpon.subgeminatum.1_tyr3275	rhizocarpon.subgeminatum.1	Newly-annotated lichen genomes	Lecanoromycetes	Lichen-forming	Clade B
umbilicaria.crustulosa.1_tyr1022	umbilicaria.crustulosa.1	Newly-annotated lichen genomes	Lecanoromycetes	Lichen-forming	Clade B
umbilicaria.crustulosa.2_tyr2734	umbilicaria.crustulosa.2	Newly-annotated lichen genomes	Lecanoromycetes	Lichen-forming	Clade B
umbilicaria.deusta.4_tyr2123	umbilicaria.deusta.4	Newly-annotated lichen genomes	Lecanoromycetes	Lichen-forming	Clade B
umbilicaria.spodochroa.2_tyr2686	umbilicaria.spodochroa.2	Newly-annotated lichen genomes	Lecanoromycetes	Lichen-forming	Clade B
endocarpon.pusillum.1_tyr2645	endocarpon.pusillum.1	Newly-annotated lichen genomes	Other Eurotiomycetes	Lichen-forming	Clade B
endocarpon.pusillum.1_tyr3654	endocarpon.pusillum.1	Newly-annotated lichen genomes	Other Eurotiomycetes	Lichen-forming	Clade B
endocarpon.pusillum.1_tyr746	endocarpon.pusillum.1	Newly-annotated lichen genomes	Other Eurotiomycetes	Lichen-forming	Clade B
endocarpon.pusillum.2_tyr309	endocarpon.pusillum.2	Newly-annotated lichen genomes	Other Eurotiomycetes	Lichen-forming	Clade B
muellerella.sp..1_tyr1325	muellerella.sp..1	Newly-annotated lichen genomes	Other Eurotiomycetes	Non-mycobiont lichen-associated	Clade B
muellerella.sp..1_tyr3262	muellerella.sp..1	Newly-annotated lichen genomes	Other Eurotiomycetes	Non-mycobiont lichen-associated	Clade B
muellerella.sp..1_tyr3721	muellerella.sp..1	Newly-annotated lichen genomes	Other Eurotiomycetes	Non-mycobiont lichen-associated	Clade B
phachl1_tyr9076	phachl1	Gluck-Thaler and Vogan (2024)	Other Eurotiomycetes	Non-Lichen	Clade B
penfla1_tyr8805	penfla1	Gluck-Thaler and Vogan (2024)	Outgroup	Outgroup	Clade B
chrysothrix.sp..1_tyr3844	chrysothrix.sp..1	Newly-annotated lichen genomes	Arthoniomycetes	Lichen-forming	Clade C
coniocarpon.cinnabarinum.2_tyr3740	coniocarpon.cinnabarinum.2	Newly-annotated lichen genomes	Arthoniomycetes	Lichen-forming	Clade C
coniocarpon.cinnabarinum.2_tyr169	coniocarpon.cinnabarinum.2	Newly-annotated lichen genomes	Arthoniomycetes	Lichen-forming	Clade C
opegrapha.moroziana.1_tyr3182	opegrapha.moroziana.1	Newly-annotated lichen genomes	Arthoniomycetes	Lichen-forming	Clade C

cladophialophora.endolichena.1_tyr430	cladophialophora.endolichena.1	Newly-annotated lichen genomes	Chaetothyriales	Non-mycobiont lichen-associated	Clade C
exosid1_tyr5117	exosid1	Gluck-Thaler and Vogan (2024)	Chaetothyriales	Non-Lichen	Clade C
exoxen1_tyr5127	exoxen1	Gluck-Thaler and Vogan (2024)	Chaetothyriales	Non-Lichen	Clade C
rhilig1_72239	rhilig1	Gluck-Thaler and Vogan (2024)	Outgroup	Outgroup	Clade C
acanthothecis.fontana.1_tyr2875	acanthothecis.fontana.1	Newly-annotated lichen genomes	Lecanoromycetes	Lichen-forming	Clade C
bacidia.gigantensis.1_tyr1202	bacidia.gigantensis.1	Newly-annotated lichen genomes	Lecanoromycetes	Lichen-forming	Clade C
bacidia.rubella.1_tyr2101	bacidia.rubella.1	Newly-annotated lichen genomes	Lecanoromycetes	Lichen-forming	Clade C
baeomycetales.sp..4_tyr2128	baeomycetales.sp..4	Newly-annotated lichen genomes	Lecanoromycetes	Lichen-forming	Clade C
biatora.pontica.1_tyr1294	biatora.pontica.1	Newly-annotated lichen genomes	Lecanoromycetes	Lichen-forming	Clade C
brianaria.bauschiana.1_tyr2644	brianaria.bauschiana.1	Newly-annotated lichen genomes	Lecanoromycetes	Lichen-forming	Clade C
brianaria.bauschiana.1_tyr3821	brianaria.bauschiana.1	Newly-annotated lichen genomes	Lecanoromycetes	Lichen-forming	Clade C
bryoria.fremontii.1_tyr2985	bryoria.fremontii.1	Newly-annotated lichen genomes	Lecanoromycetes	Lichen-forming	Clade C
bryoria.fremontii.1_tyr796	bryoria.fremontii.1	Newly-annotated lichen genomes	Lecanoromycetes	Lichen-forming	Clade C
bulbothrix.scortella.1_tyr1565	bulbothrix.scortella.1	Newly-annotated lichen genomes	Lecanoromycetes	Lichen-forming	Clade C
bulbothrix.scortella.1_tyr2019	bulbothrix.scortella.1	Newly-annotated lichen genomes	Lecanoromycetes	Lichen-forming	Clade C
byssoloma.subdiscordans.1_tyr3807	byssoloma.subdiscordans.1	Newly-annotated lichen genomes	Lecanoromycetes	Lichen-forming	Clade C
cetrelia.chicitae.1_tyr1584	cetrelia.chicitae.1	Newly-annotated lichen genomes	Lecanoromycetes	Lichen-forming	Clade C
cladonia.ochrochlora.1_tyr1069	cladonia.ochrochlora.1	Newly-annotated lichen genomes	Lecanoromycetes	Lichen-forming	Clade C
cladonia.petrophila.1_tyr211	cladonia.petrophila.1	Newly-annotated lichen genomes	Lecanoromycetes	Lichen-forming	Clade C
cladonia.pyxidata.1_tyr2964	cladonia.pyxidata.1	Newly-annotated lichen genomes	Lecanoromycetes	Lichen-forming	Clade C

cladonia.rangiferina.2_tyr2351	cladonia.rangiferina.2	Newly-annotated lichen genomes	Lecanoromycetes	Lichen-forming	Clade C
cladonia.rangiferina.3_tyr774	cladonia.rangiferina.3	Newly-annotated lichen genomes	Lecanoromycetes	Lichen-forming	Clade C
cladonia.sp..3_tyr3641	cladonia.sp..3	Newly-annotated lichen genomes	Lecanoromycetes	Lichen-forming	Clade C
cladonia.sp..8_tyr24	cladonia.sp..8	Newly-annotated lichen genomes	Lecanoromycetes	Lichen-forming	Clade C
cladonia.sp..9_tyr1278	cladonia.sp..9	Newly-annotated lichen genomes	Lecanoromycetes	Lichen-forming	Clade C
cladonia.squamosa.1_tyr159	cladonia.squamosa.1	Newly-annotated lichen genomes	Lecanoromycetes	Lichen-forming	Clade C
cladonia.squamosa.4_tyr3051	cladonia.squamosa.4	Newly-annotated lichen genomes	Lecanoromycetes	Lichen-forming	Clade C
cladonia.subtenuis.1_tyr676	cladonia.subtenuis.1	Newly-annotated lichen genomes	Lecanoromycetes	Lichen-forming	Clade C
cladonia.uncialis.1_tyr1299	cladonia.uncialis.1	Newly-annotated lichen genomes	Lecanoromycetes	Lichen-forming	Clade C
cladonia.uncialis.4_tyr103	cladonia.uncialis.4	Newly-annotated lichen genomes	Lecanoromycetes	Lichen-forming	Clade C
clagra1_11292	clagra1	Gluck-Thaler and Vogan (2024)	Lecanoromycetes	Lichen-forming	Clade C
cornicularia.normoerica.2_tyr176	cornicularia.normoerica.2	Newly-annotated lichen genomes	Lecanoromycetes	Lichen-forming	Clade C
crespoa.crozalsiana.1_tyr3017	crespoa.crozalsiana.1	Newly-annotated lichen genomes	Lecanoromycetes	Lichen-forming	Clade C
dermiscellum.oulocheilum.1_tyr3730	dermiscellum.oulocheilum.1	Newly-annotated lichen genomes	Lecanoromycetes	Lichen-forming	Clade C
dimelaena.oreina.1_tyr4064	dimelaena.oreina.1	Newly-annotated lichen genomes	Lecanoromycetes	Lichen-forming	Clade C
evernia.divaricata.1_tyr246	evernia.divaricata.1	Newly-annotated lichen genomes	Lecanoromycetes	Lichen-forming	Clade C
evernia.prunastri.1_tyr3467	evernia.prunastri.1	Newly-annotated lichen genomes	Lecanoromycetes	Lichen-forming	Clade C
evernia.prunastri.1_tyr2325	evernia.prunastri.1	Newly-annotated lichen genomes	Lecanoromycetes	Lichen-forming	Clade C
evernia.prunastri.1_tyr3163	evernia.prunastri.1	Newly-annotated lichen genomes	Lecanoromycetes	Lichen-forming	Clade C
evernia.prunastri.1_tyr3751	evernia.prunastri.1	Newly-annotated lichen genomes	Lecanoromycetes	Lichen-forming	Clade C

evernia.prunastri.1_tyr3749	evernia.prunastri.1	Newly-annotated lichen genomes	Lecanoromycetes	Lichen-forming	Clade C
flavocetraria.cucullata.1_tyr3192	flavocetraria.cucullata.1	Newly-annotated lichen genomes	Lecanoromycetes	Lichen-forming	Clade C
flavopunctelia.flaventior.1_tyr3604	flavopunctelia.flaventior.1	Newly-annotated lichen genomes	Lecanoromycetes	Lichen-forming	Clade C
fulgogasparrea.decipioides.1_tyr2952	fulgogasparrea.decipioides.1	Newly-annotated lichen genomes	Lecanoromycetes	Lichen-forming	Clade C
fuscopannaria.leucosticta.1_tyr817	fuscopannaria.leucosticta.1	Newly-annotated lichen genomes	Lecanoromycetes	Lichen-forming	Clade C
grascr1_10188	grascr1	Gluck-Thaler and Vogan (2024)	Lecanoromycetes	Lichen-forming	Clade C
imsale1_tyr7676	imsale1	Gluck-Thaler and Vogan (2024)	Lecanoromycetes	Lichen-forming	Clade C
imshaugia.aleurites.1_tyr890	imshaugia.aleurites.1	Newly-annotated lichen genomes	Lecanoromycetes	Lichen-forming	Clade C
imshaugia.aleurites.1_tyr2162	imshaugia.aleurites.1	Newly-annotated lichen genomes	Lecanoromycetes	Lichen-forming	Clade C
imshaugia.aleurites.1_tyr304	imshaugia.aleurites.1	Newly-annotated lichen genomes	Lecanoromycetes	Lichen-forming	Clade C
imshaugia.aleurites.1_tyr2620	imshaugia.aleurites.1	Newly-annotated lichen genomes	Lecanoromycetes	Lichen-forming	Clade C
imshaugia.aleurites.2_tyr2746	imshaugia.aleurites.2	Newly-annotated lichen genomes	Lecanoromycetes	Lichen-forming	Clade C
imshaugia.aleurites.2_tyr183	imshaugia.aleurites.2	Newly-annotated lichen genomes	Lecanoromycetes	Lichen-forming	Clade C
imshaugia.aleurites.2_tyr1162	imshaugia.aleurites.2	Newly-annotated lichen genomes	Lecanoromycetes	Lichen-forming	Clade C
lecanora.strobilina.1_tyr1096	lecanora.strobilina.1	Newly-annotated lichen genomes	Lecanoromycetes	Lichen-forming	Clade C
lecanora.subimmergens.1_tyr3127	lecanora.subimmergens.1	Newly-annotated lichen genomes	Lecanoromycetes	Lichen-forming	Clade C
lecanora.subimmergens.1_tyr531	lecanora.subimmergens.1	Newly-annotated lichen genomes	Lecanoromycetes	Lichen-forming	Clade C
lecanorales.sp..1_tyr3277	lecanorales.sp..1	Newly-annotated lichen genomes	Lecanoromycetes	Lichen-forming	Clade C
lecanoromycetes.sp..2_tyr232	lecanoromycetes.sp..2	Newly-annotated lichen genomes	Lecanoromycetes	Lichen-forming	Clade C
lecanoromycetes.sp..5_tyr214	lecanoromycetes.sp..5	Newly-annotated lichen genomes	Lecanoromycetes	Lichen-forming	Clade C

lecanoromycetes.sp..5_tyr2729	lecanoromycetes.sp..5	Newly-annotated lichen genomes	Lecanoromycetes	Lichen-forming	Clade C
lecidella.sp..1_tyr1433	lecidella.sp..1	Newly-annotated lichen genomes	Lecanoromycetes	Lichen-forming	Clade C
lepraria.oxybapha.1_tyr2815	lepraria.oxybapha.1	Newly-annotated lichen genomes	Lecanoromycetes	Lichen-forming	Clade C
lepraria.sp..1_tyr911	lepraria.sp..1	Newly-annotated lichen genomes	Lecanoromycetes	Lichen-forming	Clade C
letrouitia.sp..1_tyr120	letrouitia.sp..1	Newly-annotated lichen genomes	Lecanoromycetes	Lichen-forming	Clade C
letrouitia.subvulpina.1_tyr3600	letrouitia.subvulpina.1	Newly-annotated lichen genomes	Lecanoromycetes	Lichen-forming	Clade C
letrouitia.subvulpina.1_tyr2785	letrouitia.subvulpina.1	Newly-annotated lichen genomes	Lecanoromycetes	Lichen-forming	Clade C
letrouitia.transgressa.1_tyr741	letrouitia.transgressa.1	Newly-annotated lichen genomes	Lecanoromycetes	Lichen-forming	Clade C
letrouitia.transgressa.1_tyr1835	letrouitia.transgressa.1	Newly-annotated lichen genomes	Lecanoromycetes	Lichen-forming	Clade C
letrouitia.transgressa.1_tyr1836	letrouitia.transgressa.1	Newly-annotated lichen genomes	Lecanoromycetes	Lichen-forming	Clade C
letrouitia.transgressa.2_tyr37	letrouitia.transgressa.2	Newly-annotated lichen genomes	Lecanoromycetes	Lichen-forming	Clade C
letrouitia.transgressa.2_tyr670	letrouitia.transgressa.2	Newly-annotated lichen genomes	Lecanoromycetes	Lichen-forming	Clade C
micareopsis.irriguata.1_tyr82	micareopsis.irriguata.1	Newly-annotated lichen genomes	Lecanoromycetes	Lichen-forming	Clade C
mycoblastus.sanguinarius.1_tyr1766	mycoblastus.sanguinarius.1	Newly-annotated lichen genomes	Lecanoromycetes	Lichen-forming	Clade C
mycoblastus.sanguinarius.2_tyr3880	mycoblastus.sanguinarius.2	Newly-annotated lichen genomes	Lecanoromycetes	Lichen-forming	Clade C
nadvornikia.sorediata.1_tyr881	nadvornikia.sorediata.1	Newly-annotated lichen genomes	Lecanoromycetes	Lichen-forming	Clade C
nadvornikia.sorediata.1_tyr3379	nadvornikia.sorediata.1	Newly-annotated lichen genomes	Lecanoromycetes	Lichen-forming	Clade C
nephroma.helveticum.1_tyr1188	nephroma.helveticum.1	Newly-annotated lichen genomes	Lecanoromycetes	Lichen-forming	Clade C
niebla.homalea.1_tyr2552	niebla.homalea.1	Newly-annotated lichen genomes	Lecanoromycetes	Lichen-forming	Clade C
ochrolechia.trochophora.1_tyr1000	ochrolechia.trochophora.1	Newly-annotated lichen genomes	Lecanoromycetes	Lichen-forming	Clade C

ochrolechia.trochophora.1_tyr784	ochrolechia.trochophora.1	Newly-annotated lichen genomes	Lecanoromycetes	Lichen-forming	Clade C
parmeliaceae.sp..15_tyr1785	parmeliaceae.sp..15	Newly-annotated lichen genomes	Lecanoromycetes	Lichen-forming	Clade C
parmeliaceae.sp..2_tyr3179	parmeliaceae.sp..2	Newly-annotated lichen genomes	Lecanoromycetes	Lichen-forming	Clade C
parmeliaceae.sp..2_tyr1548	parmeliaceae.sp..2	Newly-annotated lichen genomes	Lecanoromycetes	Lichen-forming	Clade C
parmeliaceae.sp..3_tyr570	parmeliaceae.sp..3	Newly-annotated lichen genomes	Lecanoromycetes	Lichen-forming	Clade C
parmeliaceae.sp..6_tyr3757	parmeliaceae.sp..6	Newly-annotated lichen genomes	Lecanoromycetes	Lichen-forming	Clade C
parmeliaceae.sp..6_tyr1684	parmeliaceae.sp..6	Newly-annotated lichen genomes	Lecanoromycetes	Lichen-forming	Clade C
parmotrema.hypotropum.1_tyr1906	parmotrema.hypotropum.1	Newly-annotated lichen genomes	Lecanoromycetes	Lichen-forming	Clade C
parmotrema.hypotropum.1_tyr1144	parmotrema.hypotropum.1	Newly-annotated lichen genomes	Lecanoromycetes	Lichen-forming	Clade C
parmotrema.perlatum.1_tyr3500	parmotrema.perlatum.1	Newly-annotated lichen genomes	Lecanoromycetes	Lichen-forming	Clade C
phaeophyscia.rubropulchra.1_tyr537	phaeophyscia.rubropulchra.1	Newly-annotated lichen genomes	Lecanoromycetes	Lichen-forming	Clade C
phlyctis.petraea.1_tyr2956	phlyctis.petraea.1	Newly-annotated lichen genomes	Lecanoromycetes	Lichen-forming	Clade C
platismatia.glauca.1_tyr3613	platismatia.glauca.1	Newly-annotated lichen genomes	Lecanoromycetes	Lichen-forming	Clade C
platismatia.glauca.1_tyr1582	platismatia.glauca.1	Newly-annotated lichen genomes	Lecanoromycetes	Lichen-forming	Clade C
platismatia.glauca.1_tyr1012	platismatia.glauca.1	Newly-annotated lichen genomes	Lecanoromycetes	Lichen-forming	Clade C
platismatia.glauca.1_tyr3612	platismatia.glauca.1	Newly-annotated lichen genomes	Lecanoromycetes	Lichen-forming	Clade C
platismatia.glauca.1_tyr3812	platismatia.glauca.1	Newly-annotated lichen genomes	Lecanoromycetes	Lichen-forming	Clade C
platismatia.glauca.2_tyr3893	platismatia.glauca.2	Newly-annotated lichen genomes	Lecanoromycetes	Lichen-forming	Clade C
platismatia.glauca.3_tyr2295	platismatia.glauca.3	Newly-annotated lichen genomes	Lecanoromycetes	Lichen-forming	Clade C
platismatia.glauca.3_tyr1502	platismatia.glauca.3	Newly-annotated lichen genomes	Lecanoromycetes	Lichen-forming	Clade C

porina.heterospora.1_tyr1249	porina.heterospora.1	Newly-annotated lichen genomes	Lecanoromycetes	Lichen-forming	Clade C
porpidia.albocaerulescens.1_tyr665	porpidia.albocaerulescens.1	Newly-annotated lichen genomes	Lecanoromycetes	Lichen-forming	Clade C
porpidia.macrocarpa.1_tyr578	porpidia.macrocarpa.1	Newly-annotated lichen genomes	Lecanoromycetes	Lichen-forming	Clade C
protoblastenia.rupestris.1_tyr3083	protoblastenia.rupestris.1	Newly-annotated lichen genomes	Lecanoromycetes	Lichen-forming	Clade C
pseudephebe.minuscula.1_tyr3984	pseudephebe.minuscula.1	Newly-annotated lichen genomes	Lecanoromycetes	Lichen-forming	Clade C
pseudevernia.cladonia.1_tyr1027	pseudevernia.cladonia.1	Newly-annotated lichen genomes	Lecanoromycetes	Lichen-forming	Clade C
pseudevernia.consocians.1_tyr3771	pseudevernia.consocians.1	Newly-annotated lichen genomes	Lecanoromycetes	Lichen-forming	Clade C
pseudevernia.consocians.1_tyr2995	pseudevernia.consocians.1	Newly-annotated lichen genomes	Lecanoromycetes	Lichen-forming	Clade C
pseudevernia.furfuracea.1_tyr327	pseudevernia.furfuracea.1	Newly-annotated lichen genomes	Lecanoromycetes	Lichen-forming	Clade C
pseudevernia.furfuracea.1_tyr3916	pseudevernia.furfuracea.1	Newly-annotated lichen genomes	Lecanoromycetes	Lichen-forming	Clade C
pseudevernia.furfuracea.2_tyr1279	pseudevernia.furfuracea.2	Newly-annotated lichen genomes	Lecanoromycetes	Lichen-forming	Clade C
pseudevernia.furfuracea.3_tyr1486	pseudevernia.furfuracea.3	Newly-annotated lichen genomes	Lecanoromycetes	Lichen-forming	Clade C
pseudevernia.furfuracea.4_tyr3413	pseudevernia.furfuracea.4	Newly-annotated lichen genomes	Lecanoromycetes	Lichen-forming	Clade C
pseudosagedia.sp..1_tyr3004	pseudosagedia.sp..1	Newly-annotated lichen genomes	Lecanoromycetes	Lichen-forming	Clade C
psora.crenata.1_tyr1714	psora.crenata.1	Newly-annotated lichen genomes	Lecanoromycetes	Lichen-forming	Clade C
ramalina.petrina.1_tyr473	ramalina.petrina.1	Newly-annotated lichen genomes	Lecanoromycetes	Lichen-forming	Clade C
ramalina.siliquosa.1_tyr1191	ramalina.siliquosa.1	Newly-annotated lichen genomes	Lecanoromycetes	Lichen-forming	Clade C
solorina.crocea.1_tyr4188	solorina.crocea.1	Newly-annotated lichen genomes	Lecanoromycetes	Lichen-forming	Clade C
stereocaulon.dactylophyllum.1_tyr2235	stereocaulon.dactylophyllum.1	Newly-annotated lichen genomes	Lecanoromycetes	Lichen-forming	Clade C
stereocaulon.dactylophyllum.1_tyr2574	stereocaulon.dactylophyllum.1	Newly-annotated lichen genomes	Lecanoromycetes	Lichen-forming	Clade C

sticta.canariensis.1_tyr4091	sticta.canariensis.1	Newly-annotated lichen genomes	Lecanoromycetes	Lichen-forming	Clade C
stictis.urceolata.1_tyr2397	stictis.urceolata.1	Newly-annotated lichen genomes	Lecanoromycetes	Lichen-forming	Clade C
stictis.urceolata.1_tyr2464	stictis.urceolata.1	Newly-annotated lichen genomes	Lecanoromycetes	Lichen-forming	Clade C
stictis.urceolata.2_tyr1764	stictis.urceolata.2	Newly-annotated lichen genomes	Lecanoromycetes	Lichen-forming	Clade C
stictis.urceolata.2_tyr738	stictis.urceolata.2	Newly-annotated lichen genomes	Lecanoromycetes	Lichen-forming	Clade C
trapelia.coarctata.1_tyr2449	trapelia.coarctata.1	Newly-annotated lichen genomes	Lecanoromycetes	Lichen-forming	Clade C
trapelia.coarctata.1_tyr3269	trapelia.coarctata.1	Newly-annotated lichen genomes	Lecanoromycetes	Lichen-forming	Clade C
trapelia.coarctata.2_tyr1443	trapelia.coarctata.2	Newly-annotated lichen genomes	Lecanoromycetes	Lichen-forming	Clade C
umbilicaria.crustulosa.1_tyr1108	umbilicaria.crustulosa.1	Newly-annotated lichen genomes	Lecanoromycetes	Lichen-forming	Clade C
umbilicaria.crustulosa.1_tyr2611	umbilicaria.crustulosa.1	Newly-annotated lichen genomes	Lecanoromycetes	Lichen-forming	Clade C
umbilicaria.crustulosa.1_tyr450	umbilicaria.crustulosa.1	Newly-annotated lichen genomes	Lecanoromycetes	Lichen-forming	Clade C
umbilicaria.crustulosa.2_tyr1386	umbilicaria.crustulosa.2	Newly-annotated lichen genomes	Lecanoromycetes	Lichen-forming	Clade C
umbilicaria.crustulosa.2_tyr1223	umbilicaria.crustulosa.2	Newly-annotated lichen genomes	Lecanoromycetes	Lichen-forming	Clade C
umbilicaria.crustulosa.2_tyr1382	umbilicaria.crustulosa.2	Newly-annotated lichen genomes	Lecanoromycetes	Lichen-forming	Clade C
umbilicaria.crustulosa.2_tyr2371	umbilicaria.crustulosa.2	Newly-annotated lichen genomes	Lecanoromycetes	Lichen-forming	Clade C
umbilicaria.crustulosa.3_tyr2757	umbilicaria.crustulosa.3	Newly-annotated lichen genomes	Lecanoromycetes	Lichen-forming	Clade C
umbilicaria.freyi.1_tyr2107	umbilicaria.freyi.1	Newly-annotated lichen genomes	Lecanoromycetes	Lichen-forming	Clade C
umbilicaria.freyi.3_tyr3881	umbilicaria.freyi.3	Newly-annotated lichen genomes	Lecanoromycetes	Lichen-forming	Clade C
umbilicaria.hispanica.1_tyr1614	umbilicaria.hispanica.1	Newly-annotated lichen genomes	Lecanoromycetes	Lichen-forming	Clade C
umbilicaria.hispanica.2_tyr2278	umbilicaria.hispanica.2	Newly-annotated lichen genomes	Lecanoromycetes	Lichen-forming	Clade C

umbilicaria.hispanica.3_tyr2280	umbilicaria.hispanica.3	Newly-annotated lichen genomes	Lecanoromycetes	Lichen-forming	Clade C
umbilicaria.iberica.2_tyr1991	umbilicaria.iberica.2	Newly-annotated lichen genomes	Lecanoromycetes	Lichen-forming	Clade C
umbilicaria.mammulata.1_tyr3067	umbilicaria.mammulata.1	Newly-annotated lichen genomes	Lecanoromycetes	Lichen-forming	Clade C
umbilicaria.spodochroa.4_tyr1177	umbilicaria.spodochroa.4	Newly-annotated lichen genomes	Lecanoromycetes	Lichen-forming	Clade C
umbilicaria.subpolyphylla.1_tyr886	umbilicaria.subpolyphylla.1	Newly-annotated lichen genomes	Lecanoromycetes	Lichen-forming	Clade C
umbilicaria.vellea.1_tyr4141	umbilicaria.vellea.1	Newly-annotated lichen genomes	Lecanoromycetes	Lichen-forming	Clade C
usnea.hakonensis.1_tyr2018	usnea.hakonensis.1	Newly-annotated lichen genomes	Lecanoromycetes	Lichen-forming	Clade C
usnea.hakonensis.1_tyr792	usnea.hakonensis.1	Newly-annotated lichen genomes	Lecanoromycetes	Lichen-forming	Clade C
usnea.hakonensis.1_tyr1050	usnea.hakonensis.1	Newly-annotated lichen genomes	Lecanoromycetes	Lichen-forming	Clade C
usnea.hakonensis.1_tyr1059	usnea.hakonensis.1	Newly-annotated lichen genomes	Lecanoromycetes	Lichen-forming	Clade C
usnea.hakonensis.1_tyr4165	usnea.hakonensis.1	Newly-annotated lichen genomes	Lecanoromycetes	Lichen-forming	Clade C
usnea.hakonensis.1_tyr3823	usnea.hakonensis.1	Newly-annotated lichen genomes	Lecanoromycetes	Lichen-forming	Clade C
usnea.hakonensis.1_tyr513	usnea.hakonensis.1	Newly-annotated lichen genomes	Lecanoromycetes	Lichen-forming	Clade C
usnea.hakonensis.1_tyr2187	usnea.hakonensis.1	Newly-annotated lichen genomes	Lecanoromycetes	Lichen-forming	Clade C
usnea.halei.1_tyr3825	usnea.halei.1	Newly-annotated lichen genomes	Lecanoromycetes	Lichen-forming	Clade C
usnea.merrillii.1_tyr1195	usnea.merrillii.1	Newly-annotated lichen genomes	Lecanoromycetes	Lichen-forming	Clade C
usnea.merrillii.1_tyr2828	usnea.merrillii.1	Newly-annotated lichen genomes	Lecanoromycetes	Lichen-forming	Clade C
usnea.merrillii.1_tyr3933	usnea.merrillii.1	Newly-annotated lichen genomes	Lecanoromycetes	Lichen-forming	Clade C
wetmoreana.sp..1_tyr2867	wetmoreana.sp..1	Newly-annotated lichen genomes	Lecanoromycetes	Lichen-forming	Clade C
xanthoria.aureola.1_tyr4090	xanthoria.aureola.1	Newly-annotated lichen genomes	Lecanoromycetes	Lichen-forming	Clade C

elagra1_OXV10984.1	elagra1	Gluck-Thaler and Vogan (2024)	Other Eurotiomycetes	Non-Lichen	Clade C
elagra2_6724	elagra2	Gluck-Thaler and Vogan (2024)	Other Eurotiomycetes	Non-Lichen	Clade C
talpin1_KAF3392106.1	talpin1	Gluck-Thaler and Vogan (2024)	Other Eurotiomycetes	Non-Lichen	Clade C
talrug1_tyr10331	talrug1	Gluck-Thaler and Vogan (2024)	Other Eurotiomycetes	Non-Lichen	Clade C
talsti1_4262	talsti1	Gluck-Thaler and Vogan (2024)	Other Eurotiomycetes	Non-Lichen	Clade C

Table S16. Sequences used in a phylogenetic tree to confirm taxonomy of the genomes.

Sequence description	NCBI Accession Number
AF101285.1 Xanthoria resendei strain BCC_Lich 13259	AF101285.1
AF224347.1 Xanthoria parietina	AF224347.1
AF279888.1 Xanthoria parietina isolate We6144	AF279888.1
AF353944.1 Xanthoria parietina subsp. calcicola	AF353944.1
AJ320118.1 Xanthoria parietina specimen voucher 98	AJ320118.1
AJ320119.1 Xanthoria parietina specimen voucher 100	AJ320119.1
AJ320120.1 Xanthoria parietina specimen voucher 60	AJ320120.1
AJ320121.1 Xanthoria parietina specimen voucher 26	AJ320121.1
AJ320122.1 Xanthoria parietina specimen voucher 27	AJ320122.1
AJ320123.1 Xanthoria parietina specimen voucher 1/1	AJ320123.1
AJ320124.1 Xanthoria parietina specimen voucher 1/2	AJ320124.1
AJ320125.1 Xanthoria parietina specimen voucher 6	AJ320125.1
AJ320126.1 Xanthoria parietina specimen voucher 16	AJ320126.1
AJ320127.1 Xanthoria parietina specimen voucher 89	AJ320127.1
AJ320128.1 Xanthoria parietina specimen voucher 97	AJ320128.1
AJ320129.1 Xanthoria parietina specimen voucher 18	AJ320129.1
AJ320133.1 Xanthoria calcicola specimen voucher 44	AJ320133.1
AJ320135.1 Xanthoria ectaneoides specimen voucher 83, 84	AJ320135.1
AJ320136.1 Xanthoria parietina specimen voucher 106	AJ320136.1
AJ320137.1 Xanthoria parietina specimen voucher 99	AJ320137.1
AJ320140.1 Xanthoria sp. SH85_2001 specimen voucher SH85_2001	AJ320140.1
AJ320141.1 Xanthoria sp. SH87_2001 specimen voucher SH87_2001	AJ320141.1
AJ320142.1 Xanthoria sp. SH5_2001 specimen voucher SH5_2001	AJ320142.1
AJ320144.1 Xanthoria parietina specimen voucher 10	AJ320144.1

AJ320145.1 Xanthoria parietina specimen voucher 14	AJ320145.1
AJ320146.1 Xanthoria parietina specimen voucher 88	AJ320146.1
AJ320147.1 Xanthoria parietina specimen voucher 4	AJ320147.1
AJ320148.1 Xanthoria parietina specimen voucher 91/1, 91/2	AJ320148.1
AJ320149.1 Xanthoria ectaneoides specimen voucher 90/1, 90/2	AJ320149.1
AJ320150.1 Xanthoria calcicola specimen voucher 80	AJ320150.1
AJ320151.1 Xanthoria calcicola specimen voucher 105/2	AJ320151.1
AJ320152.1 Xanthoria calcicola specimen voucher 105/1	AJ320152.1
AM263332.2 Xanthomendoza alfredi specimen voucher Obermayer W. 50_P3 GZU	AM263332.2
AM292806.1 Xanthoria sp. M10t1a1_97.3 isolate M10t1a197.3	AM292806.1
AM292811.1 Xanthoria parietina isolate M51t1av153.6	AM292811.1
AM292820.1 Xanthoria parietina isolate M133t1a197.6	AM292820.1
AM292822.1 Xanthoria ectaneoides isolate L174t1100.3	AM292822.1
AM292823.1 Xanthoria parietina isolate L184t181.16	AM292823.1
AM292824.1 Xanthoria parietina isolate M198t2ad50.7	AM292824.1
AM292828.1 Xanthoria parietina isolate M218t1ad50.10	AM292828.1
AM292829.1 Xanthoria sp. L243t1b_117.9 isolate L243t1b117.9	AM292829.1
AM292838.1 Xanthoria sp. M276t2ad_97.13 isolate M276t2ad97.13	AM292838.1
AM292843.1 Xanthoria sp. L288t3_100.4 isolate L288t3100.4	AM292843.1
AM292844.1 Xanthoria sp. L303t2p2_81.1 isolate L303t2p281.1	AM292844.1
AM292845.1 Xanthoria sp. L304 isolate L304	AM292845.1
AM292851.1 Xanthoria sp. L337t1_127.12 isolate L337t1127.12	AM292851.1
AM292852.1 Xanthoria parietina isolate L348t268.2	AM292852.1
AM292854.1 Xanthoria sp. L356t1p281.19 isolate L356t1p281.19	AM292854.1
AM408403.1 Xanthoria ectaneoides isolate 11775.8	AM408403.1
AM697817.1 Xanthoria sp. 356107.9 specimen voucher R. Honegger Z+ZT 423t2	AM697817.1

AM697819.1 Xanthoria sp. 8662.16 specimen voucher R. Honegger Z+ZT 276t3	AM697819.1
AM697820.1 Xanthoria sp. 1430.3 specimen voucher R. Honegger Z+ZT 11t1	AM697820.1
AM697821.1 Xanthoria sp. 1577143.1 specimen voucher R. Honegger Z+ZT 486t1	AM697821.1
AM697824.1 Xanthoria sp. 1574143.5 specimen voucher R. Honegger Z+ZT 481t2	AM697824.1
AM697825.1 Xanthoria sp. 10768.4 specimen voucher R. Honegger Z+ZT 355t1	AM697825.1
AM697826.1 Xanthoria sp. 1572143.7 specimen voucher R. Honegger Z+ZT 355t2	AM697826.1
AM697827.1 Xanthoria sp. 1645145.5 specimen voucher R. Honegger Z+ZT 358t1	AM697827.1
AM697831.1 Xanthoria sp. 1575143.3 specimen voucher R. Honegger Z+ZT 483t2	AM697831.1
AM697832.1 Xanthoria sp. 1580143.4 specimen voucher R. Honegger Z+ZT 481t1	AM697832.1
AM697833.1 Xanthoria sp. 1576143.2 specimen voucher R. Honegger Z+ZT 483t1	AM697833.1
AM697836.1 Xanthoria parietina specimen voucher R. Honegger Z+ZT 118t1	AM697836.1
AM697837.1 Xanthoria sp. 10581.5 specimen voucher R. Honegger Z+ZT 349t1	AM697837.1
AM697838.1 Xanthoria parietina specimen voucher R. Honegger Z+ZT 348t4	AM697838.1
AM697839.1 Xanthoria parietina specimen voucher R. Honegger Z+ZT 220t1	AM697839.1
AM697840.1 Xanthoria parietina specimen voucher R. Honegger Z+ZT 250t2	AM697840.1
AM697841.1 Xanthoria parietina specimen voucher R. Honegger Z+ZT 271t1	AM697841.1
AM697842.1 Xanthoria parietina specimen voucher R. Honegger Z+ZT 347	AM697842.1
AM697843.1 Xanthoria sp. 11268.8 specimen voucher R. Honegger Z+ZT 357t1	AM697843.1
AM697844.1 Xanthoria parietina specimen voucher R. Honegger Z+ZT 348t3	AM697844.1
AM697845.1 Xanthoria parietina specimen voucher R. Honegger Z+ZT 56t3	AM697845.1
AM697846.1 Xanthoria sp. 852117.20 specimen voucher R. Honegger Z+ZT 451t1	AM697846.1
AM697847.1 Xanthoria parietina specimen voucher R. Honegger Z+ZT 265t1	AM697847.1
AM697848.1 Xanthoria parietina specimen voucher R. Honegger Z+ZT 320t2	AM697848.1
AM697849.1 Xanthoria parietina specimen voucher R. Honegger Z+ZT 260t1	AM697849.1
AM697850.1 Xanthoria parietina specimen voucher R. Honegger Z+ZT 260t2	AM697850.1
AM697851.1 Xanthoria parietina specimen voucher R. Honegger Z+ZT 260t3	AM697851.1

AM697852.1 Xanthoria sp. 93 specimen voucher R. Honegger Z+ZT 287t1	AM697852.1
AM697853.1 Xanthoria parietina specimen voucher R. Honegger Z+ZT 281t2	AM697853.1
AM697854.1 Xanthoria parietina specimen voucher R. Honegger Z+ZT 282t2	AM697854.1
AM697855.1 Xanthoria sp. 944131.1 specimen voucher R. Honegger Z+ZT 355t3	AM697855.1
AM697856.1 Xanthoria sp. 1573143.8 specimen voucher R. Honegger Z+ZT 355t3	AM697856.1
AM697857.1 Xanthoria parietina specimen voucher R. Honegger Z+ZT 479t1	AM697857.1
AM697858.1 Xanthoria parietina specimen voucher R. Honegger Z+ZT 489t1	AM697858.1
AM697859.1 Xanthoria parietina specimen voucher R. Honegger Z+ZT 490t1	AM697859.1
AM697860.1 Xanthoria sp. 12081.3 specimen voucher R. Honegger Z+ZT 385t3	AM697860.1
AM697861.1 Xanthoria sp. 888126.2 specimen voucher R. Honegger Z+ZT 452t1	AM697861.1
AM697862.1 Xanthoria sp. 12276.8 specimen voucher R. Honegger Z+ZT 389t1	AM697862.1
AM697863.1 Xanthoria sp. 1522127.12 specimen voucher R. Honegger Z+ZT 337t1	AM697863.1
AM697865.1 Xanthoria calcicola specimen voucher R. Honegger Z+ZT 460t1	AM697865.1
AM746505.1 Xanthoria sp. 360t3 specimen voucher R. Honegger Z+ZT 360t3	AM746505.1
AY081157.1 Xanthomendoza montana	AY081157.1
AY081159.1 Xanthomendoza ulophyllodes	AY081159.1
AY081160.1 Xanthomendoza alfredi	AY081160.1
AY143398.1 Xanthoria parietina	AY143398.1
AY438275.1 Xanthoria aureola haplotype 16	AY438275.1
AY438276.1 Xanthoria aureola haplotype 17	AY438276.1
AY438280.1 Xanthoria calcicola haplotype 18	AY438280.1
AY438281.1 Xanthoria calcicola haplotype 19	AY438281.1
AY438282.1 Xanthoria calcicola haplotype 20	AY438282.1
AY438297.1 Xanthoria parietina haplotype 1	AY438297.1
AY438298.1 Xanthoria parietina haplotype 2	AY438298.1
AY438299.1 Xanthoria parietina haplotype 3	AY438299.1

AY438300.1 Xanthoria parietina haplotype 4	AY438300.1
AY438301.1 Xanthoria parietina haplotype 5	AY438301.1
AY438302.1 Xanthoria parietina haplotype 6	AY438302.1
AY438303.1 Xanthoria parietina haplotype 7	AY438303.1
AY438304.1 Xanthoria parietina haplotype 8	AY438304.1
AY438305.1 Xanthoria parietina haplotype 9	AY438305.1
AY438306.1 Xanthoria parietina haplotype 10	AY438306.1
AY438307.1 Xanthoria parietina haplotype 11	AY438307.1
AY438308.1 Xanthoria parietina haplotype 12	AY438308.1
AY438309.1 Xanthoria parietina haplotype 13	AY438309.1
AY438310.1 Xanthoria parietina haplotype 14	AY438310.1
AY438311.1 Xanthoria parietina haplotype 15	AY438311.1
AY926499.1 Xanthoria parietina haplotype 1	AY926499.1
AY926500.1 Xanthoria parietina haplotype 2	AY926500.1
AY926501.1 Xanthoria parietina haplotype 3	AY926501.1
AY926502.1 Xanthoria parietina haplotype 4	AY926502.1
AY926503.1 Xanthoria parietina haplotype 5	AY926503.1
AY926504.1 Xanthoria parietina haplotype 6	AY926504.1
AY926505.1 Xanthoria parietina haplotype 7	AY926505.1
AY926506.1 Xanthoria parietina haplotype 8	AY926506.1
AY926507.1 Xanthoria parietina haplotype 9	AY926507.1
AY926508.1 Xanthoria parietina haplotype 10	AY926508.1
AY926509.1 Xanthoria parietina haplotype 11	AY926509.1
AY926510.1 Xanthoria parietina haplotype 12	AY926510.1
AY926511.1 Xanthoria parietina haplotype 13	AY926511.1
AY926512.1 Xanthoria parietina haplotype 14	AY926512.1

AY926513.1 Xanthoria parietina haplotype 15	AY926513.1
AY926514.1 Xanthoria parietina haplotype 16	AY926514.1
DQ472212.1 Xanthoria parietina haplotype 1	DQ472212.1
DQ472213.1 Xanthoria parietina haplotype 2	DQ472213.1
DQ472214.1 Xanthoria parietina haplotype 3	DQ472214.1
DQ472215.1 Xanthoria parietina haplotype 4	DQ472215.1
DQ472216.1 Xanthoria parietina haplotype 5	DQ472216.1
DQ472217.1 Xanthoria parietina haplotype 6	DQ472217.1
DQ472218.1 Xanthoria parietina haplotype 7	DQ472218.1
DQ472219.1 Xanthoria parietina haplotype 8	DQ472219.1
DQ472220.1 Xanthoria parietina haplotype 9	DQ472220.1
DQ472221.1 Xanthoria parietina haplotype 10	DQ472221.1
DQ472222.1 Xanthoria parietina haplotype 11	DQ472222.1
DQ472223.1 Xanthoria parietina haplotype 12	DQ472223.1
DQ472224.1 Xanthoria parietina haplotype 13	DQ472224.1
DQ472225.1 Xanthoria parietina haplotype 14	DQ472225.1
DQ472226.1 Xanthoria parietina haplotype 15	DQ472226.1
DQ472227.1 Xanthoria parietina haplotype 16	DQ472227.1
DQ888716.1 Xanthoria parietina	DQ888716.1
EU266089.1 Xanthoria stenophylla voucher Hur H06083	EU266089.1
EU360734.1 Xanthoria mawsonii haplotype 6	EU360734.1
EU360735.1 Xanthoria mawsonii haplotype 1	EU360735.1
EU360767.1 Xanthoria mawsonii haplotype 2	EU360767.1
EU639640.1 Xanthoria parietina isolate Xpar198	EU639640.1
EU639641.1 Xanthoria resendei isolate Xres233b	EU639641.1
EU681288.1 Xanthoria parietina isolate FNM_017	EU681288.1

EU681289.1 Xanthoria parietina isolate FNM_177	EU681289.1
EU681290.1 Xanthoria parietina isolate FNM_133	EU681290.1
EU681292.1 Xanthoria coomae isolate FNM_007	EU681292.1
EU681294.1 Xanthoria monofoliosa isolate FNM_209	EU681294.1
EU681295.1 Xanthoria calcicola isolate FNM_088	EU681295.1
EU681296.1 Xanthoria calcicola isolate FNM_189	EU681296.1
EU681297.1 Xanthoria mediterranea isolate FNM_193	EU681297.1
EU681299.1 Xanthoria ectaneoides isolate FNM_087	EU681299.1
EU681307.1 Xanthoria streimannii isolate FNM_023	EU681307.1
EU681309.1 Xanthoria elixii isolate FNM_062	EU681309.1
EU681318.1 Xanthoria inflata isolate FNM_124	EU681318.1
EU681325.1 Xanthoria alaskana isolate FNM_210	EU681325.1
EU681329.1 Xanthoria ucrainica isolate FNM_038	EU681329.1
EU681330.1 Xanthoria papillifera isolate FNM_204	EU681330.1
EU681331.1 Xanthoria papillifera isolate FNM_018	EU681331.1
EU681334.1 Xanthoria polycarpa isolate FNM_115	EU681334.1
EU681338.1 Xanthoria digitata isolate FNM_191	EU681338.1
EU681339.1 Xanthoria papillifera isolate FNM_097	EU681339.1
EU681342.1 Xanthoria ulophyllodes isolate FNM_198	EU681342.1
EU681343.1 Xanthoria sp. FNM_030	EU681343.1
EU681345.1 Xanthoria alfredii isolate FNM_152	EU681345.1
EU681354.1 Xanthoria sogdiana isolate FNM_200	EU681354.1
EU681356.1 Xanthoria montana isolate FNM_077	EU681356.1
EU681358.1 Xanthoria coppinsii isolate FNM_106	EU681358.1
EU681359.1 Xanthoria coppinsii isolate FNM_108	EU681359.1
EU681360.1 Xanthoria coppinsii isolate FNM_107	EU681360.1

JF831884.1 Xanthoria parietina voucher M_0102305	JF831884.1
JF831885.1 Xanthoria parietina voucher M_0102307	JF831885.1
JF831886.1 Xanthoria parietina voucher M_0102328	JF831886.1
JF831887.1 Xanthoria parietina voucher M_0102327	JF831887.1
JF831888.1 Xanthoria parietina voucher M_0102325	JF831888.1
JF831889.1 Xanthoria parietina voucher M_0102916	JF831889.1
JF831890.1 Xanthoria parietina voucher M_0102919	JF831890.1
JF831891.1 Xanthoria parietina voucher M_0102308	JF831891.1
JF831892.1 Xanthoria parietina voucher M_0102309	JF831892.1
JF831893.1 Xanthoria parietina voucher M_0102306	JF831893.1
JF831894.1 Xanthoria parietina voucher M_0102316	JF831894.1
JF831895.1 Xanthoria parietina voucher M_0102307	JF831895.1
JF831896.1 Xanthoria parietina voucher M_0102925	JF831896.1
JF831897.1 Xanthoria parietina voucher M_0102151	JF831897.1
JF831898.1 Xanthoria parietina voucher M_0102152	JF831898.1
JF831899.1 Xanthoria parietina voucher M_0102305	JF831899.1
JF831900.1 Xanthoria parietina voucher M_0102305	JF831900.1
JF831901.1 Xanthoria parietina voucher M_0102304	JF831901.1
JF831902.1 Xanthoria parietina voucher M_0102304	JF831902.1
JN984131.1 Xanthoria resendei isolate SK3185	JN984131.1
JN984136.1 Xanthoria monofoliosa isolate SK3191	JN984136.1
JQ301685.1 Teloschistes flavicans	JQ301685.1
JQ301690.1 Xanthoria aureola	JQ301690.1
JQ301691.1 Xanthoria parietina	JQ301691.1
JQ301692.1 Xanthoria sp. Gaya 31	JQ301692.1
KC179405.1 Rusavskia cf. papillifera PF313	KC179405.1

KC179410.1 Xanthoria coomae	KC179410.1
KC179411.1 Xanthoria cf. parietina 1 UA_2013	KC179411.1
KC179412.1 Xanthoria cf. parietina 2 UA_2013	KC179412.1
KF589956.1 Xanthoria parietina isolate pop1_1	KF589956.1
KF589957.1 Xanthoria parietina isolate pop1_2	KF589957.1
KF589958.1 Xanthoria parietina isolate pop1_3	KF589958.1
KF589959.1 Xanthoria parietina isolate pop1_4	KF589959.1
KF589960.1 Xanthoria parietina isolate pop1_5	KF589960.1
KF589961.1 Xanthoria parietina isolate pop1_6	KF589961.1
KF589962.1 Xanthoria parietina isolate pop1_7	KF589962.1
KF589963.1 Xanthoria parietina isolate pop1_8	KF589963.1
KF589964.1 Xanthoria parietina isolate pop1_9	KF589964.1
KF589965.1 Xanthoria parietina isolate pop1_11	KF589965.1
KF589966.1 Xanthoria parietina isolate pop1_12	KF589966.1
KF589967.1 Xanthoria parietina isolate pop1_13	KF589967.1
KF589968.1 Xanthoria parietina isolate pop1_17	KF589968.1
KF589970.1 Xanthoria parietina isolate pop1_19	KF589970.1
KF589971.1 Xanthoria parietina isolate pop1_20	KF589971.1
KF589972.1 Xanthoria parietina isolate pop1_21	KF589972.1
KF589973.1 Xanthoria parietina isolate pop1_22	KF589973.1
KF589974.1 Xanthoria parietina isolate pop2_1	KF589974.1
KF589975.1 Xanthoria parietina isolate pop2_2	KF589975.1
KF589976.1 Xanthoria parietina isolate pop2_3	KF589976.1
KF589977.1 Xanthoria parietina isolate pop2_6	KF589977.1
KF589978.1 Xanthoria parietina isolate pop2_7	KF589978.1
KF589979.1 Xanthoria parietina isolate pop2_8	KF589979.1

KF589980.1 Xanthoria parietina isolate pop2_10	KF589980.1
KF589981.1 Xanthoria parietina isolate pop2_11	KF589981.1
KF589982.1 Xanthoria parietina isolate pop2_12	KF589982.1
KF589983.1 Xanthoria parietina isolate pop2_14	KF589983.1
KF589984.1 Xanthoria parietina isolate pop2_16	KF589984.1
KF589985.1 Xanthoria parietina isolate pop2_18	KF589985.1
KF589986.1 Xanthoria parietina isolate pop2_22	KF589986.1
KF589987.1 Xanthoria parietina isolate pop2_24	KF589987.1
KF589988.1 Xanthoria parietina isolate pop2_25	KF589988.1
KF589989.1 Xanthoria parietina isolate pop2_26	KF589989.1
KF589990.1 Xanthoria parietina isolate pop2_27	KF589990.1
KF589991.1 Xanthoria parietina isolate pop2_29	KF589991.1
KF589992.1 Xanthoria parietina isolate pop3_1	KF589992.1
KF589995.1 Xanthoria parietina isolate pop3_4	KF589995.1
KF589996.1 Xanthoria parietina isolate pop3_5	KF589996.1
KF589997.1 Xanthoria parietina isolate pop3_6	KF589997.1
KF589998.1 Xanthoria parietina isolate pop3_7	KF589998.1
KF589999.1 Xanthoria parietina isolate pop3_8	KF589999.1
KF590000.1 Xanthoria parietina isolate pop3_10	KF590000.1
KF590001.1 Xanthoria parietina isolate pop3_11	KF590001.1
KF590002.1 Xanthoria parietina isolate pop3_22	KF590002.1
KF590003.1 Xanthoria parietina isolate pop3_23	KF590003.1
KF590004.1 Xanthoria parietina isolate pop4_1	KF590004.1
KF590005.1 Xanthoria parietina isolate pop4_2	KF590005.1
KF590006.1 Xanthoria parietina isolate pop4_3	KF590006.1
KF590007.1 Xanthoria parietina isolate pop4_4	KF590007.1

KF590008.1 Xanthoria parietina isolate pop4_5	KF590008.1
KF590009.1 Xanthoria parietina isolate pop4_6	KF590009.1
KF590010.1 Xanthoria parietina isolate pop4_7	KF590010.1
KF590011.1 Xanthoria parietina isolate pop4_10	KF590011.1
KF590012.1 Xanthoria parietina isolate pop4_11	KF590012.1
KF590013.1 Xanthoria parietina isolate pop4_12	KF590013.1
KF590014.1 Xanthoria parietina isolate pop4_13	KF590014.1
KF590015.1 Xanthoria parietina isolate pop4_15	KF590015.1
KF590016.1 Xanthoria parietina isolate pop4_16	KF590016.1
KF590017.1 Xanthoria parietina isolate pop4_18	KF590017.1
KF590018.1 Xanthoria parietina isolate pop4_19	KF590018.1
KF590019.1 Xanthoria parietina isolate pop4_20	KF590019.1
KF590020.1 Xanthoria parietina isolate pop4_21	KF590020.1
KF590021.1 Xanthoria parietina isolate pop4_24	KF590021.1
KF590022.1 Xanthoria parietina isolate pop4_25	KF590022.1
KF590023.1 Xanthoria parietina isolate pop4_26	KF590023.1
KF720397.1 Xanthoria montana voucher Leavitt 6142 BRY_C	KF720397.1
KJ027702.1 Xanthoria parietina isolate I3	KJ027702.1
KJ027703.1 Xanthoria parietina isolate I2	KJ027703.1
KJ027704.1 Xanthoria parietina isolate I1	KJ027704.1
KJ027705.1 Xanthoria parietina isolate D1	KJ027705.1
KJ027706.1 Xanthoria parietina isolate D3	KJ027706.1
KJ027707.1 Xanthoria parietina isolate D6	KJ027707.1
KJ027708.1 Xanthoria parietina isolate D2	KJ027708.1
KJ027709.1 Xanthoria parietina isolate D4	KJ027709.1
KJ027710.1 Xanthoria parietina isolate D5	KJ027710.1

KJ027711.1 Xanthoria parietina isolate D7	KJ027711.1
KT291474.1 Xanthoria montana isolate Xmmon84	KT291474.1
KT291475.1 Xanthoria calcicola isolate Gaya 3	KT291475.1
KU056847.1 Rusavskia dasanensis voucher KoLRI 011123 SK 773	KU056847.1
KU926996.1 Xanthoria nowakii voucher JV14465	KU926996.1
KY379230.1 Xanthoria parietina strain BGK443	KY379230.1
KY748948.1 Rusavskia sp. isolate JM6656	KY748948.1
KY748949.1 Rusavskia sp. isolate L11741	KY748949.1
MG554690.1 Xanthoria parietina strain BGK443	MG554690.1
MG954159.1 Rusavskia sp. voucher Vondrak18207	MG954159.1
MH714517.1 Xanthoria parietina isolate ALV16819	MH714517.1
MK536697.1 Xanthoria sp. clone OTU152	MK536697.1
MK811702.1 Xanthoria parietina voucher O_L_206852	MK811702.1
MK812134.1 Xanthoria parietina voucher O_L_196081	MK812134.1
MK812349.1 Xanthoria parietina voucher O_L_195782	MK812349.1
MN006804.1 Xanthoria polycarpa isolate sample59	MN006804.1
MN006805.1 Xanthoria parietina isolate sample60	MN006805.1
MN006806.1 Xanthoria parietina isolate sample61	MN006806.1
MN006807.1 Xanthoria parietina isolate sample62	MN006807.1
MN985506.1 Xanthoria parietina isolate 98GB04	MN985506.1
MT513231.1 Xanthoria parietina strain L2379	MT513231.1
MT644879.1 Xanthoria parietina isolate DM1149	MT644879.1
MT928333.1 Xanthoria polessica strain U3115	MT928333.1
MW590812.1 Rusavskia sp. voucher BL_2 5.8S	MW590812.1
MZ159401.1 Xanthoria parietina voucher KM 165047	MZ159401.1
MZ196456.1 Xanthoria sp. voucher KHER 12109a	MZ196456.1

MZ196457.1 Xanthoria sp. voucher KHER 12109	MZ196457.1
MZ303030.1 Xanthoria sp. voucher KHER 11232	MZ303030.1
OK491793.1 Xanthoria parietina isolate ZZ_G1_Fungus	OK491793.1
OK577905.1 Xanthoria ulophyllodes var. subsorediosa isolate HP_19L	OK577905.1
ON437600.1 Xanthoria parietina voucher SS0065	ON437600.1
ON510778.1 Xanthoria parietina voucher BYU Field Sample	ON510778.1
OQ249822.1 Rusavskia elegans isolate SF161	OQ249822.1
OQ249823.1 Rusavskia sorediata isolate AM32	OQ249823.1
OQ249839.1 Xanthoria parietina isolate AM310	OQ249839.1
OQ249840.1 Xanthoria parietina isolate AM311	OQ249840.1
OQ249841.1 Xanthoria parietina isolate AM352E	OQ249841.1
OQ249842.1 Xanthoria parietina isolate AM353E	OQ249842.1
OQ249843.1 Xanthoria parietina isolate AM446	OQ249843.1
OQ249844.1 Xanthoria parietina isolate AM447	OQ249844.1
OQ249845.1 Xanthoria parietina isolate AM553	OQ249845.1
OQ249846.1 Xanthoria parietina isolate AM555	OQ249846.1
OQ249847.1 Xanthoria parietina isolate AM559	OQ249847.1
OQ249848.1 Xanthoria parietina isolate AM560	OQ249848.1
OQ249849.1 Xanthoria parietina isolate AM561	OQ249849.1
OQ249850.1 Xanthoria parietina isolate AM562	OQ249850.1
OQ249851.1 Xanthoria parietina isolate AM563	OQ249851.1
OQ249852.1 Xanthoria parietina isolate AM564	OQ249852.1
OQ249853.1 Xanthoria parietina isolate AM565	OQ249853.1
OQ249854.1 Xanthoria parietina isolate AM638	OQ249854.1
OQ249855.1 Xanthoria parietina isolate AM639	OQ249855.1
OQ249856.1 Xanthoria parietina isolate AM640	OQ249856.1

OQ249857.1 Xanthoria parietina isolate AM641	OQ249857.1
OQ616504.1 Xanthoria sp. voucher SS0131	OQ616504.1
Xanthoria_parietina_GTX0501_57:4166-4674	
Xanthoria_calcicola_GTX0491_16:1511-2024	
Xanthoria_parietina_glXanPari20_scaffold_4:3660-4168	
Xanthoria_parietina_glXanPari1_OZ210035.1:1764803-1765311	

Table S17. Metadata of lichen-associated genomes used to find signatures of Starship horizontal transfer amongst lichen symbionts.

Genome ID	Accession ID	Species	Class	Lifestyle	Host	Assembly Release Date	WGS project accession
alyxoria.varia.1	GCA_026054675.1	Alyxoria varia	Arthoniomycetes	Lichen-forming	N/A	11-7-22	JAPETI01
arthonia.radiata.1	GCA_002989075.1	Arthonia radiata	Arthoniomycetes	Lichen-forming	N/A	3-9-18	PSQN01
arthonia.rubella.1	GCA_964256465.1	Arthonia rubella	Arthoniomycetes	Lichen-forming	N/A	9-21-24	CAXVBU01
arthonia.susa.1	GCA_964256665.1	Arthonia susa	Arthoniomycetes	Lichen-forming	N/A	9-21-24	CAXVBT01
arthoniomycete.sp..1	GCA_049996255.1	Arthoniomycete sp.	Arthoniomycetes	Non-mycobiont lichen-associated	Umbillicaria	4-29-25	JBAWTR01
arthoniomycete.sp..2	GCA_964257325.1	Arthoniomycete sp.	Arthoniomycetes	Lichen-forming	N/A	9-21-24	CAXVEJ01
arthothelium.ruanum.1	GCA_964255825.1	Arthothelium ruanum	Arthoniomycetes	Lichen-forming	N/A	9-21-24	CAXUZJ01
chrysothrix.sp..1	GCA_026023555.1	Chrysothrix sp.	Arthoniomycetes	Lichen-forming	N/A	11-7-22	JAPETR01
coniocarpon.cinnabarinum.1	GCA_051942205.1	Coniocarpon cinnabarinum	Arthoniomycetes	Lichen-forming	N/A	8-12-25	JAVECO01
coniocarpon.cinnabarinum.2	GCA_051942265.1	Coniocarpon cinnabarinum	Arthoniomycetes	Lichen-forming	N/A	8-12-25	JAVECK01
opegrapha.moroziana.1	GCA_964257255.1	Opegrapha moroziana	Arthoniomycetes	Lichen-forming	N/A	9-21-24	CAXVED01
zwackhia.viridis.1	GCA_964255065.1	Zwackhia viridis	Arthoniomycetes	Lichen-forming	N/A	9-21-24	CAXUUG01
mycosphaerellales.sp..1	GCA_049996175.1	Mycosphaerellales sp.	Dothidiomycetes	Non-mycobiont lichen-associated	Umbillicaria	4-29-25	JBAWTN01
mycosphaerellales.sp..2	GCA_049996215.1	Mycosphaerellales sp.	Dothidiomycetes	Non-mycobiont lichen-associated	Umbillicaria	4-29-25	JBAWTP01
pseudopyrenochaetaceae.sp..1	GCA_049996235.1	Pseudopyrenochaetaceae sp.	Dothidiomycetes	Non-mycobiont lichen-associated	Umbillicaria	4-29-25	JBAWTQ01
teratosphaeriaceae.sp..1	GCA_049996275.1	Teratosphaeriaceae sp.	Dothidiomycetes	Non-mycobiont lichen-associated	Umbillicaria	4-29-25	JBAWTS01
chaetothyriales.sp..1	GCA_049996355.1	Chaetothyriales sp.	Eurotiomycetes	Non-mycobiont lichen-associated	Umbillicaria	4-29-25	JBAWTW01

cladophialophora.endolichena.1	GCA_049996375.1	Cladophialophora endolichena	Eurotiomycetes	Non-mycobiont lichen-associated	Umbillicaria	4-29-25	JBAWTX01
cladophialophora.sp..1	GCA_049996295.1	Cladophialophora sp.	Eurotiomycetes	Non-mycobiont lichen-associated	Umbillicaria	4-29-25	JBAWTT01
cladophialophora.sp..2	GCA_049996315.1	Cladophialophora sp.	Eurotiomycetes	Non-mycobiont lichen-associated	Umbillicaria	4-29-25	JBAWTU01
endocarpon.pusillum.1	GCA_000464535.1	Endocarpon pusillum	Eurotiomycetes	Lichen-forming	N/A	9-5-13	APWS01
endocarpon.pusillum.2	GCA_000611755.1	Endocarpon pusillum	Eurotiomycetes	Lichen-forming	N/A	4-2-14	JFDM01
endocarpon.pusillum.3	GCA_014607435.1	Endocarpon pusillum	Eurotiomycetes	Lichen-forming	N/A	9-16-20	JAACFV01
exophiala.sp..1	GCA_049996405.1	Exophiala sp.	Eurotiomycetes	Non-mycobiont lichen-associated	Umbillicaria	4-29-25	JBAWTY01
melanina.sp..1	GCA_049996335.1	Melanina sp.	Eurotiomycetes	Non-mycobiont lichen-associated	Umbillicaria	4-29-25	JBAWTV01
melanina.sp..2	GCA_049996395.1	Melanina sp.	Eurotiomycetes	Non-mycobiont lichen-associated	Umbillicaria	4-29-25	JBAWTZ01
muellerella.sp..1	GCA_049996195.1	Muellerella sp.	Eurotiomycetes	Non-mycobiont lichen-associated	Umbillicaria	4-29-25	JBAWTO01
paracladophialophora.sp..1	GCA_049996155.1	Paracladophialophora sp.	Eurotiomycetes	Non-mycobiont lichen-associated	Umbillicaria	4-29-25	JBAWTM01
acanthothecis.fontana.1	GCA_964256675.1	Acanthothecis fontana	Lecanoromycetes	Lichen-forming	N/A	9-21-24	CAXVDH01
acarospora.socialis.1	GCA_025617375.2	Acarospora socialis	Lecanoromycetes	Lichen-forming	N/A	3-31-23	JAMYGW02
acarospora.sp..1	GCA_964255005.1	Acarospora sp.	Lecanoromycetes	Lichen-forming	N/A	9-21-24	CAXUUZ01
acarospora.strigata.1	GCA_022814355.1	Acarospora strigata	Lecanoromycetes	Lichen-forming	N/A	4-4-22	JALDZA01
acarospora.strigata.2	GCA_964256185.1	Acarospora strigata	Lecanoromycetes	Lichen-forming	N/A	9-21-24	CAXUZR01
agyrium.rufum.1	GCA_022814335.1	Agyrium rufum	Lecanoromycetes	Lichen-forming	N/A	4-4-22	JALDZB01
alectoria.fallacina.1	GCA_905337325.1	Alectoria fallacina	Lecanoromycetes	Lichen-forming	N/A	3-25-21	CAJPDR01
alectoria.fallacina.2	GCA_964255875.1	Alectoria fallacina	Lecanoromycetes	Lichen-forming	N/A	9-21-24	CAXUZS01

alectoria.ochroleuca.1	GCA_964257205.1	Alectoria ochroleuca	Lecanoromycetes	Lichen-forming	N/A	9-21-24	CAXVDO01
alectoria.sarmentosa.1	GCA_964255965.1	Alectoria sarmentosa	Lecanoromycetes	Lichen-forming	N/A	9-21-24	CAXUYW01
alectoria.sarmentosa.2	GCA_009733775.1	Alectoria sarmentosa	Lecanoromycetes	Lichen-forming	N/A	12-6-19	WOGZ01
alectoria.sarmentosa.3	GCA_904859925.1	Alectoria sarmentosa	Lecanoromycetes	Lichen-forming	N/A	7-19-23	CAJHES01
alectoria.sarmentosa.4	GCA_904859945.1	Alectoria sarmentosa	Lecanoromycetes	Lichen-forming	N/A	7-19-23	CAJHER01
alectoria.sp..1	GCA_937857905.1	Alectoria sp.	Lecanoromycetes	Lichen-forming	N/A	1-12-23	CALEER01
anzia.colpodes.1	GCA_964254895.1	Anzia colpodes	Lecanoromycetes	Lichen-forming	N/A	9-21-24	CAXUXD01
aspicilia.laevata.1	GCA_964257315.1	Aspicilia laevata	Lecanoromycetes	Lichen-forming	N/A	9-21-24	CAXVER01
bachmanniomyces.sp..1	GCA_022814255.1	Bachmanniomyces sp.	Lecanoromycetes	Lichen-forming	N/A	4-4-22	JALDZF01
bachmanniomyces.sp..2	GCA_964257385.1	Bachmanniomyces sp.	Lecanoromycetes	Lichen-forming	N/A	9-21-24	CAXVEI01
bacidia.gigantensis.1	GCA_019456465.1	Bacidia gigantensis	Lecanoromycetes	Lichen-forming	N/A	8-6-21	JAHXPR01
bacidia.rubella.1	GCA_032158265.1	Bacidia rubella	Lecanoromycetes	Lichen-forming	N/A	9-28-23	JAVTIF01
baeomycetales.sp..1	GCA_964255745.1	Baeomycetales sp.	Lecanoromycetes	Lichen-forming	N/A	9-21-24	CAXUYN01
baeomycetales.sp..2	GCA_964254815.1	Baeomycetales sp.	Lecanoromycetes	Lichen-forming	N/A	9-21-24	CAXUVC01
baeomycetales.sp..3	GCA_964257565.1	Baeomycetales sp.	Lecanoromycetes	Lichen-forming	N/A	9-21-24	CAXVFJ01
baeomycetales.sp..4	GCA_964254865.1	Baeomycetales sp.	Lecanoromycetes	Lichen-forming	N/A	9-21-24	CAXUXO01
biatora.appalachensis.1	GCA_964256235.1	Biatora appalachensis	Lecanoromycetes	Lichen-forming	N/A	9-21-24	CAXVAA01
biatora.pontica.1	GCA_964255495.1	Biatora pontica	Lecanoromycetes	Lichen-forming	N/A	9-21-24	CAXUWI01
blastenia.crenularia.1	GCA_051941205.1	Blastenia crenularia	Lecanoromycetes	Lichen-forming	N/A	8-12-25	JAVEBG01
botryolepraria.lesdainii.1	GCA_964256435.1	Botryolepraria lesdainii	Lecanoromycetes	Lichen-forming	N/A	9-21-24	CAXVBJ01
brianaria.bauschiana.1	GCA_964255265.1	Brianaria bauschiana	Lecanoromycetes	Lichen-forming	N/A	9-21-24	CAXUXS01
brigantiaea.leucoxantha.1	GCA_051941505.1	Brigantiaea leucoxantha	Lecanoromycetes	Lichen-forming	N/A	8-12-25	JAVEAU01
brigantiaea.leucoxantha.2	GCA_964254945.1	Brigantiaea leucoxantha	Lecanoromycetes	Lichen-forming	N/A	9-21-24	CAXUWT01
bryoria.fremontii.1	GCA_964254905.1	Bryoria fremontii	Lecanoromycetes	Lichen-forming	N/A	9-21-24	CAXUUC01
bryoria.tenuis.1	GCA_964255205.1	Bryoria tenuis	Lecanoromycetes	Lichen-forming	N/A	9-21-24	CAXUWM01
buellia.mamillana.1	GCA_964256085.1	Buellia mamillana	Lecanoromycetes	Lichen-forming	N/A	9-21-24	CAXUZP01
buellia.spuria.1	GCA_964256355.1	Buellia spuria	Lecanoromycetes	Lichen-forming	N/A	9-21-24	CAXVAG01
buellia.spuria.2	GCA_964256835.1	Buellia spuria	Lecanoromycetes	Lichen-forming	N/A	9-21-24	CAXVDX01
buellia.stillingiana.1	GCA_964256125.1	Buellia stillingiana	Lecanoromycetes	Lichen-forming	N/A	9-21-24	CAXVAX01
bulbothrix.scortella.1	GCA_964256735.1	Bulbothrix scortella	Lecanoromycetes	Lichen-forming	N/A	9-21-24	CAXVCE01

byssoloma.meadii.1	GCA_964254735.1	Byssoloma meadii	Lecanoromycetes	Lichen-forming	N/A	9-21-24	CAXUUQ01
byssoloma.subdiscordans.1	GCA_964254835.1	Byssoloma subdiscordans	Lecanoromycetes	Lichen-forming	N/A	9-21-24	CAXUUD01
calogaya.arnoldii.1	GCA_051941235.1	Calogaya arnoldii	Lecanoromycetes	Lichen-forming	N/A	8-12-25	JAVEBK01
calogaya.arnoldii.2	GCA_051941725.1	Calogaya arnoldii	Lecanoromycetes	Lichen-forming	N/A	8-12-25	JAVEAF01
caloplaca.aegaea.1	GCA_023646655.1	Caloplaca aegaea	Lecanoromycetes	Lichen-forming	N/A	6-2-22	JALAIP01
caloplaca.aetnensis.1	GCA_023646255.1	Caloplaca aetnensis	Lecanoromycetes	Lichen-forming	N/A	6-2-22	JALAIC01
caloplaca.ligustica.1	GCA_023645885.1	Caloplaca ligustica	Lecanoromycetes	Lichen-forming	N/A	6-2-22	JALAIE01
caloplaca.sp..1	GCA_051941525.1	Caloplaca sp.	Lecanoromycetes	Lichen-forming	N/A	8-12-25	JAVEAR01
caloplaca.sp..2	GCA_051941485.1	Caloplaca sp.	Lecanoromycetes	Lichen-forming	N/A	8-12-25	JAVEAT01
caloplaca.sp..3	GCA_052059075.1	Caloplaca sp.	Lecanoromycetes	Lichen-forming	N/A	8-15-25	JAVEAY01
cetradonia.linearis.1	GCA_003521265.1	Cetradonia linearis	Lecanoromycetes	Lichen-forming	N/A	9-10-18	QKZZ01
cetrelia.chicitae.1	GCA_964257465.1	Cetrelia chicitae	Lecanoromycetes	Lichen-forming	N/A	9-21-24	CAXVFK01
cetrelia.olivetorum.1	GCA_964255315.1	Cetrelia olivetorum	Lecanoromycetes	Lichen-forming	N/A	9-21-24	CAXUWO01
circinaria.calcarea.1	GCA_051942165.1	Circinaria calcarea	Lecanoromycetes	Lichen-forming	N/A	8-12-25	JAVECS01
cladonia.arbuscula.1	GCA_964255635.1	Cladonia arbuscula	Lecanoromycetes	Lichen-forming	N/A	9-21-24	CAXUXX01
cladonia.borealis.1	GCA_018257855.2	Cladonia borealis	Lecanoromycetes	Lichen-forming	N/A	7-12-23	JAFEKC02
cladonia.caroliniana.1	GCA_964256855.1	Cladonia caroliniana	Lecanoromycetes	Lichen-forming	N/A	9-21-24	CAXVCL01
cladonia.coccifera.1	GCA_964254445.1	Cladonia coccifera	Lecanoromycetes	Lichen-forming	N/A	9-21-24	CAXUUS01
cladonia.didyma.1	GCA_964255485.1	Cladonia didyma	Lecanoromycetes	Lichen-forming	N/A	9-21-24	CAXUXM01
cladonia.diversa.1	GCA_964605785.1	Cladonia diversa	Lecanoromycetes	Lichen-forming	N/A	1-2-25	CAZSEB01
cladonia.diversa.2	GCA_964605305.1	Cladonia diversa	Lecanoromycetes	Lichen-forming	N/A	1-2-25	CAZSCN01
cladonia.furcata.1	GCA_964255445.1	Cladonia furcata	Lecanoromycetes	Lichen-forming	N/A	9-21-24	CAXUWC01
cladonia.grayi.1	GCA_964255175.1	Cladonia grayi	Lecanoromycetes	Lichen-forming	N/A	9-21-24	CAXUWB01
cladonia.kurokawae.1	GCA_054654975.1	Cladonia kurokawae	Lecanoromycetes	Lichen-forming	N/A	9-25-25	BAAIEH01
cladonia.leporina.1	GCA_964254565.1	Cladonia leporina	Lecanoromycetes	Lichen-forming	N/A	9-21-24	CAXUUT01
cladonia.macilenta.1	GCA_964254615.1	Cladonia macilenta	Lecanoromycetes	Lichen-forming	N/A	9-21-24	CAXUWP01
cladonia.macilenta.2	GCA_000444155.1	Cladonia macilenta	Lecanoromycetes	Lichen-forming	N/A	8-6-13	AUPP01
cladonia.mateocyatha.1	GCA_964254505.1	Cladonia mateocyatha	Lecanoromycetes	Lichen-forming	N/A	9-21-24	CAXUUM01
cladonia.metacorallifera.1	GCA_000482085.2	Cladonia metacorallifera	Lecanoromycetes	Lichen-forming	N/A	1-7-14	AXCT02
cladonia.ochrochlora.1	GCA_964256405.1	Cladonia ochrochlora	Lecanoromycetes	Lichen-forming	N/A	9-21-24	CAXVBG01
cladonia.petrophila.1	GCA_964256395.1	Cladonia petrophila	Lecanoromycetes	Lichen-forming	N/A	9-21-24	CAXVAT01

cladonia.peziziformis.1	GCA_964256715.1	Cladonia peziziformis	Lecanoromycetes	Lichen-forming	N/A	9-21-24	CAXVCP01
cladonia.polycarpoides.1	GCA_964256725.1	Cladonia polycarpoides	Lecanoromycetes	Lichen-forming	N/A	9-21-24	CAXVDT01
cladonia.portentosa.1	GCA_965153415.1	Cladonia portentosa	Lecanoromycetes	Lichen-forming	N/A	2-26-25	
cladonia.pyxidata.1	GCA_964254885.1	Cladonia pyxidata	Lecanoromycetes	Lichen-forming	N/A	9-21-24	CAXUXL01
cladonia.rangiferina.1	GCA_023065455.1	Cladonia rangiferina	Lecanoromycetes	Lichen-forming	N/A	4-20-22	JAJREH01
cladonia.rangiferina.2	GCA_006146055.1	Cladonia rangiferina	Lecanoromycetes	Lichen-forming	N/A	6-7-19	VALI01
cladonia.rangiferina.3	GCA_964254875.1	Cladonia rangiferina	Lecanoromycetes	Lichen-forming	N/A	9-21-24	CAXUUJ01
cladonia.robbinsii.1	GCA_964255325.1	Cladonia robbinsii	Lecanoromycetes	Lichen-forming	N/A	9-21-24	CAXUXT01
cladonia.sp..1	GCA_937857425.1	Cladonia sp.	Lecanoromycetes	Lichen-forming	N/A	1-12-23	CALHCZ01
cladonia.sp..10	GCA_937857195.1	Cladonia sp.	Lecanoromycetes	Lichen-forming	N/A	1-12-23	CALHCT01
cladonia.sp..11	GCA_937857275.1	Cladonia sp.	Lecanoromycetes	Lichen-forming	N/A	1-12-23	CALAOP01
cladonia.sp..2	GCA_937857405.1	Cladonia sp.	Lecanoromycetes	Lichen-forming	N/A	1-12-23	CALAON01
cladonia.sp..3	GCA_937857105.1	Cladonia sp.	Lecanoromycetes	Lichen-forming	N/A	1-12-23	CAKZWY01
cladonia.sp..4	GCA_937857955.1	Cladonia sp.	Lecanoromycetes	Lichen-forming	N/A	1-12-23	CALEEM01
cladonia.sp..5	GCA_937857855.1	Cladonia sp.	Lecanoromycetes	Lichen-forming	N/A	1-12-23	CALEEO01
cladonia.sp..6	GCA_963971245.1	Cladonia sp.	Lecanoromycetes	Lichen-forming	N/A	3-2-24	
cladonia.sp..7	GCA_963971275.1	Cladonia sp.	Lecanoromycetes	Lichen-forming	N/A	3-4-24	CAXARJ01
cladonia.sp..8	GCA_937857205.1	Cladonia sp.	Lecanoromycetes	Lichen-forming	N/A	1-12-23	CALAOO01
cladonia.sp..9	GCA_937857785.1	Cladonia sp.	Lecanoromycetes	Lichen-forming	N/A	1-12-23	CALHCX01
cladonia.squamosa.1	GCA_947623385.2	Cladonia squamosa	Lecanoromycetes	Lichen-forming	N/A	5-13-23	CANQGY02
cladonia.squamosa.2	GCA_947623575.2	Cladonia squamosa	Lecanoromycetes	Lichen-forming	N/A	5-13-23	CANQGX02
cladonia.squamosa.3	GCA_964256895.1	Cladonia squamosa	Lecanoromycetes	Lichen-forming	N/A	9-21-24	CAXVCN01
cladonia.squamosa.4	GCA_964255015.1	Cladonia squamosa	Lecanoromycetes	Lichen-forming	N/A	9-21-24	CAXUUU01
cladonia.stellaris.1	GCA_040955005.1	Cladonia stellaris	Lecanoromycetes	Lichen-forming	N/A	7-30-24	JBECUK01
cladonia.stellaris.2	GCA_040955015.1	Cladonia stellaris	Lecanoromycetes	Lichen-forming	N/A	7-30-24	JBECUJ01
cladonia.strepsilis.1	GCA_964254655.1	Cladonia strepsilis	Lecanoromycetes	Lichen-forming	N/A	9-21-24	CAXUWX01
cladonia.subtenuis.1	GCA_964256865.1	Cladonia subtenuis	Lecanoromycetes	Lichen-forming	N/A	9-21-24	CAXVCJ01
cladonia.uncialis.1	GCA_977014525.1	Cladonia uncialis	Lecanoromycetes	Lichen-forming	N/A	11-8-25	CDRNKR01
cladonia.uncialis.2	GCA_977014875.1	Cladonia uncialis	Lecanoromycetes	Lichen-forming	N/A	9-21-24	CAXUTX01
cladonia.uncialis.3	GCA_002927785.1	Cladonia uncialis	Lecanoromycetes	Lichen-forming	N/A	2-14-18	NAPT01
cladonia.uncialis.4	GCA_964254785.1	Cladonia uncialis	Lecanoromycetes	Lichen-forming	N/A	9-21-24	CAXUUO01

coccocarpia.palmicola.1	GCA_964256785.1	Coccocarpia palmicola	Lecanoromycetes	Lichen-forming	N/A	9-21-24	CAXVCG01
collema.furfuraceum.1	GCA_964256385.1	Collema furfuraceum	Lecanoromycetes	Lichen-forming	N/A	9-21-24	CAXVBI01
cornicularia.normoerica.1	GCA_964254975.1	Cornicularia normoerica	Lecanoromycetes	Lichen-forming	N/A	9-21-24	CAXUXC01
cornicularia.normoerica.2	GCA_051942105.1	Cornicularia normoerica	Lecanoromycetes	Lichen-forming	N/A	8-12-25	JAVECU01
crespoa.crozalsiana.1	GCA_964256695.1	Crespoa crozalsiana	Lecanoromycetes	Lichen-forming	N/A	9-21-24	CAXVCF01
cyanodermella.asteris.1	GCA_900618795.1	Cyanodermella asteris	Lecanoromycetes	Endophyte	N/A	12-5-18	UYJD01
dendriscocaulon.intricatulum.1	GCA_964255535.1	Dendriscocaulon intricatulum	Lecanoromycetes	Lichen-forming	N/A	9-21-24	CAXUXZ01
dermiscellum.oulocheilum.1	GCA_964257525.1	Dermiscellum oulocheilum	Lecanoromycetes	Lichen-forming	N/A	9-21-24	CAXVFH01
dibaeis.baeomyces.1	GCA_964254685.1	Dibaeis baeomyces	Lecanoromycetes	Lichen-forming	N/A	9-21-24	CAXUTT01
dibaeis.sorediata.1	GCA_964255195.1	Dibaeis sorediata	Lecanoromycetes	Lichen-forming	N/A	9-21-24	CAXUVO01
dimelaena.oreina.1	GCA_964257695.1	Dimelaena oreina	Lecanoromycetes	Lichen-forming	N/A	9-21-24	CAXVFS01
diploschistes.diacapsis.1	GCA_023645945.1	Diploschistes diacapsis	Lecanoromycetes	Lichen-forming	N/A	6-2-22	JALAIB01
dirinaria.frostii.1	GCA_964257345.1	Dirinaria frostii	Lecanoromycetes	Lichen-forming	N/A	9-21-24	CAXVFC01
dirinaria.sp..1	GCA_051941275.1	Dirinaria sp.	Lecanoromycetes	Lichen-forming	N/A	8-12-25	JAVEBF01
dirinaria.sp..2	GCA_013315955.1	Dirinaria sp.	Lecanoromycetes	Lichen-forming	N/A	6-10-20	SZQE01
evernia.divaricata.1	GCA_964255455.1	Evernia divaricata	Lecanoromycetes	Lichen-forming	N/A	9-21-24	CAXUUW01
evernia.prunastri.1	GCA_003184365.1	Evernia prunastri	Lecanoromycetes	Lichen-forming	N/A	6-4-18	NKYR01
evernia.prunastri.2	GCA_964255555.1	Evernia prunastri	Lecanoromycetes	Lichen-forming	N/A	9-21-24	CAXUXW01
everniopsis.trulla.1	GCA_964257165.1	Everniopsis trulla	Lecanoromycetes	Lichen-forming	N/A	9-21-24	CAXVDL01
fissurina.insidiosa.1	GCA_964256455.1	Fissurina insidiosa	Lecanoromycetes	Lichen-forming	N/A	9-21-24	CAXVBW01
flavocetraria.cucullata.1	GCA_964257225.1	Flavocetraria cucullata	Lecanoromycetes	Lichen-forming	N/A	9-21-24	CAXVDQ01
flavoparmelia.baltimorensis.1	GCA_964254775.1	Flavoparmelia baltimorensis	Lecanoromycetes	Lichen-forming	N/A	9-21-24	CAXUWQ01
flavoplaca.austrocitrina.1	GCA_051942045.1	Flavoplaca austrocitrina	Lecanoromycetes	Lichen-forming	N/A	8-12-25	JAVEBJ01
flavoplaca.citrina.1	GCA_051941225.1	Flavoplaca citrina	Lecanoromycetes	Lichen-forming	N/A	8-12-25	JAVEBH01
flavoplaca.flavocitrina.1	GCA_051942005.1	Flavoplaca flavocitrina	Lecanoromycetes	Lichen-forming	N/A	8-12-25	JAVEBO01
flavoplaca.limonia.1	GCA_051942465.1	Flavoplaca limonia	Lecanoromycetes	Lichen-forming	N/A	8-12-25	JAVEBZ01
flavoplaca.marina.1	GCA_965225635.1	Flavoplaca marina	Lecanoromycetes	Lichen-forming	N/A	3-28-25	CBDDCU01
flavoplaca.marina.2	GCA_965226325.1	Flavoplaca marina	Lecanoromycetes	Lichen-forming	N/A	3-28-25	CBDDFF01
flavoplaca.navasiana.1	GCA_051941985.1	Flavoplaca navasiana	Lecanoromycetes	Lichen-forming	N/A	8-12-25	JAVEBN01
flavoplaca.navasiana.2	GCA_051942405.1	Flavoplaca navasiana	Lecanoromycetes	Lichen-forming	N/A	8-12-25	JAVECD01

flavoplaca.oasis.1	GCA_023646635.1	Flavoplaca oasis	Lecanoromycetes	Lichen-forming	N/A	6-2-22	JALAIS01
flavoplaca.sp..1	GCA_052059015.1	Flavoplaca sp.	Lecanoromycetes	Lichen-forming	N/A	8-15-25	JAVEBI01
flavopunctelia.flaventior.1	GCA_964257575.1	Flavopunctelia flaventior	Lecanoromycetes	Lichen-forming	N/A	9-21-24	CAXVGB01
fulgogasparrea.decipioides.1	GCA_051941625.1	Fulgogasparrea decipioides	Lecanoromycetes	Lichen-forming	N/A	8-12-25	JAVEAO01
fuscopannaria.leucosticta.1	GCA_964256325.1	Fuscopannaria leucosticta	Lecanoromycetes	Lichen-forming	N/A	9-21-24	CAXVAS01
gallowayella.concinna.1	GCA_051941645.1	Gallowayella concinna	Lecanoromycetes	Lichen-forming	N/A	8-12-25	JAVEAL01
gallowayella.fulva.1	GCA_023646365.1	Gallowayella fulva	Lecanoromycetes	Lichen-forming	N/A	6-2-22	JALAIN01
gallowayella.weberi.1	GCA_051941305.1	Gallowayella weberi	Lecanoromycetes	Lichen-forming	N/A	8-12-25	JAVEBD01
gallowayella.weberi.2	GCA_051941295.1	Gallowayella weberi	Lecanoromycetes	Lichen-forming	N/A	8-12-25	JAVEBE01
gomphillus.americanus.1	GCA_905337335.1	Gomphillus americanus	Lecanoromycetes	Lichen-forming	N/A	4-26-18	PEKF01
gomphillus.americanus.2	GCA_964255845.1	Gomphillus americanus	Lecanoromycetes	Lichen-forming	N/A	9-21-24	CAXUZW01
gomphillus.calycioides.1	GCA_964255765.1	Gomphillus calycioides	Lecanoromycetes	Lichen-forming	N/A	9-21-24	CAXUYI01
gyalecta.farlowii.1	GCA_964256515.1	Gyalecta farlowii	Lecanoromycetes	Lichen-forming	N/A	9-21-24	CAXVAY01
gyalolechia.ehrenbergii.1	GCA_023646125.1	Gyalolechia ehrenbergii	Lecanoromycetes	Lichen-forming	N/A	6-2-22	JALAIJ01
gyalolechia.fulgens.1	GCA_051942345.1	Gyalolechia fulgens	Lecanoromycetes	Lichen-forming	N/A	8-12-25	JAVECG01
gyalolechia.gomerana.1	GCA_051942385.1	Gyalolechia gomerana	Lecanoromycetes	Lichen-forming	N/A	8-12-25	JAVECC01
gyalolechia.marmorata.1	GCA_051941165.1	Gyalolechia marmorata	Lecanoromycetes	Lichen-forming	N/A	8-12-25	JAVEBL01
gyalolechia.sp..1	GCA_051941825.1	Gyalolechia sp.	Lecanoromycetes	Lichen-forming	N/A	8-12-25	JAVEBU01
gyalolechia.sp..2	GCA_051941865.1	Gyalolechia sp.	Lecanoromycetes	Lichen-forming	N/A	8-12-25	JAVEBT01
gypsoplaca.macrophylla.1	GCA_964255185.1	Gypsoplaca macrophylla	Lecanoromycetes	Lichen-forming	N/A	9-21-24	CAXUTV01
haloplaca.sp..1	GCA_051942025.1	Haloplaca sp.	Lecanoromycetes	Lichen-forming	N/A	8-12-25	JAVEBM01
haloplaca.sp..2	GCA_051941845.1	Haloplaca sp.	Lecanoromycetes	Lichen-forming	N/A	8-12-25	JAVEBV01
heterodermia.albicans.1	GCA_964256065.1	Heterodermia albicans	Lecanoromycetes	Lichen-forming	N/A	9-21-24	CAXUZU01
heterodermia.echinata.1	GCA_964255435.1	Heterodermia echinata	Lecanoromycetes	Lichen-forming	N/A	9-21-24	CAXUWH01
heterodermia.hypoleuca.1	GCA_964254765.1	Heterodermia hypoleuca	Lecanoromycetes	Lichen-forming	N/A	9-21-24	CAXUVN01
heterodermia.langdoniana.1	GCA_964254605.1	Heterodermia langdoniana	Lecanoromycetes	Lichen-forming	N/A	9-21-24	CAXUVR01
heterodermia.neglecta.1	GCA_964257475.1	Heterodermia neglecta	Lecanoromycetes	Lichen-forming	N/A	9-21-24	CAXVFO01
heterodermia.obscurata.1	GCA_051941365.1	Heterodermia obscurata	Lecanoromycetes	Lichen-forming	N/A	8-12-25	JAVEBB01
heterodermia.sp..1	GCA_937857265.1	Heterodermia sp.	Lecanoromycetes	Lichen-forming	N/A	1-12-23	CALHCU01
heterodermia.sp..2	GCA_937857845.1	Heterodermia sp.	Lecanoromycetes	Lichen-forming	N/A	1-12-23	CALEEL01

heterodermia.sp..3	GCA_937858105.1	Heterodermia sp.	Lecanoromycetes	Lichen-forming	N/A	1-12-23	CALHCR01
heterodermia.speciosa.1	GCA_964255885.1	Heterodermia speciosa	Lecanoromycetes	Lichen-forming	N/A	9-21-24	CAXUZH01
heterodermia.speciosa.2	GCA_905337345.1	Heterodermia speciosa	Lecanoromycetes	Lichen-forming	N/A	3-26-21	CAJPDS01
heterodermia.speciosa.3	GCA_937857615.1	Heterodermia speciosa	Lecanoromycetes	Lichen-forming	N/A	1-12-23	CAKZXC01
hypocenomyce.scalaris.1	GCA_964256115.1	Hypocenomyce scalaris	Lecanoromycetes	Lichen-forming	N/A	9-21-24	CAXVAN01
hypocenomyce.scalaris.2	GCA_022814315.1	Hypocenomyce scalaris	Lecanoromycetes	Lichen-forming	N/A	4-4-22	JALDZC01
hypocenomyce.scalaris.3	GCA_964255775.1	Hypocenomyce scalaris	Lecanoromycetes	Lichen-forming	N/A	9-21-24	CAXUYR01
hypogymnia.physodes.1	GCA_964254535.1	Hypogymnia physodes	Lecanoromycetes	Lichen-forming	N/A	9-21-24	CAXUUK01
hypogymnia.vittata.1	GCA_964257435.1	Hypogymnia vittata	Lecanoromycetes	Lichen-forming	N/A	9-21-24	CAXVFF01
hypotrachyna.appalachensis.1	GCA_964256775.1	Hypotrachyna appalachensis	Lecanoromycetes	Lichen-forming	N/A	9-21-24	CAXVCY01
hypotrachyna.catawbiensis.1	GCA_964257295.1	Hypotrachyna catawbiensis	Lecanoromycetes	Lichen-forming	N/A	9-21-24	CAXVFA01
hypotrachyna.lividescens.1	GCA_964255505.1	Hypotrachyna lividescens	Lecanoromycetes	Lichen-forming	N/A	9-21-24	CAXUXH01
hypotrachyna.osseoalba.1	GCA_964256905.1	Hypotrachyna osseoalba	Lecanoromycetes	Lichen-forming	N/A	9-21-24	CAXVDA01
icmadophila.ericetorum.1	GCA_022814295.1	Icmadophila ericetorum	Lecanoromycetes	Lichen-forming	N/A	4-4-22	JALDZD01
icmadophila.ericetorum.2	GCA_937857975.1	Icmadophila ericetorum	Lecanoromycetes	Lichen-forming	N/A	1-12-23	CALFGM01
ikaeria.aurantiellina.1	GCA_051941805.1	Ikaeria aurantiellina	Lecanoromycetes	Lichen-forming	N/A	8-12-25	JAVEBW01
imshaugia.aleurites.1	GCA_905337355.1	Imshaugia aleurites	Lecanoromycetes	Lichen-forming	N/A	3-25-21	CAJPDT01
imshaugia.aleurites.2	GCA_964255935.1	Imshaugia aleurites	Lecanoromycetes	Lichen-forming	N/A	9-21-24	CAXUZY01
imshaugia.aleurites.3	GCA_937858075.1	Imshaugia aleurites	Lecanoromycetes	Lichen-forming	N/A	1-12-23	CALCMY01
ionaspis.alba.1	GCA_964255285.1	Ionaspis alba	Lecanoromycetes	Lichen-forming	N/A	9-21-24	CAXUUL01
ionaspis.lacustris.1	GCA_964257415.1	Ionaspis lacustris	Lecanoromycetes	Lichen-forming	N/A	9-21-24	CAXVEU01
kaernefeltia.merrillii.1	GCA_964257335.1	Kaernefeltia merrillii	Lecanoromycetes	Lichen-forming	N/A	9-21-24	CAXVEG01
kuettlingeria.erythrocarpa.1	GCA_051941955.1	Kuettlingeria erythrocarpa	Lecanoromycetes	Lichen-forming	N/A	8-12-25	JAVEBP01
lambiella.insularis.1	GCA_022814265.1	Lambiella insularis	Lecanoromycetes	Lichen-forming	N/A	3-16-20	SAUG01
lasallia.papulosa.1	GCA_964254525.1	Lasallia papulosa	Lecanoromycetes	Lichen-forming	N/A	9-21-24	CAXUVG01
lasallia.pustulata.1	GCA_008636195.1	Lasallia pustulata	Lecanoromycetes	Lichen-forming	N/A	9-23-19	VXIT01
lasallia.pustulata.2	GCA_023184575.1	Lasallia pustulata	Lecanoromycetes	Lichen-forming	N/A	4-29-22	JALILU01
lasallia.pustulata.3	GCA_053477765.1	Lasallia pustulata	Lecanoromycetes	Lichen-forming	N/A	11-14-25	JBNORX01
lasallia.pustulata.4	GCA_053477785.1	Lasallia pustulata	Lecanoromycetes	Lichen-forming	N/A	11-14-25	JBNORW01
lasallia.pustulata.5	GCA_900169345.1	Lasallia pustulata	Lecanoromycetes	Lichen-forming	N/A	4-14-17	FWEW01

lasallia.pustulata.6	GCA_964255565.1	Lasallia pustulata	Lecanoromycetes	Lichen-forming	N/A	9-21-24	CAXUXG01
lasallia.pustulata.7	GCA_000938525.1	Lasallia pustulata	Lecanoromycetes	Lichen-forming	N/A	3-3-15	JYIL01
lecanora.albella.1	GCA_964254485.1	Lecanora albella	Lecanoromycetes	Lichen-forming	N/A	9-21-24	CAXUUR01
lecanora.atra.1	GCA_964256165.1	Lecanora atra	Lecanoromycetes	Lichen-forming	N/A	9-21-24	CAXVAF01
lecanora.helva.1	GCA_050886345.1	Lecanora helva	Lecanoromycetes	Lichen-forming	N/A	6-11-25	JBKOTY01
lecanora.hybocarpa.1	GCA_964256265.1	Lecanora hybocarpa	Lecanoromycetes	Lichen-forming	N/A	9-21-24	CAXVAR01
lecanora.markjohnstonii.1	GCA_964254635.1	Lecanora markjohnstonii	Lecanoromycetes	Lichen-forming	N/A	9-21-24	CAXUXP01
lecanora.masana.1	GCA_964256795.1	Lecanora masana	Lecanoromycetes	Lichen-forming	N/A	9-21-24	CAXVCB01
lecanora.oreinoides.1	GCA_964255375.1	Lecanora oreinoides	Lecanoromycetes	Lichen-forming	N/A	9-21-24	CAXUVL01
lecanora.rugosella.1	GCA_964255525.1	Lecanora rugosella	Lecanoromycetes	Lichen-forming	N/A	9-21-24	CAXUXQ01
lecanora.strobilina.1	GCA_964254475.1	Lecanora strobilina	Lecanoromycetes	Lichen-forming	N/A	9-21-24	CAXUUN01
lecanora.subimmergens.1	GCA_964256505.1	Lecanora subimmergens	Lecanoromycetes	Lichen-forming	N/A	9-21-24	CAXVBA01
lecanorales.sp..1	GCA_964254455.1	Lecanorales sp.	Lecanoromycetes	Lichen-forming	N/A	9-21-24	CAXUVZ01
lecanorales.sp..2	GCA_964254845.1	Lecanorales sp.	Lecanoromycetes	Lichen-forming	N/A	9-21-24	CAXUVI01
lecanorales.sp..3	GCA_964255655.1	Lecanorales sp.	Lecanoromycetes	Lichen-forming	N/A	9-21-24	CAXUYJ01
lecanorales.sp..4	GCA_964256805.1	Lecanorales sp.	Lecanoromycetes	Lichen-forming	N/A	9-21-24	CAXVCM01
lecanorales.sp..5	GCA_964255755.1	Lecanorales sp.	Lecanoromycetes	Lichen-forming	N/A	9-21-24	CAXUYL01
lecanorales.sp..6	GCA_964257705.1	Lecanorales sp.	Lecanoromycetes	Lichen-forming	N/A	9-21-24	CAXVFZ01
lecanoromycetes.sp..1	GCA_937858085.1	Lecanoromycetes sp.	Lecanoromycetes	Lichen-forming	N/A	1-12-23	CALHCS01
lecanoromycetes.sp..2	GCA_964254985.1	Lecanoromycetes sp.	Lecanoromycetes	Lichen-forming	N/A	9-21-24	CAXUWR01
lecanoromycetes.sp..3	GCA_937857585.1	Lecanoromycetes sp.	Lecanoromycetes	Lichen-forming	N/A	1-12-23	CALBWY01
lecanoromycetes.sp..4	GCA_964256955.1	Lecanoromycetes sp.	Lecanoromycetes	Lichen-forming	N/A	9-21-24	CAXVCS01
lecanoromycetes.sp..5	GCA_937857895.1	Lecanoromycetes sp.	Lecanoromycetes	Lichen-forming	N/A	1-12-23	CALEEP01
lecanoromycetidae.sp..1	GCA_035521555.1	Lecanoromycetidae sp.	Lecanoromycetes	Lichen-forming	N/A	1-10-24	JASZXT01
lecidea.sp..1	GCA_964254725.1	Lecidea sp.	Lecanoromycetes	Lichen-forming	N/A	9-21-24	CAXUVV01
lecidea.tessellata.1	GCA_964256205.1	Lecidea tessellata	Lecanoromycetes	Lichen-forming	N/A	9-21-24	CAXVAI01
lecidella.sp..1	GCA_964255055.1	Lecidella sp.	Lecanoromycetes	Lichen-forming	N/A	9-21-24	CAXUUV01
lepra.amara.1	GCA_964255245.1	Lepra amara	Lecanoromycetes	Lichen-forming	N/A	9-21-24	CAXUXJ01
lepraria.finkii.1	GCA_042464665.1	Lepraria finkii	Lecanoromycetes	Lichen-forming	N/A	9-30-24	JBHFEH01
lepraria.leprolomopsis.1	GCA_964254625.1	Lepraria leprolomopsis	Lecanoromycetes	Lichen-forming	N/A	9-21-24	CAXUWK01
lepraria.neglecta.1	GCA_033220425.1	Lepraria neglecta	Lecanoromycetes	Lichen-forming	N/A	11-3-23	JASNWA01

lepraria.oxybapha.1	GCA_964257485.1	Lepraria oxybapha	Lecanoromycetes	Lichen-forming	N/A	9-21-24	CAXVFY01
lepraria.sp..1	GCA_937857285.1	Lepraria sp.	Lecanoromycetes	Lichen-forming	N/A	1-12-23	CALCMZ01
leprocaulales.sp..1	GCA_964255695.1	Leprocaulales sp.	Lecanoromycetes	Lichen-forming	N/A	9-21-24	CAXUYV01
leprocaulon.nicholsiae.1	GCA_964256825.1	Leprocaulon nicholsiae	Lecanoromycetes	Lichen-forming	N/A	9-21-24	CAXVDB01
leptogium.austroamericanum.1	GCA_964255575.1	Leptogium austroamericanum	Lecanoromycetes	Lichen-forming	N/A	9-21-24	CAXUYE01
leptogium.austroamericanum.2	GCA_964255685.1	Leptogium austroamericanum	Lecanoromycetes	Lichen-forming	N/A	9-21-24	CAXUZK01
leptogium.chloromelum.1	GCA_964254935.1	Leptogium chloromelum	Lecanoromycetes	Lichen-forming	N/A	9-21-24	CAXUXI01
leptogium.corticola.1	GCA_964256315.1	Leptogium corticola	Lecanoromycetes	Lichen-forming	N/A	9-21-24	CAXVBD01
letharia.columbiana.1	GCA_014066305.1	Letharia columbiana	Lecanoromycetes	Lichen-forming	N/A	8-5-20	JACCJC01
letharia.lupina.1	GCA_014066315.1	Letharia lupina	Lecanoromycetes	Lichen-forming	N/A	8-6-20	JACCJB01
letharia.vulpina.1	GCA_964257005.1	Letharia vulpina	Lecanoromycetes	Lichen-forming	N/A	9-21-24	CAXVCQ01
letrouitia.sp..1	GCA_051942305.1	Letrouitia sp.	Lecanoromycetes	Lichen-forming	N/A	8-12-25	JAVECH01
letrouitia.subvulpina.1	GCA_052059055.1	Letrouitia subvulpina	Lecanoromycetes	Lichen-forming	N/A	8-15-25	JAVECI01
letrouitia.transgressa.1	GCA_023646045.1	Letrouitia transgressa	Lecanoromycetes	Lichen-forming	N/A	6-2-22	JALAIO01
letrouitia.transgressa.2	GCA_023645855.1	Letrouitia transgressa	Lecanoromycetes	Lichen-forming	N/A	6-2-22	JALAIG01
leucodecton.sp..1	GCA_964257275.1	Leucodecton sp.	Lecanoromycetes	Lichen-forming	N/A	9-21-24	CAXVEL01
leucodermia.appalachiensis.1	GCA_964255645.1	Leucodermia appalachiensis	Lecanoromycetes	Lichen-forming	N/A	9-21-24	CAXUYC01
leucodermia.leucomelos.1	GCA_964257595.1	Leucodermia leucomelos	Lecanoromycetes	Lichen-forming	N/A	9-21-24	CAXVGA01
lignoscripta.atroalba.1	GCA_022814235.1	Lignoscripta atroalba	Lecanoromycetes	Lichen-forming	N/A	4-4-22	JALDZG01
lignoscripta.atroalba.2	GCA_964256495.1	Lignoscripta atroalba	Lecanoromycetes	Lichen-forming	N/A	9-21-24	CAXVAJ01
lobaria.immixta.1	GCA_022814215.1	Lobaria immixta	Lecanoromycetes	Lichen-forming	N/A	4-4-22	JALDZH01
lobaria.pulmonaria.1	GCA_964257085.1	Lobaria pulmonaria	Lecanoromycetes	Lichen-forming	N/A	9-21-24	CAXVDS01
loekoesia.sp..1	GCA_051941565.1	Loekoesia sp.	Lecanoromycetes	Lichen-forming	N/A	8-12-25	JAVEAQ01
loxospora.cismonica.1	GCA_964256565.1	Loxospora cismonica	Lecanoromycetes	Lichen-forming	N/A	9-21-24	CAXVAP01
loxospora.ochrophaea.1	GCA_022814175.1	Loxospora ochrophaea	Lecanoromycetes	Lichen-forming	N/A	4-4-22	JALDZI01
massalongia.carnosa.1	GCA_965645245.1	Massalongia carnosa	Lecanoromycetes	Lichen-forming	N/A	7-28-25	CBFFUZ01
melanelia.culbersonii.1	GCA_964255045.1	Melanelia culbersonii	Lecanoromycetes	Lichen-forming	N/A	9-21-24	CAXUVT01
melanohalea.halei.1	GCA_964256215.1	Melanohalea halei	Lecanoromycetes	Lichen-forming	N/A	9-21-24	CAXVAM01
micareopsis.irriguata.1	GCA_964256875.1	Micareopsis irriguata	Lecanoromycetes	Lichen-forming	N/A	9-21-24	CAXVCO01
mobergia.calculiformis.1	GCA_964255985.1	Mobergia calculiformis	Lecanoromycetes	Lichen-forming	N/A	9-21-24	CAXUZB01

mycoblastus.sanguinarius.1	GCA_022814195.1	Mycoblastus sanguinarius	Lecanoromycetes	Lichen-forming	N/A	4-4-22	JALDZJ01
mycoblastus.sanguinarius.2	GCA_964256545.1	Mycoblastus sanguinarius	Lecanoromycetes	Lichen-forming	N/A	9-21-24	CAXVBO01
nadvornikia.sorediata.1	GCA_964256945.1	Nadvornikia sorediata	Lecanoromycetes	Lichen-forming	N/A	9-21-24	CAXVDI01
nephroma.helveticum.1	GCA_964254995.1	Nephroma helveticum	Lecanoromycetes	Lichen-forming	N/A	9-21-24	CAXUWS01
niebla.homalea.1	GCA_019925105.1	Niebla homalea	Lecanoromycetes	Lichen-forming	N/A	9-10-21	JAHGAU01
obscuroplaca.camptidia.1	GCA_964255815.1	Obscuroplaca camptidia	Lecanoromycetes	Lichen-forming	N/A	9-21-24	CAXUYU01
ochrolechia.arborea.1	GCA_964255665.1	Ochrolechia arborea	Lecanoromycetes	Lichen-forming	N/A	9-21-24	CAXUYB01
ochrolechia.trochophora.1	GCA_964257425.1	Ochrolechia trochophora	Lecanoromycetes	Lichen-forming	N/A	9-21-24	CAXVEZ01
opeltia.flavorubescens.1	GCA_000442125.1	Opeltia flavorubescens	Lecanoromycetes	Lichen-forming	N/A	7-30-13	AUPK01
oxnerella.safavidiorum.1	GCA_964257185.1	Oxnerella safavidiorum	Lecanoromycetes	Lichen-forming	N/A	9-21-24	CAXVEV01
pannaria.tavaresii.1	GCA_964257215.1	Pannaria tavaresii	Lecanoromycetes	Lichen-forming	N/A	9-21-24	CAXVFB01
parmelia.sp..1	GCA_018257885.1	Parmelia sp.	Lecanoromycetes	Lichen-forming	N/A	5-4-21	JAEUBB01
parmelia.squarrosa.1	GCA_964255235.1	Parmelia squarrosa	Lecanoromycetes	Lichen-forming	N/A	9-21-24	CAXUWY01
parmeliaceae.sp..1	GCA_937857515.1	Parmeliaceae sp.	Lecanoromycetes	Lichen-forming	N/A	1-12-23	CALHCY01
parmeliaceae.sp..10	GCA_937857575.1	Parmeliaceae sp.	Lecanoromycetes	Lichen-forming	N/A	1-12-23	CALCMX01
parmeliaceae.sp..11	GCA_937857215.1	Parmeliaceae sp.	Lecanoromycetes	Lichen-forming	N/A	1-12-23	CALHCV01
parmeliaceae.sp..12	GCA_937857935.1	Parmeliaceae sp.	Lecanoromycetes	Lichen-forming	N/A	1-12-23	CALEES01
parmeliaceae.sp..13	GCA_937854535.1	Parmeliaceae sp.	Lecanoromycetes	Lichen-forming	N/A	1-12-23	CALHDA01
parmeliaceae.sp..14	GCA_937857565.1	Parmeliaceae sp.	Lecanoromycetes	Lichen-forming	N/A	1-12-23	CALBEM01
parmeliaceae.sp..15	GCA_937832375.1	Parmeliaceae sp.	Lecanoromycetes	Lichen-forming	N/A	1-12-23	CALFGJ01
parmeliaceae.sp..16	GCA_937836395.1	Parmeliaceae sp.	Lecanoromycetes	Lichen-forming	N/A	1-12-23	CALEEH01
parmeliaceae.sp..17	GCA_964255415.1	Parmeliaceae sp.	Lecanoromycetes	Lichen-forming	N/A	9-21-24	CAXUUI01
parmeliaceae.sp..18	GCA_964254515.1	Parmeliaceae sp.	Lecanoromycetes	Lichen-forming	N/A	9-21-24	CAXUTS01
parmeliaceae.sp..19	GCA_937840615.1	Parmeliaceae sp.	Lecanoromycetes	Lichen-forming	N/A	1-12-23	CALGBA01
parmeliaceae.sp..2	GCA_937836435.1	Parmeliaceae sp.	Lecanoromycetes	Lichen-forming	N/A	1-12-23	CALEEF01
parmeliaceae.sp..20	GCA_937822845.1	Parmeliaceae sp.	Lecanoromycetes	Lichen-forming	N/A	1-12-23	CALEED01
parmeliaceae.sp..21	GCA_937857985.1	Parmeliaceae sp.	Lecanoromycetes	Lichen-forming	N/A	1-12-23	CALFGL01
parmeliaceae.sp..22	GCA_937822555.1	Parmeliaceae sp.	Lecanoromycetes	Lichen-forming	N/A	1-12-23	CALGAY01
parmeliaceae.sp..23	GCA_937840635.1	Parmeliaceae sp.	Lecanoromycetes	Lichen-forming	N/A	1-12-23	CALGAZ01
parmeliaceae.sp..3	GCA_937858145.1	Parmeliaceae sp.	Lecanoromycetes	Lichen-forming	N/A	1-12-23	CALFGR01
parmeliaceae.sp..4	GCA_964257545.1	Parmeliaceae sp.	Lecanoromycetes	Lichen-forming	N/A	9-21-24	CAXVGG01

parmeliaceae.sp..5	GCA_937857945.1	Parmeliaceae sp.	Lecanoromycetes	Lichen-forming	N/A	1-12-23	CALFGK01
parmeliaceae.sp..6	GCA_937822895.1	Parmeliaceae sp.	Lecanoromycetes	Lichen-forming	N/A	1-12-23	CALGAX01
parmeliaceae.sp..7	GCA_937857925.1	Parmeliaceae sp.	Lecanoromycetes	Lichen-forming	N/A	1-12-23	CALEEN01
parmeliaceae.sp..8	GCA_964256295.1	Parmeliaceae sp.	Lecanoromycetes	Lichen-forming	N/A	9-21-24	CAXVBE01
parmeliaceae.sp..9	GCA_937849145.1	Parmeliaceae sp.	Lecanoromycetes	Lichen-forming	N/A	1-12-23	CALGBD01
parmotrema.austrosinense.1	GCA_964256935.1	Parmotrema austrosinense	Lecanoromycetes	Lichen-forming	N/A	9-21-24	CAXVDR01
parmotrema.cetratum.1	GCA_964257665.1	Parmotrema cetratum	Lecanoromycetes	Lichen-forming	N/A	9-21-24	CAXVGF01
parmotrema.diffractaicum.1	GCA_964255725.1	Parmotrema diffractaicum	Lecanoromycetes	Lichen-forming	N/A	9-21-24	CAXUYQ01
parmotrema.hypotropum.1	GCA_964254755.1	Parmotrema hypotropum	Lecanoromycetes	Lichen-forming	N/A	9-21-24	CAXUWD01
parmotrema.internexum.1	GCA_964255545.1	Parmotrema internexum	Lecanoromycetes	Lichen-forming	N/A	9-21-24	CAXUXY01
parmotrema.mellissii.1	GCA_964255255.1	Parmotrema mellissii	Lecanoromycetes	Lichen-forming	N/A	9-21-24	CAXUXR01
parmotrema.neotropicum.1	GCA_964257615.1	Parmotrema neotropicum	Lecanoromycetes	Lichen-forming	N/A	9-21-24	CAXVFT01
parmotrema.perlatum.1	GCA_964257535.1	Parmotrema perlatum	Lecanoromycetes	Lichen-forming	N/A	9-21-24	CAXVFX01
parmotrema.rampoddense.1	GCA_964254915.1	Parmotrema rampoddense	Lecanoromycetes	Lichen-forming	N/A	9-21-24	CAXUWF01
parmotrema.reticulatum.1	GCA_964257175.1	Parmotrema reticulatum	Lecanoromycetes	Lichen-forming	N/A	9-21-24	CAXVEN01
parmotrema.stuppeum.1	GCA_964255715.1	Parmotrema stuppeum	Lecanoromycetes	Lichen-forming	N/A	9-21-24	CAXUYO01
parmotrema.submarginale.1	GCA_964257635.1	Parmotrema submarginale	Lecanoromycetes	Lichen-forming	N/A	9-21-24	CAXVFW01
parmotrema.tinctorum.1	GCA_964257305.1	Parmotrema tinctorum	Lecanoromycetes	Lichen-forming	N/A	9-21-24	CAXVEX01
parmotrema.ultralucens.1	GCA_964257235.1	Parmotrema ultralucens	Lecanoromycetes	Lichen-forming	N/A	9-21-24	CAXVEO01
parmotrema.xanthinum.1	GCA_964256175.1	Parmotrema xanthinum	Lecanoromycetes	Lichen-forming	N/A	9-21-24	CAXVAD01
pectenia.cyanoloma.1	GCA_964254465.1	Pectenia cyanoloma	Lecanoromycetes	Lichen-forming	N/A	9-21-24	CAXUUA01
pectenia.plumbea.1	GCA_964255295.1	Pectenia plumbea	Lecanoromycetes	Lichen-forming	N/A	9-21-24	CAXUUB01
peltigera.aphthosa.1	GCA_964257115.1	Peltigera aphthosa	Lecanoromycetes	Lichen-forming	N/A	9-21-24	CAXVES01
peltigera.dolichorrhiza.1	GCA_964257155.1	Peltigera dolichorrhiza	Lecanoromycetes	Lichen-forming	N/A	9-21-24	CAXVEE01
peltigera.evansiana.1	GCA_964256605.1	Peltigera evansiana	Lecanoromycetes	Lichen-forming	N/A	9-21-24	CAXVBZ01
peltigera.extenuata.1	GCA_964255975.1	Peltigera extenuata	Lecanoromycetes	Lichen-forming	N/A	9-21-24	CAXUZV01
peltigera.hydrothyria.1	GCA_964256015.1	Peltigera hydrothyria	Lecanoromycetes	Lichen-forming	N/A	9-21-24	CAXVAL01
peltigera.leucophlebia.1	GCA_022814155.1	Peltigera leucophlebia	Lecanoromycetes	Lichen-forming	N/A	4-4-22	JALDZK01
peltigera.phyllidiosa.1	GCA_964256585.1	Peltigera phyllidiosa	Lecanoromycetes	Lichen-forming	N/A	9-21-24	CAXVCI01
pertusaria.andersoniae.1	GCA_964254805.1	Pertusaria andersoniae	Lecanoromycetes	Lichen-forming	N/A	9-21-24	CAXUVW01

pertusaria.epixantha.1	GCA_964254645.1	Pertusaria epixantha	Lecanoromycetes	Lichen-forming	N/A	9-21-24	CAXUVB01
pertusaria.obruta.1	GCA_964256965.1	Pertusaria obruta	Lecanoromycetes	Lichen-forming	N/A	9-21-24	CAXVDG01
pertusaria.ostiolata.1	GCA_964255625.1	Pertusaria ostiolata	Lecanoromycetes	Lichen-forming	N/A	9-21-24	CAXUYD01
pertusaria.plittiana.1	GCA_964256485.1	Pertusaria plittiana	Lecanoromycetes	Lichen-forming	N/A	9-21-24	CAXVBQ01
pertusaria.propinqua.1	GCA_964255355.1	Pertusaria propinqua	Lecanoromycetes	Lichen-forming	N/A	9-21-24	CAXUVU01
pertusariales.sp..1	GCA_964254795.1	Pertusariales sp.	Lecanoromycetes	Lichen-forming	N/A	9-21-24	CAXUUP01
phaeophyscia.hispidula.1	GCA_964257555.1	Phaeophyscia hispidula	Lecanoromycetes	Lichen-forming	N/A	9-21-24	CAXVFI01
phaeophyscia.rubropulchra.1	GCA_964256345.1	Phaeophyscia rubropulchra	Lecanoromycetes	Lichen-forming	N/A	9-21-24	CAXVAW01
phaeophyscia.squarrosa.1	GCA_964255615.1	Phaeophyscia squarrosa	Lecanoromycetes	Lichen-forming	N/A	9-21-24	CAXUXV01
phlyctis.boliviensis.1	GCA_964256025.1	Phlyctis boliviensis	Lecanoromycetes	Lichen-forming	N/A	9-21-24	CAXUZZ01
phlyctis.petraea.1	GCA_964256925.1	Phlyctis petraea	Lecanoromycetes	Lichen-forming	N/A	9-21-24	CAXVCW01
phlyctis.speirea.1	GCA_964255155.1	Phlyctis speirea	Lecanoromycetes	Lichen-forming	N/A	9-21-24	CAXUVS01
phyllopsora.corallina.1	GCA_964256425.1	Phyllopsora corallina	Lecanoromycetes	Lichen-forming	N/A	9-21-24	CAXVBK01
phyllopsora.parvifolia.1	GCA_964257265.1	Phyllopsora parvifolia	Lecanoromycetes	Lichen-forming	N/A	9-21-24	CAXVEW01
physcia.biziana.1	GCA_964257135.1	Physcia biziana	Lecanoromycetes	Lichen-forming	N/A	9-21-24	CAXVDJ01
physcia.sp..1	GCA_937840595.1	Physcia sp.	Lecanoromycetes	Lichen-forming	N/A	1-12-23	CALGBB01
physcia.stellaris.1	GCA_018902385.1	Physcia stellaris	Lecanoromycetes	Lichen-forming	N/A	6-16-21	JABSSW01
physcia.stellaris.2	GCA_964256995.1	Physcia stellaris	Lecanoromycetes	Lichen-forming	N/A	9-21-24	CAXVDV01
physciaceae.sp..1	GCA_937854325.1	Physciaceae sp.	Lecanoromycetes	Lichen-forming	N/A	1-12-23	CALHDC01
physciaceae.sp..2	GCA_937857885.1	Physciaceae sp.	Lecanoromycetes	Lichen-forming	N/A	1-12-23	CALEEK01
physciaceae.sp..3	GCA_937857245.1	Physciaceae sp.	Lecanoromycetes	Lichen-forming	N/A	1-12-23	CALCMW01
physciaceae.sp..4	GCA_937857025.1	Physciaceae sp.	Lecanoromycetes	Lichen-forming	N/A	1-12-23	CALBWW01
physciaceae.sp..5	GCA_937858035.1	Physciaceae sp.	Lecanoromycetes	Lichen-forming	N/A	1-12-23	CAKZWZ01
physciella.chloantha.1	GCA_964257145.1	Physciella chloantha	Lecanoromycetes	Lichen-forming	N/A	9-21-24	CAXVDE01
pilophorus.fibula.1	GCA_964256335.1	Pilophorus fibula	Lecanoromycetes	Lichen-forming	N/A	9-21-24	CAXVAQ01
placynthium.petersii.1	GCA_964256885.1	Placynthium petersii	Lecanoromycetes	Lichen-forming	N/A	9-21-24	CAXVCT01
platismatia.glauca.1	GCA_963556305.1	Platismatia glauca	Lecanoromycetes	Lichen-forming	N/A	10-11-23	
platismatia.glauca.2	GCA_963556265.1	Platismatia glauca	Lecanoromycetes	Lichen-forming	N/A	10-11-23	CAUVYW01
platismatia.glauca.3	GCA_964255145.1	Platismatia glauca	Lecanoromycetes	Lichen-forming	N/A	9-21-24	CAXUTY01
platismatia.tuckermanii.1	GCA_964256155.1	Platismatia tuckermanii	Lecanoromycetes	Lichen-forming	N/A	9-21-24	CAXVAC01
pleopsidium.flavum.1	GCA_026027315.1	Pleopsidium flavum	Lecanoromycetes	Lichen-forming	N/A	11-7-22	JAPEUA01

polyblastidium.squamulosum.1	GCA_964257495.1	Polyblastidium squamulosum	Lecanoromycetes	Lichen-forming	N/A	9-21-24	CAXVFG01
polycauliona.sp..1	GCA_051942425.1	Polycauliona sp.	Lecanoromycetes	Lichen-forming	N/A	8-12-25	JAVECB01
polycauliona.sp..2	GCA_051941665.1	Polycauliona sp.	Lecanoromycetes	Lichen-forming	N/A	8-12-25	JAVEAK01
porina.heterospora.1	GCA_964256445.1	Porina heterospora	Lecanoromycetes	Lichen-forming	N/A	9-21-24	CAXVCA01
porpidia.albocaerulescens.1	GCA_964256365.1	Porpidia albocaerulescens	Lecanoromycetes	Lichen-forming	N/A	9-21-24	CAXVAV01
porpidia.contraponenda.1	GCA_964254925.1	Porpidia contraponenda	Lecanoromycetes	Lichen-forming	N/A	9-21-24	CAXUVY01
porpidia.crustulata.1	GCA_964255105.1	Porpidia crustulata	Lecanoromycetes	Lichen-forming	N/A	9-21-24	CAXUWE01
porpidia.macrocarpa.1	GCA_964257605.1	Porpidia macrocarpa	Lecanoromycetes	Lichen-forming	N/A	9-21-24	CAXVGD01
porpidia.subsimplex.1	GCA_964257625.1	Porpidia subsimplex	Lecanoromycetes	Lichen-forming	N/A	9-21-24	CAXVFV01
protoblastenia.rupestris.1	GCA_964256915.1	Protoblastenia rupestris	Lecanoromycetes	Lichen-forming	N/A	9-21-24	CAXVCR01
protoblastenia.rupestris.2	GCA_052058935.1	Protoblastenia rupestris	Lecanoromycetes	Lichen-forming	N/A	8-15-25	JAVECP01
pseudephebe.minuscula.1	GCA_964255465.1	Pseudephebe minuscula	Lecanoromycetes	Lichen-forming	N/A	9-21-24	CAXUVP01
pseudevernia.cladonia.1	GCA_964254705.1	Pseudevernia cladonia	Lecanoromycetes	Lichen-forming	N/A	9-21-24	CAXUVF01
pseudevernia.consocians.1	GCA_051942225.1	Pseudevernia consocians	Lecanoromycetes	Lichen-forming	N/A	8-12-25	JAVECM01
pseudevernia.furfuracea.1	GCA_022315165.1	Pseudevernia furfuracea	Lecanoromycetes	Lichen-forming	N/A	2-17-22	JAIUPS01
pseudevernia.furfuracea.2	GCA_022315425.1	Pseudevernia furfuracea	Lecanoromycetes	Lichen-forming	N/A	2-17-22	JAIUPT01
pseudevernia.furfuracea.3	GCA_003184345.1	Pseudevernia furfuracea	Lecanoromycetes	Lichen-forming	N/A	9-21-24	CAXVDS01
pseudevernia.furfuracea.4	GCA_964256985.1	Pseudevernia furfuracea	Lecanoromycetes	Lichen-forming	N/A	9-21-24	CAXVCZ01
pseudocyphellaria.aurata.1	GCA_022814125.1	Pseudocyphellaria aurata	Lecanoromycetes	Lichen-forming	N/A	4-4-22	JALDZL01
pseudosagedia.cestrensis.1	GCA_964257285.1	Pseudosagedia cestrensis	Lecanoromycetes	Lichen-forming	N/A	9-21-24	CAXVEY01
pseudosagedia.isidiata.1	GCA_964255805.1	Pseudosagedia isidiata	Lecanoromycetes	Lichen-forming	N/A	9-21-24	CAXUZD01
pseudosagedia.sp..1	GCA_964257195.1	Pseudosagedia sp.	Lecanoromycetes	Lichen-forming	N/A	9-21-24	CAXVEA01
psora.crenata.1	GCA_051942185.1	Psora crenata	Lecanoromycetes	Lichen-forming	N/A	8-12-25	JAVECR01
psora.testacea.1	GCA_051942125.1	Psora testacea	Lecanoromycetes	Lichen-forming	N/A	8-12-25	JAVECV01
ptychographa.xylographoides.1	GCA_022814105.1	Ptychographa xylographoides	Lecanoromycetes	Lichen-forming	N/A	4-4-22	JALDZM01
ptychographa.xylographoides.2	GCA_964257075.1	Ptychographa xylographoides	Lecanoromycetes	Lichen-forming	N/A	9-21-24	CAXVDK01
punctelia.appalachensis.1	GCA_964256225.1	Punctelia appalachensis	Lecanoromycetes	Lichen-forming	N/A	9-21-24	CAXVAH01
punctelia.caseana.1	GCA_964254955.1	Punctelia caseana	Lecanoromycetes	Lichen-forming	N/A	9-21-24	CAXUTU01
puttea.exsequens.1	GCA_022814085.1	Puttea exsequens	Lecanoromycetes	Lichen-forming	N/A	4-4-22	JALDZM01

puttea.exsequens.2	GCA_964257365.1	Puttea exsequens	Lecanoromycetes	Lichen-forming	N/A	9-21-24	CAXVEH01
pyrenodesmia.sp..1	GCA_051941895.1	Pyrenodesmia sp.	Lecanoromycetes	Lichen-forming	N/A	8-12-25	JAVEBS01
pyrenodesmia.sp..2	GCA_051941465.1	Pyrenodesmia sp.	Lecanoromycetes	Lichen-forming	N/A	8-12-25	JAVEAV01
pyrrhospora.varians.1	GCA_964256375.1	Pyrrhospora varians	Lecanoromycetes	Lichen-forming	N/A	9-21-24	CAXVBL01
pyxine.sorediata.1	GCA_964255475.1	Pyxine sorediata	Lecanoromycetes	Lichen-forming	N/A	9-21-24	CAXUWU01
ramalina.americana.1	GCA_964254555.1	Ramalina americana	Lecanoromycetes	Lichen-forming	N/A	9-21-24	CAXUUH01
ramalina.culbersoniorum.1	GCA_964255135.1	Ramalina culbersoniorum	Lecanoromycetes	Lichen-forming	N/A	9-21-24	CAXUVA01
ramalina.farinacea.1	GCA_029948375.1	Ramalina farinacea	Lecanoromycetes	Lichen-forming	N/A	5-4-23	JAPUFD01
ramalina.intermedia.1	GCA_003073195.1	Ramalina intermedia	Lecanoromycetes	Lichen-forming	N/A	4-26-18	PEKF01
ramalina.menziesii.1	GCA_964254825.1	Ramalina menziesii	Lecanoromycetes	Lichen-forming	N/A	9-21-24	CAXUTX01
ramalina.peruviana.1	GCA_001956345.1	Ramalina peruviana	Lecanoromycetes	Lichen-forming	N/A	1-18-17	MSTJ01
ramalina.petrina.1	GCA_964257245.1	Ramalina petrina	Lecanoromycetes	Lichen-forming	N/A	9-21-24	CAXVEF01
ramalina.siliquosa.1	GCA_965218175.1	Ramalina siliquosa	Lecanoromycetes	Lichen-forming	N/A	3-23-25	CBDCKL01
ramalina.thrausta.1	GCA_964257405.1	Ramalina thrausta	Lecanoromycetes	Lichen-forming	N/A	9-21-24	CAXVEP01
ramalinaceae.sp..1	GCA_964256575.1	Ramalinaceae sp.	Lecanoromycetes	Lichen-forming	N/A	9-21-24	CAXVBM01
rhizocarpon.geographicum.1	GCA_964257585.1	Rhizocarpon geographicum	Lecanoromycetes	Lichen-forming	N/A	9-21-24	CAXVFU01
rhizocarpon.subgeminatum.1	GCA_964255705.1	Rhizocarpon subgeminatum	Lecanoromycetes	Lichen-forming	N/A	9-21-24	CAXUYP01
rinodina.dolichospora.1	GCA_964257455.1	Rinodina dolichospora	Lecanoromycetes	Lichen-forming	N/A	9-21-24	CAXVFQ01
rinodina.sp..1	GCA_964256615.1	Rinodina sp.	Lecanoromycetes	Lichen-forming	N/A	9-21-24	CAXVBF01
rinodina.tephraspis.1	GCA_964254855.1	Rinodina tephraspis	Lecanoromycetes	Lichen-forming	N/A	9-21-24	CAXUXU01
rusavskia.elegans.1	GCA_011316305.1	Rusavskia elegans	Lecanoromycetes	Lichen-forming	N/A	3-16-20	SAUG01
rusavskia.elegans.2	GCA_051941435.1	Rusavskia elegans	Lecanoromycetes	Lichen-forming	N/A	8-12-25	JAVEAW01
schaereria.dolodes.1	GCA_964255955.1	Schaereria dolodes	Lecanoromycetes	Lichen-forming	N/A	9-21-24	CAXUZA01
schaereria.dolodes.2	GCA_022814065.1	Schaereria dolodes	Lecanoromycetes	Lichen-forming	N/A	4-4-22	JALDZO01
seirophora.lacunosa.1	GCA_023645825.1	Seirophora lacunosa	Lecanoromycetes	Lichen-forming	N/A	6-2-22	JALAHX01
seirophora.lacunosa.2	GCA_023646185.1	Seirophora lacunosa	Lecanoromycetes	Lichen-forming	N/A	6-2-22	JALAIF01
seirophora.lacunosa.3	GCA_051942285.1	Seirophora lacunosa	Lecanoromycetes	Lichen-forming	N/A	8-12-25	JAVECJ01
seirophora.scorigena.1	GCA_051942365.1	Seirophora scorigena	Lecanoromycetes	Lichen-forming	N/A	8-12-25	JAVECE01
seirophora.villosa.1	GCA_023645815.1	Seirophora villosa	Lecanoromycetes	Lichen-forming	N/A	6-2-22	JALAHY01
seirophora.villosa.2	GCA_051941785.1	Seirophora villosa	Lecanoromycetes	Lichen-forming	N/A	8-12-25	JAVEBX01

solorina.crocea.1	GCA_964257125.1	Solorina crocea	Lecanoromycetes	Lichen-forming	N/A	9-21-24	CAXVEC01
squamulea.sp..1	GCA_051941405.1	Squamulea sp.	Lecanoromycetes	Lichen-forming	N/A	8-12-25	JAVEAZ01
squamulea.squamosa.1	GCA_051941385.1	Squamulea squamosa	Lecanoromycetes	Lichen-forming	N/A	8-12-25	JAVEBA01
stereocaulon.alpinum.1	GCA_018257865.1	Stereocaulon alpinum	Lecanoromycetes	Lichen-forming	N/A	5-4-21	JAEUBA01
stereocaulon.dactylophyllum.1	GCA_964254575.1	Stereocaulon dactylophyllum	Lecanoromycetes	Lichen-forming	N/A	9-21-24	CAXUUX01
stereocaulon.virgatum.1	GCA_042464655.1	Stereocaulon virgatum	Lecanoromycetes	Lichen-forming	N/A	9-30-24	JBEFKJ01
sticta.canariensis.1	GCA_022814045.1	Sticta canariensis	Lecanoromycetes	Lichen-forming	N/A	4-4-22	JALDZP01
sticta.sp..1	GCA_964254715.1	Sticta sp.	Lecanoromycetes	Lichen-forming	N/A	9-21-24	CAXUWN01
stictis.urceolata.1	GCA_022814025.1	Stictis urceolata	Lecanoromycetes	Lichen-forming	N/A	4-4-22	JALDZQ01
stictis.urceolata.2	GCA_964257505.1	Stictis urceolata	Lecanoromycetes	Lichen-forming	N/A	9-21-24	CAXVFL01
sulcaria.badia.1	GCA_964257055.1	Sulcaria badia	Lecanoromycetes	Lichen-forming	N/A	9-21-24	CAXVDM01
teloschistales.sp..1	GCA_964255085.1	Teloschistales sp.	Lecanoromycetes	Lichen-forming	N/A	9-21-24	CAXUVJ01
teloschistes.chrysophthalmus.1	GCA_023646055.1	Teloschistes chrysophthalmus	Lecanoromycetes	Lichen-forming	N/A	6-2-22	JALAIL01
teloschistes.chrysophthalmus.2	GCA_051940965.1	Teloschistes chrysophthalmus	Lecanoromycetes	Lichen-forming	N/A	8-12-25	JAVEAD01
teloschistes.exilis.1	GCA_052059115.1	Teloschistes exilis	Lecanoromycetes	Lichen-forming	N/A	8-15-25	JAVEAI01
teloschistes.exilis.2	GCA_051941425.1	Teloschistes exilis	Lecanoromycetes	Lichen-forming	N/A	8-12-25	JAVEAX01
teloschistes.exilis.3	GCA_051940905.1	Teloschistes exilis	Lecanoromycetes	Lichen-forming	N/A	8-12-25	JAVEAE01
teloschistes.flavicans.1	GCA_051941685.1	Teloschistes flavicans	Lecanoromycetes	Lichen-forming	N/A	8-12-25	JAVEAJ01
teloschistes.flavicans.2	GCA_023646325.1	Teloschistes flavicans	Lecanoromycetes	Lichen-forming	N/A	6-2-22	JALAIQ01
teloschistes.hypoglaucus.1	GCA_052059135.1	Teloschistes hypoglaucus	Lecanoromycetes	Lichen-forming	N/A	8-15-25	JAVEAH01
teloschistes.peruensis.1	GCA_023645835.1	Teloschistes peruensis	Lecanoromycetes	Lichen-forming	N/A	6-2-22	JALAHW01
thelotrema.defectum.1	GCA_964257675.1	Thelotrema defectum	Lecanoromycetes	Lichen-forming	N/A	9-21-24	CAXVFP01
thelotrema.lepadinum.1	GCA_022813995.1	Thelotrema lepadinum	Lecanoromycetes	Lichen-forming	N/A	4-4-22	JALDZR01
thelotrema.lepadinum.2	GCA_964257395.1	Thelotrema lepadinum	Lecanoromycetes	Lichen-forming	N/A	9-21-24	CAXVET01
thelotrema.subtile.1	GCA_964256685.1	Thelotrema subtile	Lecanoromycetes	Lichen-forming	N/A	9-21-24	CAXVBY01
toensbergia.leucococca.1	GCA_022813965.1	Toensbergia leucococca	Lecanoromycetes	Lichen-forming	N/A	4-4-22	JALDZS01
toensbergia.leucococca.2	GCA_964255925.1	Toensbergia leucococca	Lecanoromycetes	Lichen-forming	N/A	9-21-24	CAXUZM01
toniniopsis.dissimilis.1	GCA_043520055.1	Toniniopsis dissimilis	Lecanoromycetes	Lichen-forming	N/A	10-21-24	JBGRPS01
trapelia.coarctata.1	GCA_022813945.1	Trapelia coarctata	Lecanoromycetes	Lichen-forming	N/A	4-4-22	JALDZT01
trapelia.coarctata.2	GCA_964256815.1	Trapelia coarctata	Lecanoromycetes	Lichen-forming	N/A	9-21-24	CAXVCH01

trapeliopsis.flexuosa.1	GCA_964254545.1	Trapeliopsis flexuosa	Lecanoromycetes	Lichen-forming	N/A	9-21-24	CAXUUY01
tuckermannopsis.ciliaris.1	GCA_964255215.1	Tuckermannopsis ciliaris	Lecanoromycetes	Lichen-forming	N/A	9-21-24	CAXUVQ01
umbilicaria.americana.1	GCA_964255515.1	Umbilicaria americana	Lecanoromycetes	Lichen-forming	N/A	9-21-24	CAXUVK01
umbilicaria.crustulosa.1	GCA_053478165.1	Umbilicaria crustulosa	Lecanoromycetes	Lichen-forming	N/A	11-14-25	JBNORC01
umbilicaria.crustulosa.2	GCA_053478185.1	Umbilicaria crustulosa	Lecanoromycetes	Lichen-forming	N/A	11-14-25	JBNORB01
umbilicaria.crustulosa.3	GCA_053478205.1	Umbilicaria crustulosa	Lecanoromycetes	Lichen-forming	N/A	11-14-25	JBNORA01
umbilicaria.deusta.1	GCA_023184635.1	Umbilicaria deusta	Lecanoromycetes	Lichen-forming	N/A	4-29-22	JALILR01
umbilicaria.deusta.2	GCA_053478105.1	Umbilicaria deusta	Lecanoromycetes	Lichen-forming	N/A	11-14-25	JBNORE01
umbilicaria.deusta.3	GCA_053478145.1	Umbilicaria deusta	Lecanoromycetes	Lichen-forming	N/A	11-14-25	JBNORD01
umbilicaria.deusta.4	GCA_964340765.1	Umbilicaria deusta	Lecanoromycetes	Lichen-forming	N/A	11-18-24	CAYFAF01
umbilicaria.deusta.5	GCA_964340595.1	Umbilicaria deusta	Lecanoromycetes	Lichen-forming	N/A	11-18-24	CAYFAE01
umbilicaria.freyi.1	GCA_023184675.1	Umbilicaria freyi	Lecanoromycetes	Lichen-forming	N/A	4-29-22	JALILQ01
umbilicaria.freyi.2	GCA_053478085.1	Umbilicaria freyi	Lecanoromycetes	Lichen-forming	N/A	11-14-25	JBNORG01
umbilicaria.freyi.3	GCA_053478125.1	Umbilicaria freyi	Lecanoromycetes	Lichen-forming	N/A	11-14-25	JBNORF01
umbilicaria.grisea.1	GCA_023184595.1	Umbilicaria grisea	Lecanoromycetes	Lichen-forming	N/A	4-29-22	JALILX01
umbilicaria.grisea.2	GCA_053477165.1	Umbilicaria grisea	Lecanoromycetes	Lichen-forming	N/A	11-14-25	JBNORI01
umbilicaria.grisea.3	GCA_053478065.1	Umbilicaria grisea	Lecanoromycetes	Lichen-forming	N/A	11-14-25	JBNORH01
umbilicaria.hispanica.1	GCA_023184715.1	Umbilicaria hispanica	Lecanoromycetes	Lichen-forming	N/A	4-29-22	JALILS01
umbilicaria.hispanica.2	GCA_053478025.1	Umbilicaria hispanica	Lecanoromycetes	Lichen-forming	N/A	11-14-25	JBNORK01
umbilicaria.hispanica.3	GCA_053478045.1	Umbilicaria hispanica	Lecanoromycetes	Lichen-forming	N/A	11-14-25	JBNORJ01
umbilicaria.hispanica.4	GCA_003254425.1	Umbilicaria hispanica	Lecanoromycetes	Lichen-forming	N/A	6-18-18	PKMA01
umbilicaria.iberica.1	GCA_053477965.1	Umbilicaria iberica	Lecanoromycetes	Lichen-forming	N/A	11-14-25	JBNORN01
umbilicaria.iberica.2	GCA_053477985.1	Umbilicaria iberica	Lecanoromycetes	Lichen-forming	N/A	11-14-25	JBNORM01
umbilicaria.iberica.3	GCA_053478005.1	Umbilicaria iberica	Lecanoromycetes	Lichen-forming	N/A	11-14-25	JBNORL01
umbilicaria.mammulata.1	GCA_964255095.1	Umbilicaria mammulata	Lecanoromycetes	Lichen-forming	N/A	9-21-24	CAXUVM01
umbilicaria.muhlenbergii.1	GCA_000611775.1	Umbilicaria muhlenbergii	Lecanoromycetes	Lichen-forming	N/A	4-2-14	JFDN01
umbilicaria.nylanderiana.1	GCA_053477925.1	Umbilicaria nylanderiana	Lecanoromycetes	Lichen-forming	N/A	11-14-25	JBNORP01
umbilicaria.nylanderiana.2	GCA_053477945.1	Umbilicaria nylanderiana	Lecanoromycetes	Lichen-forming	N/A	11-14-25	JBNORO01
umbilicaria.phaea.1	GCA_023184695.1	Umbilicaria phaea	Lecanoromycetes	Lichen-forming	N/A	4-29-22	JALILT01
umbilicaria.phaea.2	GCA_053477885.1	Umbilicaria phaea	Lecanoromycetes	Lichen-forming	N/A	11-14-25	JBNORQ01
umbilicaria.phaea.3	GCA_053477905.1	Umbilicaria phaea	Lecanoromycetes	Lichen-forming	N/A	11-14-25	JBNORR01

umbilicaria.polyphylla.1	GCA_053477805.1	Umbilicaria polyphylla	Lecanoromycetes	Lichen-forming	N/A	11-14-25	JBNORV01
umbilicaria.polyphylla.2	GCA_053477825.1	Umbilicaria polyphylla	Lecanoromycetes	Lichen-forming	N/A	11-14-25	JBNORS01
umbilicaria.polyphylla.3	GCA_053477845.1	Umbilicaria polyphylla	Lecanoromycetes	Lichen-forming	N/A	11-14-25	JBNORU01
umbilicaria.polyphylla.4	GCA_053477865.1	Umbilicaria polyphylla	Lecanoromycetes	Lichen-forming	N/A	11-14-25	JBNORT01
umbilicaria.spodochroa.1	GCA_023184705.1	Umbilicaria spodochroa	Lecanoromycetes	Lichen-forming	N/A	4-29-22	JALILV01
umbilicaria.spodochroa.2	GCA_053477155.1	Umbilicaria spodochroa	Lecanoromycetes	Lichen-forming	N/A	11-14-25	JBNOSA01
umbilicaria.spodochroa.3	GCA_053477705.1	Umbilicaria spodochroa	Lecanoromycetes	Lichen-forming	N/A	11-14-25	JBNORZ01
umbilicaria.spodochroa.4	GCA_053477745.1	Umbilicaria spodochroa	Lecanoromycetes	Lichen-forming	N/A	11-14-25	JBNORY01
umbilicaria.subpolyphylla.1	GCA_023184615.1	Umbilicaria subpolyphylla	Lecanoromycetes	Lichen-forming	N/A	4-29-22	JALILW01
umbilicaria.vellea.1	GCA_023645895.1	Umbilicaria vellea	Lecanoromycetes	Lichen-forming	N/A	6-2-22	JALAID01
usnea.aurantiacoatra.1	GCA_964256655.1	Usnea aurantiacoatra	Lecanoromycetes	Lichen-forming	N/A	9-21-24	CAXVEB01
usnea.cavernosa.1	GCA_964255125.1	Usnea cavernosa	Lecanoromycetes	Lichen-forming	N/A	9-21-24	CAXUUF01
usnea.ceratina.1	GCA_964256075.1	Usnea ceratina	Lecanoromycetes	Lichen-forming	N/A	9-21-24	CAXUZW01
usnea.ceratina.2	GCA_964257515.1	Usnea ceratina	Lecanoromycetes	Lichen-forming	N/A	9-21-24	CAXVFM01
usnea.cornuta.1	GCA_964256645.1	Usnea cornuta	Lecanoromycetes	Lichen-forming	N/A	9-21-24	CAXVDP01
usnea.florida.1	GCA_052784015.1	Usnea florida	Lecanoromycetes	Lichen-forming	N/A	4-4-22	JALEAC01
usnea.hakonensis.1	GCA_013423325.1	Usnea hakonensis	Lecanoromycetes	Lichen-forming	N/A	7-13-20	BLJB01
usnea.halei.1	GCA_964255035.1	Usnea halei	Lecanoromycetes	Lichen-forming	N/A	9-21-24	CAXUVX01
usnea.merrillii.1	GCA_964255025.1	Usnea merrillii	Lecanoromycetes	Lichen-forming	N/A	9-21-24	CAXUXB01
usnea.mutabilis.1	GCA_964257655.1	Usnea mutabilis	Lecanoromycetes	Lichen-forming	N/A	9-21-24	CAXVFR01
usnea.strigosa.1	GCA_964254745.1	Usnea strigosa	Lecanoromycetes	Lichen-forming	N/A	9-21-24	CAXUWL01
usnea.subfusca.1	GCA_964256035.1	Usnea subfusca	Lecanoromycetes	Lichen-forming	N/A	9-21-24	CAXVAB01
usnea.subgracilis.1	GCA_964255225.1	Usnea subgracilis	Lecanoromycetes	Lichen-forming	N/A	9-21-24	CAXUYA01
usnea.subscabrosa.1	GCA_964254585.1	Usnea subscabrosa	Lecanoromycetes	Lichen-forming	N/A	9-21-24	CAXUWV01
usnocetraria.oakesiana.1	GCA_964254695.1	Usnocetraria oakesiana	Lecanoromycetes	Lichen-forming	N/A	9-21-24	CAXUXA01
usnochroma.carphineum.1	GCA_023645975.1	Usnochroma carphineum	Lecanoromycetes	Lichen-forming	N/A	6-2-22	JALAHV01
varicellaria.rhodocarpa.1	GCA_022813925.1	Varicellaria rhodocarpa	Lecanoromycetes	Lichen-forming	N/A	4-4-22	JALDZU01
varicellaria.rhodocarpa.2	GCA_964255865.1	Varicellaria rhodocarpa	Lecanoromycetes	Lichen-forming	N/A	9-21-24	CAXUYX01
variospora.aurantia.1	GCA_023645955.1	Variospora aurantia	Lecanoromycetes	Lichen-forming	N/A	6-2-22	JALAHZ01
variospora.flavescens.1	GCA_051942445.1	Variospora flavescens	Lecanoromycetes	Lichen-forming	N/A	8-12-25	JAVECA01
variospora.fuerteventurae.1	GCA_051941765.1	Variospora fuerteventurae	Lecanoromycetes	Lichen-forming	N/A	8-12-25	JAVEBY01

variospora.sp..1	GCA_051942325.1	Variospora sp.	Lecanoromycetes	Lichen-forming	N/A	8-12-25	JAVECF01
variospora.velana.1	GCA_051941705.1	Variospora velana	Lecanoromycetes	Lichen-forming	N/A	8-12-25	JAVEAG01
villophora.microphyllina.1	GCA_051941605.1	Villophora microphyllina	Lecanoromycetes	Lichen-forming	N/A	8-12-25	JAVEAN01
vulpicida.juniperinus.1	GCA_964255425.1	Vulpicida juniperinus	Lecanoromycetes	Lichen-forming	N/A	9-21-24	CAXUTW01
wetmoreana.brouardii.1	GCA_051941545.1	Wetmoreana brouardii	Lecanoromycetes	Lichen-forming	N/A	8-12-25	JAVEAS01
wetmoreana.sp..1	GCA_052059095.1	Wetmoreana sp.	Lecanoromycetes	Lichen-forming	N/A	8-15-25	JAVEAP01
xanthocarpia.feracissima.1	GCA_964255735.1	Xanthocarpia feracissima	Lecanoromycetes	Lichen-forming	N/A	9-21-24	CAXUYH01
xanthocarpia.lactea.1	GCA_051941915.1	Xanthocarpia lactea	Lecanoromycetes	Lichen-forming	N/A	8-12-25	JAVEBR01
xanthocarpia.ochracea.1	GCA_051941935.1	Xanthocarpia ochracea	Lecanoromycetes	Lichen-forming	N/A	8-12-25	JAVEBQ01
xanthomendoza.sp..1	GCA_051941585.1	Xanthomendoza sp.	Lecanoromycetes	Lichen-forming	N/A	8-12-25	JAVEAM01
xanthomendoza.sp..2	GCA_051941345.1	Xanthomendoza sp.	Lecanoromycetes	Lichen-forming	N/A	8-12-25	JAVEBC01
xanthoparmelia.plittii.1	GCA_964256595.1	Xanthoparmelia plittii	Lecanoromycetes	Lichen-forming	N/A	9-21-24	CAXVBC01
xanthoparmelia.taractica.1	GCA_033085395.1	Xanthoparmelia taractica	Lecanoromycetes	Lichen-forming	N/A	10-16-23	JANQBS01
xanthoparmelia.tasmanica.1	GCA_964256055.1	Xanthoparmelia tasmanica	Lecanoromycetes	Lichen-forming	N/A	9-21-24	CAXVCK01
xanthoparmelia.verrucella.1	GCA_964256005.1	Xanthoparmelia verrucella	Lecanoromycetes	Lichen-forming	N/A	9-21-24	CAXUZO01
xanthoria.aureola.1	GCA_023646375.1	Xanthoria aureola	Lecanoromycetes	Lichen-forming	N/A	6-2-22	JALAII01
xanthoria.mediterranea.1	GCA_023645845.1	Xanthoria mediterranea	Lecanoromycetes	Lichen-forming	N/A	6-2-22	JALAIA01
xanthoria.sp..1	GCA_023647215.1	Xanthoria sp.	Lecanoromycetes	Lichen-forming	N/A	6-3-22	JALAIR01
xanthoria.sp..2	GCA_023646395.1	Xanthoria sp.	Lecanoromycetes	Lichen-forming	N/A	6-2-22	JALAIM01
xanthoria.steineri.1	GCA_023646075.1	Xanthoria steineri	Lecanoromycetes	Lichen-forming	N/A	6-2-22	JALAIK01
xylographa.bjoerkii.1	GCA_022813905.1	Xylographa bjoerkii	Lecanoromycetes	Lichen-forming	N/A	4-4-22	JALDZV01
xylographa.carneopallida.1	GCA_022813885.1	Xylographa carneopallida	Lecanoromycetes	Lichen-forming	N/A	4-4-22	JALDZW01
xylographa.carneopallida.2	GCA_964255915.1	Xylographa carneopallida	Lecanoromycetes	Lichen-forming	N/A	9-21-24	CAXUYT01
xylographa.opegraphella.1	GCA_022813865.1	Xylographa opegraphella	Lecanoromycetes	Lichen-forming	N/A	4-4-22	JALDZX01
xylographa.pallens.1	GCA_022813845.1	Xylographa pallens	Lecanoromycetes	Lichen-forming	N/A	4-4-22	JALDZY01
xylographa.parallela.1	GCA_022813825.1	Xylographa parallela	Lecanoromycetes	Lichen-forming	N/A	4-4-22	JALDZZ01
xylographa.soralifera.1	GCA_022813805.1	Xylographa soralifera	Lecanoromycetes	Lichen-forming	N/A	4-4-22	JALEAA01
xylographa.trunciseda.1	GCA_022813785.1	Xylographa trunciseda	Lecanoromycetes	Lichen-forming	N/A	4-4-22	JALEAB01
xylographa.trunciseda.2	GCA_964255115.1	Xylographa trunciseda	Lecanoromycetes	Lichen-forming	N/A	9-21-24	CAXUVE01
xylographa.vitiligo.1	GCA_022813765.1	Xylographa vitiligo	Lecanoromycetes	Lichen-forming	N/A	4-4-22	JALEAC01
xylographa.vitiligo.2	GCA_964256195.1	Xylographa vitiligo	Lecanoromycetes	Lichen-forming	N/A	9-21-24	CAXUZQ01

xylographa.vitiligo.3	GCA_964256275.1	Xylographa vitiligo	Lecanoromycetes	Lichen-forming	N/A	9-21-24	CAXVBN01

Table S18. Tyrosine recombinases (tyrRs) included in the structure-based phylogeny. The set included manually selected representative set of captians from Gluck-Thaler&Vogan (2024), predicted tyrRs from a larger set of lichen-associated fungi, and Tangerine captains. The family assignments correspond to that from Gluck-Thaler&Vogan (2024).

Captain	familyID	Source	Genome
lambiella.insularis.1_tyr2509		Newly-annotated lichen genomes	lambiella.insularis.1
porpidia.subsimplex.1_tyr3430		Newly-annotated lichen genomes	porpidia.subsimplex.1
porpidia.macrocarpa.1_tyr3848		Newly-annotated lichen genomes	porpidia.macrocarpa.1
caloplaca.sp..2_tyr2595		Newly-annotated lichen genomes	caloplaca.sp..2
baeomycetales.sp..3_tyr2166		Newly-annotated lichen genomes	baeomycetales.sp..3
Starship-1_Tmar-Captain	fam02	Gluck-Thaler and Vogan (2024)	
aspsyd2_tyr2767	fam02	Gluck-Thaler and Vogan (2024)	aspsyd2
aspgla1_OJJ83717.1	fam02	Gluck-Thaler and Vogan (2024)	aspgla1
Starship-1_Afel-Captain	fam02	Gluck-Thaler and Vogan (2024)	
Starship-1_Afum-Captain	fam02	Gluck-Thaler and Vogan (2024)	
HEPHAESTUS_Pvar-Captain	fam02	Gluck-Thaler and Vogan (2024)	
talver1_KUL81284.1	fam02	Gluck-Thaler and Vogan (2024)	talver1
acarospora.strigata.1_tyr1148		Newly-annotated lichen genomes	acarospora.strigata.1
blader3_11177	fam02	Gluck-Thaler and Vogan (2024)	blader3
xanthoria.parietina.3_tyr2694		Newly-annotated lichen genomes	xanthoria.parietina.3
usnea.strigosa.1_tyr808		Newly-annotated lichen genomes	usnea.strigosa.1
aspnig10_tyr2036	fam02	Gluck-Thaler and Vogan (2024)	aspnig10
solorina.crocea.1_tyr1894		Newly-annotated lichen genomes	solorina.crocea.1
peltigera.extenuata.1_tyr2566		Newly-annotated lichen genomes	peltigera.extenuata.1
solorina.crocea.1_tyr2911		Newly-annotated lichen genomes	solorina.crocea.1
gluame1_KAH0542861.1	clade01	Gluck-Thaler and Vogan (2024)	gluame1
sticta.sp..1_tyr2883		Newly-annotated lichen genomes	sticta.sp..1

cladonia.sp..7_tyr4047		Newly-annotated lichen genomes	cladonia.sp..7
usnea.hakonensis.1_tyr3170		Newly-annotated lichen genomes	usnea.hakonensis.1
gyalolechia.sp..2_tyr1533		Newly-annotated lichen genomes	gyalolechia.sp..2
imsale1_tyr7676	fam05	Gluck-Thaler and Vogan (2024)	imsale1
grascr1_10188	fam05	Gluck-Thaler and Vogan (2024)	grascr1
sticta.canariensis.1_tyr4091		Newly-annotated lichen genomes	sticta.canariensis.1
protoblastenia.rupestris.2_tyr3826		Newly-annotated lichen genomes	protoblastenia.rupestris.2
lecanora.subimmergens.1_tyr4145		Newly-annotated lichen genomes	lecanora.subimmergens.1
lecanora.markjohnstonii.1_tyr1576		Newly-annotated lichen genomes	lecanora.markjohnstonii.1
biatora.pontica.1_tyr2094		Newly-annotated lichen genomes	biatora.pontica.1
calogaya.arnoldii.1_tyr2951		Newly-annotated lichen genomes	calogaya.arnoldii.1
porpidia.macrocarpa.1_tyr578		Newly-annotated lichen genomes	porpidia.macrocarpa.1
lecidea.sp..1_tyr968		Newly-annotated lichen genomes	lecidea.sp..1
lecanoromycetes.sp..3_tyr122		Newly-annotated lichen genomes	lecanoromycetes.sp..3
cladonia.sp..9_tyr1278		Newly-annotated lichen genomes	cladonia.sp..9
usnea.ceratina.2_tyr4164		Newly-annotated lichen genomes	usnea.ceratina.2
diploschistes.diacapsis.1_tyr1448		Newly-annotated lichen genomes	diploschistes.diacapsis.1
parmeliaceae.sp..20_tyr1063		Newly-annotated lichen genomes	parmeliaceae.sp..20
parmotrema.stuppeum.1_tyr3760		Newly-annotated lichen genomes	parmotrema.stuppeum.1
cladonia.rangiferina.2_tyr2351		Newly-annotated lichen genomes	cladonia.rangiferina.2
protoblastenia.rupestris.1_tyr3520		Newly-annotated lichen genomes	protoblastenia.rupestris.1
exoxen1_tyr5127	fam05	Gluck-Thaler and Vogan (2024)	exoxen1
talrug1_tyr10331	fam05	Gluck-Thaler and Vogan (2024)	talrug1
talpin1_KAF3392106.1	fam05	Gluck-Thaler and Vogan (2024)	talpin1
umbilicaria.hispanica.4_tyr1397		Newly-annotated lichen genomes	umbilicaria.hispanica.4
umbilicaria.deusta.1_tyr131		Newly-annotated lichen genomes	umbilicaria.deusta.1

coniocarpon.cinnabarinum.2_tyr169		Newly-annotated lichen genomes	coniocarpon.cinnabarinum.2
hiscap4_tyr7429	fam05	Gluck-Thaler and Vogan (2024)	hiscap4
emmcre2_KKZ61489.1	fam05	Gluck-Thaler and Vogan (2024)	emmcre2
asppho1_RDK41021.1	fam05	Gluck-Thaler and Vogan (2024)	asppho1
aspory7_tyr2325	fam05	Gluck-Thaler and Vogan (2024)	aspory7
triton1_5425	fam05	Gluck-Thaler and Vogan (2024)	triton1
aspasp7_tyr613	fam05	Gluck-Thaler and Vogan (2024)	aspasp7
talsti1_5901	fam05	Gluck-Thaler and Vogan (2024)	talsti1
talatr1_4403	fam05	Gluck-Thaler and Vogan (2024)	talatr1
fussp.6_KAF5007163.1	fam05	Gluck-Thaler and Vogan (2024)	fussp.6
aspuda6_tyr3047	fam05	Gluck-Thaler and Vogan (2024)	aspuda6
aspara2_tyr470	fam05	Gluck-Thaler and Vogan (2024)	aspara2
paeniv1_62125	fam05	Gluck-Thaler and Vogan (2024)	paeniv1
penroq1_3856	fam05	Gluck-Thaler and Vogan (2024)	penroq1
aspflo1_tyr1229	fam05	Gluck-Thaler and Vogan (2024)	aspflo1
aspber2_KAE8379024.1	fam05	Gluck-Thaler and Vogan (2024)	aspber2
elagra2_2112	fam05	Gluck-Thaler and Vogan (2024)	elagra2
aspasp2_tyr555	fam05	Gluck-Thaler and Vogan (2024)	aspasp2
asppar5_tyr2394	fam05	Gluck-Thaler and Vogan (2024)	asppar5
aspnom1_155723	fam05	Gluck-Thaler and Vogan (2024)	aspnom1
aspter9_tyr2936	fam05	Gluck-Thaler and Vogan (2024)	aspter9
emmcre2_KKZ60871.1	fam05	Gluck-Thaler and Vogan (2024)	emmcre2
blaper1_9954	fam05	Gluck-Thaler and Vogan (2024)	blaper1
blaper1_2381	fam05	Gluck-Thaler and Vogan (2024)	blaper1
hiscap1_tyr7335	fam05	Gluck-Thaler and Vogan (2024)	hiscap1
aspnid4_tyr2028	fam05	Gluck-Thaler and Vogan (2024)	aspnid4

aspscl6_tyr2635	fam05	Gluck-Thaler and Vogan (2024)	aspscl6
Starship-2_Afla-Captain	fam05	Gluck-Thaler and Vogan (2024)	
pensp.11_PCG88942.1	fam05	Gluck-Thaler and Vogan (2024)	pensp.11
lenflu1_KAF2676861.1	fam05	Gluck-Thaler and Vogan (2024)	lenflu1
cocmiy1_41658	fam05	Gluck-Thaler and Vogan (2024)	cocmiy1
pyrtri5_tyr9846	fam05	Gluck-Thaler and Vogan (2024)	pyrtri5
ascrab1_tyr338	fam05	Gluck-Thaler and Vogan (2024)	ascrab1
bipmay1_ENH98990.1	fam05	Gluck-Thaler and Vogan (2024)	bipmay1
pennor1_tyr8865	fam05	Gluck-Thaler and Vogan (2024)	pennor1
psesp.16_tyr9418	fam05	Gluck-Thaler and Vogan (2024)	psesp.16
bimnov1_tyr3321	fam05	Gluck-Thaler and Vogan (2024)	bimnov1
cengeo1_tyr3835	fam05	Gluck-Thaler and Vogan (2024)	cengeo1
mytres1_384697	fam05	Gluck-Thaler and Vogan (2024)	mytres1
mytres1_491224	fam05	Gluck-Thaler and Vogan (2024)	mytres1
peltigera.leucophlebia.1_tyr4120		Newly-annotated lichen genomes	peltigera.leucophlebia.1
calmar1_577054	fam05	Gluck-Thaler and Vogan (2024)	calmar1
trihir1_KAH0563021.1	fam05	Gluck-Thaler and Vogan (2024)	trihir1
phisco1_133811	fam05	Gluck-Thaler and Vogan (2024)	phisco1
calmar1_178488	fam05	Gluck-Thaler and Vogan (2024)	calmar1
rhysec1_tyr10006	fam05	Gluck-Thaler and Vogan (2024)	rhysec1
baeomycetales.sp..2_tyr1857		Newly-annotated lichen genomes	baeomycetales.sp..2
acemac1_707798	fam05	Gluck-Thaler and Vogan (2024)	acemac1
icmadophila.ericetorum.1_tyr4177		Newly-annotated lichen genomes	icmadophila.ericetorum.1
rhysec1_tyr10005	fam05	Gluck-Thaler and Vogan (2024)	rhysec1
lacari1_TVY13717.1	fam05	Gluck-Thaler and Vogan (2024)	lacari1
umbilicaria.spodochroa.2_tyr828		Newly-annotated lichen genomes	umbilicaria.spodochroa.2

lachya1_TVY25215.1	fam05	Gluck-Thaler and Vogan (2024)	lachya1
bipmay2_EMD88483.1	fam05	Gluck-Thaler and Vogan (2024)	bipmay2
zoprhi1_tyr11022	fam05	Gluck-Thaler and Vogan (2024)	zoprhi1
zoprhi1_KAF2184210.1	fam05	Gluck-Thaler and Vogan (2024)	zoprhi1
hypocenomyce.scalaris.2_tyr2723		Newly-annotated lichen genomes	hypocenomyce.scalaris.2
fuscopannaria.leucosticta.1_tyr170		Newly-annotated lichen genomes	fuscopannaria.leucosticta.1
flavoplaca.sp..1_tyr2392		Newly-annotated lichen genomes	flavoplaca.sp..1
treper1_542511	fam05	Gluck-Thaler and Vogan (2024)	treper1
fuspro7_tyr6510	fam05	Gluck-Thaler and Vogan (2024)	fuspro7
fussp.5_KAF5611984.1	fam05	Gluck-Thaler and Vogan (2024)	fussp.5
fustup1_tyr6815	fam05	Gluck-Thaler and Vogan (2024)	fustup1
fusfus29_tyr5699	fam05	Gluck-Thaler and Vogan (2024)	fusfus29
fususs4_tyr6846	fam05	Gluck-Thaler and Vogan (2024)	fususs4
fusequ4_tyr5487	fam05	Gluck-Thaler and Vogan (2024)	fusequ4
fusfus65_tyr5881	fam05	Gluck-Thaler and Vogan (2024)	fusfus65
Starship-1_Foxy_Fo5176-Captain	fam05	Gluck-Thaler and Vogan (2024)	
dacmac1_659788	fam05	Gluck-Thaler and Vogan (2024)	dacmac1
fusequ5_tyr5495	fam05	Gluck-Thaler and Vogan (2024)	fusequ5
mictri1_425164	fam05	Gluck-Thaler and Vogan (2024)	mictri1
toloph1_1610	fam05	Gluck-Thaler and Vogan (2024)	toloph1
porpidia.macrocarpa.1_tyr2028		Newly-annotated lichen genomes	porpidia.macrocarpa.1
hirrho1_KAH0961187.1	fam05	Gluck-Thaler and Vogan (2024)	hirrho1
colcam1_tyr4276	fam05	Gluck-Thaler and Vogan (2024)	colcam1
xylsp.18_tyr10859	fam05	Gluck-Thaler and Vogan (2024)	xylsp.18
funsp.2_GAW15977.1	fam05	Gluck-Thaler and Vogan (2024)	funsp.2
xyllon1_530278	fam05	Gluck-Thaler and Vogan (2024)	xyllon1

xylsp.11_322789	fam05	Gluck-Thaler and Vogan (2024)	xylsp.11
xylcub1_494674	fam05	Gluck-Thaler and Vogan (2024)	xylcub1
xylsp.15_432324	fam05	Gluck-Thaler and Vogan (2024)	xylsp.15
diaamp1_10693	fam05	Gluck-Thaler and Vogan (2024)	diaamp1
xylpal1_601133	fam05	Gluck-Thaler and Vogan (2024)	xylpal1
xylsp.2_355315	fam05	Gluck-Thaler and Vogan (2024)	xylsp.2
elagra2_tyr4808	fam05	Gluck-Thaler and Vogan (2024)	elagra2
aspter9_tyr2940	fam05	Gluck-Thaler and Vogan (2024)	aspter9
parbra2_EEH47566.2	fam05	Gluck-Thaler and Vogan (2024)	parbra2
asplat5_tyr1682	fam05	Gluck-Thaler and Vogan (2024)	asplat5
pesfic1_tyr9064	fam05	Gluck-Thaler and Vogan (2024)	pesfic1
talmar2_KFX47975.1	fam05	Gluck-Thaler and Vogan (2024)	talmar2
penbra1_tyr8704	fam05	Gluck-Thaler and Vogan (2024)	penbra1
aspqua1_tyr2555	fam05	Gluck-Thaler and Vogan (2024)	aspqua1
aspasp12_tyr525	fam05	Gluck-Thaler and Vogan (2024)	aspasp12
asplep2_tyr1824	fam05	Gluck-Thaler and Vogan (2024)	asplep2
aspfla13_tyr1195	fam05	Gluck-Thaler and Vogan (2024)	aspfla13
penrub1_CAP94151.1	fam05	Gluck-Thaler and Vogan (2024)	penrub1
penroq1_4089	fam05	Gluck-Thaler and Vogan (2024)	penroq1
penoxa1_6225	fam05	Gluck-Thaler and Vogan (2024)	penoxa1
lasallia.pustulata.7_tyr482		Newly-annotated lichen genomes	lasallia.pustulata.7
lasallia.pustulata.1_tyr3965		Newly-annotated lichen genomes	lasallia.pustulata.1
rutsp.1_PQE27284.1	fam05	Gluck-Thaler and Vogan (2024)	rutsp.1
emmcre2_KKZ61922.1	fam05	Gluck-Thaler and Vogan (2024)	emmcre2
blader5_EQL32851.1	fam05	Gluck-Thaler and Vogan (2024)	blader5
polhys1_6343	fam05	Gluck-Thaler and Vogan (2024)	polhys1

parbra1_2719	fam05	Gluck-Thaler and Vogan (2024)	parbra1
parbra2_EEH48327.2	fam05	Gluck-Thaler and Vogan (2024)	parbra2
emepas2_OJD10625.1	fam05	Gluck-Thaler and Vogan (2024)	emepas2
botdot1_290492	fam05	Gluck-Thaler and Vogan (2024)	botdot1
lasthe1_tyr7811	fam05	Gluck-Thaler and Vogan (2024)	lasthe1
teloschistes.exilis.3_tyr927		Newly-annotated lichen genomes	teloschistes.exilis.3
teloschistes.exilis.1_tyr1399		Newly-annotated lichen genomes	teloschistes.exilis.1
protoblastenia.rupestris.2_tyr1319		Newly-annotated lichen genomes	protoblastenia.rupestris.2
teloschistes.chrysophthalmus.1_tyr372		Newly-annotated lichen genomes	teloschistes.chrysophthalmus.1
phaeophyscia.rubropulchra.1_tyr3009		Newly-annotated lichen genomes	phaeophyscia.rubropulchra.1
dirinaria.frostii.1_tyr148		Newly-annotated lichen genomes	dirinaria.frostii.1
lepraria.finkii.1_tyr3231		Newly-annotated lichen genomes	lepraria.finkii.1
parmotrema.hypotropum.1_tyr2695		Newly-annotated lichen genomes	parmotrema.hypotropum.1
rhizocarpon.geographicum.1_tyr1672		Newly-annotated lichen genomes	rhizocarpon.geographicum.1
massalongia.carnosa.1_tyr897		Newly-annotated lichen genomes	massalongia.carnosa.1
porina.heterospora.1_tyr1986		Newly-annotated lichen genomes	porina.heterospora.1
psehyp1_505659	fam05	Gluck-Thaler and Vogan (2024)	psehyp1
teratosphaeriaceae.sp..1_tyr2580		Newly-annotated lichen genomes	teratosphaeriaceae.sp..1
arthoniomycete.sp..2_tyr4012		Newly-annotated lichen genomes	arthoniomycete.sp..2
cerber1_tyr3938	fam05	Gluck-Thaler and Vogan (2024)	cerber1
xylographa.bjoerkii.1_tyr2039		Newly-annotated lichen genomes	xylographa.bjoerkii.1
teratosphaeriaceae.sp..1_tyr4056		Newly-annotated lichen genomes	teratosphaeriaceae.sp..1
frisim1_TKA68777.1	fam05	Gluck-Thaler and Vogan (2024)	frisim1
zascel1_tyr10949	fam05	Gluck-Thaler and Vogan (2024)	zascel1
stereocaulon.virgatum.1_tyr1769		Newly-annotated lichen genomes	stereocaulon.virgatum.1
teloschistes.peruensis.1_tyr1355		Newly-annotated lichen genomes	teloschistes.peruensis.1

rhizocarpon.geographicum.1_tyr223		Newly-annotated lichen genomes	rhizocarpon.geographicum.1
gyalolechia.ehrenbergii.1_tyr1703		Newly-annotated lichen genomes	gyalolechia.ehrenbergii.1
phaeophyscia.hispidula.1_tyr2662		Newly-annotated lichen genomes	phaeophyscia.hispidula.1
ramalina.thrausta.1_tyr185		Newly-annotated lichen genomes	ramalina.thrausta.1
asptan1_tyr2879	fam05	Gluck-Thaler and Vogan (2024)	asptan1
aspwen1_OJJ29631.1	fam05	Gluck-Thaler and Vogan (2024)	aspwen1
aspory7_tyr2327	fam05	Gluck-Thaler and Vogan (2024)	aspory7
aspasp7_tyr614	fam05	Gluck-Thaler and Vogan (2024)	aspasp7
talcel1_GAM41794.1	fam05	Gluck-Thaler and Vogan (2024)	talcel1
stictis.urceolata.1_tyr1910		Newly-annotated lichen genomes	stictis.urceolata.1
umbilicaria.crustulosa.1_tyr1105		Newly-annotated lichen genomes	umbilicaria.crustulosa.1
muellerella.sp..1_tyr1789		Newly-annotated lichen genomes	muellerella.sp..1
endocarpon.pusillum.2_tyr2476		Newly-annotated lichen genomes	endocarpon.pusillum.2
melanina.sp..2_tyr248		Newly-annotated lichen genomes	melanina.sp..2
melanohalea.halei.1_tyr1352		Newly-annotated lichen genomes	melanohalea.halei.1
parmeliaceae.sp..18_tyr3517		Newly-annotated lichen genomes	parmeliaceae.sp..18
usnochroma.carphineum.1_tyr2888		Newly-annotated lichen genomes	usnochroma.carphineum.1
lecanorales.sp..2_tyr388		Newly-annotated lichen genomes	lecanorales.sp..2
xanthoparmelia.tasmanica.1_tyr1678		Newly-annotated lichen genomes	xanthoparmelia.tasmanica.1
lobaria.pulmonaria.1_tyr474		Newly-annotated lichen genomes	lobaria.pulmonaria.1
pertusaria.ostiolata.1_tyr3886		Newly-annotated lichen genomes	pertusaria.ostiolata.1
ramalina.siliquosa.1_tyr3421		Newly-annotated lichen genomes	ramalina.siliquosa.1
lepra.amara.1_tyr2907		Newly-annotated lichen genomes	lepra.amara.1
baeomycetales.sp..2_tyr2485		Newly-annotated lichen genomes	baeomycetales.sp..2
pertusaria.andersoniae.1_tyr225		Newly-annotated lichen genomes	pertusaria.andersoniae.1
lecanorales.sp..2_tyr529		Newly-annotated lichen genomes	lecanorales.sp..2

lecanora.hybocarpa.1_tyr1670	Newly-annotated lichen genomes	lecanora.hybocarpa.1
xanthoria.parietina.3_tyr4037	Newly-annotated lichen genomes	xanthoria.parietina.3
flavoplaca.navasiana.2_tyr1859	Newly-annotated lichen genomes	flavoplaca.navasiana.2
xanthoparmelia.verrucella.1_tyr1823	Newly-annotated lichen genomes	xanthoparmelia.verrucella.1
variospora.velana.1_tyr718	Newly-annotated lichen genomes	variospora.velana.1
botryolepraria.lesdainii.1_tyr3146	Newly-annotated lichen genomes	botryolepraria.lesdainii.1
xylographa.vitiligo.3_tyr1675	Newly-annotated lichen genomes	xylographa.vitiligo.3
lecanorales.sp..5_tyr2453	Newly-annotated lichen genomes	lecanorales.sp..5
heterodermia.langdoniana.1_tyr1258	Newly-annotated lichen genomes	heterodermia.langdoniana.1
leucodermia.leucomelos.1_tyr2768	Newly-annotated lichen genomes	leucodermia.leucomelos.1
heterodermia.sp..1_tyr3905	Newly-annotated lichen genomes	heterodermia.sp..1
loekoesia.sp..1_tyr2499	Newly-annotated lichen genomes	loekoesia.sp..1
zwackhia.viridis.1_tyr2925	Newly-annotated lichen genomes	zwackhia.viridis.1
umbilicaria.polyphylla.4_tyr1573	Newly-annotated lichen genomes	umbilicaria.polyphylla.4
lecanoromycetidae.sp..1_tyr127	Newly-annotated lichen genomes	lecanoromycetidae.sp..1
icmadophila.ericetorum.1_tyr3762	Newly-annotated lichen genomes	icmadophila.ericetorum.1
icmadophila.ericetorum.1_tyr3161	Newly-annotated lichen genomes	icmadophila.ericetorum.1
umbilicaria.phaea.2_tyr434	Newly-annotated lichen genomes	umbilicaria.phaea.2
umbilicaria.phaea.3_tyr1623	Newly-annotated lichen genomes	umbilicaria.phaea.3
dibaeis.baeomyces.1_tyr557	Newly-annotated lichen genomes	dibaeis.baeomyces.1
flavoplaca.marina.2_tyr642	Newly-annotated lichen genomes	flavoplaca.marina.2
teratosphaeriaceae.sp..1_tyr1571	Newly-annotated lichen genomes	teratosphaeriaceae.sp..1
xylographa.bjoerkii.1_tyr2510	Newly-annotated lichen genomes	xylographa.bjoerkii.1
xylographa.soralifera.1_tyr654	Newly-annotated lichen genomes	xylographa.soralifera.1
ptychographa.xylographoides.1_tyr1363	Newly-annotated lichen genomes	ptychographa.xylographoides.1
xanthoria.parietina.3_tyr2910	Newly-annotated lichen genomes	xanthoria.parietina.3

xanthoria.parietina.3_tyr1370	Newly-annotated lichen genomes	xanthoria.parietina.3
umbilicaria.phaea.3_tyr2049	Newly-annotated lichen genomes	umbilicaria.phaea.3
lecanora.masana.1_tyr3081	Newly-annotated lichen genomes	lecanora.masana.1
cladonia.sp..1_tyr781	Newly-annotated lichen genomes	cladonia.sp..1
cladonia.sp..11_tyr794	Newly-annotated lichen genomes	cladonia.sp..11
leprocaulon.nicholsiae.1_tyr3065	Newly-annotated lichen genomes	leprocaulon.nicholsiae.1
umbilicaria.muhlenbergii.1_tyr4001	Newly-annotated lichen genomes	umbilicaria.muhlenbergii.1
umbilicaria.americana.1_tyr1621	Newly-annotated lichen genomes	umbilicaria.americana.1
umbilicaria.spodochroa.3_tyr4111	Newly-annotated lichen genomes	umbilicaria.spodochroa.3
umbilicaria.crustulosa.2_tyr632	Newly-annotated lichen genomes	umbilicaria.crustulosa.2
umbilicaria.spodochroa.4_tyr384	Newly-annotated lichen genomes	umbilicaria.spodochroa.4
umbilicaria.phaea.1_tyr3806	Newly-annotated lichen genomes	umbilicaria.phaea.1
umbilicaria.crustulosa.3_tyr1110	Newly-annotated lichen genomes	umbilicaria.crustulosa.3
baeomycetales.sp..2_tyr423	Newly-annotated lichen genomes	baeomycetales.sp..2
pertusaria.propinqua.1_tyr292	Newly-annotated lichen genomes	pertusaria.propinqua.1
calogaya.arnoldii.1_tyr823	Newly-annotated lichen genomes	calogaya.arnoldii.1
pertusaria.epixantha.1_tyr2831	Newly-annotated lichen genomes	pertusaria.epixantha.1
heterodermia.sp..3_tyr1517	Newly-annotated lichen genomes	heterodermia.sp..3
physciaceae.sp..4_tyr3794	Newly-annotated lichen genomes	physciaceae.sp..4
dibaeis.sorediata.1_tyr503	Newly-annotated lichen genomes	dibaeis.sorediata.1
vulpicida.juniperinus.1_tyr1524	Newly-annotated lichen genomes	vulpicida.juniperinus.1
platismatia.glauca.3_tyr315	Newly-annotated lichen genomes	platismatia.glauca.3
aspicilia.laevata.1_tyr1038	Newly-annotated lichen genomes	aspicilia.laevata.1
xanthoria.mediterranea.1_tyr2442	Newly-annotated lichen genomes	xanthoria.mediterranea.1
xylographa.pallens.1_tyr2912	Newly-annotated lichen genomes	xylographa.pallens.1
umbilicaria.crustulosa.2_tyr771	Newly-annotated lichen genomes	umbilicaria.crustulosa.2

umbilicaria.freyi.3_tyr3882	Newly-annotated lichen genomes	umbilicaria.freyi.3
buellia.spuria.2_tyr979	Newly-annotated lichen genomes	buellia.spuria.2
lasallia.pustulata.3_tyr1316	Newly-annotated lichen genomes	lasallia.pustulata.3
xanthoparmelia.taractica.1_tyr2241	Newly-annotated lichen genomes	xanthoparmelia.taractica.1
lepraria.leprolomopsis.1_tyr3692	Newly-annotated lichen genomes	lepraria.leprolomopsis.1
umbilicaria.muhlenbergii.1_tyr4000	Newly-annotated lichen genomes	umbilicaria.muhlenbergii.1
lecanoromycetes.sp..3_tyr4150	Newly-annotated lichen genomes	lecanoromycetes.sp..3
physciaceae.sp..4_tyr1788	Newly-annotated lichen genomes	physciaceae.sp..4
stereocaulon.virgatum.1_tyr2237	Newly-annotated lichen genomes	stereocaulon.virgatum.1
imshaugia.aleurites.2_tyr1734	Newly-annotated lichen genomes	imshaugia.aleurites.2
parmotrema.hypotropum.1_tyr1608	Newly-annotated lichen genomes	parmotrema.hypotropum.1
lepra.amara.1_tyr1662	Newly-annotated lichen genomes	lepra.amara.1
buellia.mamillana.1_tyr3147	Newly-annotated lichen genomes	buellia.mamillana.1
collema.furfuraceum.1_tyr1343	Newly-annotated lichen genomes	collema.furfuraceum.1
lobaria.pulmonaria.1_tyr2226	Newly-annotated lichen genomes	lobaria.pulmonaria.1
lecidea.sp..1_tyr3010	Newly-annotated lichen genomes	lecidea.sp..1
stictis.urceolata.2_tyr2769	Newly-annotated lichen genomes	stictis.urceolata.2
dibaeis.sorediata.1_tyr947	Newly-annotated lichen genomes	dibaeis.sorediata.1
acarospora.socialis.1_tyr3129	Newly-annotated lichen genomes	acarospora.socialis.1
leucodecton.sp..1_tyr3879	Newly-annotated lichen genomes	leucodecton.sp..1
buellia.spuria.2_tyr789	Newly-annotated lichen genomes	buellia.spuria.2
lecidea.sp..1_tyr2797	Newly-annotated lichen genomes	lecidea.sp..1
phlyctis.boliviensis.1_tyr3281	Newly-annotated lichen genomes	phlyctis.boliviensis.1
endocarpon.pusillum.1_tyr617	Newly-annotated lichen genomes	endocarpon.pusillum.1
porpidia.contraponenda.1_tyr2424	Newly-annotated lichen genomes	porpidia.contraponenda.1
cornicularia.normoerica.2_tyr2093	Newly-annotated lichen genomes	cornicularia.normoerica.2

fuscopannaria.leucosticta.1_tyr966	Newly-annotated lichen genomes	fuscopannaria.leucosticta.1
cladonia.sp..5_tyr3258	Newly-annotated lichen genomes	cladonia.sp..5
pyrenodesmia.sp..1_tyr594	Newly-annotated lichen genomes	pyrenodesmia.sp..1
umbilicaria.mammulata.1_tyr624	Newly-annotated lichen genomes	umbilicaria.mammulata.1
micareopsis.irriguata.1_tyr3669	Newly-annotated lichen genomes	micareopsis.irriguata.1
cladonia.peziziformis.1_tyr751	Newly-annotated lichen genomes	cladonia.peziziformis.1
umbilicaria.crustulosa.1_tyr446	Newly-annotated lichen genomes	umbilicaria.crustulosa.1
cladonia.uncialis.3_tyr2968	Newly-annotated lichen genomes	cladonia.uncialis.3
pilophorus.fibula.1_tyr2273	Newly-annotated lichen genomes	pilophorus.fibula.1
umbilicaria.deusta.2_tyr762	Newly-annotated lichen genomes	umbilicaria.deusta.2
lecidea.tessellata.1_tyr2260	Newly-annotated lichen genomes	lecidea.tessellata.1
porpidia.subsimplex.1_tyr2619	Newly-annotated lichen genomes	porpidia.subsimplex.1
peltigera.hydrothyria.1_tyr591	Newly-annotated lichen genomes	peltigera.hydrothyria.1
lambiella.insularis.1_tyr2786	Newly-annotated lichen genomes	lambiella.insularis.1
xylographa.soralifera.1_tyr502	Newly-annotated lichen genomes	xylographa.soralifera.1
xanthoria.parietina.3_tyr4166	Newly-annotated lichen genomes	xanthoria.parietina.3
paracladophialophora.sp..1_tyr1473	Newly-annotated lichen genomes	paracladophialophora.sp..1
chaetothyriales.sp..1_tyr1264	Newly-annotated lichen genomes	chaetothyriales.sp..1
xanthoria.parietina.3_tyr2100	Newly-annotated lichen genomes	xanthoria.parietina.3
cladophialophora.sp..1_tyr1004	Newly-annotated lichen genomes	cladophialophora.sp..1
cladophialophora.sp..1_tyr4136	Newly-annotated lichen genomes	cladophialophora.sp..1
cladophialophora.sp..2_tyr400	Newly-annotated lichen genomes	cladophialophora.sp..2
lambiella.insularis.1_tyr1952	Newly-annotated lichen genomes	lambiella.insularis.1
micareopsis.irriguata.1_tyr4069	Newly-annotated lichen genomes	micareopsis.irriguata.1
cladonia.macilenta.1_tyr1721	Newly-annotated lichen genomes	cladonia.macilenta.1
pertusaria.obruta.1_tyr1248	Newly-annotated lichen genomes	pertusaria.obruta.1

porpidia.albocaerulescens.1_tyr3319	Newly-annotated lichen genomes	porpidia.albocaerulescens.1
fissurina.insidiosa.1_tyr1217	Newly-annotated lichen genomes	fissurina.insidiosa.1
pilophorus.fibula.1_tyr3577	Newly-annotated lichen genomes	pilophorus.fibula.1
caloplaca.ligustica.1_tyr294	Newly-annotated lichen genomes	caloplaca.ligustica.1
pyxine.sorediata.1_tyr2651	Newly-annotated lichen genomes	pyxine.sorediata.1
pannaria.tavaresii.1_tyr3625	Newly-annotated lichen genomes	pannaria.tavaresii.1
kuettlingeria.erythrocarpa.1_tyr2630	Newly-annotated lichen genomes	kuettlingeria.erythrocarpa.1
fissurina.insidiosa.1_tyr3001	Newly-annotated lichen genomes	fissurina.insidiosa.1
flavoplaca.austrocitrina.1_tyr1793	Newly-annotated lichen genomes	flavoplaca.austrocitrina.1
hypocenomyce.scalaris.3_tyr182	Newly-annotated lichen genomes	hypocenomyce.scalaris.3
gyalecta.farlowii.1_tyr1838	Newly-annotated lichen genomes	gyalecta.farlowii.1
baeomycetales.sp..2_tyr4053	Newly-annotated lichen genomes	baeomycetales.sp..2
buellia.spuria.2_tyr29	Newly-annotated lichen genomes	buellia.spuria.2
acanthothecis.fontana.1_tyr3737	Newly-annotated lichen genomes	acanthothecis.fontana.1
pseudocyphellaria.aurata.1_tyr2535	Newly-annotated lichen genomes	pseudocyphellaria.aurata.1
variospora.aurantia.1_tyr252	Newly-annotated lichen genomes	variospora.aurantia.1
porpidia.subsimplex.1_tyr3856	Newly-annotated lichen genomes	porpidia.subsimplex.1
pertusaria.obruta.1_tyr1756	Newly-annotated lichen genomes	pertusaria.obruta.1
caloplaca.aegaea.1_tyr3005	Newly-annotated lichen genomes	caloplaca.aegaea.1
dendriscocaulon.intricatulum.1_tyr934	Newly-annotated lichen genomes	dendriscocaulon.intricatulum.1
coniocarpon.cinnabarinum.1_tyr383	Newly-annotated lichen genomes	coniocarpon.cinnabarinum.1
arthonia.susa.1_tyr48	Newly-annotated lichen genomes	arthonia.susa.1
arthothelium.ruanum.1_tyr3382	Newly-annotated lichen genomes	arthothelium.ruanum.1
lecidea.sp..1_tyr3029	Newly-annotated lichen genomes	lecidea.sp..1
variospora.velana.1_tyr1969	Newly-annotated lichen genomes	variospora.velana.1
leprocaulales.sp..1_tyr100	Newly-annotated lichen genomes	leprocaulales.sp..1

xanthoria.parietina.2_tyr4027	Newly-annotated lichen genomes	xanthoria.parietina.2
dermiscellum.oulocheilum.1_tyr3978	Newly-annotated lichen genomes	dermiscellum.oulocheilum.1
mycoblastus.sanguinarius.1_tyr3252	Newly-annotated lichen genomes	mycoblastus.sanguinarius.1
umbilicaria.hispanica.2_tyr338	Newly-annotated lichen genomes	umbilicaria.hispanica.2
mycoblastus.sanguinarius.2_tyr1893	Newly-annotated lichen genomes	mycoblastus.sanguinarius.2
protoblastenia.rupestris.2_tyr3986	Newly-annotated lichen genomes	protoblastenia.rupestris.2
lecanora.oreinoides.1_tyr2196	Newly-annotated lichen genomes	lecanora.oreinoides.1
lepraria.neglecta.1_tyr2664	Newly-annotated lichen genomes	lepraria.neglecta.1
mycoblastus.sanguinarius.2_tyr3955	Newly-annotated lichen genomes	mycoblastus.sanguinarius.2
chaetothyriales.sp..1_tyr953	Newly-annotated lichen genomes	chaetothyriales.sp..1
gyalolechia.fulgens.1_tyr1661	Newly-annotated lichen genomes	gyalolechia.fulgens.1
gyalolechia.sp..2_tyr1934	Newly-annotated lichen genomes	gyalolechia.sp..2
cetrelia.chicitae.1_tyr2618	Newly-annotated lichen genomes	cetrelia.chicitae.1
variospora.aurantia.1_tyr838	Newly-annotated lichen genomes	variospora.aurantia.1
umbilicaria.hispanica.3_tyr2281	Newly-annotated lichen genomes	umbilicaria.hispanica.3
umbilicaria.spodochroa.1_tyr3402	Newly-annotated lichen genomes	umbilicaria.spodochroa.1
xanthoria.parietina.3_tyr2404	Newly-annotated lichen genomes	xanthoria.parietina.3
dermiscellum.oulocheilum.1_tyr302	Newly-annotated lichen genomes	dermiscellum.oulocheilum.1
cladophialophora.sp..1_tyr3153	Newly-annotated lichen genomes	cladophialophora.sp..1
lecidea.sp..1_tyr1765	Newly-annotated lichen genomes	lecidea.sp..1
umbilicaria.vellea.1_tyr1155	Newly-annotated lichen genomes	umbilicaria.vellea.1
umbilicaria.polyphylla.1_tyr3046	Newly-annotated lichen genomes	umbilicaria.polyphylla.1
umbilicaria.freyi.1_tyr1075	Newly-annotated lichen genomes	umbilicaria.freyi.1
umbilicaria.polyphylla.4_tyr437	Newly-annotated lichen genomes	umbilicaria.polyphylla.4
umbilicaria.polyphylla.1_tyr3044	Newly-annotated lichen genomes	umbilicaria.polyphylla.1
umbilicaria.spodochroa.2_tyr2687	Newly-annotated lichen genomes	umbilicaria.spodochroa.2

parmotrema.rampoddense.1_tyr1744	Newly-annotated lichen genomes	parmotrema.rampoddense.1
mycoblastus.sanguinarius.2_tyr2714	Newly-annotated lichen genomes	mycoblastus.sanguinarius.2
leucodermia.appalachiensis.1_tyr1199	Newly-annotated lichen genomes	leucodermia.appalachiensis.1
melanelia.culbersonii.1_tyr1605	Newly-annotated lichen genomes	melanelia.culbersonii.1
lecanora.strobilina.1_tyr2168	Newly-annotated lichen genomes	lecanora.strobilina.1
umbilicaria.crustulosa.1_tyr445	Newly-annotated lichen genomes	umbilicaria.crustulosa.1
cladonia.sp..8_tyr3200	Newly-annotated lichen genomes	cladonia.sp..8
stereocaulon.dactylophyllum.1_tyr3962	Newly-annotated lichen genomes	stereocaulon.dactylophyllum.1
cladonia.strepsilis.1_tyr4046	Newly-annotated lichen genomes	cladonia.strepsilis.1
puttea.exsequens.1_tyr868	Newly-annotated lichen genomes	puttea.exsequens.1
caloplaca.sp..2_tyr1916	Newly-annotated lichen genomes	caloplaca.sp..2
cladonia.kurokawae.1_tyr1067	Newly-annotated lichen genomes	cladonia.kurokawae.1
parmotrema.rampoddense.1_tyr589	Newly-annotated lichen genomes	parmotrema.rampoddense.1
stictis.urceolata.2_tyr2530	Newly-annotated lichen genomes	stictis.urceolata.2
porpidia.macrocarpa.1_tyr313	Newly-annotated lichen genomes	porpidia.macrocarpa.1
bacidia.gigantensis.1_tyr2857	Newly-annotated lichen genomes	bacidia.gigantensis.1
usnea.ceratina.2_tyr3511	Newly-annotated lichen genomes	usnea.ceratina.2
micareopsis.irriguata.1_tyr1350	Newly-annotated lichen genomes	micareopsis.irriguata.1
gomphillus.americanus.1_tyr3818	Newly-annotated lichen genomes	gomphillus.americanus.1
umbilicaria.muhlenbergii.1_tyr4002	Newly-annotated lichen genomes	umbilicaria.muhlenbergii.1
phlyctis.petraea.1_tyr677	Newly-annotated lichen genomes	phlyctis.petraea.1
pectenia.cyanoloma.1_tyr365	Newly-annotated lichen genomes	pectenia.cyanoloma.1
xanthoria.parietina.3_tyr2487	Newly-annotated lichen genomes	xanthoria.parietina.3
phlyctis.speirea.1_tyr496	Newly-annotated lichen genomes	phlyctis.speirea.1
stictis.urceolata.1_tyr3647	Newly-annotated lichen genomes	stictis.urceolata.1
gyalolechia.sp..1_tyr1287	Newly-annotated lichen genomes	gyalolechia.sp..1

gyalolechia.sp..2_tyr2980		Newly-annotated lichen genomes	gyalolechia.sp..2
xanthoria.steineri.1_tyr3649		Newly-annotated lichen genomes	xanthoria.steineri.1
calogaya.arnoldii.1_tyr3150		Newly-annotated lichen genomes	calogaya.arnoldii.1
teloschistes.exilis.3_tyr1430		Newly-annotated lichen genomes	teloschistes.exilis.3
teloschistes.exilis.2_tyr3635		Newly-annotated lichen genomes	teloschistes.exilis.2
letharia.lupina.1_tyr4140		Newly-annotated lichen genomes	letharia.lupina.1
letharia.vulpina.1_tyr3504		Newly-annotated lichen genomes	letharia.vulpina.1
agyrium.rufum.1_tyr1892		Newly-annotated lichen genomes	agyrium.rufum.1
dibaeis.baeomyces.1_tyr240		Newly-annotated lichen genomes	dibaeis.baeomyces.1
teratosphaeriaceae.sp..1_tyr521		Newly-annotated lichen genomes	teratosphaeriaceae.sp..1
mycosphaerellales.sp..1_tyr2340		Newly-annotated lichen genomes	mycosphaerellales.sp..1
diploschistes.diacapsis.1_tyr3119		Newly-annotated lichen genomes	diploschistes.diacapsis.1
lecanorales.sp..3_tyr2207		Newly-annotated lichen genomes	lecanorales.sp..3
usnea.cornuta.1_tyr715		Newly-annotated lichen genomes	usnea.cornuta.1
teloschistes.chrysophthalmus.1_tyr1428		Newly-annotated lichen genomes	teloschistes.chrysophthalmus.1
stereocaulon.virgatum.1_tyr769		Newly-annotated lichen genomes	stereocaulon.virgatum.1
teloschistes.exilis.1_tyr3137		Newly-annotated lichen genomes	teloschistes.exilis.1
lecanoromycetes.sp..5_tyr3128		Newly-annotated lichen genomes	lecanoromycetes.sp..5
icmadophila.ericetorum.1_tyr1628		Newly-annotated lichen genomes	icmadophila.ericetorum.1
dibbae1_5584_5585	n.d.	Gluck-Thaler and Vogan (2024)	dibbae1
acanthothecis.fontana.1_tyr566		Newly-annotated lichen genomes	acanthothecis.fontana.1
lambiella.insularis.1_tyr1955		Newly-annotated lichen genomes	lambiella.insularis.1
rhizocarpon.subgeminatum.1_tyr1956		Newly-annotated lichen genomes	rhizocarpon.subgeminatum.1
thelotrema.lepadinum.2_tyr3246		Newly-annotated lichen genomes	thelotrema.lepadinum.2
grascr1_687	clade01	Gluck-Thaler and Vogan (2024)	grascr1
flavoplaca.marina.2_tyr1563		Newly-annotated lichen genomes	flavoplaca.marina.2

xanthoria.parietina.3_tyr3143		Newly-annotated lichen genomes	xanthoria.parietina.3
XANPAGTX0501_001716-T1		Tangerine element from X. parietina	X. parietina GTX0501
teloschistes.exilis.1_tyr919		Newly-annotated lichen genomes	teloschistes.exilis.1
teloschistes.peruensis.1_tyr1136		Newly-annotated lichen genomes	teloschistes.peruensis.1
teloschistes.exilis.2_tyr1488		Newly-annotated lichen genomes	teloschistes.exilis.2
teloschistes.exilis.1_tyr2639		Newly-annotated lichen genomes	teloschistes.exilis.1
teloschistes.exilis.3_tyr2393		Newly-annotated lichen genomes	teloschistes.exilis.3
stictis.urceolata.1_tyr1442		Newly-annotated lichen genomes	stictis.urceolata.1
umbpus1_103454	clade01	Gluck-Thaler and Vogan (2024)	umbpus1
umbilicaria.iberica.2_tyr712		Newly-annotated lichen genomes	umbilicaria.iberica.2
lasallia.pustulata.7_tyr1272		Newly-annotated lichen genomes	lasallia.pustulata.7
porina.heterospora.1_tyr2130		Newly-annotated lichen genomes	porina.heterospora.1
arthoniomycete.sp..1_tyr3894		Newly-annotated lichen genomes	arthoniomycete.sp..1
arthoniomycete.sp..1_tyr3896		Newly-annotated lichen genomes	arthoniomycete.sp..1
arthonia.rubella.1_tyr714		Newly-annotated lichen genomes	arthonia.rubella.1
opegrapha.moroziana.1_tyr1878		Newly-annotated lichen genomes	opegrapha.moroziana.1
cloaqu1_tyr4087	fam04	Gluck-Thaler and Vogan (2024)	cloaqu1
polfus1_606100	fam04	Gluck-Thaler and Vogan (2024)	polfus1
polfus1_599705	fam04	Gluck-Thaler and Vogan (2024)	polfus1
cengeo1_542205	fam04	Gluck-Thaler and Vogan (2024)	cengeo1
leppal1_OCK79459.1	fam04	Gluck-Thaler and Vogan (2024)	leppal1
moncan1_RYO85021.1	fam04	Gluck-Thaler and Vogan (2024)	moncan1
amnlig1_424037	fam04	Gluck-Thaler and Vogan (2024)	amnlig1
pletra1_KAF2844130.1	fam04	Gluck-Thaler and Vogan (2024)	pletra1
lizemp1_186564	fam04	Gluck-Thaler and Vogan (2024)	lizemp1
didker1_KAF3049100.1	fam04	Gluck-Thaler and Vogan (2024)	didker1

cladophialophora.endolichena.1_tyr8		Newly-annotated lichen genomes	cladophialophora.endolichena.1
claban1_134700	fam04	Gluck-Thaler and Vogan (2024)	claban1
melanina.sp..2_tyr2115		Newly-annotated lichen genomes	melanina.sp..2
psora.testacea.1_tyr44		Newly-annotated lichen genomes	psora.testacea.1
chrysothrix.sp..1_tyr942		Newly-annotated lichen genomes	chrysothrix.sp..1
punctelia.appalachensis.1_tyr1715		Newly-annotated lichen genomes	punctelia.appalachensis.1
lecanoromycetes.sp..4_tyr2353		Newly-annotated lichen genomes	lecanoromycetes.sp..4
xanthoparmelia.tasmanica.1_tyr1273		Newly-annotated lichen genomes	xanthoparmelia.tasmanica.1
xanthoparmelia.taractica.1_tyr3903		Newly-annotated lichen genomes	xanthoparmelia.taractica.1
madmyc1_9294	fam04	Gluck-Thaler and Vogan (2024)	madmyc1
stacha2_KFA51963.1	fam04	Gluck-Thaler and Vogan (2024)	stacha2
plecuc2_353822	fam04	Gluck-Thaler and Vogan (2024)	plecuc2
verdah2_tyr10676	fam04	Gluck-Thaler and Vogan (2024)	verdah2
madmyc1_9889	fam04	Gluck-Thaler and Vogan (2024)	madmyc1
neodit1_2170	fam04	Gluck-Thaler and Vogan (2024)	neodit1
hirrho1_KAH0968669.1	fam04	Gluck-Thaler and Vogan (2024)	hirrho1
fussub5_tyr6719	fam04	Gluck-Thaler and Vogan (2024)	fussub5
monsp.2_RYP32009.1	fam04	Gluck-Thaler and Vogan (2024)	monsp.2
cadmal1_KAG4411212.1	fam04	Gluck-Thaler and Vogan (2024)	cadmal1
sclcep1_KAF7866863.1	fam04	Gluck-Thaler and Vogan (2024)	sclcep1
sclcep1_KAF7870603.1	fam04	Gluck-Thaler and Vogan (2024)	sclcep1
psesp.14_KFY31966.1	fam04	Gluck-Thaler and Vogan (2024)	psesp.14
psesp.14_tyr9380	fam04	Gluck-Thaler and Vogan (2024)	psesp.14
micbol1_109359	fam04	Gluck-Thaler and Vogan (2024)	micbol1
helsp.4_KAE8442194.1	fam04	Gluck-Thaler and Vogan (2024)	helsp.4
xanthoparmelia.taractica.1_tyr115		Newly-annotated lichen genomes	xanthoparmelia.taractica.1

parmeliaceae.sp..17_tyr228		Newly-annotated lichen genomes	parmeliaceae.sp..17
pseudephebe.minuscula.1_tyr2975		Newly-annotated lichen genomes	pseudephebe.minuscula.1
usnea.aurantiacoatra.1_tyr2546		Newly-annotated lichen genomes	usnea.aurantiacoatra.1
xanthoparmelia.tasmanica.1_tyr326		Newly-annotated lichen genomes	xanthoparmelia.tasmanica.1
punctelia.appalachensis.1_tyr2301		Newly-annotated lichen genomes	punctelia.appalachensis.1
lasallia.pustulata.1_tyr486		Newly-annotated lichen genomes	lasallia.pustulata.1
umbilicaria.freyi.1_tyr3376		Newly-annotated lichen genomes	umbilicaria.freyi.1
umbilicaria.vellea.1_tyr2822		Newly-annotated lichen genomes	umbilicaria.vellea.1
umbilicaria.grisea.1_tyr989		Newly-annotated lichen genomes	umbilicaria.grisea.1
lasallia.pustulata.3_tyr2307		Newly-annotated lichen genomes	lasallia.pustulata.3
umbilicaria.hispanica.1_tyr1613		Newly-annotated lichen genomes	umbilicaria.hispanica.1
umbilicaria.iberica.1_tyr4018		Newly-annotated lichen genomes	umbilicaria.iberica.1
umbilicaria.iberica.1_tyr2561		Newly-annotated lichen genomes	umbilicaria.iberica.1
umbilicaria.iberica.2_tyr1863		Newly-annotated lichen genomes	umbilicaria.iberica.2
lasallia.papulosa.1_tyr3891		Newly-annotated lichen genomes	lasallia.papulosa.1
lasallia.pustulata.7_tyr318		Newly-annotated lichen genomes	lasallia.pustulata.7
umbilicaria.spodochroa.1_tyr820		Newly-annotated lichen genomes	umbilicaria.spodochroa.1
umbilicaria.spodochroa.2_tyr1539		Newly-annotated lichen genomes	umbilicaria.spodochroa.2
daccio1_otherdc0103420	fam03	Gluck-Thaler and Vogan (2024)	daccio1
orboli2_tyr8545	fam03	Gluck-Thaler and Vogan (2024)	orboli2
orboli2_TGJ70084.1	fam03	Gluck-Thaler and Vogan (2024)	orboli2
coniocarpon.cinnabarinum.1_tyr1072		Newly-annotated lichen genomes	coniocarpon.cinnabarinum.1
coniocarpon.cinnabarinum.2_tyr2144		Newly-annotated lichen genomes	coniocarpon.cinnabarinum.2
zymtri5_SMR62249.1	fam03	Gluck-Thaler and Vogan (2024)	zymtri5
aspfis5_tyr1178	fam03	Gluck-Thaler and Vogan (2024)	aspfis5
aspcro1_tyr1048	fam03	Gluck-Thaler and Vogan (2024)	aspcro1

aspind3_tyr1556	fam03	Gluck-Thaler and Vogan (2024)	aspind3
aspgla1_OJJ87743.1	fam03	Gluck-Thaler and Vogan (2024)	aspgla1
penfla1_2705	fam03	Gluck-Thaler and Vogan (2024)	penfla1
emmcre2_KKZ67376.1	fam03	Gluck-Thaler and Vogan (2024)	emmcre2
hiscap4_QSS56076.1	fam03	Gluck-Thaler and Vogan (2024)	hiscap4
penfla1_tyr8805	fam03	Gluck-Thaler and Vogan (2024)	penfla1
lambiella.insularis.1_tyr164		Newly-annotated lichen genomes	lambiella.insularis.1
acarospora.sp..1_tyr498		Newly-annotated lichen genomes	acarospora.sp..1
schaereria.dolodes.1_tyr788		Newly-annotated lichen genomes	schaereria.dolodes.1
muellerella.sp..1_tyr1325		Newly-annotated lichen genomes	muellerella.sp..1
lambiella.insularis.1_tyr2656		Newly-annotated lichen genomes	lambiella.insularis.1
hetspe1_CAF9941352.1	clade01	Gluck-Thaler and Vogan (2024)	hetspe1
polyblastidium.squamulosum.1_tyr1700		Newly-annotated lichen genomes	polyblastidium.squamulosum.1
heterodermia.obscurata.1_tyr2316		Newly-annotated lichen genomes	heterodermia.obscurata.1
heterodermia.speciosa.2_tyr3678		Newly-annotated lichen genomes	heterodermia.speciosa.2
umbilicaria.spodochroa.4_tyr606		Newly-annotated lichen genomes	umbilicaria.spodochroa.4
umbilicaria.deusta.4_tyr867		Newly-annotated lichen genomes	umbilicaria.deusta.4
umbilicaria.hispanica.4_tyr2253		Newly-annotated lichen genomes	umbilicaria.hispanica.4
umbilicaria.polyphylla.1_tyr3045		Newly-annotated lichen genomes	umbilicaria.polyphylla.1
umbilicaria.iberica.2_tyr3685		Newly-annotated lichen genomes	umbilicaria.iberica.2
umbilicaria.crustulosa.1_tyr1415		Newly-annotated lichen genomes	umbilicaria.crustulosa.1
umbilicaria.hispanica.4_tyr3652		Newly-annotated lichen genomes	umbilicaria.hispanica.4
umbilicaria.grisea.2_tyr2585		Newly-annotated lichen genomes	umbilicaria.grisea.2
umbilicaria.hispanica.2_tyr2692		Newly-annotated lichen genomes	umbilicaria.hispanica.2
trapelia.coarctata.1_tyr1301		Newly-annotated lichen genomes	trapelia.coarctata.1
icmadophila.ericetorum.1_tyr2978		Newly-annotated lichen genomes	icmadophila.ericetorum.1

blaper1_675	fam03	Gluck-Thaler and Vogan (2024)	blaper1
blaper1_5882	fam03	Gluck-Thaler and Vogan (2024)	blaper1
blaper1_6736	fam03	Gluck-Thaler and Vogan (2024)	blaper1
parlut1_5370	fam03	Gluck-Thaler and Vogan (2024)	parlut1
cocpos5_KMM73308.1	fam03	Gluck-Thaler and Vogan (2024)	cocpos5
blaper1_4528	fam03	Gluck-Thaler and Vogan (2024)	blaper1
wilmik1_113864	fam03	Gluck-Thaler and Vogan (2024)	wilmik1
tubmag1_tyr10581	clade01	Gluck-Thaler and Vogan (2024)	tubmag1
choven1_786032	clade01	Gluck-Thaler and Vogan (2024)	choven1
trihyb1_897299	clade01	Gluck-Thaler and Vogan (2024)	trihyb1
Starship-1_Mimp-Captain	clade01	Gluck-Thaler and Vogan (2024)	
Starship-1_Ooli-Captain	clade01	Gluck-Thaler and Vogan (2024)	
porpidia.macrocarpa.1_tyr3690		Newly-annotated lichen genomes	porpidia.macrocarpa.1
placynthium.petersii.1_tyr3666		Newly-annotated lichen genomes	placynthium.petersii.1
coniocarpon.cinnabarinum.2_tyr1581		Newly-annotated lichen genomes	coniocarpon.cinnabarinum.2
icmadophila.ericetorum.1_tyr1705		Newly-annotated lichen genomes	icmadophila.ericetorum.1
ptychographa.xylographoides.1_tyr2152		Newly-annotated lichen genomes	ptychographa.xylographoides.1
botacl1_KAF7955521.1	fam01	Gluck-Thaler and Vogan (2024)	botacl1
psesp.4_tyr9585	fam01	Gluck-Thaler and Vogan (2024)	psesp.4
psedes1_tyr9258	fam01	Gluck-Thaler and Vogan (2024)	psedes1
circinaria.calcarea.1_tyr556		Newly-annotated lichen genomes	circinaria.calcarea.1
monfru1_KAA8565543.1	fam01	Gluck-Thaler and Vogan (2024)	monfru1
rutsp.2_PQE09269.1	fam01	Gluck-Thaler and Vogan (2024)	rutsp.2
sclcep1_KAF7865656.1	fam01	Gluck-Thaler and Vogan (2024)	sclcep1
rutsp.1_PQE28656.1	fam01	Gluck-Thaler and Vogan (2024)	rutsp.1
xylographa.soralifera.1_tyr1475		Newly-annotated lichen genomes	xylographa.soralifera.1

phamin1_EOO01307.1	fam01	Gluck-Thaler and Vogan (2024)	phamin1
metrob3_EFZ00634.1	fam01	Gluck-Thaler and Vogan (2024)	metrob3
hirmin1_5623	fam01	Gluck-Thaler and Vogan (2024)	hirmin1
metani1_5588	fam01	Gluck-Thaler and Vogan (2024)	metani1
stachl1_3593	fam01	Gluck-Thaler and Vogan (2024)	stachl1
peltigera.hydrothyria.1_tyr3337		Newly-annotated lichen genomes	peltigera.hydrothyria.1
lecanora.atra.1_tyr1534		Newly-annotated lichen genomes	lecanora.atra.1
monfru1_KAA8574662.1	fam01	Gluck-Thaler and Vogan (2024)	monfru1
umbilicaria.spodochroa.2_tyr3569		Newly-annotated lichen genomes	umbilicaria.spodochroa.2
solorina.crocea.1_tyr419		Newly-annotated lichen genomes	solorina.crocea.1
acarospora.sp..1_tyr2916		Newly-annotated lichen genomes	acarospora.sp..1
monlax1_KAB8302114.1	fam01	Gluck-Thaler and Vogan (2024)	monlax1
scylig1_RFU28318.1	fam01	Gluck-Thaler and Vogan (2024)	scylig1
psesp.15_KFY65136.1	fam01	Gluck-Thaler and Vogan (2024)	psesp.15
fuscom2_tyr5400	fam01	Gluck-Thaler and Vogan (2024)	fuscom2
fusxyl4_KAG5801712.1	fam01	Gluck-Thaler and Vogan (2024)	fusxyl4
fusoxy42_ENH71189.1	fam01	Gluck-Thaler and Vogan (2024)	fusoxy42
fuscir3_tyr5364	fam01	Gluck-Thaler and Vogan (2024)	fuscir3
fusalb2_tyr5195	fam01	Gluck-Thaler and Vogan (2024)	fusalb2
moncan1_RYO82366.1	fam01	Gluck-Thaler and Vogan (2024)	moncan1
fusvan1_EEU40131.1	fam01	Gluck-Thaler and Vogan (2024)	fusvan1
metgui1_4539	fam01	Gluck-Thaler and Vogan (2024)	metgui1
coltof2_tyr4412	fam01	Gluck-Thaler and Vogan (2024)	coltof2
kredeu2_tyr7716	fam01	Gluck-Thaler and Vogan (2024)	kredeu2
dalgra1_438190	fam01	Gluck-Thaler and Vogan (2024)	dalgra1
crypar1_KAF3763523.1	fam01	Gluck-Thaler and Vogan (2024)	crypar1

spoins1_661	fam01	Gluck-Thaler and Vogan (2024)	spoins1
dimelaena.oreina.1_tyr177		Newly-annotated lichen genomes	dimelaena.oreina.1
anzia.colpodes.1_tyr2549		Newly-annotated lichen genomes	anzia.colpodes.1
rusear1_1273445	fam01	Gluck-Thaler and Vogan (2024)	rusear1
hypsp.2_644004	fam01	Gluck-Thaler and Vogan (2024)	hypsp.2
icmadophila.ericetorum.1_tyr1831		Newly-annotated lichen genomes	icmadophila.ericetorum.1
caloplaca.ligustica.1_tyr3435		Newly-annotated lichen genomes	caloplaca.ligustica.1
monsp.2_RYP22820.1	fam01	Gluck-Thaler and Vogan (2024)	monsp.2
monsp.3_RYP20312.1	fam01	Gluck-Thaler and Vogan (2024)	monsp.3
monsp.2_RYP34851.1	fam01	Gluck-Thaler and Vogan (2024)	monsp.2
moncan1_tyr8276	fam01	Gluck-Thaler and Vogan (2024)	moncan1
fusbur1_tyr5341	fam01	Gluck-Thaler and Vogan (2024)	fusbur1
spoins1_7186	fam01	Gluck-Thaler and Vogan (2024)	spoins1
fustri4_tyr6766	fam01	Gluck-Thaler and Vogan (2024)	fustri4
fusgad1_KAF4947046.1	fam01	Gluck-Thaler and Vogan (2024)	fusgad1
monibe1_RYO96122.1	fam01	Gluck-Thaler and Vogan (2024)	monibe1
triham1_3351	fam01	Gluck-Thaler and Vogan (2024)	triham1
triaru1_7814	fam01	Gluck-Thaler and Vogan (2024)	triaru1
valmal3_tyr10655	fam01	Gluck-Thaler and Vogan (2024)	valmal3
mictri1_377953	fam01	Gluck-Thaler and Vogan (2024)	mictri1
fusoxy45_KAG7413195.1	fam01	Gluck-Thaler and Vogan (2024)	fusoxy45
fusbra1_tyr5309	fam01	Gluck-Thaler and Vogan (2024)	fusbra1
clorhi1_VUC06060.1	fam01	Gluck-Thaler and Vogan (2024)	clorhi1
fusfus24_tyr5659	fam01	Gluck-Thaler and Vogan (2024)	fusfus24
dalsp.1_361367	fam01	Gluck-Thaler and Vogan (2024)	dalsp.1
dacmac1_34260	fam01	Gluck-Thaler and Vogan (2024)	dacmac1

colsoj1_KAF6812414.1	fam01	Gluck-Thaler and Vogan (2024)	colsoj1
crydep1_ODN97795.1	fam01	Gluck-Thaler and Vogan (2024)	crydep1
golmag1_tyr7146	fam01	Gluck-Thaler and Vogan (2024)	golmag1
leclec1_6749	fam01	Gluck-Thaler and Vogan (2024)	leclec1
mictri1_300110	fam01	Gluck-Thaler and Vogan (2024)	mictri1
micbol1_226188	fam01	Gluck-Thaler and Vogan (2024)	micbol1
phlyctis.petraea.1_tyr3242		Newly-annotated lichen genomes	phlyctis.petraea.1
fissurina.insidiosa.1_tyr235		Newly-annotated lichen genomes	fissurina.insidiosa.1
stictis.urceolata.1_tyr4160		Newly-annotated lichen genomes	stictis.urceolata.1
pertusaria.obruta.1_tyr2294		Newly-annotated lichen genomes	pertusaria.obruta.1
pertusaria.ostiolata.1_tyr2776		Newly-annotated lichen genomes	pertusaria.ostiolata.1
pertusaria.propinqua.1_tyr3578		Newly-annotated lichen genomes	pertusaria.propinqua.1
cladonia.sp..5_tyr4013		Newly-annotated lichen genomes	cladonia.sp..5
brigantiaea.leucoxantha.2_tyr4135		Newly-annotated lichen genomes	brigantiaea.leucoxantha.2
umbilicaria.freyi.3_tyr815		Newly-annotated lichen genomes	umbilicaria.freyi.3
parpol1_tyr8651	fam01	Gluck-Thaler and Vogan (2024)	parpol1
lepsp.1_739939	fam01	Gluck-Thaler and Vogan (2024)	lepsp.1
zalvar1_689006	fam01	Gluck-Thaler and Vogan (2024)	zalvar1
lepsp.1_674126	fam01	Gluck-Thaler and Vogan (2024)	lepsp.1
psedes1_5938	fam01	Gluck-Thaler and Vogan (2024)	psedes1
psesp.13_KFX93134.1	fam01	Gluck-Thaler and Vogan (2024)	psesp.13
psesp.13_tyr9350	fam01	Gluck-Thaler and Vogan (2024)	psesp.13
parpol1_tyr8652	fam01	Gluck-Thaler and Vogan (2024)	parpol1
rhesp.1_195900	fam01	Gluck-Thaler and Vogan (2024)	rhesp.1
parpol1_3559	fam01	Gluck-Thaler and Vogan (2024)	parpol1
phisub1_tyr9118	fam01	Gluck-Thaler and Vogan (2024)	phisub1

buellia.spuria.1_tyr2629		Newly-annotated lichen genomes	buellia.spuria.1
buellia.spuria.2_tyr1802		Newly-annotated lichen genomes	buellia.spuria.2
Phoenix_Mpha_mp053-Captain	fam01	Gluck-Thaler and Vogan (2024)	
rhimac1_8905	fam01	Gluck-Thaler and Vogan (2024)	rhimac1
chasp.7_RMZ87469.1	fam01	Gluck-Thaler and Vogan (2024)	chasp.7
exooli1_134015	fam01	Gluck-Thaler and Vogan (2024)	exooli1
zalvar1_685428	fam01	Gluck-Thaler and Vogan (2024)	zalvar1
endocarpon.pusillum.2_tyr310		Newly-annotated lichen genomes	endocarpon.pusillum.2
endocarpon.pusillum.1_tyr3655		Newly-annotated lichen genomes	endocarpon.pusillum.1
peltigera.aphthosa.1_tyr3865		Newly-annotated lichen genomes	peltigera.aphthosa.1
penrub2_KAF3021169.1	fam01	Gluck-Thaler and Vogan (2024)	penrub2
aspasp2_tyr552	fam01	Gluck-Thaler and Vogan (2024)	aspasp2
phyllopsora.corallina.1_tyr3776		Newly-annotated lichen genomes	phyllopsora.corallina.1
neopar1_6999	fam01	Gluck-Thaler and Vogan (2024)	neopar1
metrob3_tyr8212	fam01	Gluck-Thaler and Vogan (2024)	metrob3
toloph1_tyr10453	fam01	Gluck-Thaler and Vogan (2024)	toloph1
ilysp.2_681009	fam01	Gluck-Thaler and Vogan (2024)	ilysp.2
beabas1_10225	fam01	Gluck-Thaler and Vogan (2024)	beabas1
podcom1_tyr9194	fam01	Gluck-Thaler and Vogan (2024)	podcom1
coniocarpon.cinnabarinum.1_tyr976		Newly-annotated lichen genomes	coniocarpon.cinnabarinum.1
porpidia.albocaerulescens.1_tyr1306		Newly-annotated lichen genomes	porpidia.albocaerulescens.1
botryolepraria.lesdainii.1_tyr4168		Newly-annotated lichen genomes	botryolepraria.lesdainii.1
cetradonia.linearis.1_tyr2739		Newly-annotated lichen genomes	cetradonia.linearis.1
xanthoria.sp..1_tyr4191		Newly-annotated lichen genomes	xanthoria.sp..1
psora.crenata.1_tyr256		Newly-annotated lichen genomes	psora.crenata.1
blaper1_5625	fam01	Gluck-Thaler and Vogan (2024)	blaper1

seirophora.lacunosa.1_tyr3068		Newly-annotated lichen genomes	seirophora.lacunosa.1
xylographa.bjoerkii.1_tyr1640		Newly-annotated lichen genomes	xylographa.bjoerkii.1
pilophorus.fibula.1_tyr173		Newly-annotated lichen genomes	pilophorus.fibula.1
mobergia.calculiformis.1_tyr3516		Newly-annotated lichen genomes	mobergia.calculiformis.1
icmadophila.ericetorum.1_tyr3968		Newly-annotated lichen genomes	icmadophila.ericetorum.1
porpidia.albocaerulescens.1_tyr3320		Newly-annotated lichen genomes	porpidia.albocaerulescens.1
phaeophyscia.squarrosa.1_tyr3355		Newly-annotated lichen genomes	phaeophyscia.squarrosa.1
lecanora.subimmergens.1_tyr3478		Newly-annotated lichen genomes	lecanora.subimmergens.1
umbilicaria.subpolyphylla.1_tyr3558		Newly-annotated lichen genomes	umbilicaria.subpolyphylla.1
placynthium.petersii.1_tyr162		Newly-annotated lichen genomes	placynthium.petersii.1
rinodina.dolichospora.1_tyr1995		Newly-annotated lichen genomes	rinodina.dolichospora.1
dermiscellum.oulocheilum.1_tyr1667		Newly-annotated lichen genomes	dermiscellum.oulocheilum.1
xanthoparmelia.verrucella.1_tyr2858		Newly-annotated lichen genomes	xanthoparmelia.verrucella.1
umbpus1_107339	fam01	Gluck-Thaler and Vogan (2024)	umbpus1
lasallia.pustulata.7_tyr1579		Newly-annotated lichen genomes	lasallia.pustulata.7
lasallia.pustulata.7_tyr3777		Newly-annotated lichen genomes	lasallia.pustulata.7
umbilicaria.nylanderiana.1_tyr505		Newly-annotated lichen genomes	umbilicaria.nylanderiana.1
bacgig1_KAG8531634.1	fam01	Gluck-Thaler and Vogan (2024)	bacgig1
teloschistes.peruensis.1_tyr2827		Newly-annotated lichen genomes	teloschistes.peruensis.1
leucodermia.leucomelos.1_tyr3140		Newly-annotated lichen genomes	leucodermia.leucomelos.1
stereocaulon.dactylophyllum.1_tyr3223		Newly-annotated lichen genomes	stereocaulon.dactylophyllum.1
phyllopsora.corallina.1_tyr703		Newly-annotated lichen genomes	phyllopsora.corallina.1
lecidea.sp..1_tyr1019		Newly-annotated lichen genomes	lecidea.sp..1
stereocaulon.alpinum.1_tyr67		Newly-annotated lichen genomes	stereocaulon.alpinum.1
platismatia.glauca.3_tyr1645		Newly-annotated lichen genomes	platismatia.glauca.3
stereocaulon.virgatum.1_tyr2063		Newly-annotated lichen genomes	stereocaulon.virgatum.1

xylographa.parallela.1_tyr39		Newly-annotated lichen genomes	xylographa.parallela.1
solorina.crocea.1_tyr2958		Newly-annotated lichen genomes	solorina.crocea.1
rinodina.dolichospora.1_tyr3024		Newly-annotated lichen genomes	rinodina.dolichospora.1
opegrapha.moroziana.1_tyr668		Newly-annotated lichen genomes	opegrapha.moroziana.1
pertusaria.ostiolata.1_tyr2466		Newly-annotated lichen genomes	pertusaria.ostiolata.1
umbilicaria.crustulosa.1_tyr1088		Newly-annotated lichen genomes	umbilicaria.crustulosa.1
aspfis5_tyr1169	fam01	Gluck-Thaler and Vogan (2024)	aspfis5
asphir2_tyr1512	fam01	Gluck-Thaler and Vogan (2024)	asphir2
asplat1_tyr1608	fam01	Gluck-Thaler and Vogan (2024)	asplat1
cladonia.squamosa.3_tyr3665		Newly-annotated lichen genomes	cladonia.squamosa.3
blaper1_4705	fam01	Gluck-Thaler and Vogan (2024)	blaper1
artunc1_KAF3492431.1	fam01	Gluck-Thaler and Vogan (2024)	artunc1
blagil1_OAT07656.1	fam01	Gluck-Thaler and Vogan (2024)	blagil1
parmin1_KAF9728395.1	fam01	Gluck-Thaler and Vogan (2024)	parmin1
zoprhi1_KAF2179529.1	fam01	Gluck-Thaler and Vogan (2024)	zoprhi1
zoprhi1_KAF2174473.1	fam01	Gluck-Thaler and Vogan (2024)	zoprhi1
pyrtri10_PZD03750.1	fam01	Gluck-Thaler and Vogan (2024)	pyrtri10
zwackhia.viridis.1_tyr611		Newly-annotated lichen genomes	zwackhia.viridis.1
virvir1_581593	fam01	Gluck-Thaler and Vogan (2024)	virvir1
friend1_TKA26544.1	clade01	Gluck-Thaler and Vogan (2024)	friend1
teratosphaeriaceae.sp..1_tyr278		Newly-annotated lichen genomes	teratosphaeriaceae.sp..1
zascel1_66988	clade01	Gluck-Thaler and Vogan (2024)	zascel1
teratosphaeriaceae.sp..1_tyr2413		Newly-annotated lichen genomes	teratosphaeriaceae.sp..1
mycosphaerellales.sp..1_tyr1726		Newly-annotated lichen genomes	mycosphaerellales.sp..1

SI References

1. B. J. Walker, *et al.*, Pilon: an integrated tool for comprehensive microbial variant detection and genome assembly improvement. *PLoS One* **9**, e112963 (2014).

2. B. Langmead, S. L. Salzberg, Fast gapped-read alignment with Bowtie 2. *Nat Methods* **9**, 357–359 (2012).

3. D. D. Kang, *et al.*, MetaBAT 2: an adaptive binning algorithm for robust and efficient genome reconstruction from metagenome assemblies. *PeerJ* **7**, e7359 (2019).

4. M. Seppey, M. Manni, E. M. Zdobnov, BUSCO: Assessing Genome Assembly and Annotation Completeness. *Methods Mol Biol* **1962**, 227–245 (2019).

5. K. Katoh, D. M. Standley, MAFFT multiple sequence alignment software version 7: improvements in performance and usability. *Mol Biol Evol* **30**, 772–780 (2013).

6. S. Capella-Gutiérrez, J. M. Silla-Martínez, T. Gabaldón, trimAl: a tool for automated alignment trimming in large-scale phylogenetic analyses. *Bioinformatics* **25**, 1972–1973 (2009).

7. B. Q. Minh, *et al.*, IQ-TREE 2: New Models and Efficient Methods for Phylogenetic Inference in the Genomic Era. *Mol Biol Evol* **37**, 1530–1534 (2020).

8. S.-Y. Park, S.-H. Jang, S.-O. Oh, J. A. Kim, J.-S. Hur, An easy, rapid, and cost-effective method for DNA extraction from various lichen taxa and specimens suitable for analysis of fungal and algal strains. *Mycobiology* **42**, 311–316 (2014).

9. G. Benoit, *et al.*, High-quality metagenome assembly from long accurate reads with metaMDBG. *Nat Biotechnol* **42**, 1378–1383 (2024).

10. H. Li, Minimap2: pairwise alignment for nucleotide sequences. *Bioinformatics* **34**, 3094–3100 (2018).

11. P. Saary, A. L. Mitchell, R. D. Finn, Estimating the quality of eukaryotic genomes recovered from metagenomic analysis with EukCC. *Genome Biol* **21**, 244 (2020).

12. C. Zhou, S. A. McCarthy, R. Durbin, YaHS: yet another Hi-C scaffolding tool. *Bioinformatics* **39** (2023).

13. G. Tagirdzhanova, *et al.*, Complexity of the lichen symbiosis revealed by metagenome and transcriptome analysis of Xanthoria parietina. *Curr Biol* **35**, 799–817.e5 (2025).

14. J. M. Palmer, J. Stajich, *Funannotate v1.8.1: Eukaryotic genome annotation* (Zenodo, 2020).

15. J. M. Flynn, *et al.*, RepeatModeler2 for automated genomic discovery of transposable element families. *Proc. Natl. Acad. Sci. U. S. A.* **117**, 9451–9457 (2020).

16. A. Lomsadze, V. Ter-Hovhannisyan, Y. O. Chernoff, M. Borodovsky, Gene identification in novel eukaryotic genomes by self-training algorithm. *Nucleic Acids Res.* **33**, 6494–6506 (2005).

17. M. Stanke, S. Waack, Gene prediction with a hidden Markov model and a new intron submodel. *Bioinformatics* **19 Suppl 2**, ii215–25 (2003).

18. A. C. Testa, J. K. Hane, S. R. Ellwood, R. P. Oliver, CodingQuarry: highly accurate hidden Markov model gene prediction in fungal genomes using RNA-seq transcripts. *BMC*

Genomics **16**, 170 (2015).

19. W. H. Majoros, M. Pertea, S. L. Salzberg, TigrScan and GlimmerHMM: two open source ab initio eukaryotic gene-finders. *Bioinformatics* **20**, 2878–2879 (2004).

20. B. J. Haas, *et al.*, Automated eukaryotic gene structure annotation using EVidenceModeler and the Program to Assemble Spliced Alignments. *Genome Biol.* **9**, R7 (2008).

21. P. P. Chan, T. M. Lowe, tRNAscan-SE: Searching for tRNA Genes in Genomic Sequences. *Methods Mol. Biol.* **1962**, 1–14 (2019).

22. J. Mistry, *et al.*, Pfam: The protein families database in 2021. *Nucleic Acids Res.* **49**, D412–D419 (2021).

23. UniProt Consortium, UniProt: the Universal Protein Knowledgebase in 2023. *Nucleic Acids Res.* **51**, D523–D531 (2023).

24. N. D. Rawlings, M. Waller, A. J. Barrett, A. Bateman, MEROPS: the database of proteolytic enzymes, their substrates and inhibitors. *Nucleic Acids Res.* **42**, D503–9 (2014).

25. Y. Yin, *et al.*, dbCAN: a web resource for automated carbohydrate-active enzyme annotation. *Nucleic Acids Res.* **40**, W445–51 (2012).

26. T. Paysan-Lafosse, *et al.*, InterPro in 2022. *Nucleic Acids Res.* **51**, D418–D427 (2023).

27. C. P. Cantalapiedra, A. Hernández-Plaza, I. Letunic, P. Bork, J. Huerta-Cepas, eggNOG-mapper v2: Functional Annotation, Orthology Assignments, and Domain Prediction at the Metagenomic Scale. *Mol. Biol. Evol.* **38**, 5825–5829 (2021).

28. T. Baril, J. Galbraith, A. Hayward, Earl Grey: A Fully Automated User-Friendly Transposable Element Annotation and Analysis Pipeline. *Mol Biol Evol* **41** (2024).

29. J. Storer, R. Hubley, J. Rosen, T. J. Wheeler, A. F. Smit, The Dfam community resource of transposable element families, sequence models, and genome annotations. *Mob DNA* **12**, 2 (2021).

30. S. van Wyk, *et al.*, The RIPper, a web-based tool for genome-wide quantification of Repeat-Induced Point (RIP) mutations. *PeerJ* **7**, e7447 (2019).

31. W. Shen, S. Le, Y. Li, F. Hu, SeqKit: A Cross-Platform and Ultrafast Toolkit for FASTA/Q File Manipulation. *PLoS One* **11**, e0163962 (2016).

32. J. T. Lovell, *et al.*, GENESPACE tracks regions of interest and gene copy number variation across multiple genomes. *Elife* **11** (2022).

33. E. Gluck-Thaler, A. A. Vogan, Systematic identification of cargo-mobilizing genetic elements reveals new dimensions of eukaryotic diversity. *Nucleic Acids Res* **52**, 5496–5513 (2024).

34. T. Hackl, M. Ankenbrand, B. van Adrichem, D. Wilkins, K. Haslinger, gggenomes: effective and versatile visualizations for comparative genomics. *arXiv [q-bio.GN]* (2024).

35. G. Marçais, *et al.*, MUMmer4: A fast and versatile genome alignment system. *PLoS Comput Biol* **14**, e1005944 (2018).

36. J. T. Robinson, H. Thorvaldsdottir, D. Turner, J. P. Mesirov, igv.js: an embeddable JavaScript implementation of the Integrative Genomics Viewer (IGV). *Bioinformatics* **39** (2023).

37. C. Camacho, *et al.*, BLAST+: architecture and applications. *BMC Bioinformatics* **10**, 421 (2009).

38. T. Llewellyn, *et al.*, Metagenomics Shines Light on the Evolution of "Sunscreen" Pigment Metabolism in the Teloschistales (Lichen-Forming Ascomycota). *Genome Biol Evol* **15** (2023).

39. A. Dobin, *et al.*, STAR: ultrafast universal RNA-seq aligner. *Bioinformatics* **29**, 15–21 (2013).

40. E. Levy Karin, M. Mirdita, J. Söding, MetaEuk-sensitive, high-throughput gene discovery, and annotation for large-scale eukaryotic metagenomics. *Microbiome* **8**, 48 (2020).

41. M. Steinegger, J. Söding, MMseqs2 enables sensitive protein sequence searching for the analysis of massive data sets. *Nat Biotechnol* **35**, 1026–1028 (2017).

42. D. Moi, *et al.*, Structural phylogenetics unravels the evolutionary diversification of communication systems in gram-positive bacteria and their viruses. *bioRxiv* (2023).

43. E. Gluck-Thaler, A. A. Vogan, Systematic identification of cargo-mobilizing genetic elements reveals new dimensions of eukaryotic diversity. *Nucleic Acids Res* **52**, 5496–5513 (2024).

44. J. Jumper, *et al.*, Highly accurate protein structure prediction with AlphaFold. *Nature* **596**, 583–589 (2021).

45. J. Abramson, *et al.*, Accurate structure prediction of biomolecular interactions with AlphaFold 3. *Nature* **630**, 493–500 (2024).

46. E. C. Meng, *et al.*, UCSF ChimeraX: Tools for structure building and analysis. *Protein Sci* **32**, e4792 (2023).

47. C. Elfmann, J. Stülke, PAE viewer: a webserver for the interactive visualization of the predicted aligned error for multimer structure predictions and crosslinks. *Nucleic Acids Res* **51**, W404–W410 (2023).

48. E. A. Gladyshev, M. Meselson, I. R. Arkhipova, Massive horizontal gene transfer in bdelloid rotifers. *Science* **320**, 1210–1213 (2008).

49. D. M. Emms, S. Kelly, OrthoFinder: phylogenetic orthology inference for comparative genomics. *Genome Biol* **20**, 238 (2019).

50. I. Letunic, P. Bork, Interactive Tree of Life (iTOL) v6: recent updates to the phylogenetic tree display and annotation tool. *Nucleic Acids Res* **52**, W78–W82 (2024).
